# Programmable Stereoregular Fully Aromatic-Substituted Polymethylenes

**DOI:** 10.1021/jacs.5c20802

**Published:** 2026-03-03

**Authors:** Jiaxi Xu, Jingjing Liu, Nikos Hadjichristidis

**Affiliations:** Polymer Synthesis Laboratory, KAUST Catalysis Center, Physical Sciences and Engineering Division, 127355 King Abdullah University of Science and Technology (KAUST), Thuwal 23955, Saudi Arabia

## Abstract

The discovery of new classes of polymers continues to redefine the scope of macromolecular chemistry. However, one category has remained largely unexplored: fully aromatic-substituted polymethylenes bearing functional groups on every backbone carbon. These polymers represent the densest functionalization achievable in carbon–carbon chain backbones but have long been regarded as synthetically inaccessible because of severe steric congestion. Furthermore, achieving stereoregularity in C1 polymerization remains challenging, as the mechanism involves an additional elimination step and is highly sensitive to steric and electronic effects. Here, we report a controlled C1 polymerization that enables the synthesis of stereoregular and functionalized poly­(phenylmethylene)­s, a fundamentally new class of carbon-chain polymers that combine fully aromatic backbone substitution with programmable tacticity. Two distinct catalytic pathways were developed: (1) a carbenium-initiated cationic polymerization affording isotactic polymers, and (2) a Ni­(acac)_2_-induced carbene polymerization affording syndiotactic polymers. Both methods produce polymers with controlled molecular weights, diverse aryl substituents, and distinct thermal, photochemical, and supramolecular characteristics. This work establishes a new structural structural framework for carbon-chain polymers that integrates functionality and stereoregularity, thus opening new avenues for designing functional materials beyond the limits of conventional polyolefin chemistry.

## Graphical abstract

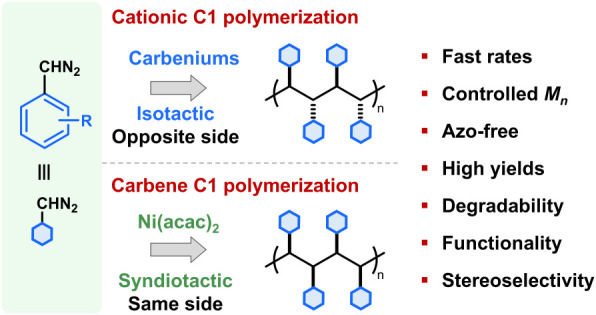

## Introduction

The discovery of new classes of polymers has continuously advanced the field of polymer chemistry. From polyolefins,^1–6^ which revolutionized commodity plastics, to conjugated polymers,^7–12^ enabling electronic and optoelectronic materials, and to degradable polyesters,^13–16^ opening sustainable pathways for materials design, each breakthrough has expanded the scope of monomer and catalyst development, (co)­polymerization strategies, structural and compositional control, and mechanistic understanding. These advances underscore the central role of creating entirely new new classes of polymers in shaping the boundaries of macromolecular chemistry and opening opportunities for future materials innovation. However, in contrast to previously established polymer categories, one fundamental structural motif has received little attention: fully substituted polymethylenes bearing functional groups on every backbone carbon (Scheme 1A). Such polymers represent the ultimate evolution of carbon–carbon main-chain design, combining dense functionality with unique chain dynamics. Their synthesis relies on C1 polymerization, which in principle enables substitution on every backbone carbon.^17–20^ However, the scope of C1 polymerization remains narrow, largely confined to unsubstituted polymethylene and carbonyl-substituted polymethylenes.^21–30^ The synthesis of fully aromatic-substituted polymethylenes has long been considered infeasible because of the severe steric congestion surrounding each methylene unit, even though it could impart rigidity, enhanced interchain interactions, and new functional opportunities.^31–35^ Previous attempts using boron trifluoride, alcohol, or palladium catalysts have yielded only low-molecular-weight products with poor control and undesired azo linkages, reinforcing the long-standing perception that such polymers are synthetically inaccessible.^36–41^

**Scheme 1:**
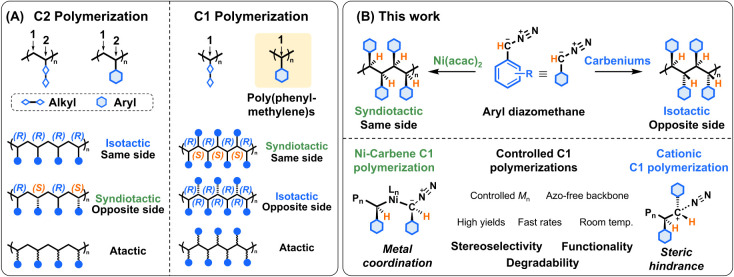
(A) Comparison of C2 Polymerization (Two-Carbon Growth) and C1 Polymerization (One-Carbon Growth). (B) Cationic Polymerization of Aryl Diazomethyl Monomers Using Carbenium Catalysts Affords Isotactic Poly­(phenylmethylene)­s, Where Ni­(acac)_2_ Produces Syndiotactic Chains

Beyond composition and functionality, stereoregularity constitutes a fundamental dimension in polymer design, governing the chain conformation and thus influencing the material properties.^42–45^ Stereoregular polymers exhibit superior thermomechanical performance compared with their stereoirregular counterparts.^42,43^ Stereoregular polymers, stereoregular polymers can be obtained either by polymerizing enantiopure monomers or through enantioselective polymerization of racemic monomers using chiral catalysts or initiators.^46–48^ In contrast, stereoregular polyolefins derived from achiral monomers arise from site control during coordination–insertion polymerization, in which the catalyst geometry dictates the orientation of successive monomer insertions.^49–51^ Unlike these systems, achieving stereoregularity in C1 polymerization of achiral monomers is considerably more complex, as the mechanism involves an additional elimination step.^18,19,52^ Moreover, steric and electronic effects often disrupt chain propagation and stereochemical control, making it extremely challenging to achieve stereoregularity in a specific direction in densely substituted or structurally congested polymers. To date, stereoregular C1 polymerizations have been realized almost exclusively in Rh- and Pd-catalyzed polymerizations of diazo esters, affording only syndiotactic polymers through chain-end control.^25,53–62^ However, these metal complexes suffer from low initiation efficiency and show poor compatibility with other diazo monomers, underscoring the narrow scope of current stereoregular C1 polymerization systems.^56,63^

Here, we report the synthesis of stereoregular and functionalized poly­(phenylmethylene)­s, representing a fundamentally new class of carbon–carbon chain polymers (Scheme 1B). Using distinct catalyst systems, we achieve high molecular weights together with programmable tacticity, while enabling the incorporation of diverse aryl substituents at every backbone carbon. Cationic polymerization of aryl diazomethyl using carbenium catalysts yields isotactic polymers, while Ni-carbene polymerization mediated by nickel acetylacetonate [Ni­(acac)_2_] affords syndiotactic chains. This unprecedented combination of backbone aromatic substitution and stereocontrol creates a versatile platform for probing structure–property relationships and for accessing functionalities that are inaccessible in conventional polyolefin chemistry. Our results establish poly­(phenylmethylene)­s as a new structural paradigm in polymer chemistry and open broad opportunities for the design of stereoregular and functional polymers.

## Results and Discussion

### C1 Polymerization of (Diazomethyl)­benzene (1)

High-purity monomer is essential for controlled polymerization. Previous syntheses of (diazomethyl)­benzene (**1**) suffer from residual methanol and inseparable impurities such as *trans*-stilbene or azine. We addressed this issue by using anhydrous dichloromethane during the precursor preparation and combining the pyrolysis step. This modification eliminates key impurities and enables the isolation of **1** in high purity without column chromatography, offering a reproducible, scalable, and functional-group-compatible route for downstream C1 polymerization (Figures S1–S94).

Controlled polymerization of **1** remains challenging, and achieving stereocontrol is even more demanding. These limitations highlight the need for a new catalytic strategy capable of simultaneously enabling activation and tacticity. Given the nucleophilic character of the diazo carbon, we envisioned that Lewis and Brønsted acids could initiate/catalyze polymerization via electrophilic attack, releasing nitrogen, and generating benzylic chain growth. We further hypothesized that the progressive phenyl substitution at the chain-end would impose steric hindrance, controlling monomer orientation such that the substituent is positioned *anti* to the growing chain. This consistent spatial arrangement favors identical chiral configurations and thus promotes isotactic sequences. Alternatively, catalyst-controlled monomer alignment during insertion could guide the formation of syndiotactic sequences.

Catalyst screening revealed that carbenium-based Lewis acids efficiently induce polymerization of **1** (Table S1). All polymerizations were conducted in toluene, chosen for its low polarity and weak coordinating ability, which minimizes interference with the propagation species. The C­(Ph)_3_^+^BF_4_^–^ and Trop^+^BF_4_^–^ triggered instantaneous polymerization, yielding polymers with *M*_n_ of 12.8 and 12.3 kg mol^–1^ (*Đ* = 1.52 and 1.44, respectively). In contrast, the less soluble C­(Ph)_3_^+^PF_6_^–^ exhibited a slightly slower kinetic (2 min) and produced polymers with higher *M*_n_, consistent with lower effective catalyst concentration and supporting the role of carbenium species as initiators. To enhance solubility, we tested a weakly coordinating carbenium C­(Ph)_3_^+^B­(C_6_F_5_)_4_^–^. Although small gas evolution indicated initial activation, no polymer formed, suggesting insufficient stabilization of the growing carbocation led to premature termination. Control experiments with Brønsted acids and the same counterions of BF_4_^–^ and PF_6_^–^ showed no polymer formation, ruling out protonation and counterions as the active event (Table S3).

Carbenium-initiated C1 polymerization proceeds with excellent control. Systematic variation of the [**1**]_0_/[C­(Ph)_3_^+^BF_4_^–^]_0_ ratio from 30/1 to 200/1 revealed an increase in *M*_n_ from 12.8 to 67.8 kg mol^–1^, consistent with controlled chain growth (Table S1, Figure 1A). Even at a 200/1 feed ratio, full monomer conversion was achieved within 5 minutes, highlighting its exceptional reactivity. The resulting polymers exhibited relatively narrow dispersities (*Đ* < 1.55), indicating well-regulated propagation. At higher monomer-to-initiator ratios, inefficient *M*_n_ growth and tailing were observed, indicative of enhanced chain-transfer and termination typical of cationic polymerization. Chain-end living was confirmed by monomer readdition experiments (Figure S250). These results firmly establish carbenium Lewis acid initiators as an effective platform for fast, controlled, and extendable C1 polymerization under mild conditions.

**Figure 1:**
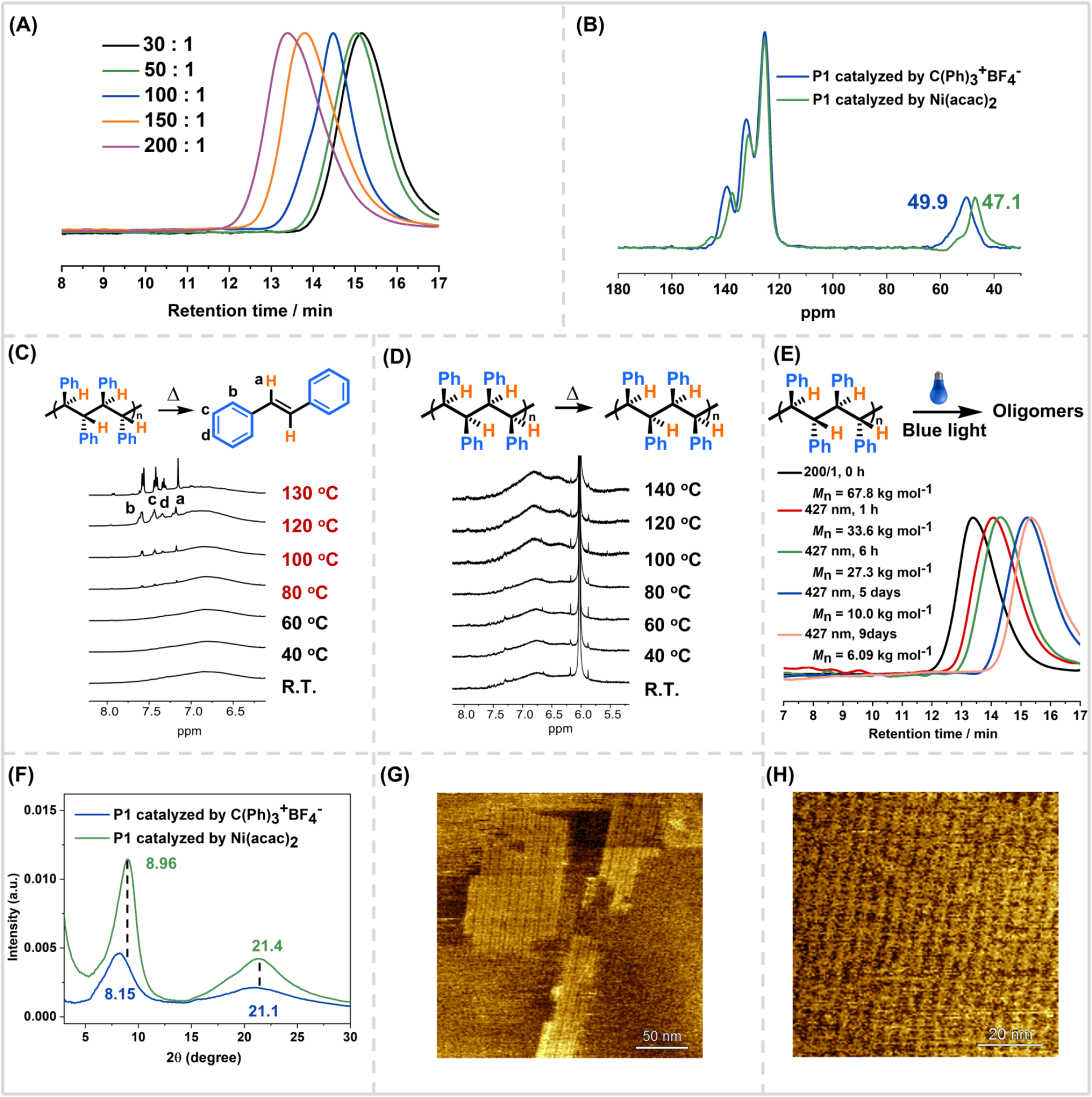
(A) SEC traces of polymer **P1** synthesized using C­(Ph)_3_^+^BF_4_^–^ as a catalyst at the different [**1**]_0_/[cat.]_0_ ratios. (B) ^13^C CP/MAS NMR spectra of **P1**. (C) *In-situ* ^1^H NMR (600 MHz, TCE-*d*_2_) spectra of **P1** catalyzed by C­(Ph)_3_^+^BF_4_^–^, collected at different temperatures. (D) *In-situ* ^1^H NMR (600 MHz, TCE-*d*_2_) spectra of **P1** catalyzed by Ni­(acac)_2_, collected at different temperatures. (E) SEC traces of polymer **P1** synthesized using C­(Ph)_3_^+^BF_4_^–^ as a catalyst ([**1**]_0_/[cat.]_0_ = 200/1, before and after UV light (427 nm). (F) WAXS pattern of polymer **P1** synthesized using C­(Ph)_3_^+^BF_4_^–^ (blue) and Ni­(acac)_2_ (green) as catalysts. (G) AFM images of **P1** catalyzed by C­(Ph)_3_^+^BF_4_^–^ on HOPG. (H) AFM images of **P1** catalyzed by Ni­(acac)_2_ on HOPG.

In contrast to chain-end steric induction for isotacticity, we envisioned that syndiotactic propagation could be achieved through coordination control. Specifically, transition-metal catalysts could preorganize the diazo monomer via coordination, guiding its orientation during each insertion. This spatial preorganization would bias the approach of incoming monomers, favoring syndiotactic sequences during chain growth.

To test our hypothesis and broaden the tunability of Lewis acidity and coordination environment, we investigated metal halides (AlCl_3_, FeCl_3_, ZnCl_2_, SnCl_4_, TiCl_4_, AuCl_3_, CuBr), known electrophilic catalysts in cationic polymerizations (Table S3). Among them, FeCl_3_, ZnCl_2_, SnCl_4_, and CuBr induced nitrogen release and full monomer consumption, but failed to generate polymer, while AlCl_3_, TiCl_4_, and AuCl_3_ showed no reactivity. These results suggest that although some metal halides can activate the diazo group, they lack the coordination dynamics to sustain chain propagation. To further explore catalytic systems capable of both activation and propagation, we extended our investigation to a broader range of transition metal complexes (Ni, Pd, Co, Au, Ru, Rh, Cu). Remarkably, only nickel­(II) acetylacetonate [Ni­(acac)_2_] afforded high-molecular-weight polymers (Tables S2 and S3). Other complexes either consumed the monomer without forming polymer, yielded complex mixtures, or remained inactive. These findings highlight Ni­(acac)_2_ as uniquely capable of coupling diazo activation with efficient propagation, enabling a metal-mediated route to C1 polymerization.

Unlike the carbenium-initiated C1 polymerizations, where the resulting polymers remained fully soluble in toluene throughout the reaction and dissolved well in common organic solvents, the polymers obtained from Ni­(acac)_2_-catalyzed C1 polymerization gradually precipitated during the reaction. The isolated polymers exhibited poor solubility in a wide range of solvents, including benzene, toluene, dichloromethane, chloroform, tetrahydrofuran, *N*,*N*-dimethylformamide, dimethyl sulfoxide, methanol, and hexafluoroisopropanol. This limited solubility complicates molecular weight analysis and hampers reliable chain-extension experiments, thereby impeding assessment of the living character of the system. This striking contrast in solubility profiles strongly suggests a fundamental difference in the microstructure or stereoregularity of the resulting polymers.

### Structural Characterization

Given the pronounced solubility differences, we hypothesized that the two polymers differ in stereoregularity. However, structural analysis by ^1^H NMR and ^13^C NMR revealed only broad and weak signals for both polymers, even at high concentrations, corresponding to aromatic and benzylic methylene groups (Figures S95 and S96). The extensive signal broadening without fine splitting, unchanged by solvent and temperature, suggests extensive phenyl stacking along the polymer backbone that averages local magnetic environments and severely suppresses fine structural resolution. Solid-state ^13^C CP/MAS NMR provided clearer structural features, showing aromatic resonances at 115–150 ppm and benzylic methylene signals at 49.9 and 47.1 ppm (Figure 1B). For the Ni­(acac)_2_-catalyzed **P1**, the benzylic methylene resonances are shifted to higher field and display narrower line shapes compared to the carbenium-derived **P1**, consistent with tighter π–π stacking of phenyl groups aligned on the same side of the backbone. Both polymers exhibit crystalline characteristics, but the Ni­(acac)_2_-derived sample shows more pronounced intrachain stacking and local ordering.

Elemental analysis showed C:H ratios of 7:5.96 and 7:6.05 for both polymers, closely matching the theoretical 7:6 (Tables S7 and S8), with negligible nitrogen content (<0.31%, attributed to air exposure), confirming complete nitrogen release and the expected poly­(phenylmethylene) structure. FTIR spectra supported a linear structure, displaying characteristic aromatic C–H and C–C vibrations and combination bands at 2000–1650 cm^–1^, consistent with monosubstituted benzene rings and excluding cross-linking (Figure S144). Notably, the Ni­(acac)_2_-derived **P1** exhibited sharper, higher-frequency sp^3^ C–H stretching at 2934 cm^–1^ (vs 2910 cm^–1^), and more distinct aromatic C–H out-of-plane and in-plane bending at 764 cm^–1^, 1354, and 1261 cm^–1^, suggesting higher phenyl symmetry, tighter packing, and back regularity. Raman spectra reinforced these distinctions (Figure S164), revealing differences in out-of-plane (776 vs 747 cm^–1^) and in-plane (1193 cm^–1^) aromatic C–H bending. These spectral features indicate that the Ni­(acac)_2_-derived polymer adopts a more syndiotactic microstructure, while the carbenium-derived polymer is likely isotactic.

### Degradation Behavior

Given the dense aromatic packing in **P1**, we hypothesized that its backbone might undergo cleavage through low-barrier vibrational activation. To test this hypothesis, variable-temperature *in situ* ^1^H NMR experiments were conducted in TCE-*d*_2_, selected for its high boiling point, which enables NMR monitoring at elevated temperature. For the carbenium-derived **P1**, heating to 80 °C led to the appearance of signals corresponding to *trans*-stilbene, which became more prominent at 130 °C (Figure 1C and Figure S135). SEC analysis confirmed a decrease in molecular weight from 12.8 to 4.0 kg mol^–1^, indicating substantial backbone scission (Figure S286). Notably, only *trans*-stilbene was observed with no evidence for *cis*-stilbene formation (Figures S137–S143), supporting the isotactic sequence. Importantly, identical degradation behavior was observed upon direct thermal treatment of the polymer in the solid state, followed by NMR analysis in CDCl_3_ at room temperature, indicating that the exclusive formation of *trans*-stilbene is intrinsic to the polymer microstructure rather than solvent-dependent. The facile cleavage is likely facilitated by the steric crowding of pendant phenyl groups, which elongates the backbone C–C bonds (approximately 1.57–1.60 Å) and lowers the energy required for bond dissociation. In contrast, Ni­(acac)_2_-derived **P1** remained intact with no detectable any stilbene generation (Figure 1D and Figure S136). This difference is attributed to the syndiotactic sequence, where strong π–π stacking among pendant phenyl groups suppresses vibrational energy transfer and enhances thermal stability. These findings are consistent with the stereochemical assignments and demonstrate that tacticity influences thermal degradability. In addition to thermal degradation, the photodegradability of both polymers was assessed under blue light irradiation (427 nm). The carbenium-derived **P1** showed a gradual molecular weight decrease, evidenced by a distinct SEC shift, indicating efficient photoinduced backbone scission (Figure 1E). In contrast, the Ni­(acac)_2_-derived **P1** remained largely intact, with only minimal degradation after 60 days of exposure (Figure S285). These observations further support the crucial influence of stereoregularity on polymer stability.

### Microstructural Analysis

Wide-angle X-ray scattering (WAXS) analysis revealed distinct chain packing behavior (Figure 1F). Both samples displayed a broad diffraction centered at about 20°, indicative of short-range packing within dense π-stacked aromatic domains, typically of polystyrene-like systems. Notably, the Ni­(acac)_2_-derived **P1** showed a sharper reflection at 2θ = 8.96° (d = 0.99 nm), while the carbenium-derived **P1** exhibited a slightly broader peak at 8.15° (d = 1.08 nm). These lattice distances (0.99 and 1.08 nm) match the cross-sectional dimension of a single helix, providing direct structural evidence of a helical chain arrangement.^64^ The higher-angle and sharper peak indicates more compact interchain interactions, stronger π–π stacking, and more uniform phenyl alignment, consistent with syndiotactic order, while the broader, lower-angle peak reflects looser, less regular packing of isotactic microstructure. These differences in long-range ordering corroborate the enhanced structural rigidity and higher thermal and photostability of the syndiotactic polymer.

AFM analysis revealed supramolecular organization dictated by tacticity. Both polymers self-assembled into 2D smectic-like helical bundles on highly oriented pyrolytic graphite (HOPG), confirming layered π-stacked architectures (Figure 1G,H). The Ni­(acac)_2_-derived **P1** formed more uniform and continuous monolayers, reflecting its higher stereoregularity and stronger interchain interactions. In contrast, the carbenium-derived **P1** exhibited weaker adhesion to the HOPG substrate and was prone to displacement during scanning, suggesting the looser packing and reduced interfacial affinity. The presence of helical domains in both cases confirms chain conformation, but their differing packing efficiency and surface stability highlight the critical role of tacticity in supramolecular organization. These findings correlate well with observed differences in solubility, spectroscopic resolution, and degradation behavior.

Circular dichroism (CD) analysis probed potential macromolecular conformations. The Ni­(acac)_2_-derived **P1** was too insoluble for reliable measurements, whereas the carbenium-derived **P1** exhibited weak but discernible CD signals (Figure S288). Since the polymer backbone contains pseudochiral rather than truly chiral centers, this optical activity likely arises from helical superstructures in solution, which restrict bond rotation and induce chiral environments.

### Proposed Mechanisms

We propose two distinct C1 polymerization mechanisms: (1) a carbenium-initiated (rearrangement-assisted) cationic propagation mechanism, and (2) a Ni­(acac)_2_-catalyzed metal-carbene propagation mechanism (Figure 2). Both afford stereocontrol but operate through distinct activation modes. In the carbenium system, [C­(Ph)_3_^+^BF_4_^–^] triggers electrophilic attack on the diazo carbon (TS1, ΔG^‡^ = 11.9 kcal mol^–1^), followed by rapid N_2_ elimination (TS2, ΔG^‡^ = 0.2 kcal mol^–1^) to form a secondary carbocation (Figures S289–S291). A rapid intramolecular phenyl 1,2-migration (TS3, ΔG^‡^ = 1.6 kcal mol^–1^) is computationally predicted to stabilize the propagating center by forming a tertiary carbenium ion. This migration is proposed to facilitate chain propagation at the early stage of polymerization; however, as steric congestion increases along the growing chain, the migration is expected to become progressively suppressed. Under these conditions, propagation is more likely to proceed via a secondary carbocation pathway. The carbenium intermediates with a planar geometry allow electrophilic attack of the diazo carbon from either the Re- or Si-face to form a transient near-tetrahedral intermediate, which then eliminates N_2_ to restore planarity. This regeneration relaxes into an *anti*-arrangement of adjacent substituents, minimizing steric congestion. This preferred geometry, calculated to be 6.6 kcal mol^–1^ more stable than the *syn* alternative, enforces an *isotactic* propagation mode. In the Ni­(acac)_2_-catalyzed pathway, ligand exchange at Ni^II^ proceeds through a dissociative interchange mechanism (TS4′, ΔG^‡^ = 18.1 kcal mol^–1^), generating a Ni–monomer adduct (Figures S292–S295). This intermediate interconverts between its rotamers due to low-energy bond rotations (TS7′, ΔG^‡^ = 2.7 kcal mol^–1^), and preferentially adopts a geometry that favors syndiotactic insertion owing to its lower energy. Subsequent N_2_ elimination (TS5′, ΔG^‡^ = 6.2 kcal mol^–1^) from the syndiotactic conformer proceeds with a lower activation barrier compared to the isotactic pathway, favoring selective formation of the Ni–carbene species in a stereoregular arrangement. This kinetic and thermodynamic preference directs the system toward syndiotactic chain growth. The resulting Ni–carbene then undergoes carbene insertion (TS6′, ΔG^‡^ = 2.4 kcal mol^–1^), retaining the syndiotactic configuration along the propagating chain.

**Figure 2:**
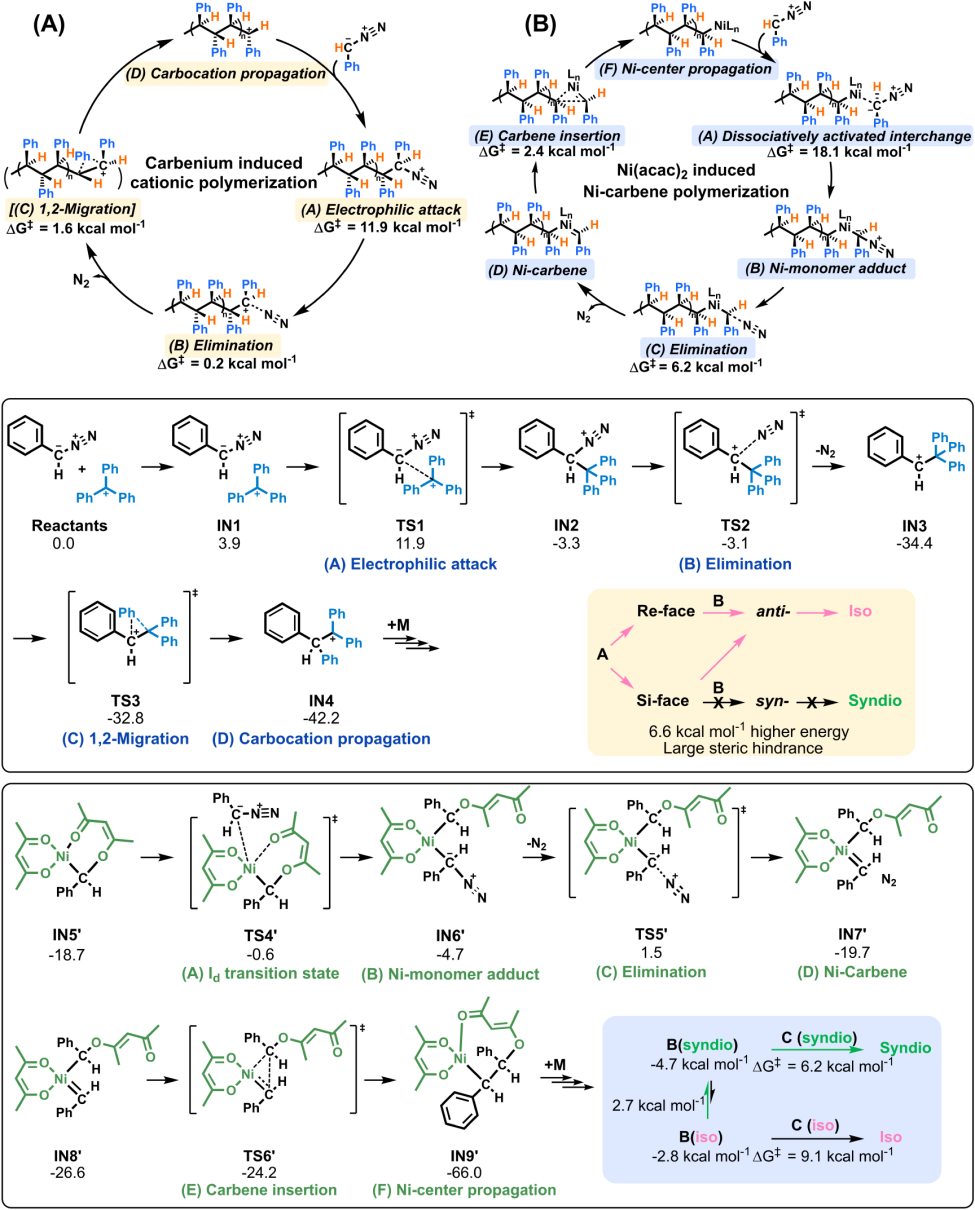
(A) Carbenium-induced C1 polymerization via electrophilic attack followed by elimination and, where applicable 1,2-migration, leading to cationic chain propagation. (B) Ni­(acac)_2_-induced C1 polymerization via a dissociatively activated interchange, elimination, and carbene insertion process, resulting in Ni-centered chain propagation.

### Functionalized Polymers

To access the scope of the two C1 polymerization strategies, 19 aryl diazomethane derivatives were synthesized via simple distillation or sublimation (Figure 3, Figures S96–S134). The C­(Ph)_3_^+^BF_4_^–^ initiated polymerization proceeded immediately across a broad range of functional groups, including halogens (F, Cl, Br), alkyl (Me, iPr, CF_3_), and esters. Notably, aryl diazomethanes bearing electron-donating substituents (e.g., *p*-Me and *p*-iPr) consistently afforded polymers (P6 and P7) with reduced dispersity, which can be attributed to enhanced stabilization of the propagating secondary carbocationic chain ends, thereby suppressing chain-transfer and termination events. Even strongly electron-withdrawing cyano and bulky naphthyl groups afforded polymers (P10 and P20), albeit with reduced molecular weights or slower rates. In contrast, Ni­(acac)_2_-catalyzed polymerizations displayed a complex dependence of rate on steric and electronic effects. Ortho-substituted monomers (Cl, Br, Me) failed to polymerize, and bulky groups such as naphthyl significantly slowed polymerization, consistent with the steric requirements of syndiotactic growth. Few electron-withdrawing substituents (e.g., *o*-F, *p*-CF_3_, *m*-CF_3_) accelerated polymerization by facilitating carbene insertion, whereas excessive electron-withdrawing substituents (*m*,*m*'-(CF_3_)_2_, C_6_F_5_, *o*-F-*m*-Br)" instead of "electron-withdrawalsuppressed the dissociative interchange step and slowed the reaction. Electron-donating groups (e.g., *p*-iPr) promoted reactivity by enhancing dissociative interchange steps. Ester substituents were tolerated but gave sluggish reaction, while cyano groups proved incompatible. These results underscore the distinct mechanistic characteristics: the cationic pathway combines broad functional-group tolerance with high rates, whereas the Ni–carbene pathway enforces tighter steric and electronic constraints, consistent with its syndiotactic propagation mechanism.

**Figure 3:**
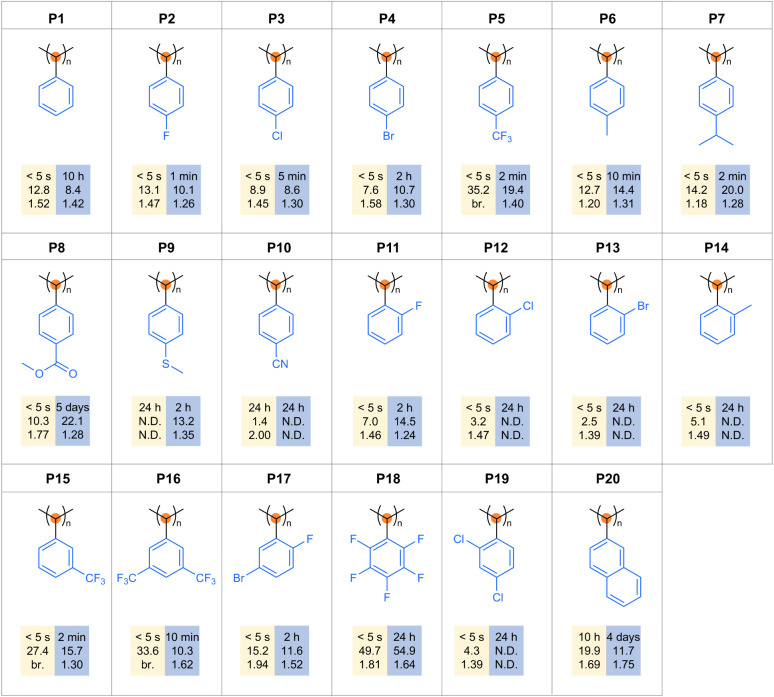
Scope of functionalized poly­(phenylmethylene)­s obtained using C­(Ph)_3_^+^BF_4_^–^ (yellow shading, conversions were detected by color disappearance) and Ni­(acac)_2_ (blue shading, conversions were detected by SEC analysis) as catalysts. The data indicate polymerization time, *M*_n,SEC_, and *Đ*.

### Thermal Properties

Thermal analysis of 20 functionalized poly­(phenylmethylene)­s revealed clear differences based on tacticity and monomer substitution (Figures S205–S245). Syndiotactic polymers from Ni­(acac)_2_ catalysis consistently exhibited higher decomposition temperatures than their isotactic counterparts from carbenium catalysts. Polymers derived from sterically hindered ortho-substitution showed lower stability, likely due to disruption of chain packing and weakened π–π stacking. Differential scanning calorimetry (DSC) detected no glass transition (*T*_g_) below 160 °C, suggesting highly restricted segmental mobility and dense local packing. These results further reinforce the correlation between stereoregularity, substitution pattern, and thermal stability in C1 polymerized poly­(phenylmethylene)­s.

## Conclusion

We have established a fundamentally new class of carbon–carbon chain polymers, stereoregular and functionalized poly­(phenylmethylene)­s, by combining backbone fully aromatic substitution with precise stereocontrol. Two distinct catalytic systems were proposed: a carbenium-initiated cationic polymerization and a Ni­(acac)_2_-induced carbene polymerization. These pathways afford isotactic and syndiotactic polymers, each exhibiting distinct thermal stability, photodegradability, chain packing, and supramolecular organization. Together, they establish a general strategy for accessing polymers with controlled molecular weight, programmable tacticity, and diverse aryl substituents, overcoming the long-standing limitations of both C2 and C1 polymerizations. This work defines a new structural paradigm for polymer chemistry, providing the platform to systematically connect stereoregularity and rich backbone functionality in carbon-chain polymers. Beyond demonstrating synthetic feasibility, these findings open broad opportunities for exploring structure–property relationships and for designing functional materials with features not accessible through conventional polyolefin chemistry.

## Supplementary Material



## Notes

Materials and methods, synthetic procedures, NMR spectra, FTIR spectra, Raman spectra, WAXS patterns, TGA curves, DSC curves, SEC traces, CD spectrum, computational details including Cartesian coordinates (PDF)
