# Supplementary material for: Programmable Stereoregular Fully Aromatic-Substituted Polymethylenes
Source: J Am Chem Soc. 2026 Mar 3;148(10):10797–805. doi: 10.1021/jacs.5c20802 (PMC13003485; doi:10.1021/jacs.5c20802)
Supplement: Supplementary file 1 [file ja5c20802_si_001.pdf]

Supporting information for

Programmable Stereoregular Fully Aromatic-Substituted Polymethylenes

Jiaxi Xu*, Jingjing Liu, and Nikos Hadjichristidis*

Corresponding authors: jiaxi.xu@kaust.edu.sa, nikolaos.hadjichristidis@kaust.edu.sa.

Polymer Synthesis Laboratory, KAUST Catalysis Center, Physical Sciences and Engineering Division, King Abdullah University of Science and Technology (KAUST); Thuwal 23955, Saudi Arabia

Table of Contents

Materials and Methods **4**

Instruments and characterizations 4

Supplementary Text **5**

General procedure for the synthesis of aryl diazomethane monomers 5

General polymerization procedures 6

AFM measurements 6

Monomer syntheses 7

Supplementary Tables **13**

Table S1. C1 polymerization of (diazomethyl)benzene (**1**) initiated by carbeniums. 13

Table S2. C1 polymerization of (diazomethyl)benzene (**1**) initiated by Ni complexes. 14

Table S3. C1 polymerization of (diazomethyl)benzene (**1**) initiated by other catalysts. 15

Table S4. C1 polymerization of functional diazo monomers initiated by $C(Ph)_3BF_4^-$. 18

Table S5. C1 polymerization of functional diazo monomers initiated by $Ni(acac)_2$. 20

Table S6. C1 polymerization of (diazomethyl)benzene (**1**) initiated by $C(Ph)_3BF_4^-$ with MeOH. 21

Table S7. Elemental analysis results of polymers synthesized using $C(Ph)_3BF_4^-$ as a catalyst. 21

Table S8. Elemental analysis results of polymers synthesized using $Ni(acac)_2$ as a catalyst 21

Supplementary Figures **23**

Fig. S1-S94. ^{1}H, ^{13}C, and ^{19}F NMR spectra of monomers (**1-20**) and their precursors. 23

Figs. S95-S134. ^{1}H and ^{13}C NMR spectra of **P1-P20**. 70

Fig. S135. *In-situ* ^{1}H NMR (600 MHz, TCE-d_2) spectra of **P1** catalyzed by $C(Ph)_3^+BF_4^-$, collected during a stepwise temperature increase from R.T. to 130 °C. 90

Fig. S136. *In-situ* ^{1}H NMR (600 MHz, TCE-d_2) spectra of **P1** catalyzed by $Ni(acac)_2$, collected during a stepwise temperature increase from R.T. to 140 °C. 90

Figs. S137-138. ^{1}H and ^{13}C NMR (TCE-d_2, 23 °C) spectrum of **P1** using $C(Ph)_3^+BF_4^-$ after heating. 91

Figs. S139-140. ^{1}H and ^{13}C NMR ($CDCl_3$, 23 °C) spectrum of *trans*-stilbene. 92

Figs. S141-142. ^{1}H and ^{13}C NMR ($CDCl_3$, 23 °C) spectrum of *cis*-stilbene 93

Fig. S143. ^{1}H NMR (400 MHz, $CDCl_3$, 23 °C) spectra of monomer **1** recorded at different time intervals at room temperature. 94

Figs. S144-163. FTIR spectra of polymers **P1-P20** catalyzed by $C(Ph)_3^+BF_4^-$ and $Ni(acac)_2$. 94

Figs. S164-183. Raman spectra polymers **P1-P20** catalyzed by $C(Ph)_3^+BF_4^-$ and $Ni(acac)_2$ with a 633 nm excitation wavelength. 104

Fig. S184. Raman spectra polymer **P1** catalyzed by $C(Ph)_3^+BF_4^-$ and $Ni(acac)_2$ with a 473 nm excitation wavelength. 114

Figs. S185-204. WAXS pattern of polymers **P1-P20** catalyzed by $C(Ph)_3^+BF_4^-$ and $Ni(acac)_2$. 115

Fig. S205. TGA curves of polymer **P1** catalyzed by $C(Ph)_3^+BF_4^-$ and *trans*-stilbene. 125

Figs. S206-225. TGA curves of polymers **P1-P20** catalyzed by $C(Ph)_3^+BF_4^-$ and $Ni(acac)_2$. 125

Figs. S226-245. DSC curves of polymers **P1-P20** catalyzed by $C(Ph)_3^+BF_4^-$ and $Ni(acac)_2$ 135

Figs. S246-248. SEC traces of polymer **P1** catalyzed by carbeniums 142

Fig. S249. SEC traces of polymers **P1** catalyzed by $C(Ph)_3^+BF_4^-$ at the different [**1**]/[cat.] ratios. 144

Fig. S250. SEC traces of polymer **P1** catalyzed by $C(Ph)_3^+BF_4^-$, before and after chain extension. 144

Figs. S251-268. SEC traces of polymers **P1-P20** catalyzed by $C(Ph)_3^+BF_4^-$. 145

Figs. S269-283. SEC traces of polymers **P1-P20** catalyzed by $Ni(acac)_2$. 154

Fig. S284. SEC traces of polymer **P1** catalyzed by $C(Ph)_3^+BF_4^-$, before and after UV light (427 nm) irradiation for different durations. 161

Fig. S285. SEC traces of polymer **P1** catalyzed by $Ni(acac)_2$, before and after UV light (427 nm) irradiation for different durations. 162

Fig. S286. SEC traces of polymer **P1** catalyzed by $C(Ph)_3^+BF_4^-$ after heating at 130 ^oC. 162

Fig. S287. Photoluminescent (PL) spectra of polymer **P1** catalyzed by $C(Ph)_3^+BF_4^-$ (blue) and $Ni(acac)_2$ (green) (λ_{ex} = 320 nm). 163

Fig. S288. CD spectrum of polymer **P1** catalyzed by $C(Ph)_3^+BF_4^-$. 163

Figs. S289-S291. Proposed mechanism and DFT-calculated results for the C1 polymerization of monomer **1** catalyzed by $C(Ph)_3^+BF_4^-$.. 165

Figs. S292-295. Proposed mechanism and DFT-calculated results for the C1 polymerization of monomer **1** catalyzed by $Ni(acac)_2$.. 170

References 326

Materials and Methods

All polymerization operations were conducted in flamed Schlenk-type glassware under an inert argon atmosphere or within an argon-filled glovebox. Toluene (Tol) was dried by refluxing over sodium and further purified by distillation from *n*-butyllithium under nitrogen. Dichloromethane (DCM) was purified by twofold distillation over calcium hydride (CaH_2) under nitrogen. Methanol was dried over activated 3 Å molecular sieves, followed by distillation under nitrogen. Sodium methoxide was freshly prepared from sodium metal and anhydrous methanol under a nitrogen atmosphere, and subsequently dried under high vacuum at 80 °C for 3 days before use. Other chemicals were purchased from Aldrich Chemicals and used as received unless stated otherwise.

Instruments and characterizations

NMR spectroscopy. ^1H NMR and ^{13}C NMR spectra were recorded at 25 ^oC on Bruker AVANCE III-400 MHz instruments. *In-situ* NMR measurements were performed on Bruker AVANCE III-600 MHz instruments. Solid-State Nuclear Magnetic Resonance Spectroscopy. One-dimensional ^1H MAS and ^{13}C CP/MAS solid state NMR spectra were recorded on Bruker AVANCE Neo spectrometers operating at 400 resonance frequencies for ^1H. Experiments at 400 MHz employed a conventional double-resonance 4 mm CP/MAS double-resonance probe. Dry nitrogen gas was utilized for sample spinning to prevent degradation of the samples. NMR chemical shifts are reported with respect to the external references TMS and adamantane. For ^{13}C CP/MAS NMR experiments, the following sequence was used: 90° pulse on the proton (pulse length 2.4 s), then a cross-polarization step with a contact time of typically 2 ms, and finally acquisition of the ^{13}C signal under high-power proton decoupling. The delay between the scans was set to 5 s to allow the complete relaxation of the ^1H nuclei, and the number of scans ranged between 1024 and 2048 for ^{13}C and was 32 for ^1H. An exponential apodization function corresponding to a line broadening of 80 Hz was applied prior to the Fourier transformation.

Size exclusion chromatography (SEC). SEC analyses were conducted on an Agilent 1260 Infinity SEC system equipped with an Agilent 1260 Infinity autosampler, refractive index detector, and PSS columns (one guard column and two PLgel 10 μm mixed-B 7.5 × 300 mm columns). THF was used as the eluent at 30 °C (1.0 mL min^{-1}), and the system was calibrated with monodisperse polystyrene standards.

Fourier-transform infrared spectroscopy (FTIR). FTIR spectra were collected using a Thermo Fisher NICOLET iS10 spectrometer.

Raman spectroscopy. Raman measurements were performed on a WITec apyron confocal Raman microscope (WITec GmbH, Germany) with a 633 nm excitation laser and a 50× objective under ambient conditions.

Thermal gravimetric analysis (TGA). TGA was conducted using a TA Instruments Q500 analyzer under nitrogen flow. Samples were heated from 25 °C to 850 °C at a heating rate of 10 °C min^{-1}.

Differential scanning calorimetry (DSC). DSC measurements were performed using a TA Instruments Discovery DSC. Samples sealed in hermetic aluminum pans were first cooled to −20 °C, then heated to 180 °C at 10 °C min^{-1} under a nitrogen atmosphere to erase thermal history. A second heating cycle was used to determine the glass transition temperature (T_g).

Wide-angle X-ray scattering (WAXS). WAXS experiments were performed at 25 °C on a XENOCS Xeuss 3.0 SAXS/WAXS system equipped with dual Cu Genix3D X-ray sources and an Eiger2 4M detector.

Atomic force microscopy (AFM). AFM imaging was performed in fluid tapping mode using a Bruker Dimension Icon equipped with a ScanAsyst-Fluid+ probe. Samples were imaged in water at room temperature with a scan rate of 1.0 Hz.

UV−visible spectroscopy. UV−visible absorption spectra were recoded using a UV–vis spectrophotometer with a quartz cuvette (path length: 10 mm).

Photoluminescence spectroscopy. Photoluminescence spectra were obtained on a Thermo Lumina fluorescence spectrometer under ambient conditions.

Supplementary Text

General procedure for the synthesis of aryl diazomethane monomers

(1)
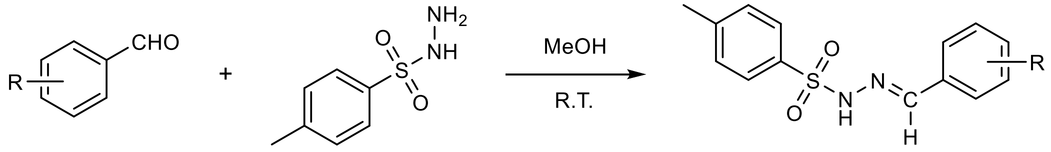

(2)
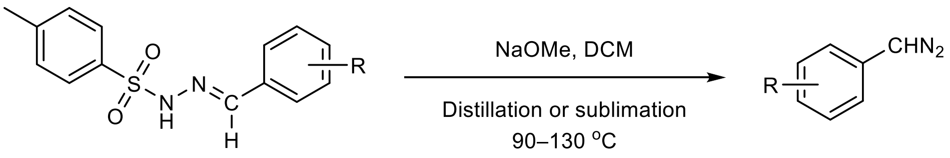

Based on a literature procedure, the benzaldehyde derivative (0.055 mol, 1.10 equiv.) was added to a solution of *p*-toluenesulfonyl hydrazide (0.050 mol, 1.00 equiv.) in methanol (30 mL) at room temperature[1]. If the benzaldehyde derivative was a solid, it was first dissolved in methanol before addition; the liquid benzaldehyde derivative was added directly. The resulting mixture was stirred at room temperature, during which a white or light-yellow solid gradually precipitated. The reaction progress was monitored by thin-layer chromatography (TLC), and upon complete consumption of the starting materials, the solid was collected by filtration. The precipitate was washed with cold diethyl ether (or cold methanol) and dried under reduced pressure to afford the corresponding *p*-toluenesulfonyl phenylhydrazone derivative.

The dried *p*-toluenesulfonyl phenylhydrazone derivative (4.92 mmol, 1.00 equiv.) was dissolved in dry dichloromethane (30 mL), followed by the addition of sodium methoxide (9.84 mmol, 2.00 equiv.). A white solid formed, and the mixture was allowed to stand at room temperature for 1–2 hours, during which the solid gradually turned pale pink. The solvent was then removed under reduced pressure, and the resulting solid was dried overnight under vacuum at room temperature. The dried precursor was heated in an oil bath at 90–130 ^{o}C under oil pump vacuum, and the volatile product was collected in a receiving flask at –20°C to afford pure aryl diazomethane.

Caution: Diazo compounds are potentially explosive, light-sensitive, and toxic. All manipulations should be performed behind a blast shield, in a well-ventilated hood, and under light-protected conditions.[2]

General polymerization procedures

Polymerizations were carried out in a nitrogen-filled glovebox at room temperature. A 20 mL vial was charged with a specified amount of catalyst and anhydrous toluene and stirred for 5 h. Polymerization was initiated by the rapid addition of a 1.0 M monomer solution in anhydrous toluene to the catalyst solution, resulting in a final monomer concentration of 0.2 M. After the desired reaction time, the reaction was quenched by exposure to the mixture to air. The product was precipitated into 50 mL of cold methanol under stirring, filtered, and washed with cold methanol to remove unreacted monomer. The polymer was dried under vacuum at room temperature for 24 h to a constant weight. The obtained polymers are intrinsically brittle and fracture readily under mild mechanical stress.

AFM measurements

Stock solutions of the **P1** polymers, synthesized using either $C(Ph)_3^+BF_4^-$ or $Ni(acac)_2$ as the catalyst, were prepared in benzene at concentrations of 0.0005 and 0.04 mg mL^{-1}, respectively. For AFM measurements, one drop of each polymer solution was cast onto a fresh cleaved highly oriented pyrolytic graphite (HOPG) substrate at room temperature. After air-drying, the HOPG

substrates were further exposed to benzene vapor in a sealed 50 mL flask containing a 2 mL vial with 1 mL of benzene for 1 h to promote self-assembly. The samples were then dried under vacuum for 2 h. A ScanAsyst-Fluid+ probe was employed, and all imaging was conducted in water at room temperature. HOPG was obtained from Bruker Co. Ltd.

Monomer syntheses

(Diazomethyl)benzene (1)

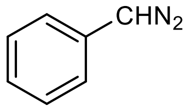

The resulting *(E)-N'*-benzylidene-4-methylbenzenesulfonohydrazide was obtained as a white solid in 55% yield (R_f = 0.27 in $CHCl_3$). ^{1}H NMR (400 MHz, $CDCl_3$): δ 8.65 (s, 1H), 7.91 (d, 2H), 7.81 (s, 1H), 7.56 (d, 2H), 7.33-7.27 (m, 5H), 2.37 (s, 3H). ^{13}C NMR (100 MHz, $CDCl_3$): δ 148.1, 144.3, 135.2, 133.3, 130.4, 129.8, 128.6, 128.0, 127.4, 21.6. (Diazomethyl)benzene was obtained as a blood-red liquid by vacuum distillation. ^{1}H NMR (400 MHz, $CDCl_3$): δ 7.32 (m, 2H), 7.07 (m, 1H), 6.96 (m, 2H), 4.97 (s, 1H). ^{13}C NMR (100 MHz, $CDCl_3$): δ 129.8, 129.1, 123.9, 121.3, 47.2.

1-(Diazomethyl)-4-fluorobenzene (2)

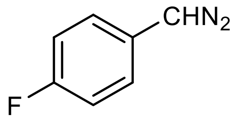

The resulting (*E*)-*N*'-(4-fluorobenzylidene)-4-methylbenzenesulfonohydrazide was obtained as a colorless crystal in 51% yield (R_f = 0.17 in $CHCl_3$). ^{1}H NMR (400 MHz, $CDCl_3$): δ 8.53 (s, 1H), 7.90 (d, *J* = 8.2 Hz, 2H), 7.80 (s, 1H), 7.60-7.51 (m, 2H), 7.33 (d, *J* = 8.2 Hz, 2H), 7.03 (t, *J* = 8.7 Hz, 2H), 2.42 (s, 3H). ^{13}C NMR (100 MHz, $CDCl_3$): δ 165.2, 162.7, 146.8, 144.4, 135.2, 129.8, 129.5(2), 129.3(2), 127.9, 115.9(2), 21.6. ^{19}F NMR (377 MHz. $CDCl_3$): δ -109.3. 1-(Diazomethyl)-4-fluorobenzene was obtained as a dark red liquid by vacuum distillation. ^{1}H NMR (400 MHz, $CDCl_3$): δ 7.13-6.75 (m, 4H), 4.93 (s, 1H). ^{13}C NMR (100 MHz. $CDCl_3$): δ 160.9, 159.3, 125.4, 122.5(2), 116.3(2), 46.7. ^{19}F NMR (377 MHz. $CDCl_3$): δ -119.4.

1-Chloro-4-(diazomethyl)benzene (3)

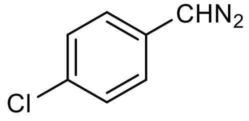

The resulting (*E*)-*N*'-(4-chlorobenzylidene)-4-methylbenzenesulfonohydrazide was obtained as a white solid in 63% yield (R_f = 0.14 in $CHCl_3$). ^{1}H NMR (400 MHz, $CDCl_3$): δ 8.14 (br, 1H), 7.89 (d, *J* = 8.4 Hz, 2H), 7.75 (s, 1H), 7.52 (d, *J* = 8.6 Hz, 2H), 7.34 (d, *J* = 8.5 Hz, 4H), 2.43 (s, 3H). ^{13}C NMR (100 MHz, $CDCl_3$): δ 146.4, 144.5, 136.4, 135.2, 131.7, 129.8, 129.0, 128.5, 127.9, 21.6. 1-Chloro-4-(diazomethyl)benzene was obtained as a dark red liquid by vacuum distillation. ^{1}H NMR (400 MHz, $CDCl_3$): δ 7.26 (d, *J* = 8.7 Hz, 2H), 6.84 (d, *J* = 8.7 Hz, 2H), 4.94 (s, 1H). ^{13}C NMR (100 MHz. $CDCl_3$): δ 129.3, 129.2, 128.5, 122.4, 47.2.

1-Bromo-4-(diazomethyl)benzene (4)

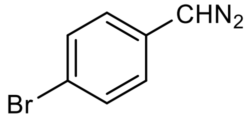

The resulting (*E*)-*N'*-(4-bromobenzylidene)-4-methylbenzenesulfonohydrazide was obtained as a white solid in 85% yield (R_f = 0.14 in $CHCl_3$). ^{1}H NMR (400 MHz, DMSO-d_6): 11.57 (s, 1H), 7.89 (s, 1H), 7.77 (d, *J* = 8.1 Hz, 2H), 7.59 (d, *J* = 8.5 Hz, 2H), 7.51 (d, *J* = 8.5 Hz, 2H), 7.41 (d, *J* = 8.1 Hz, 2H), 2.36 (s, 3H). ^{13}C NMR (100 MHz, DMSO-d_6): δ 146.2, 144.0, 136.6, 133.4, 132.3, 130.2, 129.1, 127.7, 123.8, 21.5. 1-Bromo-4-(diazomethyl)benzene was obtained as a dark red solid by sublimation. ^{1}H NMR (400 MHz, $CDCl_3$): δ 7.40 (d, *J* = 8.7 Hz, 2H), 6.78 (d, *J* = 8.7 Hz, 2H), 4.93 (s, 1H). ^{13}C NMR (100 MHz. $CDCl_3$): δ 132.1, 129.1, 122.8, 116.8, 47.3.

1-(Diazomethyl)-4-(trifluoromethyl)benzene (5)

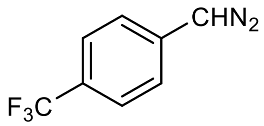

The resulting (*E*)-4-methyl-*N'*-(4-(trifluoromethyl)benzylidene)benzenesulfonohydrazide was obtained as a white solid in 26% yield (R_f = 0.14 in $CHCl_3$). ^{1}H NMR (400 MHz, $CDCl_3$): δ 8.22 (s, 1H), 7.90 (d, *J* = 8.2 Hz, 2H), 7.80 (s, 1H), 7.71 (d, *J* = 8.4 Hz, 2H), 7.63 (d, *J* = 8.2 Hz, 2H), 7.35 (d, *J* = 8.2 Hz, 2H), 2.44 (s, 3H). ^{13}C NMR (100 MHz, $CDCl_3$): δ 145.4, 144.6, 136.5, 135.1, 129.8, 128.0, 127.5, 125.7, 21.6. ^{19}F NMR (377 MHz. $CDCl_3$): δ -62.9. 1-(Diazomethyl)-4-(trifluoromethyl)benzene was obtained as a dark red liquid by vacuum distillation. ^{1}H NMR (400 MHz, $CDCl_3$): 7.54 (d, *J* = 8.4 Hz, 2H), 7.00 (d, *J* = 8.4 Hz, 2H), 5.04 (s, 1H). ^{13}C NMR (100 MHz, $CDCl_3$): δ 134.7, 126.0, 121.0, 47.8. ^{19}F NMR (377 MHz. $CDCl_3$): δ -62.1.

1-(Diazomethyl)-4-methylbenzene (6)

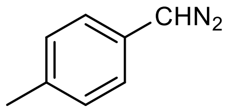

The resulting (*E*)-4-methyl-*N'*-(4-methylbenzylidene)benzenesulfonohydrazide was obtained as a white solid in 64% yield (R_f = 0.17 in $CHCl_3$). ^{1}H NMR (400 MHz, $CDCl_3$): δ 8.28 (s, 1H), 7.91 (d, 2H), 7.77 (s, 1H), 7.49 (d, 2H), 7.33 (d, 2H), 7.17 (d, 2H), 2.41 (s, 3H), 2.36 (s, 3H). ^{13}C NMR (100 MHz, $CDCl_3$): δ 148.3, 144.2, 140.8, 135.3, 130.5, 129.7, 129.4, 128.0, 127.4, 21.6, 21.5. 1-(Diazomethyl)-4-methylbenzene was obtained as a blood-red liquid by vacuum distillation. ^{1}H NMR (400 MHz, $CDCl_3$): δ 7.17 (d, 2H), 6.88 (d, 2H), 4.97 (s, 1H), 2.37 (s, 3H). ^{13}C NMR (100 MHz, $CDCl_3$): δ 133.6, 129.9, 126.3, 121.4, 47.0, 21.0.

1-(Diazomethyl)-4-isopropylbenzene (7)

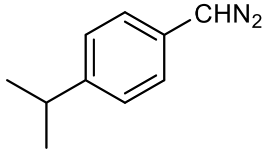

The resulting *(E)*-*N'*-(4-isopropylbenzylidene)-4-methylbenzenesulfonohydrazide was obtained as a white solid in 52% yield (R_f = 0.13 in $CHCl_3$). ^{1}H NMR (400 MHz, $CDCl_3$): δ 8.15 (s, 1H), 7.88 (d, 2H), 7.75 (s, 1H), 7.51 (d, 2H), 7.30 (d, 2H), 7.21 (d, 2H), 2.97-2.80 (m, 1H), 2.39 (s, 3H), 1.24 (d, 6H). ^{13}C NMR (100 MHz, $CDCl_3$): δ 151.9, 148.4, 144.3, 135.4, 130.9, 129.8, 128.1, 127.6, 126.9, 34.2, 23.9, 21.7. 1-(Diazomethyl)-4-isopropylbenzene was obtained as a blood-red liquid by vacuum distillation. ^{1}H NMR (400 MHz,

$CDCl_3$): δ 7.24 (d, 2H), 6.93 (d, 2H), 4.96 (s, 1H), 2.94 (m, 1H), 1.32 (d, 6H). ^{13}C NMR (100 MHz, $CDCl_3$): δ144.8, 127.3, 126.7, 121.5, 46.9, 33.7, 24.1.

Methyl 4-(diazomethyl)benzoate (8)

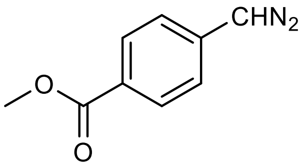

The resulting methyl (*E*)-4-((2-tosylhydrazineylidene)methyl)benzoate was obtained as a white crystal in 89% yield (R_f = 0.07 in $CHCl_3$). ^1H NMR (400 MHz, DMSO-d_6): 11.75 (s, 1H), 8.00 – 7.91 (m, 3H), 7.79 (d, *J* = 8.2 Hz, 2H), 7.70 (d, *J* = 8.4 Hz, 2H), 7.40 (d, *J* = 8.2 Hz, 2H), 3.84 (s, 3H), 2.34 (s, 3H). ^{13}C NMR (100 MHz, DMSO-d_6): δ 166.2, 146.0, 144.1, 138.5, 136.6, 130.9, 130.2, 130.1, 127.7, 127.4, 52.7, 21.4. Methyl 4-(diazomethyl)benzoate was obtained as a dark red solid by sublimation. ^1H NMR (400 MHz, $CDCl_3$): δ 7.95 (d, *J* = 8.7 Hz, 2H), 6.95 (d, *J* = 8.5 Hz, 2H), 5.05 (s, 1H), 3.90 (s, 3H). ^{13}C NMR (100 MHz. $CDCl_3$): δ 166.8, 136.0, 130.4, 125.4, 120.7, 51.9, 48.5.

(4-(Diazomethyl)phenyl)(methyl)sulfane (9)

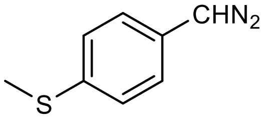

The resulting (*E*)-4-methyl-*N*'-(4-(methylthio)benzylidene)benzenesulfonohydrazide was obtained as a white solid in 70% yield (R_f = 0.22 in $CHCl_3$). ^1H NMR (400 MHz, $CDCl_3$): δ 8.28 (s, 1H), 7.88 (d, 2H), 7.72 (s, 1H), 7.47 (d, 2H), 7.30 (d, 2H), 7.17 (d, 2H), 2.47 (s, 3H), 2.39 (s, 3H). ^{13}C NMR (100 MHz, $CDCl_3$): δ 147.7, 144.3, 142.0, 135.2, 129.8, 129.7, 127.9, 127.7, 125.7, 21.6, 15.2. (4-(Diazomethyl)phenyl)(methyl)sulfane was obtained as a dark red solid by sublimation. ^1H NMR (400 MHz, $CDCl_3$): δ 7.24 (d, 2H), 6.85 (d, 2H), 4.92 (s, 1H), 2.47 (s, 3H). ^{13}C NMR (100 MHz, $CDCl_3$): δ 133.2, 128.6, 126.9, 121.9, 47.3, 16.9.

4-(Diazomethyl)benzonitrile (10)

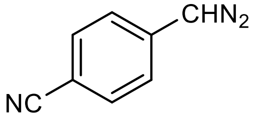

The resulting (*E*)-*N*'-(4-cyanobenzylidene)-4-methylbenzenesulfonohydrazide was obtained as a white crystal in 52% yield (R_f = 0.10 in $CHCl_3$). ^1H NMR (400 MHz, $CDCl_3$): 8.83 – 8.72 (m, 1H), 7.90 (d, *J* = 8.2 Hz, 2H), 7.81 (s, 1H), 7.66 (q, *J* = 8.3 Hz, 4H), 7.35 (d, *J* = 8.2 Hz, 2H), 2.44 (s, 3H). ^{13}C NMR (100 MHz, $CDCl_3$): δ 144.9, 144.8, 137.4, 135.0, 132.4, 129.9, 127.9, 127.6, 118.4, 113.4, 21.7. 4-(Diazomethyl)benzonitrile was obtained as a red solid by sublimation. ^1H NMR (400 MHz, $CDCl_3$): δ 7.54 (d, *J* = 8.5 Hz, 2H), 6.97 (d, *J* = 8.5 Hz, 2H), 5.08 (s, 1H). ^{13}C NMR (100 MHz. $CDCl_3$): δ 136.5, 132.8, 121.2, 119.3, 106.4, 48.9.

1-(Diazomethyl)-2-fluorobenzene (11)

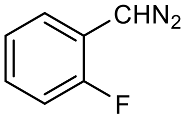

The resulting (*E*)-*N*'-(2-fluorobenzylidene)-4-methylbenzenesulfonohydrazide was obtained as a white solid in 26% yield (R_f = 0.21 in $CHCl_3$). ^1H NMR (400 MHz, $CDCl_3$): δ 8.27 (br, 1H), 8.01 (s, 1H), 7.92-7.79 (m, 3H), 7.36-7.29 (m, 3H), 7.12 (m, 1H), 7.01 (m, 1H), 2.40 (s, 3H). ^{13}C NMR (100 MHz, $CDCl_3$): δ 162.5,

160.0, 144.4, 140.7, 135.2, 132.0(2), 129.8, 128.0, 126.9, 124.4, 121.0(2), 115.8(2), 21.6. ^{19}F NMR (377 MHz. $CDCl_3$): δ -120.8. 1-(Diazomethyl)-2-fluorobenzene was obtained as a blood-red liquid by vacuum distillation. ^{1}H NMR (400 MHz, $CDCl_3$): δ 7.15-7.11 (m, 1H), 7.06-7.00 (m, 2H), 6.96-6.92 (m, 1H), 5.10 (s, 1H). ^{13}C NMR (100 MHz. $CDCl_3$): δ 157.2, 156.2, 124.6(2), 124.5(2), 122.4(2), 118.1(2), 115.5(2), 42.0. ^{19}F NMR (377 MHz. $CDCl_3$): δ -120.3.

1-Chloro-2-(diazomethyl)benzene (12)

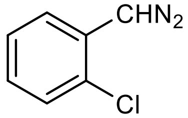

The resulting (*E*)-*N*'-(2-chlorobenzylidene)-4-methylbenzenesulfonohydrazide was obtained as a white solid in 56% yield (R_f = 0.23 in $CHCl_3$). ^{1}H NMR (400 MHz, $CDCl_3$): δ 8.40 (br, 1H), 8.21 (s, 1H), 7.92 (d, *J* = 7.0 Hz, 3H), 7.36-7.22 (m, 5H), 2.43 (s, 3H). ^{13}C NMR (100 MHz, $CDCl_3$): δ 144.4, 143.8, 135.2, 134.1, 131.3, 130.7, 129.8, 129.7, 128.0, 127.4, 127.0, 21.6. 1-Chloro-2-(diazomethyl)benzene was obtained as a dark red liquid by vacuum distillation. ^{1}H NMR (400 MHz, $CDCl_3$): δ 7.35 (d, 1H), 7.26 (t, *J* = 7.6 Hz, 3H), 7.00-6.93 (m, 2H), 5.42 (s, 1H). ^{13}C NMR (100 MHz, $CDCl_3$): δ 129.9, 128.5, 127.2, 126.7, 124.3, 122.0, 45.4.

1-Bromo-2-(diazomethyl)benzene (13)

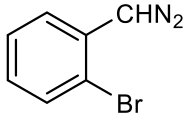

The resulting *(E)-N'*-(2-bromobenzylidene)-4-methylbenzenesulfonohydrazide was obtained as a white solid in 73% yield (R_f = 0.24 in $CHCl_3$). ^{1}H NMR (400 MHz, $CDCl_3$): δ 8.28 (br, 1H), 8.14 (s, 1H), 7.91-7.84 (m, 3H), 7.50 (d, *J* = 7.9 Hz, 1H), 7.35-7.15 (m, 4H), 2.43 (s, 3H). ^{13}C NMR (100 MHz, $CDCl_3$): δ 146.1, 144.4, 135.2, 133.0, 132.1, 131.5, 129.8, 128.0, 127.8, 127.6, 124.1, 21.6. 1-Bromo-2-(diazomethyl)benzene was obtained as a dark red liquid by vacuum distillation. ^{1}H NMR (400 MHz, $CDCl_3$): 7.51 (d, *J* = 8.0 Hz, 1H), 7.29 (t, *J* = 7.6 Hz, 1H), 6.93 (d, *J* = 8.0 Hz, 1H), 6.90 (t, *J* = 7.6 Hz, 1H), 5.47 (s, 1H). ^{13}C NMR (100 MHz, $CDCl_3$): δ 133.2, 130.2, 127.8, 124.7, 122.1, 116.4, 48.0.

1-(Diazomethyl)-2-methylbenzene (14)

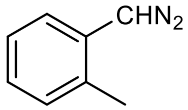

The resulting (*E*)-4-methyl-*N*'-(2-methylbenzylidene)benzenesulfonohydrazide was obtained as a white solid in 62% yield (R_f = 0.25 in $CHCl_3$). ^{1}H NMR (400 MHz, $CDCl_3$): δ 8.39 (br, 1H), 8.08 (s, 1H), 7.91 (d, *J* = 8.2 Hz, 2H), 7.68 (d, *J* = 7.6 Hz, 1H), 7.33 (d, *J* = 8.1 Hz, 2H), 7.30-7.10 (m, 3H), 2.42 (s, 3H), 2.39 (s, 3H). ^{13}C NMR (100 MHz, $CDCl_3$): δ 147.3, 144.3, 137.2, 135.3, 131.2, 130.9, 130.2, 129.7, 128.0, 127.4, 126.1, 21.6, 19.9. 1-(Diazomethyl)-2-methylbenzene was obtained as a dark red liquid by vacuum distillation. ^{1}H NMR (400 MHz, $CDCl_3$): 7.24 (t, *J* = 7.7 Hz, 1H), 7.17 (d, *J* = 7.6 Hz, 1H), 7.01 (t, *J* = 7.5 Hz, 1H), 6.96 (d, *J* = 7.6 Hz, 1H), 4.98 (s, 1H), 2.22 (s, 3H). ^{13}C NMR (100 MHz, $CDCl_3$): δ 130.6, 130.5, 128.0, 126.6, 123.5, 121.0, 44.4, 19.3.

1-(Diazomethyl)-3-(trifluoromethyl)benzene (15)

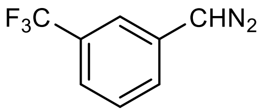

The resulting (*E*)-4-methyl-*N*'-(3-(trifluoromethyl)benzylidene)benzenesulfonohydrazide was obtained as a white solid in 35% yield (R_f = 0.16 in $CHCl_3$). ^{1}H NMR (400 MHz, $CDCl_3$): δ 8.31 (br, 1H), 7.91 (d, *J* = 8.1 Hz, 2H), 7.84-7.75 (m, 3H), 7.63 (d, *J* = 7.9 Hz, 1H), 7.50 (t, *J* = 7.8 Hz, 1H), 7.36 (*d*, J = 8.1 Hz, 2H), 2.44 (s, 3H). ^{13}C NMR (100 MHz, $CDCl_3$): δ 145.7, 144.6, 135.1, 134.0, 131.4, 131.1, 130.3, 129.8, 129.2, 128.0, 126.9, 125.1, 124.1, 21.6. ^{19}F NMR (377 MHz. $CDCl_3$): δ -62.9. 1-(Diazomethyl)-3-(trifluoromethyl)benzene was obtained as a dark red liquid by vacuum distillation. ^{1}H NMR (400 MHz, $CDCl_3$): 7.40 (t, *J* = 7.9 Hz, 1H), 7.29 (d, *J* = 7.7 Hz, 1H), 7.17-7.13 (m, 1H), 7.07 (d, *J* = 7.9 Hz, 1H), 5.02 (s, 1H). ^{13}C NMR (100 MHz, $CDCl_3$): δ 131.5, 129.5, 124.1, 120.4, 117.7, 47.6. ^{19}F NMR (377 MHz. $CDCl_3$): δ -63.1.

1-(Diazomethyl)-3,5-bis(trifluoromethyl)benzene (16)

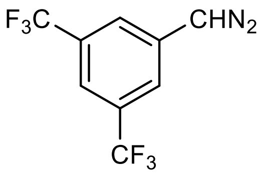

The resulting (*E*)-*N*'-(3,5-bis(trifluoromethyl)benzylidene)-4-methylbenzenesulfonohydrazide was obtained as a white crystal in 76% yield (R_f = 0.14 in $CHCl_3$). ^{1}H NMR (400 MHz, $CDCl_3$): δ 8.64 (s, 1H), 8.00 (s, 2H), 7.92 (d, *J* = 6.9 Hz, 2H), 7.88 – 7.84 (m, 2H), 7.37 (d, *J* = 8.1 Hz, 2H), 2.45 (s, 3H). ^{13}C NMR (100 MHz, $CDCl_3$): δ 144.9, 143.5, 135.4, 134.8, 132.4, 132.1, 129.9, 128.0, 126.9, 124.3, 123.5, 121.6, 21.6. ^{19}F NMR (377 MHz. $CDCl_3$): δ -63.1. 1-(Diazomethyl)-3,5-bis(trifluoromethyl)benzene was obtained as a dark red liquid by vacuum distillation. ^{1}H NMR (400 MHz, $CDCl_3$): 7.51 (s, 1H), 7.30 (s, 2H), 5.12 (s, 1H). ^{13}C NMR (100 MHz, $CDCl_3$): δ 133.6, 132.6 (4), 126.5, 124.3, 122.2, 120.4(2), 120.0, 117.0(m), 48.1. ^{19}F NMR (377 MHz. $CDCl_3$): δ -63.6.

4-Bromo-2-(diazomethyl)-1-fluorobenzene (17)

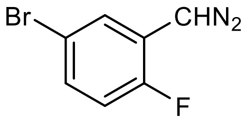

The resulting (*E*)-*N*'-(5-bromo-2-fluorobenzylidene)-4-methylbenzenesulfonohydrazide was obtained as a white solid in 42% yield (R_f = 0.15 in $CHCl_3$). ^{1}H NMR (400 MHz, $CDCl_3$): 8.55 (s, 1H), 7.97 – 7.87 (m, 4H), 7.47 – 7.40 (m, 1H), 7.36 (d, *J* = 8.1 Hz, 2H), 6.93 (t, *J* = 9.8 Hz, 1H), 2.44 (s, 3H). ^{13}C NMR (100 MHz, $CDCl_3$): δ 161.3, 158.8, 144.6, 139.0, 135.0, 134.6(2), 129.9, 129.3(2), 127.9, 123.0(2), 117.7(2), 117.3(2), 21.6. ^{19}F NMR (377 MHz. $CDCl_3$): δ -122.7. 4-Bromo-2-(diazomethyl)-1-fluorobenzene was obtained as a red solid by sublimation. ^{1}H NMR (400 MHz, $CDCl_3$): δ 7.10 – 7.05 (m, 1H), 7.02 (d, *J* = 7.0 Hz, 1H), 6.88 (t, *J* = 9.5 Hz, 1H), 5.04 (s, 1H). ^{13}C NMR (100 MHz. $CDCl_3$): δ 156.5, 154.5, 127.1(2), 124.8(2), 121.0(2), 117.3(2), 117.0(2), 42.2. ^{19}F NMR (377 MHz. $CDCl_3$): δ -122.6.

1-(Diazomethyl)-2,3,4,5,6-pentafluorobenzene (18)

The resulting *(E)*-4-methyl-*N*'-((perfluorophenyl)methylene)benzenesulfonohydrazide was obtained as a white solid in 75% yield (R_f = 0.28 in $CHCl_3$). ^{1}H NMR (400 MHz, $CDCl_3$): δ 8.72 (s, 1H), 7.91 (d, 2H), 7.83 (s, 1H), 7.38 (d, 2H), 2.46 (s, 3H). ^{13}C NMR (100 MHz, $CDCl_3$): δ

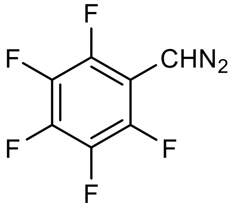

146.4, 144.8, 143.8, 140.5, 138.9, 136.5, 134.6, 134.5, 129.8, 128.1, 108.8, 21.7. ^{19}F NMR (377 MHz, $CDCl_3$): δ -140.9, -151.1, -161.5. 1-(Diazomethyl)-2,3,4,5,6-pentafluorobenzene was obtained as a blood-red liquid by vacuum distillation. ^{1}H NMR (400 MHz, $CDCl_3$): δ 4.91 (s, 1H). ^{13}C NMR (100 MHz. $CDCl_3$): δ 141.9, 139.3, 138.4, 136.7, 135.9, 106.5, 38.3. ^{19}F NMR (377 MHz. $CDCl_3$): δ -146.7, -162.3, -163.0.

2,4-Dichloro-1-(diazomethyl)benzene (19)

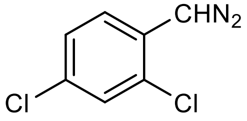

The resulting (*E*)-*N'*-(2,4-dichlorobenzylidene)-4-methylbenzenesulfonohydrazide was obtained as a white crystal in 85% yield (R_f = 0.20 in $CHCl_3$). ^{1}H NMR (400 MHz, DMSO-d_6): 11.85 (s, 1H), 8.20 (s, 1H), 7.80 – 7.72 (m, 3H), 7.66 – 7.60 (m, 1H), 7.47 – 7.37 (m, 3H), 2.35 (s, 3H). ^{13}C NMR (100 MHz, DMSO-d_6): δ 144.1, 142.0, 136.5, 135.6, 134.0, 130.4, 130.3, 129.8, 128.5, 128.2, 127.6, 21.5. 2,4-Dichloro-1-(diazomethyl)benzene was obtained as a dark red solid by sublimation. ^{1}H NMR (400 MHz, $CDCl_3$): δ 7.33 (s, 1H), 7.21 (d, J = 8.5 Hz, 1H), 6.83 (d, J = 8.5 Hz, 1H), 5.36 (s, 1H). ^{13}C NMR (100 MHz. $CDCl_3$): δ 129.7, 128.9, 127.5, 127.2, 126.9, 122.5, 45.4.

2-(Diazomethyl)naphthalene (20)

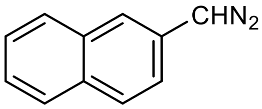

The resulting (*E*)-4-methyl-*N'*-(naphthalen-2-ylmethylene)benzenesulfonohydrazide was obtained as a white solid in 80% yield (R_f = 0.17 in $CHCl_3$). ^{1}H NMR (400 MHz, $CHCl_3$): 8.16 (s, 1H), 7.97 – 7.91 (m, 3H), 7.97 – 7.78 (m, 5H), 7.58 – 7.46 (m, 2H), 7.34 (d, J = 8.2 Hz, 2H), 2.42 (s, 3H). ^{13}C NMR (100 MHz, $CHCl_3$): δ 148.0, 144.4, 135.3, 134.4, 133.0, 130.9, 129.8, 129.2, 128.6, 128.4, 128.0, 127.9, 127.3, 126.7, 122.9, 21.6. 2-(Diazomethyl)naphthalene was obtained as a dark red solid by sublimation. ^{1}H NMR (400 MHz, $CDCl_3$): δ 8.04 – 7.22 (m,6H), 7.08 (s, 1H), 5.10 (s, 1H). ^{13}C NMR (100 MHz. $CDCl_3$): δ 134.2, 131.0, 129.0, 127.9, 127.2, 126.7 (2), 124.6, 121.0, 118.2, 48.0.

Supplementary Tables

Table S1. C1 polymerization of (diazomethyl)benzene (1) initiated by carbeniums.[a]

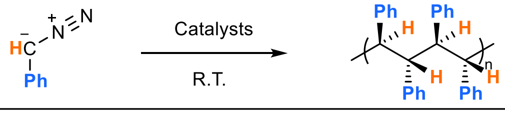

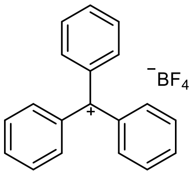

$C(Ph)_3^+BF_4^-$

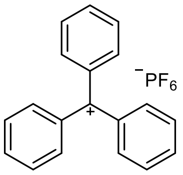

$C(Ph)_3^+PF_6^-$

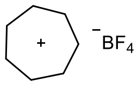

$Trop^+BF_4^-$

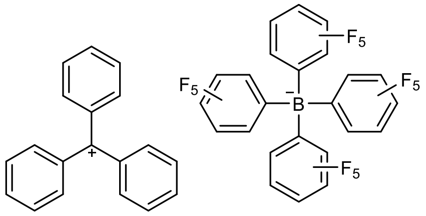

$C(Ph)_3^+B(C_6F_5)_4^-$

Entry	Cat.	$[1]_0/[Cat.]_0$	Time[b]	Conv. (%)[b]	$M_{n,SEC}$ (kg mol^{-1})[c]	$Đ$[c]	Yield (%)[d]
1	$C(Ph)_3^+BF_4^-$	30/1	< 5 s	100	12.8	1.52	88
Experiment phenomenon: Upon addition of the red monomer to the yellow catalyst solution in toluene, an immediate exothermic reaction occurred, accompanied by vigorous gas evolution. The reaction mixture instantly turned yellow.							
2	$C(Ph)_3^+PF_6^-$	30/1	2 min	100	37.8[e]	1.56	71
Experiment phenomenon: Upon addition of the red monomer to the orange catalyst solution in toluene, an immediate exothermic reaction occurred, accompanied by continuous gas evolution lasting approximately 2 minutes. During this period, the color of the reaction mixture gradually changed from red to yellow.							
3	$Trop^+BF_4^-$	30/1	< 5 s	100	12.3	1.44	79
Experiment phenomenon: Upon addition of the red monomer to the pale white catalyst solution in toluene, an immediate exothermic reaction occurred, accompanied by vigorous gas evolution. The reaction mixture instantly turned yellow.							
4	$C(Ph)_3^+B(C_6F_5)_4^-$	30/1	2 h	N.A.[f]	N.D.[f]	N.D.[f]	< 1
Experiment phenomenon: Upon addition of the red monomer to the red catalyst solution in toluene, a small amount of gas was released, after which bubbling ceased. After 2 hours, the solution gradually turned orange-red.							
5	$C(Ph)_3^+BF_4^-$	50/1	< 5 s	100	15.9	1.49	90
6	$C(Ph)_3^+BF_4^-$	100/1	1 min	100	29.0	1.47	93
7	$C(Ph)_3^+BF_4^-$	150/1	3 min	100	51.1	1.53	94
8	$C(Ph)_3^+BF_4^-$	200/1	5 min	100	67.8	1.55	93

[a]C1 polymerizations were performed in toluene at room temperature with $[1]_0$ = 0.2 M. [b]Reaction progress was monitored by color change from red to yellow. [c]Molecular weights were determined by SEC in THF, calibrated with polystyrene standards. [d]Polymer yields were calculated based on the weight of the dried product after precipitation in methanol. [e]The unusually high molecular weight could be attributed to limited solubility of $C(Ph)_3^+PF_6^-$ in toluene. [f]Throughout the experiment, the reaction solution remained red; however, since the catalyst $C(Ph)_3^+B(C_6F_5)_4^-$ itself is also red, it is difficult to determine whether the observed color originates from the unreacted monomer or the catalyst. SEC analysis did not detect any polymer formation.

Table S2. C1 polymerization of (diazomethyl)benzene (1) initiated by Ni complexes.[a]

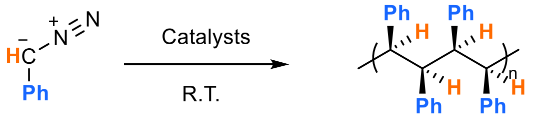

$NiCl_2$

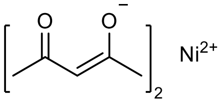

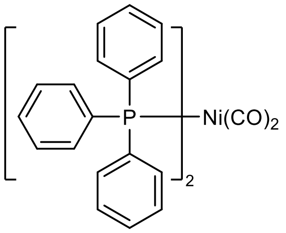

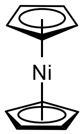

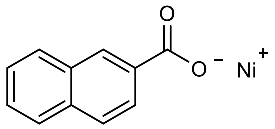

$Ni(acac)_2$ $Ni(CO)_2(PPh_3)_2$ $Ni(Cp)_2$ $Ni(2\text{-}NaphCOO)_2$

**Entry**	**Cat.**	**$[1]_0/[Cat.]_0$**	**Time[b]**	**Conv. (%)[b]**	**$M_{n,SEC}$ (kg mol^{-1})[c]**	**Đ[c]**	**Yield (%)[d]**
1	$NiCl_2$	30/1	24 h[e]	N.A.[e]	N.D.[e]	N.D.[e]	< 1
Experiment phenomenon: Upon addition of the red monomer to the yellow catalyst solution in toluene (with yellow precipitate at the bottom), the mixture turned red. No obvious reaction phenomena were observed.							
2	$Ni(acac)_2$	30/1	24 h	100	9.8[f]	1.37[f]	95
Experiment phenomenon: Upon addition of the red monomer to the cyan catalyst solution in toluene, the mixture immediately turned red. Over time, the solution gradually darkened to a deep red, accompanied by the gradual formation of a white precipitate. After completion of the reaction, the deep red solution rapidly turned yellow within a few seconds upon exposure to air.							
3	$Ni(CO)_2(PPh_3)_2$	30/1	24 h[e]	N.A.[e]	N.D.[e]	N.D. [e]	< 1
Experiment phenomenon: Upon addition of the red monomer to the white catalyst solution in toluene (with white precipitate at the bottom), the mixture turned red. No obvious reaction phenomena were observed. After standing in the glovebox for several days, the solution gradually turned into an opaque black suspension.							
4	$Ni(Cp)_2$	30/1	24 h[e]	N.A.[e]	N.D.[e]	N.D. [e]	< 1
Experiment phenomenon: Upon addition of the red monomer to the cyan catalyst solution in toluene (with cyan precipitate at the bottom), the mixture turned red. No obvious reaction phenomena were observed. After standing in the glovebox for several days, the solution gradually turned into an opaque black suspension.							
5	$Ni(2\text{-}NaphCOO)_2$	30/1	6 days[e]	N.A.[e]	2.6	1.3	< 1
Experiment phenomenon: Upon addition of the red monomer to the dark yellow catalyst solution in toluene, the mixture turned red. No obvious reaction phenomena were observed. After standing in the glovebox for several days, the solution gradually turned into an opaque dark red suspension.							

[a]C1 polymerizations were performed in toluene at room temperature with $[1]_0$ = 0.2 M. [b]Reaction progress was monitored by color change from red to yellow. [c]Molecular weights were determined by SEC in THF, calibrated with polystyrene standards. [d]Polymer yields were calculated based on the weight of the dried product after precipitation in methanol. [e]Throughout the experiment, the reaction solution remained red. SEC analysis did not detect any polymer formation. [f]The obtained polymer is difficult to dissolve in THF.

Table S3. C1 polymerization of (diazomethyl)benzene (1) initiated by other catalysts.[a]

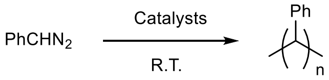

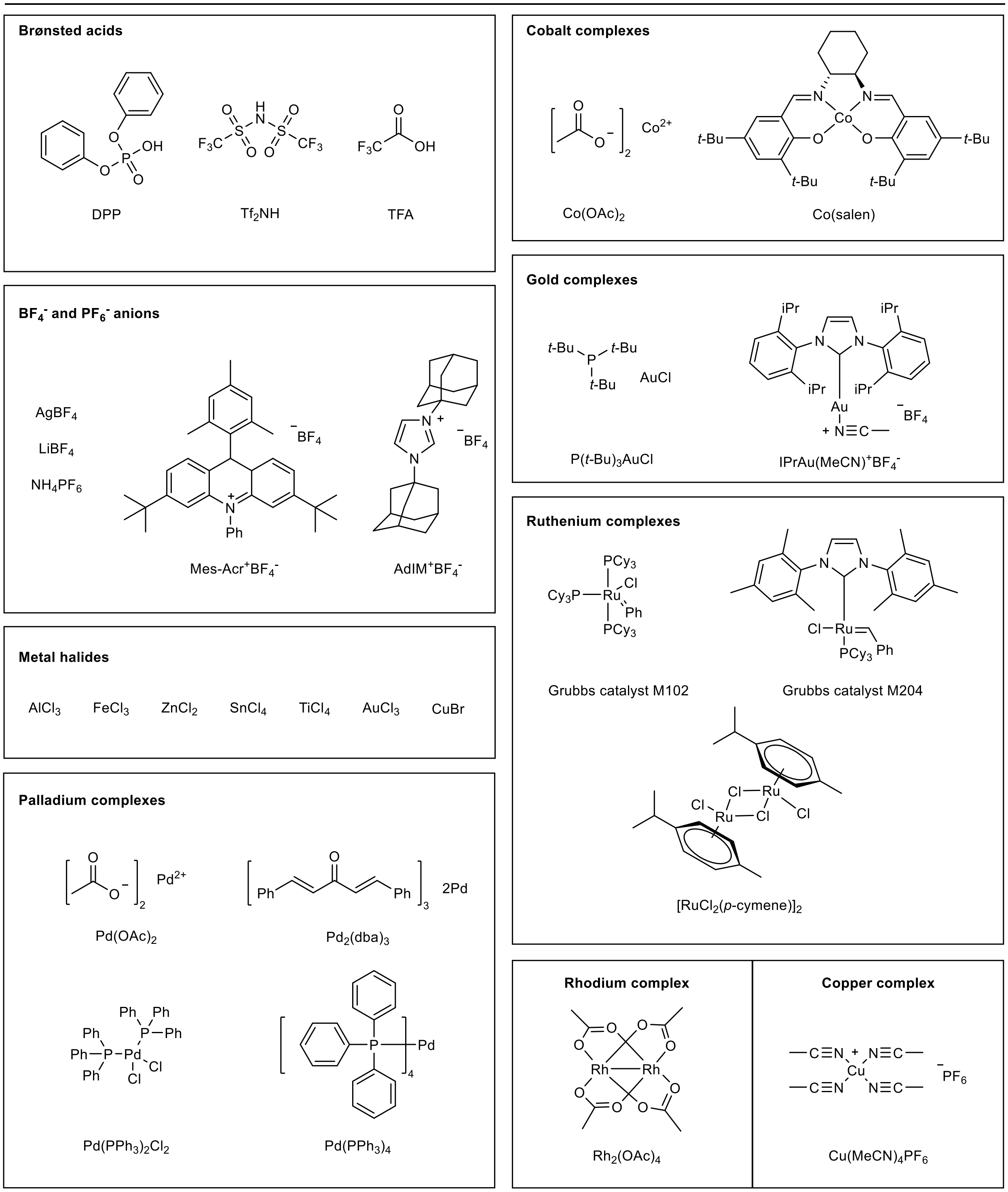

Entry	Cat.	Time[b]	Conv. (%)[b]	$M_{n,SEC}$[c]	$Đ$[c]	Yield[d]
**Brøsted acids**						
1	DPP	6 days	N.A.	N.D.	N.D.	< 1
Experiment phenomenon: Upon addition of the red monomer to the colorless catalyst solution in toluene, the mixture turned red. No obvious reaction phenomena were observed, and the red color of the monomer remained unchanged.						
2	Tf_2NH	6 days	N.A.	N.D.	N.D.	< 1
Experiment phenomenon: Upon addition of the red monomer to the colorless catalyst solution in toluene, the mixture turned red. No obvious reaction phenomena were observed, and the red color of the monomer remained unchanged.						
3	TFA	6 days	N.A.	N.D.	N.D.	< 1
Experiment phenomenon: Upon addition of the red monomer to the colorless catalyst solution in toluene, the mixture turned red. No obvious reaction phenomena were observed, and the red color of the monomer remained unchanged.						
**BF_4^- and PF_6^- anions**						
4	$AgBF_4$	6 days	N.A.	N.D.	N.D.	< 1
Experiment phenomenon: Upon addition of the red monomer to the colorless catalyst solution in toluene, a small amount of gas and heat were released, turning the solution into an opaque dark brown mixture with the formation of a black precipitate. Bubbling ceased shortly thereafter.						
5	$LiBF_4$	6 days	N.A.	N.D.	N.D.	< 1
Experiment phenomenon: Upon addition of the red monomer to the colorless catalyst solution in toluene (with white precipitate at the bottom), the mixture turned red. No obvious reaction phenomena were observed, and the red color of the monomer remained unchanged.						
6	NH_4PF_6	6 days	N.A.	N.D.	N.D.	< 1
Experiment phenomenon: Upon addition of the red monomer to the colorless catalyst solution in toluene (with white precipitate at the bottom), the mixture turned red. No obvious reaction phenomena were observed, and the red color of the monomer remained unchanged.						
7	Mes-$Acr^+BF_4^-$	6 days	N.A.	N.D.	N.D.	< 1
Experiment phenomenon: Upon addition of the red monomer to the yellow catalyst solution in toluene (with yellow precipitate at the bottom), the mixture turned red. No obvious reaction phenomena were observed, and the red color of the monomer remained unchanged.						
8	$AdIM^+BF_4^-$	6 days	N.A.	N.D.	N.D.	< 1
Experiment phenomenon: Upon addition of the red monomer to the colorless catalyst solution in toluene (with white precipitate at the bottom), the mixture turned red. No obvious reaction phenomena were observed, and the red color of the monomer remained unchanged.						
**Metal halides**						
9	$AlCl_3$	6 days	N.A.	N.D.	N.D.	< 1
Experiment phenomenon: Upon addition of the red monomer to the colorless catalyst solution in toluene (with white precipitate at the bottom), the mixture turned red. No obvious reaction phenomena were observed, and the red color of the monomer remained unchanged.						
10	$FeCl_3$	< 5 s	100%	N.D.	N.D.	< 1
Experiment phenomenon: Upon addition of the red monomer to the brown catalyst solution in toluene, an immediate exothermic reaction occurred, accompanied by vigorous gas evolution. The reaction mixture instantly turned brown.						
11	$ZnCl_2$	1 h	100%	N.D.	N.D.	< 1
Experiment phenomenon: Upon addition of the red monomer to the colorless catalyst solution in toluene, continuous gas evolution was observed for approximately 1 h. During this period, the color of the reaction mixture gradually changed from red to yellow.						
12	$SnCl_4$	10 h	100%	N.D.	N.D.	< 1
Experiment phenomenon: Upon addition of the red monomer to the colorless catalyst solution in toluene, continuous gas evolution was observed for approximately 10 h. During this period, the color of the reaction mixture gradually changed from red to yellow.						
13	$TiCl_4$	6 days	N.A.	N.D.	N.D.	< 1
Experiment phenomenon: Upon addition of the red monomer to the yellow catalyst solution in toluene, bubbles were observed at the beginning, and the red color of the monomer gradually changed to black, making it difficult to observe further changes						
14	$AuCl_3$	6 days	N.A.	N.D.	N.D.	< 1

Experiment phenomenon: Upon addition of the red monomer to the colorless catalyst solution in toluene (with black precipitate at the bottom), the red color of the monomer gradually darkened to black, accompanied by the formation of black precipitate, making it difficult to observe further changes.						
15	CuBr	20 min	100%	N.D.	N.D.	< 1
Experiment phenomenon: Upon addition of the red monomer to the colorless catalyst solution in toluene, continuous gas evolution was observed for approximately 20 min. During this period, the color of the reaction mixture gradually changed from red to yellow.						
**Palladium complexes**						
16	$Pd(OAc)_2$	10 min	100%	N.D.	N.D.	< 1
Experiment phenomenon: Upon addition of the red monomer to the yellow catalyst solution in toluene, an immediate exothermic reaction occurred, accompanied by vigorous gas evolution. During this period, the color of the reaction mixture gradually changed from red to yellow-brown. Upon continued standing, the mixture turned into an opaque black solution.						
17	$Pd_2(dba)_3$	6 days	N.A.	N.D.	N.D.	< 1
Experiment phenomenon: Upon addition of the red monomer to the black catalyst suspension in toluene, a small amount of gas and heat were released, turning the solution into an opaque black mixture, making it difficult to observe further changes						
18	$Pd(PPh_3)_2Cl_2$	10 h	100%	N.D.	N.D.	< 1
Experiment phenomenon: Upon addition of the red monomer to the yellow catalyst suspension in toluene, the mixture turned red. No obvious reaction phenomena were observed, and the red color of the monomer remained unchanged. After standing for 10 h, the mixture turned yellow.						
19	$Pd(PPh_3)_4$	6 days	N.A.	N.D.	N.D.	< 1
Experiment phenomenon: Upon addition of the red monomer to the brown catalyst suspension in toluene, the mixture turned red. No obvious reaction phenomena were observed, and the red color of the monomer remained unchanged. After standing for 1 h, the mixture turned into an opaque black mixture, making it difficult to observe further changes.						
**Cobalt complexes**						
20	$Co(OAc)_2$	6 days	N.A.	N.D.	N.D.	< 1
Experiment phenomenon: Upon addition of the red monomer to the colorless catalyst solution in toluene (with pink precipitate at the bottom), the mixture turned red. No obvious reaction phenomena were observed, and the red color of the monomer remained unchanged.						
21	Co(salen)	6 days	N.A.	N.D.	N.D.	< 1
Experiment phenomenon: Upon addition of the red monomer to the red catalyst solution in toluene, the mixture turned red. No obvious reaction phenomena were observed, and the red color of the monomer or catalyst remained unchanged. After standing for 1 h, the mixture turned into an opaque black mixture, making it difficult to observe further changes.						
**Gold complexes**						
22	$P(t\text{-}Bu)_3AuCl$	6 days	N.A.	N.D.	N.D.	< 1
Experiment phenomenon: Upon addition of the red monomer to the colorless catalyst solution in toluene, the mixture turned red. No obvious reaction phenomena were observed, and the red color of the monomer remained unchanged.						
23	$IPrAu(MeCN)^+BF_4^-$	4 h	100%	N.D.	N.D.	< 1
Experiment phenomenon: Upon addition of the red monomer to the colorless catalyst solution in toluene (with pink precipitate at the bottom), the mixture turned red, and a small amount of gas was observed. After 4 h, the solution turned yellow with black precipitate at the bottom.						
**Ruthenium complexes**						
24	Grubbs catalyst M102	6 days	N.A.	N.D.	N.D.	< 1
Experiment phenomenon: Upon addition of the red monomer to the dark red catalyst solution in toluene, the mixture turned black. No obvious reaction phenomena were observed, and the black color of the monomer remained unchanged.						
25	Grubbs catalyst M204	6 days	N.A.	N.D.	N.D.	< 1
Experiment phenomenon: Upon addition of the red monomer to the dark red catalyst solution in toluene, the mixture turned black. No obvious reaction phenomena were observed, and the black color of the monomer remained unchanged.						
26	$[RuCl_2(p\text{-}cymene)]_2$	< 10 s	100%	N.D.	N.D.	< 1

Experiment phenomenon: Upon addition of the red monomer to the yellow catalyst solution in toluene (with yellow precipitate at the bottom), an immediate exothermic reaction occurred, accompanied by vigorous gas evolution. During this period, the color of the reaction mixture gradually changed from red to yellow.						
**Rhodium complex**						
27	$Rh_2(OAc)_4$	6 days	N.A.	N.D.	N.D.	< 1
Experiment phenomenon: Upon addition of the red monomer to the black catalyst suspension in toluene, a small amount of gas was released, turning the solution into an opaque black mixture, making it difficult to observe further changes						
**Copper complex**						
28	$Cu(MeCN)_4PF_6$	< 5 s	100%	N.D.	N.D.	< 1
Experiment phenomenon: Upon addition of the red monomer to the colorless catalyst solution in toluene (with white precipitate at the bottom), vigorous gas evolution was observed. The reaction mixture instantly turned yellow.						

[a]C1 polymerizations were performed in toluene at room temperature with $[1]_0 = 0.2$ M, $[1]_0/[\text{Cat.}]_0 = 30/1$. [b]Reaction progress was monitored by color change from red to yellow. [c]Molecular weights were determined by SEC in THF, calibrated with polystyrene standards. [d]Polymer yields were calculated based on the weight of the dried product after precipitation in methanol.

Table S4. C1 polymerization of functional diazo monomers initiated by $C(Ph)_3^+BF_4^-$.[a]

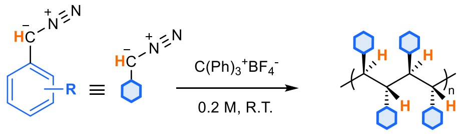

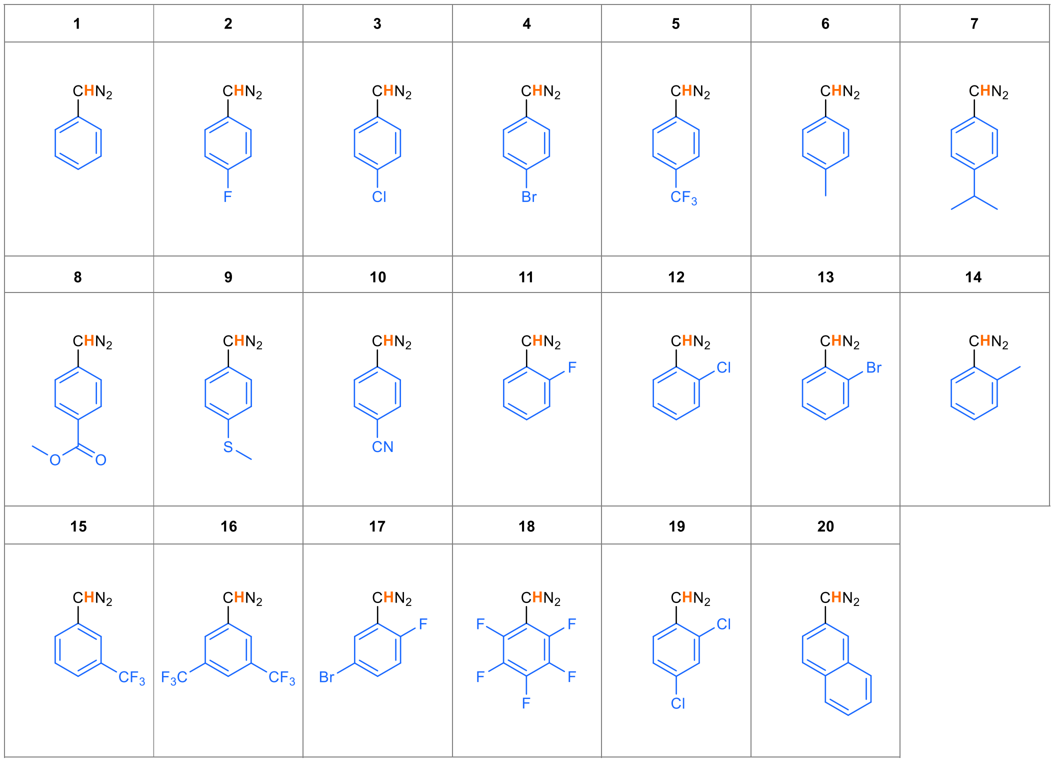

Entry	M	Time[b]	Conv. (%)[b]	$M_{n,SEC}$ (kg mol^{-1})[c]	*Đ*[c]	Yield (%)[d]
1	1	< 5 s	100	12.8	1.52	88
2	2	< 5 s	100	13.1	1.47	28
3	3	< 5 s	100	8.9	1.45	42

4	4	< 5 s	100	7.6	1.58	58
5	5	< 5 s	100	35.2[e]	5.93[e]	29
6	6	< 5 s	100	12.7	1.20	24
7	7	< 5 s	100	14.2	1.18	25
8	8	< 5 s	100	10.3	1.77	62
9	9	24 h	N.A.	N.D.	N.D.	< 1
10	10	24 h	100	1.4[e]	2.00[e]	22
11	11	< 5 s	100	7.0	1.46	79
12	12	< 5 s	100	3.2	1.47	67
13	13	< 5 s	100	2.5	1.39	56
14	14	< 5 s	100	5.1	1.49	37
15	16	< 5 s	100	27.4[e]	2.28[e]	44
16	17	< 5 s	100	33.6[e]	4.49[e]	66
17	18	< 5 s	100	15.2	1.94	93
18	20	< 5 s	100	49.7[e]	1.81[e]	64
19	21	< 5 s	100	4.3	1.39	65
20	22	10 h	100	19.9	1.69	67

[a]C1 polymerizations were performed in toluene at room temperature with $[M]_0 = 0.2$ M, $[M]_0/[Cat.]_0 = 30/1$. [b]Reaction progress was monitored by color change from red to yellow. [c]Molecular weights were determined by SEC in THF, calibrated with polystyrene standards. [d]Polymer yields were calculated based on the weight of the dried product after precipitation in methanol. [e]The polymer exhibits poor or limited solubility in THF.

Table S5. C1 polymerization of functional diazo monomers initiated by Ni(acac)$_2$.[a]

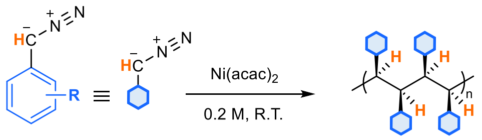

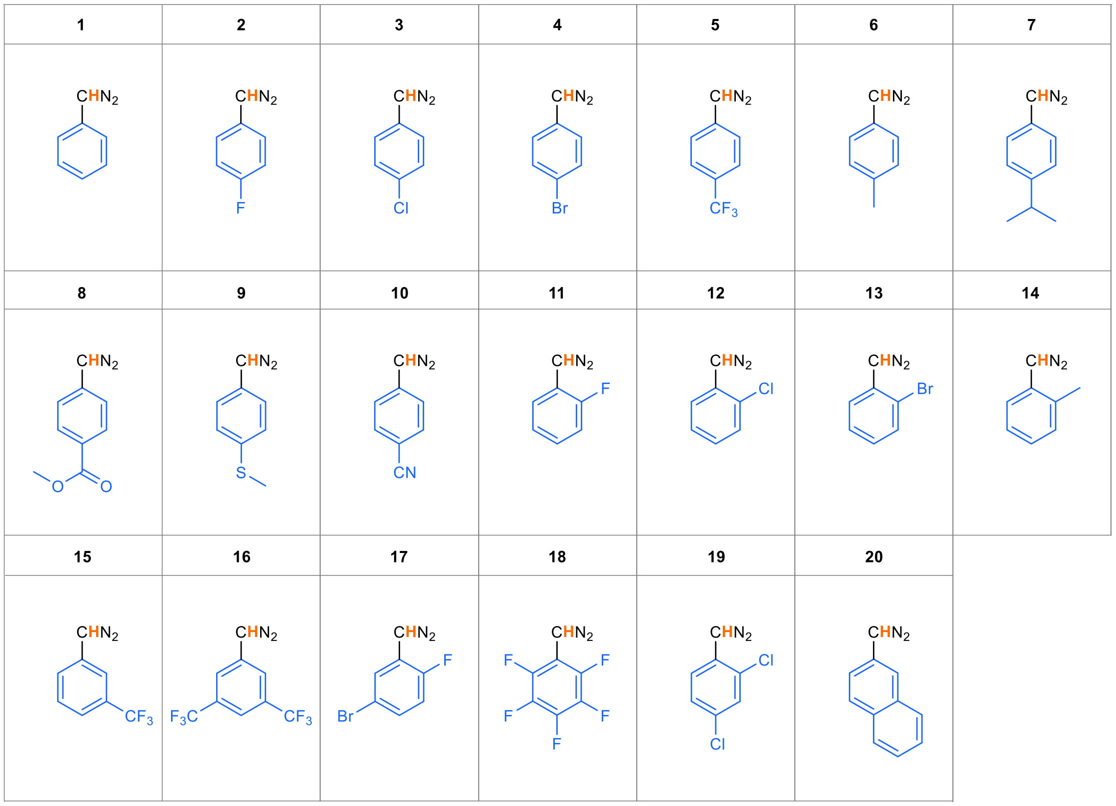

**Entry**	**M**	**Time[b]**	**Conv. (%)[b]**	**$M_{n,SEC}$ (kg mol^{-1})[c]**	**Đ[c]**	**Yield (%)[d]**
1	1	10 h	100	8.4[e]	1.42[e]	95
2	2	1 min	100	10.1[e]	1.26[e]	75
3	3	5 min	100	8.6	1.30	79
4	4	2 h	100	10.7	1.30	74
5	5	2 min	100	19.4[e]	1.40[e]	96
6	6	10 min	100	14.4	1.31	96
7	7	2 min	100	20.0	1.28	94
8	8	5 days	100	22.1[e]	1.28[e]	95
9	9	2 h	100	13.2	1.35	96
10	10	24 h	100	N.D.	N.D.	< 1
11	11	2 h	100	14.5[e]	1.24[e]	96
12	12	24 h	100	N.D.	N.D.	< 1
13	13	24 h	100	N.D.	N.D.	< 1
14	14	24 h	100	N.D.	N.D.	< 1
15	16	2 min	100	15.7[e]	1.30[e]	85
16	17	10 min	100	10.3[e]	1.62[e]	90
17	18	2 h	100	11.6	1.52	85
18	20	24 h	100	54.9[e]	1.64[e]	61
19	21	24 h	100	N.D.	N.D.	< 1

20	22	4 days	100	11.7[e]	1.75[e]	72

[a]C1 polymerizations were performed in toluene at room temperature with $[M]_0 = 0.2$ M, $[M]_0/[Cat.]_0 = 30/1$. [b]Reaction progress was monitored by a visible color change from red to yellow upon exposure to air. [c]Molecular weights were determined by SEC in THF, calibrated with polystyrene standards. [d]Polymer yields were calculated based on the weight of the dried product after precipitation in methanol. [e]The polymer exhibits poor or limited solubility in THF.

Table S6. C1 polymerization of (diazomethyl)benzene (1) initiated by $C(Ph)_3^+BF_4^-$ with MeOH.[a]

Entry	$[M]_0/[Cat.]_0/[MeOH]_0$	Time[b]	Conv. (%)[b]	$M_{n,SEC}$ (kg mol^{-1})[c]	$Đ$[c]	Yield (%)[d]
1	30/1/0	< 5 s	100	12.8	1.52	88
2	30/1/1	< 5 s	100	12.1	1.52	85
3	30/1/2	< 5 s	100	10.2	1.72	64

[a]C1 polymerizations were performed in toluene at room temperature with $[M]_0 = 0.2$ M. [b]Reaction progress was monitored by color change from red to yellow. [c]Molecular weights were determined by SEC in THF, calibrated with polystyrene standards. [d]Polymer yields were calculated based on the weight of the dried product after precipitation in methanol.

Table S7. Elemental analysis results of polymers synthesized using $C(Ph)_3^+BF_4^-$ as a catalyst.

		Element analysis				Theoretical results					
Entry	Polymer	N wt%[a]	C wt%	H wt%	S wt%	N wt%[a]	C wt%	H wt%	S wt%	C/H ratio (theo.)[b]	C/H ratio (calcd.)[c]
1	P1	0.21	92.26	6.55	0	0	93.33	6.67	0	7/6	7/5.96
2	P2	0.10	74.88	4.35	0	0	77.75	4.63	0	7/5	7/4.88
3	P3	0.23	68.57	4.22	0	0	67.74	4.03	0	7/5	7/5.15
4	P4	0.25	51.52	3.13	0	0	50.01	2.98	0	7/5	7/5.10
5	P5	0.16	61.24	2.91	0	0	60.75	3.16	0	8/5	8/4.56
6	P6	0.83	86.89	7.01	0	0	92.25	7.69	0	8/8	8/7.75
7	P7	0.79	91.43	8.84	0	0	90.85	9.08	0	10/12	10/11.6
8	P8	0.15	72.88	5.37	0	0	72.95	5.40	0	9/8	9/7.96
9	P10	12.15	81.64	3.94	0	12.17	83.45	4.35	0	8/5/1 (C/H/N)	8/4.63/1.02 (C/H/N)
10	P11	0.24	77.93	4.15	0	0	77.75	4.63	0	7/5	7/4.48
11	P12	0.49	69.05	4.21	0	0	67.74	4.03	0	7/5	7/5.12
12	P13	0.40	53.10	3.21	0	0	50.01	2.98	0	7/5	7/5.08
13	P14	0.48	92.31	7.47	0	0	92.25	7.69	0	8/8	8/7.78
14	P15	0.11	60.38	2.94	0	0	60.75	3.16	0	8/5	8/4.68
15	P16	0.15	53.54	1.47	0	0	47.78	1.77	0	9/4	9/2.97
16	P17	0.17	45.33	2.12	0	0	45.17	2.15	0	7/4	7/3.93
17	P18	0.18	52.33	0.67	0	0	46.67	0.56	0	7/1	7/1.07
18	P19	0.25	54.34	2.69	0	0	53.17	2.53	0	7/4	7/4.16
19	P20	0.61	93.48	5.69	0	0	94.25	5.71	0	11/8	11/8.03

[a]The low nitrogen content observed in elemental analysis is caused by interference from atmospheric nitrogen. [b]The theoretical results were calculated based on the polymer repeat unit with chain-end groups omitted. [c]The C/H ratio was calculated from elemental analysis by fixing the carbon content to its theoretical value, enabling direct comparison with the expected hydrogen content

Table S8. Elemental analysis results of polymers synthesized using $Ni(acac)_2$ as a catalyst.

		Element analysis	Theoretical results		

Entry	Polymer	N wt%[a]	C wt%	H wt%	S wt%	N wt%	C wt%	H wt%	S wt%	C/H ratio (theo.)[b]	C/H ratio (calcd.)[c]
1	P1	0.31	91.55	6.6	0	0	93.33	6.67	0	7/6	7/6.05
2	P2	0.10	76.90	4.85	0	0	77.75	4.63	0	7/5	7/5.30
3	P3	0.11	68.33	4.15	0	0	67.74	4.03	0	7/5	7/5.10
4	P4	0.13	50.53	3.04	0	0	50.01	2.98	0	7/5	7/5.05
5	P5	0.17	60.20	2.76	0	0	60.75	3.16	0	8/5	8/4.40
6	P6	0.45	93.79	7.61	0	0	92.25	7.69	0	8/8	8/7.80
7	P7	0.28	92.91	9.03	0	0	90.85	9.08	0	10/12	10/11.66
8	P8	0.34	68.11	5.08	0	0	72.95	5.40	0	9/8	9/8.06
9	P9	0.51	71.82	5.60	22.44	0	70.57	5.88	23.52	8/8/1 (C/H/S)	8/7.5/0.93 (C/H/S)
10	P11	0.38	78.95	4.85	0	0	77.75	4.63	0	7/5	7/5.16
11	P15	0.10	61.12	2.86	0	0	60.75	3.16	0	8/5	8/4.50
12	P16	0.16	53.00	1.43	0	0	47.78	1.77	0	9/4	9/2.91
13	P17	0.10	46.11	2.02	0	0	45.17	2.15	0	7/4	7/3.68
14	P18	0.71	51.59	0.91	0	0	46.67	0.56	0	7/1	7/1.48
15	P20	0.72	92.80	5.85	0	0	94.25	5.71	0	11/8	11/8.32

[a]The low nitrogen content observed in elemental analysis is caused by interference from atmospheric nitrogen. [b]The theoretical results were calculated based on the polymer repeat unit with chain-end groups omitted. [c]The C/H ratio was calculated from elemental analysis by fixing the carbon content to its theoretical value, enabling direct comparison with the expected hydrogen content

Supplementary Figures

NMR spectra

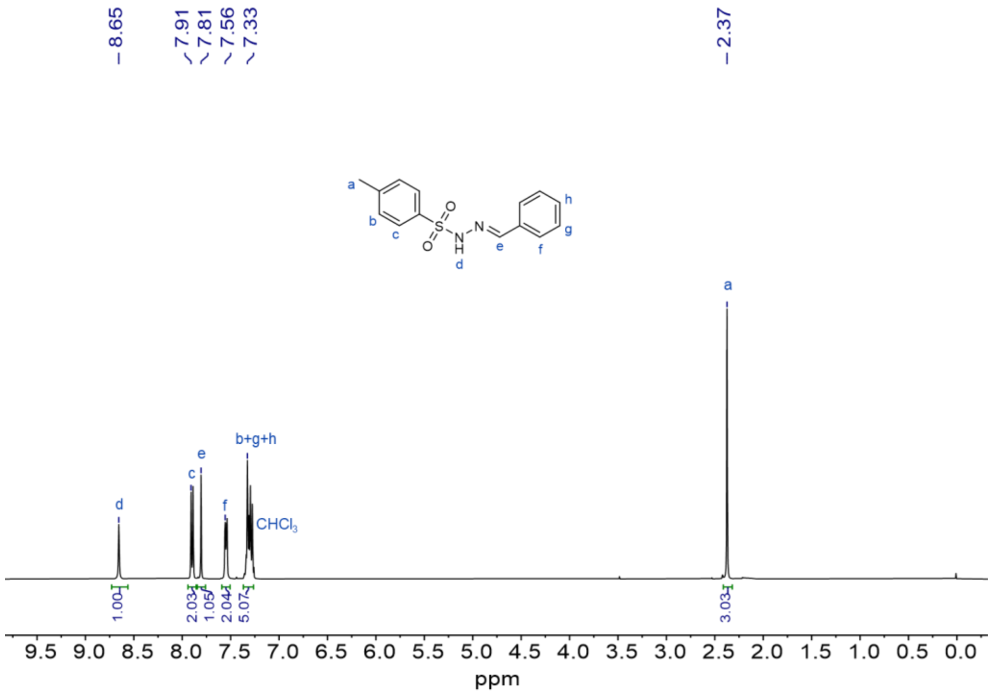

Fig. S1. ^{1}H NMR (400 MHz, $CDCl_3$, 23 °C) spectrum of (*E*)-*N'*-benzylidene-4-methylbenzenesulfonohydrazide.

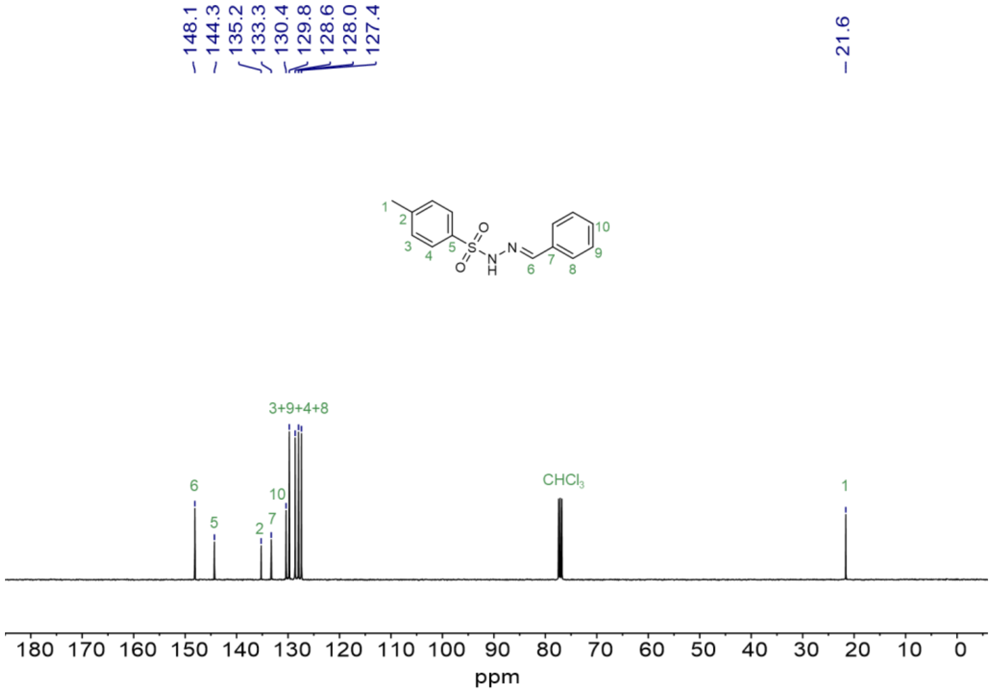

Fig. S2. ^{13}C NMR (100 MHz, $CDCl_3$, 23 °C) spectrum of (*E*)-*N'*-benzylidene-4-methylbenzenesulfonohydrazide.

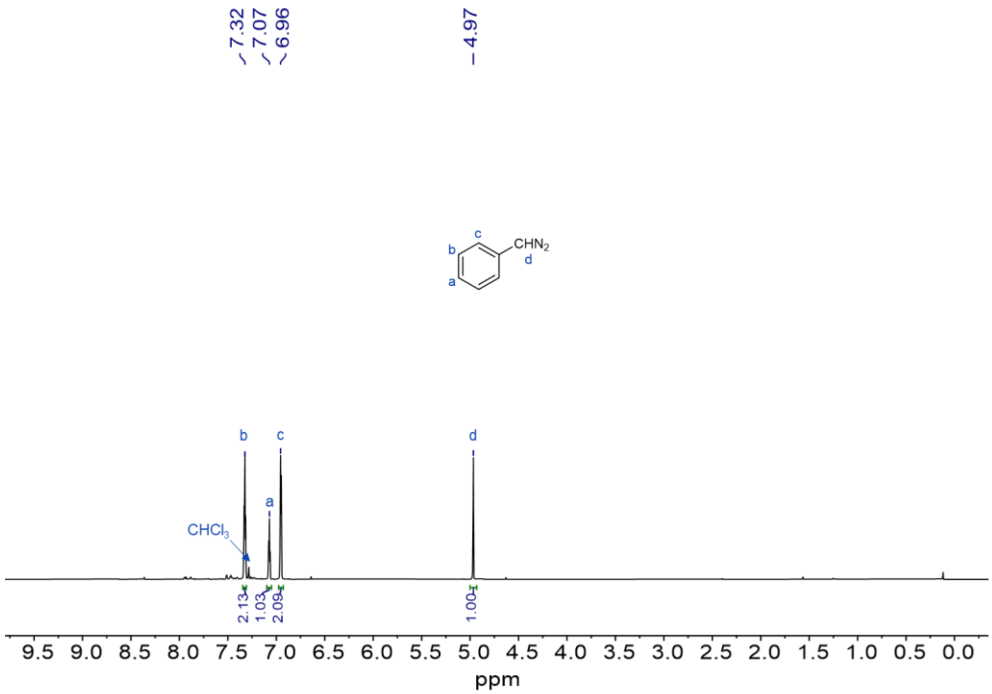

Fig. S3. ^{1}H NMR (400 MHz, $CDCl_3$, 23 ºC) spectrum of (diazomethyl)benzene (**1**).

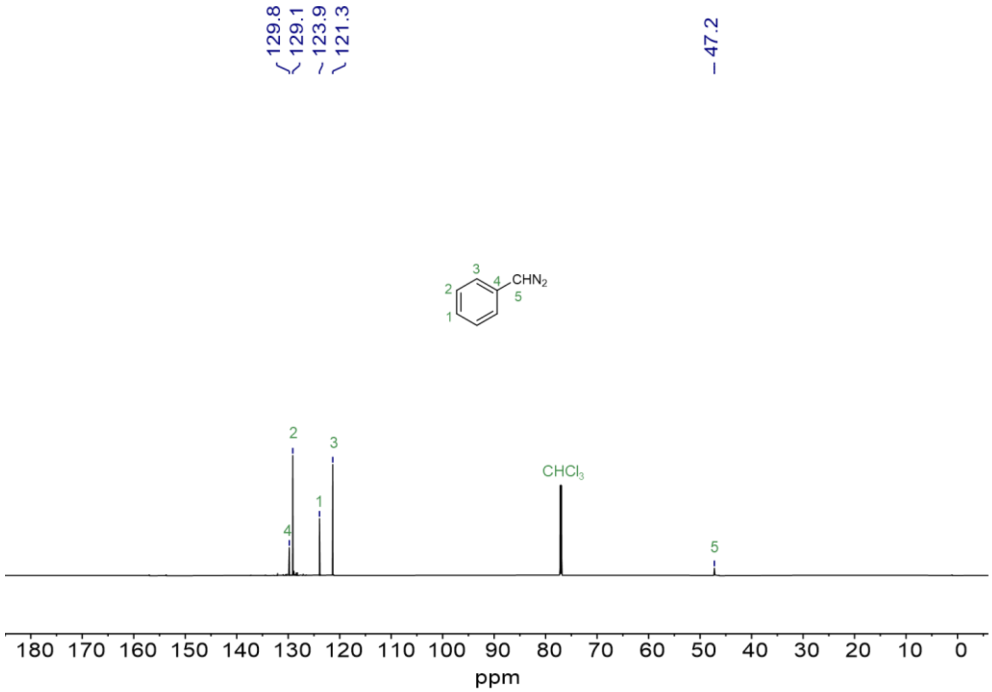

Fig. S4. ^{13}C NMR (100 MHz, $CDCl_3$, 23 ºC) spectrum of (diazomethyl)benzene (**1**).

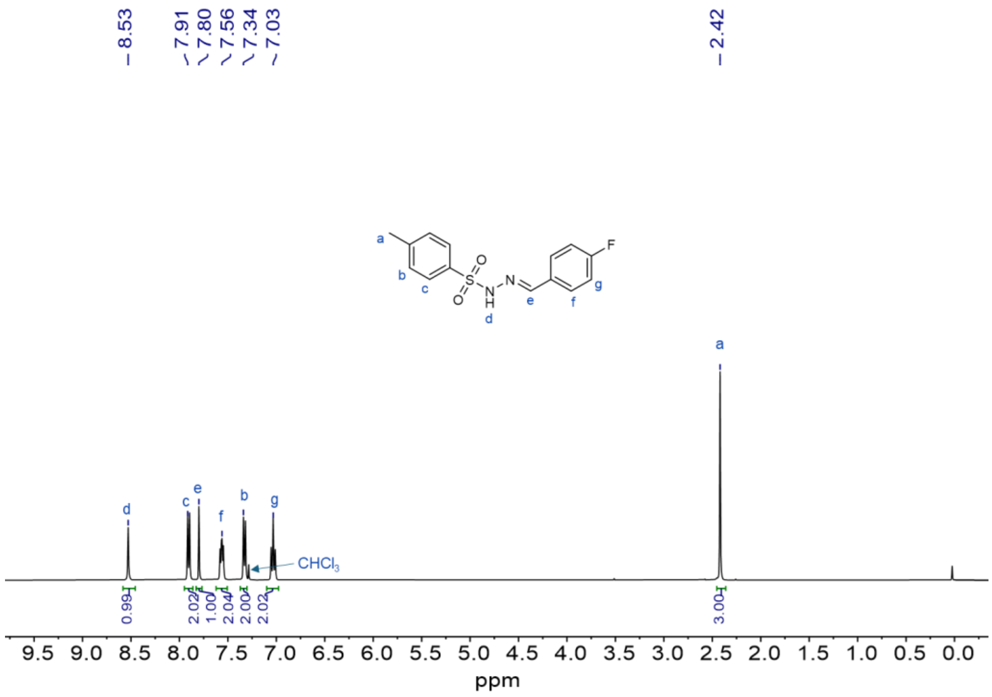

Fig. S5. ^{1}H NMR (400 MHz, $CDCl_3$, 23 ºC) spectrum of (*E*)-*N*'-(4-fluorobenzylidene)-4-methylbenzenesulfonohydrazide.

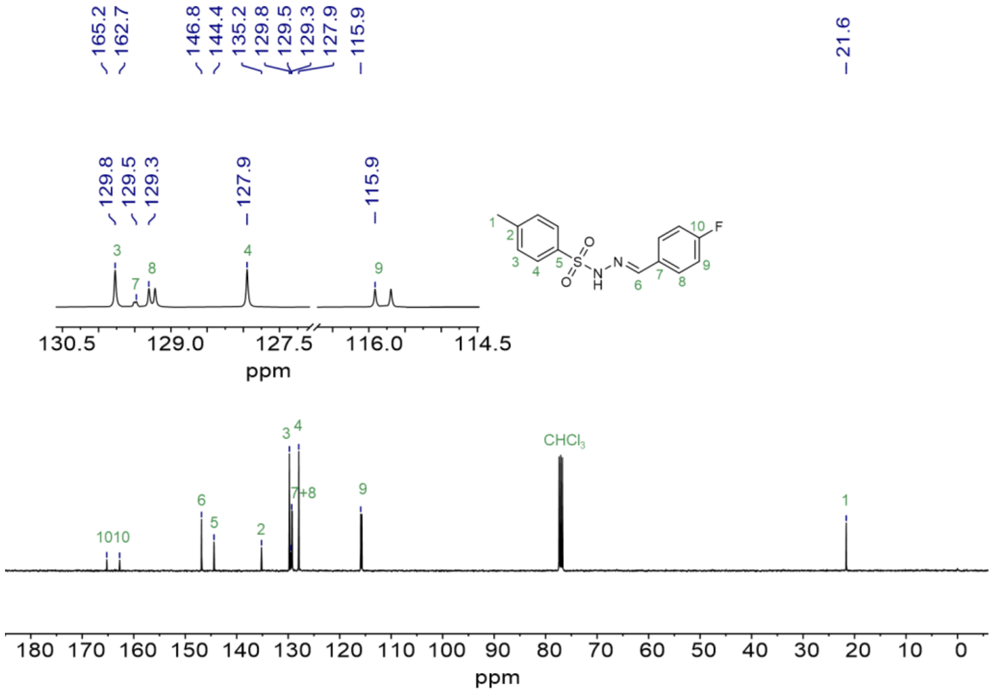

Fig. S6. ^{13}C NMR (100 MHz, $CDCl_3$, 23 ºC) spectrum of (*E*)-*N*'-(4-fluorobenzylidene)-4-methylbenzenesulfonohydrazide (C10 exhibits splitting due to ^{13}C-^{19}F coupling).

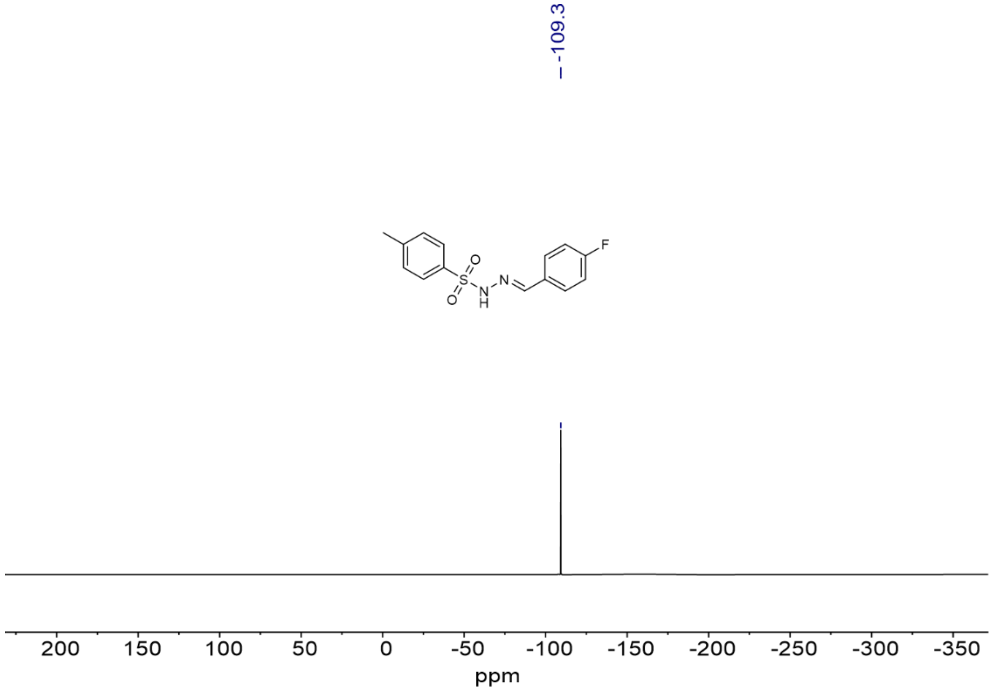

Fig. S7. ^{19}F NMR (377 MHz, $CDCl_3$, 23 ºC) spectrum of (*E*)-*N*'-(4-fluorobenzylidene)-4-methylbenzenesulfonohydrazide.

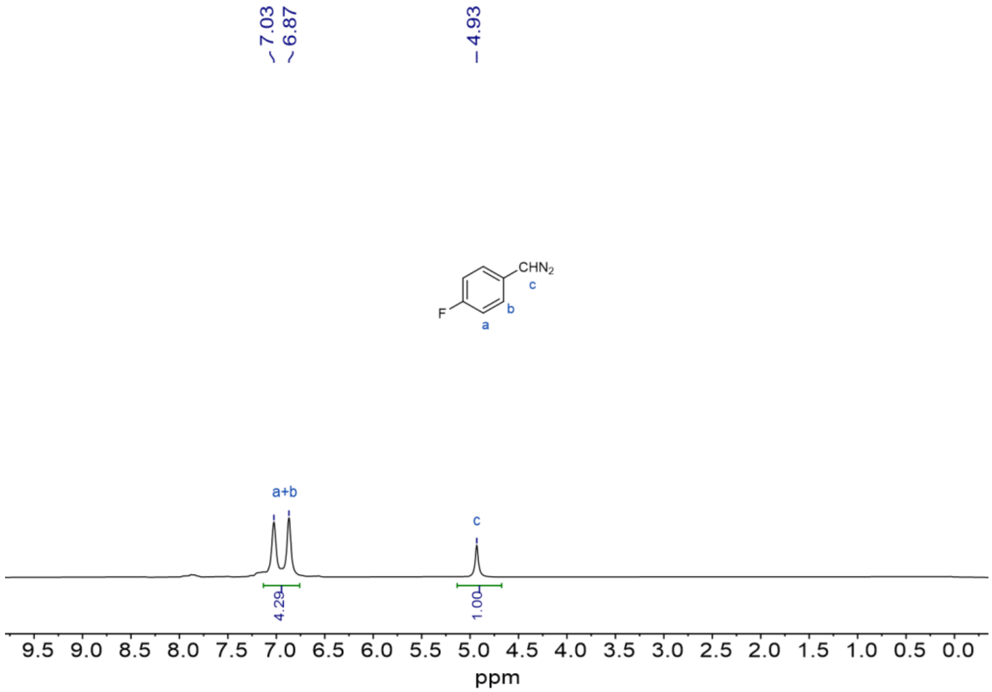

Fig. S8. ^{1}H NMR (400 MHz, $CDCl_3$, 23 ºC) spectrum of 1-(diazomethyl)-4-fluorobenzene (**2**).

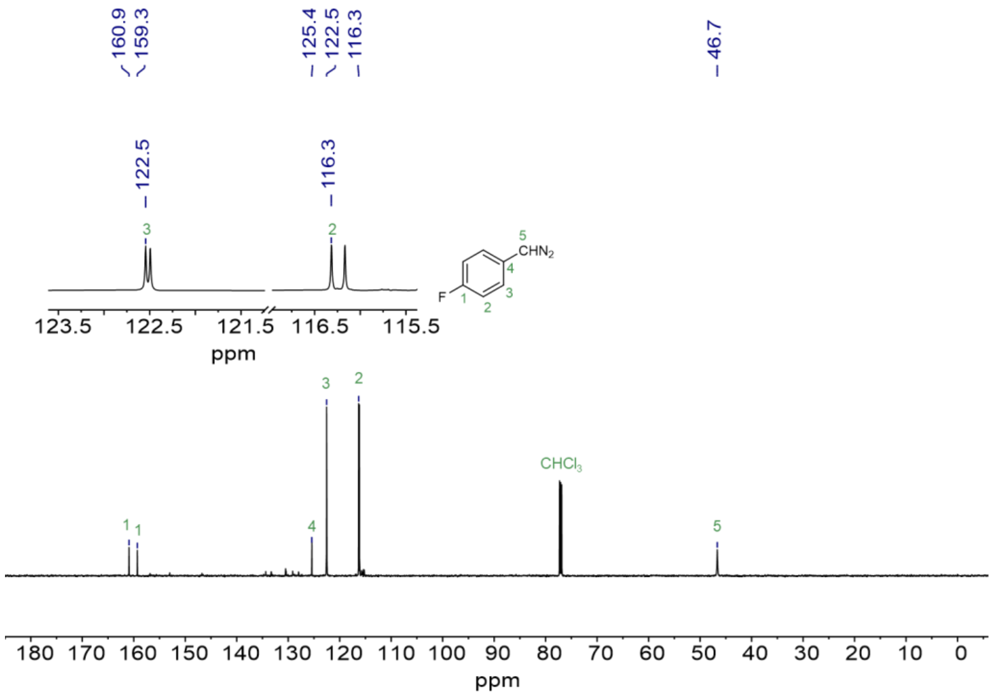

Fig. S9. ^{13}C NMR (100 MHz, $CDCl_3$, 23 °C) spectrum of 1-(diazomethyl)-4-fluorobenzene (**2**) (C1 exhibits splitting due to ^{13}C-^{19}F coupling)

.

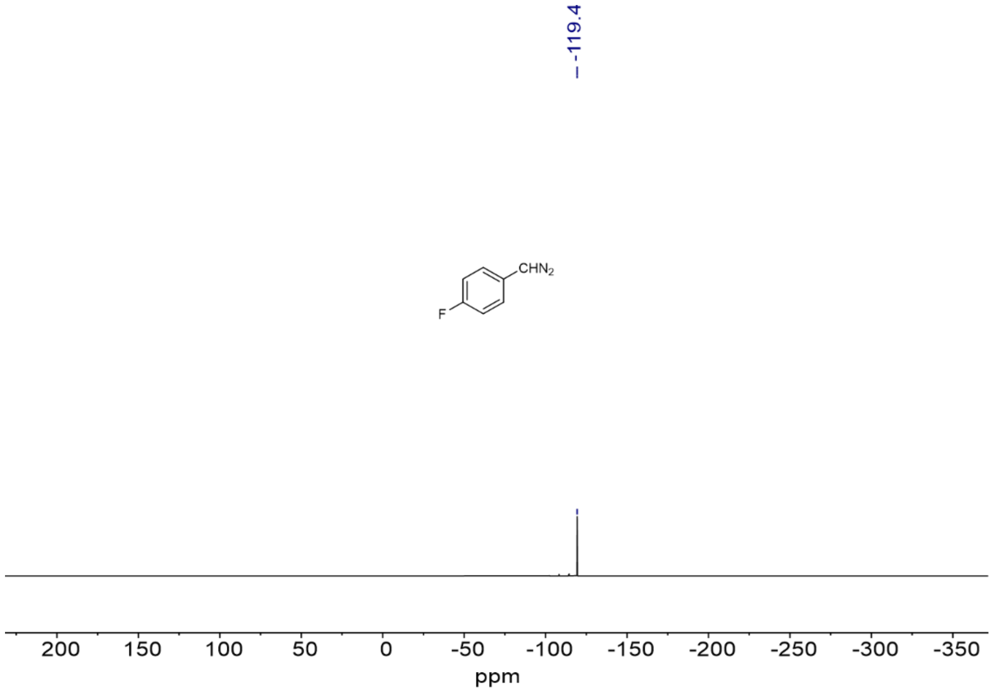

Fig. S10. ^{19}F NMR (377 MHz, $CDCl_3$, 23 °C) spectrum of 1-(diazomethyl)-4-fluorobenzene (**2**).

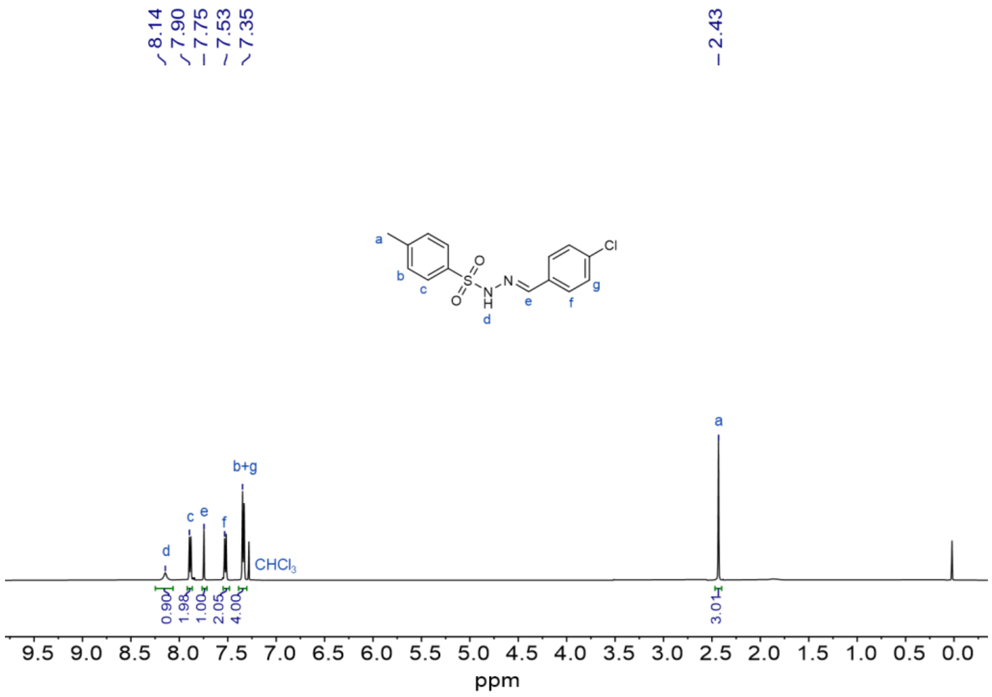

Fig. S11. ^{1}H NMR (400 MHz, $CDCl_3$, 23 ^{o}C) spectrum of (*E*)-*N*'-(4-chlorobenzylidene)-4-methylbenzenesulfonohydrazide.

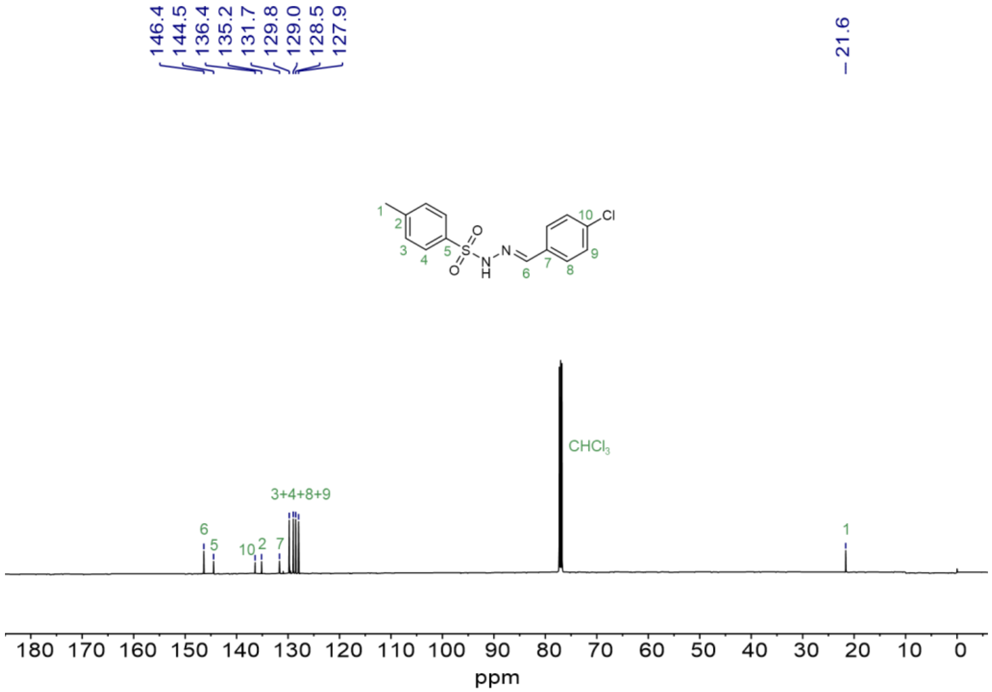

Fig. S12. ^{13}C NMR (100 MHz, $CDCl_3$, 23 ^{o}C) spectrum of (*E*)-*N*'-(4-chlorobenzylidene)-4-methylbenzenesulfonohydrazide.

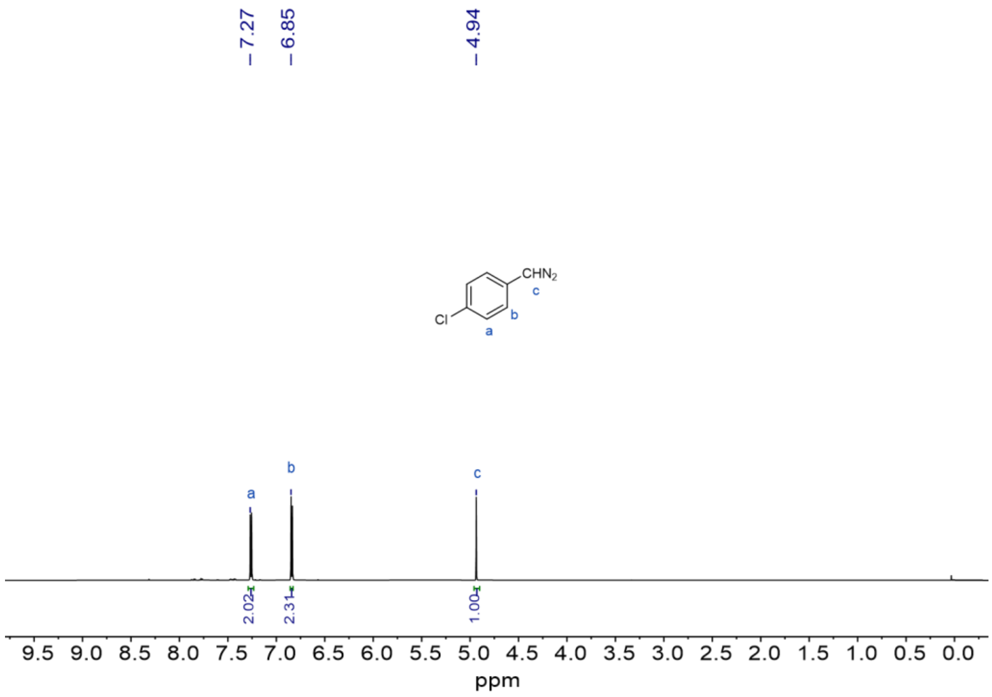

Fig. S13. ^{1}H NMR (400 MHz, $CDCl_3$, 23 °C) spectrum of 1-chloro-4-(diazomethyl)benzene (**3**).

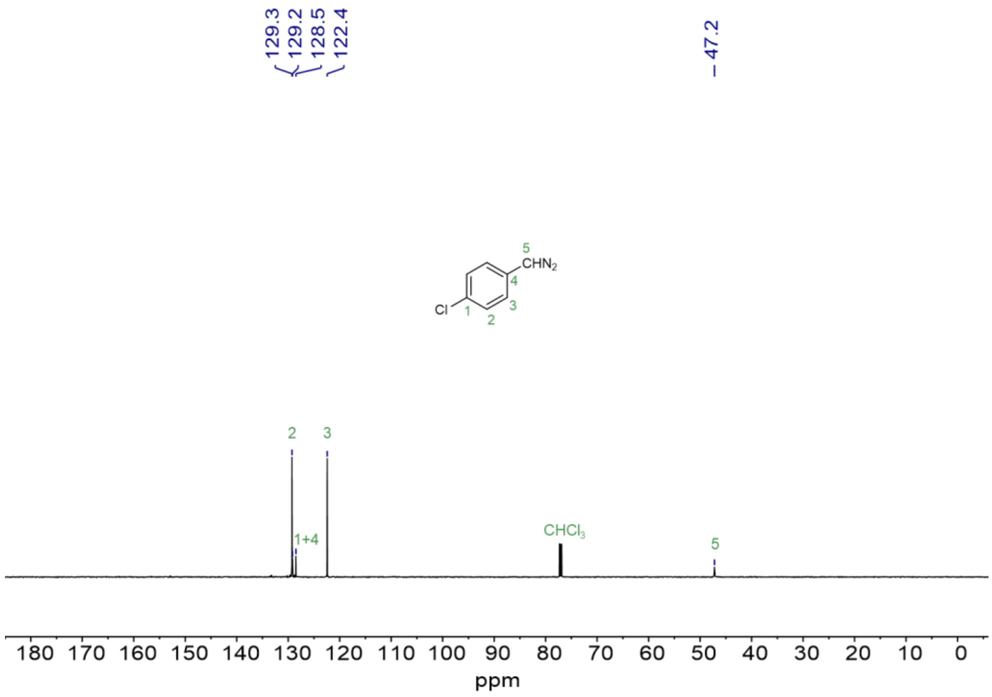

Fig. S14. ^{13}C NMR (100 MHz, $CDCl_3$, 23 °C) spectrum of 1-chloro-4-(diazomethyl)benzene (**3**).

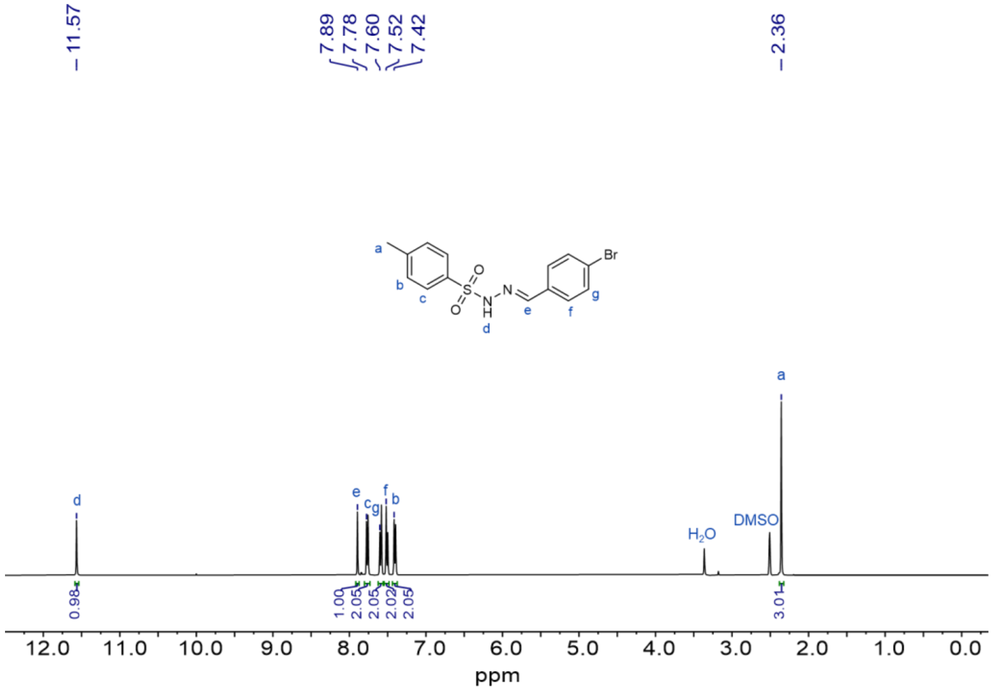

Fig. S15. ^{1}H NMR (400 MHz, DMSO-d_6, 23 °C) spectrum of (*E*)-*N*'-(4-bromobenzylidene)-4-methylbenzenesulfonohydrazide.

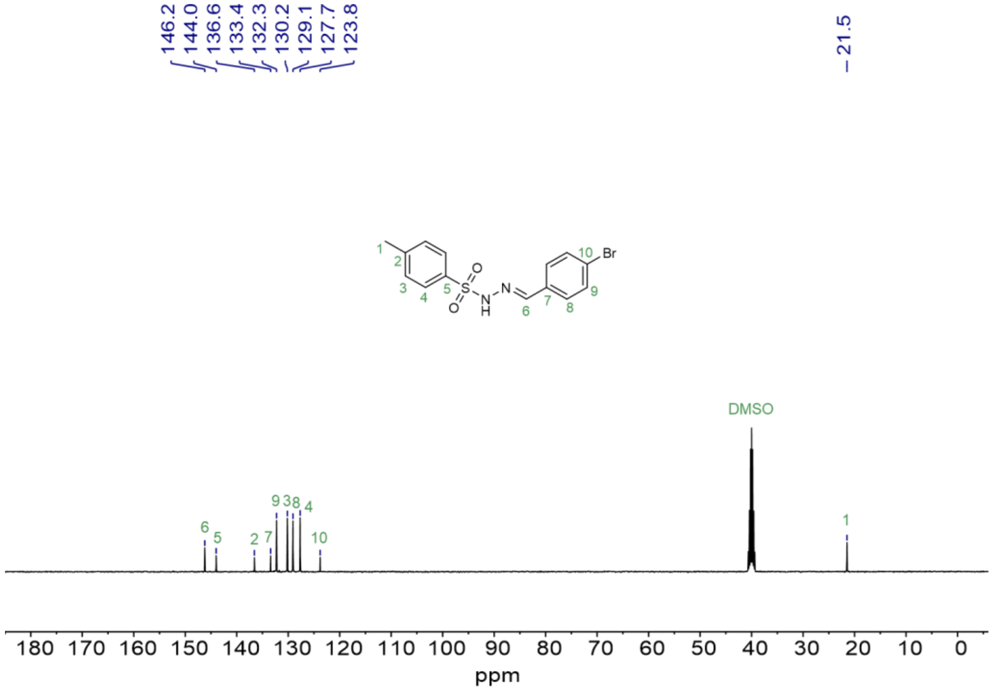

Fig. S16. ^{13}C NMR (100 MHz, DMSO-d_6, 23 °C) spectrum of (*E*)-*N*'-(4-bromobenzylidene)-4-methylbenzenesulfonohydrazide.

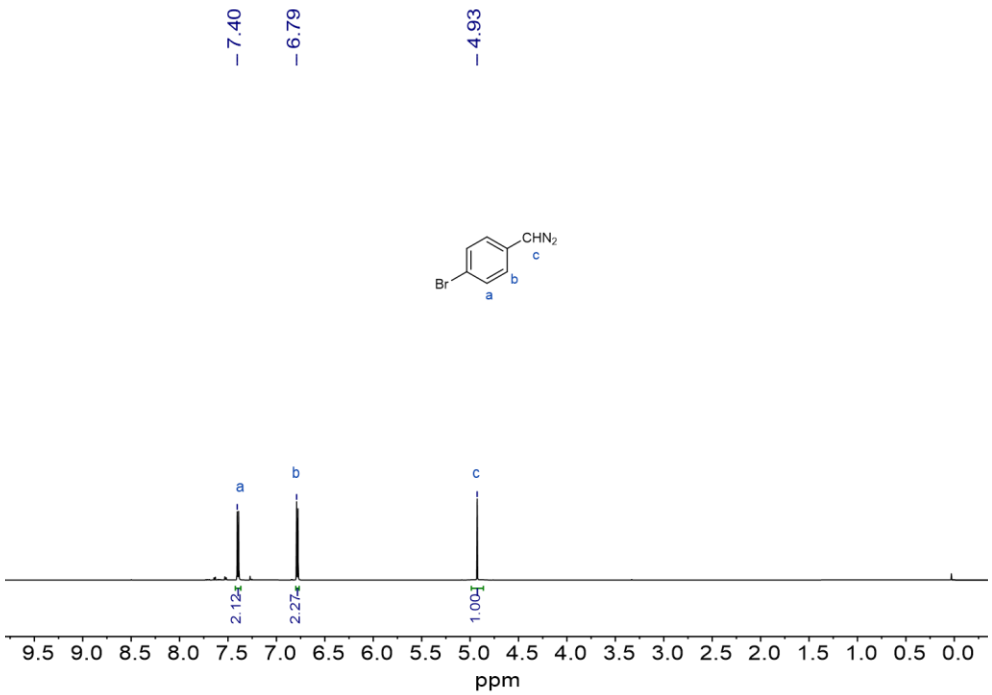

Fig. S17. ^{1}H NMR (400 MHz, $CDCl_3$, 23 °C) spectrum of 1-bromo-4-(diazomethyl)benzene (**4**).

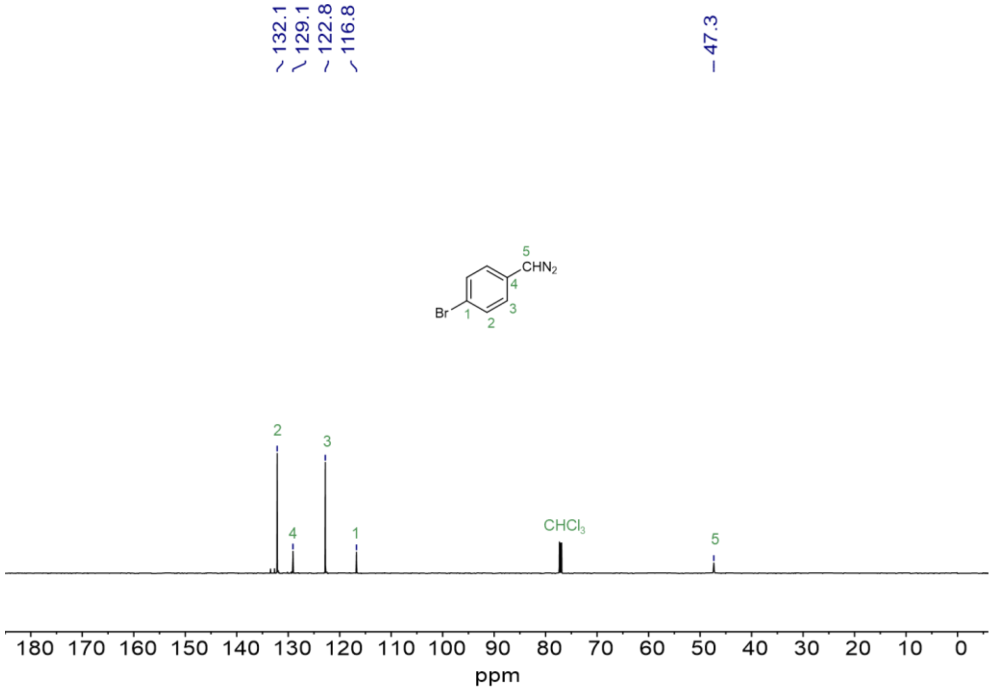

Fig. S18. ^{13}C NMR (100 MHz, $CDCl_3$, 23 °C) spectrum of 1-bromo-4-(diazomethyl)benzene (**4**).

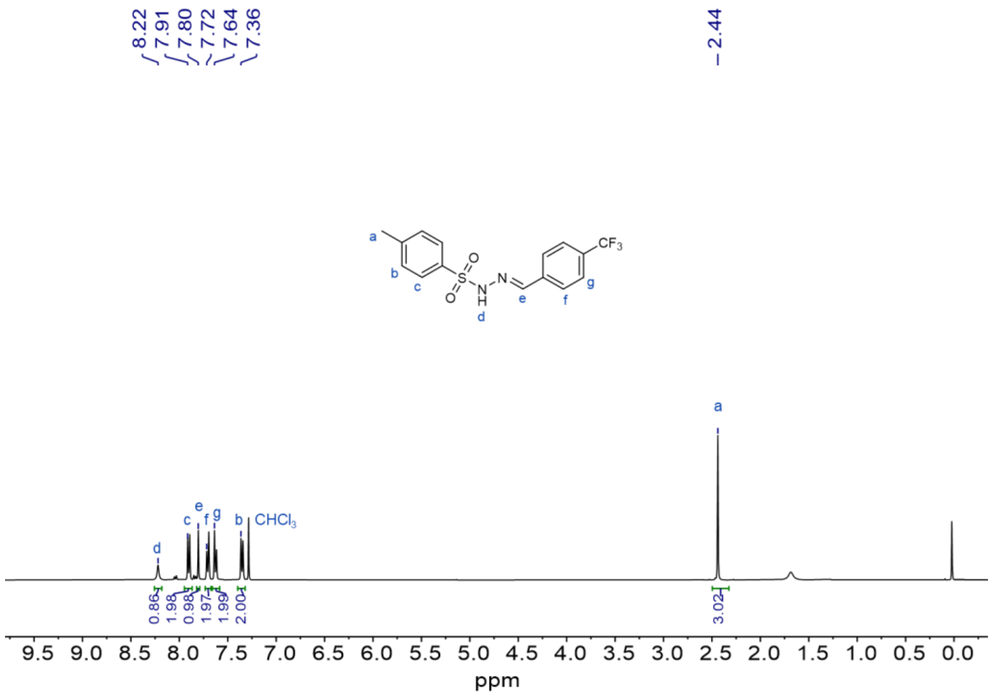

Fig. S19. ^{1}H NMR (400 MHz, $CDCl_3$, 23 °C) spectrum of (*E*)-4-methyl-*N*'-(4-(trifluoromethyl)benzylidene)benzenesulfonohydrazide.

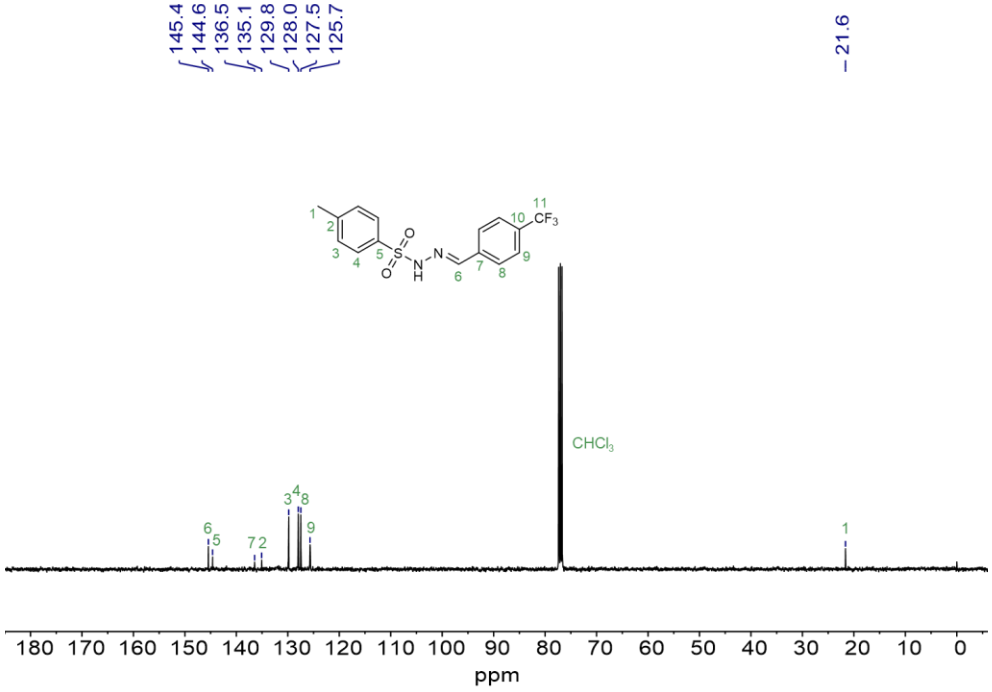

Fig. S20. ^{13}C NMR (100 MHz, $CDCl_3$, 23 °C) spectrum of (*E*)-4-methyl-*N*'-(4-(trifluoromethyl)benzylidene)benzenesulfonohydrazide. (C10 and C11 show strong ^{13}C–^{19}F coupling with the fluorine atom, resulting in complex splitting that hinders the resolution and assignment of individual carbon signals. C8 and C9 are disturbed by the fluorine atom and split).

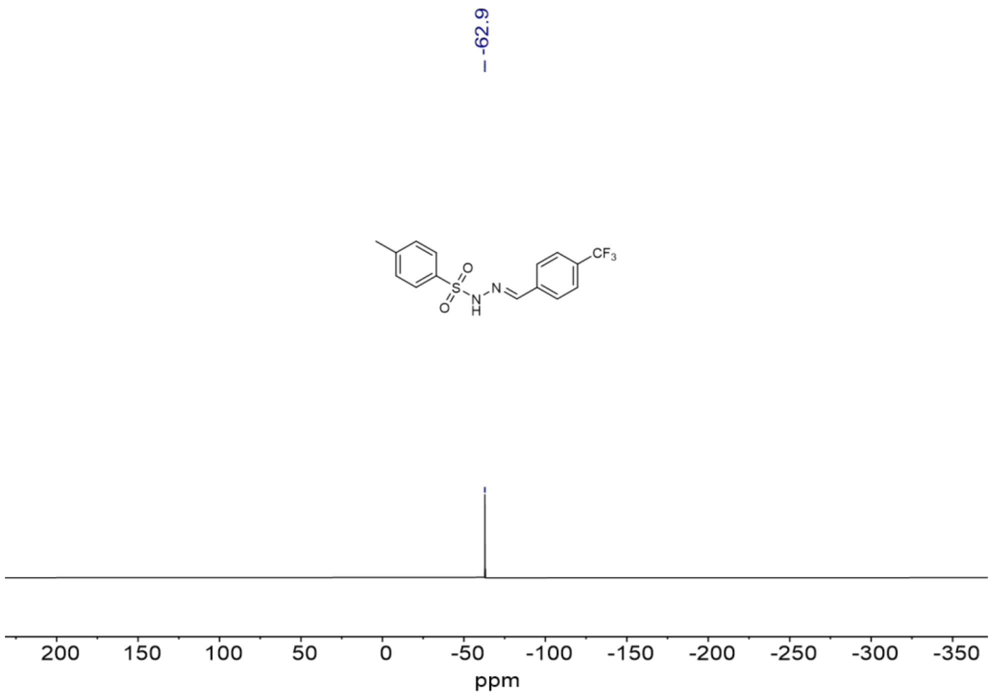

Fig. S21. ^{19}F NMR (377 MHz, $CDCl_3$, 23 °C) spectrum of (*E*)-4-methyl-*N'*-(4-(trifluoromethyl)benzylidene)benzenesulfonohydrazide.

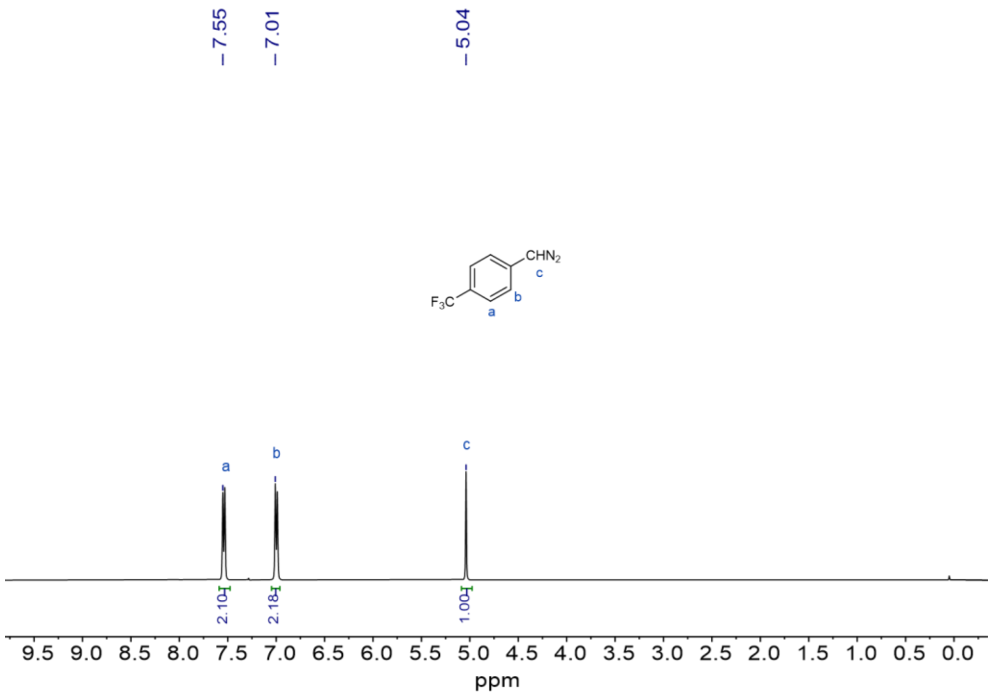

Fig. S22. ^{1}H NMR (400 MHz, $CDCl_3$, 23 °C) spectrum of 1-(diazomethyl)-4-(trifluoromethyl)benzene (**5**).

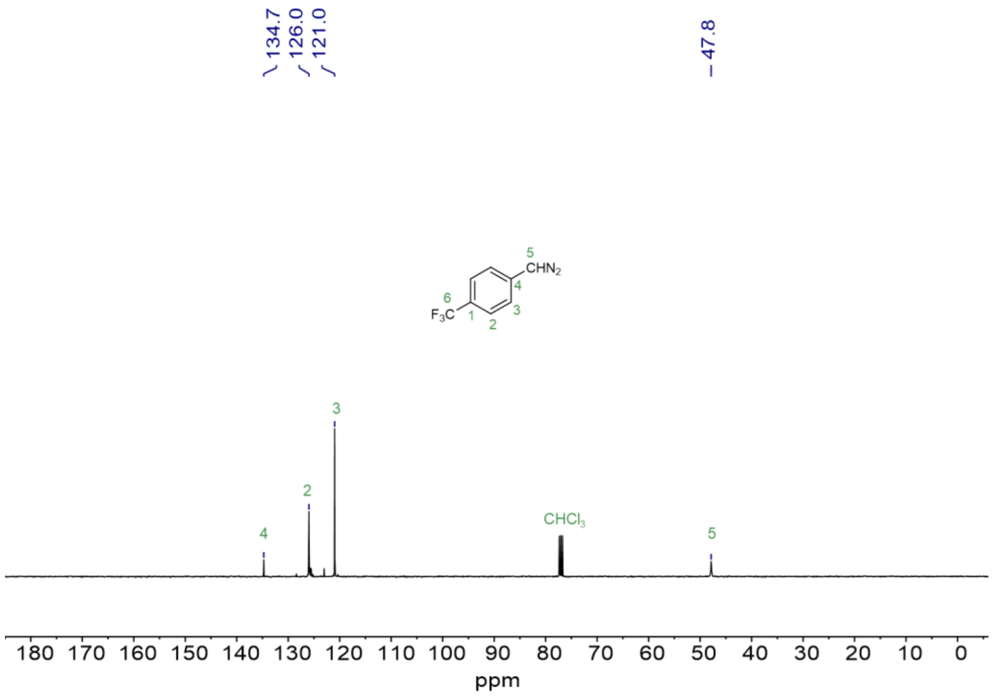

Fig. S23. ^{13}C NMR (100 MHz, $CDCl_3$, 23 ºC) spectrum of 1-(diazomethyl)-4-(trifluoromethyl)benzene (**5**). (C1 and C6 show strong ^{13}C–^{19}F coupling with the fluorine atom, resulting in complex splitting that hinders the resolution and assignment of individual carbon signals. C2 is disturbed by the fluorine atom and splits).

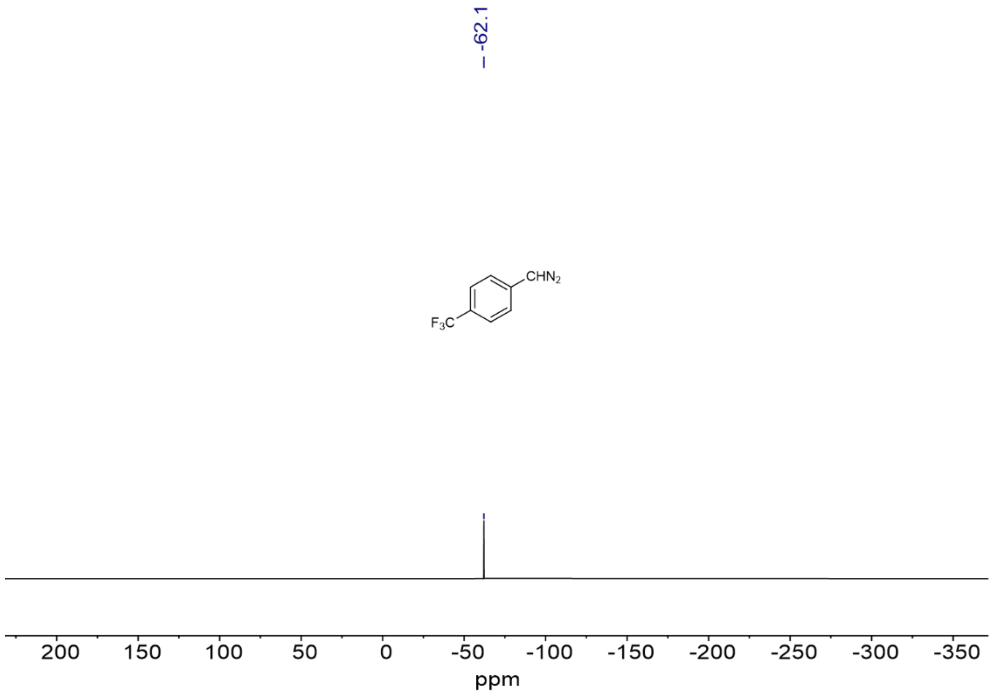

Fig. S24. ^{19}F NMR (377 MHz, $CDCl_3$, 23 ºC) spectrum of 1-(diazomethyl)-4-(trifluoromethyl)benzene (**5**).

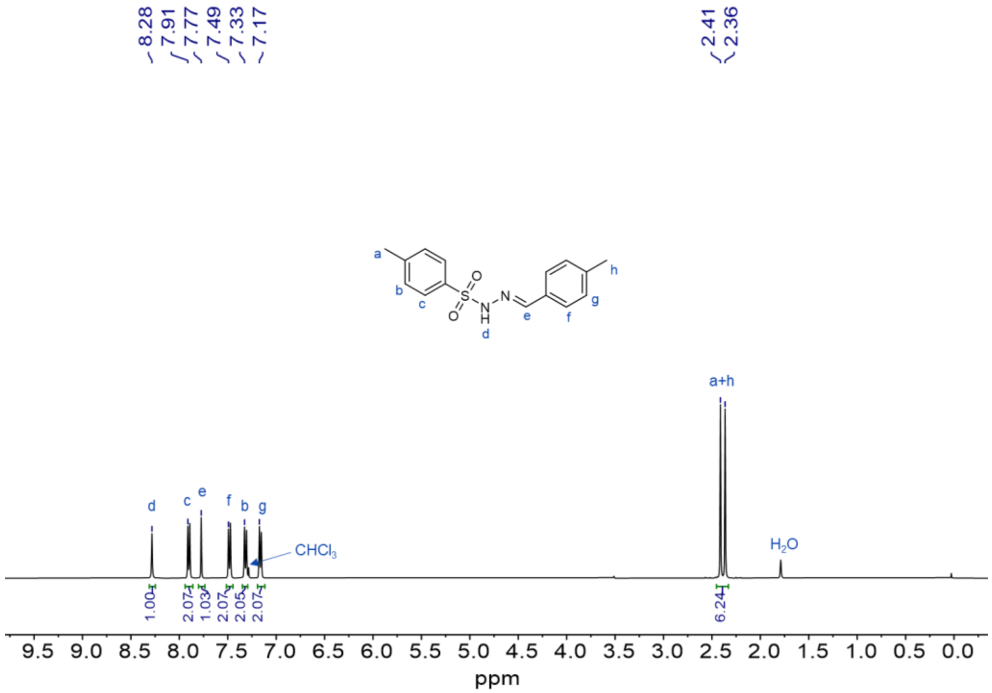

Fig. S25. ^{1}H NMR (400 MHz, $CDCl_3$, 23 ºC) spectrum of (*E*)-4-methyl-*N*'-(4-methylbenzylidene)benzenesulfonohydrazide.

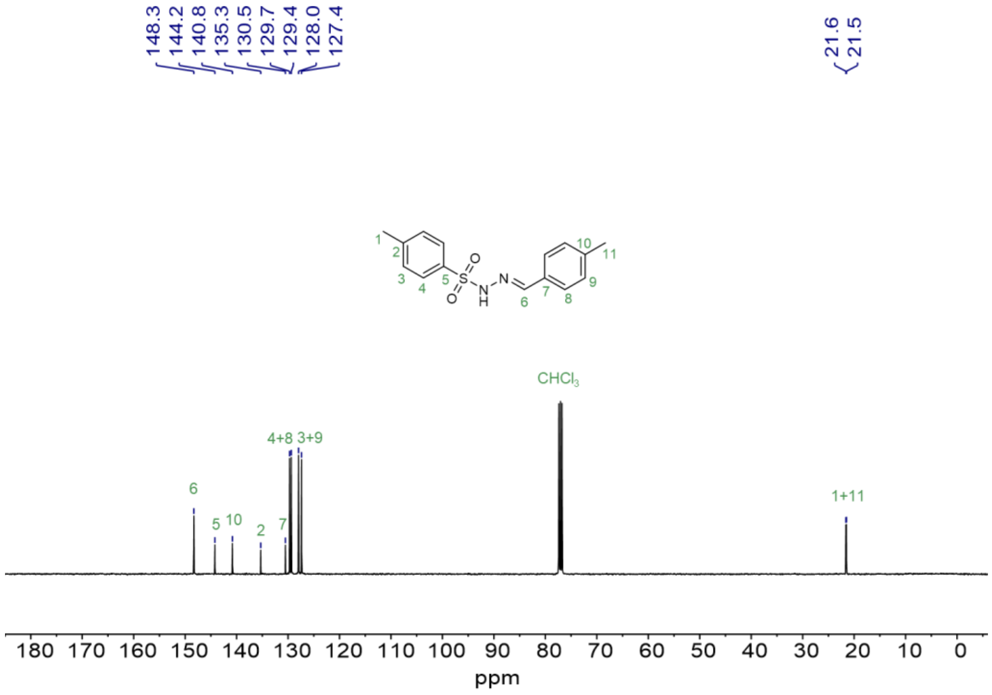

Fig. S26. ^{13}C NMR (100 MHz, $CDCl_3$, 23 ºC) spectrum of (*E*)-4-methyl-*N*'-(4-methylbenzylidene)benzenesulfonohydrazide.

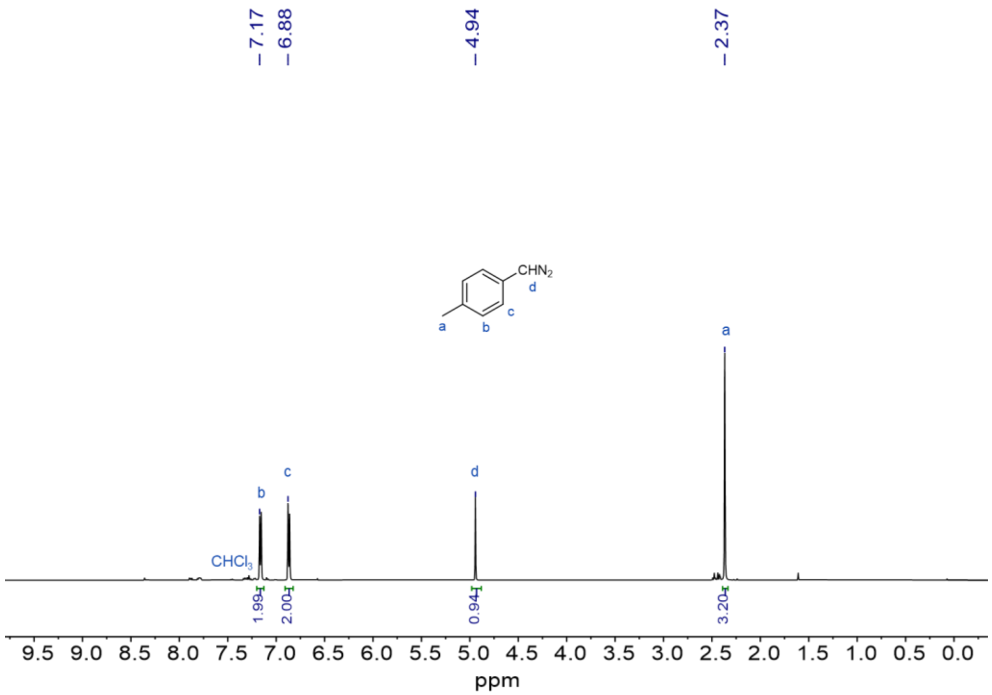

Fig. S27. ^{1}H NMR (400 MHz, $CDCl_3$, 23 °C) spectrum of 1-(diazomethyl)-4-methylbenzene (**6**).

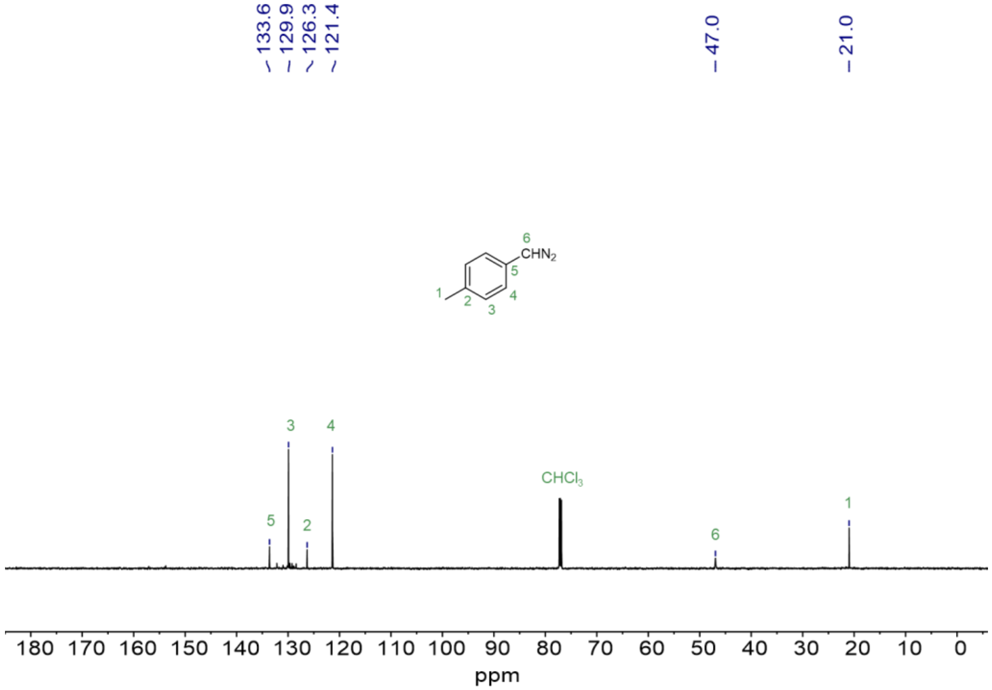

Fig. S28. ^{13}C NMR (100 MHz, $CDCl_3$, 23 °C) spectrum of 1-(diazomethyl)-4-methylbenzene (**6**).

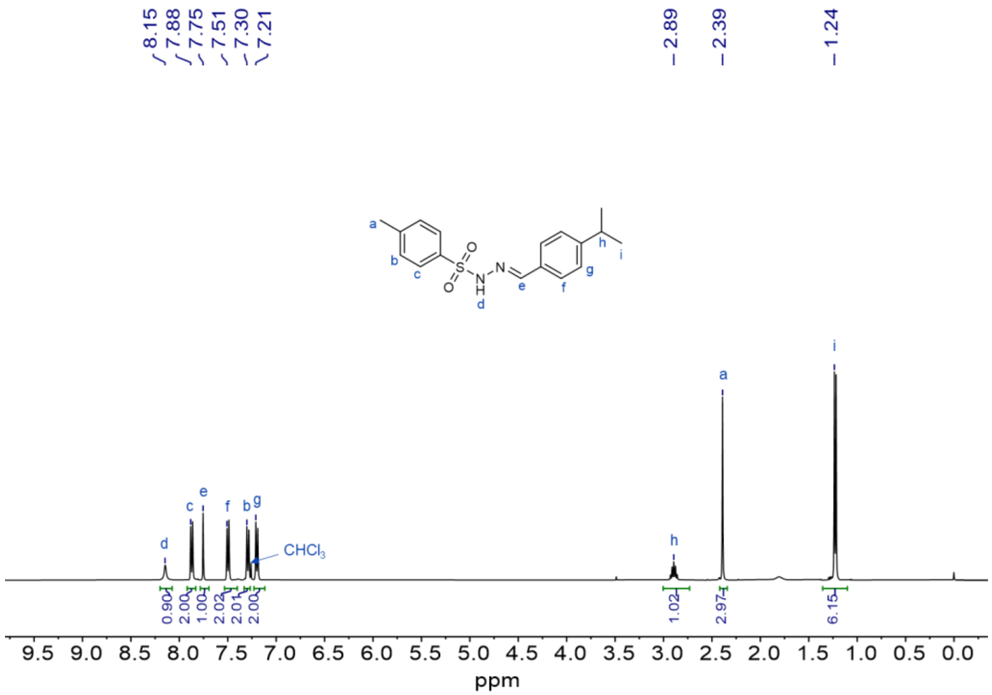

Fig. S29. ^{1}H NMR (400 MHz, $CDCl_3$, 23 ºC) spectrum of (*E*)-*N'*-(4-isopropylbenzylidene)-4-methylbenzenesulfonohydrazide.

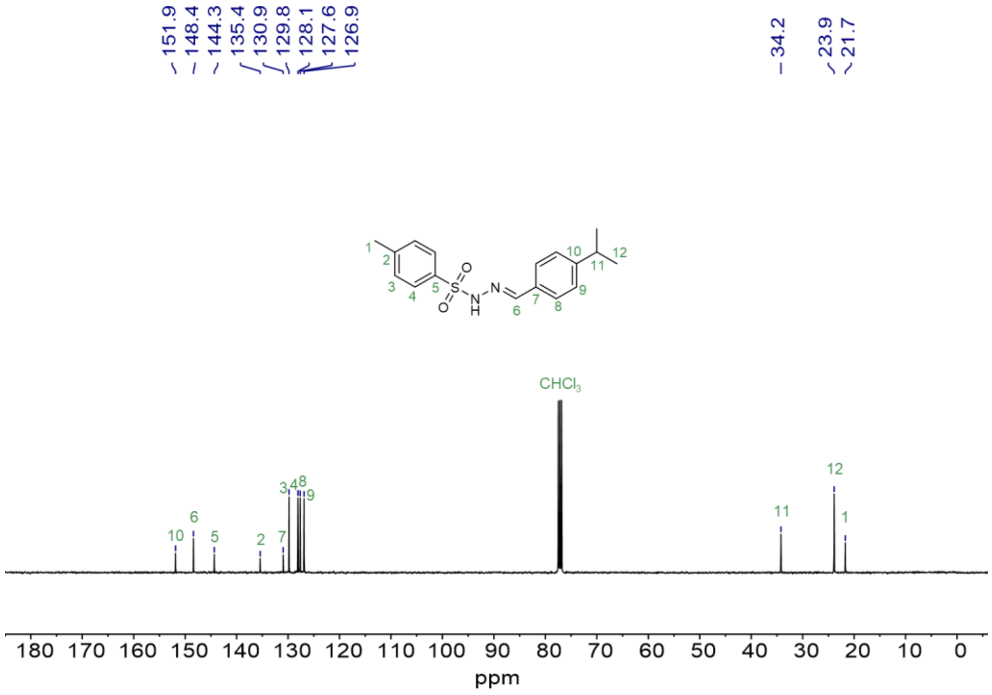

Fig. S30. ^{13}C NMR (100 MHz, $CDCl_3$, 23 ºC) spectrum of (*E*)-*N'*-(4-isopropylbenzylidene)-4-methylbenzenesulfonohydrazide.

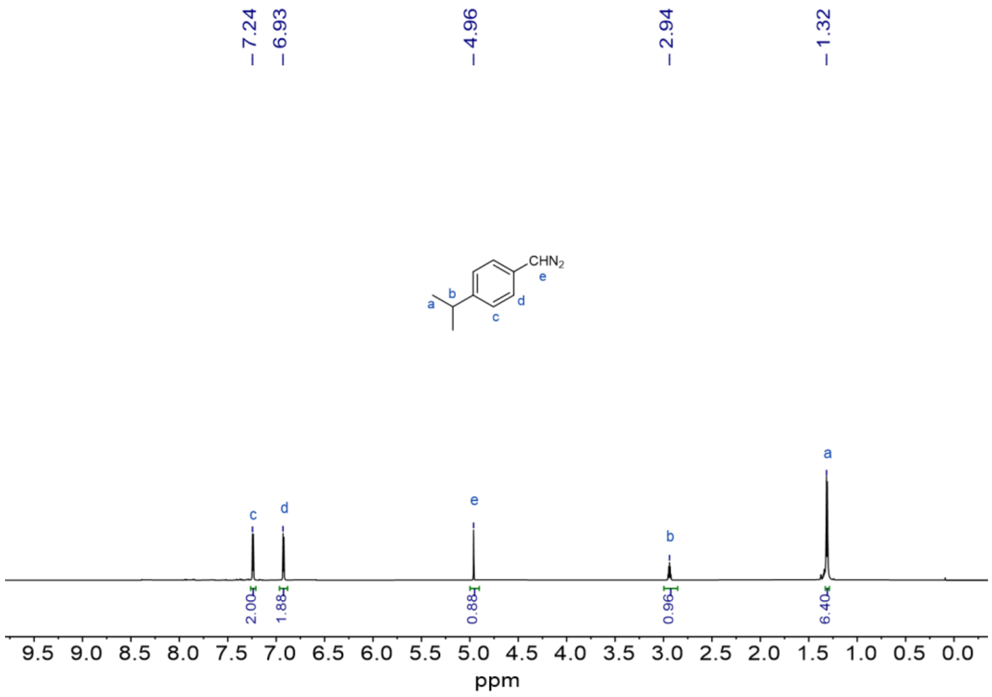

Fig. S31. ^{1}H NMR (400 MHz, $CDCl_3$, 23 °C) spectrum of 1-(diazomethyl)-4-isopropylbenzene (**7**).

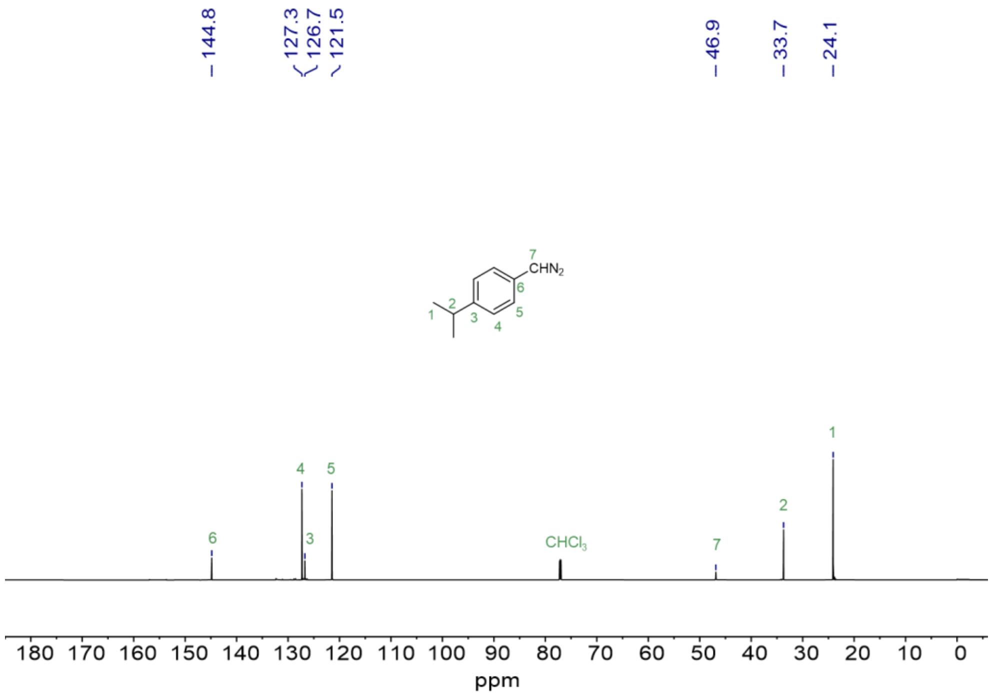

Fig. S32. ^{13}C NMR (100 MHz, $CDCl_3$, 23 °C) spectrum of 1-(diazomethyl)-4-isopropylbenzene (**7**).

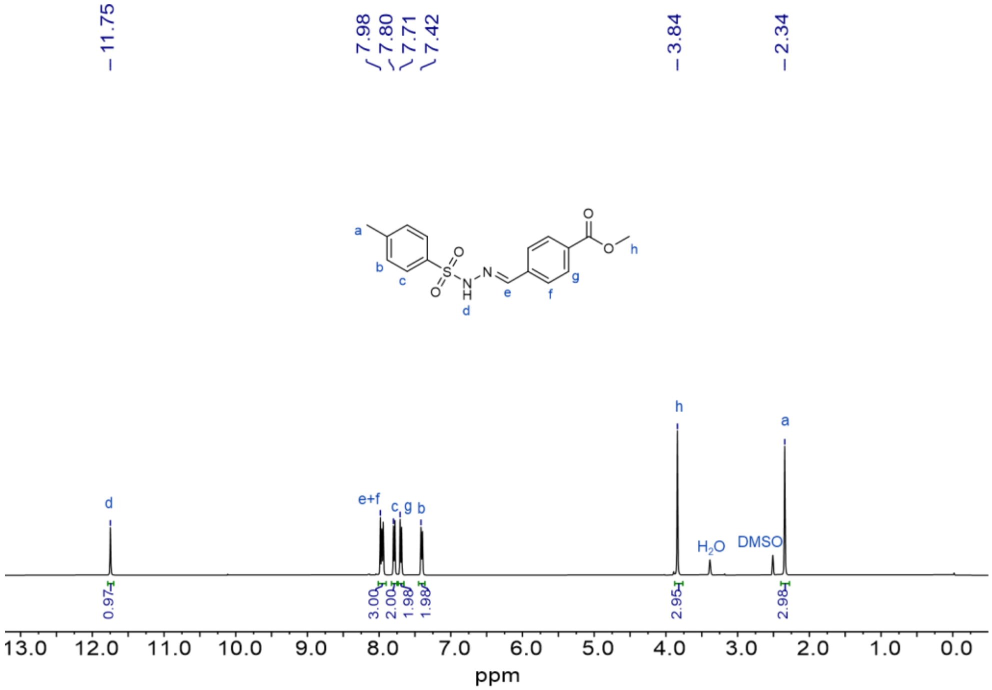

Fig. S33. ^{1}H NMR (400 MHz, DMSO-d_6, 23 °C) spectrum of methyl (*E*)-4-((2-tosylhydrazineylidene)methyl)benzoate.

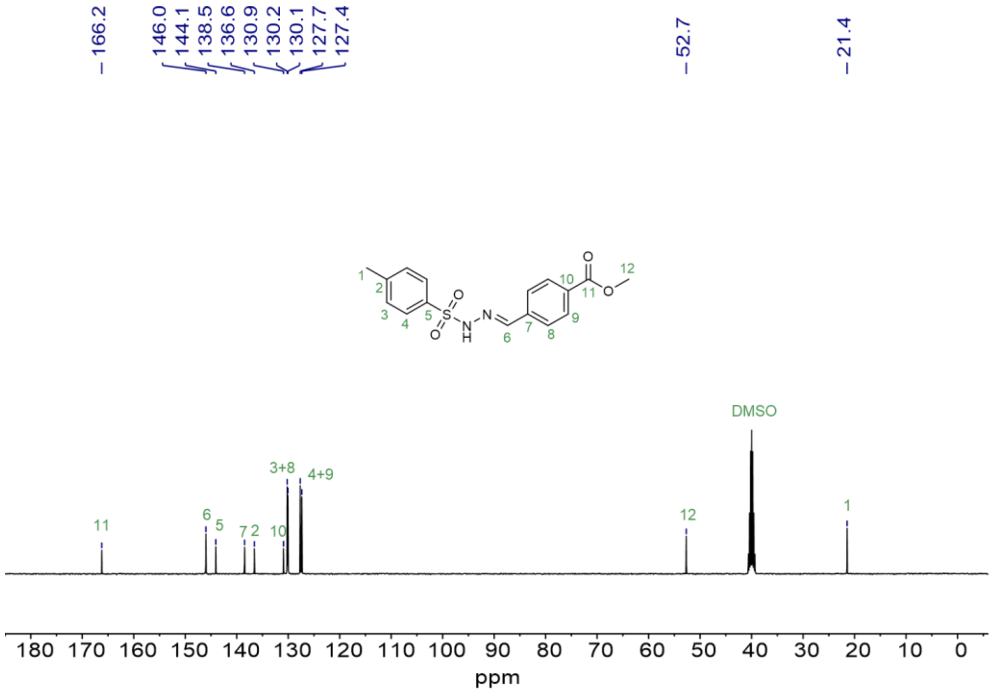

Fig. S34. ^{13}C NMR (100 MHz, DMSO-d_6, 23 °C) spectrum of methyl (*E*)-4-((2-tosylhydrazineylidene)methyl)benzoate.

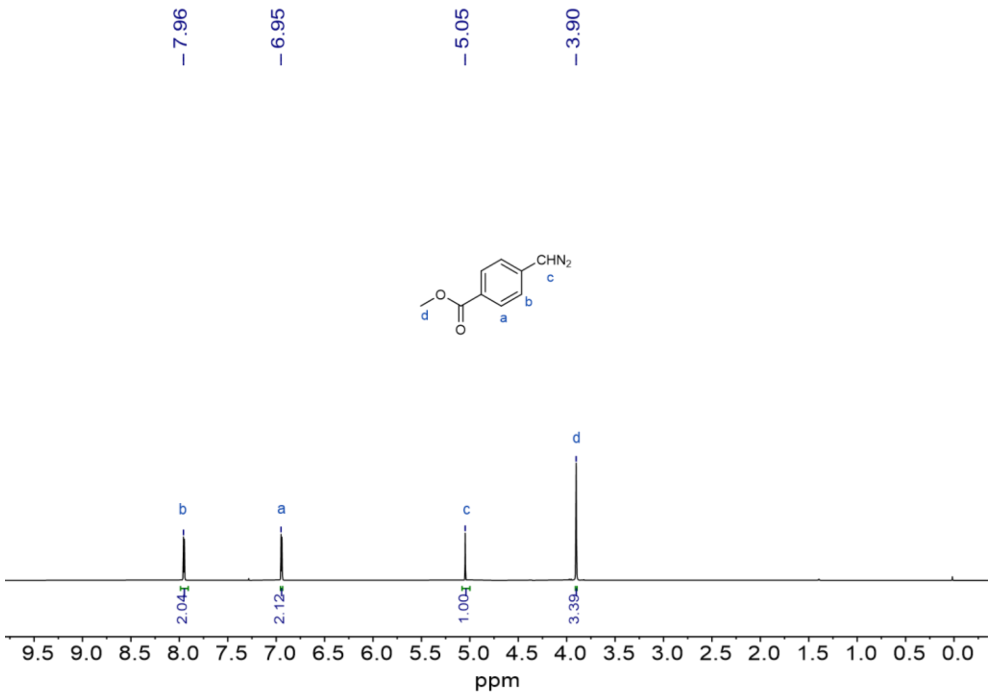

Fig. S35. ^{1}H NMR (400 MHz, $CDCl_3$, 23 ^{o}C) spectrum of methyl 4-(diazomethyl)benzoate (**8**).

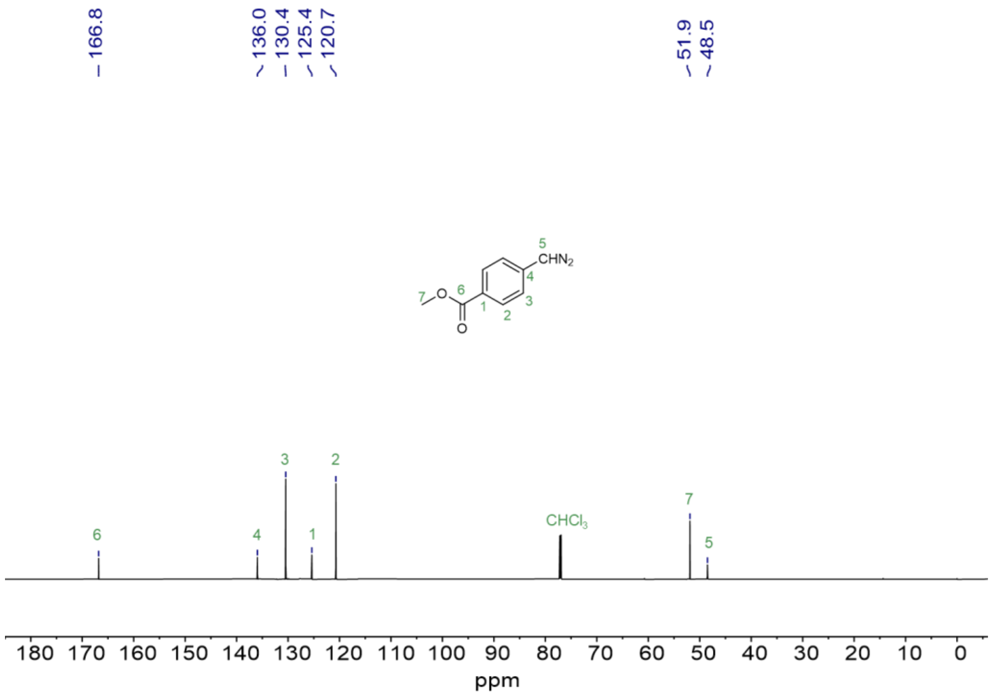

Fig. S36. ^{13}C NMR (100 MHz, $CDCl_3$, 23 ^{o}C) spectrum of methyl 4-(diazomethyl)benzoate (**8**).

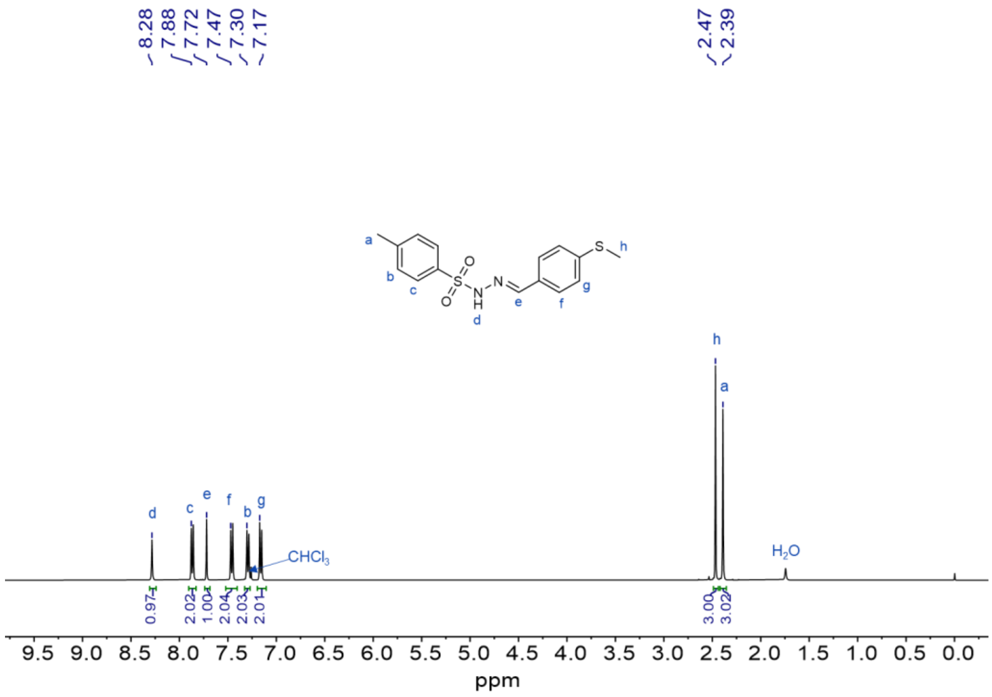

Fig. S37. ^{1}H NMR (400 MHz, $CDCl_3$, 23 °C) spectrum of (*E*)-4-methyl-*N*'-(4-(methylthio)benzylidene)benzenesulfonohydrazide.

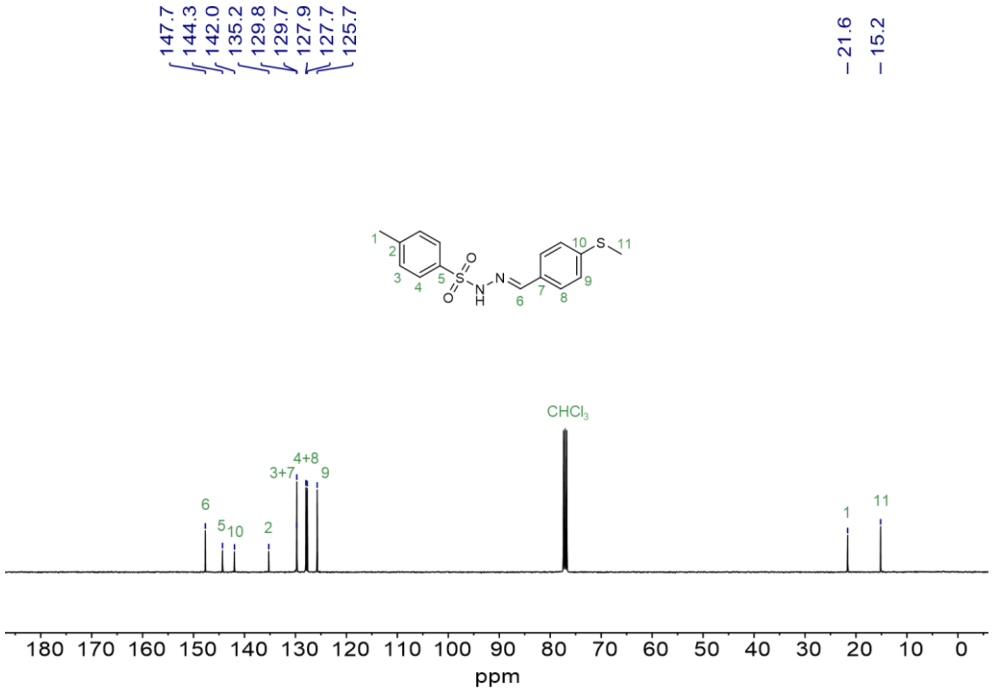

Fig. S38. ^{13}C NMR (100 MHz, $CDCl_3$, 23 °C) spectrum of (*E*)-4-methyl-*N*'-(4-(methylthio)benzylidene)benzenesulfonohydrazide.

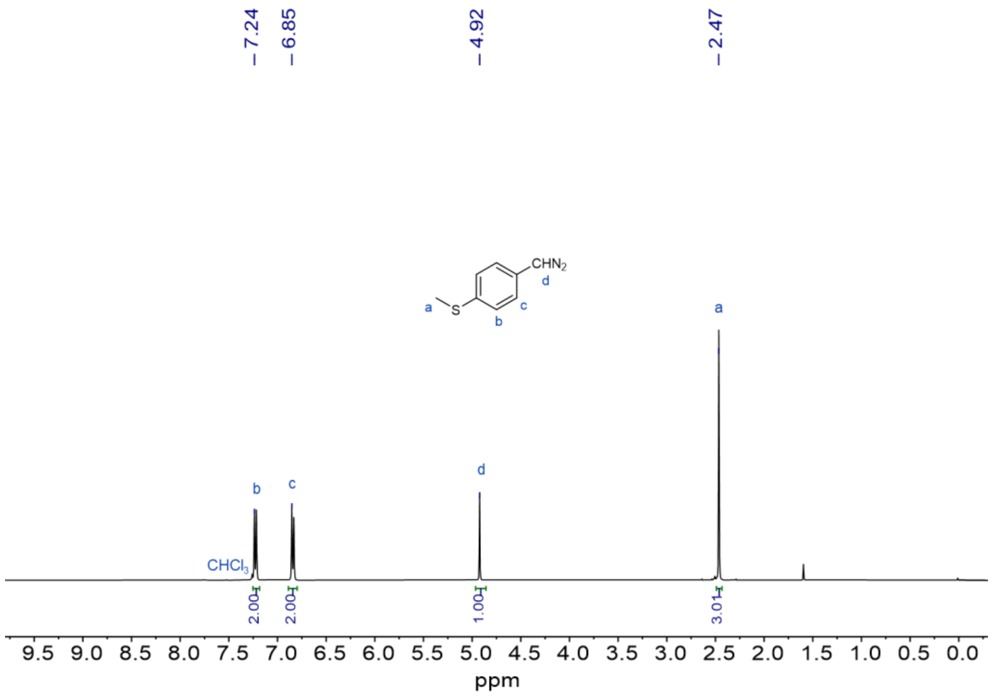

Fig. S39. ^{1}H NMR (400 MHz, $CDCl_3$, 23 °C) spectrum of (4-(diazomethyl)phenyl)(methyl)sulfane (**9**).

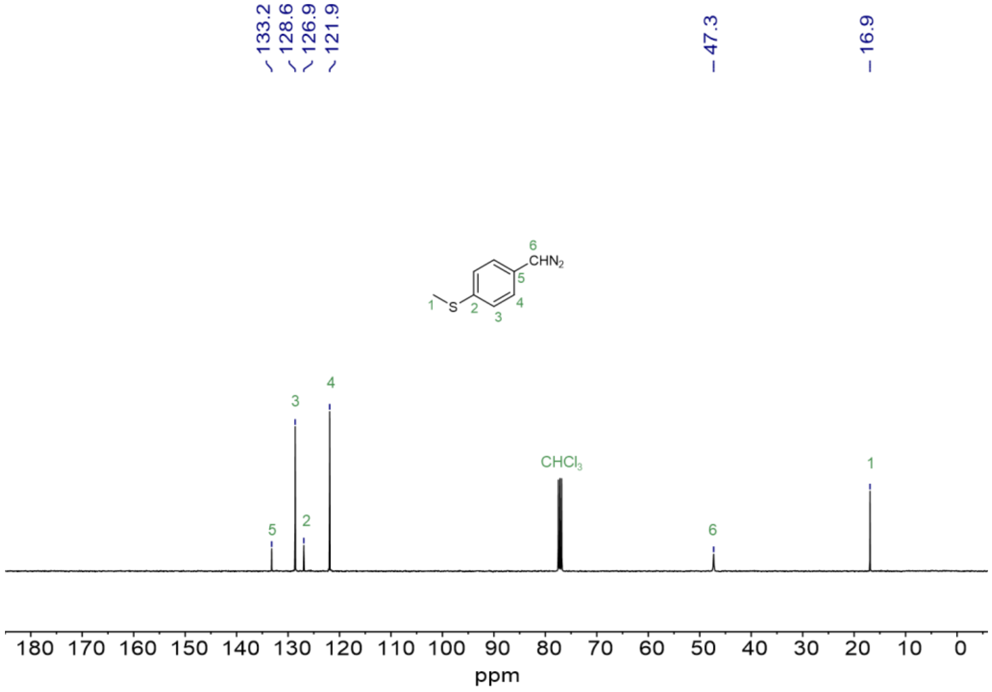

Fig. S40. ^{13}C NMR (100 MHz, $CDCl_3$, 23 °C) spectrum of (4-(diazomethyl)phenyl)(methyl)sulfane (**9**).

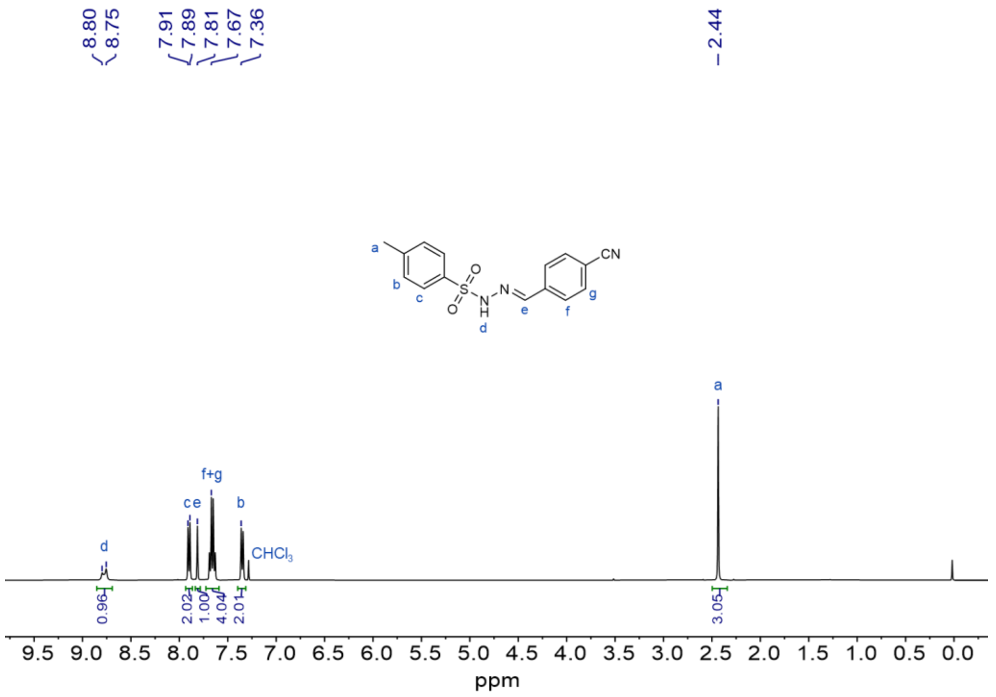

Fig. S41. ^{1}H NMR (400 MHz, $CDCl_3$, 23 ºC) spectrum of (*E*)-*N*'-(4-cyanobenzylidene)-4-methylbenzenesulfonohydrazide.

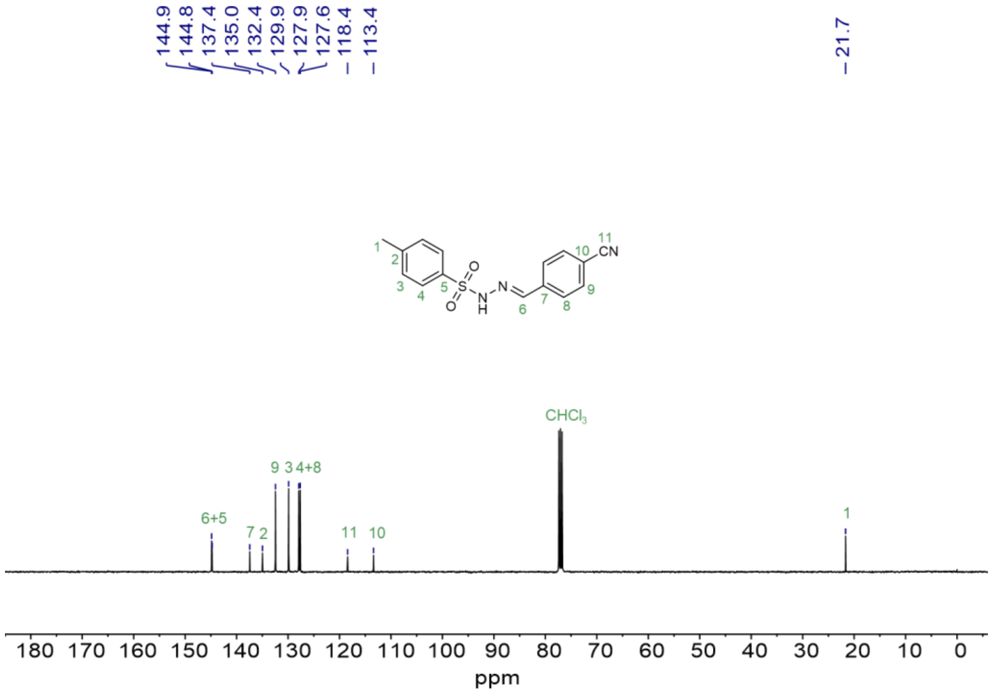

Fig. S42. ^{13}C NMR (100 MHz, $CDCl_3$, 23 ºC) spectrum of (*E*)-*N*'-(4-cyanobenzylidene)-4-methylbenzenesulfonohydrazide.

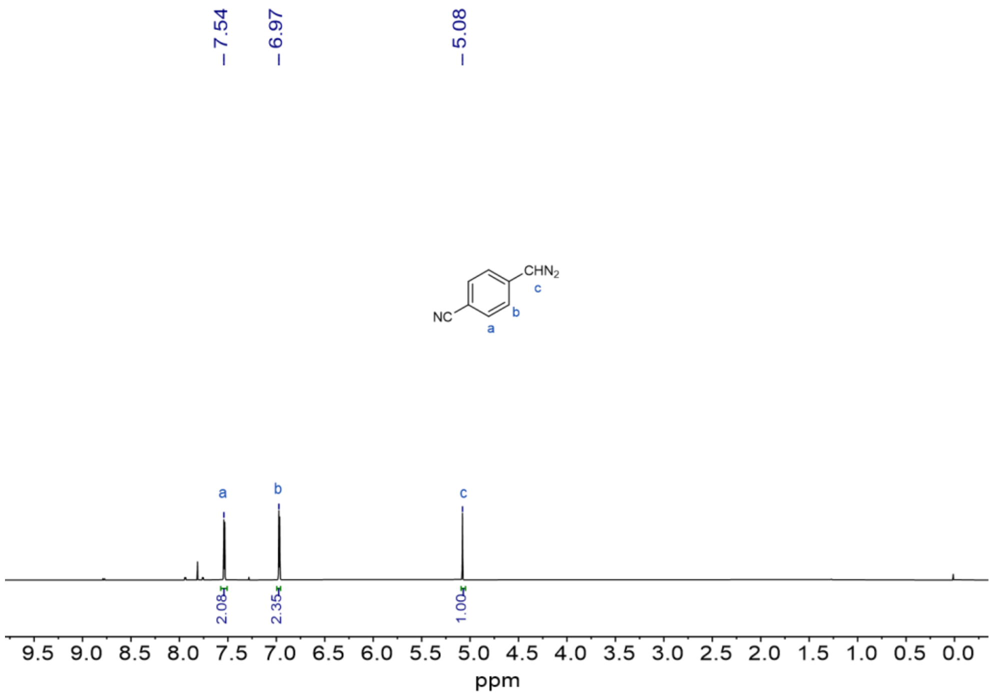

Fig. S43. ^{1}H NMR (400 MHz, $CDCl_3$, 23 ºC) spectrum of 4-(diazomethyl)benzonitrile (**10**).

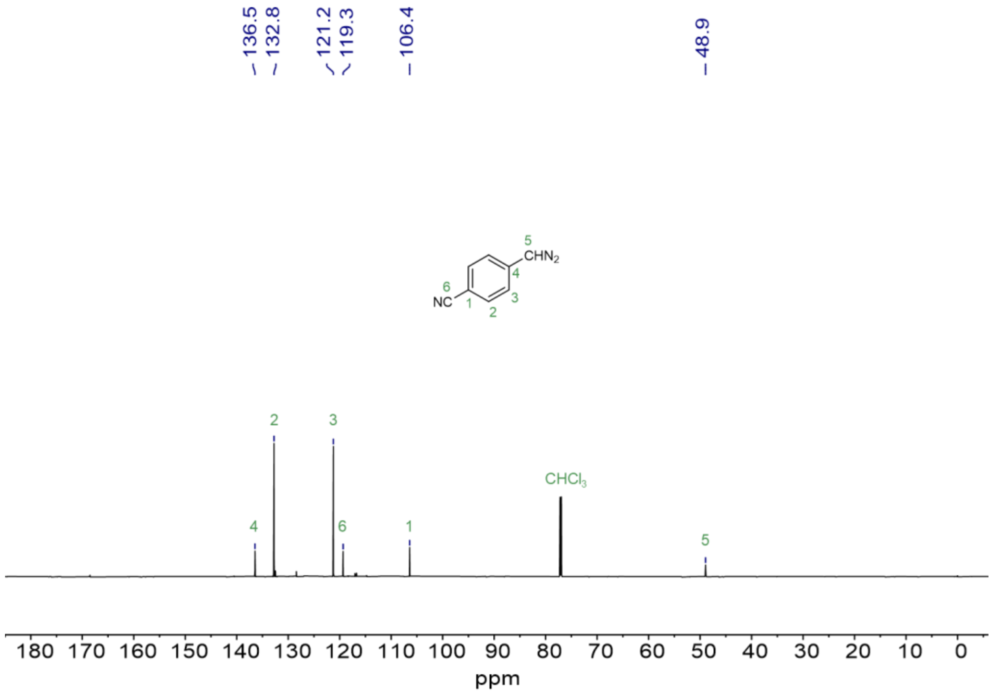

Fig. S44. ^{13}C NMR (100 MHz, $CDCl_3$, 23 ºC) spectrum of 4-(diazomethyl)benzonitrile (**10**).

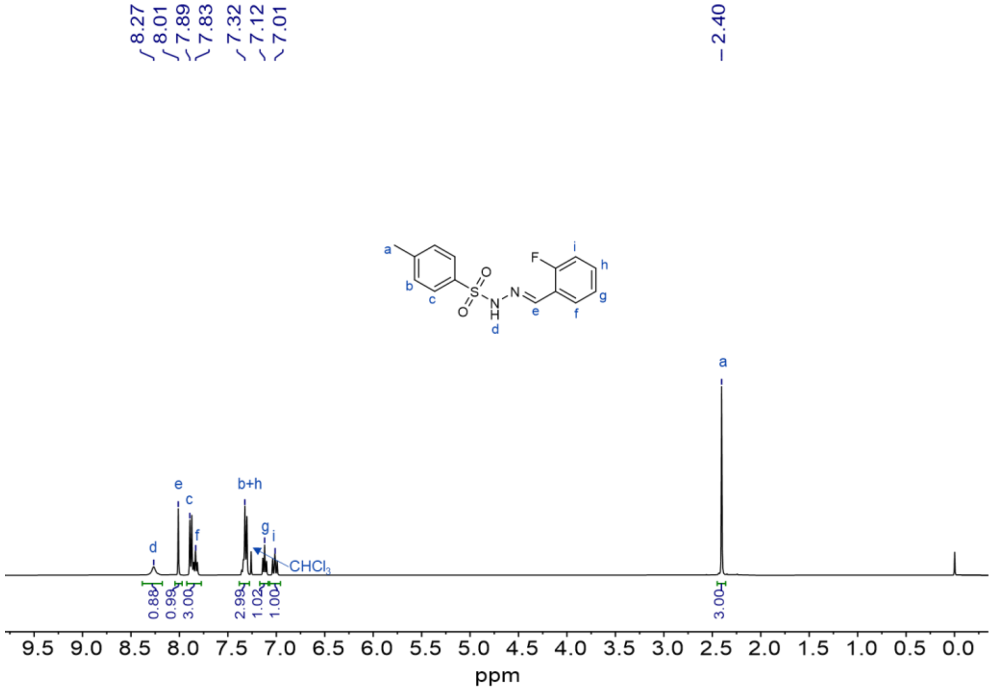

Fig. S45. ^{1}H NMR (400 MHz, $CDCl_3$, 23 ºC) spectrum of (*E*)-*N*'-(2-fluorobenzylidene)-4-methylbenzenesulfonohydrazide.

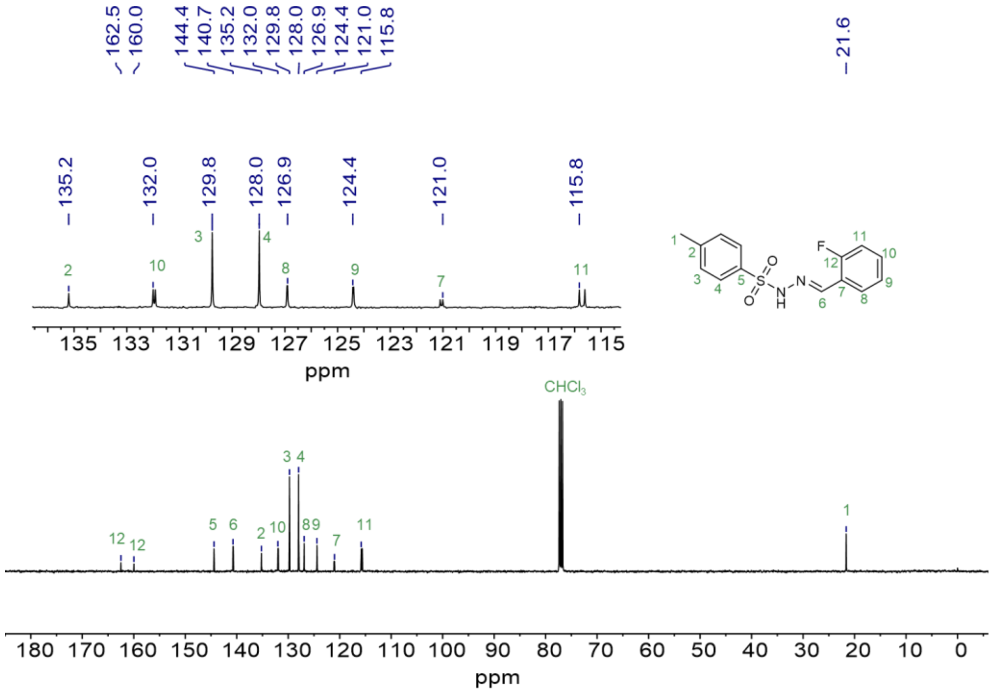

Fig. S46. ^{13}C NMR (100 MHz, $CDCl_3$, 23 ºC) spectrum of (*E*)-*N*'-(2-fluorobenzylidene)-4-methylbenzenesulfonohydrazide (C11 and C12 exhibit splitting due to ^{13}C-^{19}F coupling).

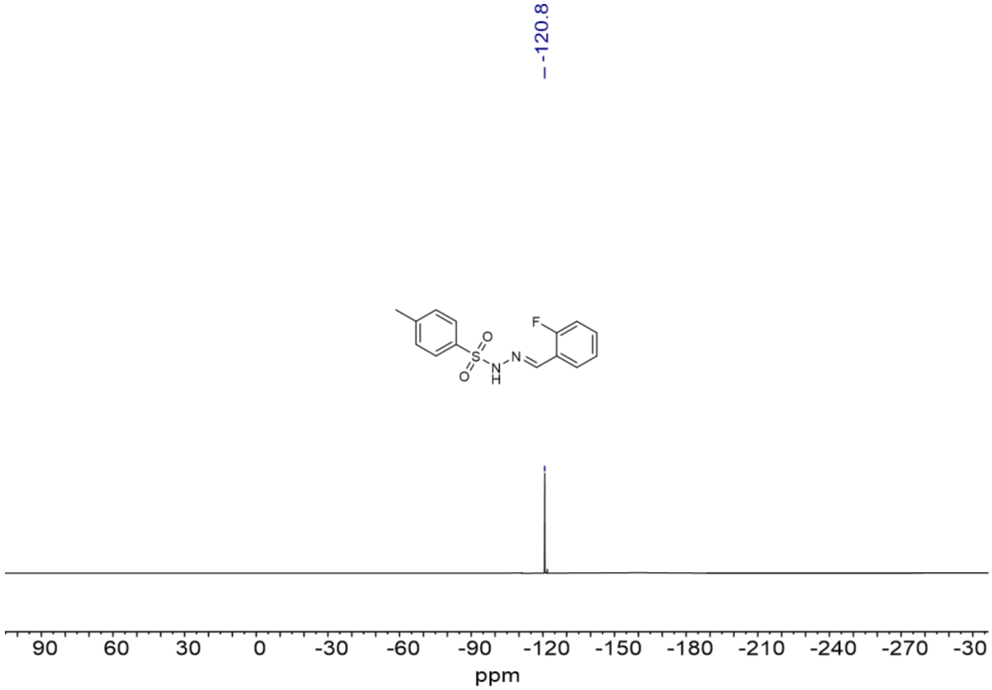

Fig. S47. ^{19}F NMR (377 MHz, $CDCl_3$, 23 °C) spectrum of (*E*)-*N'*-(2-fluorobenzylidene)-4-methylbenzenesulfonohydrazide.

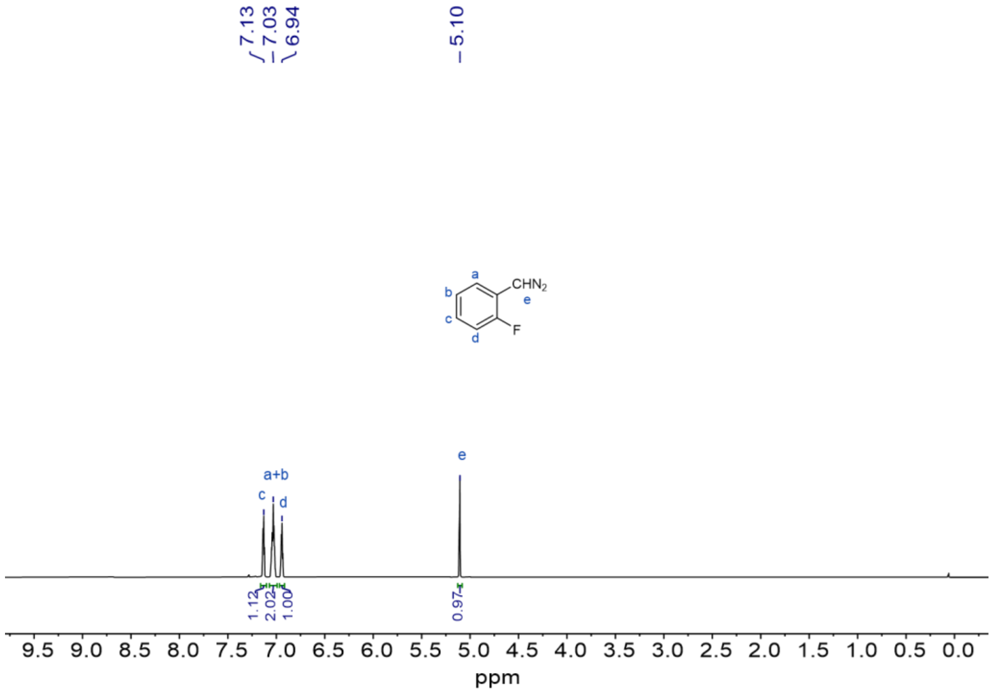

Fig. S48. ^{1}H NMR (400 MHz, $CDCl_3$, 23 °C) spectrum of 1-(diazomethyl)-2-fluorobenzene (**11**).

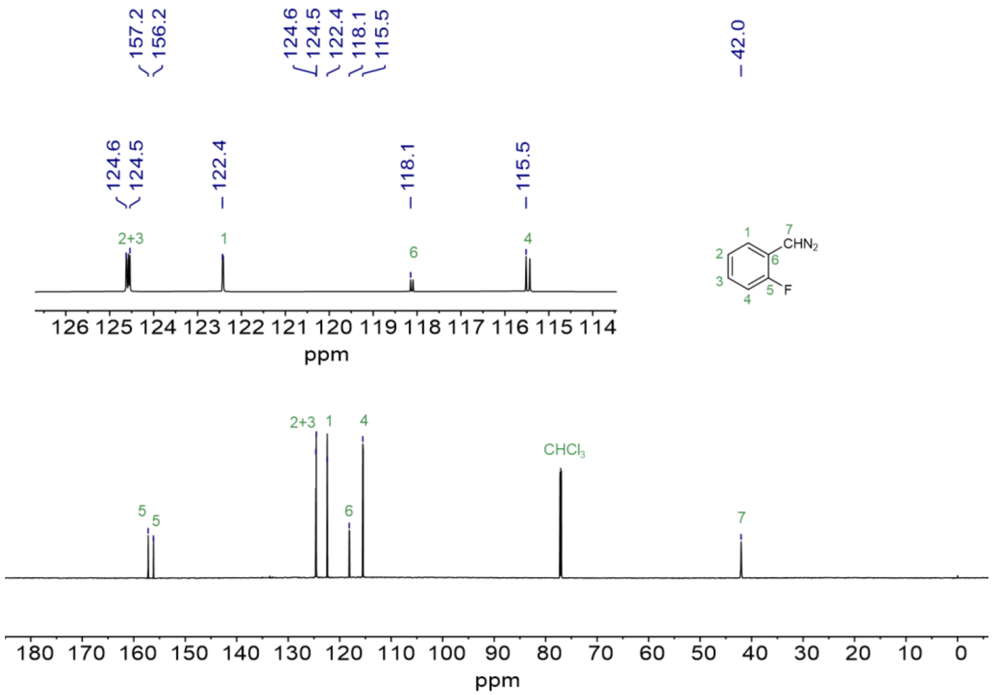

Fig. S49. ^{13}C NMR (100 MHz, $CDCl_3$, 23 °C) spectrum of 1-(diazomethyl)-2-fluorobenzene (**11**) (C4, C5, and C6 exhibit splitting due to ^{13}C-^{19}F coupling).

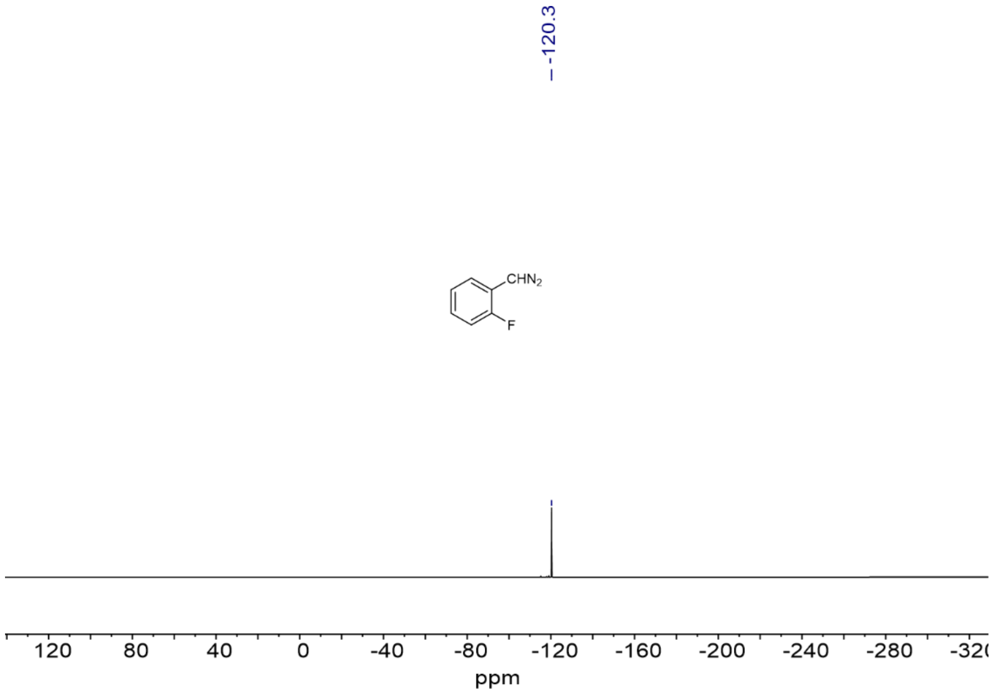

Fig. S50. ^{19}F NMR (377 MHz, $CDCl_3$, 23 °C) spectrum of 1-(diazomethyl)-2-fluorobenzene (**11**).

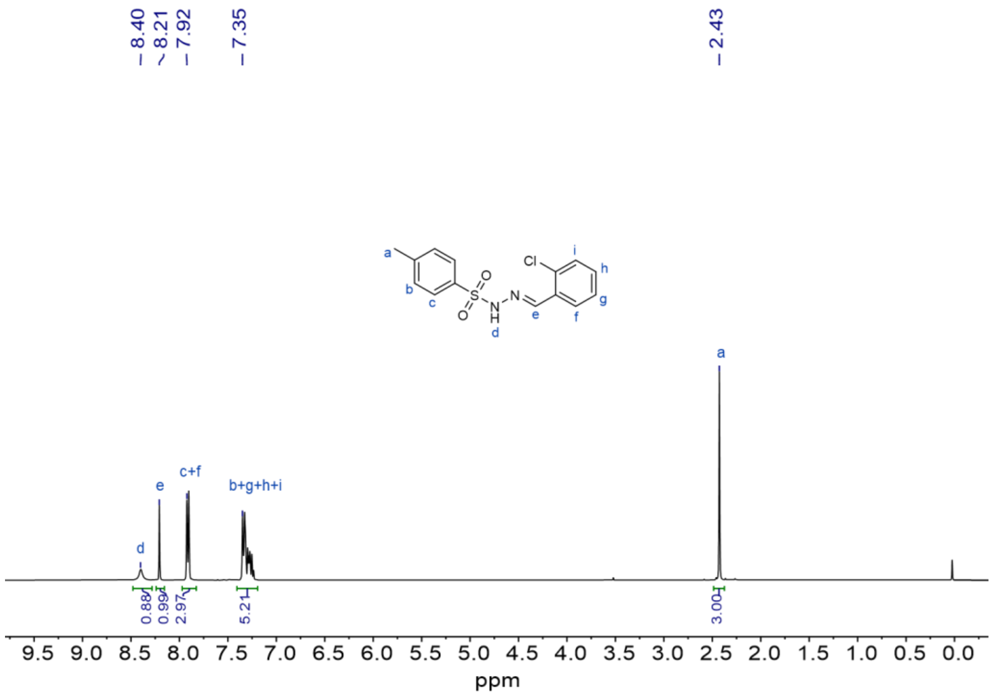

Fig. S51. ^{1}H NMR (400 MHz, $CDCl_3$, 23 ºC) spectrum of (*E*)-*N*'-(2-chlorobenzylidene)-4-methylbenzenesulfonohydrazide.

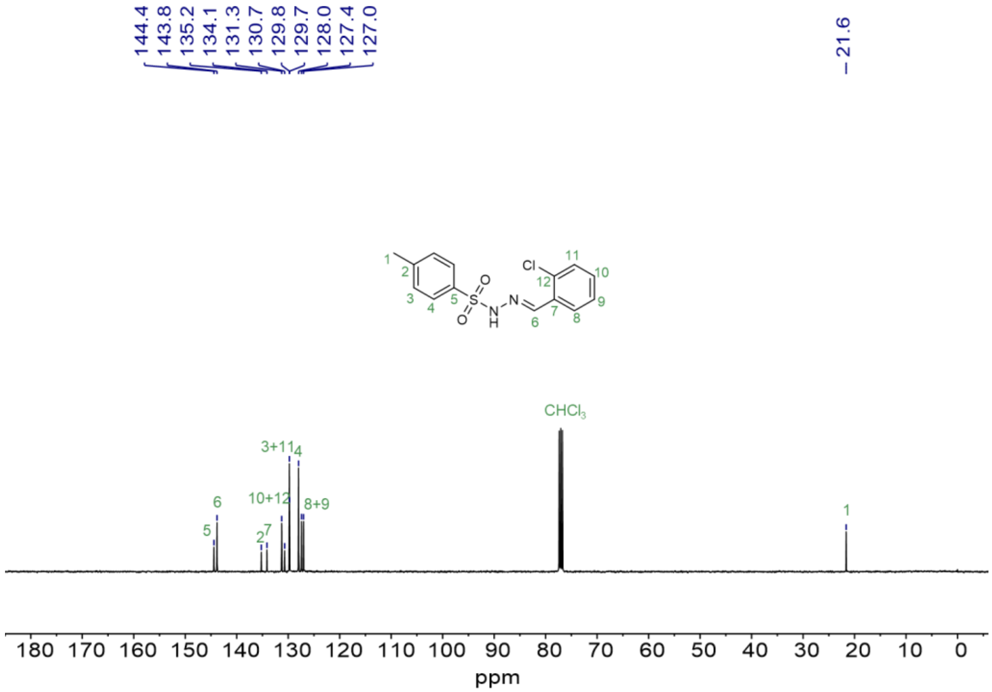

Fig. S52. ^{13}C NMR (100 MHz, $CDCl_3$, 23 ºC) spectrum of (*E*)-*N*'-(2-chlorobenzylidene)-4-methylbenzenesulfonohydrazide.

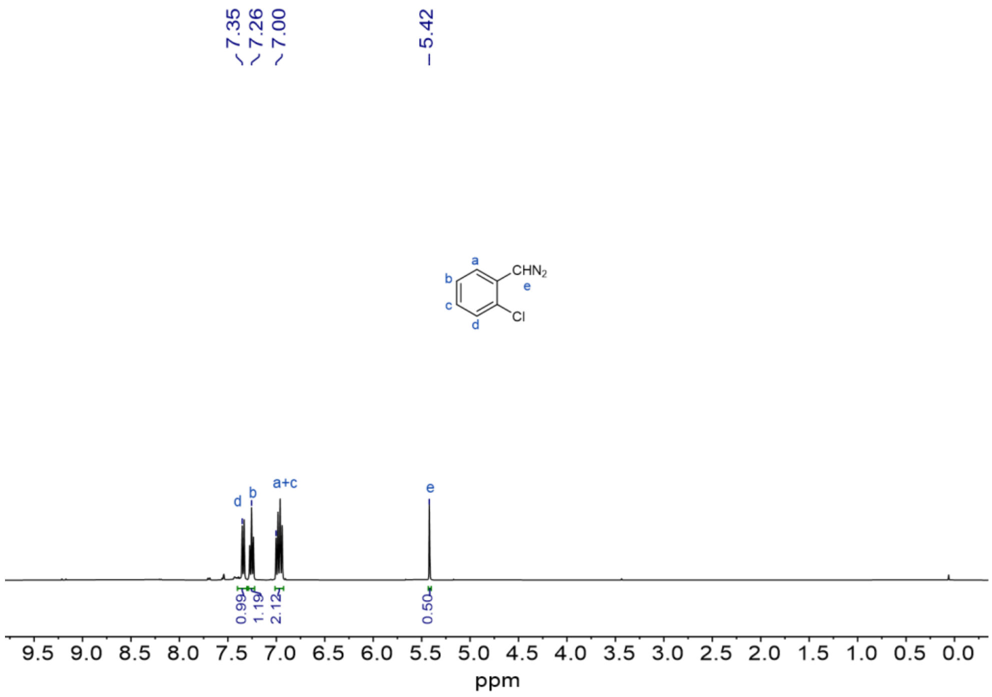

Fig. S53. ^{1}H NMR (400 MHz, $CDCl_3$, 23 °C) spectrum of 1-chloro-2-(diazomethyl)benzene (**12**).

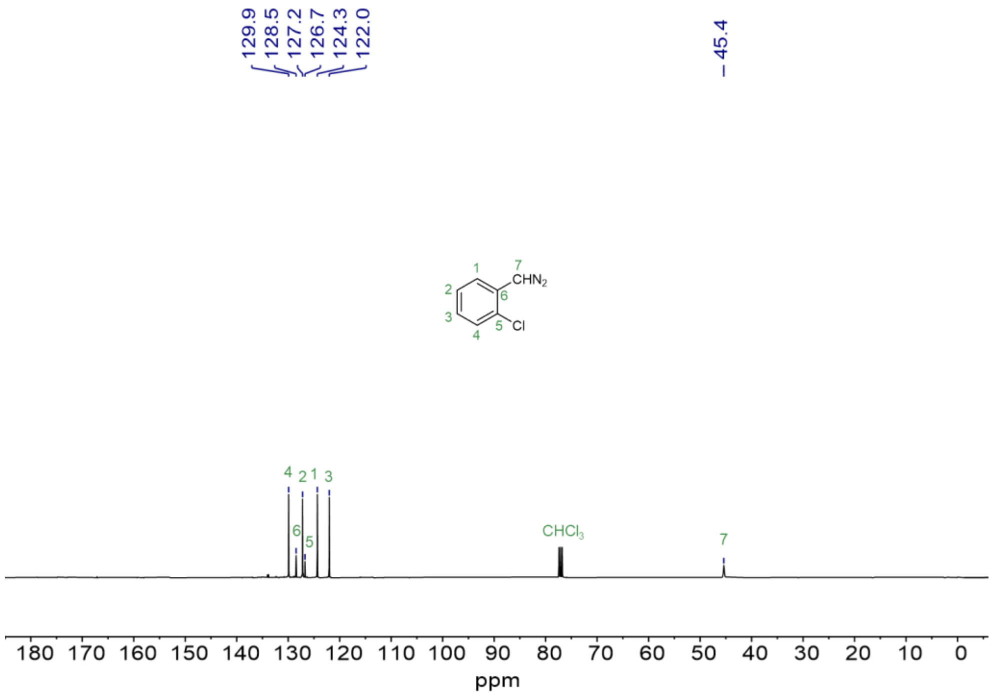

Fig. S54. ^{13}C NMR (100 MHz, $CDCl_3$, 23 °C) spectrum of 1-chloro-2-(diazomethyl)benzene (**12**).

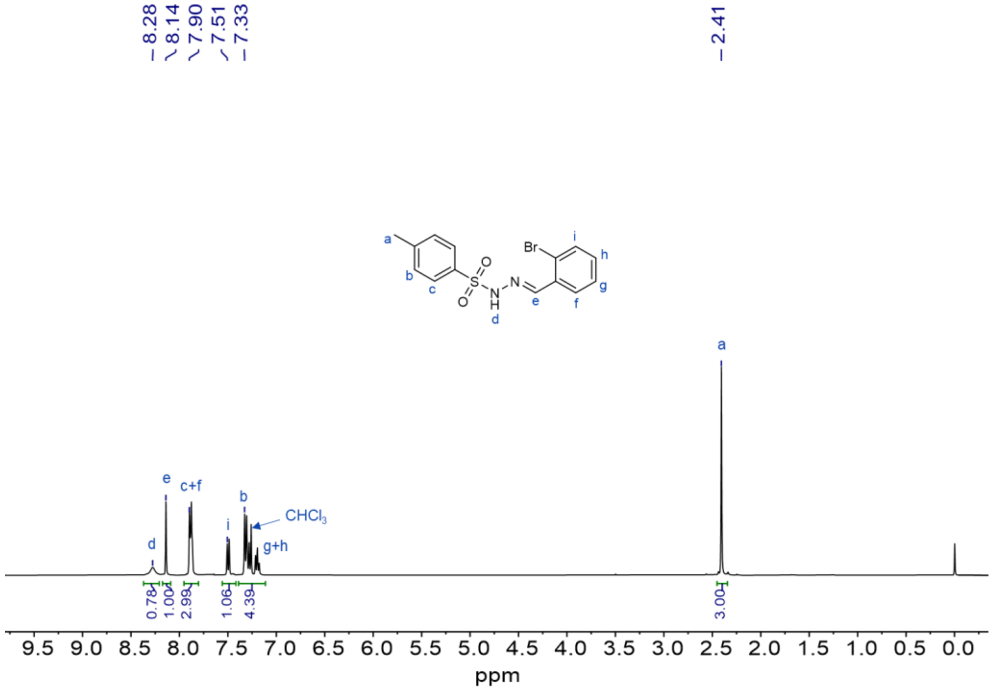

Fig. S55. ^{1}H NMR (400 MHz, $CDCl_3$, 23 ºC) spectrum of (*E*)-*N'*-(2-bromobenzylidene)-4-methylbenzenesulfonohydrazide.

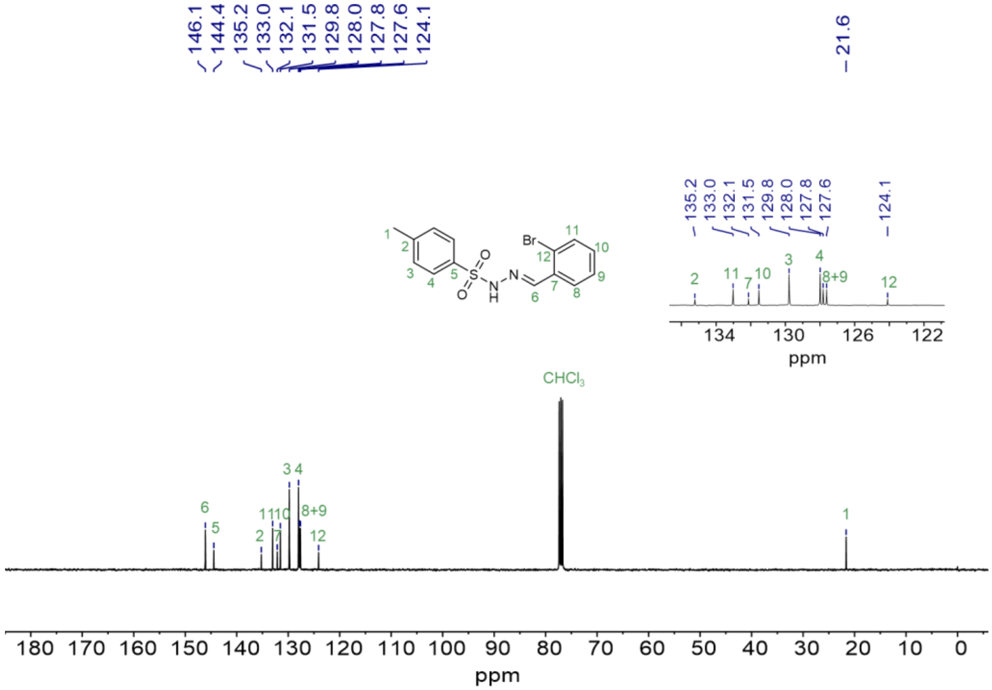

Fig. S56. ^{13}C NMR (100 MHz, $CDCl_3$, 23 ºC) spectrum of (*E*)-*N'*-(2-bromobenzylidene)-4-methylbenzenesulfonohydrazide.

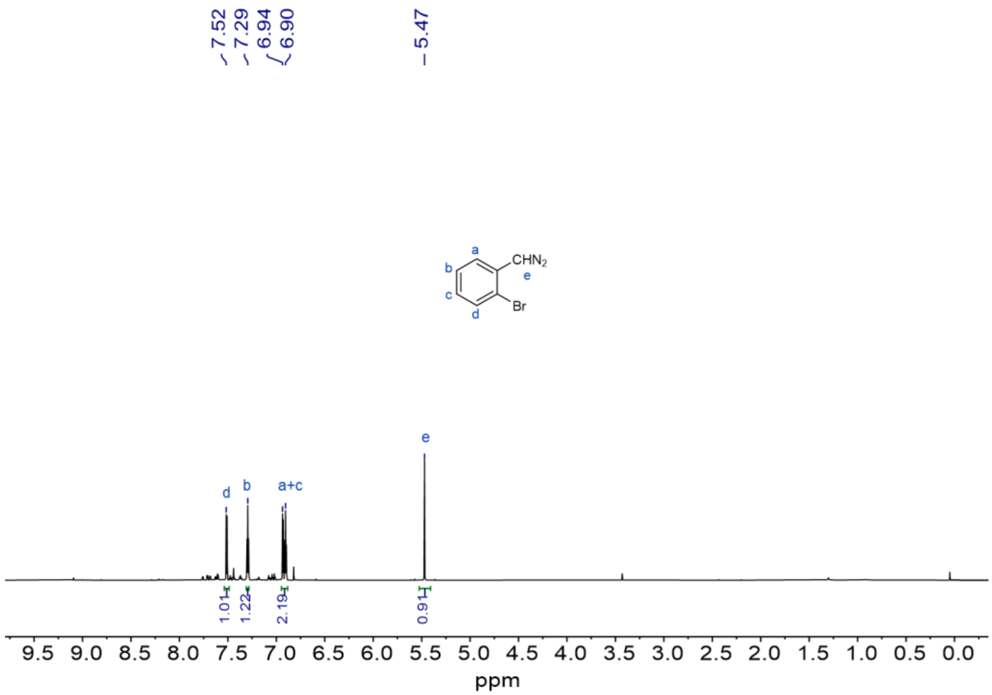

Fig. S57. ^{1}H NMR (400 MHz, $CDCl_3$, 23 ^{o}C) spectrum of 1-bromo-2-(diazomethyl)benzene (**13**).

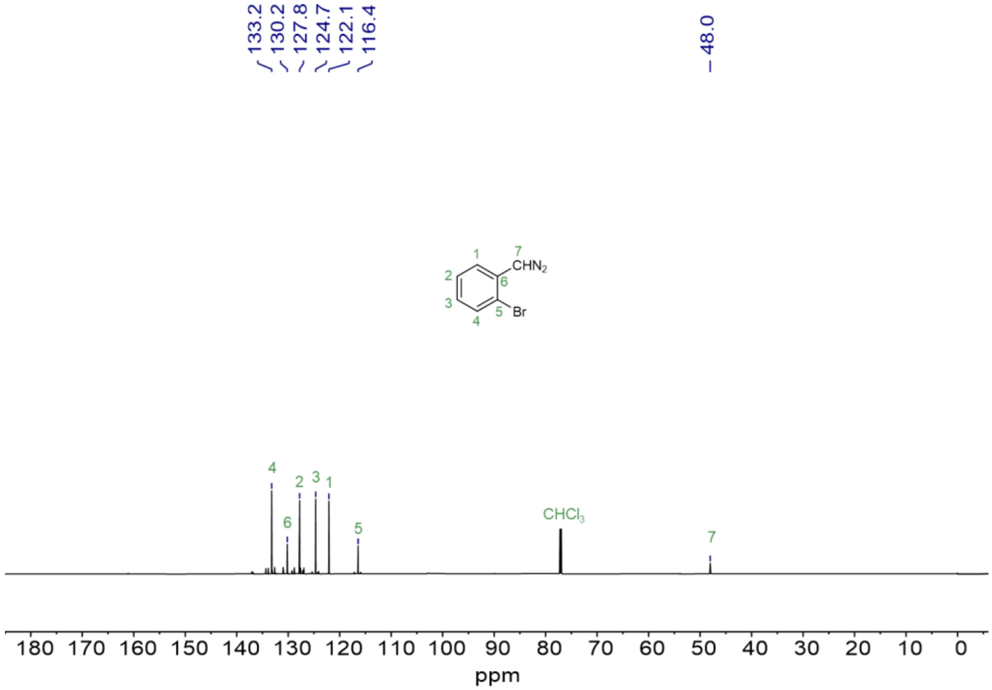

Fig. S58. ^{13}C NMR (100 MHz, $CDCl_3$, 23 ^{o}C) spectrum of 1-bromo-2-(diazomethyl)benzene (**13**).

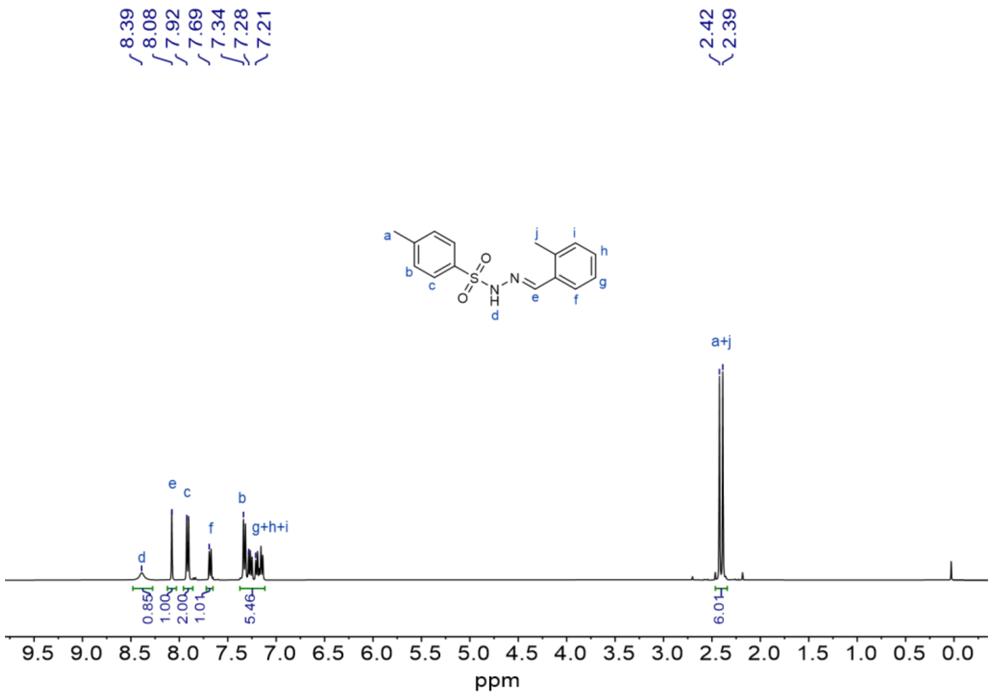

Fig. S59. ^{1}H NMR (400 MHz, $CDCl_3$, 23 °C) spectrum of (*E*)-4-methyl-*N*'-(2-methylbenzylidene)benzenesulfonohydrazide.

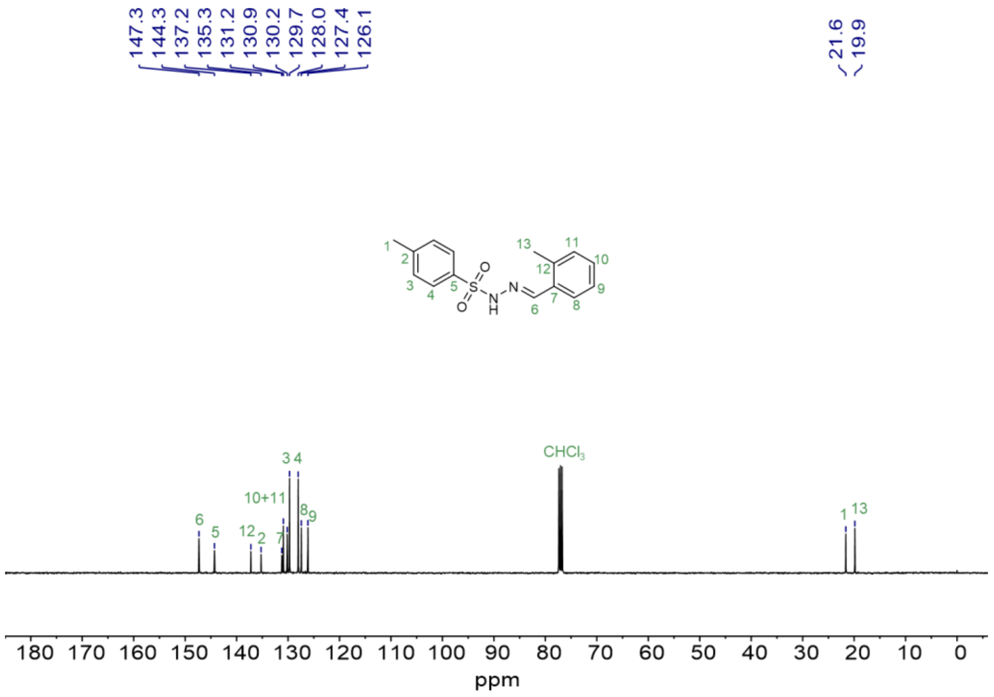

Fig. S60. ^{13}C NMR (100 MHz, $CDCl_3$, 23 °C) spectrum of (*E*)-4-methyl-*N*'-(2-methylbenzylidene)benzenesulfonohydrazide.

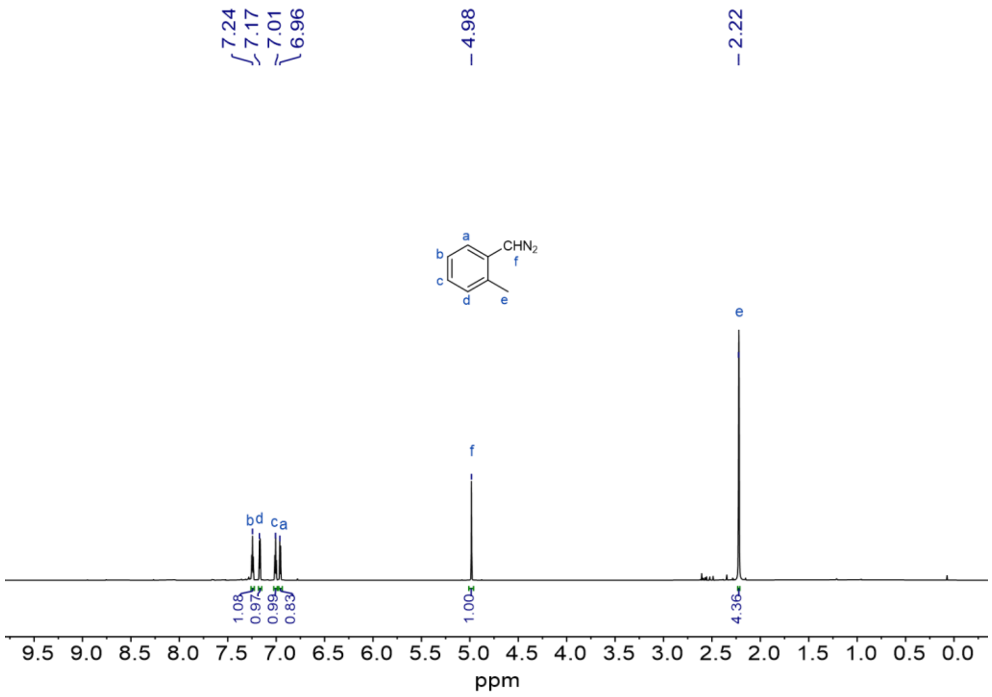

Fig. S61. ^{1}H NMR (400 MHz, $CDCl_3$, 23 °C) spectrum of 1-(diazomethyl)-2-methylbenzene (**14**).

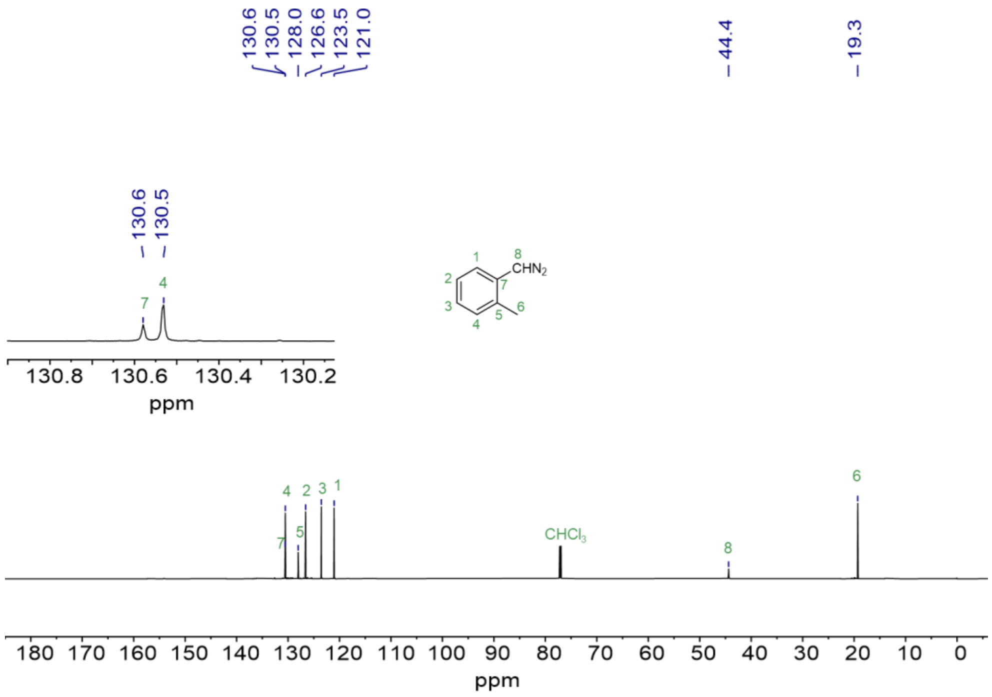

Fig. S62. ^{13}C NMR (100 MHz, $CDCl_3$, 23 °C) spectrum of 1-(diazomethyl)-2-methylbenzene (**14**).

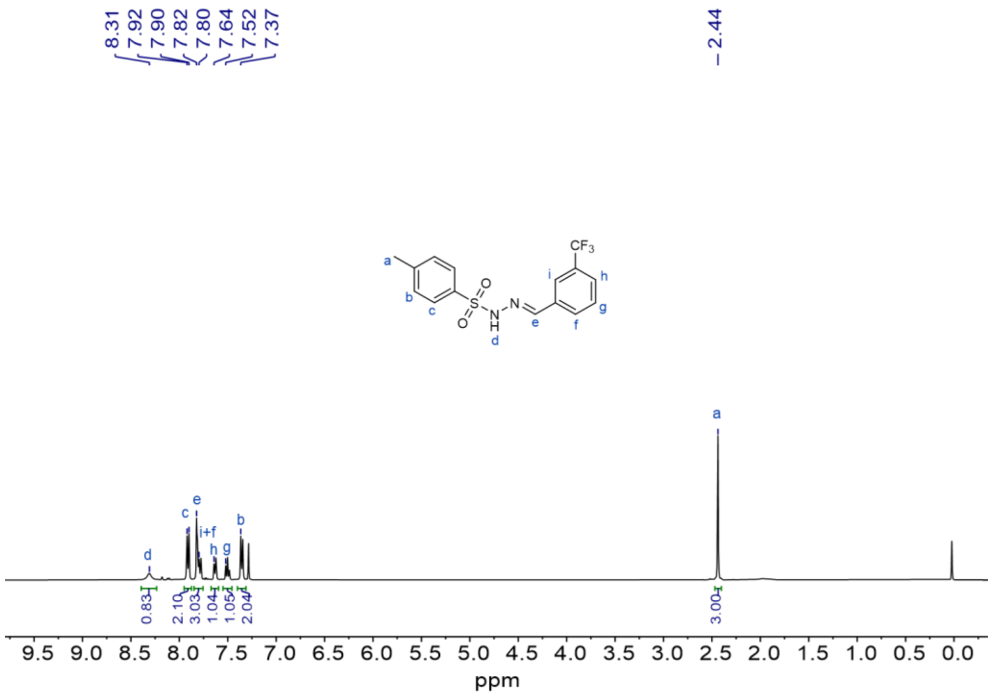

Fig. S63. ^{1}H NMR (400 MHz, $CDCl_3$, 23 °C) spectrum of (*E*)-4-methyl-*N*'-(3-(trifluoromethyl)benzylidene)benzenesulfonohydrazide.

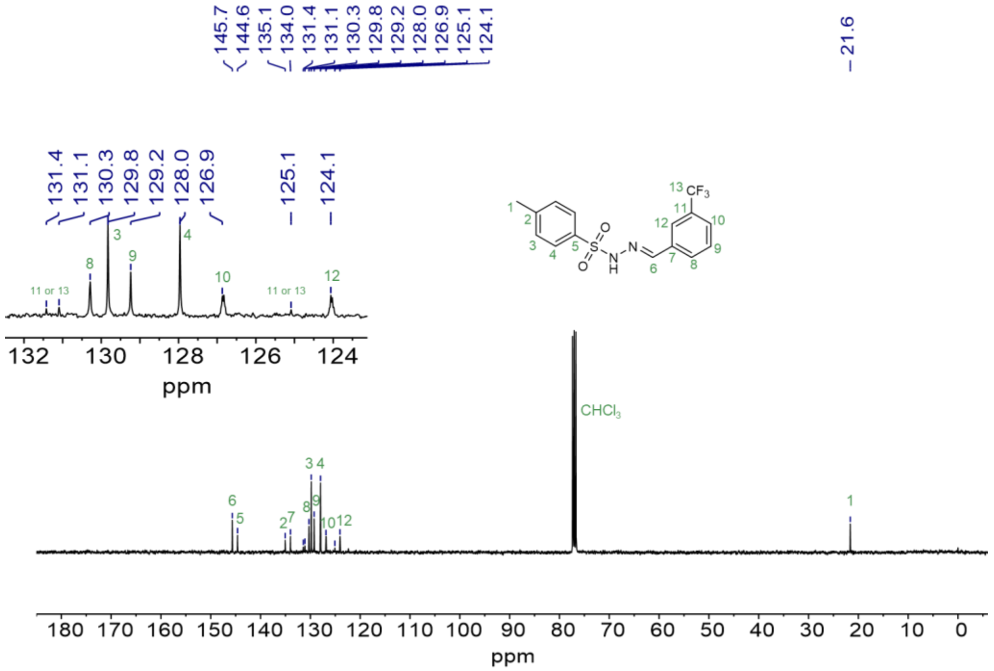

Fig. S64. ^{13}C NMR (100 MHz, $CDCl_3$, 23 °C) spectrum of (*E*)-4-methyl-*N*'-(3-(trifluoromethyl)benzylidene)benzenesulfonohydrazide (C11 and C13 show strong ^{13}C–^{19}F coupling with the fluorine atom, resulting in complex splitting that hinders the resolution and assignment of individual carbon signals. C10 and C12 are disturbed by the fluorine atom and split).

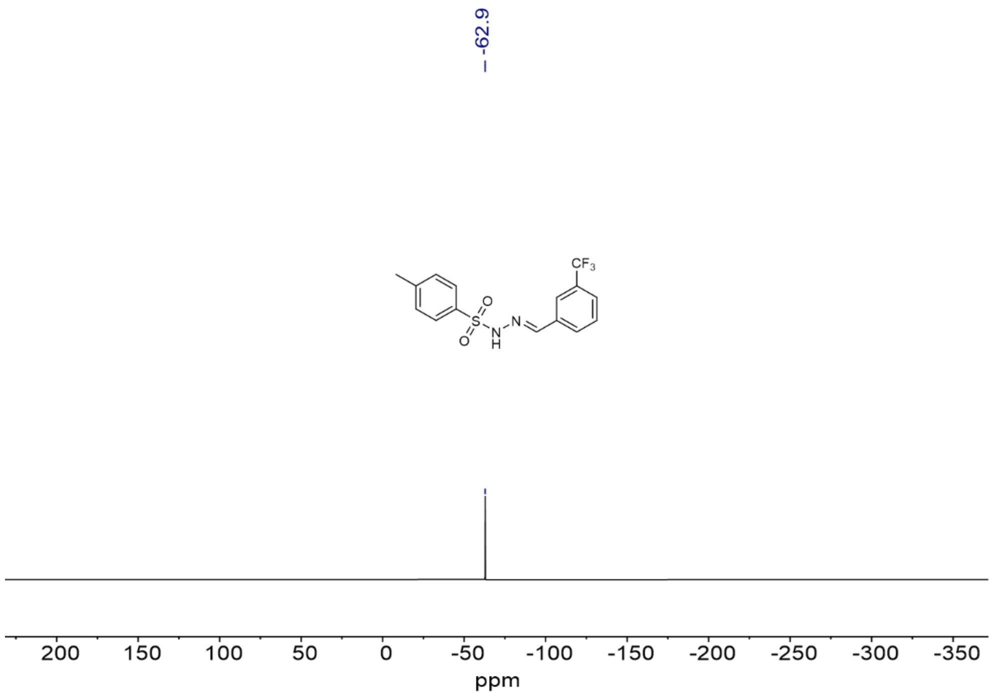

Fig. S65. ^{19}F NMR (377 MHz, $CDCl_3$, 23 °C) spectrum of (*E*)-4-methyl-*N'*-(3-(trifluoromethyl)benzylidene)benzenesulfonohydrazide.

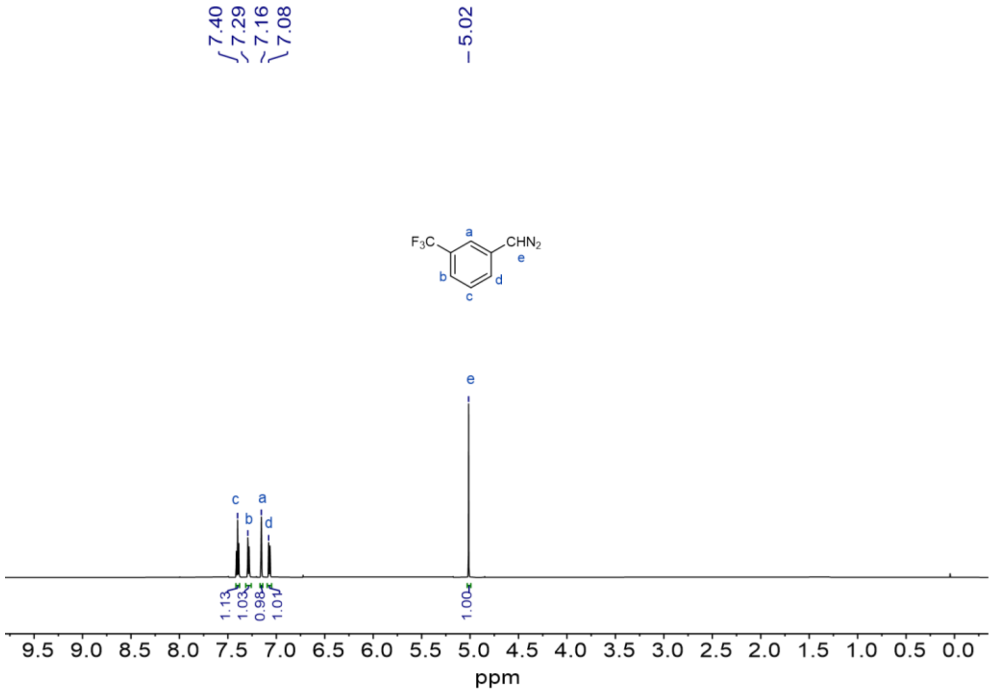

Fig. S66. ^{1}H NMR (400 MHz, $CDCl_3$, 23 °C) spectrum of 1-(diazomethyl)-3-(trifluoromethyl)benzene (**15**).

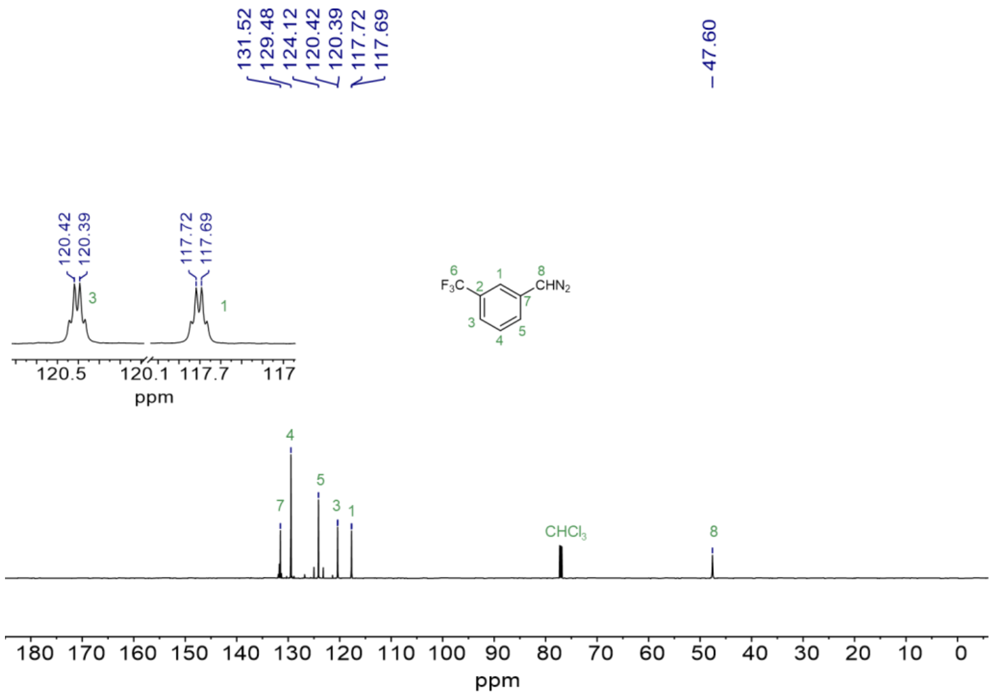

Fig. S67. ^{13}C NMR (100 MHz, $CDCl_3$, 23 ºC) spectrum of 1-(diazomethyl)-3-(trifluoromethyl)benzene (**15**). (C2 and C6 show strong ^{13}C–^{19}F coupling with the fluorine atom, resulting in complex splitting that hinders the resolution and assignment of individual carbon signals. C1 and C3 are disturbed by the fluorine atom and split).

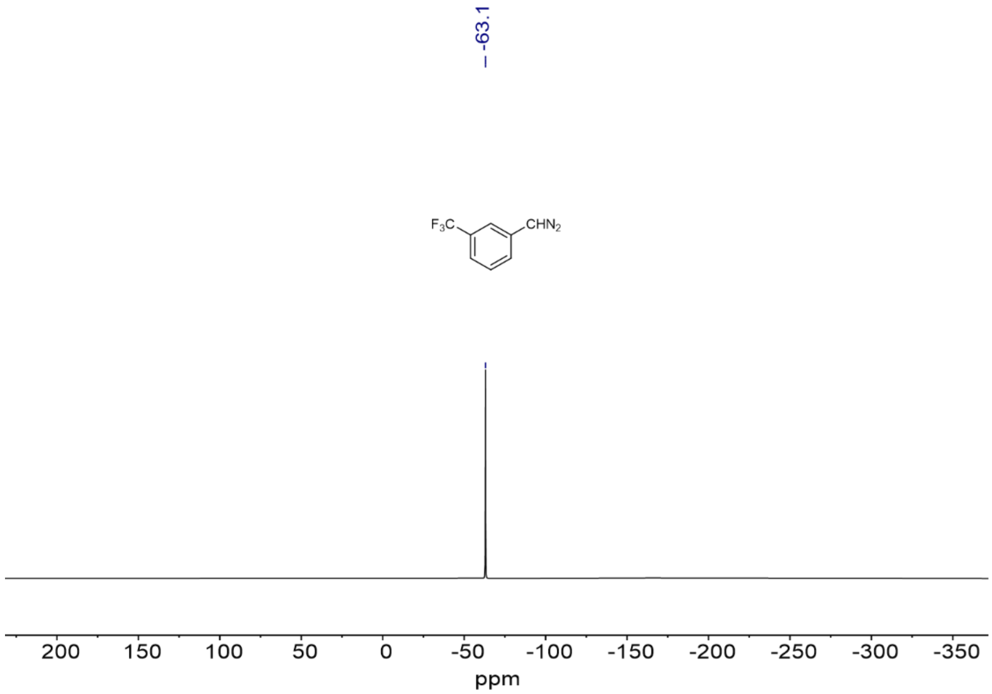

Fig. S68. ^{19}F NMR (377 MHz, $CDCl_3$, 23 ºC) spectrum of 1-(diazomethyl)-3-(trifluoromethyl)benzene (**15**).

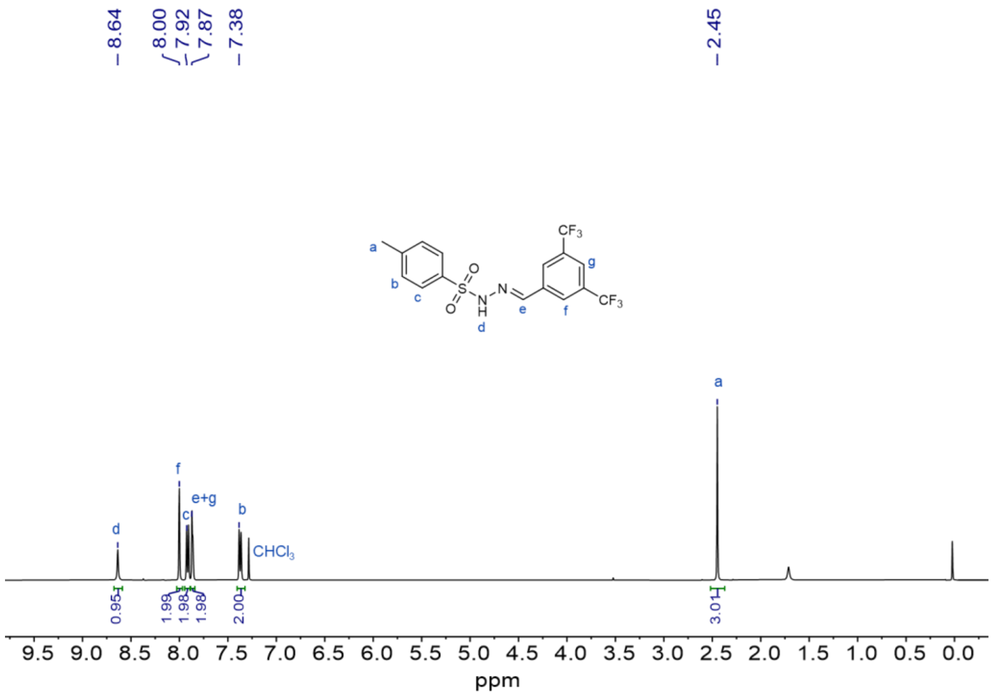

Fig. S69. ^{1}H NMR (400 MHz, $CDCl_3$, 23 ºC) spectrum of (*E*)-*N*'-(3,5-bis(trifluoromethyl)benzylidene)-4-methylbenzenesulfonohydrazide.

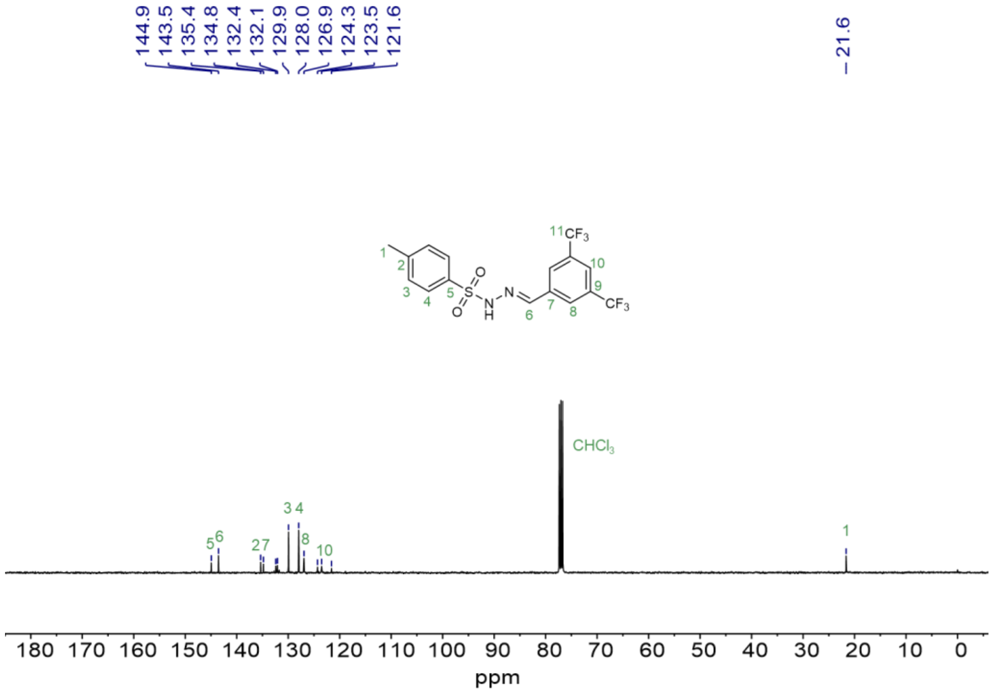

Fig. S70. ^{13}C NMR (100 MHz, $CDCl_3$, 23 ºC) spectrum of (*E*)-*N*'-(3,5-bis(trifluoromethyl)benzylidene)-4-methylbenzenesulfonohydrazide. (C9 and C11 show strong ^{13}C–^{19}F coupling with the fluorine atom, resulting in complex splitting that hinders the resolution and assignment of individual carbon signals. C8 and C10 are disturbed by the fluorine atom and split).

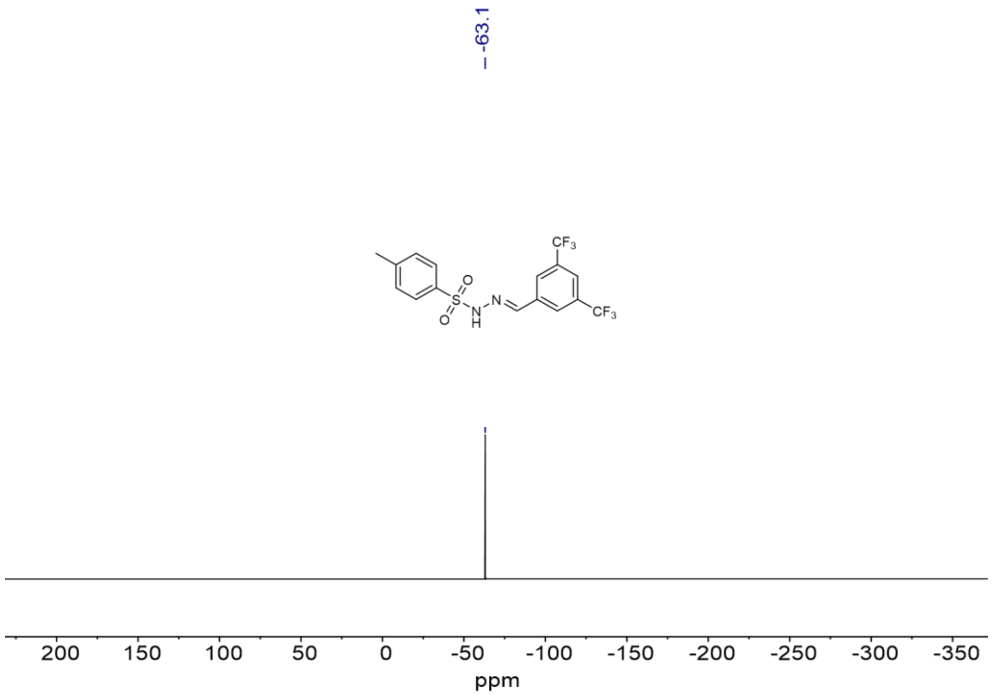

Fig. S71. ^{19}F NMR (377 MHz, $CDCl_3$, 23 ºC) spectrum of (*E*)-*N*'-(3,5-bis(trifluoromethyl)benzylidene)-4-methylbenzenesulfonohydrazide.

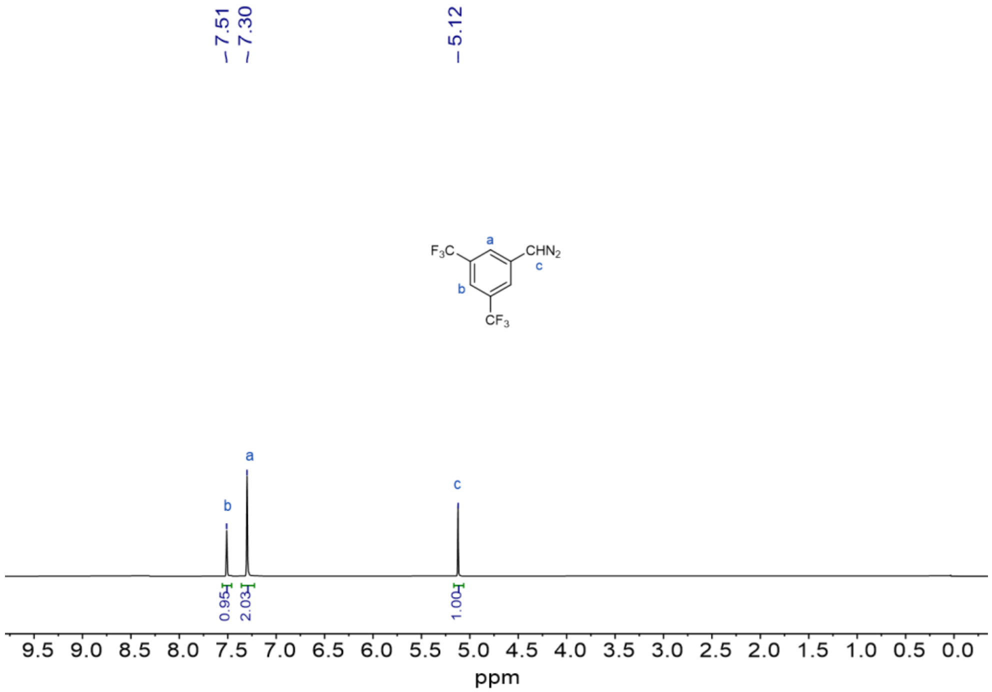

Fig. S72. ^{1}H NMR (400 MHz, $CDCl_3$, 23 ºC) spectrum of 1-(diazomethyl)-3,5-bis(trifluoromethyl)benzene (**16**).

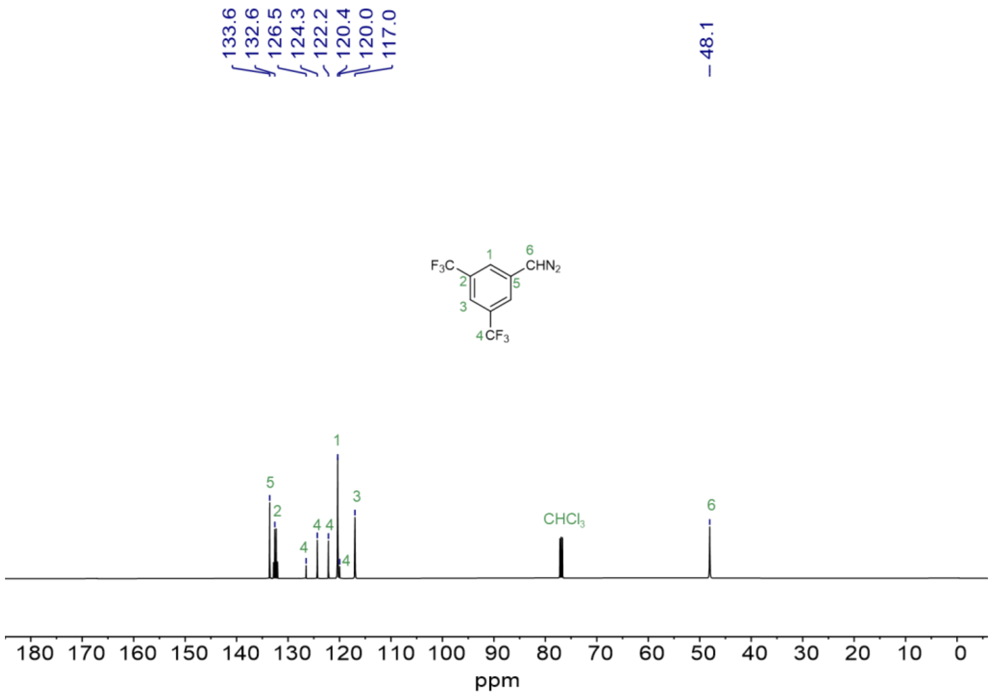

Fig. S73. ^{13}C NMR (100 MHz, $CDCl_3$, 23 °C) spectrum of 1-(diazomethyl)-3,5-bis(trifluoromethyl)benzene (**16**). (C2 and C4 are disturbed by the fluorine atom and split).

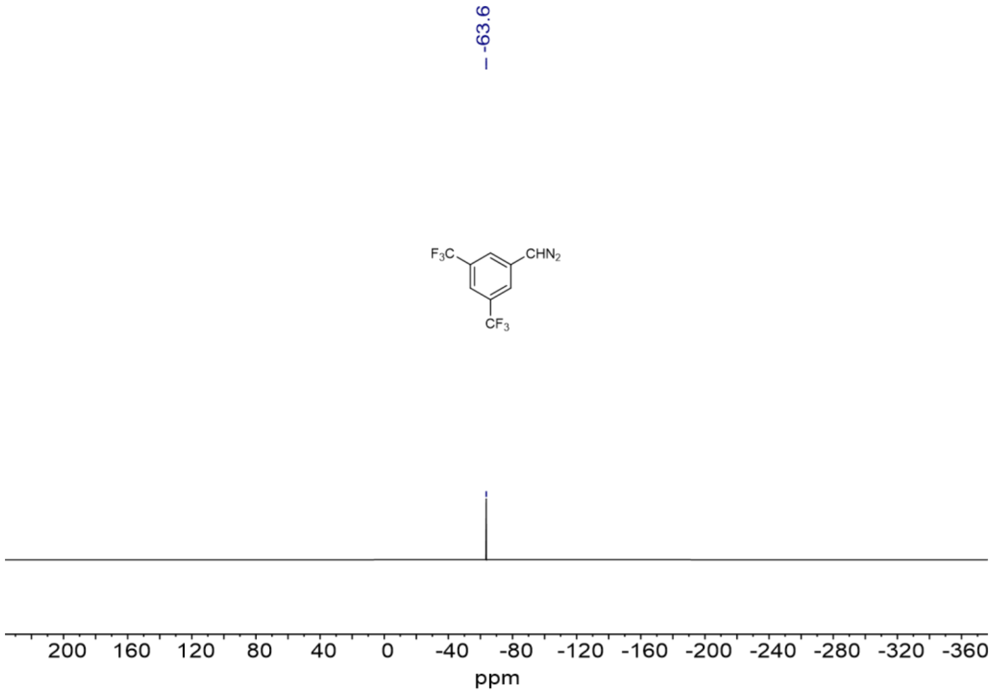

Fig. S74. ^{19}F NMR (377 MHz, $CDCl_3$, 23 °C) spectrum of 1-(diazomethyl)-3,5-bis(trifluoromethyl)benzene (**16**).

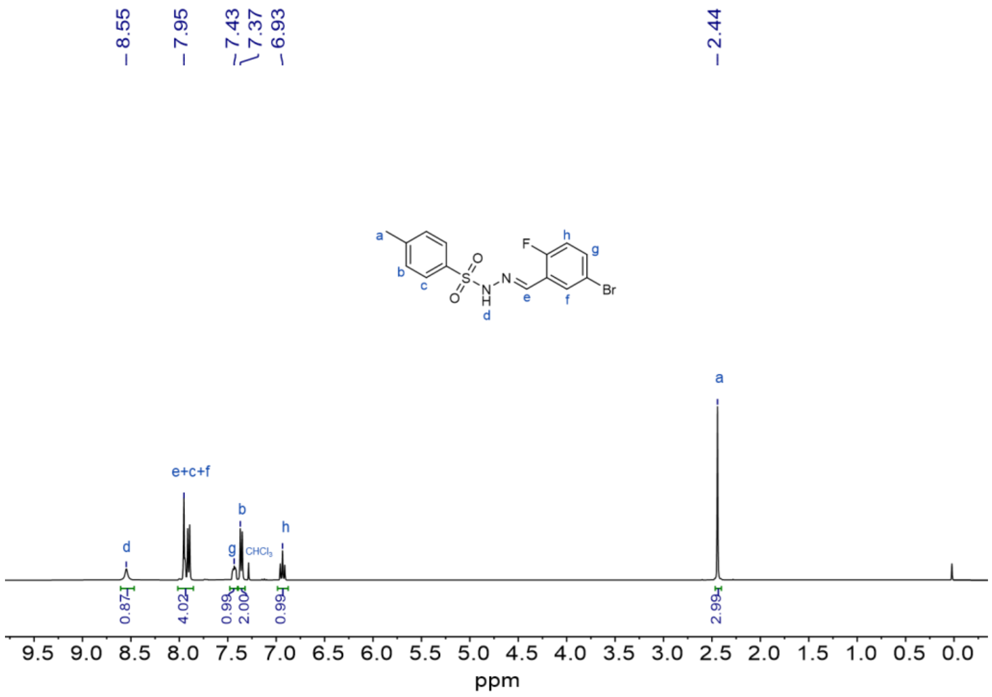

Fig. S75. ^{1}H NMR (400 MHz, $CDCl_3$, 23 ºC) spectrum of (*E*)-*N*'-(5-bromo-2-fluorobenzylidene)-4-methylbenzenesulfonohydrazide.

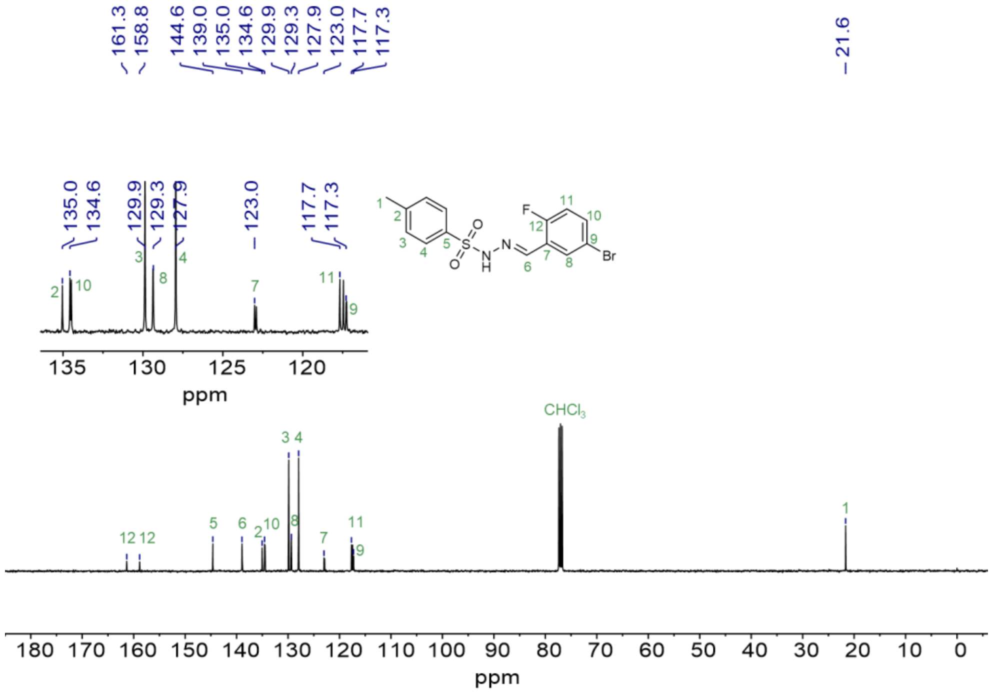

Fig. S76. ^{13}C NMR (100 MHz, $CDCl_3$, 23 ºC) spectrum of (*E*)-*N*'-(5-bromo-2-fluorobenzylidene)-4-methylbenzenesulfonohydrazide. (C7, C11, and C12 are disturbed by the fluorine atom and split).

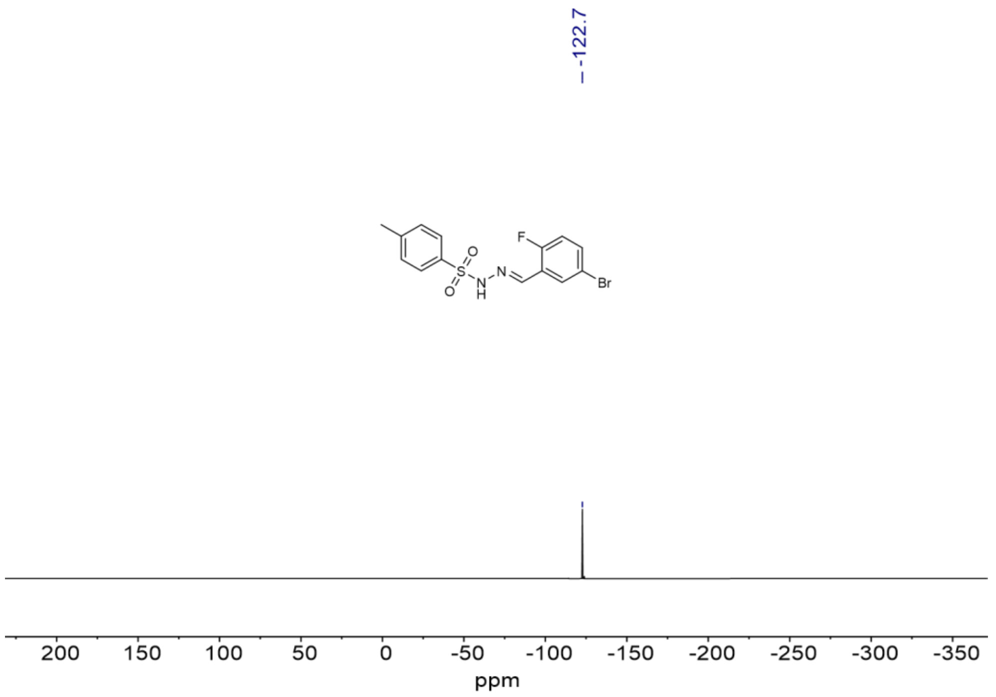

Fig. S77. ^{19}F NMR (377 MHz, $CDCl_3$, 23 ºC) spectrum of (*E*)-*N*'-(5-bromo-2-fluorobenzylidene)-4-methylbenzenesulfonohydrazide.

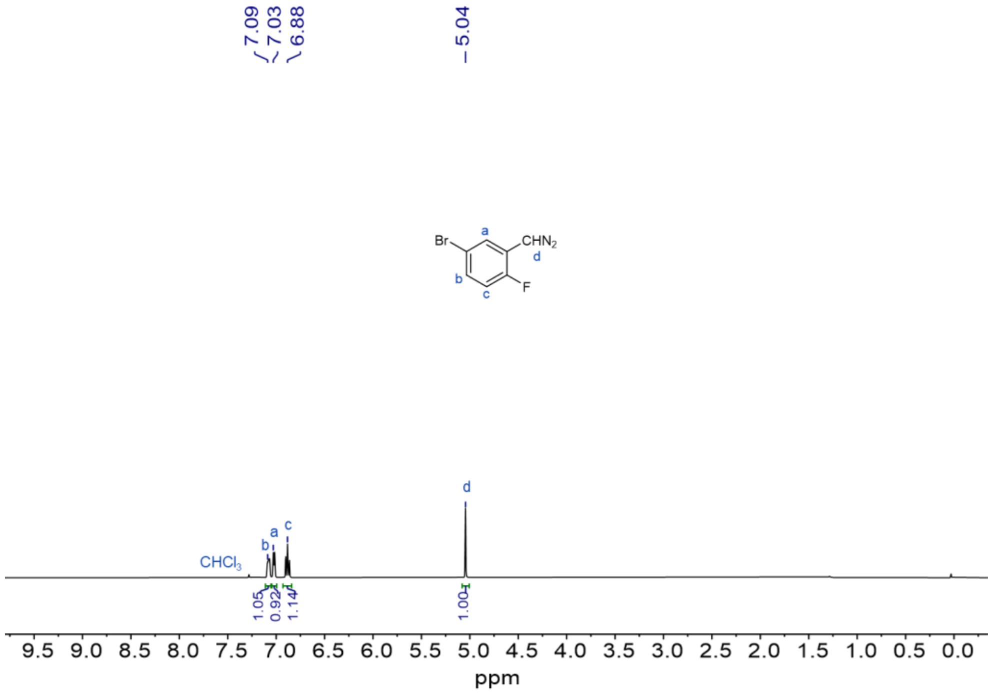

Fig. S78. ^{1}H NMR (400 MHz, $CDCl_3$, 23 ºC) spectrum of 4-bromo-2-(diazomethyl)-1-fluorobenzene (**17**).

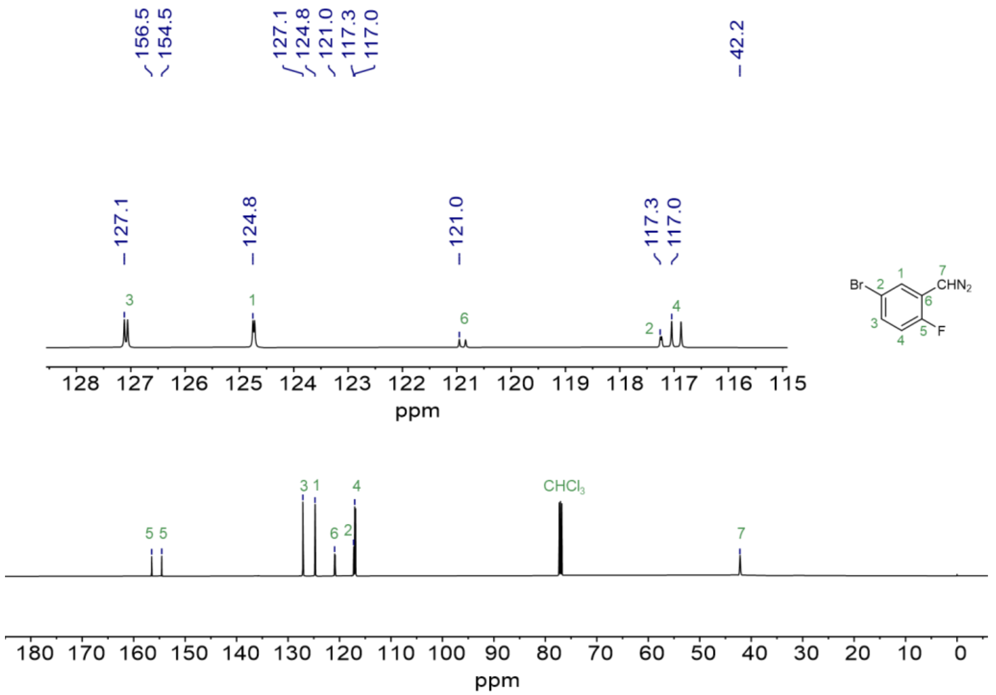

Fig. S79. ^{13}C NMR (100 MHz, $CDCl_3$, 23 ^{o}C) spectrum of 4-bromo-2-(diazomethyl)-1-fluorobenzene (**17**). (C3, C4, C5, C6 are disturbed by the fluorine atom and split).

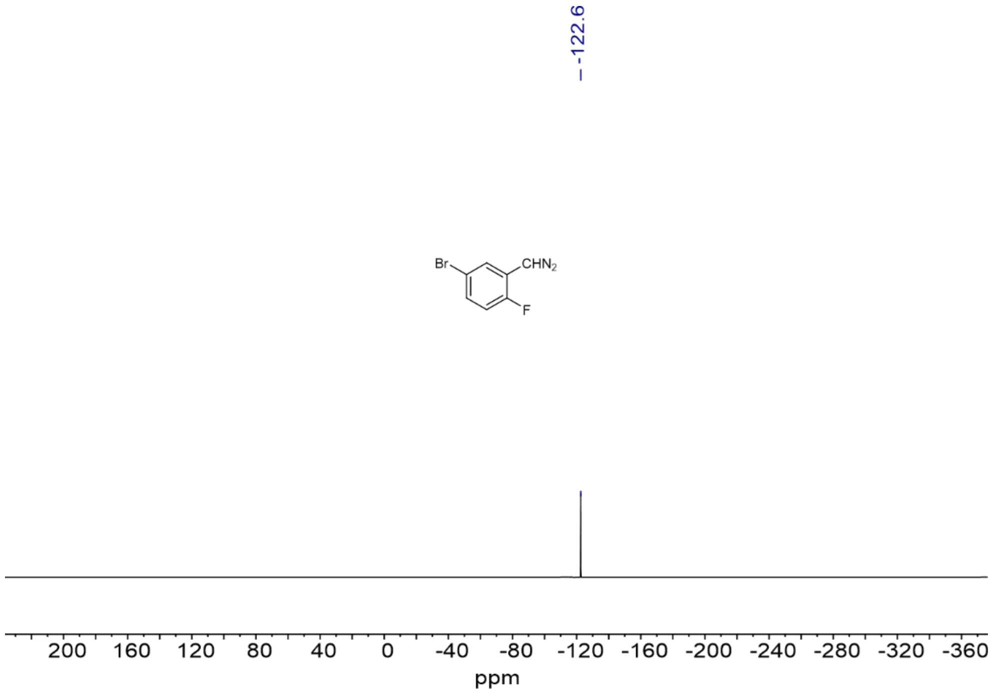

Fig. S80. ^{19}F NMR (377 MHz, $CDCl_3$, 23 ^{o}C) spectrum of 4-bromo-2-(diazomethyl)-1-fluorobenzene (**17**).

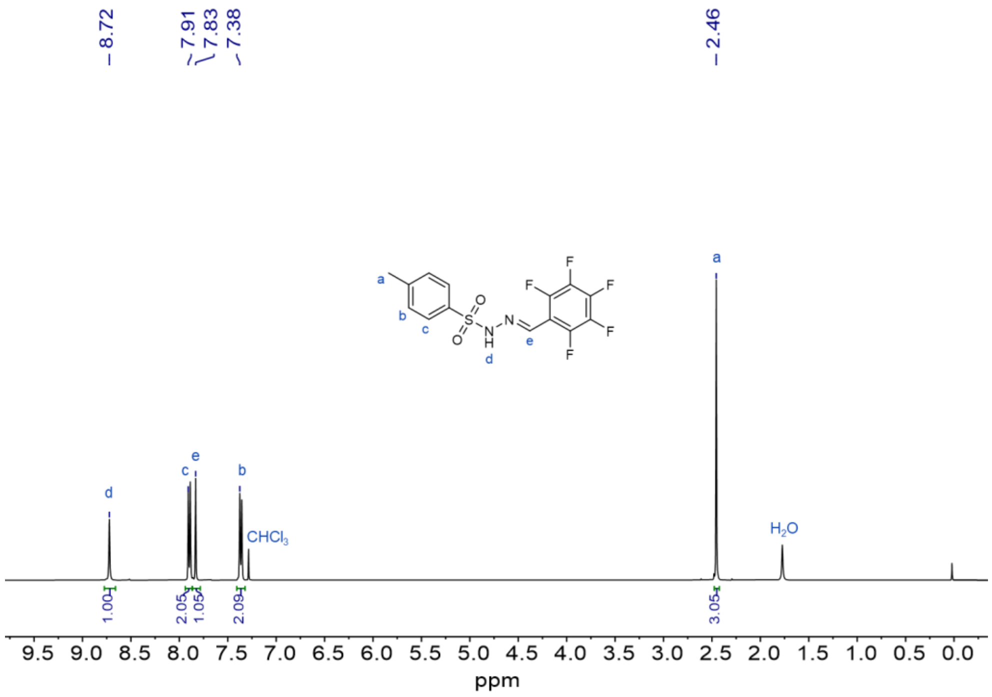

Fig. S81. ^{1}H NMR (400 MHz, $CDCl_3$, 23 °C) spectrum of (*E*)-4-methyl-*N'*-((perfluorophenyl)methylene)benzenesulfonohydrazide.

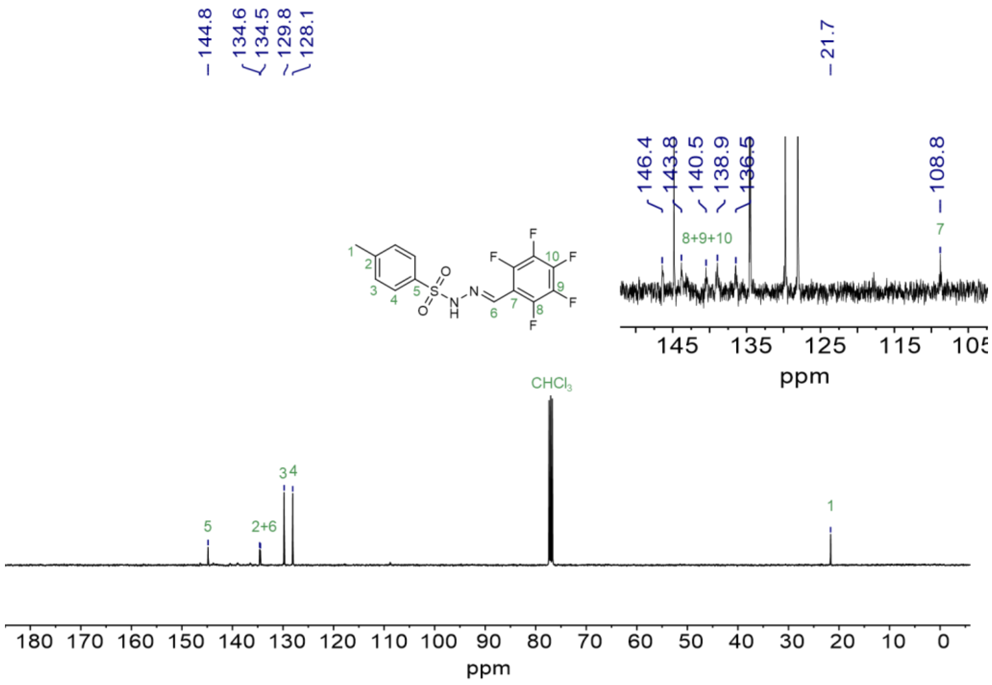

Fig. S82. ^{13}C NMR (100 MHz, $CDCl_3$, 23 °C) spectrum of (*E*)-4-methyl-*N'*-((perfluorophenyl)methylene)benzenesulfonohydrazide (7+8+9+10: the carbon atoms in the pentafluorophenyl group exhibit strong ^{13}C–^{19}F spin–spin coupling with neighboring fluorine atoms, resulting in complex splitting patterns in the ^{13}C NMR spectrum that significantly hinder the resolution and assignment of individual carbon signals).

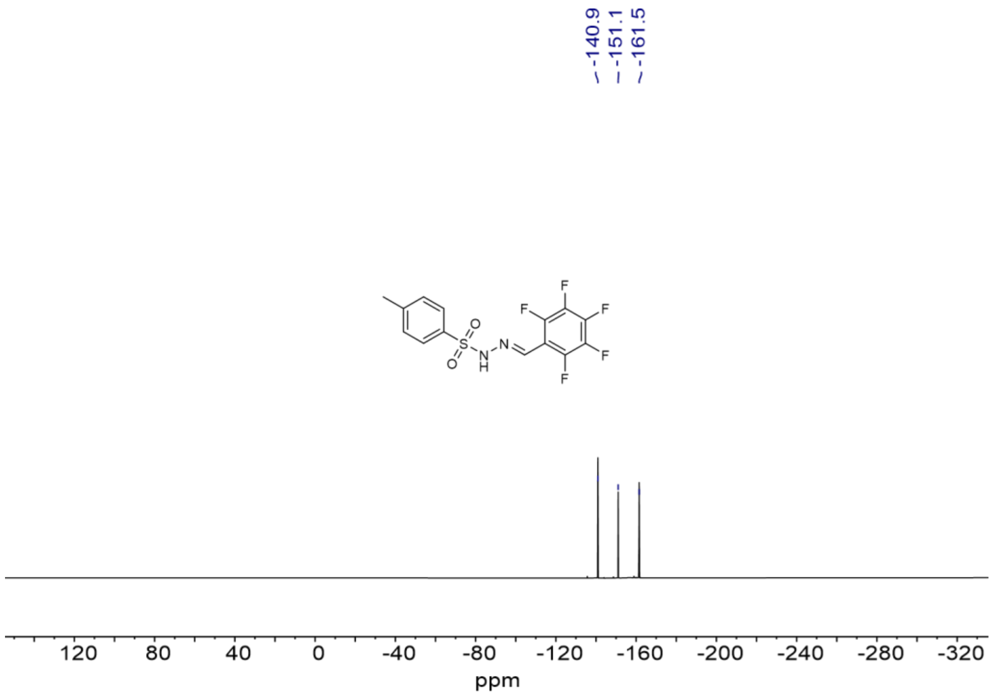

Fig. S83. ^{19}F NMR (377 MHz, $CDCl_3$, 23 °C) spectrum of (*E*)-4-methyl-*N*'-((perfluorophenyl)methylene)benzenesulfonohydrazide.

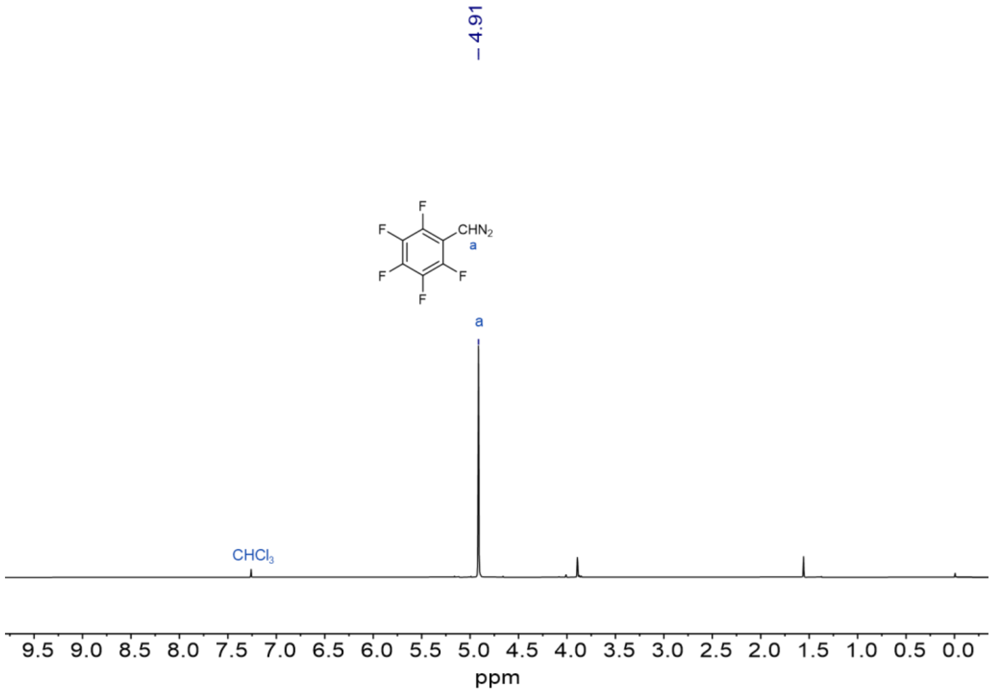

Fig. S84. ^{1}H NMR (400 MHz, $CDCl_3$, 23 °C) spectrum of 1-(diazomethyl)-2,3,4,5,6-pentafluorobenzene (**18**).

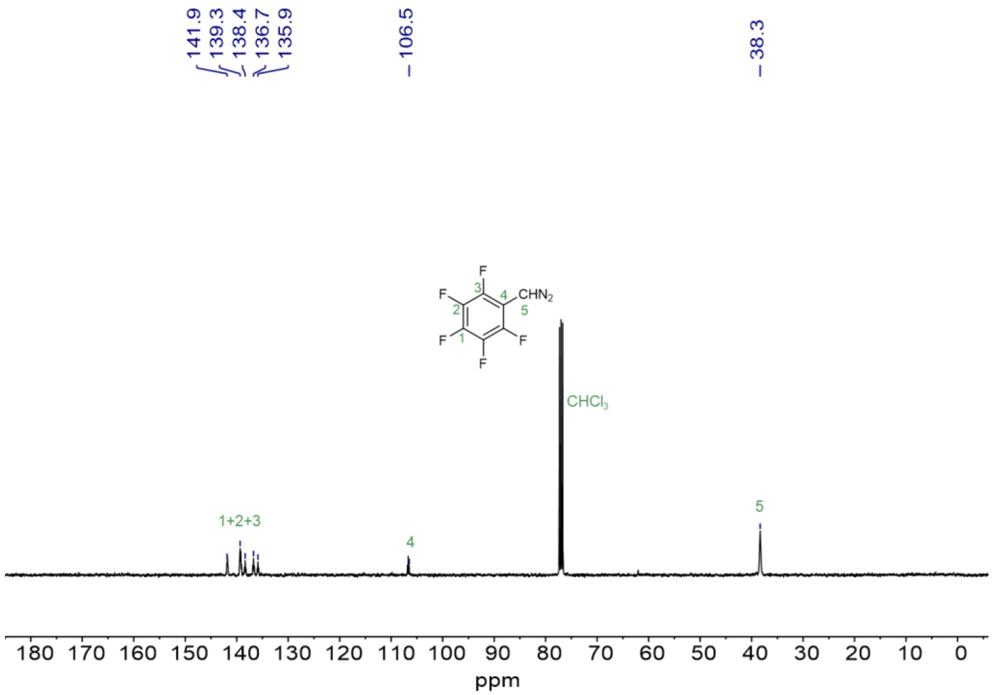

Fig. S85. ^{13}C NMR (100 MHz, $CDCl_3$, 23 ^{o}C) spectrum of 1-(diazomethyl)-2,3,4,5,6-pentafluorobenzene (**18**) (the carbon atoms in the pentafluorophenyl group exhibit strong ^{13}C–^{19}F spin–spin coupling with neighboring fluorine atoms, resulting in complex splitting patterns in the ^{13}C NMR spectrum that significantly hinder the resolution and assignment of individual carbon signals).

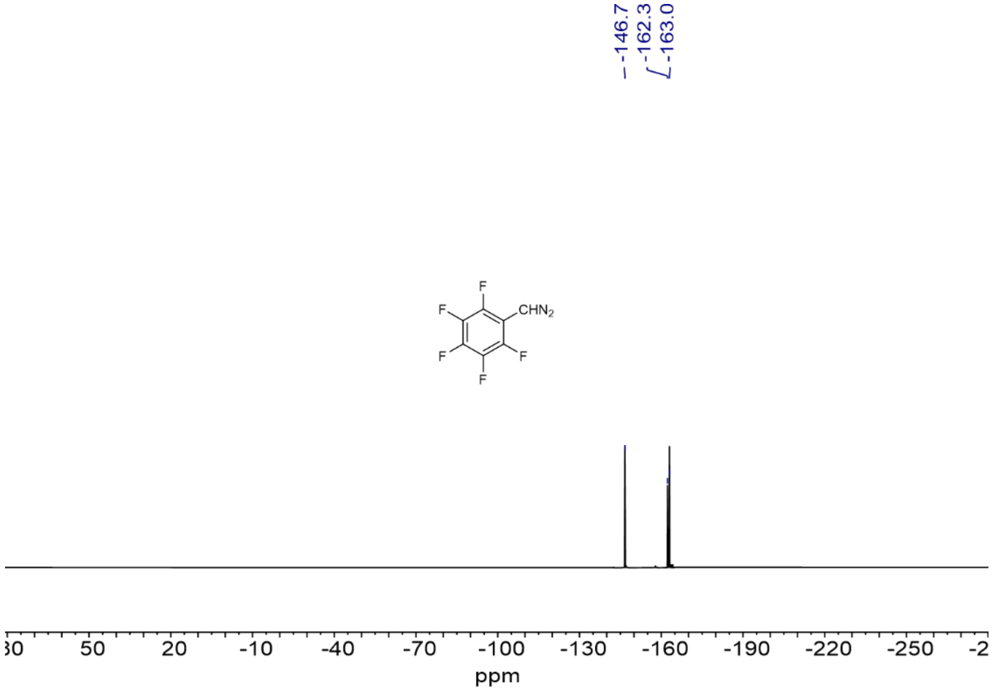

Fig. S86. ^{19}F NMR (377 MHz, $CDCl_3$, 23 ^{o}C) spectrum of 1-(diazomethyl)-2,3,4,5,6-pentafluorobenzene (18).

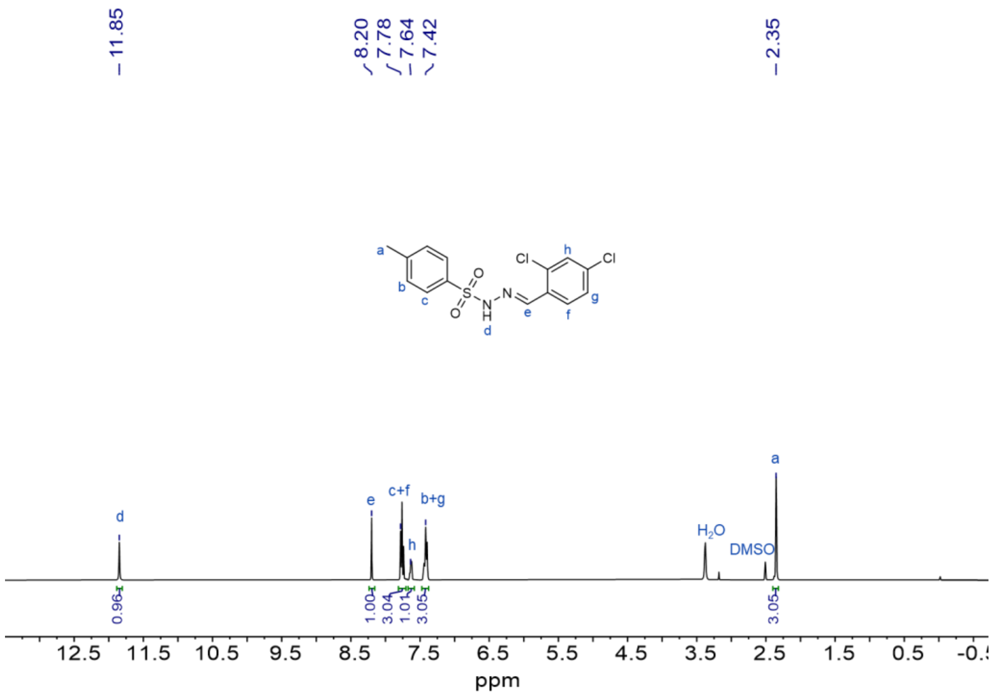

Fig. S87. ^{1}H NMR (400 MHz, DMSO-d_6, 23 ºC) spectrum of (*E*)-*N*'-(2,4-dichlorobenzylidene)-4-methylbenzenesulfonohydrazide.

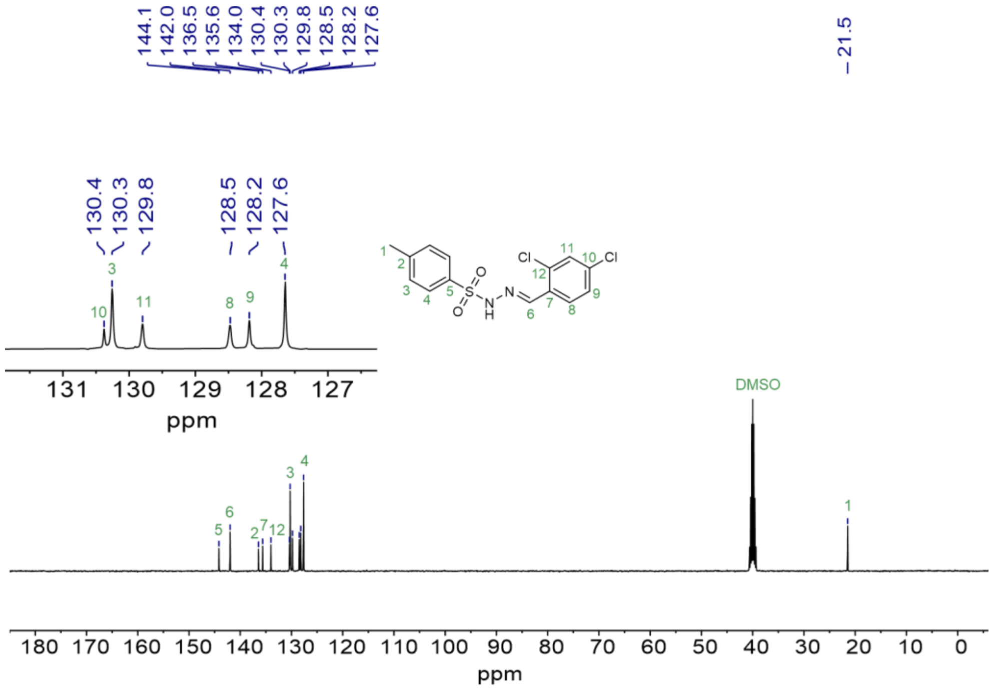

Fig. S88. ^{13}C NMR (100 MHz, DMSO-d_6, 23 ºC) spectrum of (*E*)-*N*'-(2,4-dichlorobenzylidene)-4-methylbenzenesulfonohydrazide.

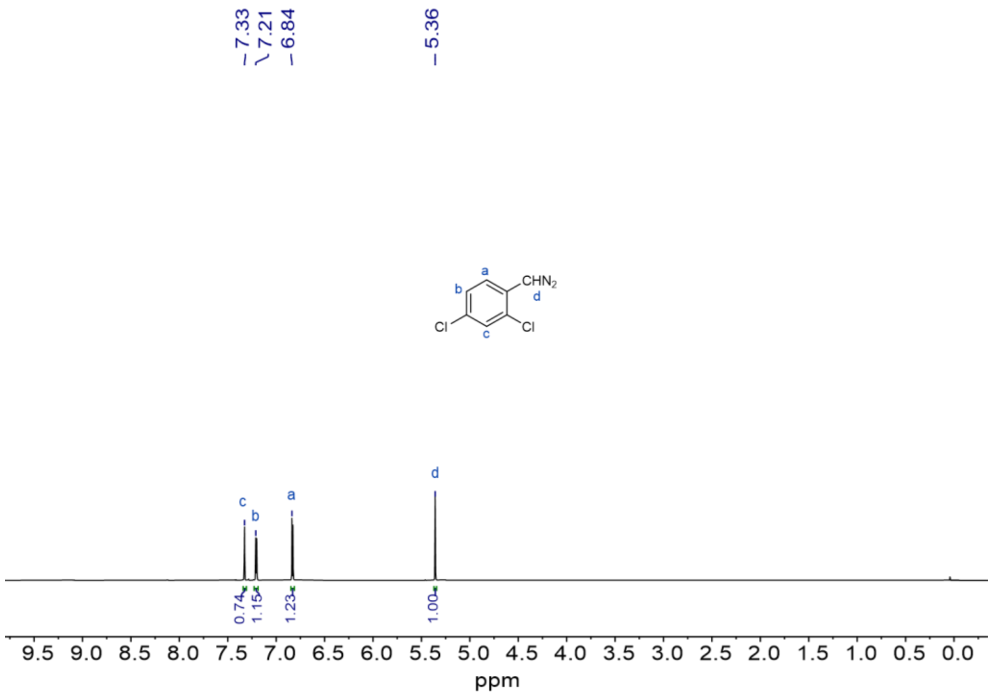

Fig. S89. ^{1}H NMR (400 MHz, $CDCl_3$, 23 °C) spectrum of 2,4-dichloro-1-(diazomethyl)benzene (19).

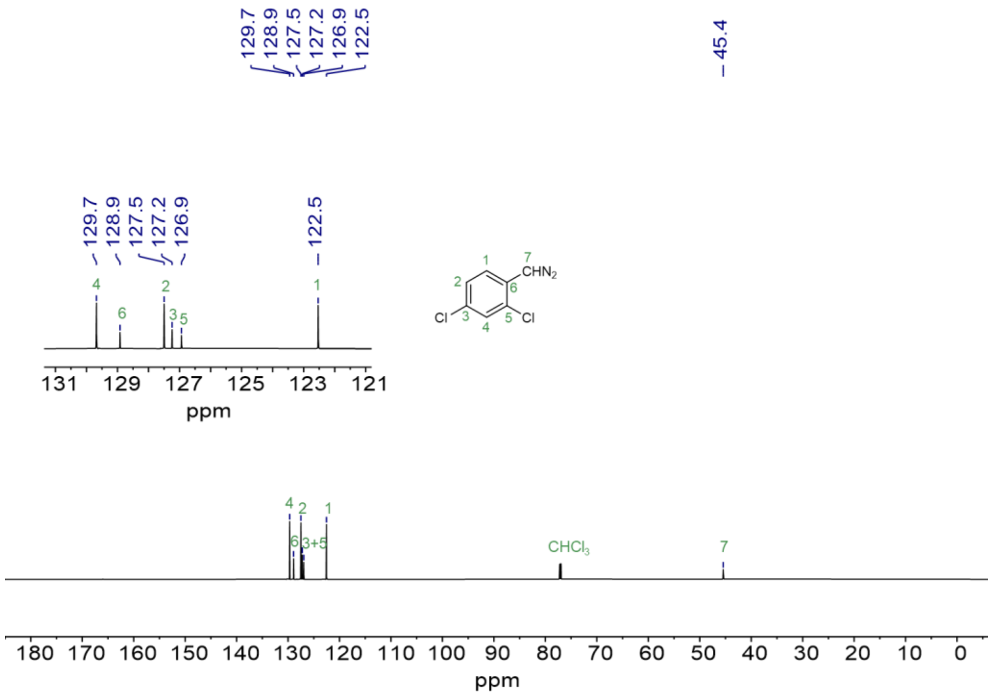

Fig. S90. ^{13}C NMR (100 MHz, $CDCl_3$, 23 °C) spectrum of 2,4-dichloro-1-(diazomethyl)benzene (19).

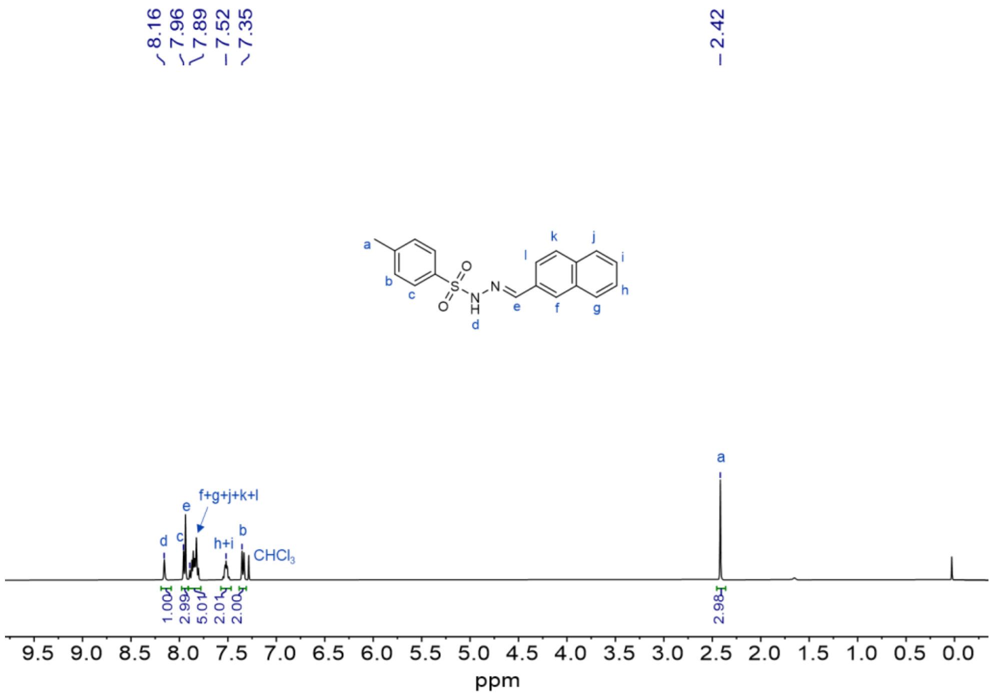

Fig. S91. ^{1}H NMR (400 MHz, $CDCl_3$, 23 °C) spectrum of (*E*)-4-methyl-*N*'-(naphthalen-2-ylmethylene)benzenesulfonohydrazide.

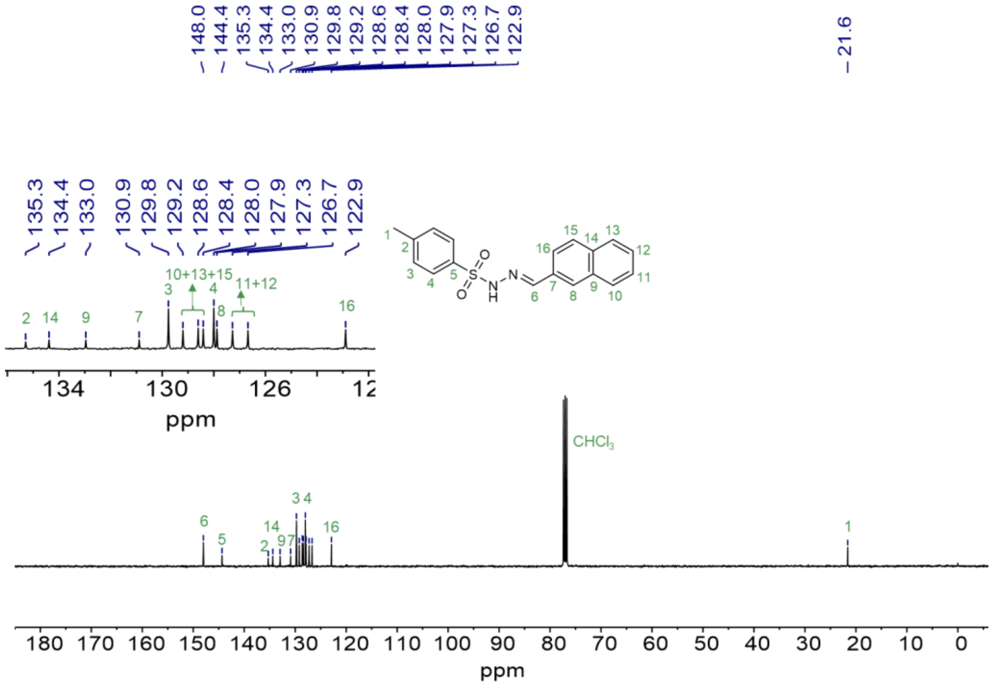

Fig. S92. ^{13}C NMR (100 MHz, $CDCl_3$, 23 °C) spectrum of (*E*)-4-methyl-*N*'-(naphthalen-2-ylmethylene)benzenesulfonohydrazide.

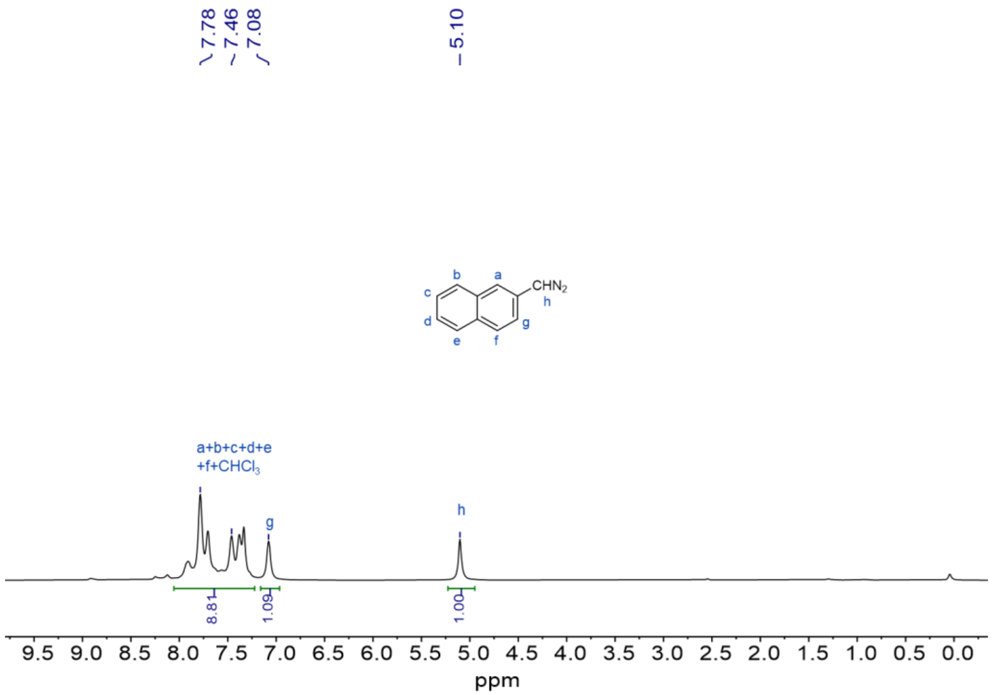

Fig. S93. ^{1}H NMR (400 MHz, $CDCl_3$, 23 °C) spectrum of 2-(diazomethyl)naphthalene (**20**).

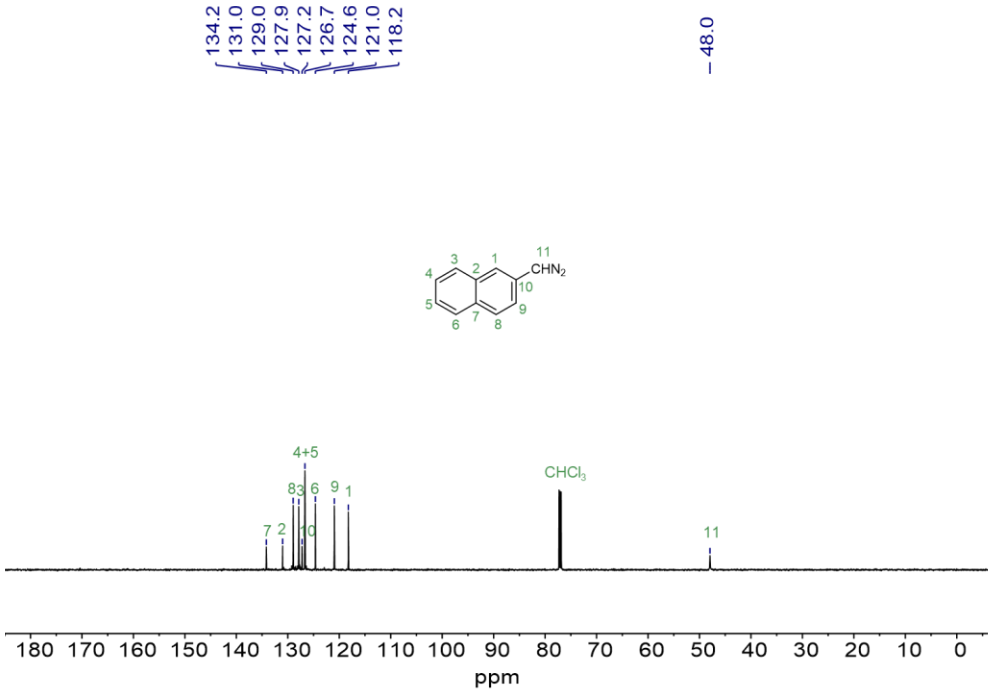

Fig. S94. ^{13}C NMR (100 MHz, $CDCl_3$, 23 °C) spectrum of 2-(diazomethyl)naphthalene (**20**).

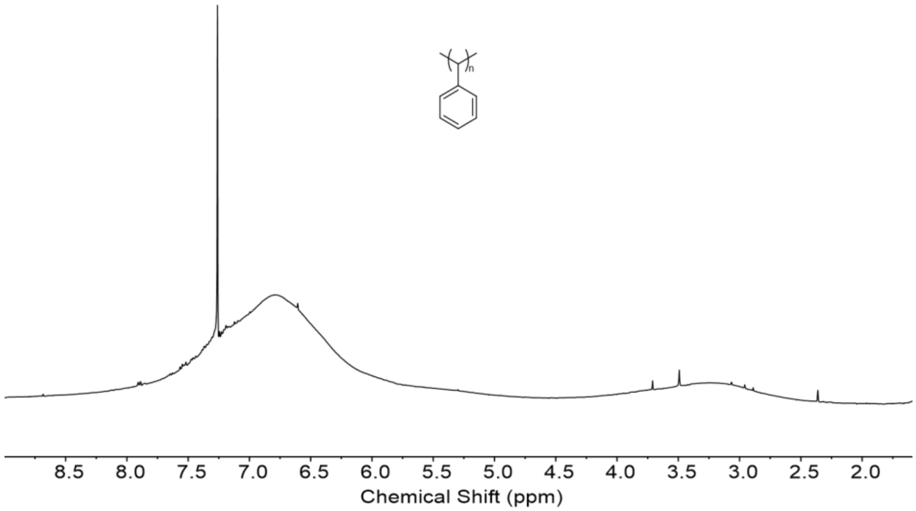

Fig. S95. ^{1}H NMR (400 MHz, $CDCl_3$, 23 ºC) spectrum of **P1**.

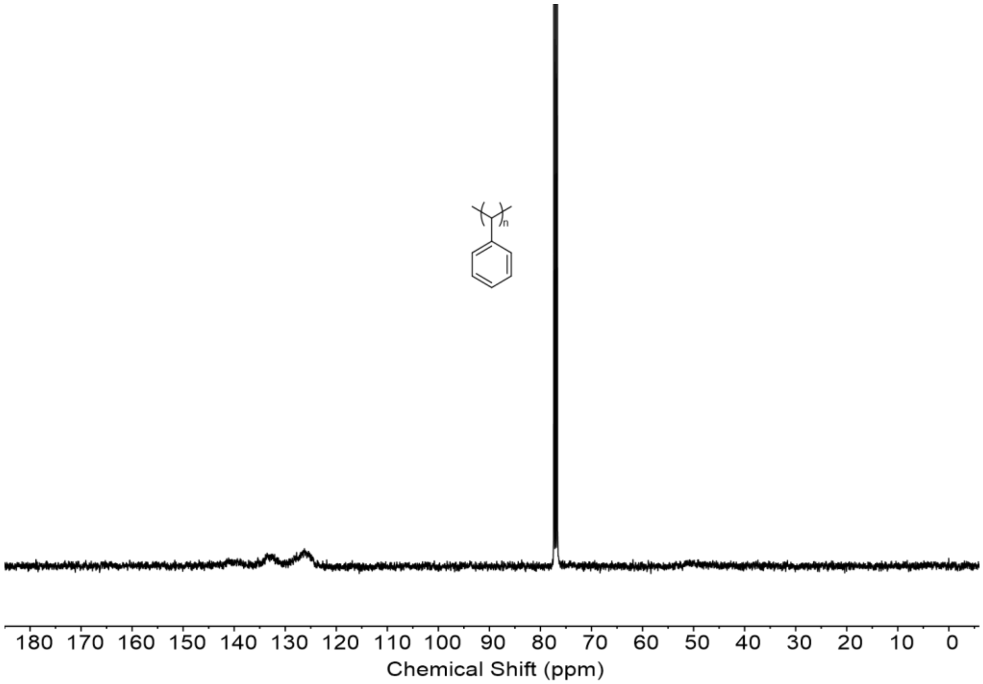

Fig. S96. ^{13}C NMR (100 MHz, $CDCl_3$, 23 ºC) spectrum of **P1**.

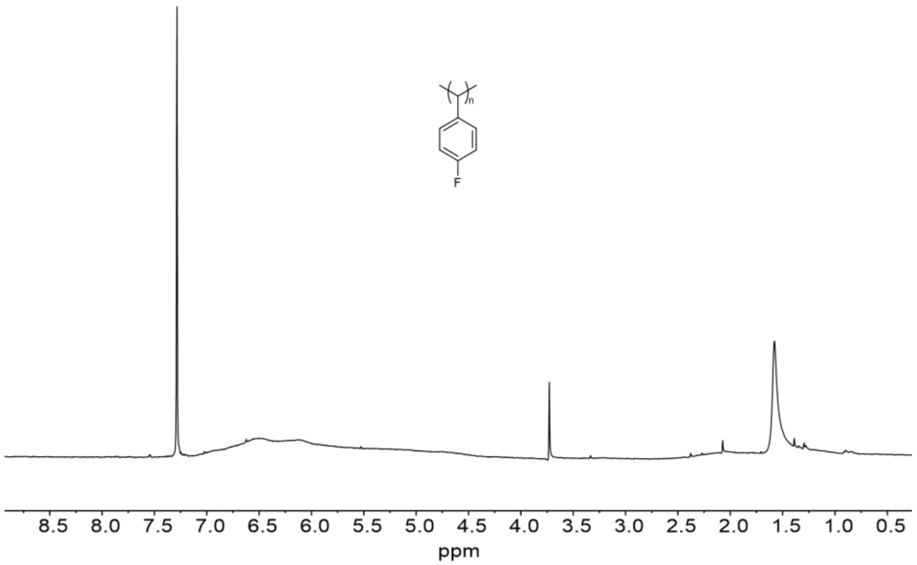

Fig. S97. ^{1}H NMR (400 MHz, $CDCl_3$, 23 °C) spectrum of **P2**.

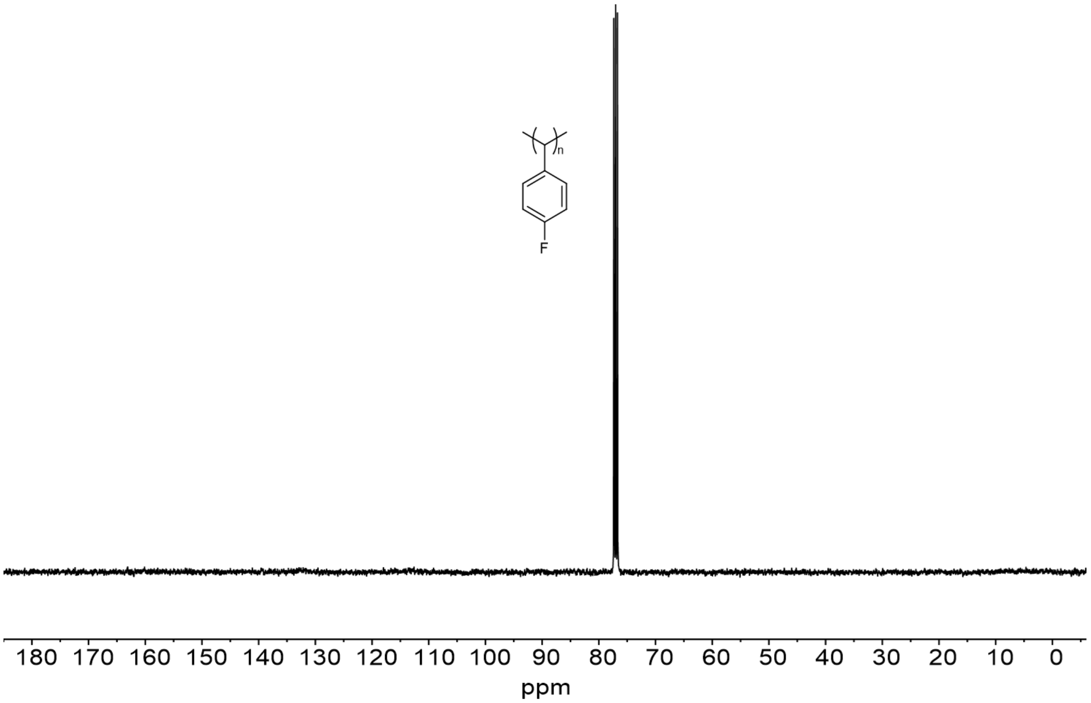

Fig. S98. ^{13}C NMR (100 MHz, $CDCl_3$, 23 °C) spectrum of **P2**.

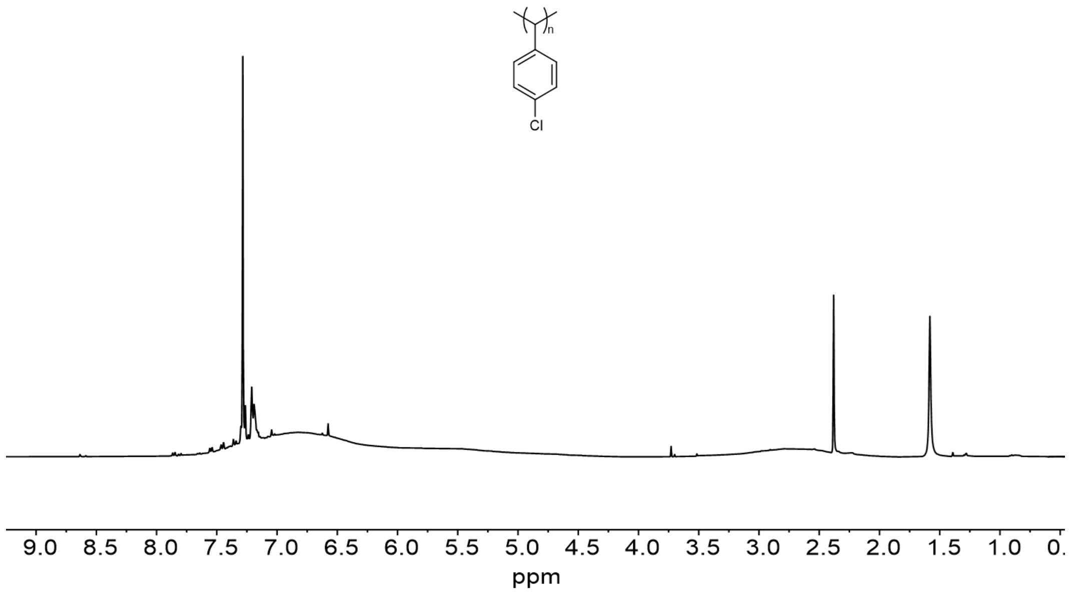

Fig. S99. ^{1}H NMR (400 MHz, $CDCl_3$, 23 ºC) spectrum of **P3**.

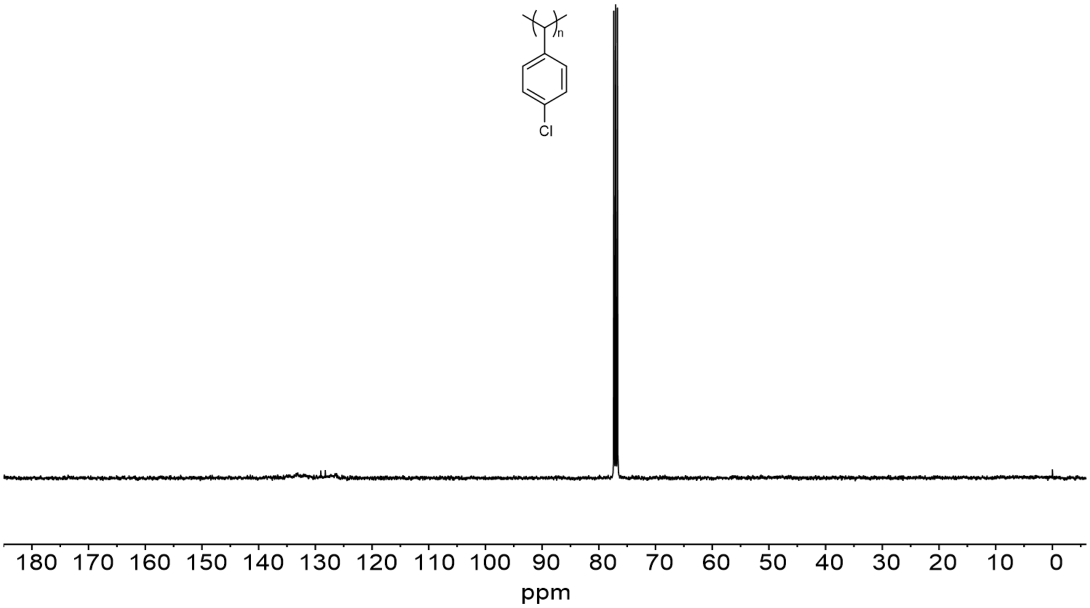

Fig. S100. ^{13}C NMR (100 MHz, $CDCl_3$, 23 ºC) spectrum of **P3**.

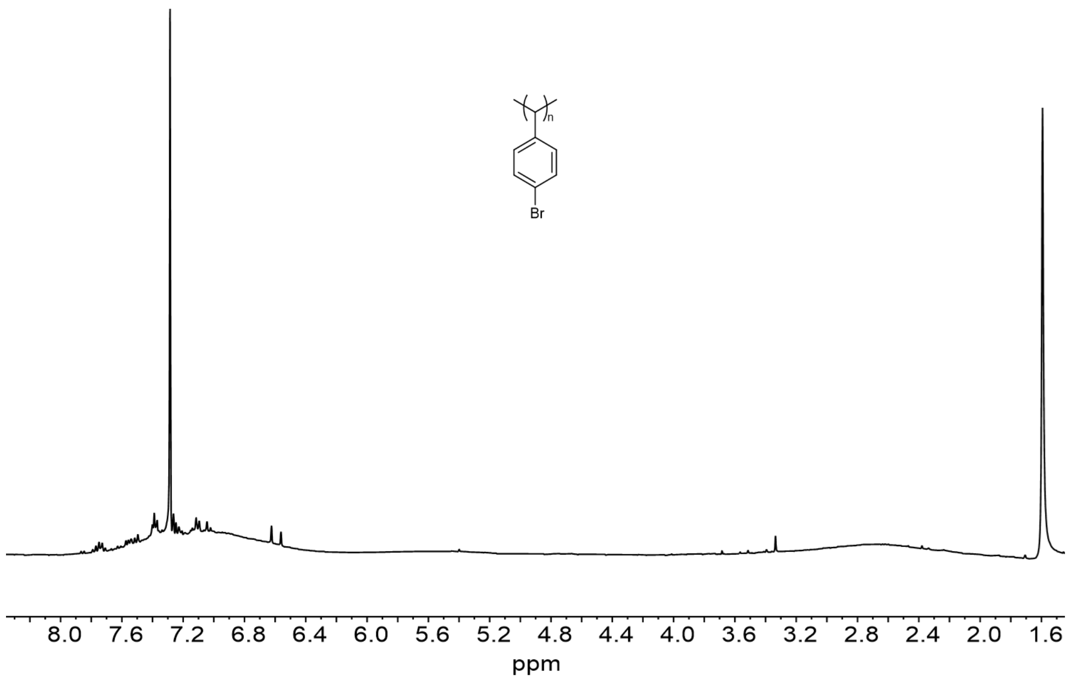

Fig. S101. ^{1}H NMR (400 MHz, $CDCl_3$, 23 ^{o}C) spectrum of **P4**.

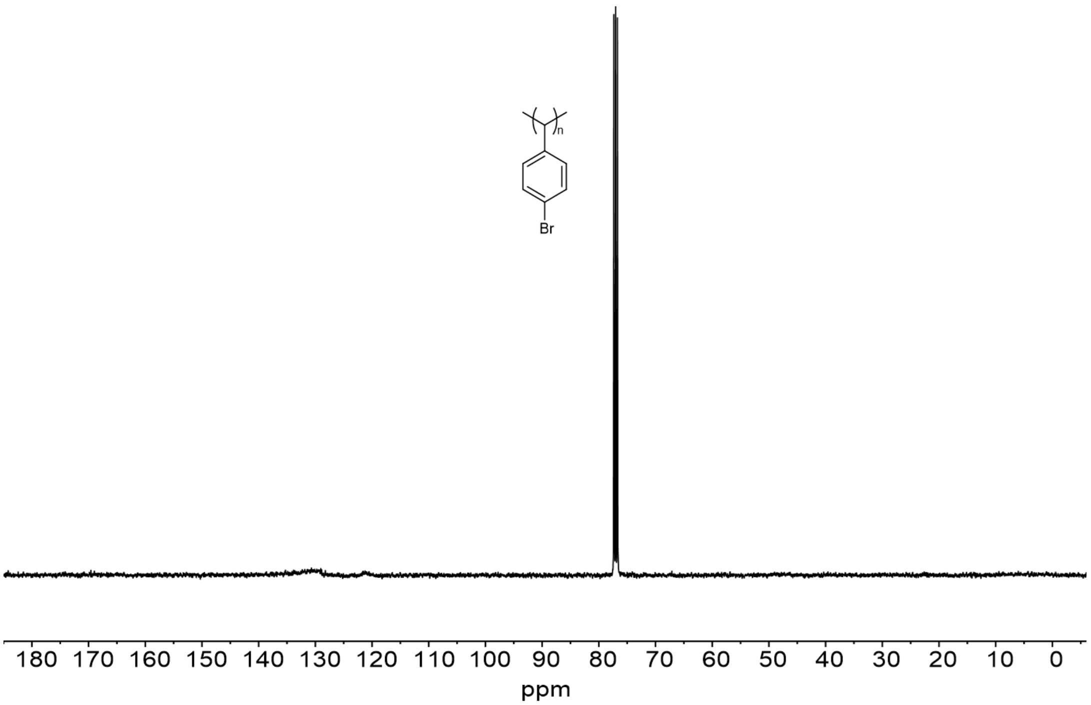

Fig. S102. ^{13}C NMR (100 MHz, $CDCl_3$, 23 ^{o}C) spectrum of **P4**.

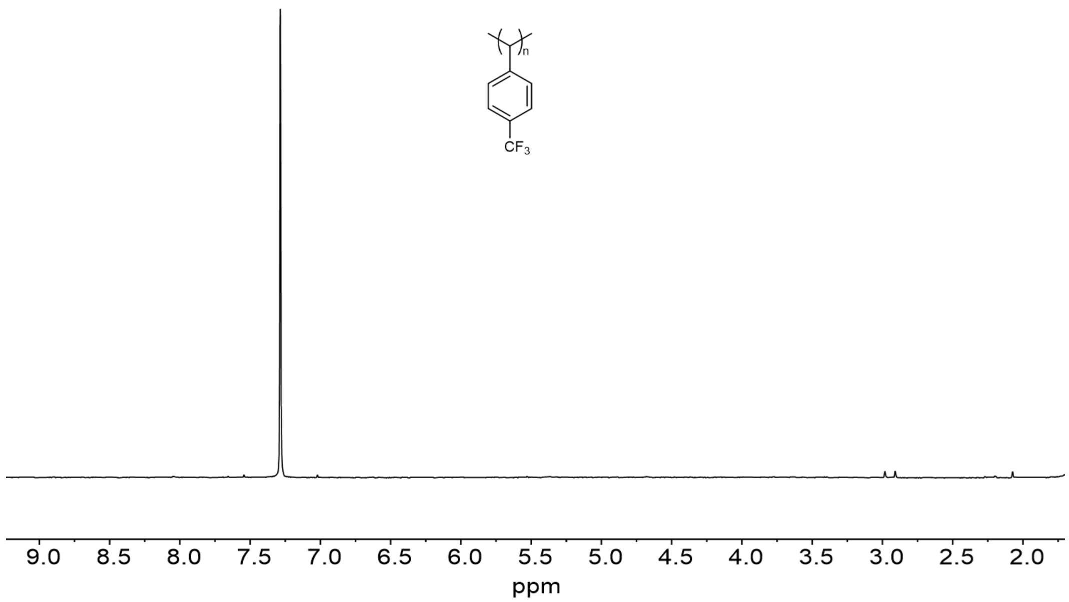

Fig. S103. ^{1}H NMR (400 MHz, $CDCl_3$, 23 ºC) spectrum of **P5** (The absence of a polymer signal is attributed to its poor solubility in the tested solvent).

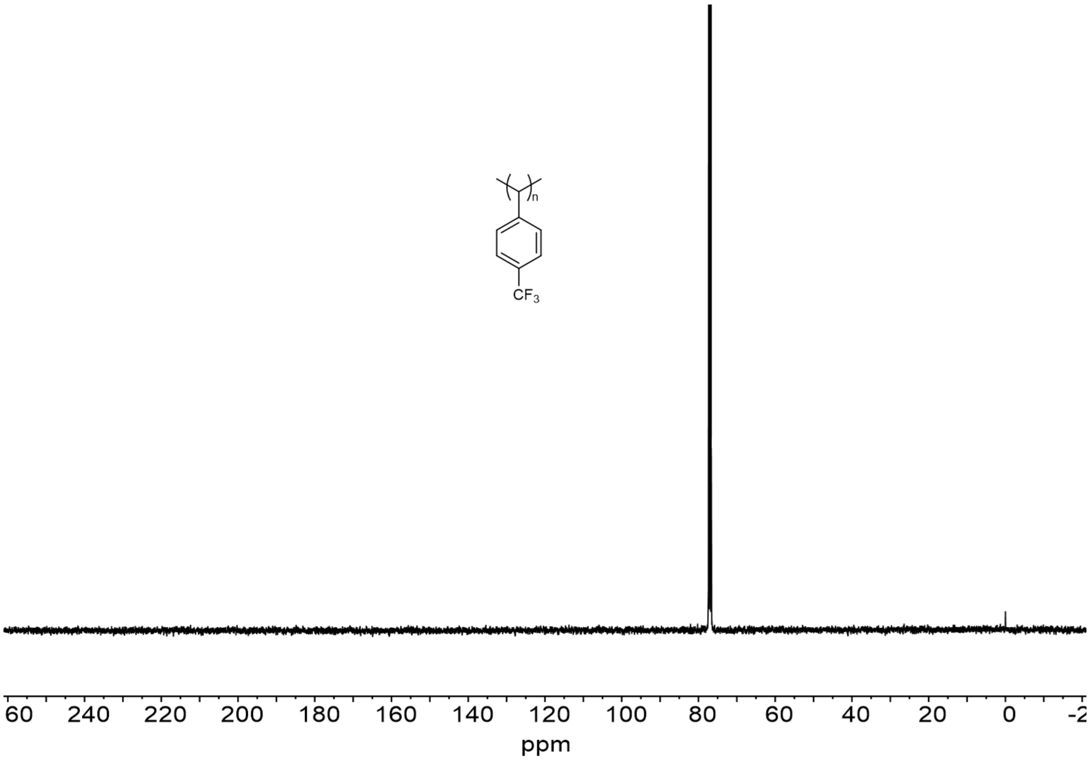

Fig. S104. ^{13}C NMR (100 MHz, $CDCl_3$, 23 ºC) spectrum of **P5** (The absence of a polymer signal is attributed to its poor solubility in the tested solvent).

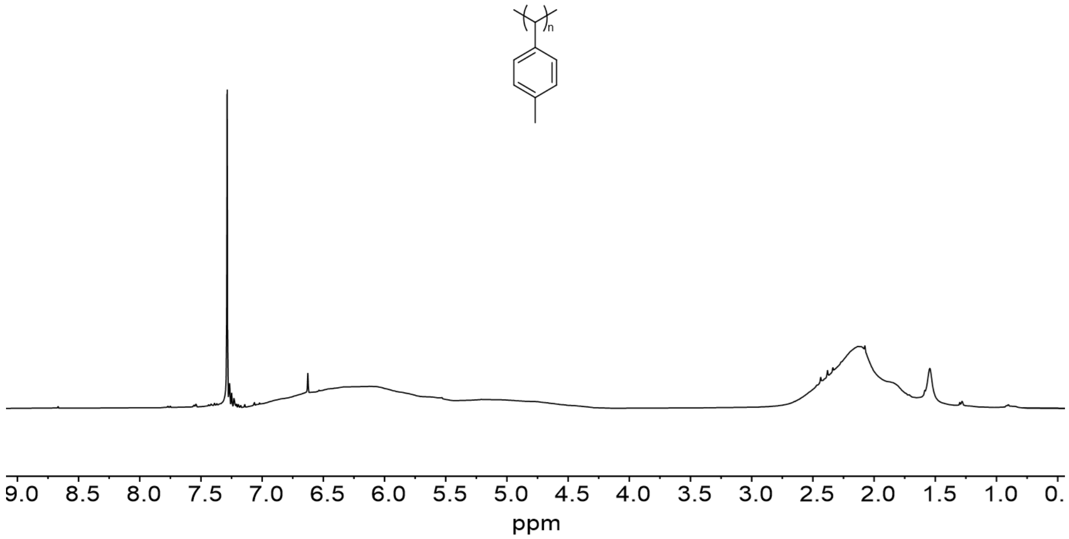

Fig. S105. ^{1}H NMR (400 MHz, $CDCl_3$, 23 °C) spectrum of **P6**.

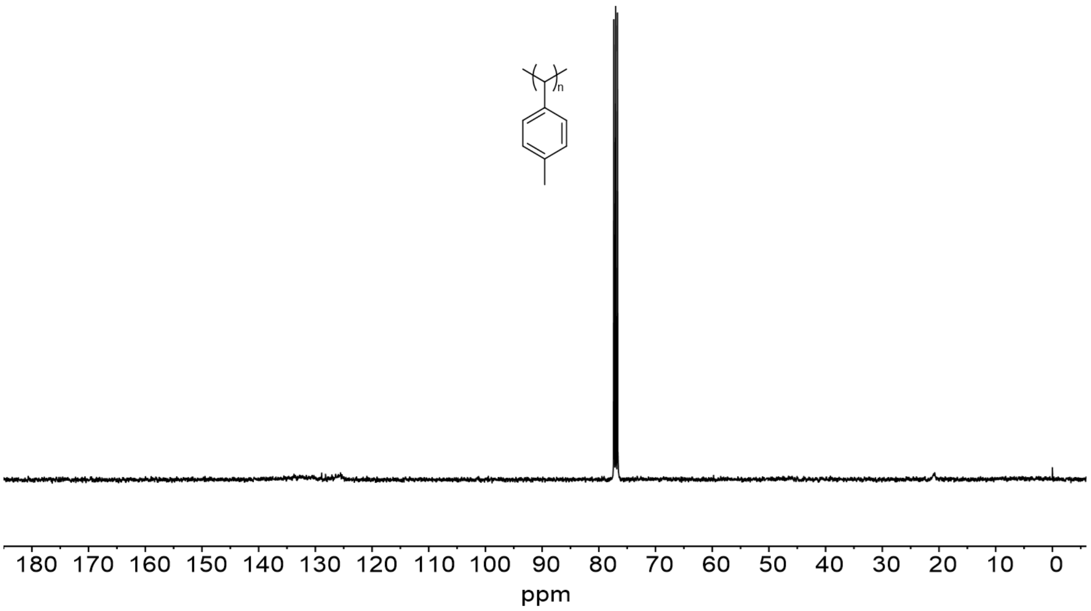

Fig. S106. ^{13}C NMR (100 MHz, $CDCl_3$, 23 °C) spectrum of **P6**.

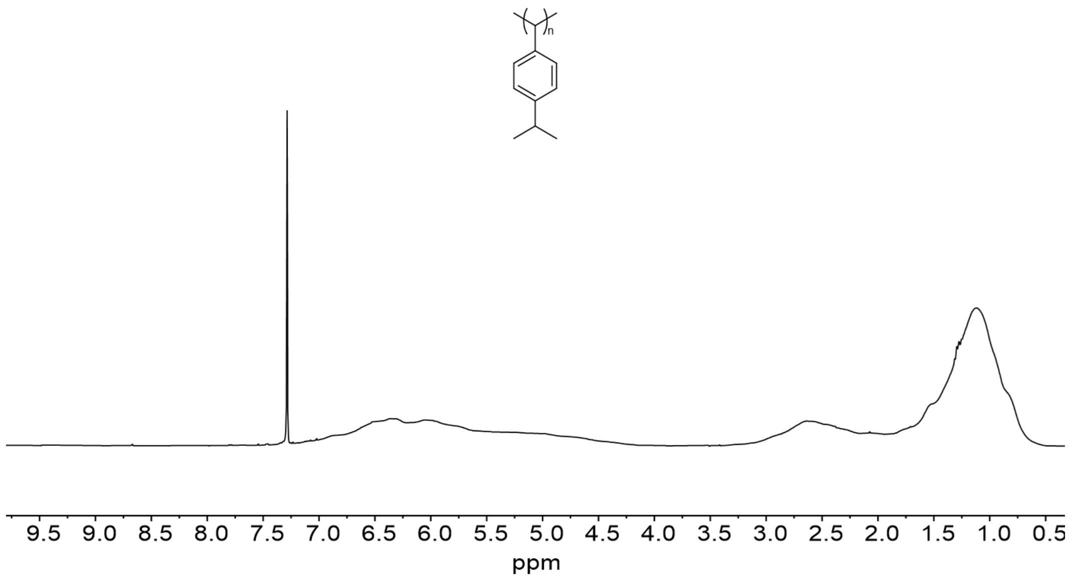

Fig. S107. ^{1}H NMR (400 MHz, $CDCl_3$, 23 °C) spectrum of **P7**.

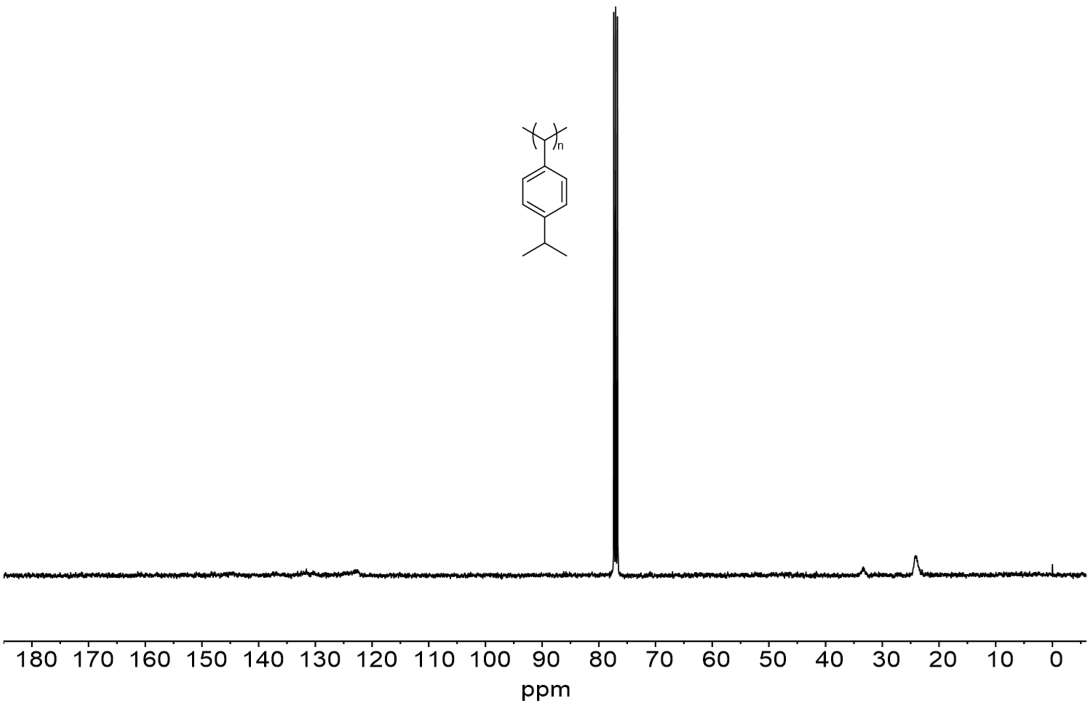

Fig. S108. ^{13}C NMR (100 MHz, $CDCl_3$, 23 °C) spectrum of **P7**.

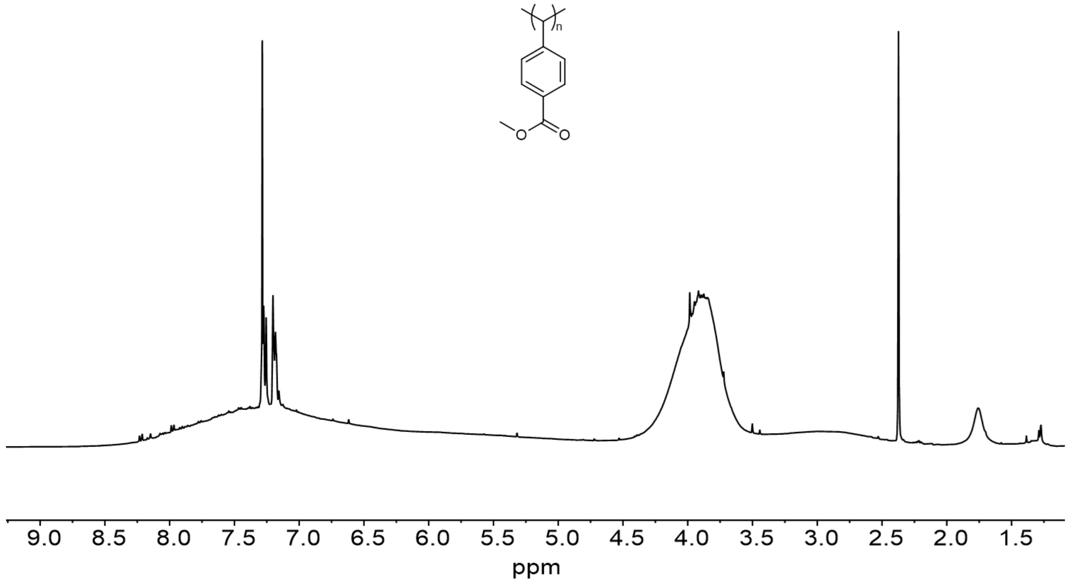

Fig. S109. ^{1}H NMR (400 MHz, $CDCl_3$, 23 °C) spectrum of **P8**.

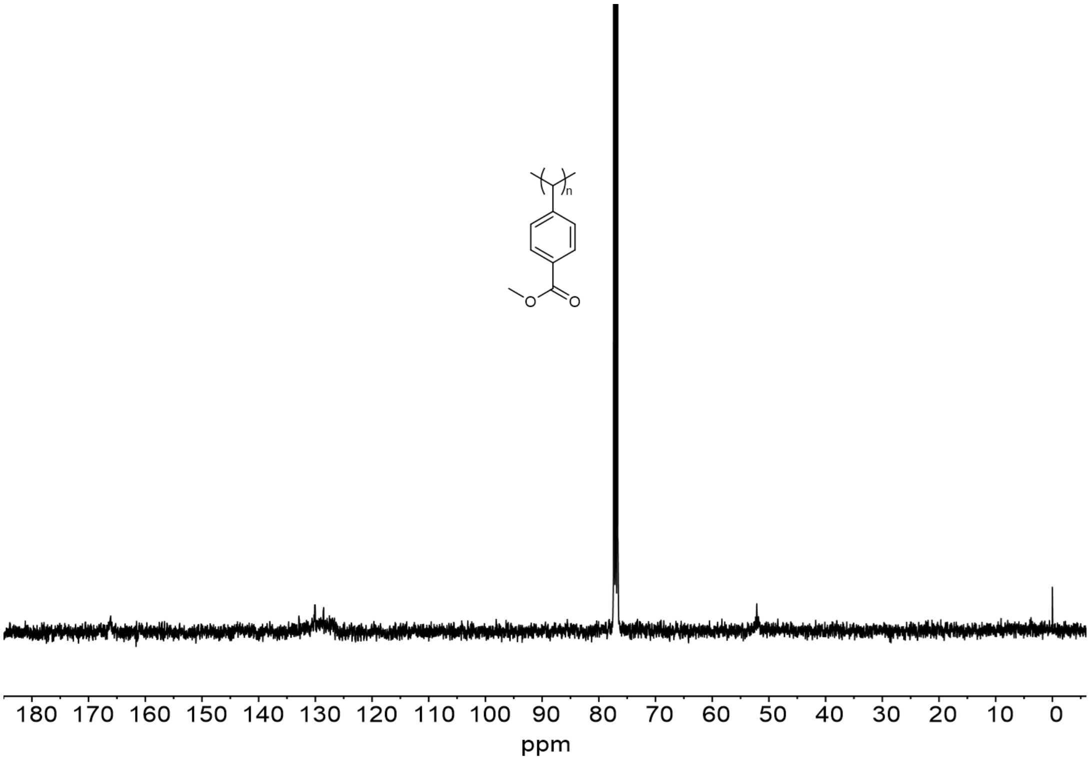

Fig. S110. ^{13}C NMR (100 MHz, $CDCl_3$, 23 °C) spectrum of **P8**.

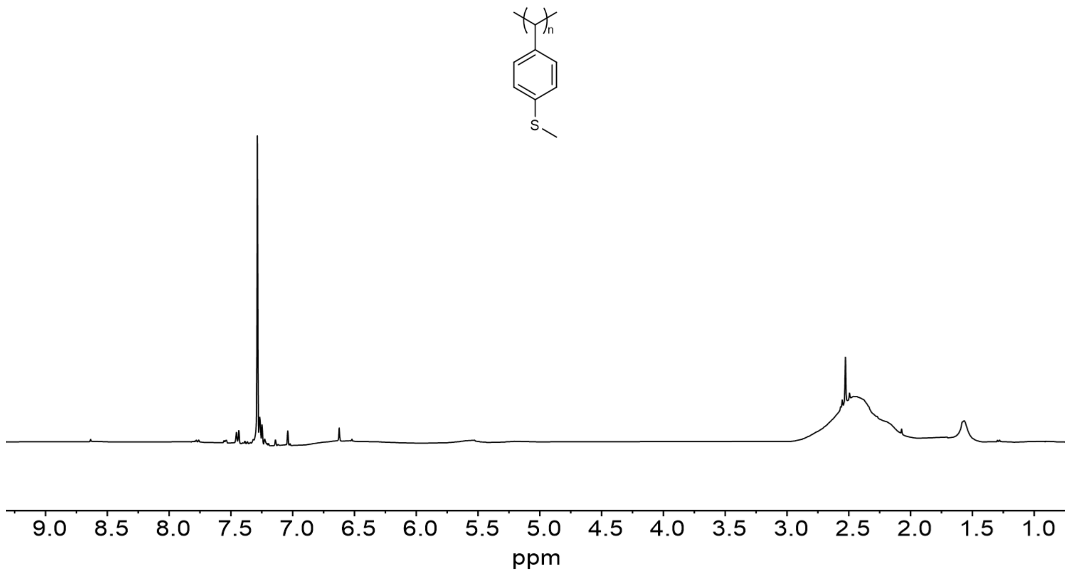

Fig. S111. ^{1}H NMR (400 MHz, $CDCl_3$, 23 ºC) spectrum of **P9**.

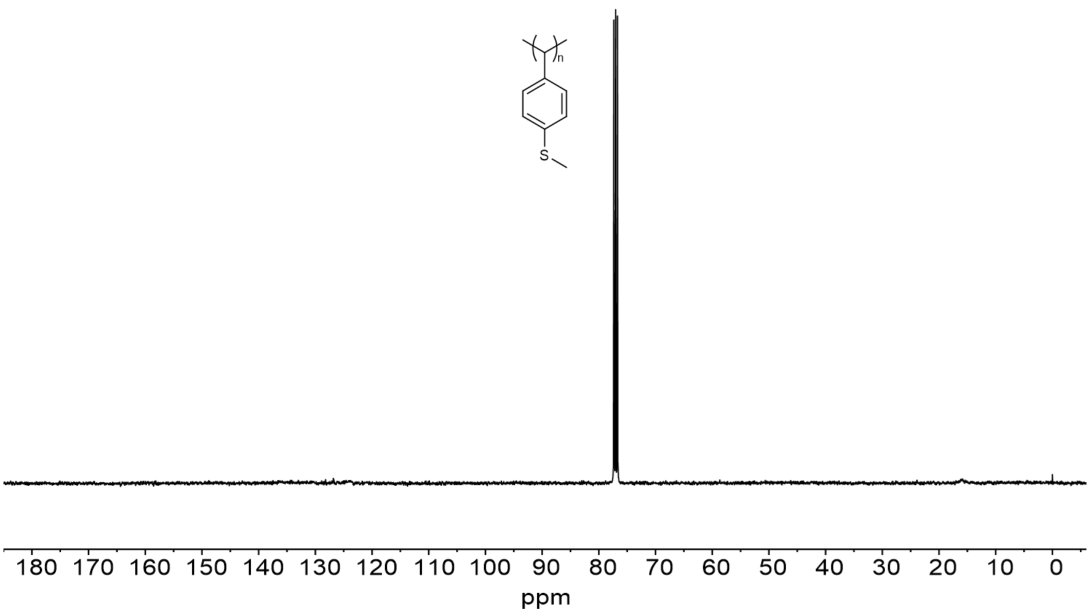

Fig. S112. ^{13}C NMR (100 MHz, $CDCl_3$, 23 ºC) spectrum of **P9**.

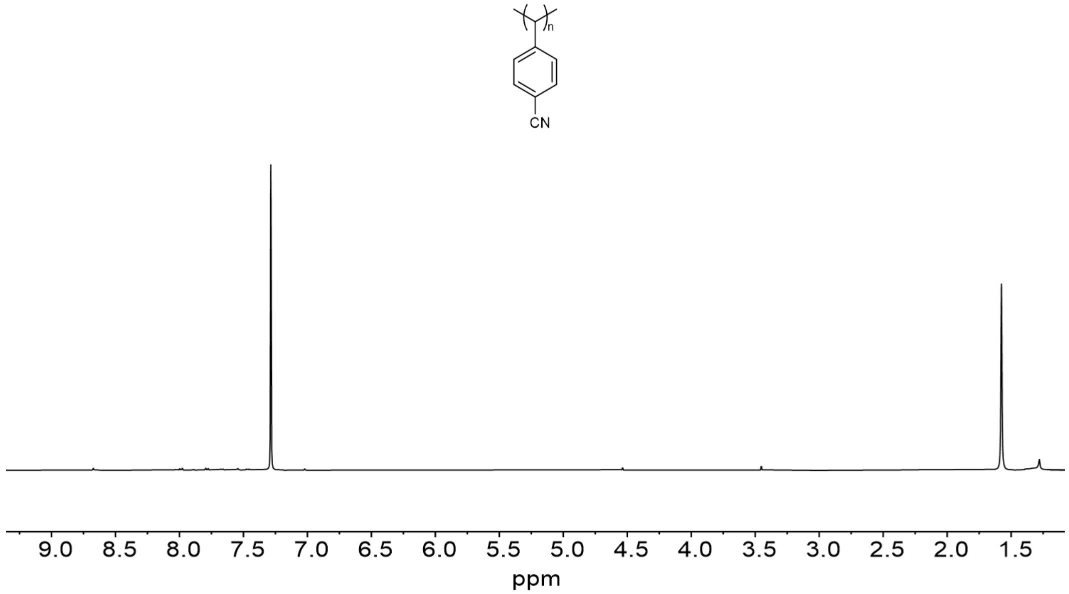

Fig. S113. ^{1}H NMR (400 MHz, $CDCl_3$, 23 ^{o}C) spectrum of **P10** (The absence of a polymer signal is attributed to its poor solubility in the tested solvent).

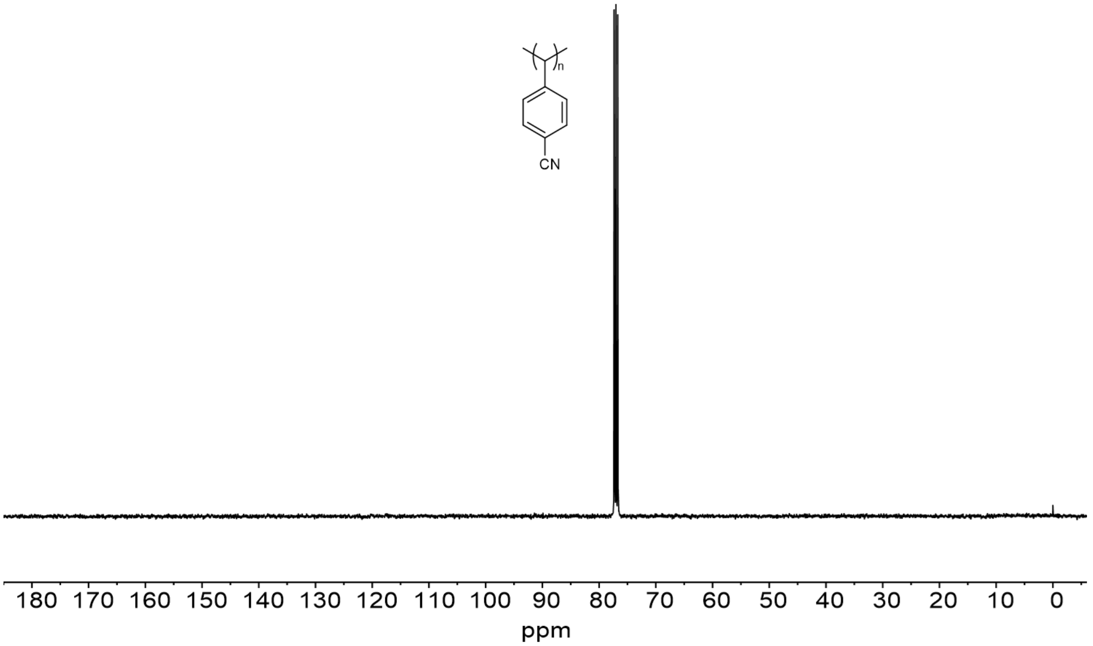

Fig. S114. ^{13}C NMR (100 MHz, $CDCl_3$, 23 ^{o}C) spectrum of **P10** (The absence of a polymer signal is attributed to its poor solubility in the tested solvent).

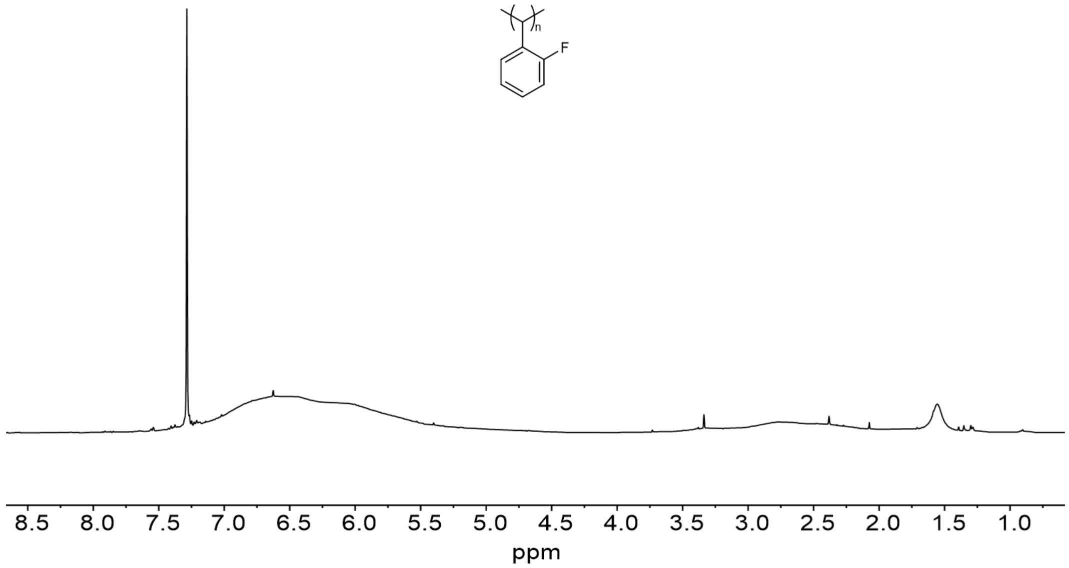

Fig. S115. ^{1}H NMR (400 MHz, $CDCl_3$, 23 °C) spectrum of **P11**.

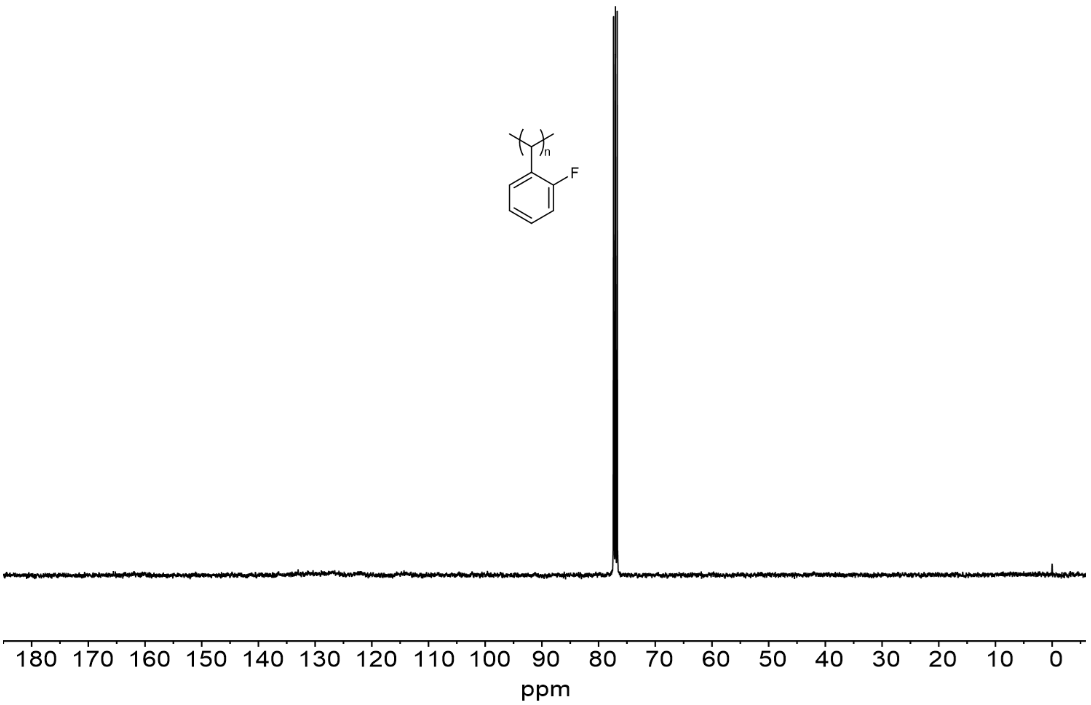

Fig. S116. ^{13}C NMR (100 MHz, $CDCl_3$, 23 °C) spectrum of **P11**.

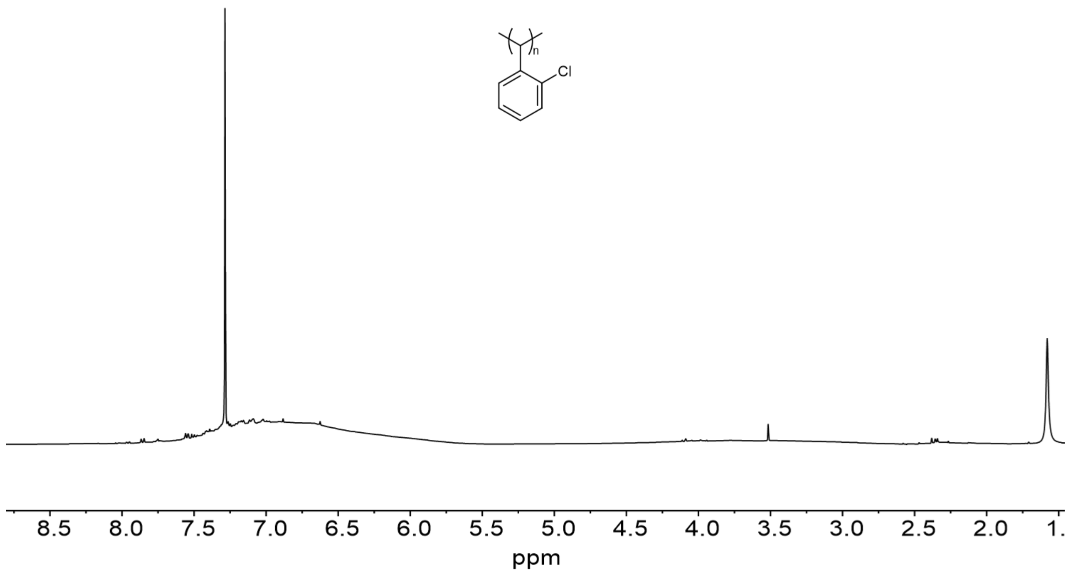

Fig. S117. ^{1}H NMR (400 MHz, $CDCl_3$, 23 ^{o}C) spectrum of **P12**.

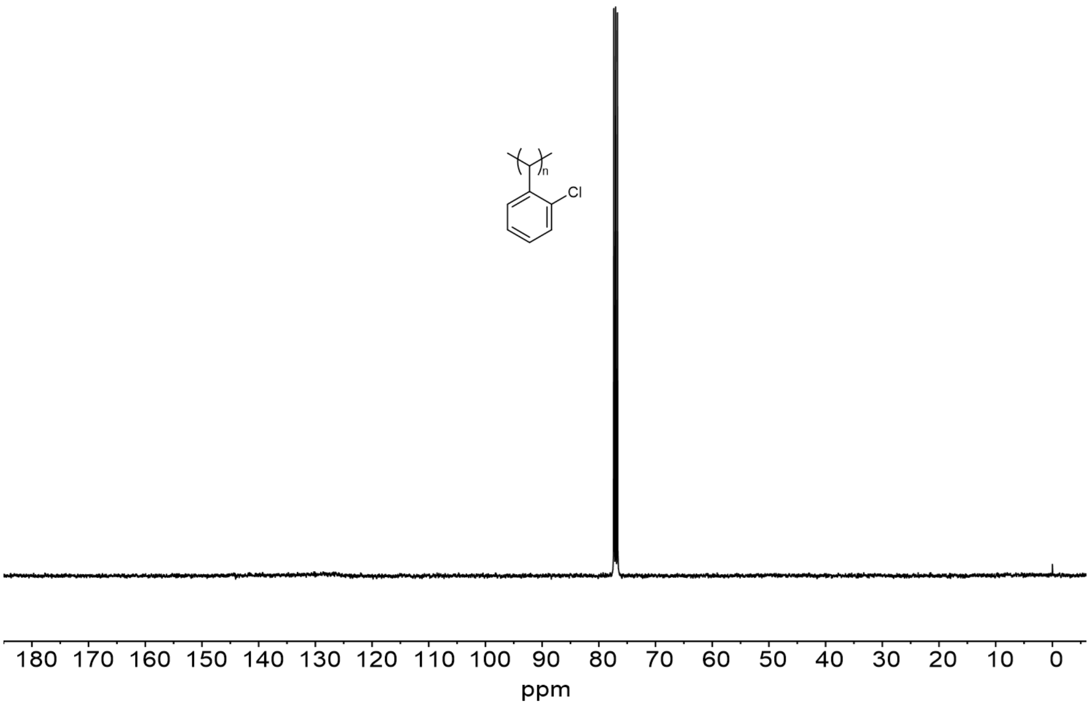

Fig. S118. ^{13}C NMR (100 MHz, $CDCl_3$, 23 ^{o}C) spectrum of **P12**.

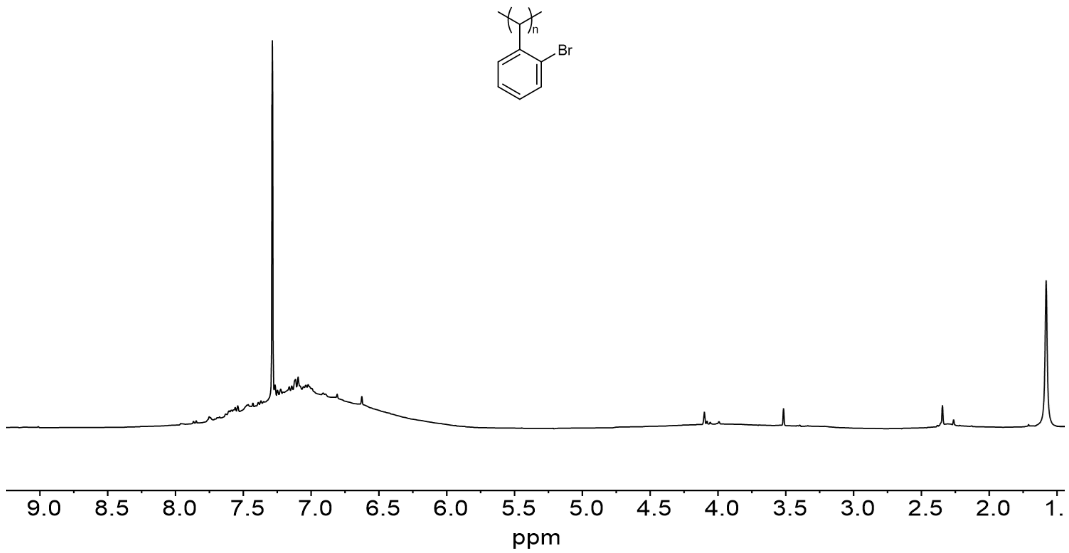

Fig. S119. ^{1}H NMR (400 MHz, $CDCl_3$, 23 ^{o}C) spectrum of **P13**.

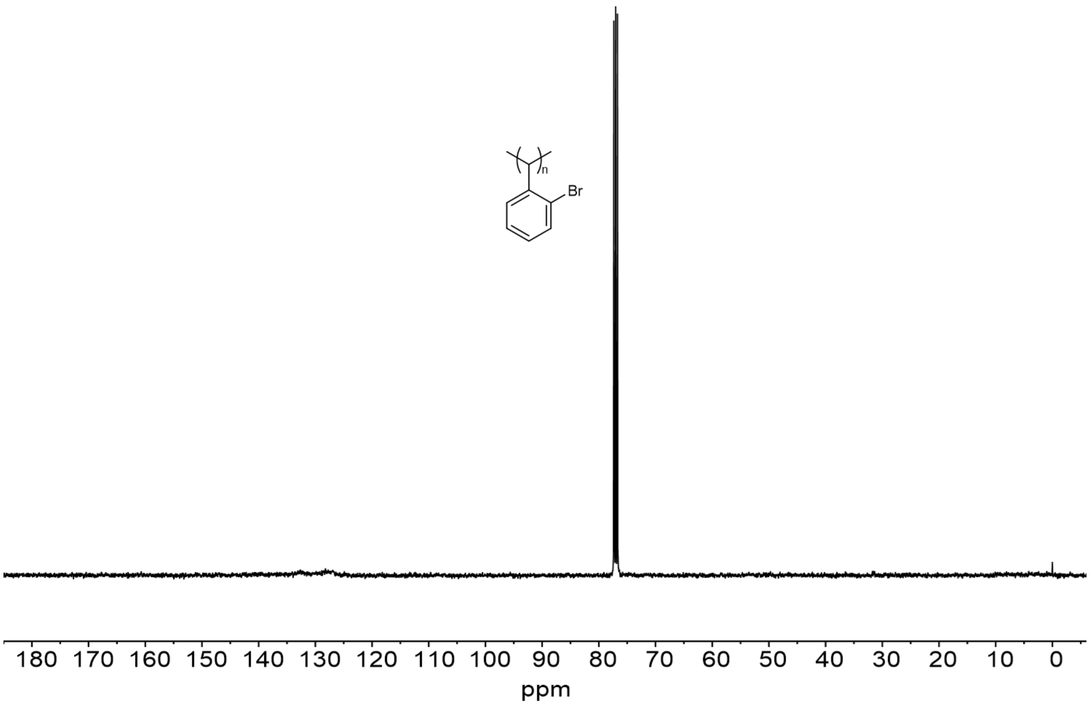

Fig. S120. ^{13}C NMR (100 MHz, $CDCl_3$, 23 ^{o}C) spectrum of **P13**.

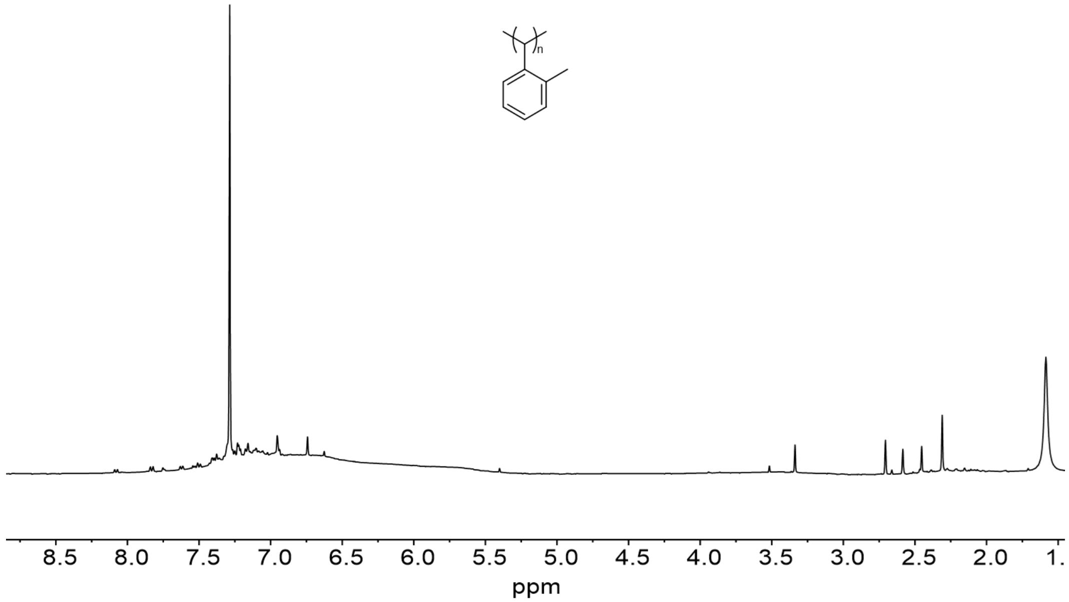

Fig. S121. ^{1}H NMR (400 MHz, $CDCl_3$, 23 °C) spectrum of **P14**.

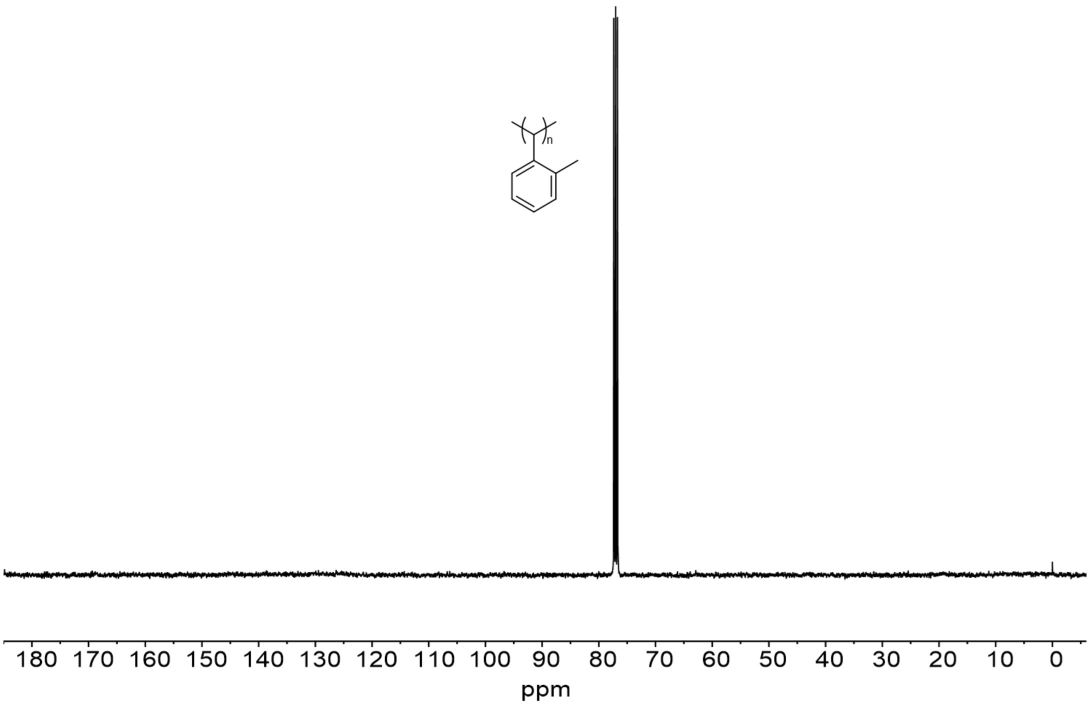

Fig. S122. ^{13}C NMR (100 MHz, $CDCl_3$, 23 °C) spectrum of **P14**.

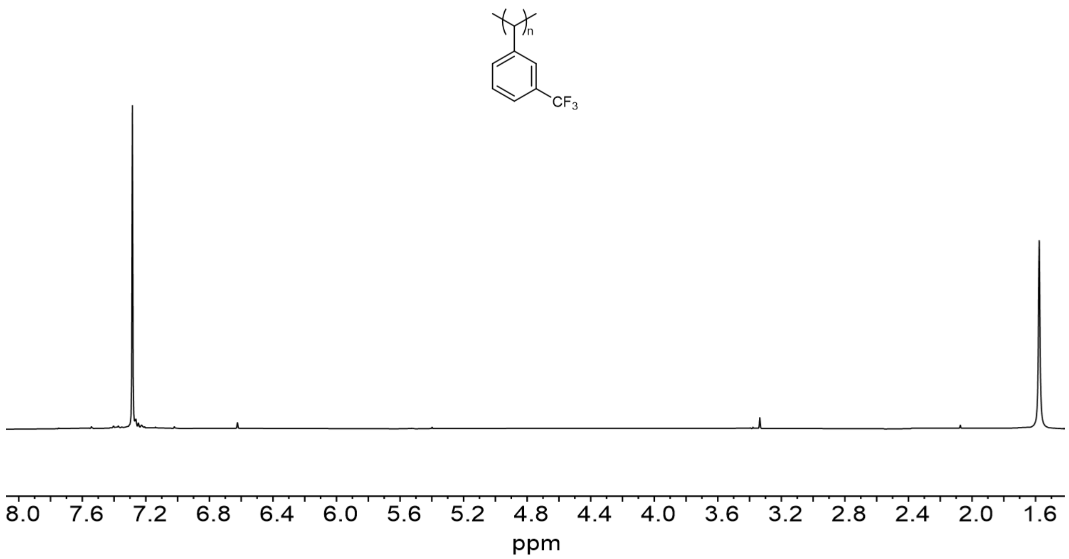

Fig. S123. ^{1}H NMR (400 MHz, $CDCl_3$, 23 ^{o}C) spectrum of **P15** (The absence of a polymer signal is attributed to its poor solubility in the tested solvent).

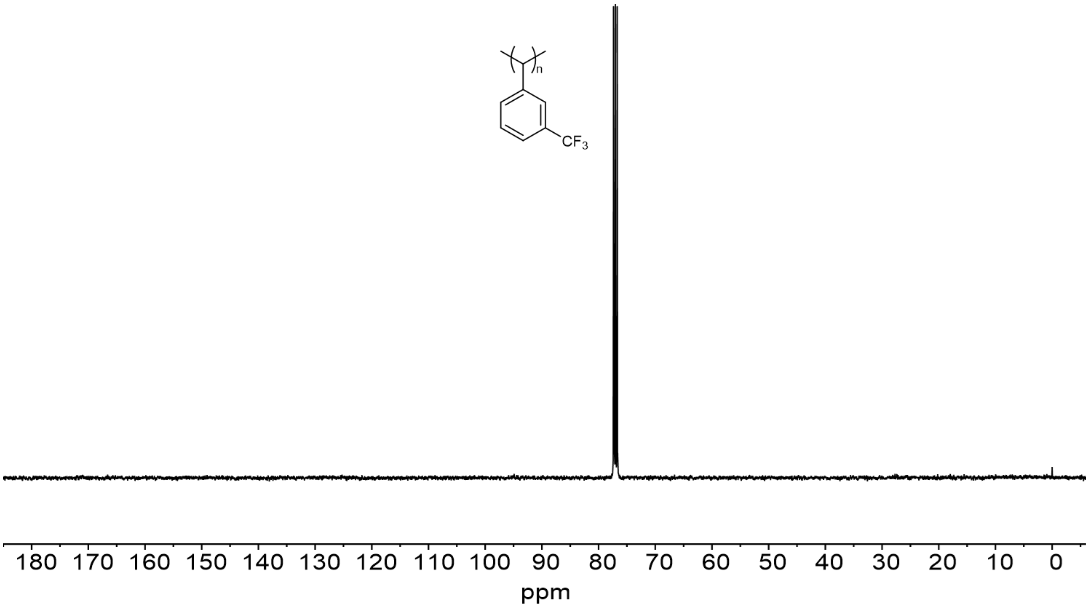

Fig. S124. ^{13}C NMR (100 MHz, $CDCl_3$, 23 ^{o}C) spectrum of **P15** (The absence of a polymer signal is attributed to its poor solubility in the tested solvent).

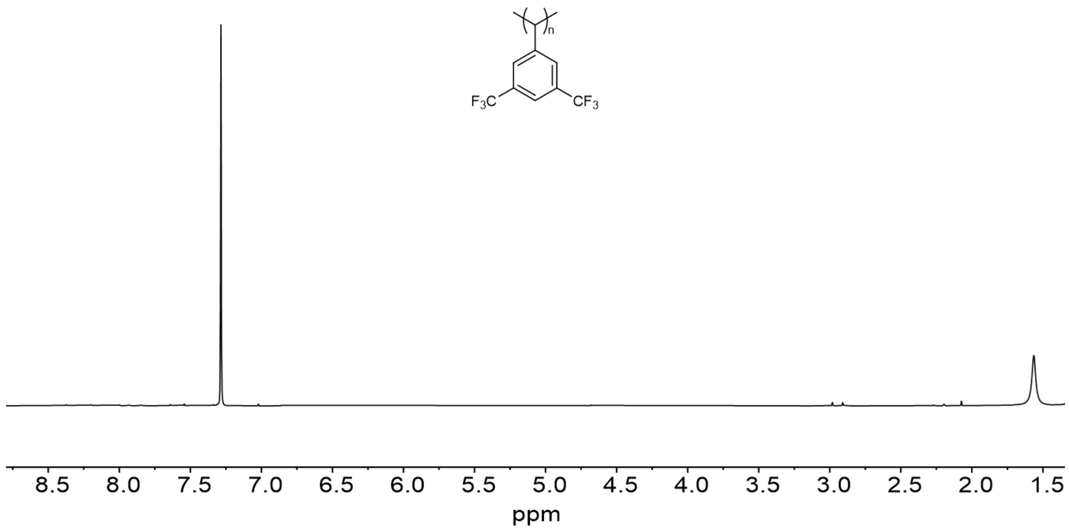

Fig. S125. ^{1}H NMR (400 MHz, $CDCl_3$, 23 ^{o}C) spectrum of **P16** (The absence of a polymer signal is attributed to its poor solubility in the tested solvent).

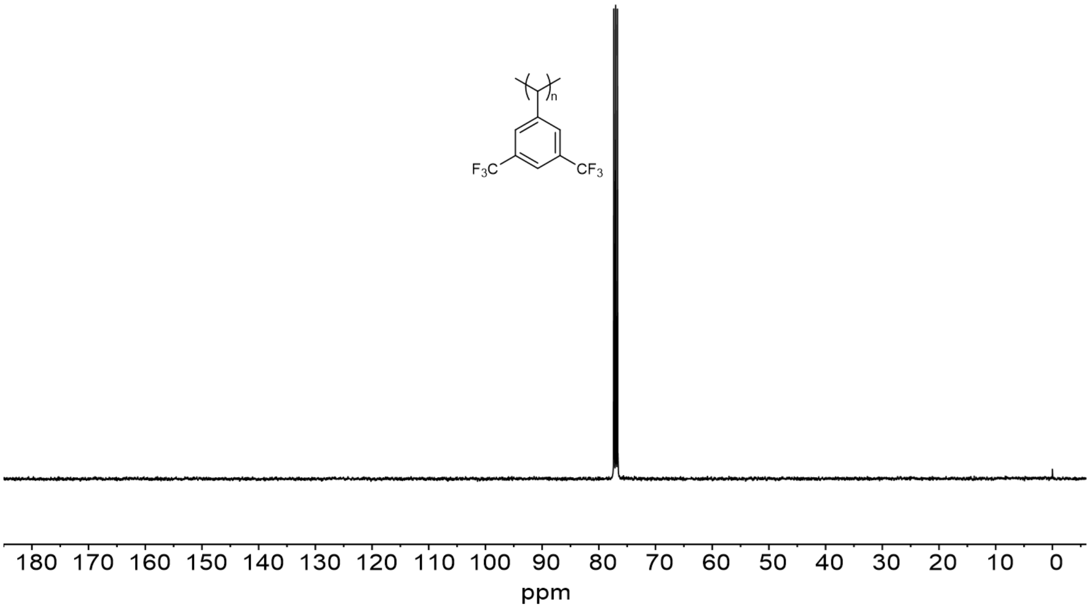

Fig. S126. ^{13}C NMR (100 MHz, $CDCl_3$, 23 ^{o}C) spectrum of **P16** (The absence of a polymer signal is attributed to its poor solubility in the tested solvent).

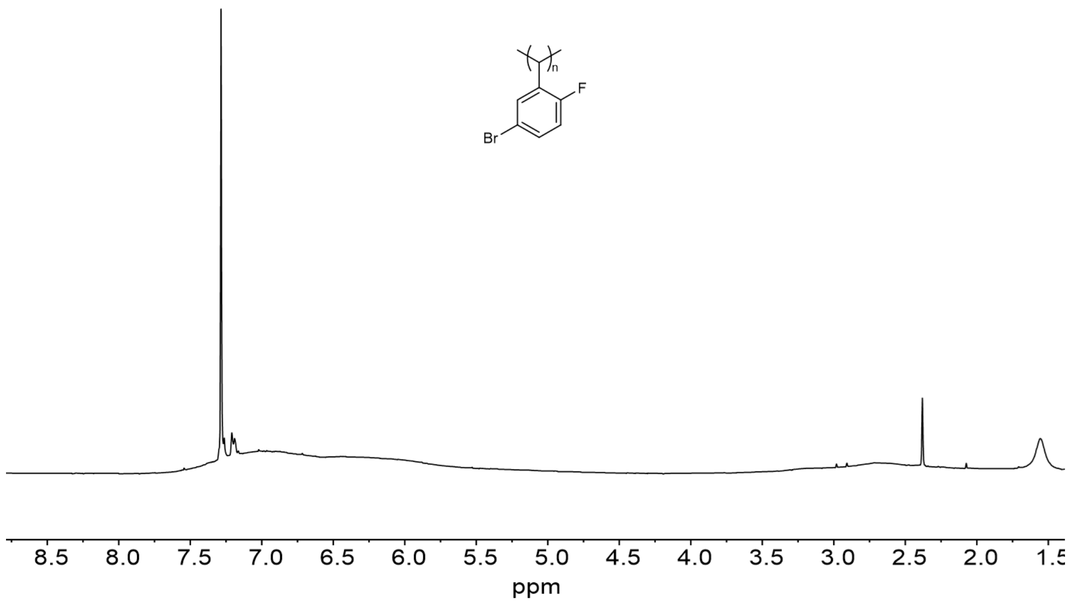

Fig. S127. ^{1}H NMR (400 MHz, $CDCl_3$, 23 °C) spectrum of **P17**.

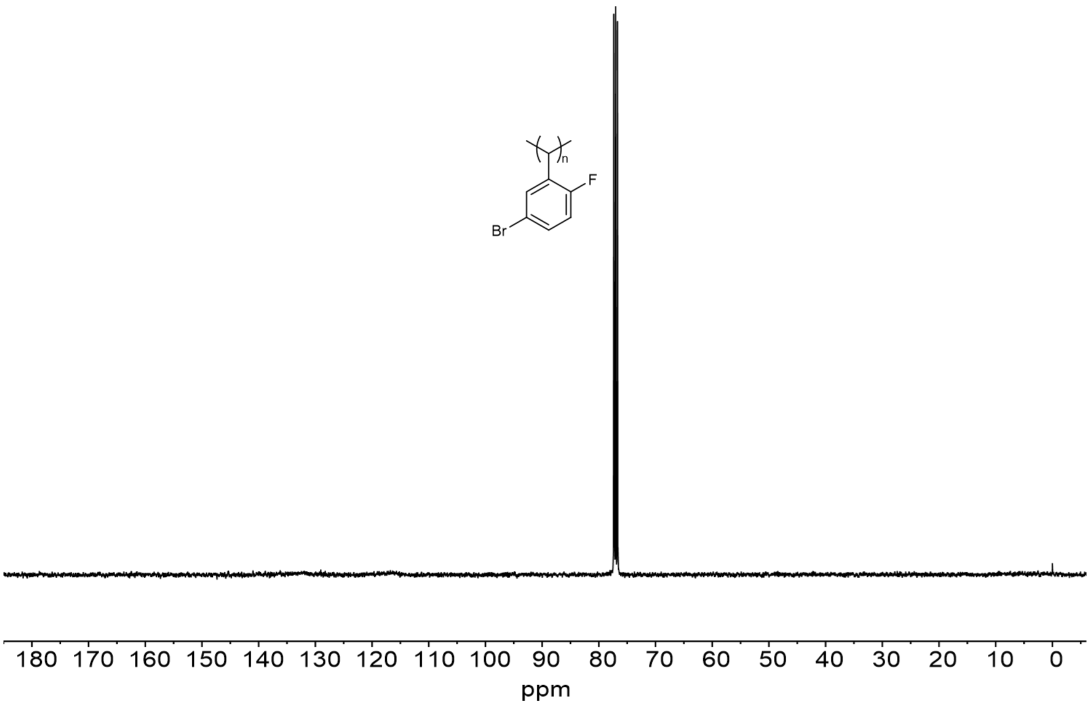

Fig. S128. ^{13}C NMR (100 MHz, $CDCl_3$, 23 °C) spectrum of **P17**.

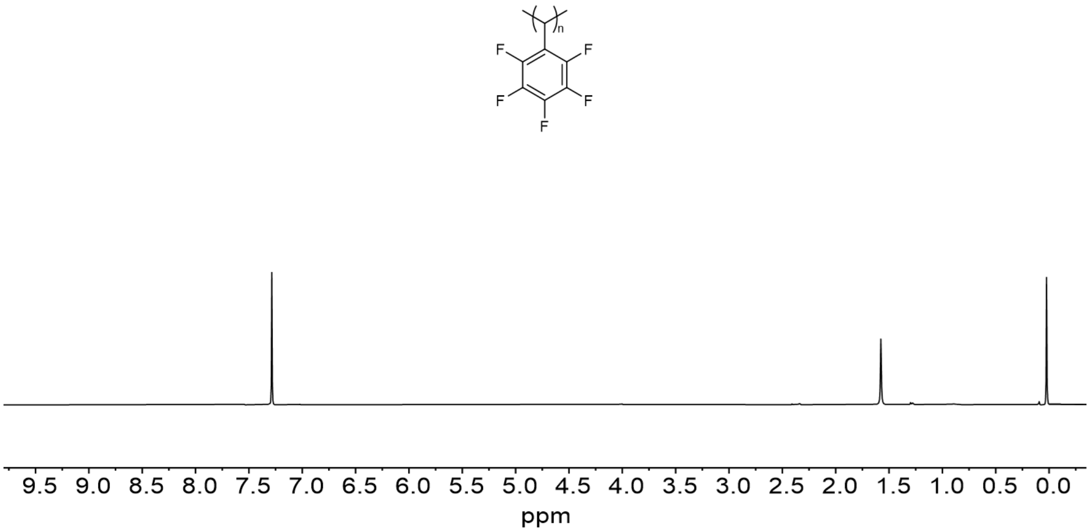

Fig. S129. ^{1}H NMR (400 MHz, $CDCl_3$, 23 ºC) spectrum of **P18** (The absence of a polymer signal is attributed to its poor solubility in the tested solvent).

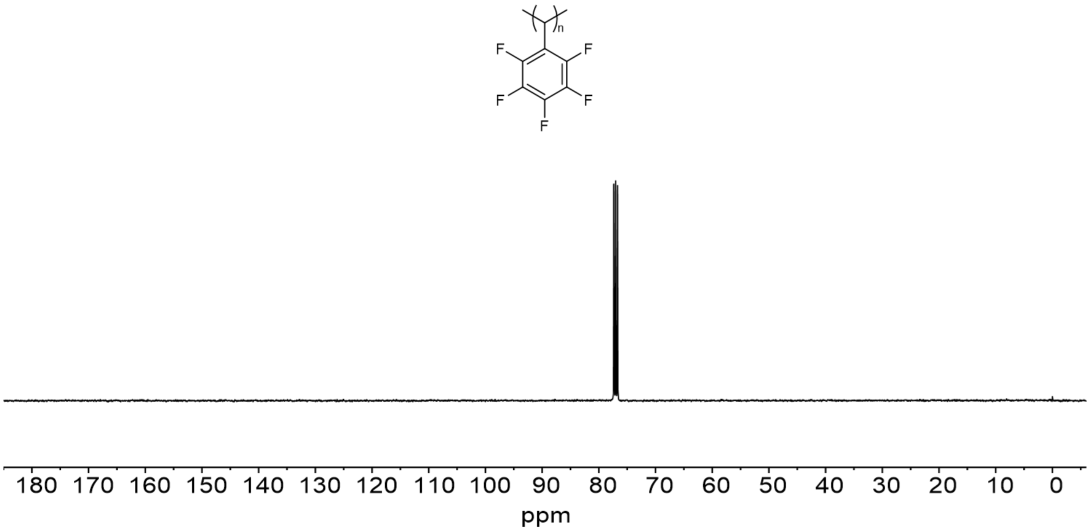

Fig. S130. ^{13}C NMR (100 MHz, $CDCl_3$, 23 ºC) spectrum of **P18** (The absence of a polymer signal is attributed to its poor solubility in the tested solvent).

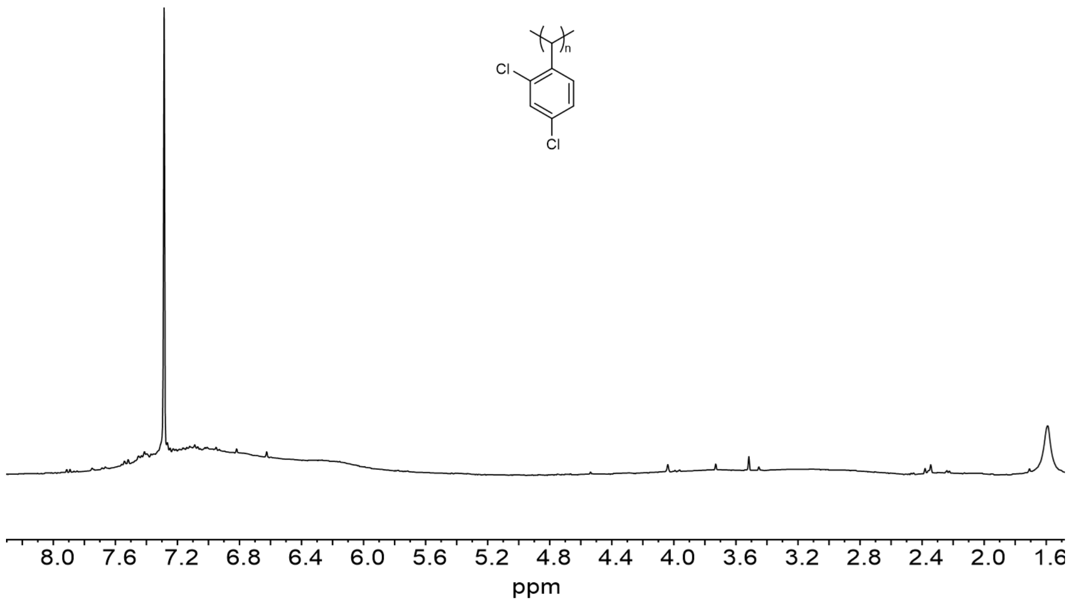

Fig. S131. ^{1}H NMR (400 MHz, $CDCl_3$, 23 °C) spectrum of **P19**.

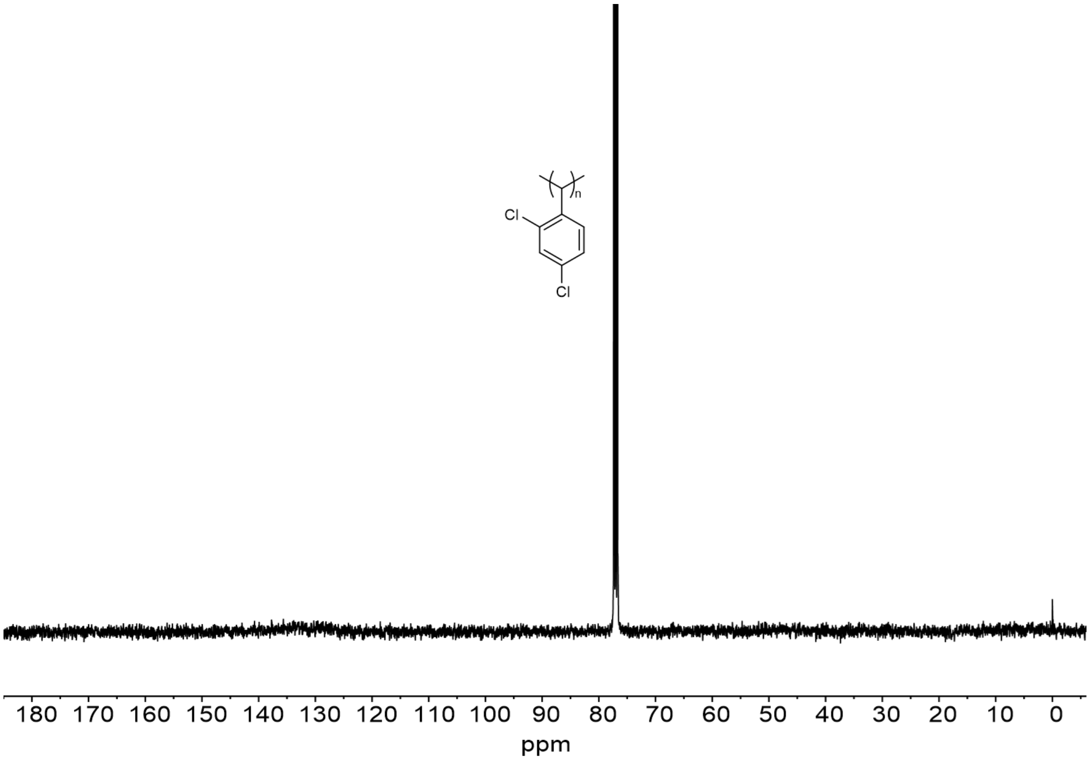

Fig. S132. ^{13}C NMR (100 MHz, $CDCl_3$, 23 °C) spectrum of **P19**.

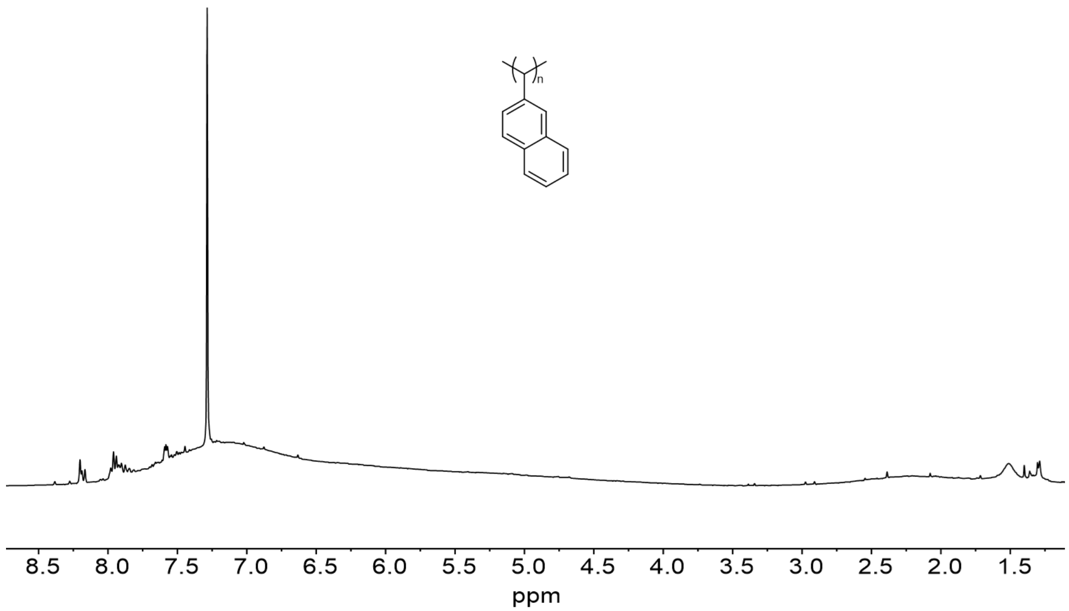

Fig. S133. ^{1}H NMR (400 MHz, $CDCl_3$, 23 °C) spectrum of **P20**.

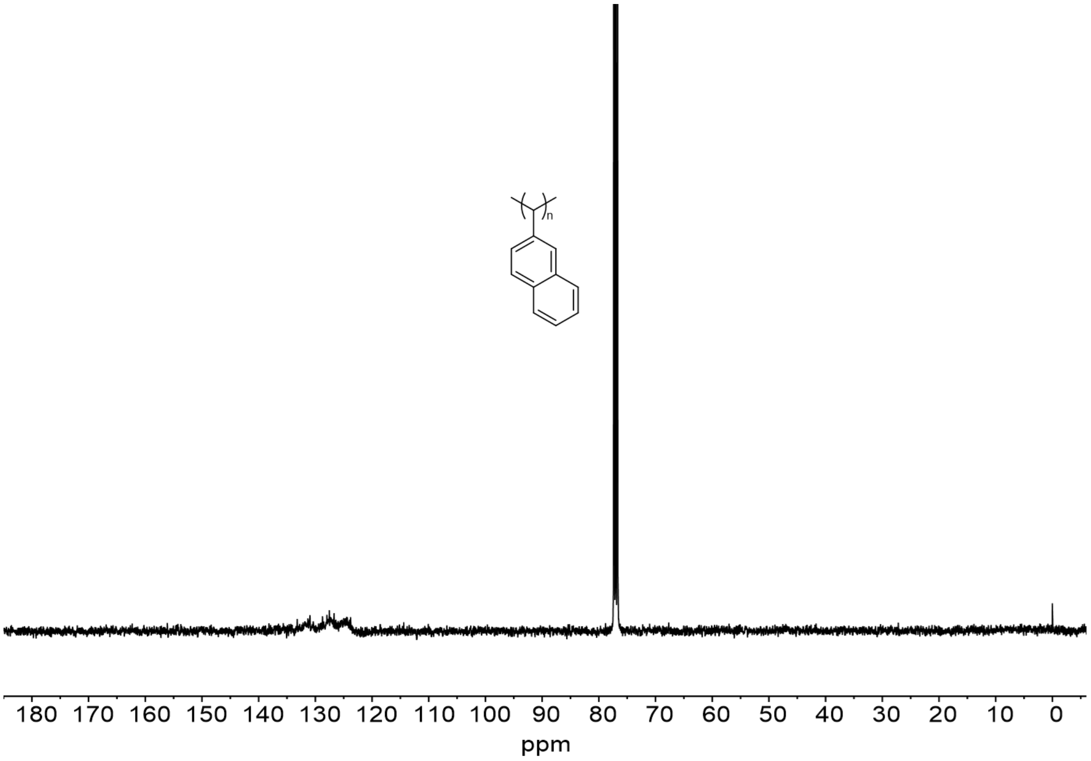

Fig. S134. ^{13}C NMR (100 MHz, $CDCl_3$, 23 °C) spectrum of **P20**.

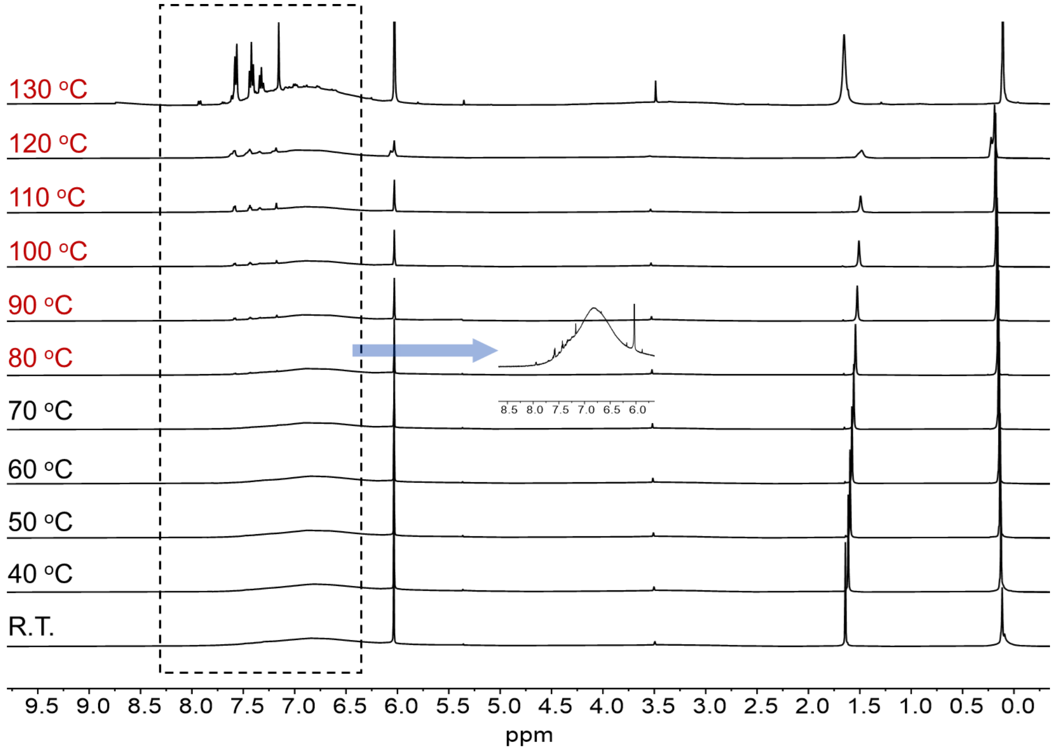

Fig. S135. *In-situ* ^{1}H NMR (600 MHz, TCE-d_2) spectra of **P1** catalyzed by $C(Ph)_3{}^{+}BF_4{}^{-}$, collected during a stepwise temperature increase from R.T. to 130 °C (Thermal decomposition was observed starting at approximately 80 °C, and the decomposition product is *trans*-stilbene).

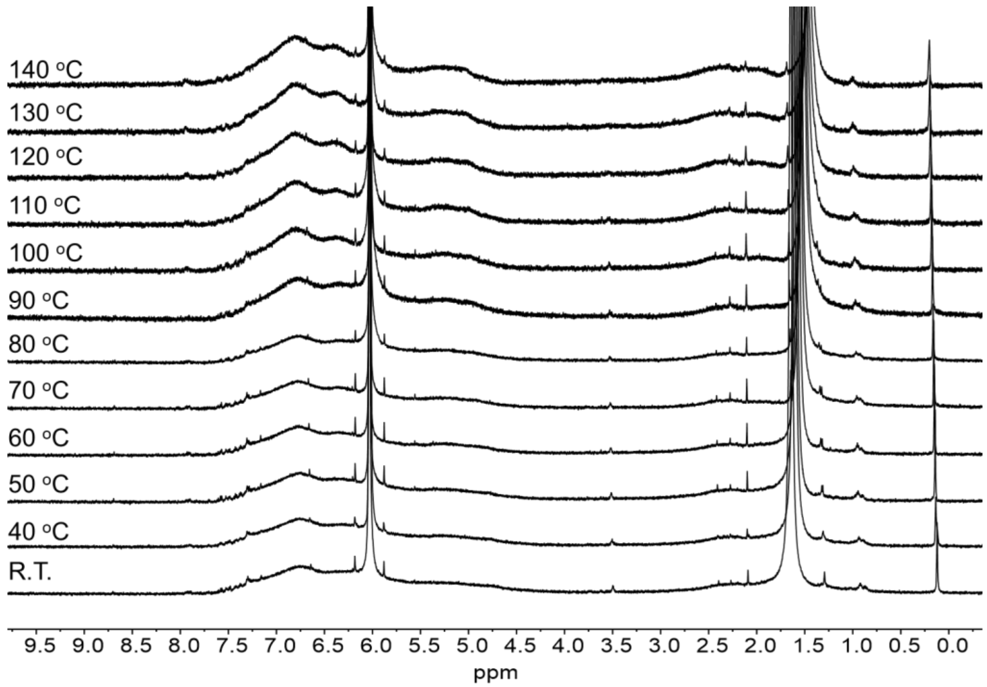

Fig. S136. *In-situ* ^{1}H NMR (600 MHz, TCE-d_2) spectra of **P1** catalyzed by $Ni(acac)_2$, collected during a stepwise temperature increase from R.T. to 140 °C (No thermal decomposition was observed until 140 °C).

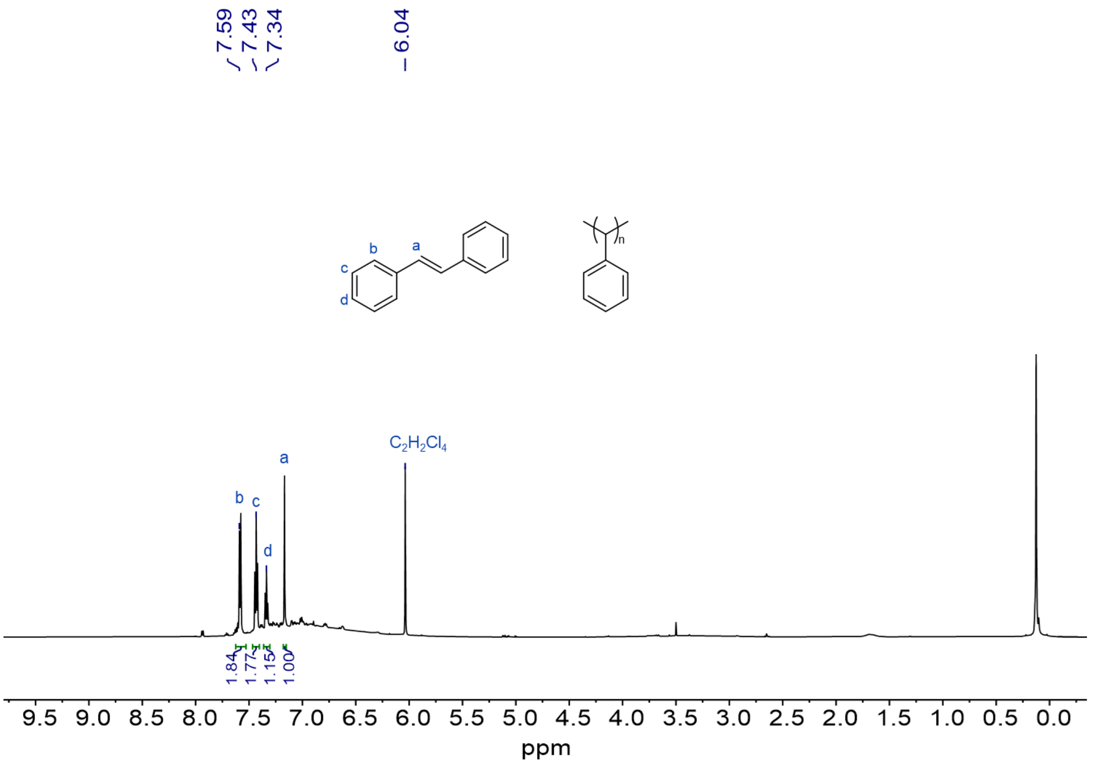

Fig. S137. ^{1}H NMR (600 MHz, TCE-d_2, 23 ºC) spectrum of **P1** catalyzed by $C(Ph)_3{}^+BF_4{}^-$ after heating (The decomposition product is *trans*-stilbene).

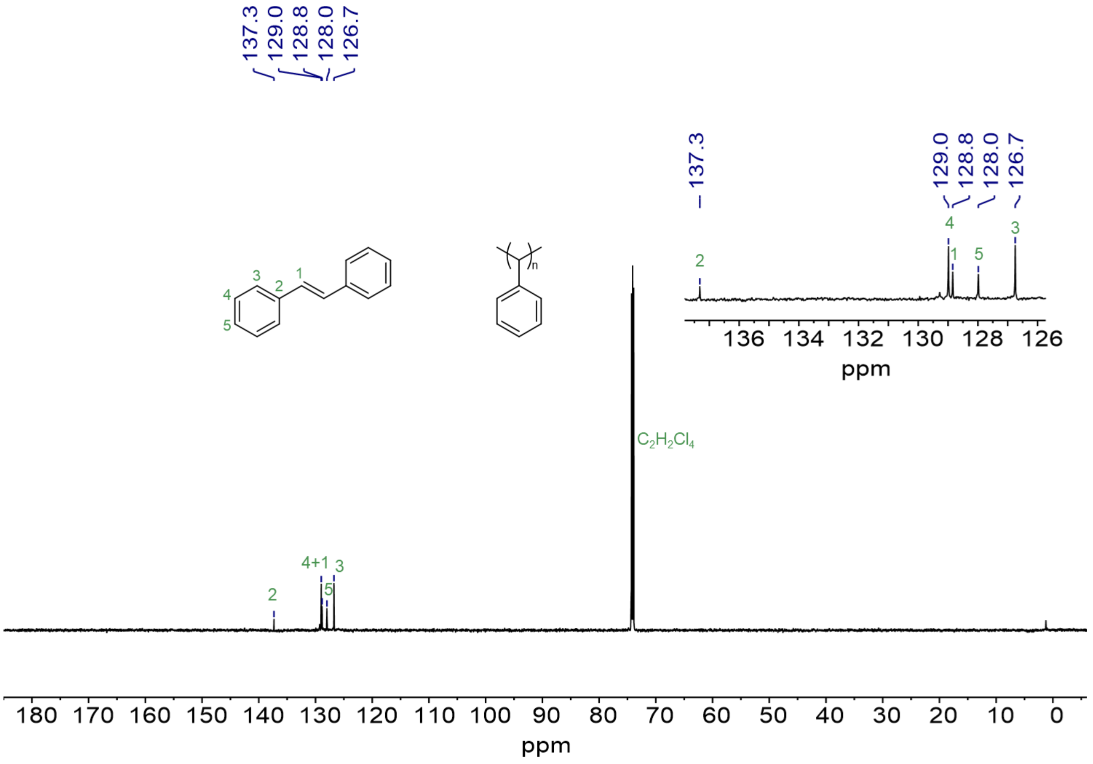

Fig. S138. ^{13}C NMR (150 MHz, TCE-d_2, 130 ºC) spectrum of **P1** catalyzed by $C(Ph)_3{}^+BF_4{}^-$ after heating (The decomposition product is *trans*-stilbene).

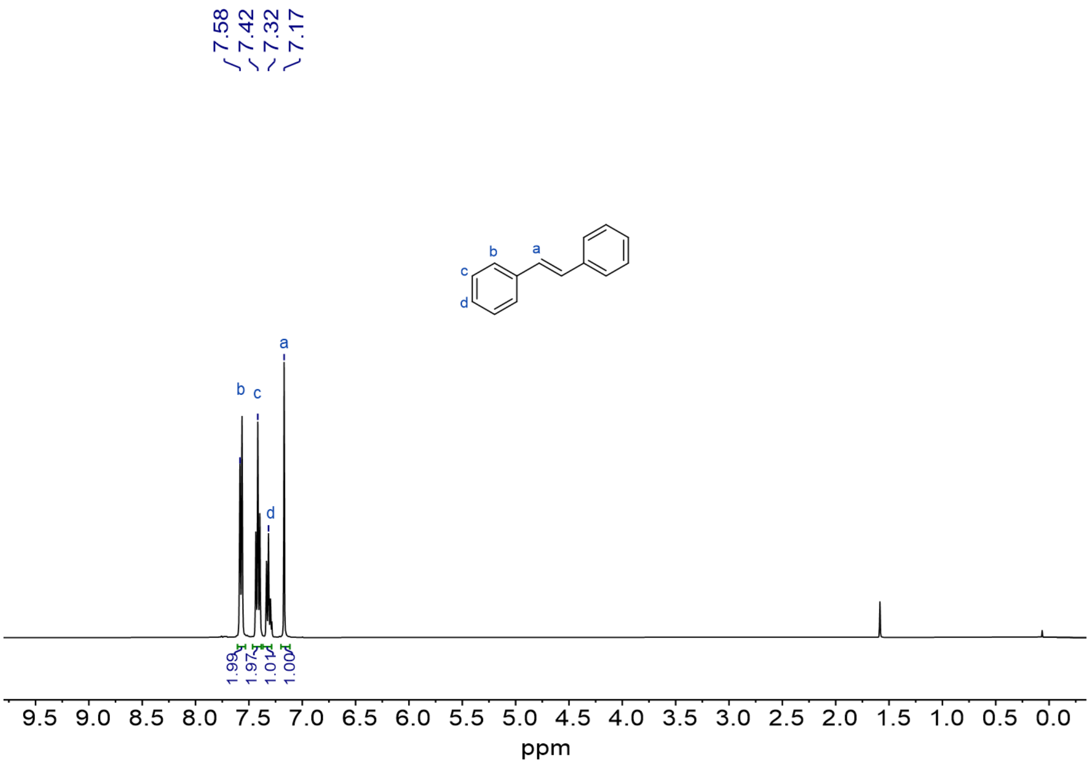

Fig. S139. ^{1}H NMR (400 MHz, $CDCl_3$, 23 ºC) spectrum of *trans*-stilbene.

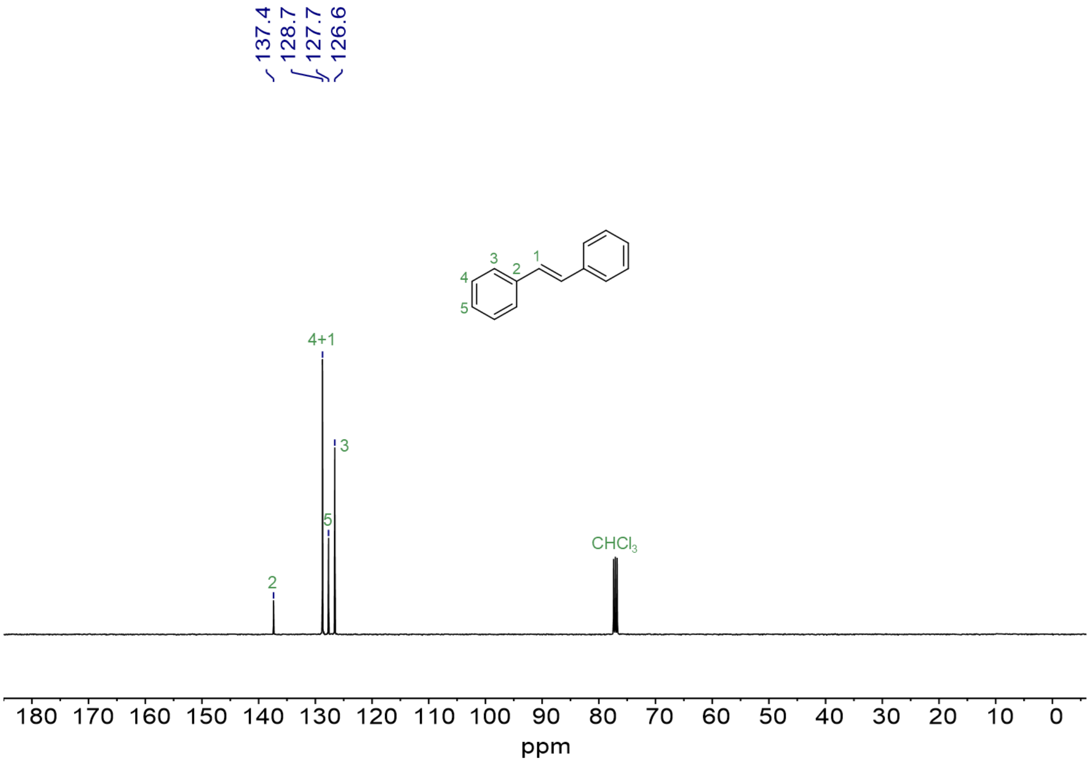

Fig. S140. ^{13}C NMR (100 MHz, $CDCl_3$, 23 ºC) spectrum of *trans*-stilbene.

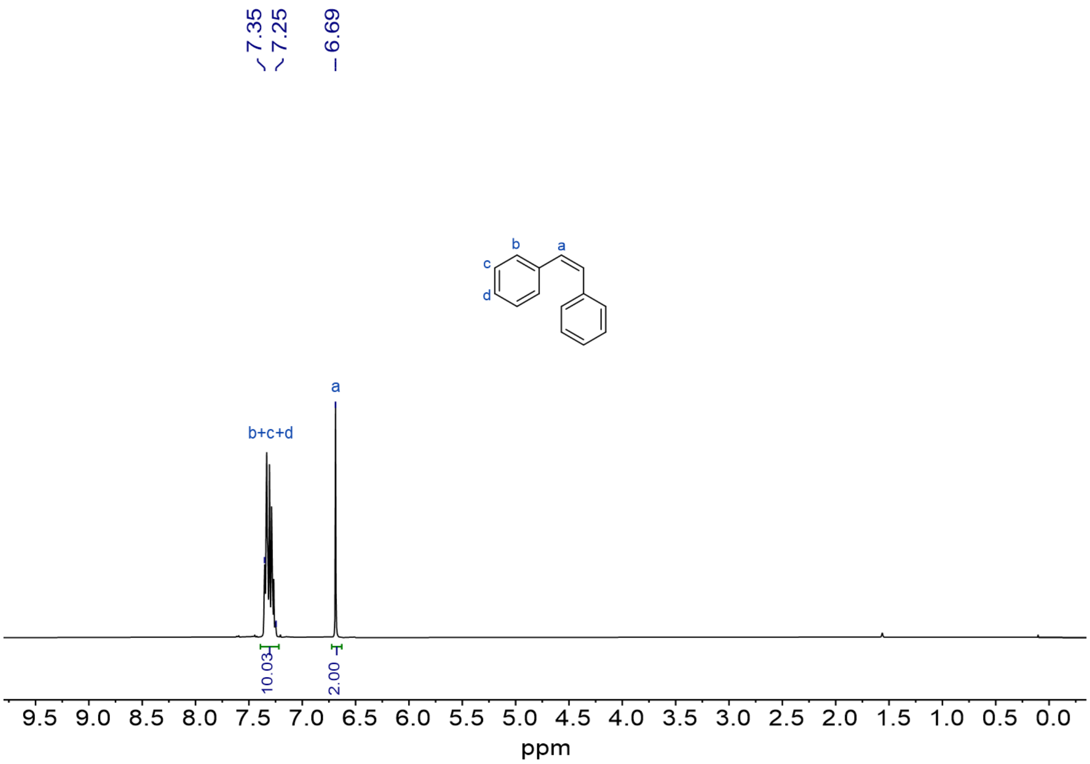

Fig. S141. ^{1}H NMR (400 MHz, $CDCl_3$, 23 ºC) spectrum of *cis*-stilbene.

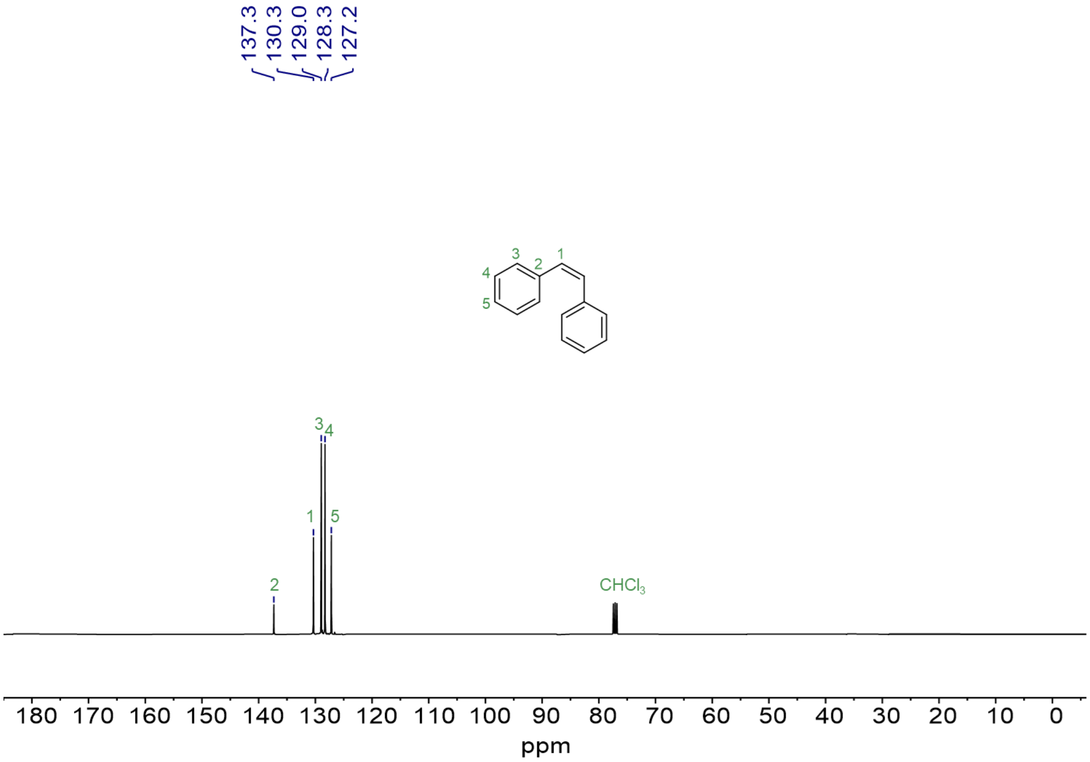

Fig. S142. ^{13}C NMR (100 MHz, $CDCl_3$, 23 ºC) spectrum of *cis*-stilbene.

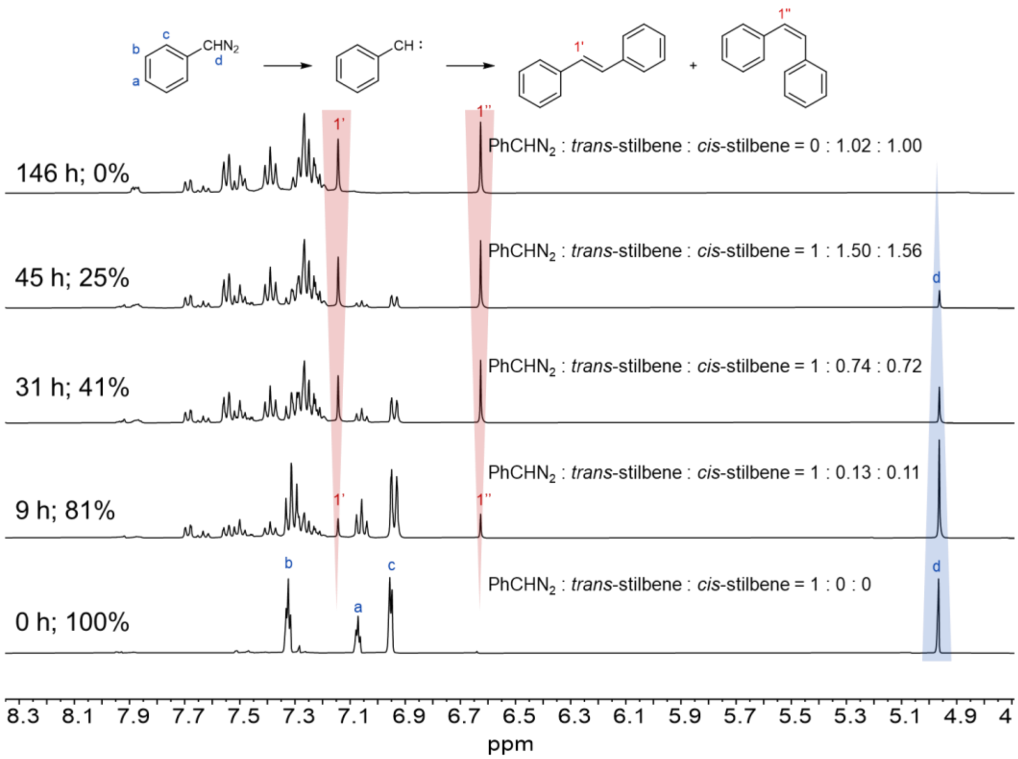

Fig. S143. ^{1}H NMR (400 MHz, $CDCl_3$, 23 ºC) spectra of monomer **1** recorded at different time intervals at room temperature.

FTIR spectra

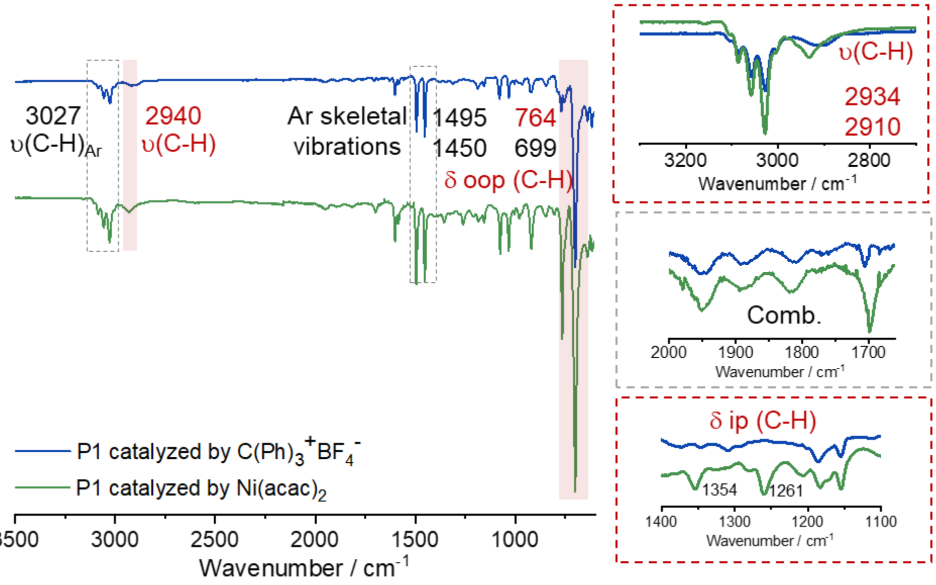

Fig. S144. FTIR spectra of polymer **P1** synthesized using $C(Ph)_3^+BF_4^-$ (blue) and $Ni(acac)_2$ (green) as catalysts, recorded at room temperature.

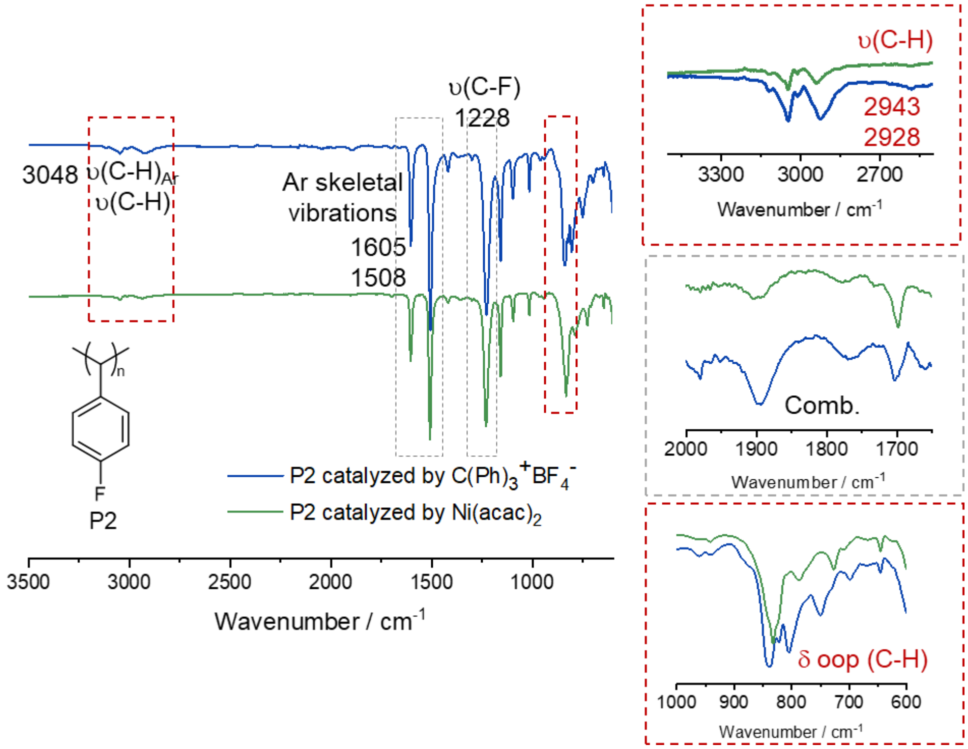

Fig. S145. FTIR spectra of polymer **P2** synthesized using $C(Ph)_3^+BF_4^-$ (blue) and $Ni(acac)_2$ (green) as catalysts, recorded at room temperature.

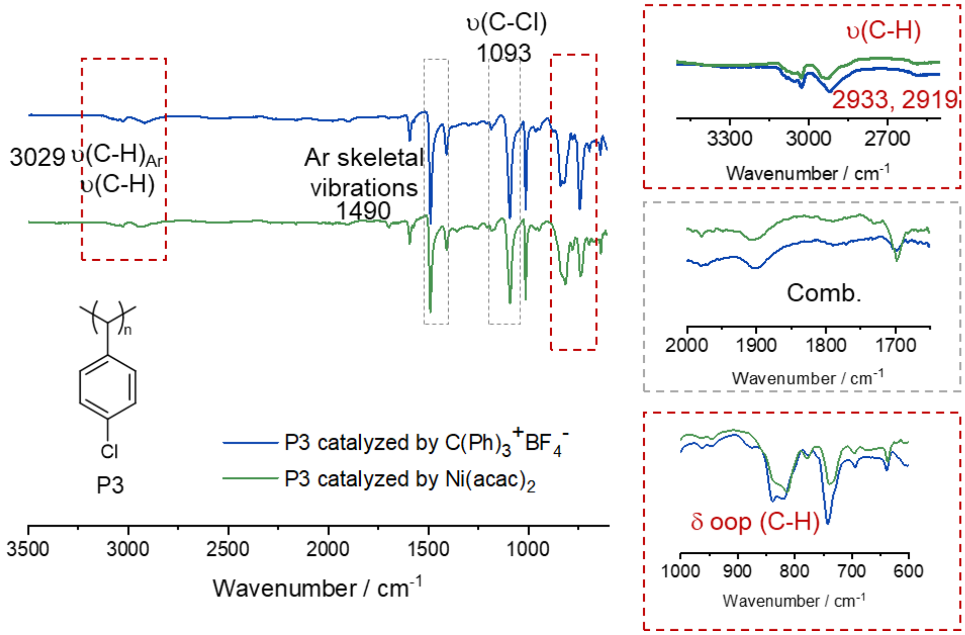

Fig. S146. FTIR spectra of polymer **P3** synthesized using $C(Ph)_3^+BF_4^-$ (blue) and $Ni(acac)_2$ (green) as catalysts, recorded at room temperature.

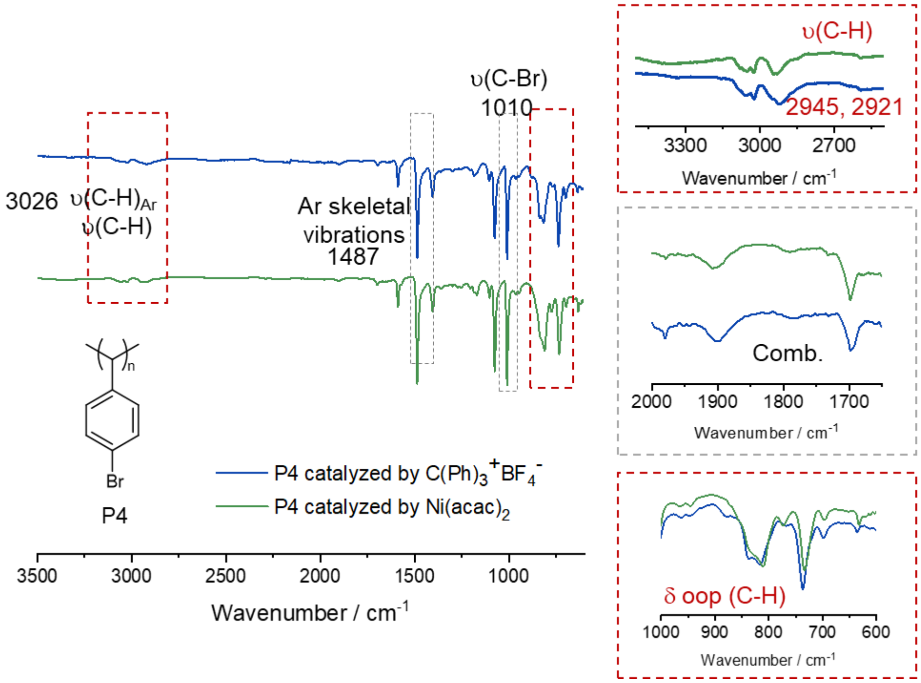

Fig. S147. FTIR spectra of polymer **P4** synthesized using $C(Ph)_3^+BF_4^-$ (blue) and $Ni(acac)_2$ (green) as catalysts, recorded at room temperature.

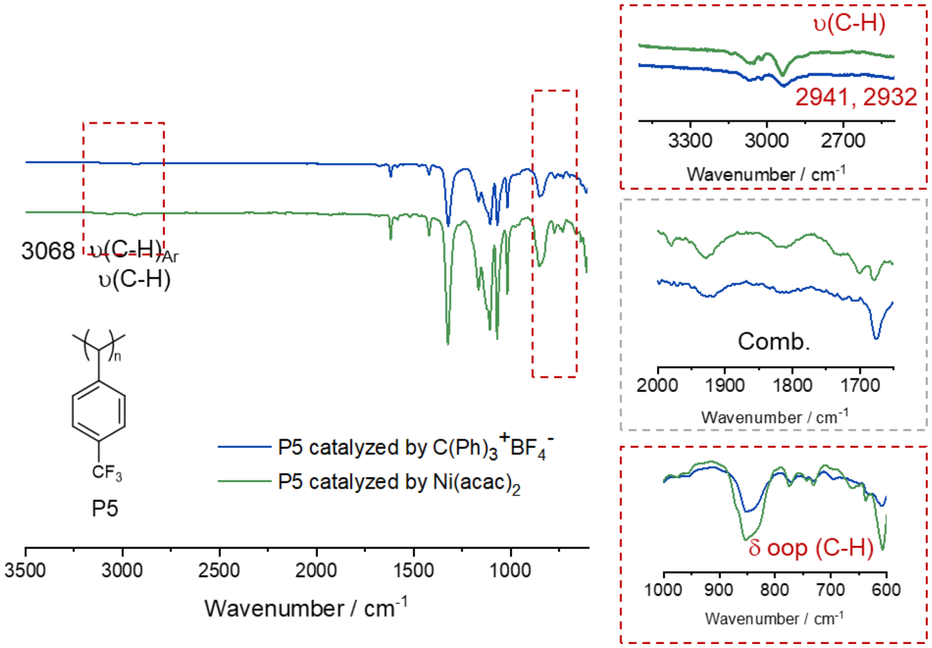

Fig. S148. FTIR spectra of polymer **P5** synthesized using $C(Ph)_3^+BF_4^-$ (blue) and $Ni(acac)_2$ (green) as catalysts, recorded at room temperature.

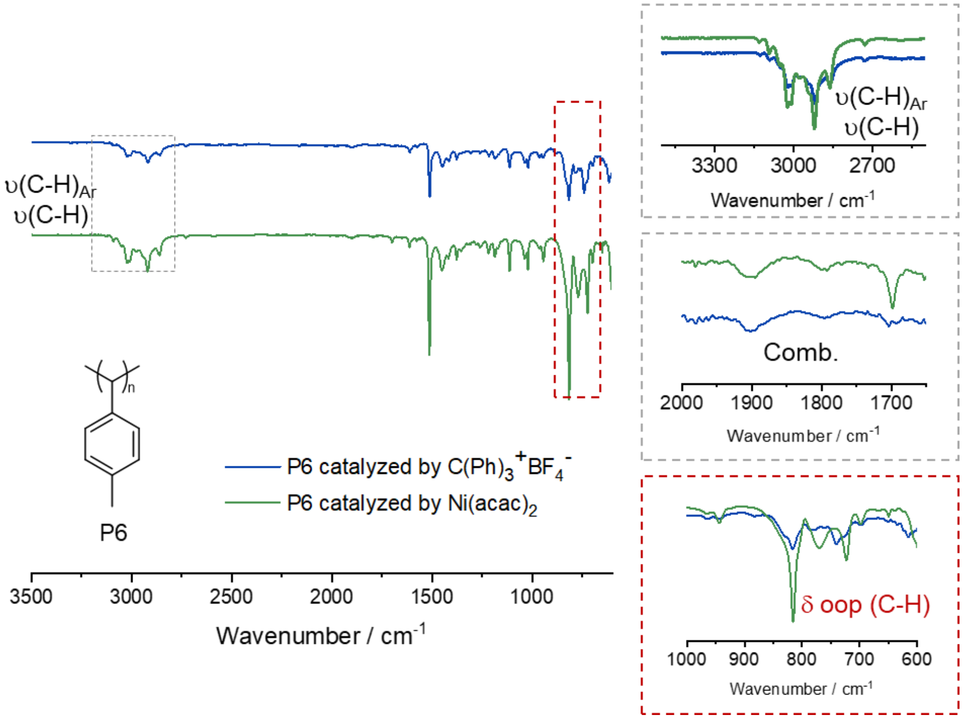

Fig. S149. FTIR spectra of polymer **P6** synthesized using $C(Ph)_3^+BF_4^-$ (blue) and $Ni(acac)_2$ (green) as catalysts, recorded at room temperature.

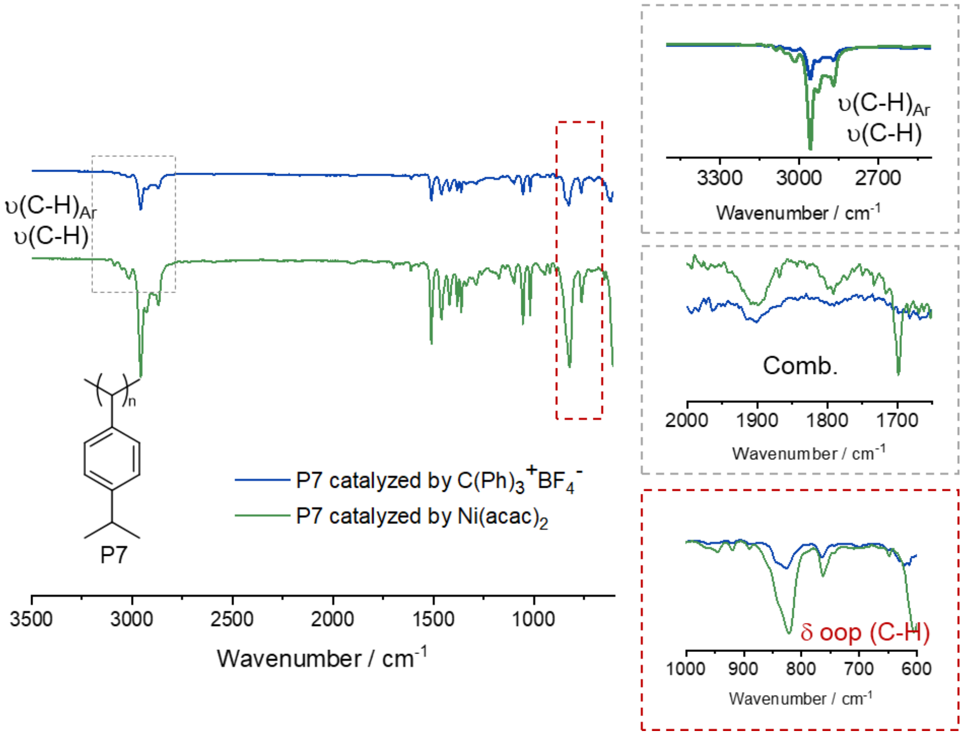

Fig. S150. FTIR spectra of polymer **P7** synthesized using $C(Ph)_3^+BF_4^-$ (blue) and $Ni(acac)_2$ (green) as catalysts, recorded at room temperature.

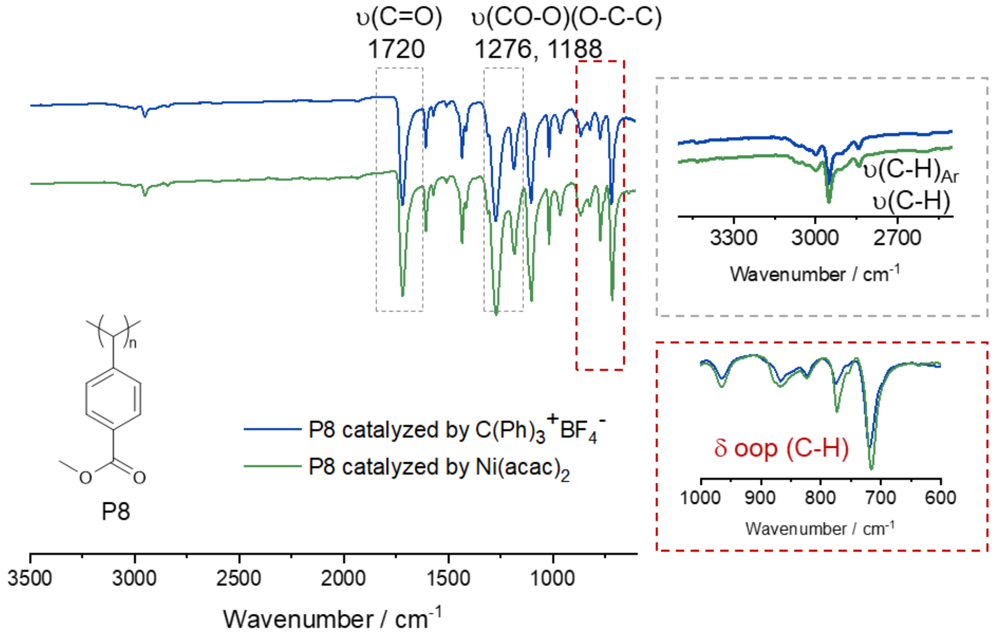

Fig. S151. FTIR spectra of polymer **P8** synthesized using $C(Ph)_3^+BF_4^-$ (blue) and $Ni(acac)_2$ (green) as catalysts, recorded at room temperature.

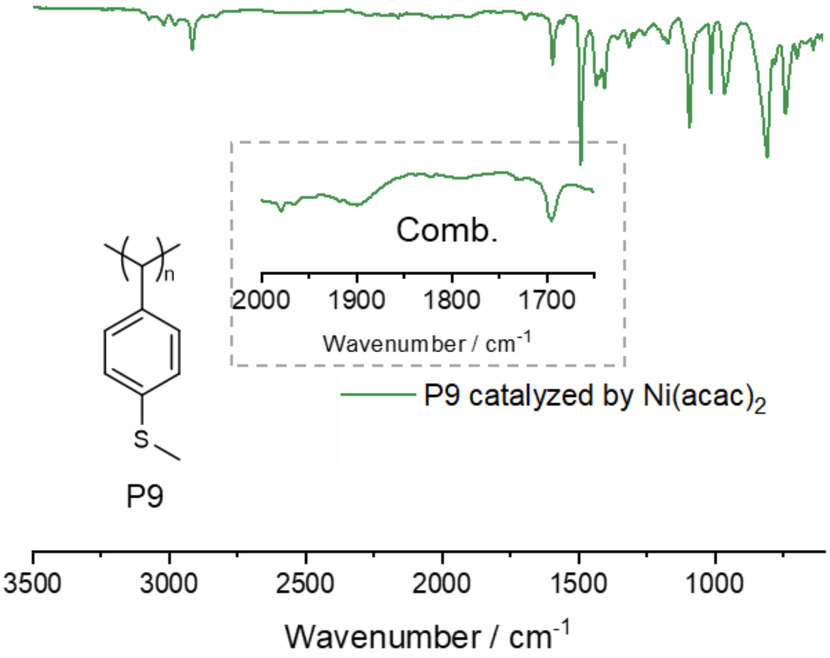

Fig. S152. FTIR spectrum of polymer **P9** synthesized using $Ni(acac)_2$ as a catalyst, recorded at room temperature.

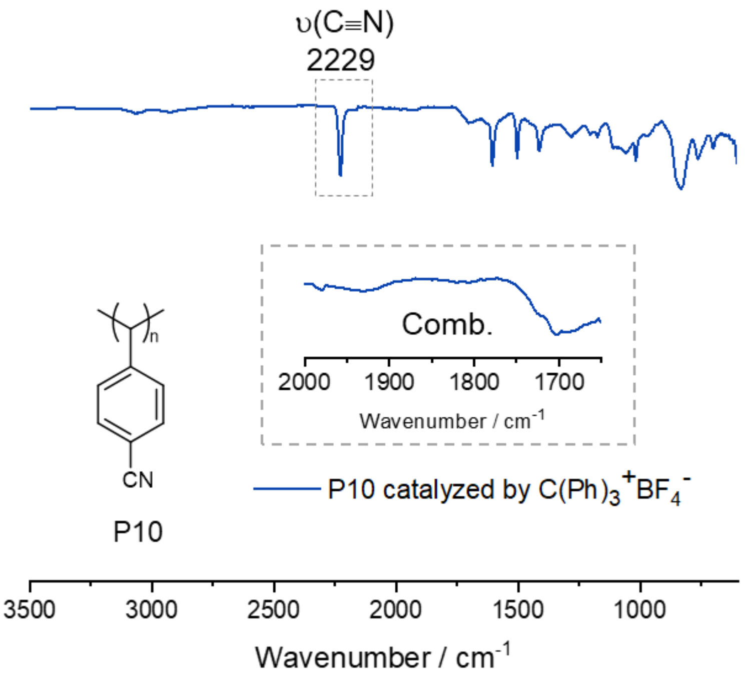

Fig. S153. FTIR spectrum of polymer **P10** synthesized using $C(Ph)_3^+BF_4^-$ as a catalyst, recorded at room temperature.

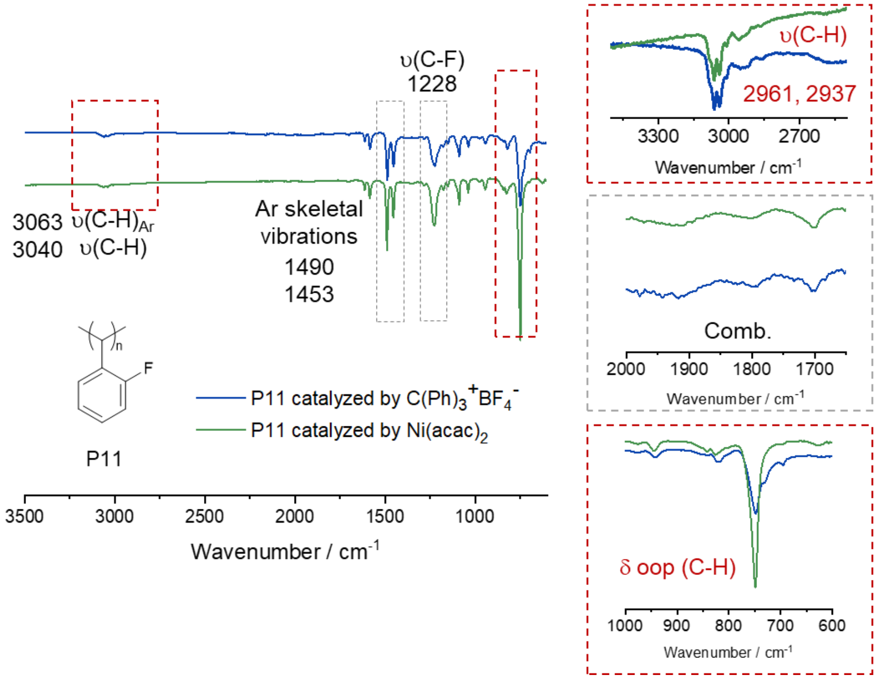

Fig. S154. FTIR spectra of polymer **P11** synthesized using $C(Ph)_3^+BF_4^-$ (blue) and $Ni(acac)_2$ (green) as catalysts, recorded at room temperature.

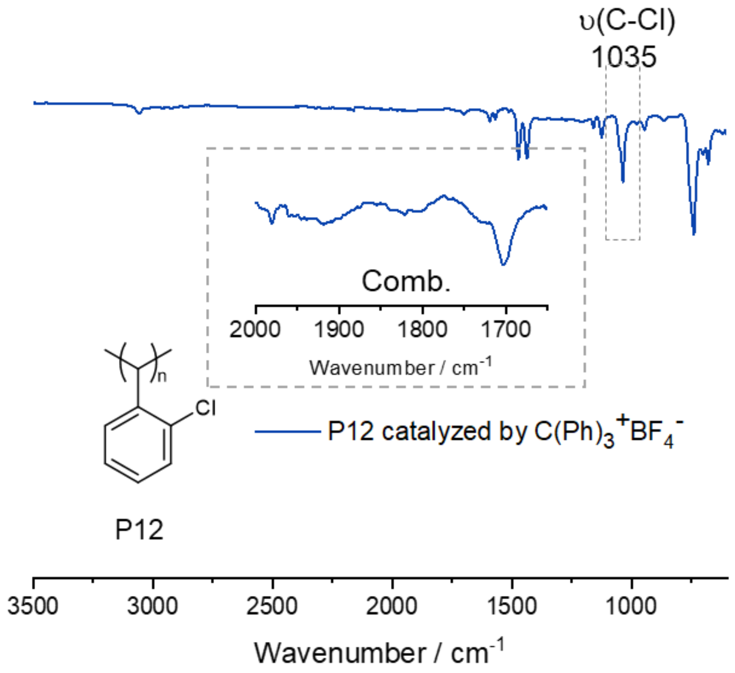

Fig. S155. FTIR spectrum of polymer **P12** synthesized using $C(Ph)_3^+BF_4^-$ as a catalyst, recorded at room temperature.

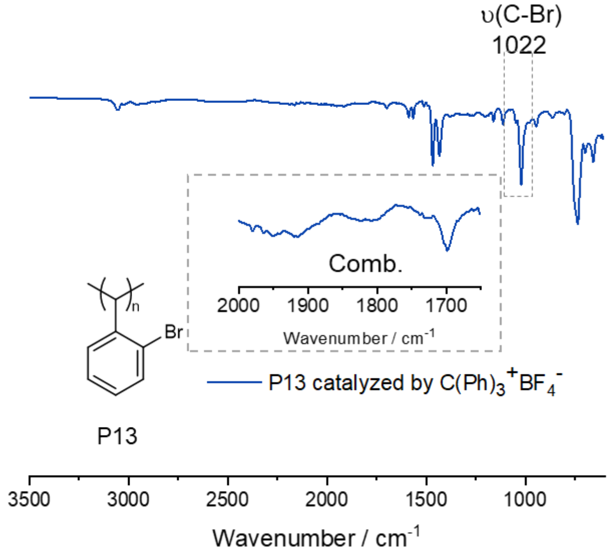

Fig. S156. FTIR spectrum of polymer **P13** synthesized using $C(Ph)_3^+BF_4^-$ as a catalyst, recorded at room temperature.

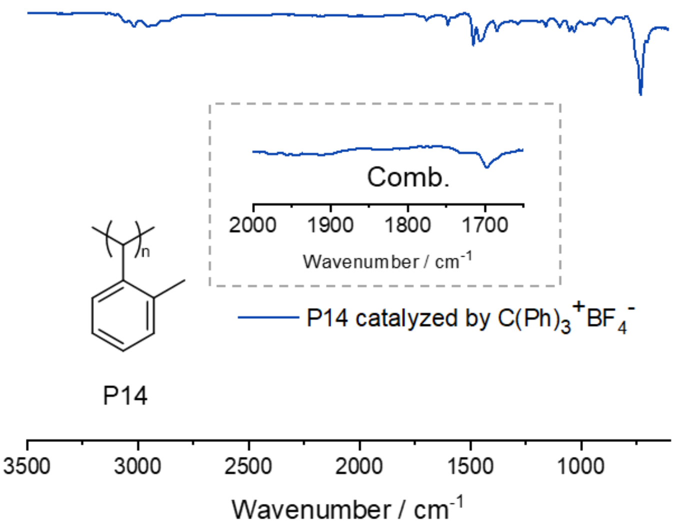

Fig. S157. FTIR spectrum of polymer **P14** synthesized using $C(Ph)_3^+BF_4^-$ as a catalyst, recorded at room temperature.

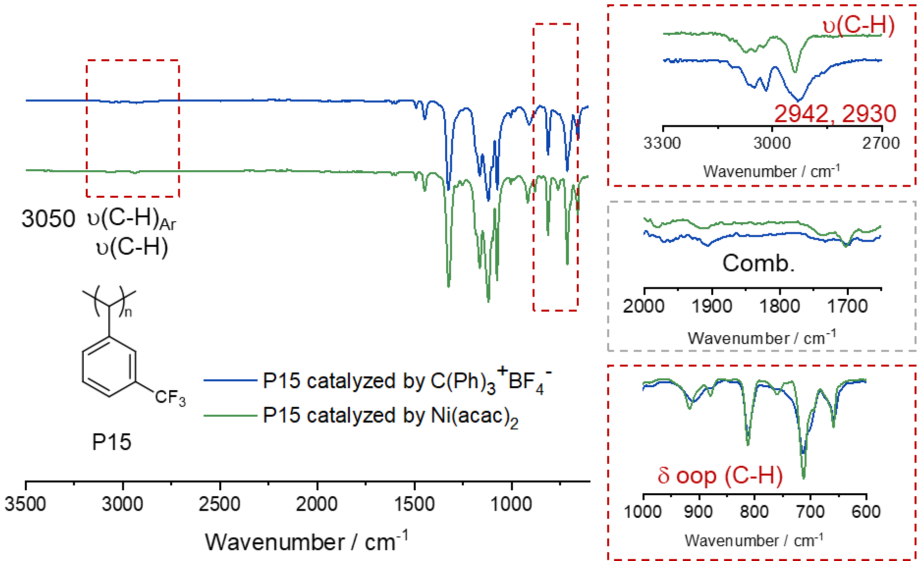

Fig. S158. FTIR spectra of polymer **P15** synthesized using $C(Ph)_3^+BF_4^-$ (blue) and $Ni(acac)_2$ (green) as catalysts, recorded at room temperature.

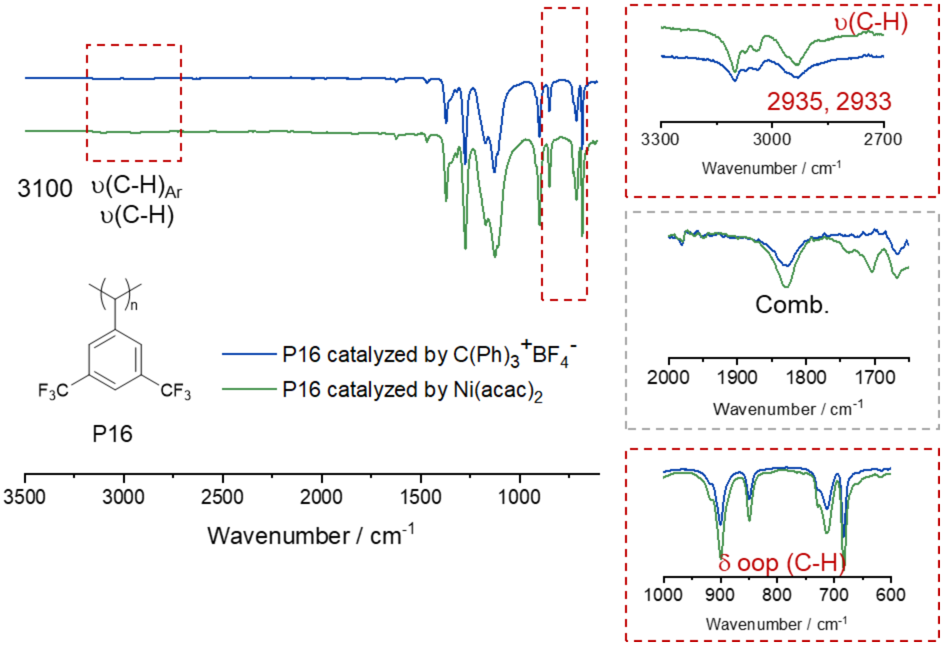

Fig. S159. FTIR spectra of polymer **P16** synthesized using $C(Ph)_3^+BF_4^-$ (blue) and $Ni(acac)_2$ (green) as catalysts, recorded at room temperature.

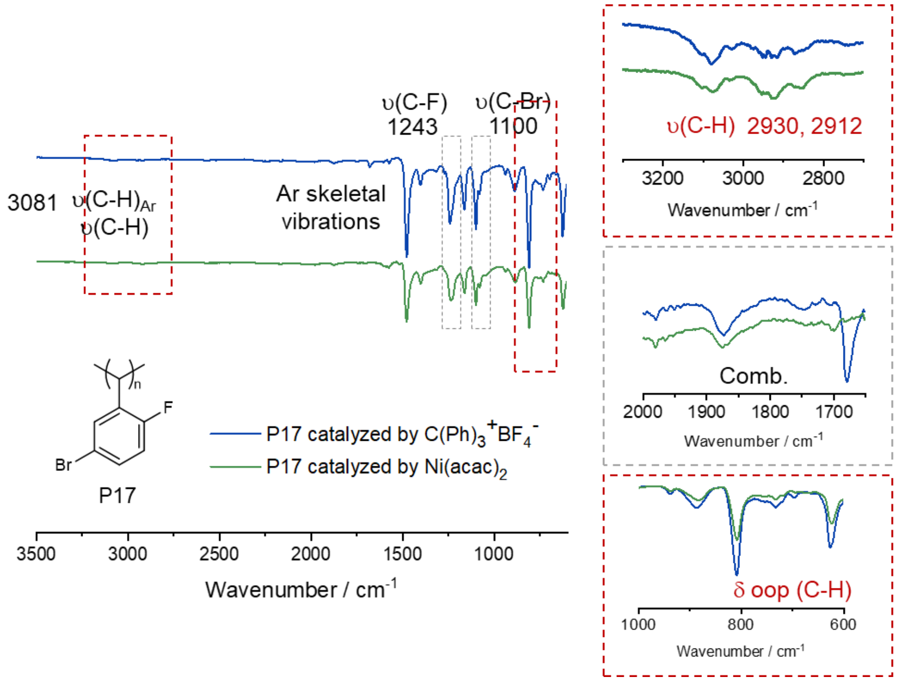

Fig. S160. FTIR spectra of polymer **P17** synthesized using $C(Ph)_3^+BF_4^-$ (blue) and $Ni(acac)_2$ (green) as catalysts, recorded at room temperature.

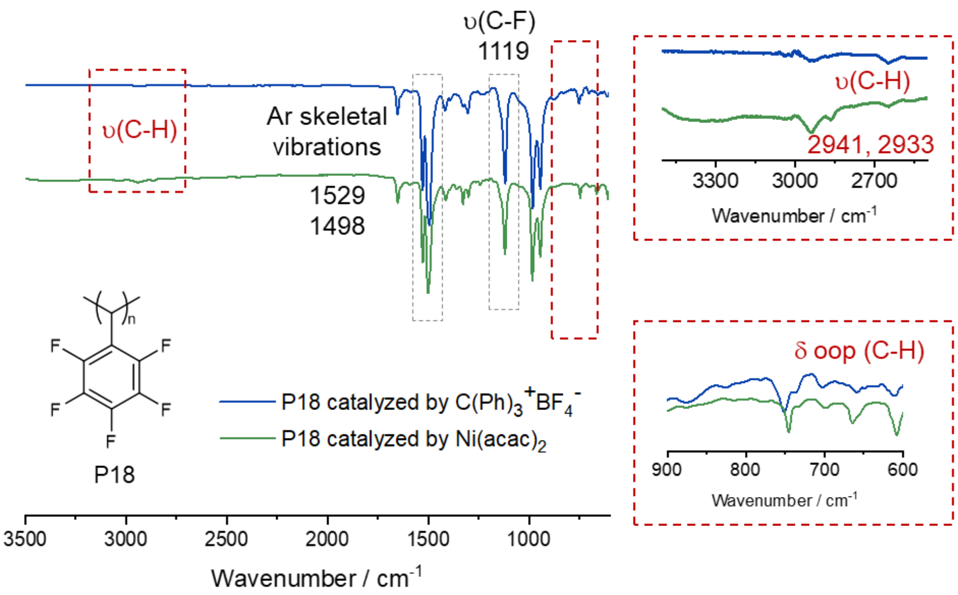

Fig. S161. FTIR spectra of polymer **P18** synthesized using $C(Ph)_3^+BF_4^-$ (blue) and $Ni(acac)_2$ (green) as catalysts, recorded at room temperature.

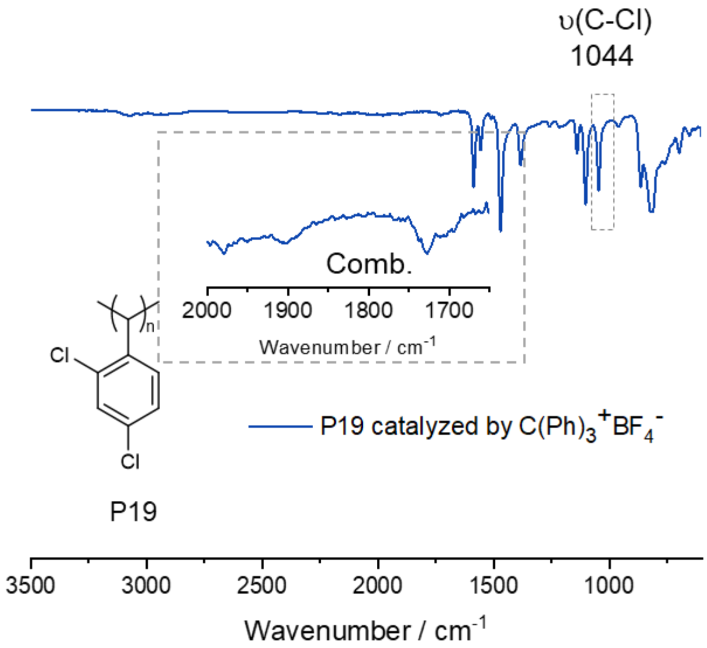

Fig. S162. FTIR spectrum of polymer **P19** synthesized using $C(Ph)_3^+BF_4^-$ as a catalyst, recorded at room temperature.

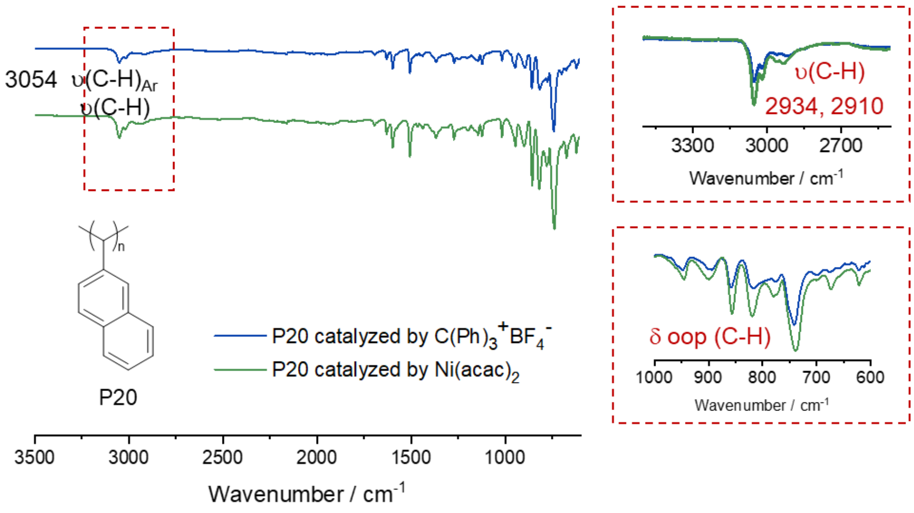

Fig. S163. FTIR spectra of polymer **P20** synthesized using $C(Ph)_3^+BF_4^-$ (blue) and $Ni(acac)_2$ (green) as catalysts, recorded at room temperature.

Raman spectra

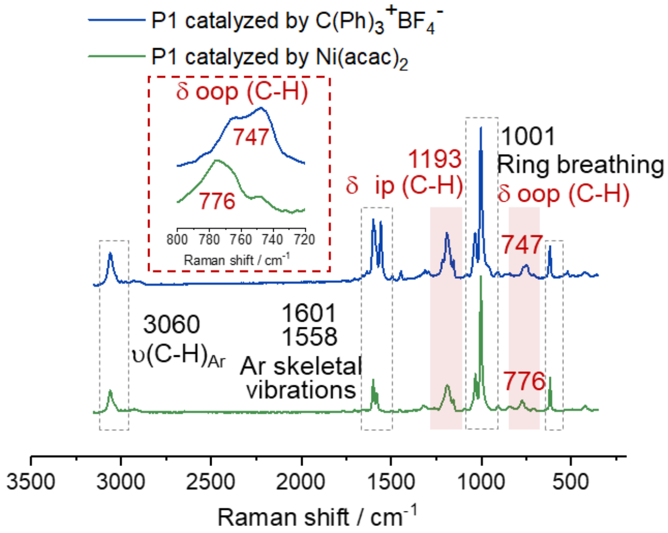

Fig. S164. Raman spectra of polymer **P1** synthesized using $C(Ph)_3^+BF_4^-$ (blue) and $Ni(acac)_2$ (green) as catalysts, recorded at room temperature with a 633 nm excitation wavelength.

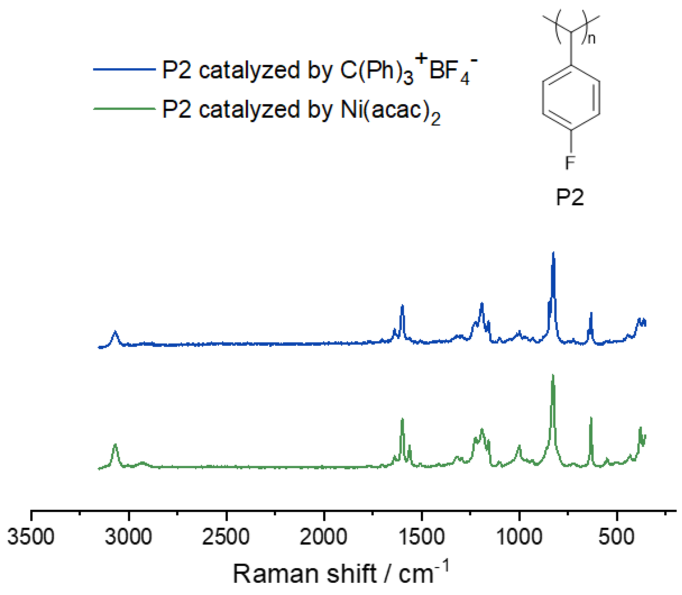

Fig. S165. Raman spectra of polymer **P2** synthesized using $C(Ph)_3^+BF_4^-$ (blue) and $Ni(acac)_2$ (green) as catalysts, recorded at room temperature with a 633 nm excitation wavelength.

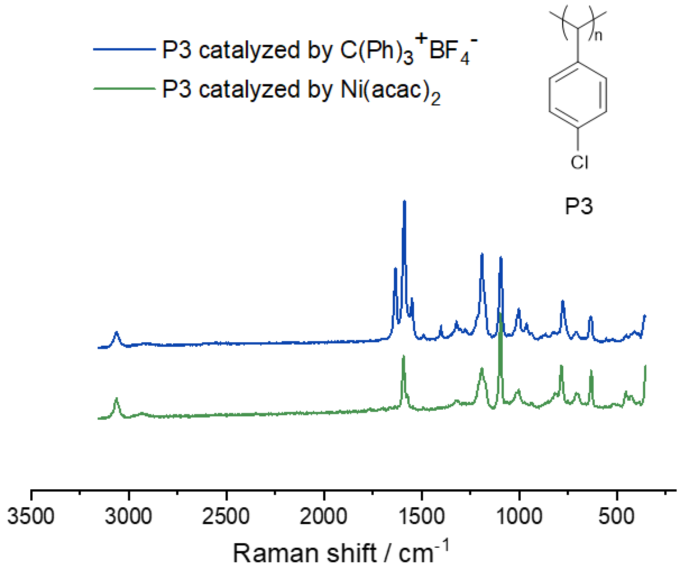

Fig. S166. Raman spectra of polymer **P3** synthesized using $C(Ph)_3^+BF_4^-$ (blue) and $Ni(acac)_2$ (green) as catalysts, recorded at room temperature with a 633 nm excitation wavelength.

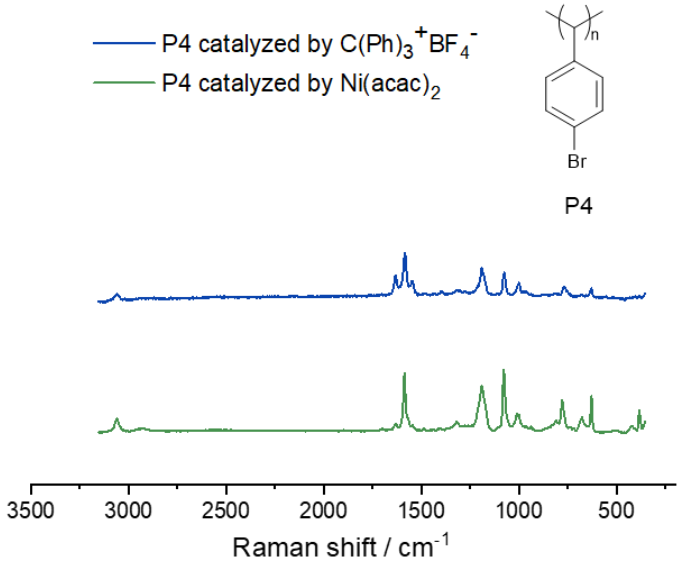

Fig. S167. Raman spectra of polymer **P4** synthesized using $C(Ph)_3^+BF_4^-$ (blue) and $Ni(acac)_2$ (green) as catalysts, recorded at room temperature with a 633 nm excitation wavelength.

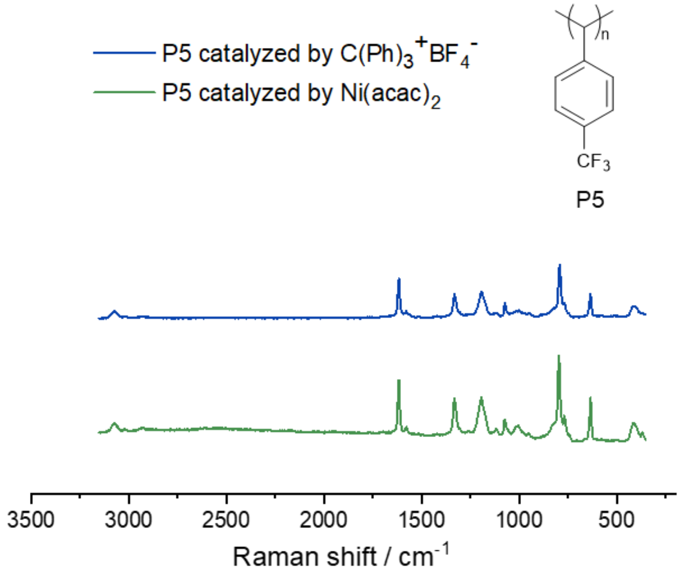

Fig. S168. Raman spectra of polymer **P5** synthesized using $C(Ph)_3^+BF_4^-$ (blue) and $Ni(acac)_2$ (green) as catalysts, recorded at room temperature with a 633 nm excitation wavelength.

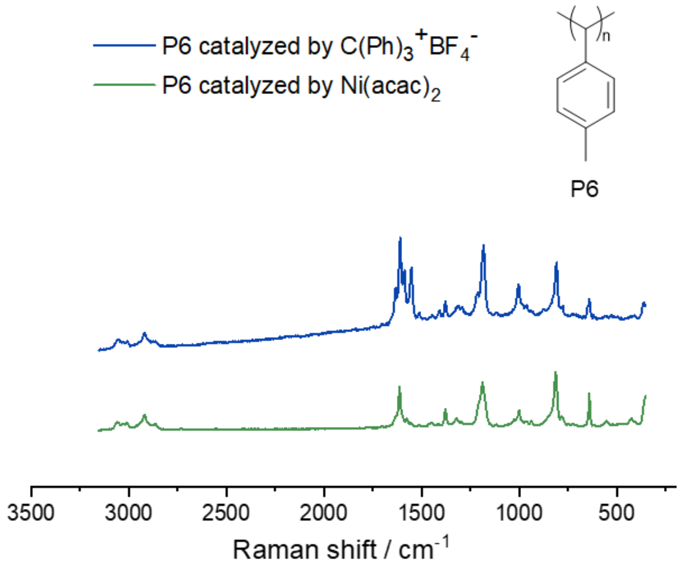

Fig. S169. Raman spectra of polymer **P6** synthesized using $C(Ph)_3^+BF_4^-$ (blue) and $Ni(acac)_2$ (green) as catalysts, recorded at room temperature with a 633 nm excitation wavelength.

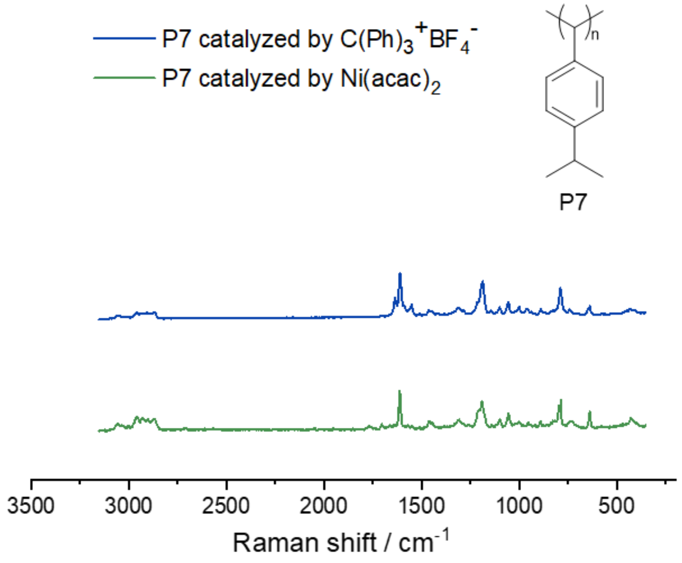

Fig. S170. Raman spectra of polymer **P7** synthesized using $C(Ph)_3^+BF_4^-$ (blue) and $Ni(acac)_2$ (green) as catalysts, recorded at room temperature with a 633 nm excitation wavelength.

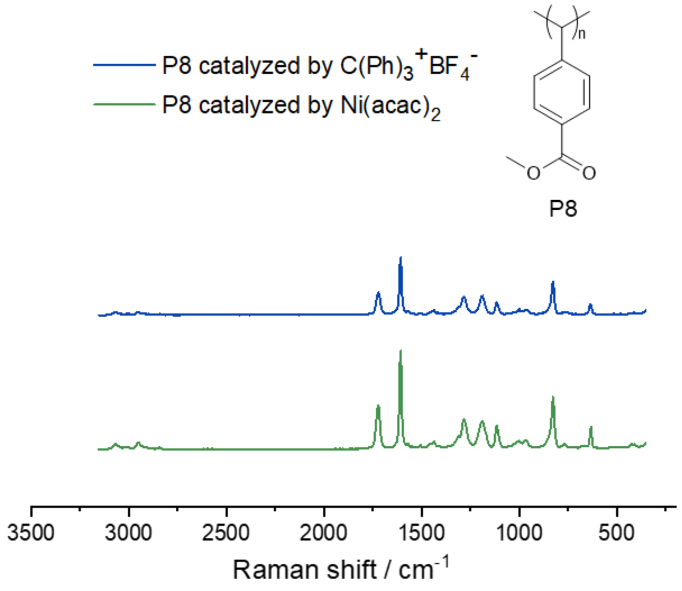

Fig. S171. Raman spectra of polymer **P8** synthesized using $C(Ph)_3^+BF_4^-$ (blue) and $Ni(acac)_2$ (green) as catalysts, recorded at room temperature with a 633 nm excitation wavelength.

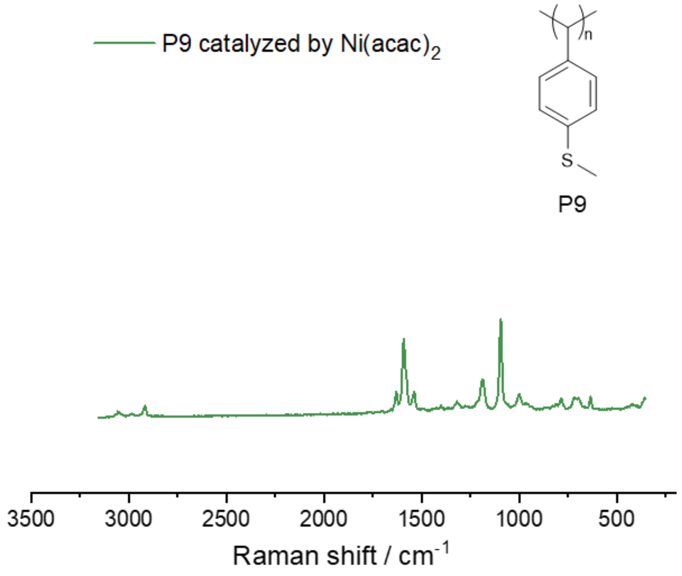

Fig. S172. Raman spectrum of polymer **P9** synthesized using $Ni(acac)_2$ as a catalyst, recorded at room temperature with a 633 nm excitation wavelength.

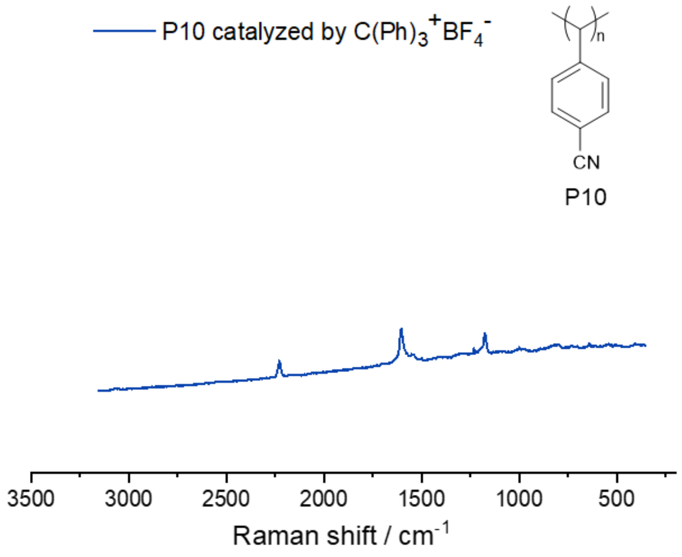

Fig. S173. Raman spectrum of polymer **P10** synthesized using $C(Ph)_3^+BF_4^-$ as a catalyst, recorded at room temperature with a 633 nm excitation wavelength.

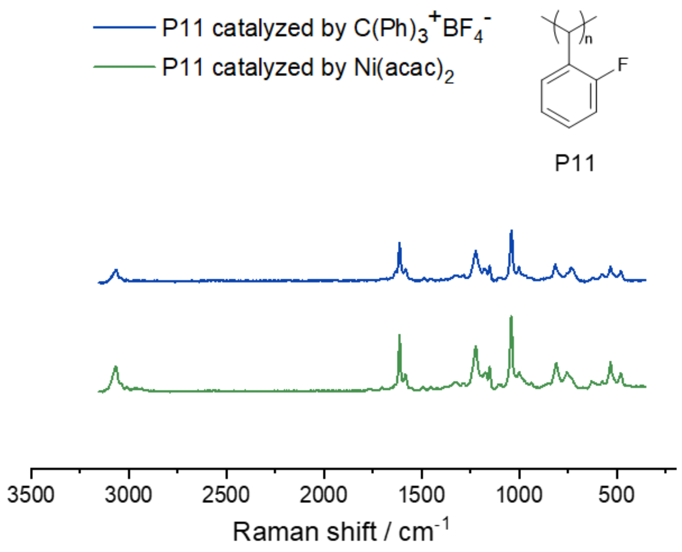

Fig. S174. Raman spectra of polymer **P11** synthesized using $C(Ph)_3^+BF_4^-$ (blue) and $Ni(acac)_2$ (green) as catalysts, recorded at room temperature with a 633 nm excitation wavelength.

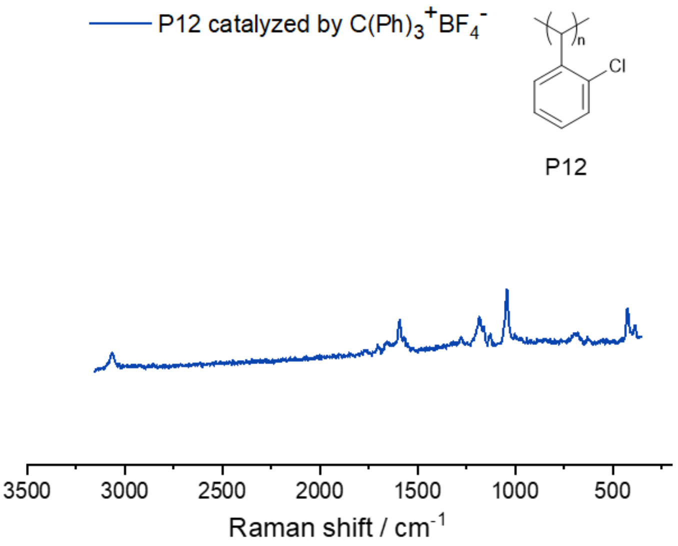

Fig. S175. Raman spectrum of polymer **P12** synthesized using $C(Ph)_3^+BF_4^-$ as a catalyst, recorded at room temperature with a 633 nm excitation wavelength.

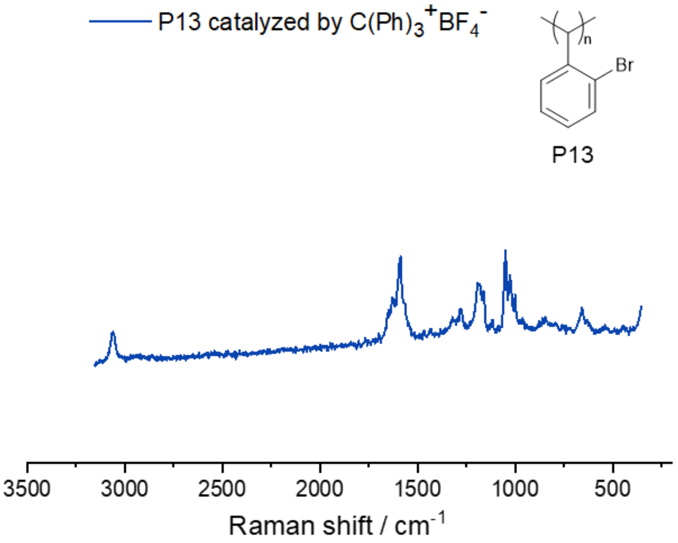

Fig. S176. Raman spectrum of polymer **P13** synthesized using $C(Ph)_3^+BF_4^-$ as a catalyst, recorded at room temperature with a 633 nm excitation wavelength.

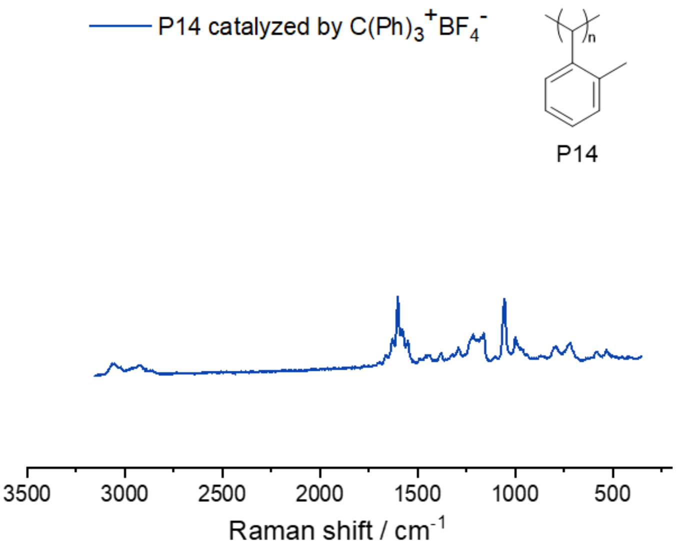

Fig. S177. Raman spectrum of polymer **P14** synthesized using $C(Ph)_3^+BF_4^-$ as a catalyst, recorded at room temperature with a 633 nm excitation wavelength.

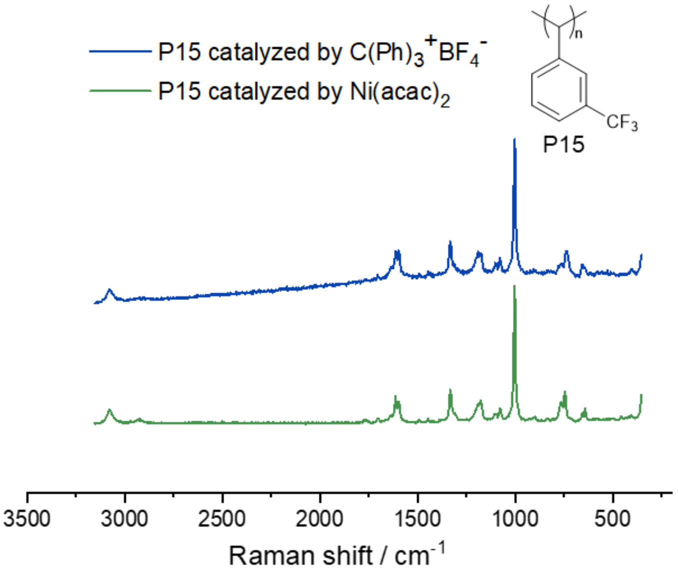

Fig. S178. Raman spectra of polymer **P15** synthesized using $C(Ph)_3^+BF_4^-$ (blue) and $Ni(acac)_2$ (green) as catalysts, recorded at room temperature with a 633 nm excitation wavelength.

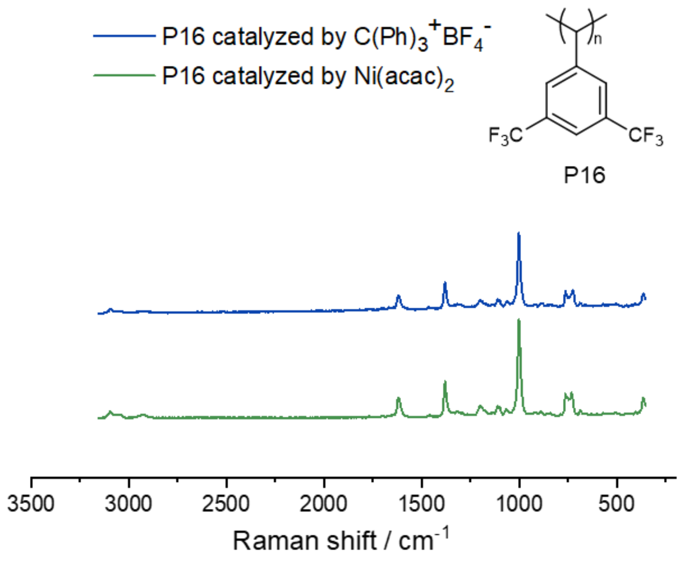

Fig. S179. Raman spectra of polymer **P16** synthesized using $C(Ph)_3^+BF_4^-$ (blue) and $Ni(acac)_2$ (green) as catalysts, recorded at room temperature with a 633 nm excitation wavelength.

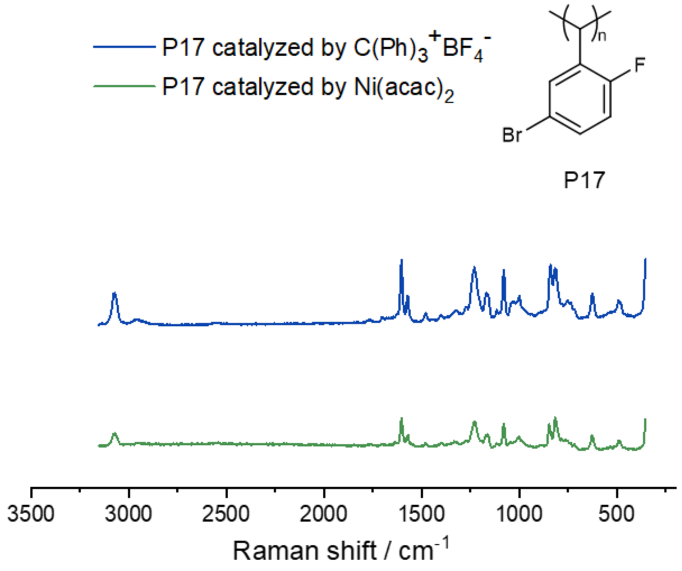

Fig. S180. Raman spectra of polymer **P17** synthesized using $C(Ph)_3^+BF_4^-$ (blue) and $Ni(acac)_2$ (green) as catalysts, recorded at room temperature with a 633 nm excitation wavelength.

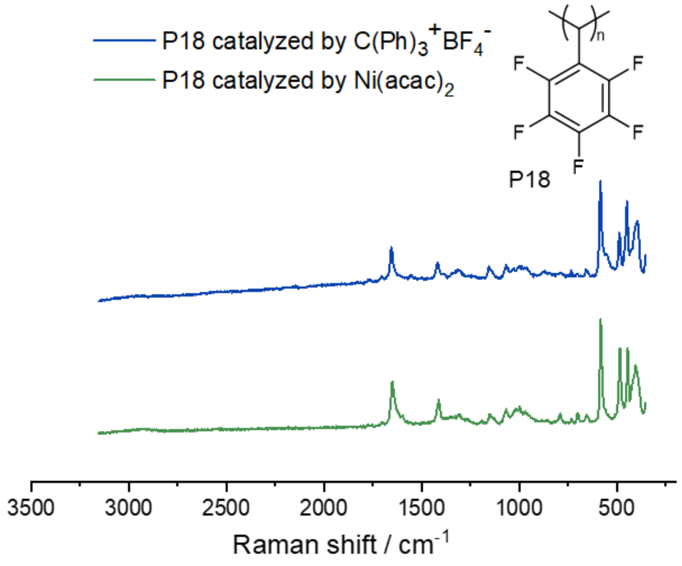

Fig. S181. Raman spectra of polymer **P18** synthesized using $C(Ph)_3^+BF_4^-$ (blue) and $Ni(acac)_2$ (green) as catalysts, recorded at room temperature with a 633 nm excitation wavelength.

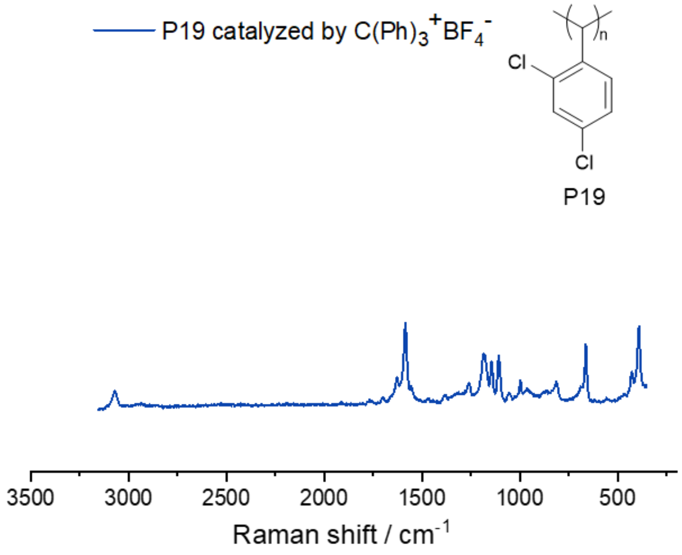

Fig. S182. Raman spectrum of polymer **P19** synthesized using $C(Ph)_3^+BF_4^-$ as a catalyst, recorded at room temperature with a 633 nm excitation wavelength.

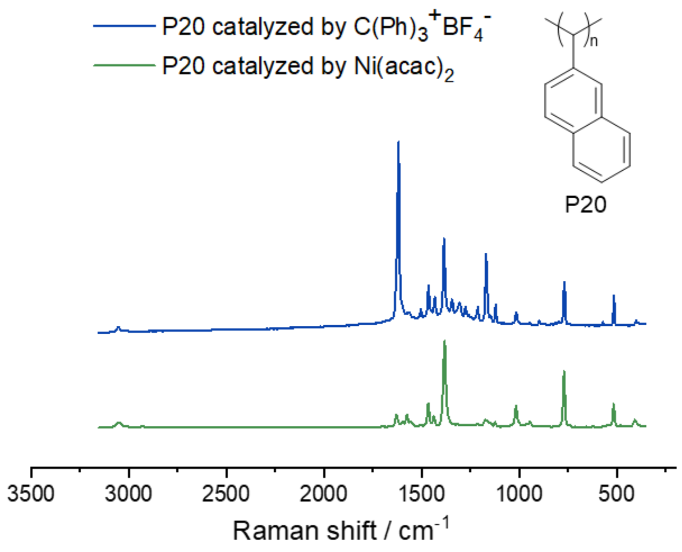

Fig. S183. Raman spectra of polymer **P20** synthesized using $C(Ph)_3^+BF_4^-$ (blue) and $Ni(acac)_2$ (green) as catalysts, recorded at room temperature with a 633 nm excitation wavelength.

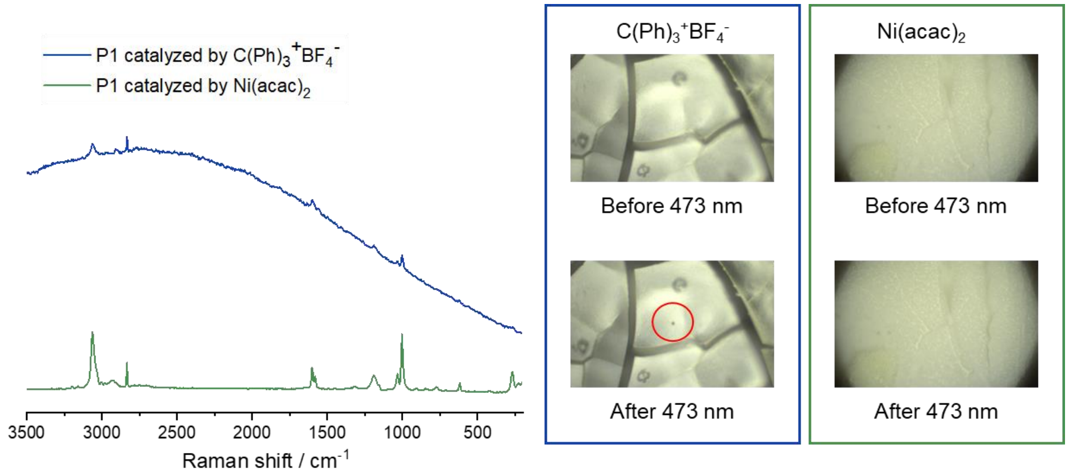

Fig. S184. Raman spectra of polymer **P1** synthesized using $C(Ph)_3^+BF_4^-$ (blue) and $Ni(acac)_2$ (green) as catalysts, recorded at room temperature with a 473 nm excitation wavelength.

WAXS pattern

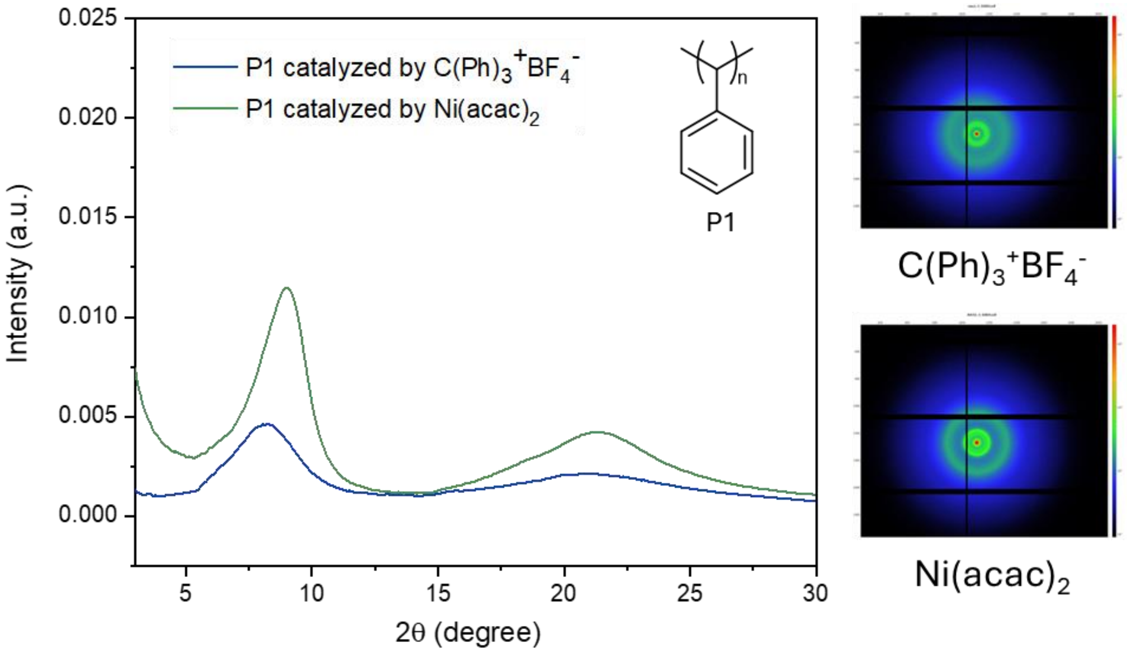

Fig. S185. WAXS pattern of polymer **P1** synthesized using $C(Ph)_3^+BF_4^-$ (blue) and $Ni(acac)_2$ (green) as catalysts, recorded at room temperature.

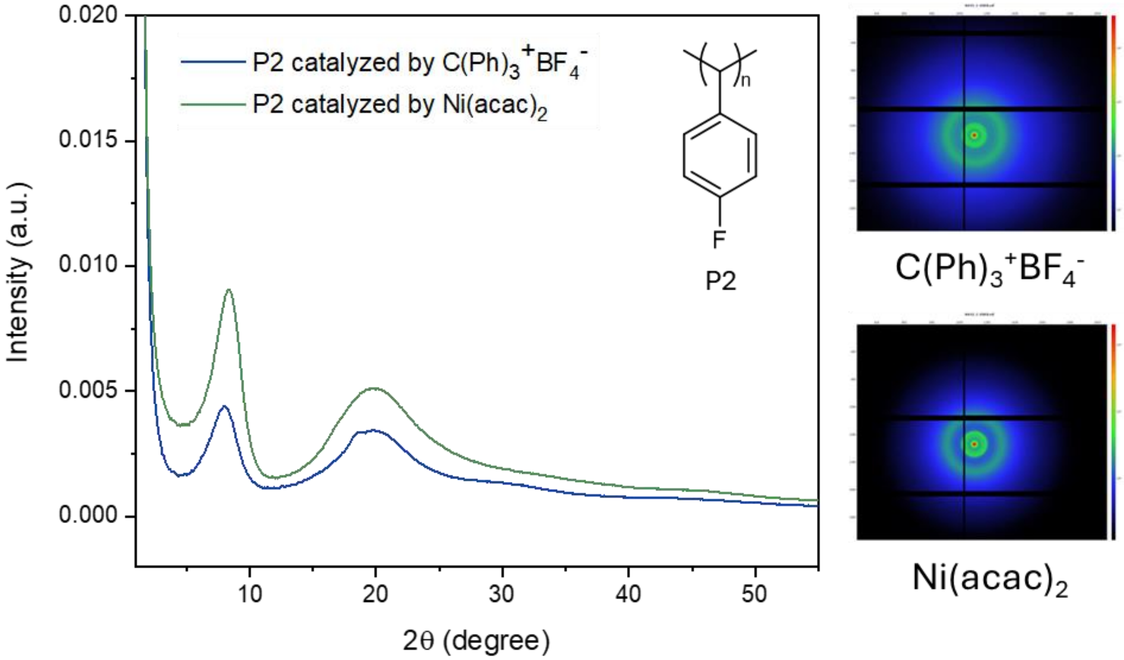

Fig. S186. WAXS pattern of polymer **P2** synthesized using $C(Ph)_3^+BF_4^-$ (blue) and $Ni(acac)_2$ (green) as catalysts, recorded at room temperature.

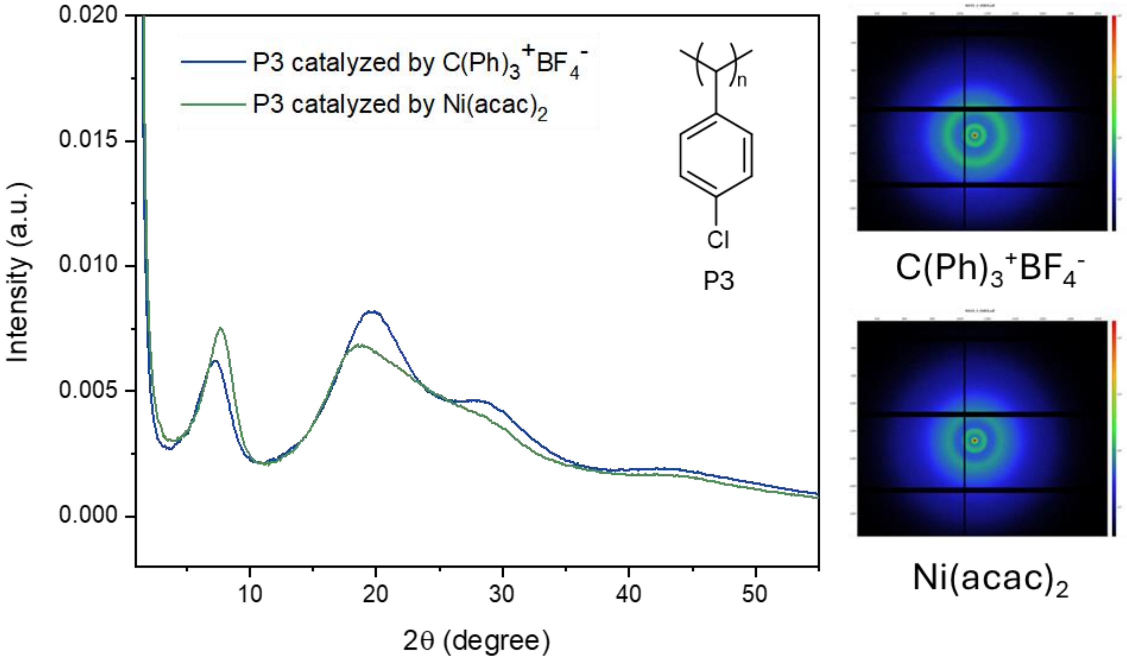

Fig. S187. WAXS pattern of polymer **P3** synthesized using $C(Ph)_3^+BF_4^-$ (blue) and $Ni(acac)_2$ (green) as catalysts, recorded at room temperature.

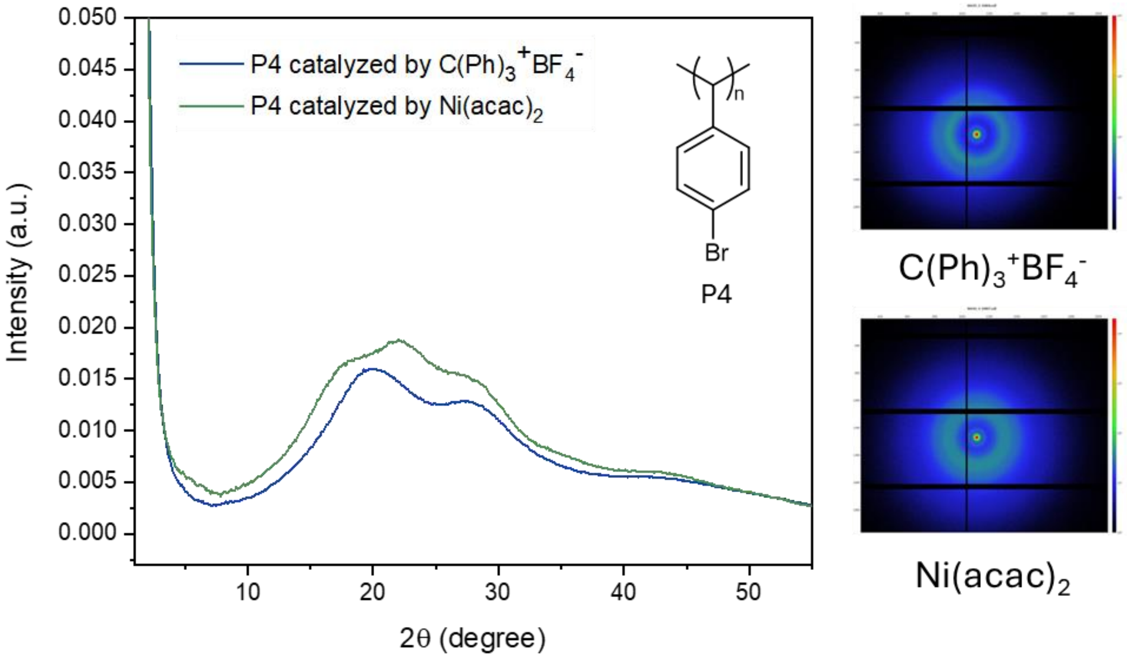

Fig. S188. WAXS pattern of polymer **P4** synthesized using $C(Ph)_3^+BF_4^-$ (blue) and $Ni(acac)_2$ (green) as catalysts, recorded at room temperature.

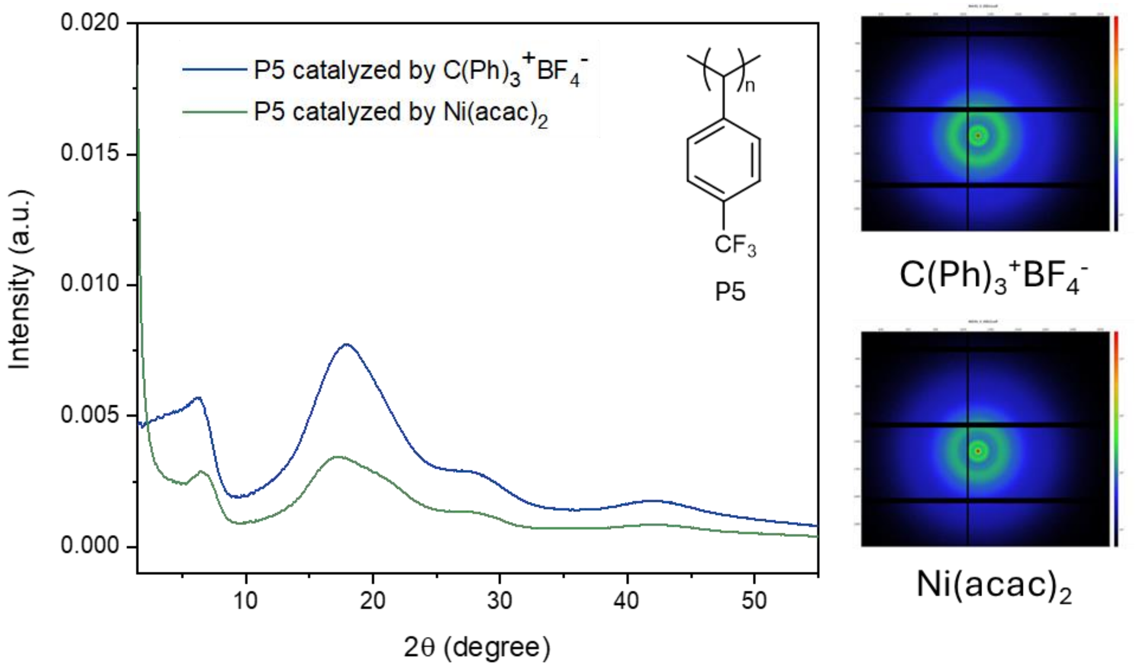

Fig. S189. WAXS pattern of polymer **P5** synthesized using $C(Ph)_3^+BF_4^-$ (blue) and $Ni(acac)_2$ (green) as catalysts, recorded at room temperature.

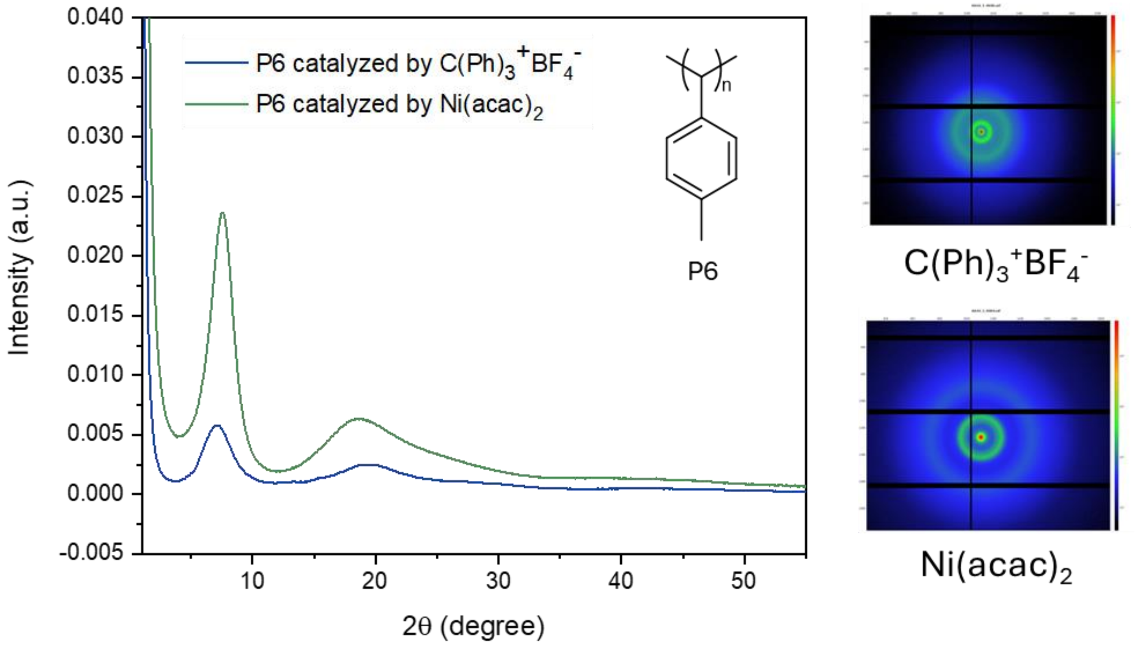

Fig. S190. WAXS pattern of polymer **P6** synthesized using $C(Ph)_3^+BF_4^-$ (blue) and $Ni(acac)_2$ (green) as catalysts, recorded at room temperature.

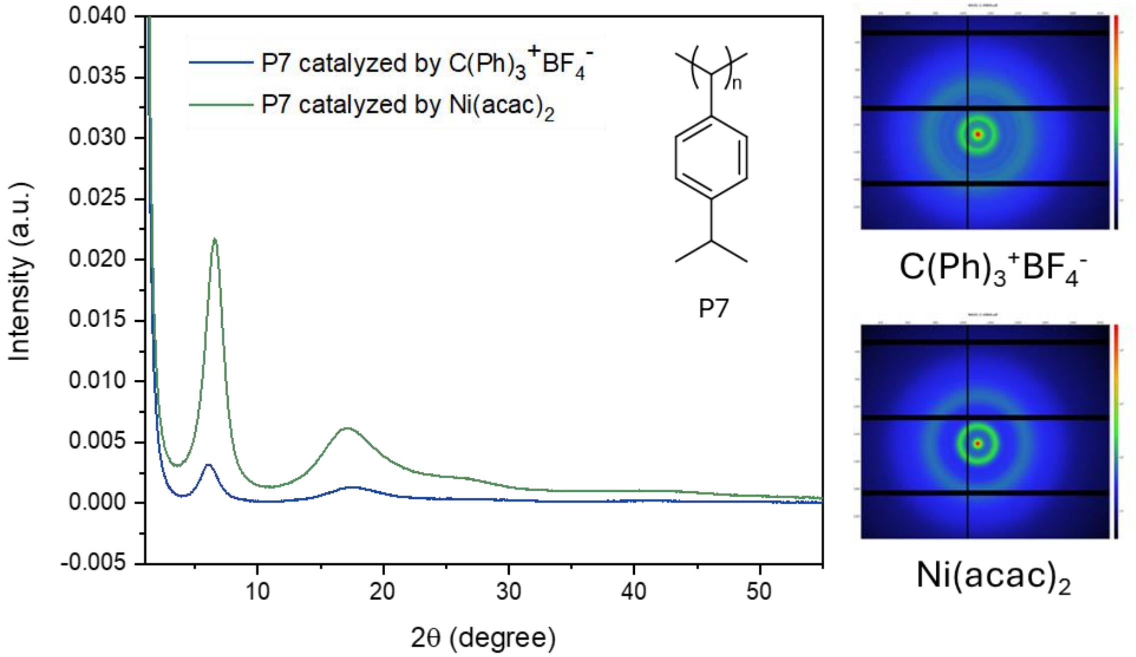

Fig. S191. WAXS pattern of polymer **P7** synthesized using $C(Ph)_3^+BF_4^-$ (blue) and $Ni(acac)_2$ (green) as catalysts, recorded at room temperature.

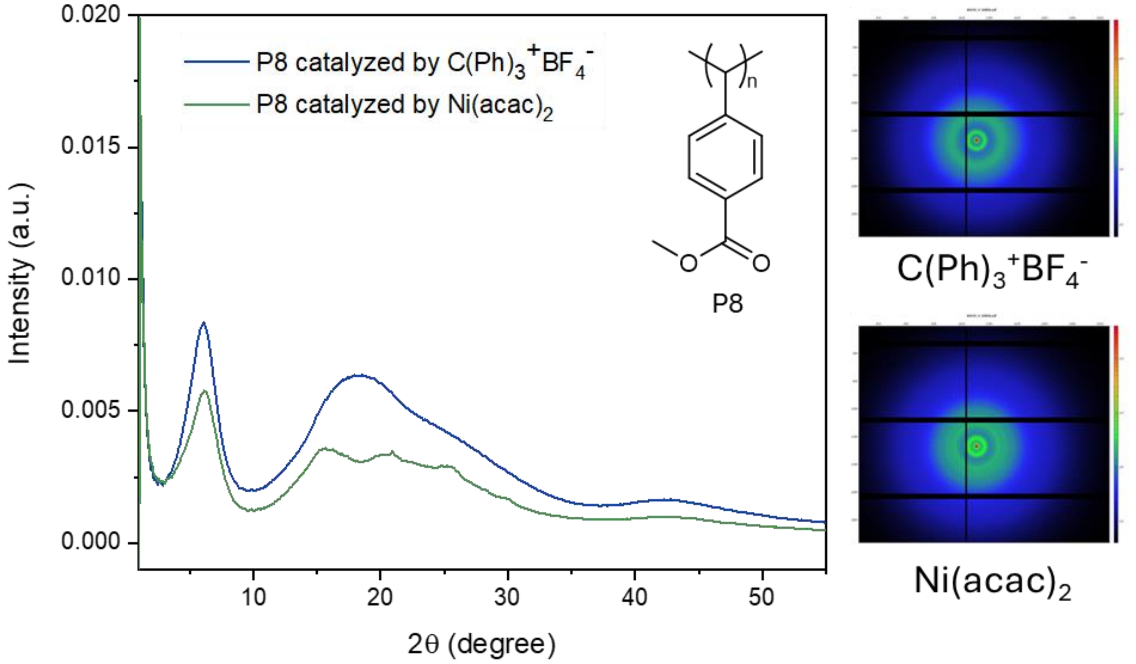

Fig. S192. WAXS pattern of polymer **P8** synthesized using $C(Ph)_3^+BF_4^-$ (blue) and $Ni(acac)_2$ (green) as catalysts, recorded at room temperature.

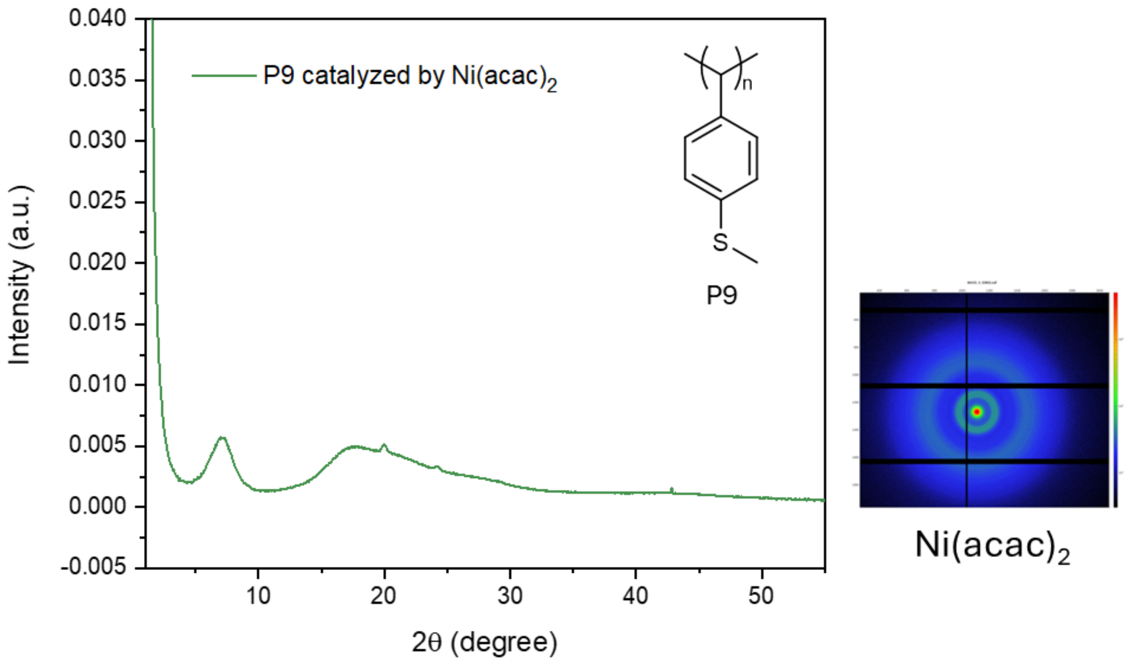

Fig. S193. WAXS pattern of polymer **P9** synthesized $Ni(acac)_2$ as a catalyst, recorded at room temperature.

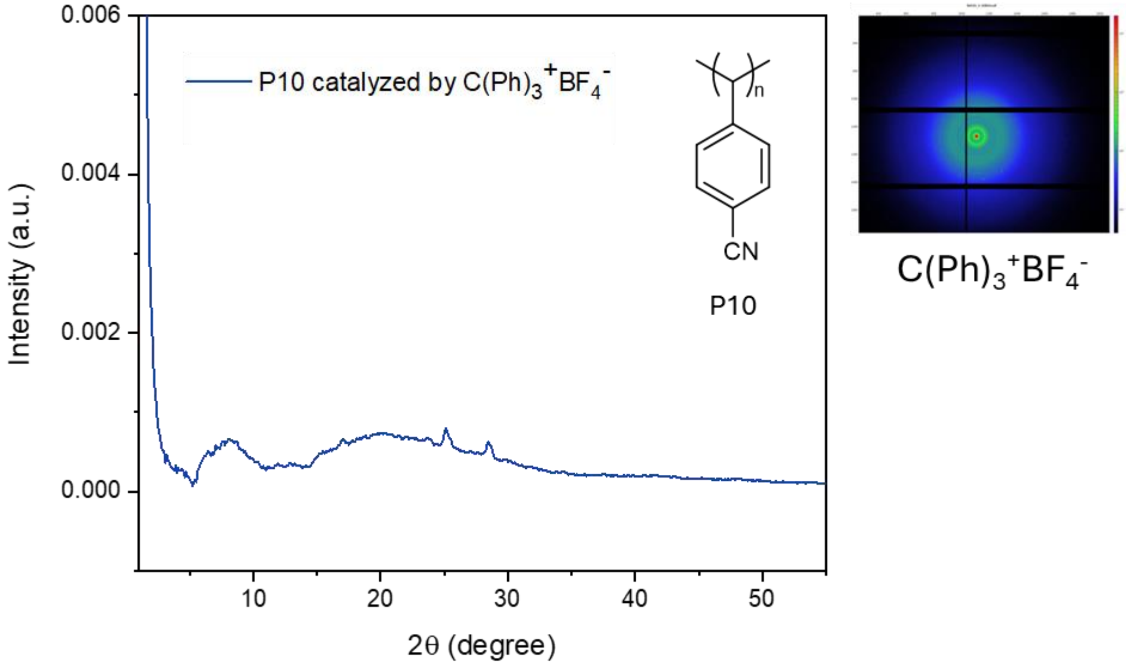

Fig. S194. WAXS pattern of polymer **P10** synthesized using $C(Ph)_3^+BF_4^-$ as a catalyst, recorded at room temperature.

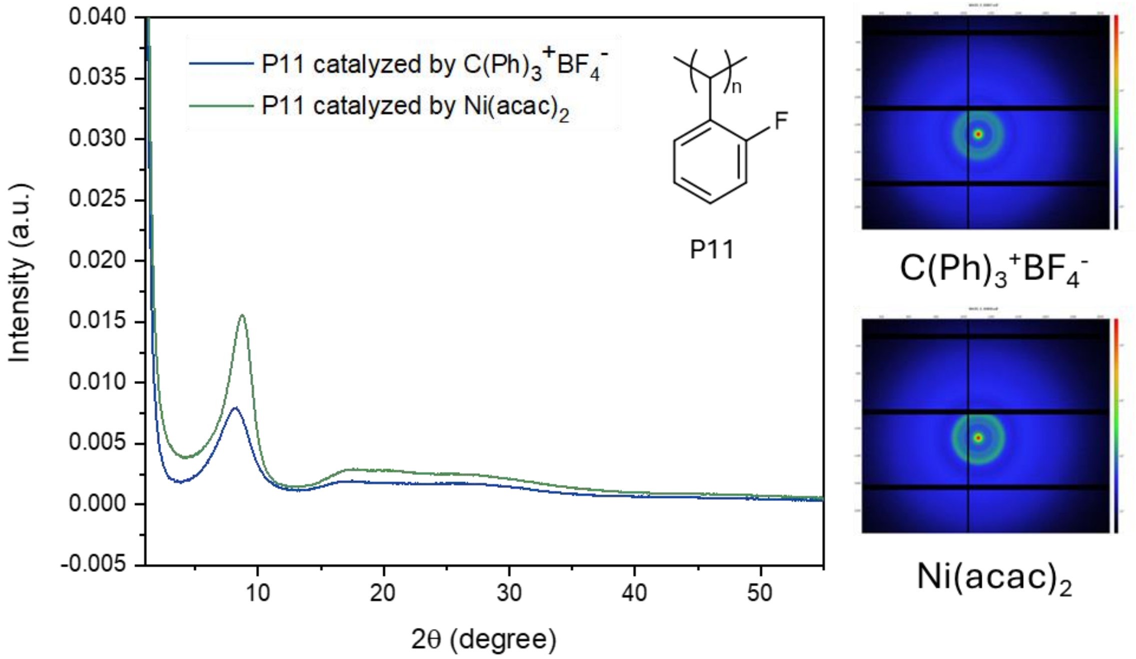

Fig. S195. WAXS pattern of polymer **P11** synthesized using $C(Ph)_3^+BF_4^-$ (blue) and $Ni(acac)_2$ (green) as catalysts, recorded at room temperature.

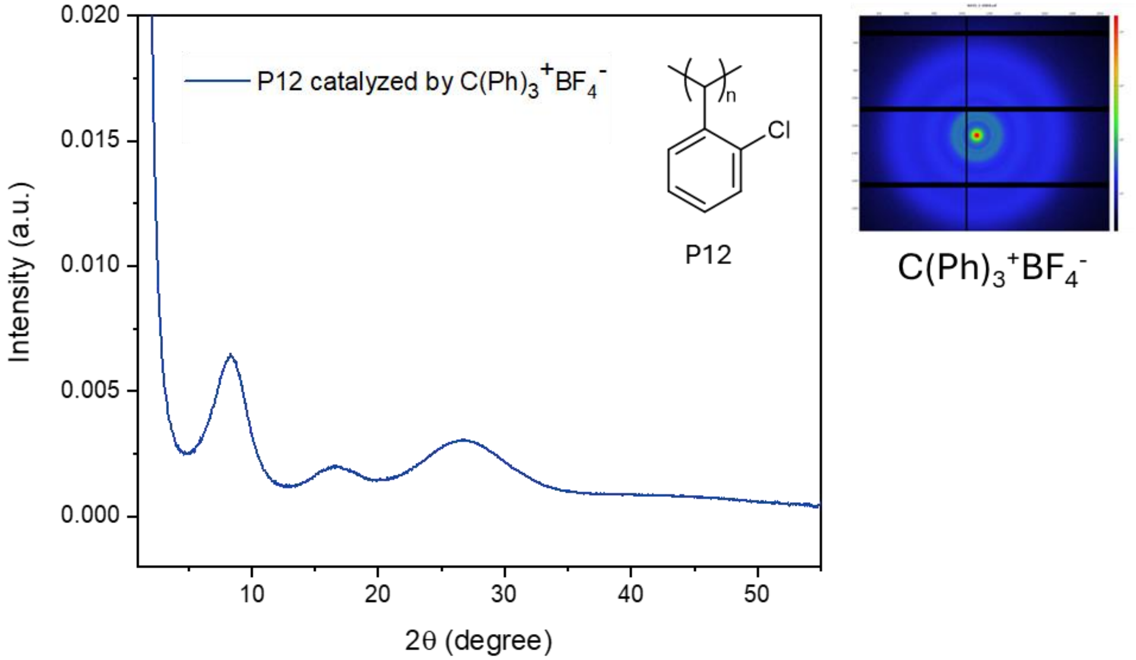

Fig. S196. WAXS pattern of polymer **P12** synthesized using $C(Ph)_3^+BF_4^-$ as a catalyst, recorded at room temperature.

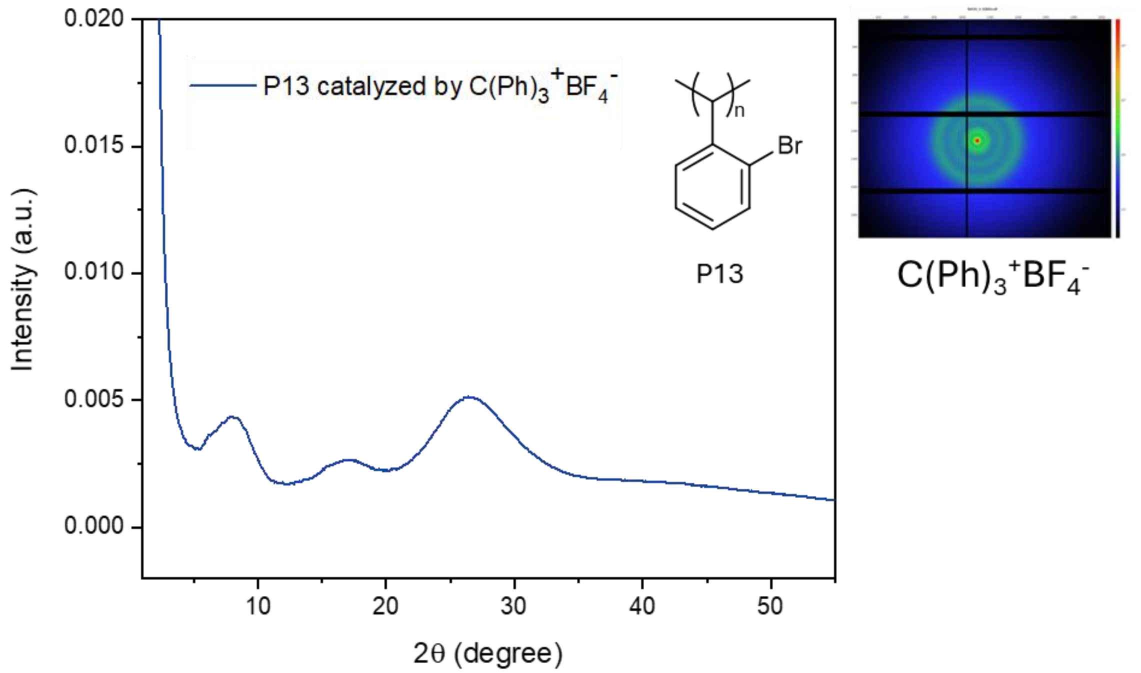

Fig. S197. WAXS pattern of polymer **P13** synthesized using $C(Ph)_3^+BF_4^-$ as a catalyst, recorded at room temperature.

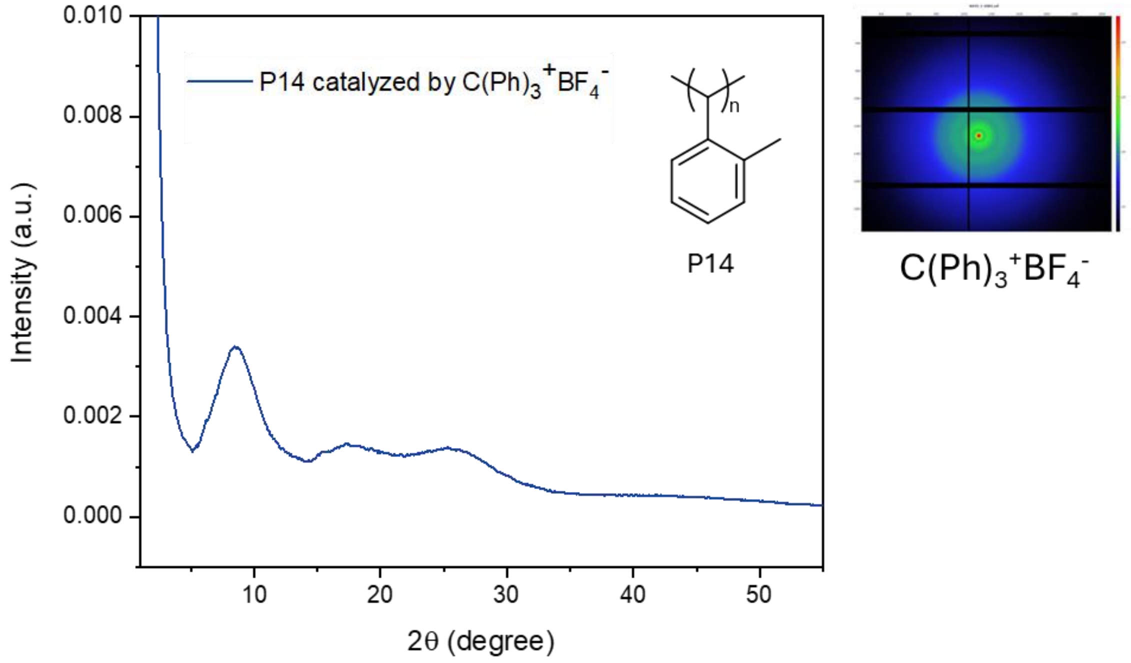

Fig. S198. WAXS pattern of polymer **P14** synthesized using $C(Ph)_3^+BF_4^-$ as a catalyst, recorded at room temperature.

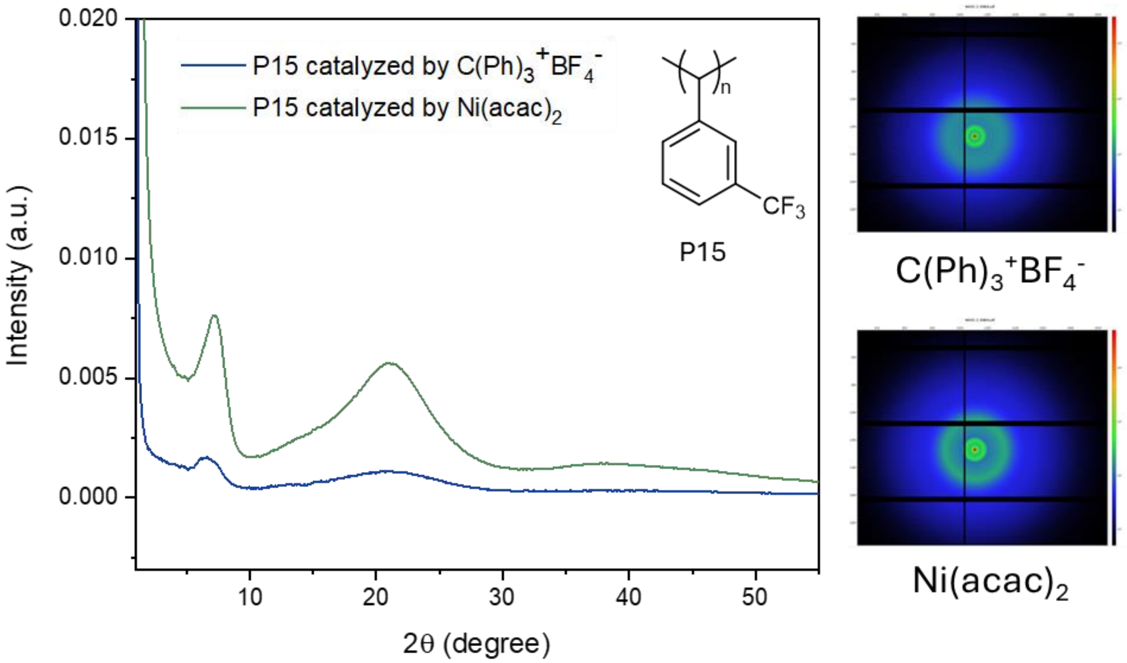

Fig. S199. WAXS pattern of polymer **P15** synthesized using $C(Ph)_3^+BF_4^-$ (blue) and $Ni(acac)_2$ (green) as catalysts, recorded at room temperature.

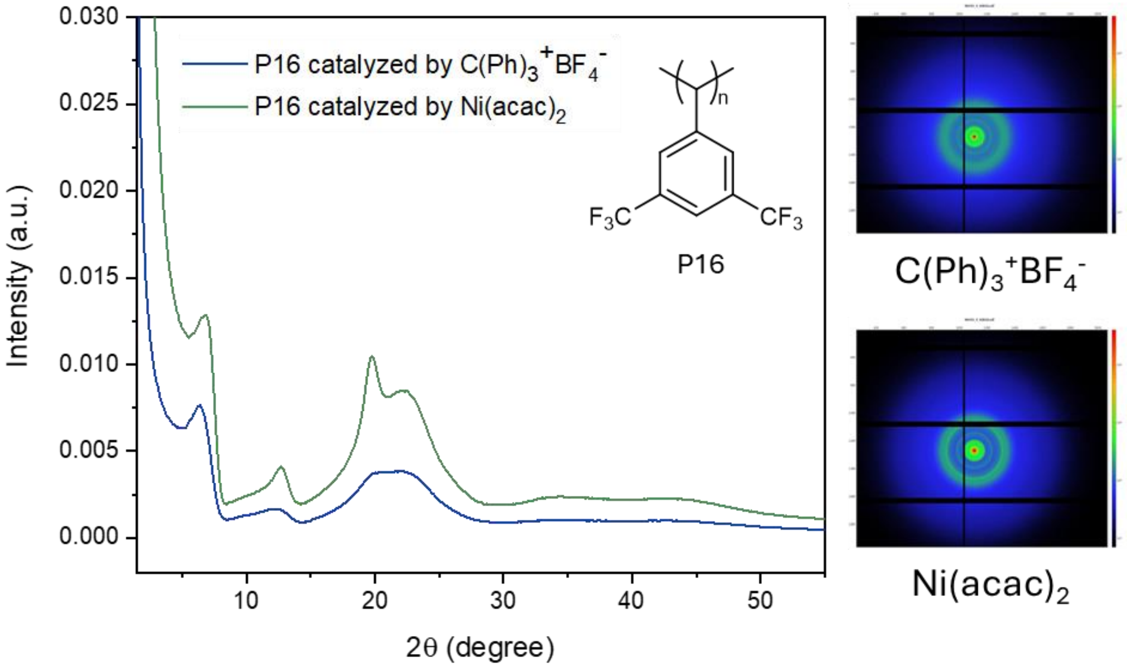

Fig. S200. WAXS pattern of polymer **P16** synthesized using $C(Ph)_3^+BF_4^-$ (blue) and $Ni(acac)_2$ (green) as catalysts, recorded at room temperature.

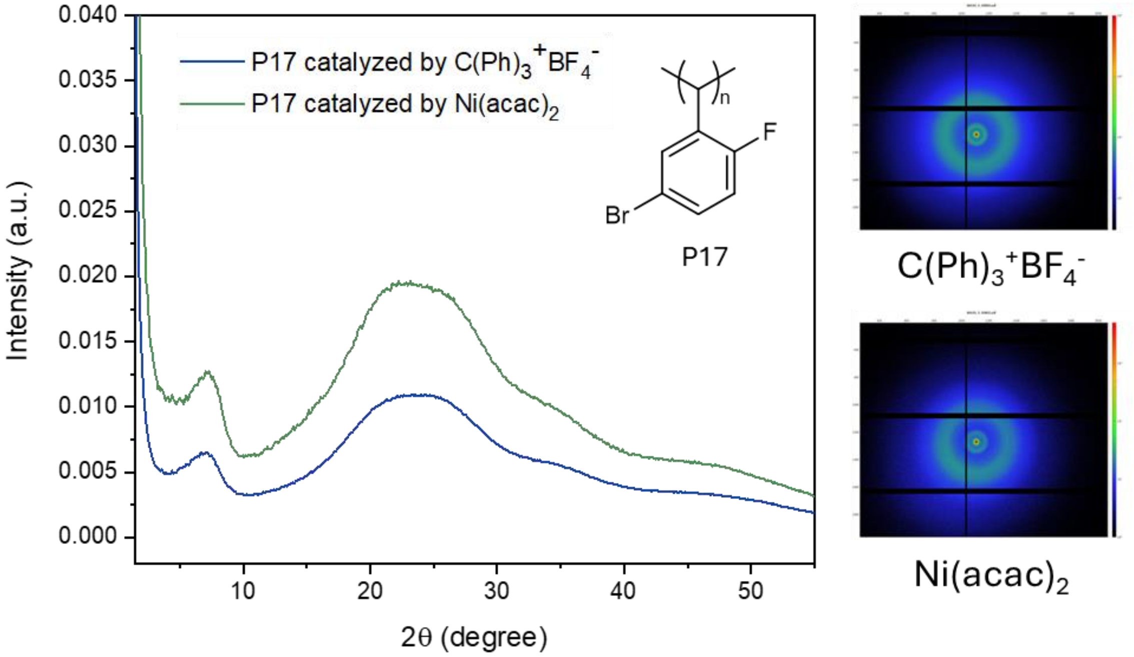

Fig. S201. WAXS pattern of polymer **P17** synthesized using $C(Ph)_3^+BF_4^-$ (blue) and $Ni(acac)_2$ (green) as catalysts, recorded at room temperature.

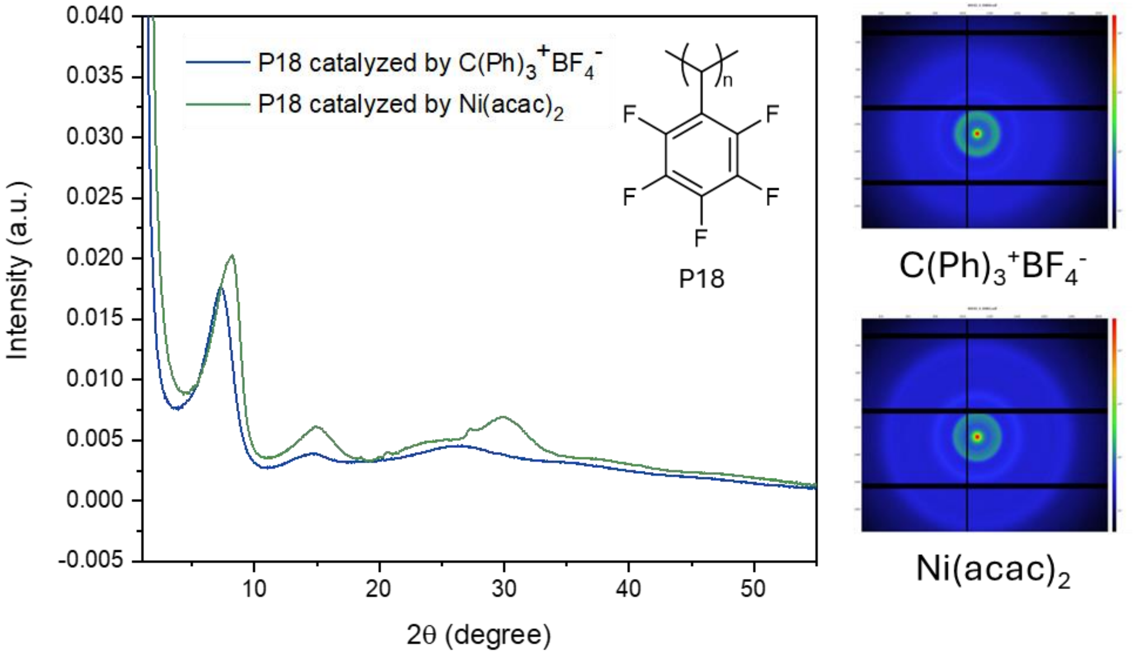

Fig. S202. WAXS pattern of polymer **P18** synthesized using $C(Ph)_3^+BF_4^-$ (blue) and $Ni(acac)_2$ (green) as catalysts, recorded at room temperature.

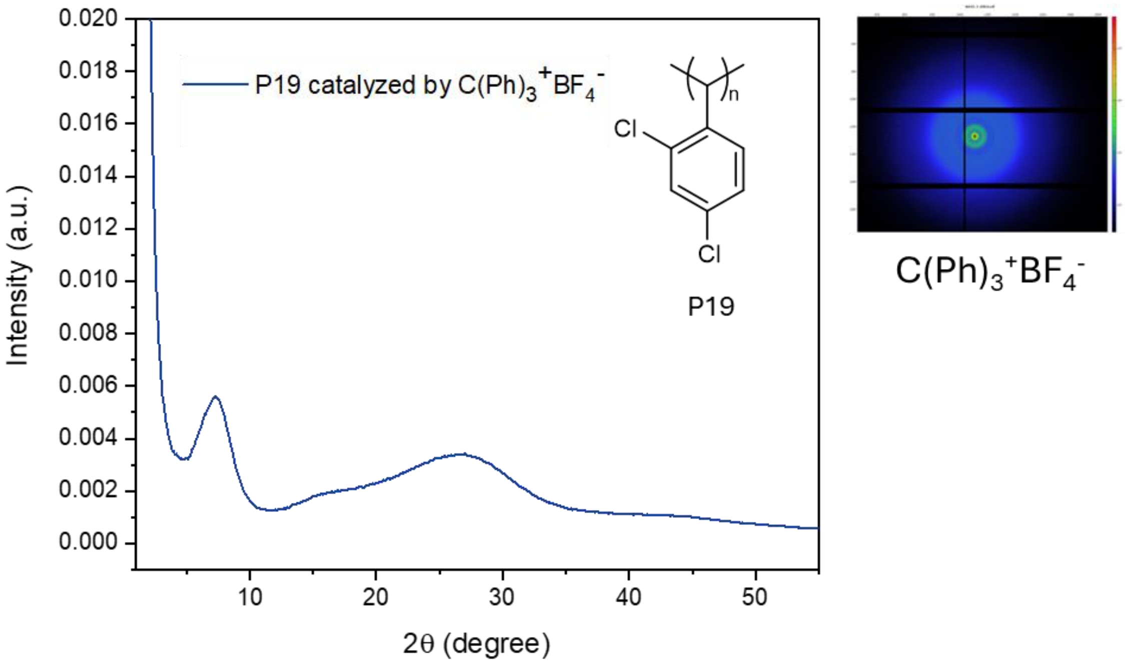

Fig. S203. WAXS pattern of polymer **P19** synthesized using $C(Ph)_3{}^+BF_4{}^-$ as a catalyst, recorded at room temperature.

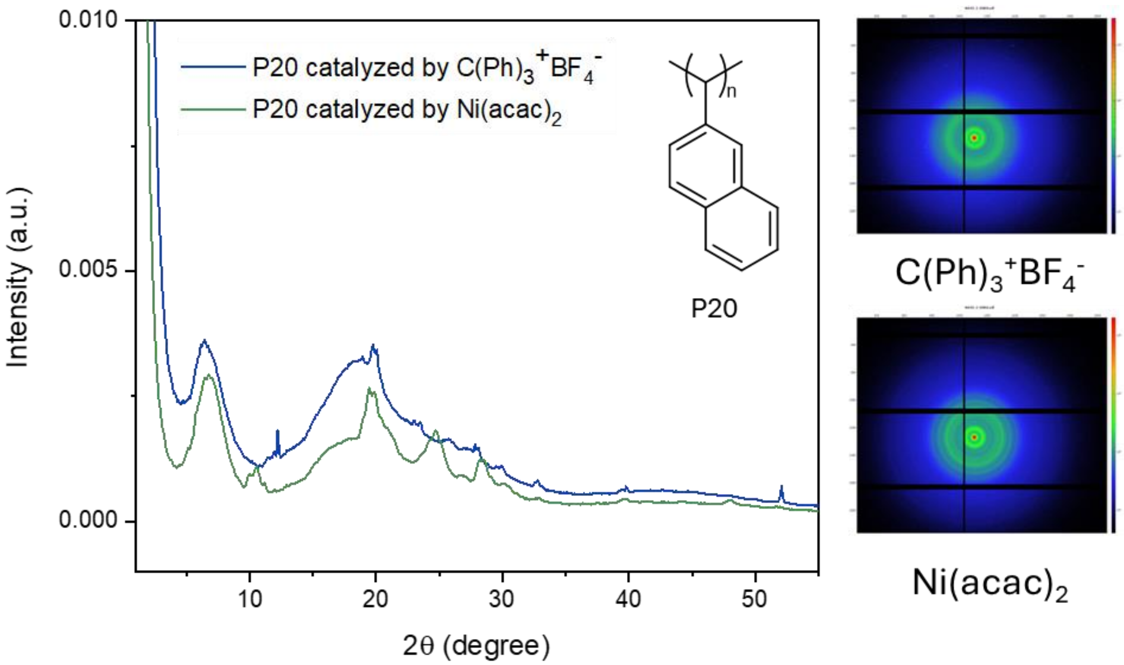

Fig. S204. WAXS pattern of polymer **P20** synthesized using $C(Ph)_3{}^+BF_4{}^-$ (blue) and $Ni(acac)_2$ (green) as catalysts, recorded at room temperature.

TGA curves

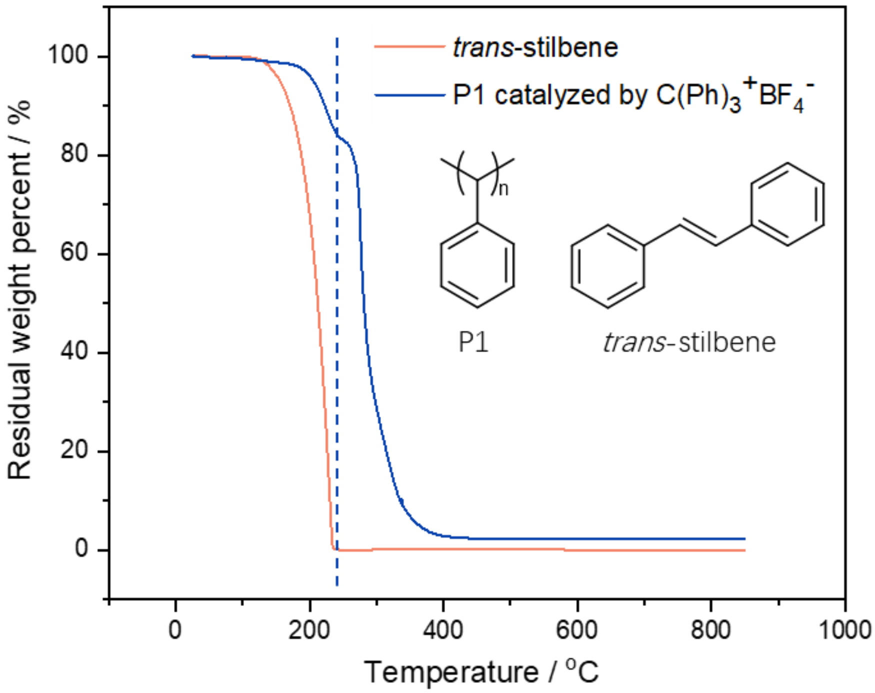

Fig. S205. TGA curves of polymer **P1** synthesized using $C(Ph)_3^+BF_4^-$ as a catalyst and *trans*-stilbene, recorded under a nitrogen atmosphere with a heating rate of 10 °C/min.

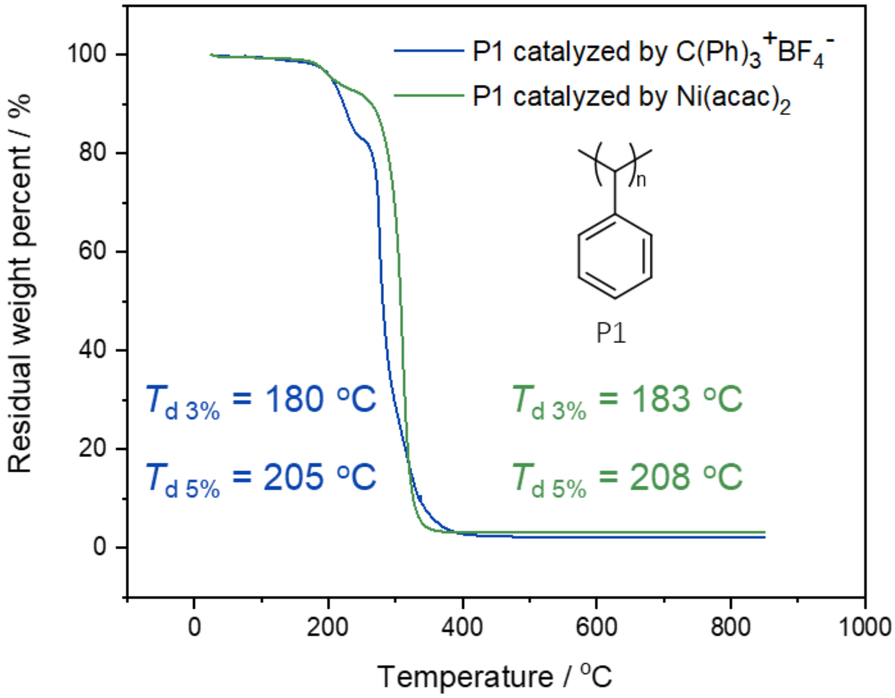

Fig. S206. TGA curves of polymer **P1** synthesized using $C(Ph)_3^+BF_4^-$ (blue) and $Ni(acac)_2$ (green) as catalysts, recorded under a nitrogen atmosphere with a heating rate of 10 °C/min.

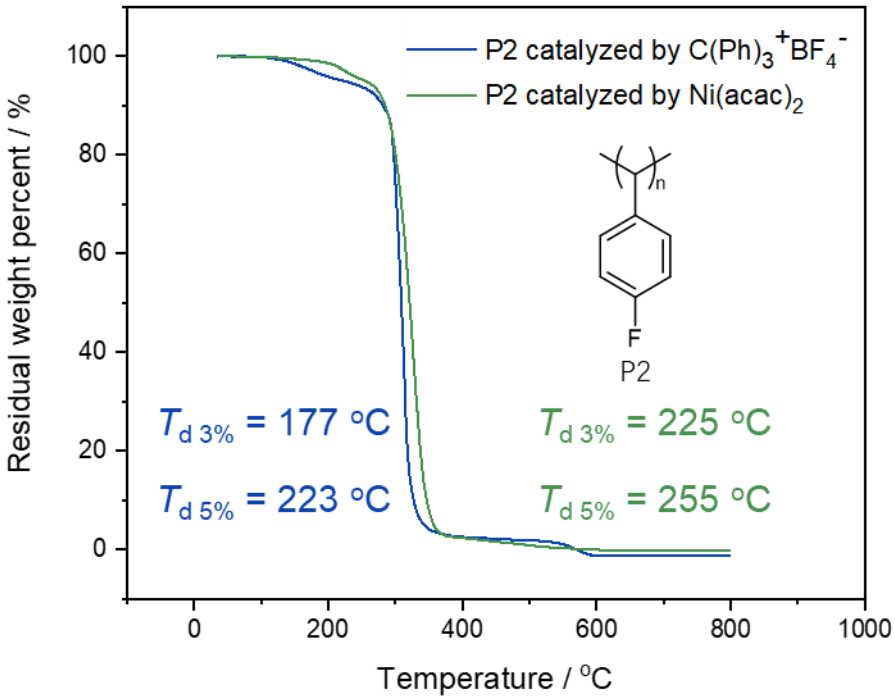

Fig. S207. TGA curves of polymer **P2** synthesized using $C(Ph)_3^+BF_4^-$ (blue) and $Ni(acac)_2$ (green) as catalysts, recorded under a nitrogen atmosphere with a heating rate of 10 °C/min.

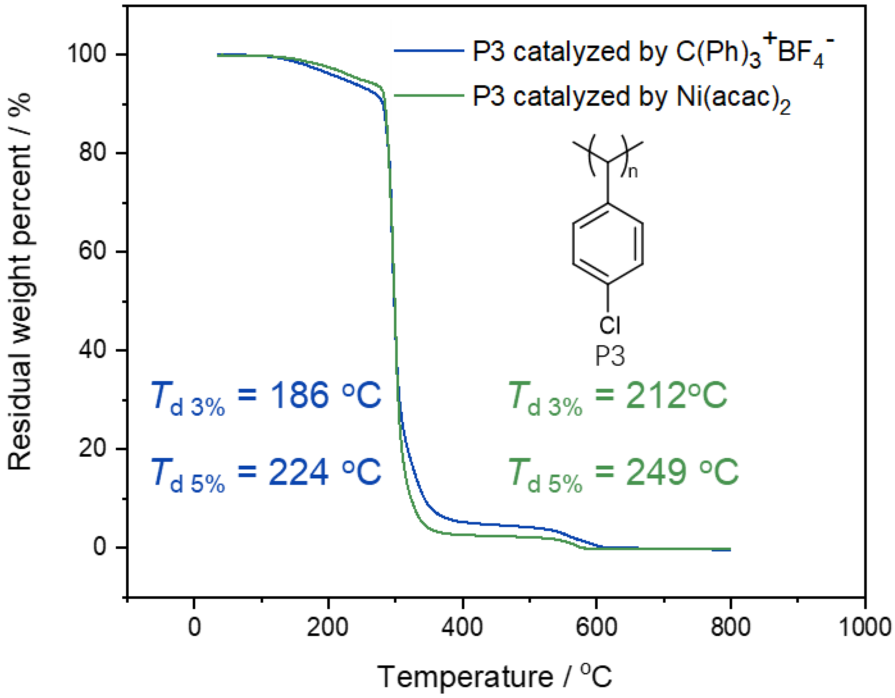

Fig. S208. TGA curves of polymer **P3** synthesized using $C(Ph)_3^+BF_4^-$ (blue) and $Ni(acac)_2$ (green) as catalysts, recorded under a nitrogen atmosphere with a heating rate of 10 °C/min.

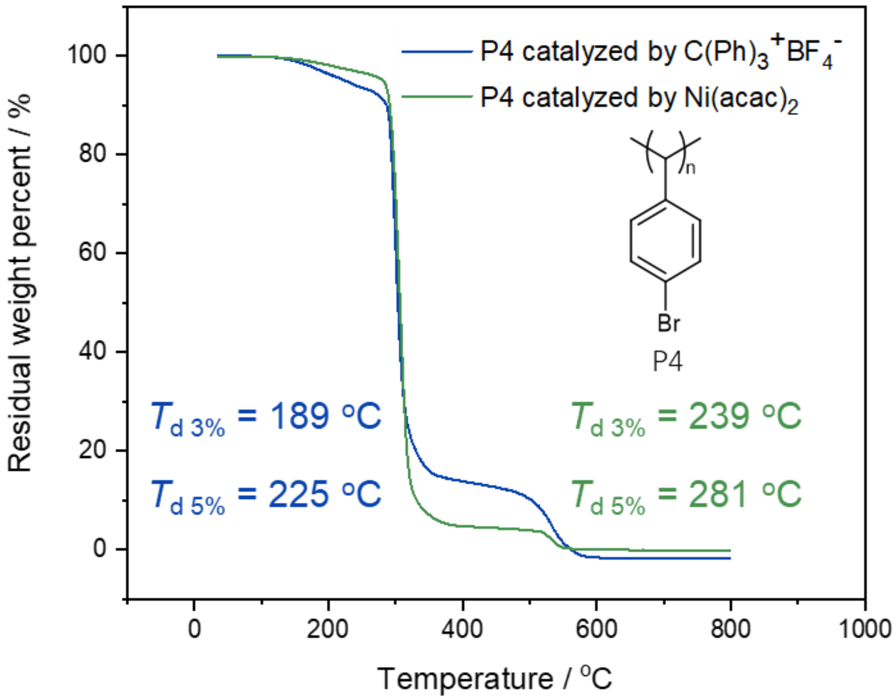

Fig. S209. TGA curves of polymer **P4** synthesized using $C(Ph)_3{}^+BF_4{}^-$ (blue) and $Ni(acac)_2$ (green) as catalysts, recorded under a nitrogen atmosphere with a heating rate of 10 °C/min.

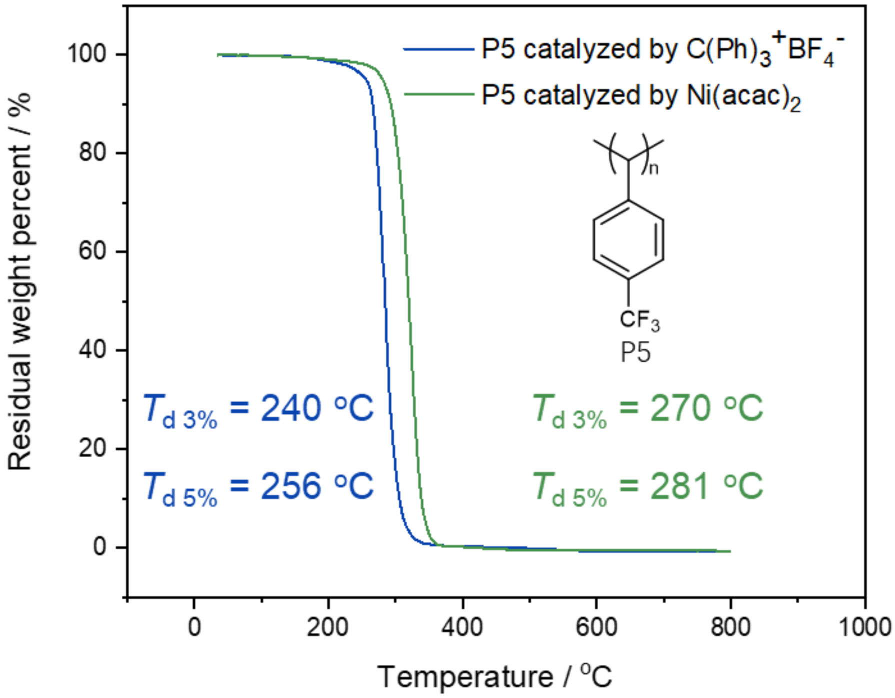

Fig. S210. TGA curves of polymer **P5** synthesized using $C(Ph)_3{}^+BF_4{}^-$ (blue) and $Ni(acac)_2$ (green) as catalysts, recorded under a nitrogen atmosphere with a heating rate of 10 °C/min.

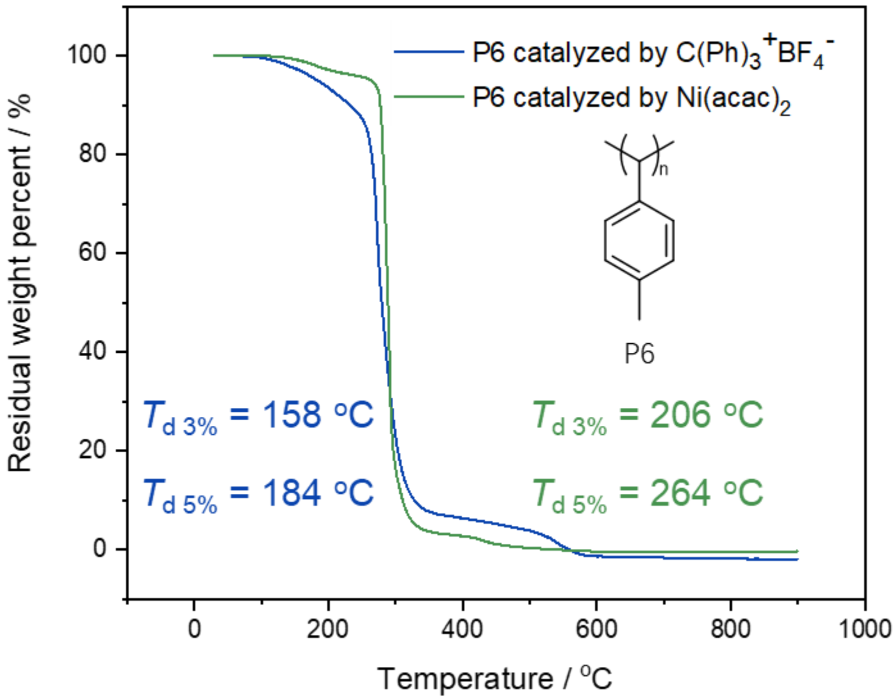

Fig. S211. TGA curves of polymer **P6** synthesized using $C(Ph)_3^+BF_4^-$ (blue) and $Ni(acac)_2$ (green) as catalysts, recorded under a nitrogen atmosphere with a heating rate of 10 °C/min.

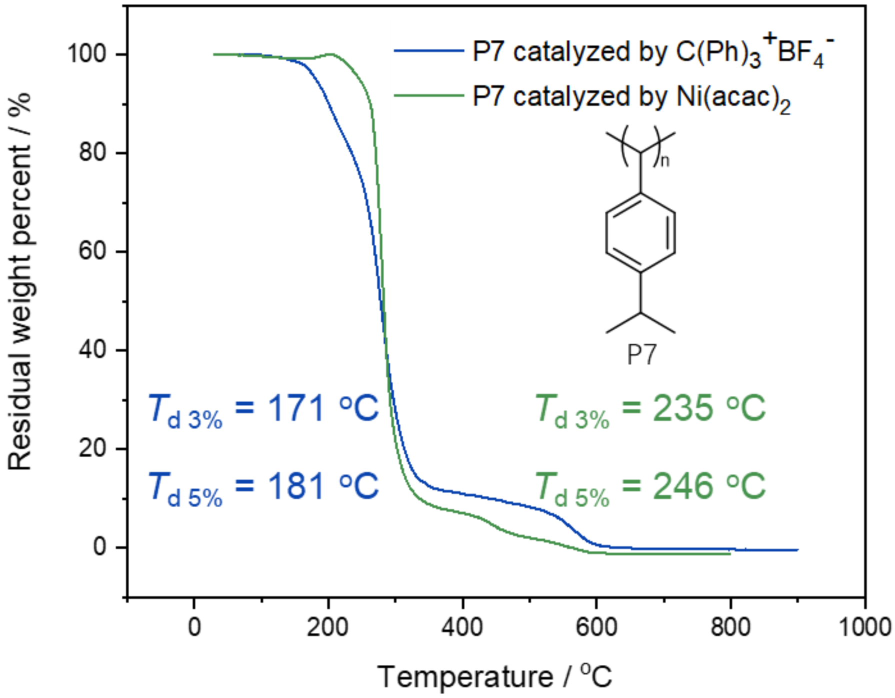

Fig. S212. TGA curves of polymer **P7** synthesized using $C(Ph)_3^+BF_4^-$ (blue) and $Ni(acac)_2$ (green) as catalysts, recorded under a nitrogen atmosphere with a heating rate of 10 °C/min.

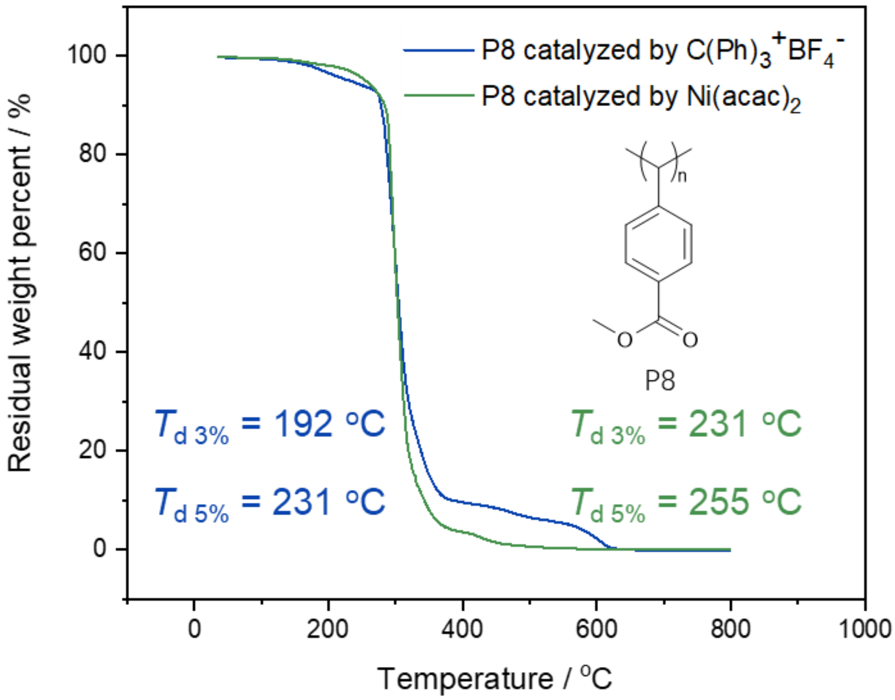

Fig. S213. TGA curves of polymer **P8** synthesized using $C(Ph)_3^+BF_4^-$ (blue) and $Ni(acac)_2$ (green) as catalysts, recorded under a nitrogen atmosphere with a heating rate of 10 °C/min.

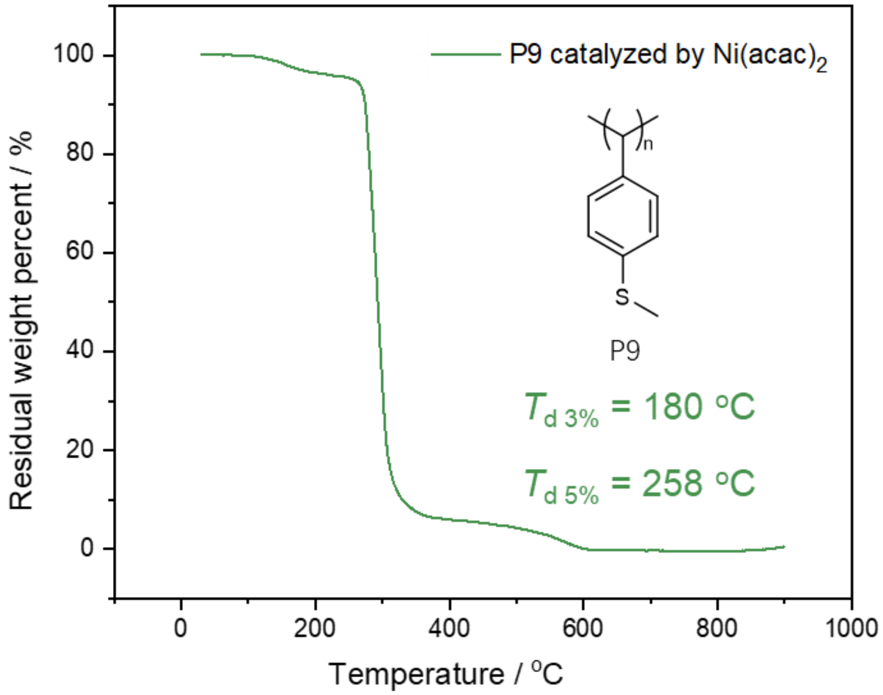

Fig. S214. TGA curve of polymer **P9** synthesized using $Ni(acac)_2$ as a catalyst, recorded under a nitrogen atmosphere with a heating rate of 10 °C/min.

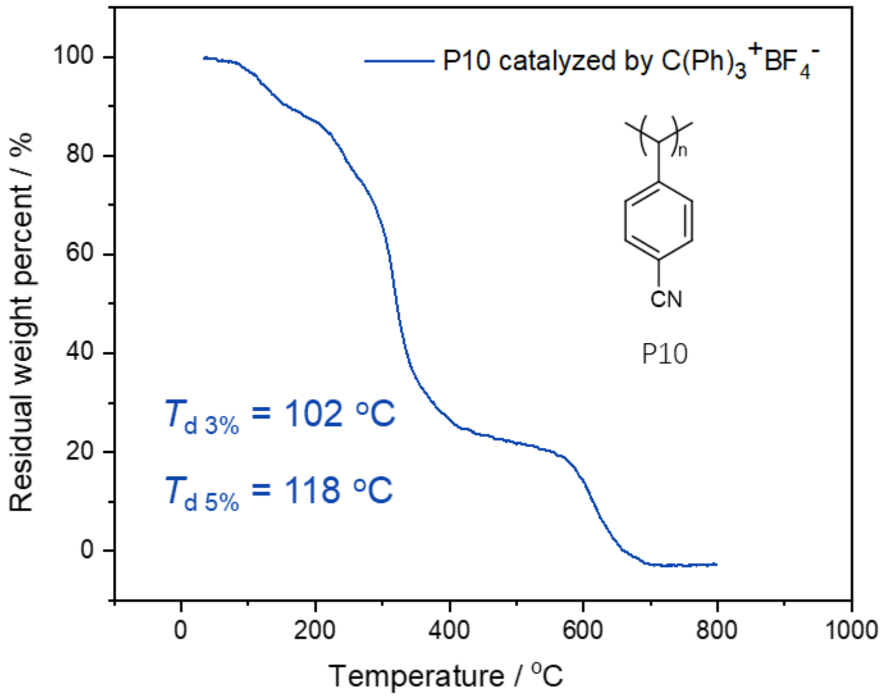

Fig. S215. TGA curve of polymer **P10** synthesized using $C(Ph)_3^+BF_4^-$ as a catalyst, recorded under a nitrogen atmosphere with a heating rate of 10 °C/min.

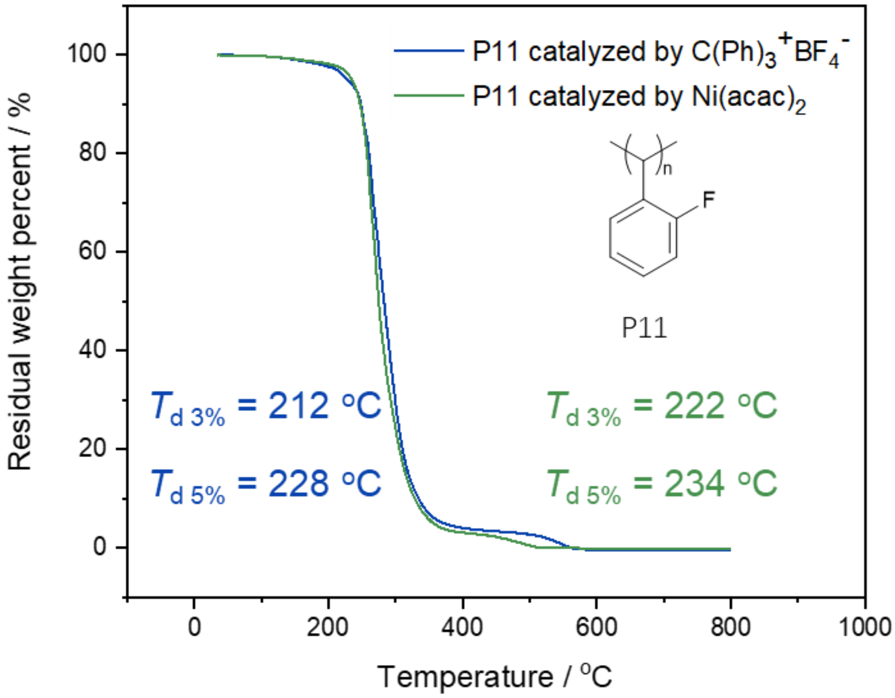

Fig. S216. TGA curves of polymer **P11** synthesized using $C(Ph)_3^+BF_4^-$ (blue) and $Ni(acac)_2$ (green) as catalysts, recorded under a nitrogen atmosphere with a heating rate of 10 °C/min.

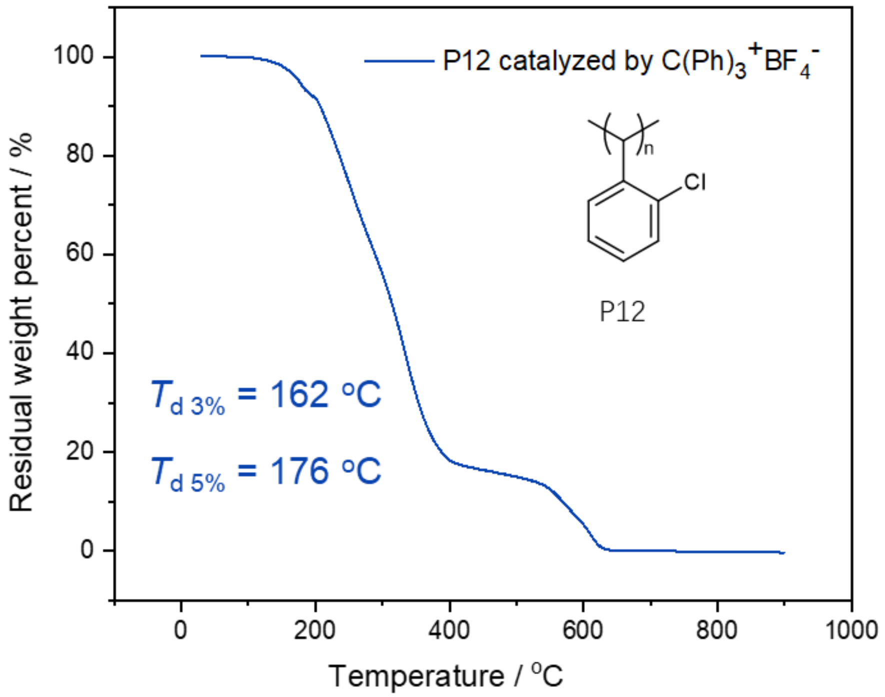

Fig. S217. TGA curve of polymer **P12** synthesized using $C(Ph)_3^+BF_4^-$ as a catalyst, recorded under a nitrogen atmosphere with a heating rate of 10 °C/min.

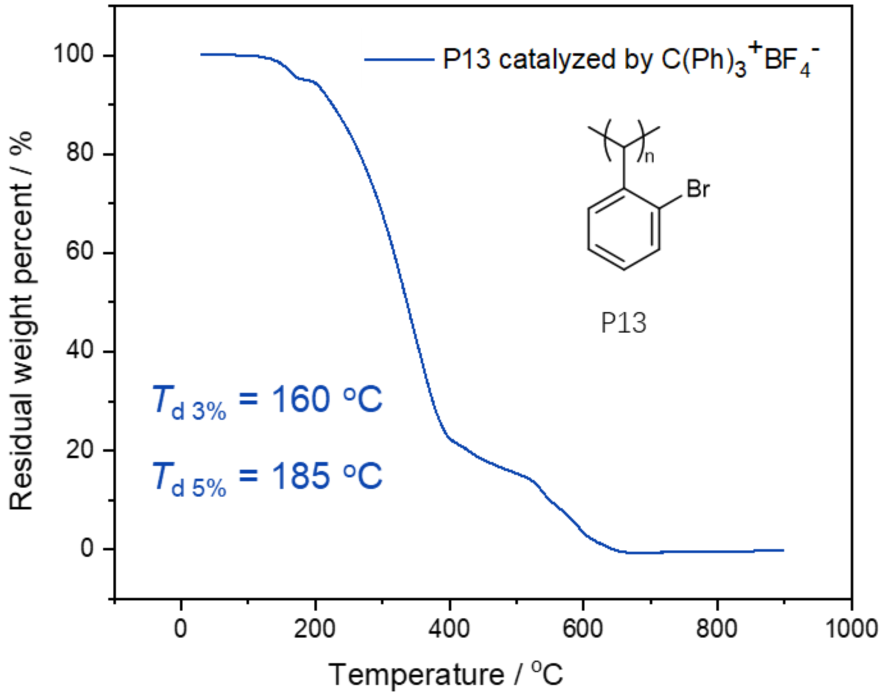

Fig. S218. TGA curve of polymer **P13** synthesized using $C(Ph)_3^+BF_4^-$ as a catalyst, recorded under a nitrogen atmosphere with a heating rate of 10 °C/min.

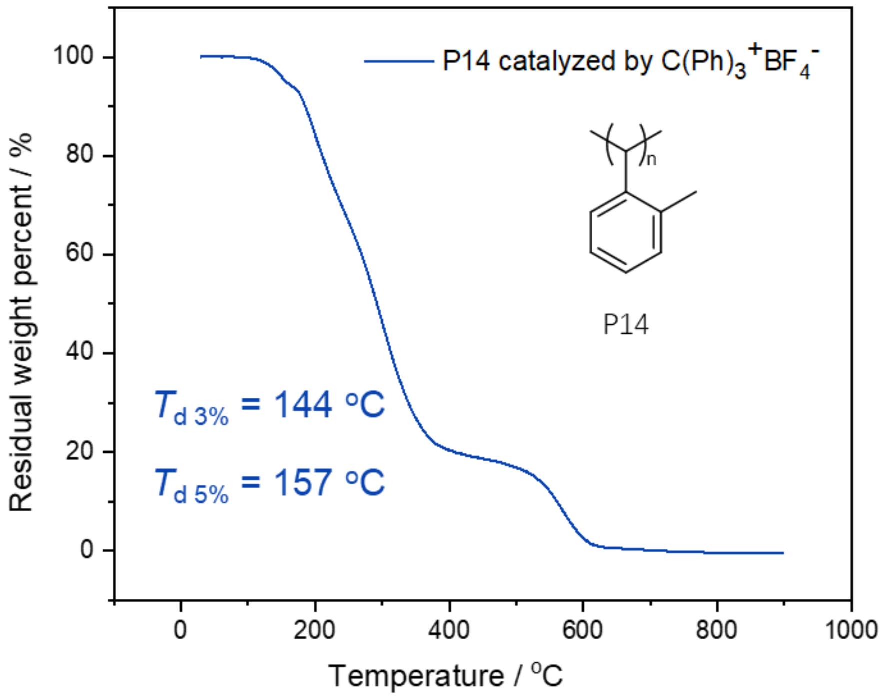

Fig. S219. TGA curve of polymer **P14** synthesized using $C(Ph)_3^+BF_4^-$ as a catalyst, recorded under a nitrogen atmosphere with a heating rate of 10 °C/min.

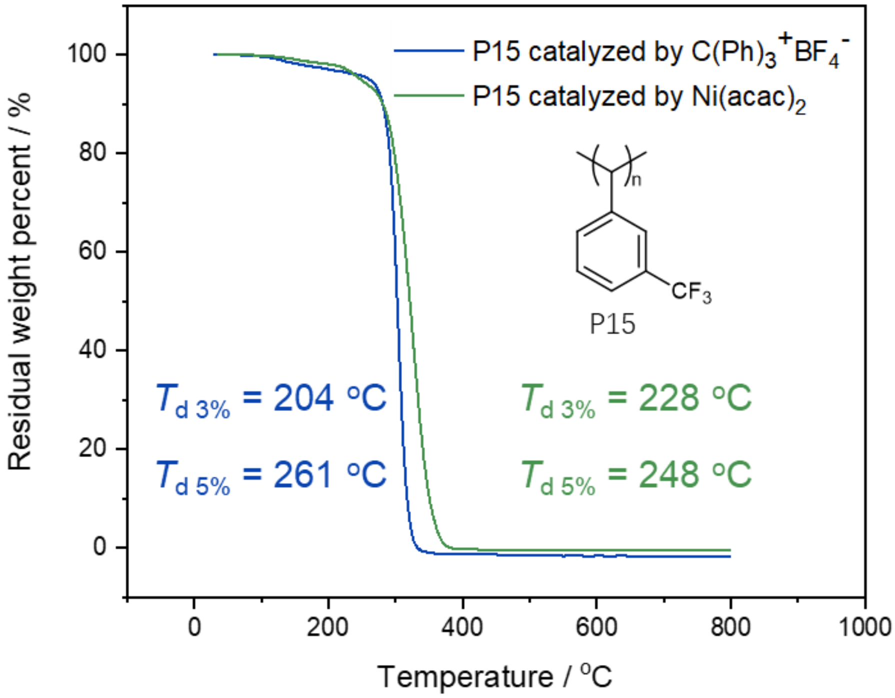

Fig. S220. TGA curves of polymer **P15** synthesized using $C(Ph)_3^+BF_4^-$ (blue) and $Ni(acac)_2$ (green) as catalysts, recorded under a nitrogen atmosphere with a heating rate of 10 °C/min.

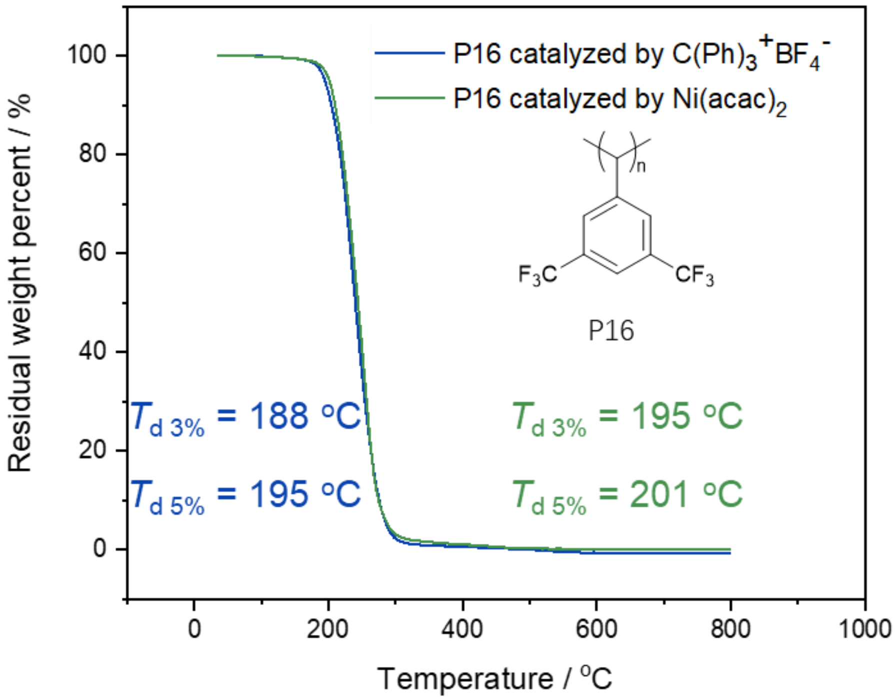

Fig. S221. TGA curves of polymer **P16** synthesized using $C(Ph)_3^+BF_4^-$ (blue) and $Ni(acac)_2$ (green) as catalysts, recorded under a nitrogen atmosphere with a heating rate of 10 °C/min.

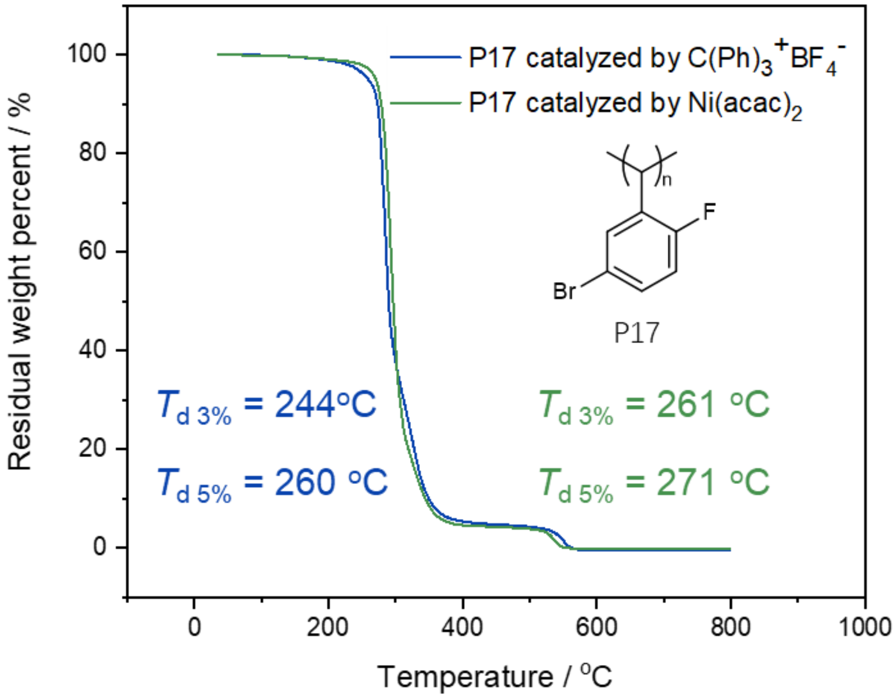

Fig. S222. TGA curves of polymer **P17** synthesized using $C(Ph)_3^+BF_4^-$ (blue) and $Ni(acac)_2$ (green) as catalysts, recorded under a nitrogen atmosphere with a heating rate of 10 °C/min.

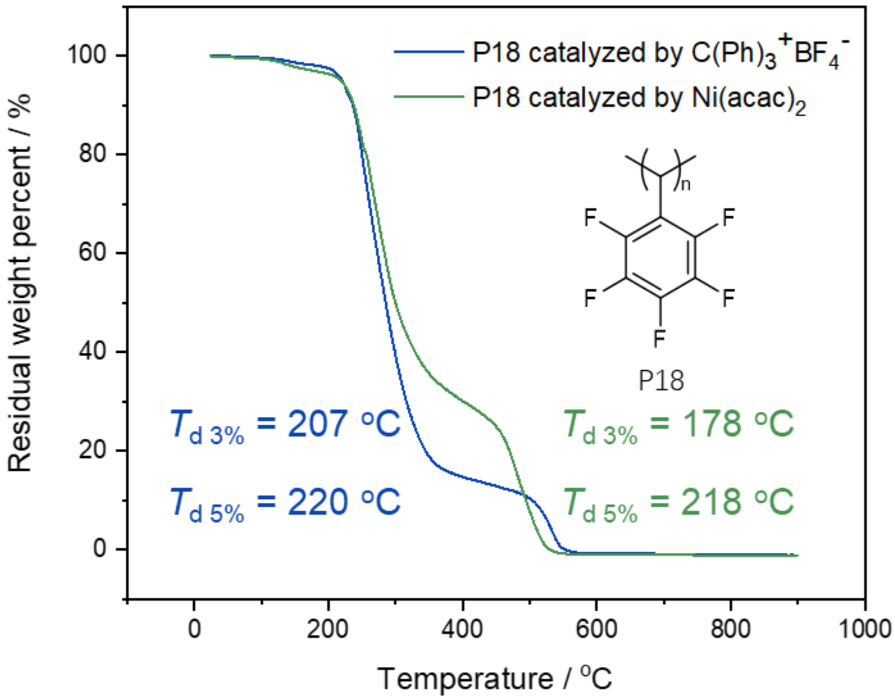

Fig. S223. TGA curves of polymer **P18** synthesized using $C(Ph)_3^+BF_4^-$ (blue) and $Ni(acac)_2$ (green) as catalysts, recorded under a nitrogen atmosphere with a heating rate of 10 °C/min.

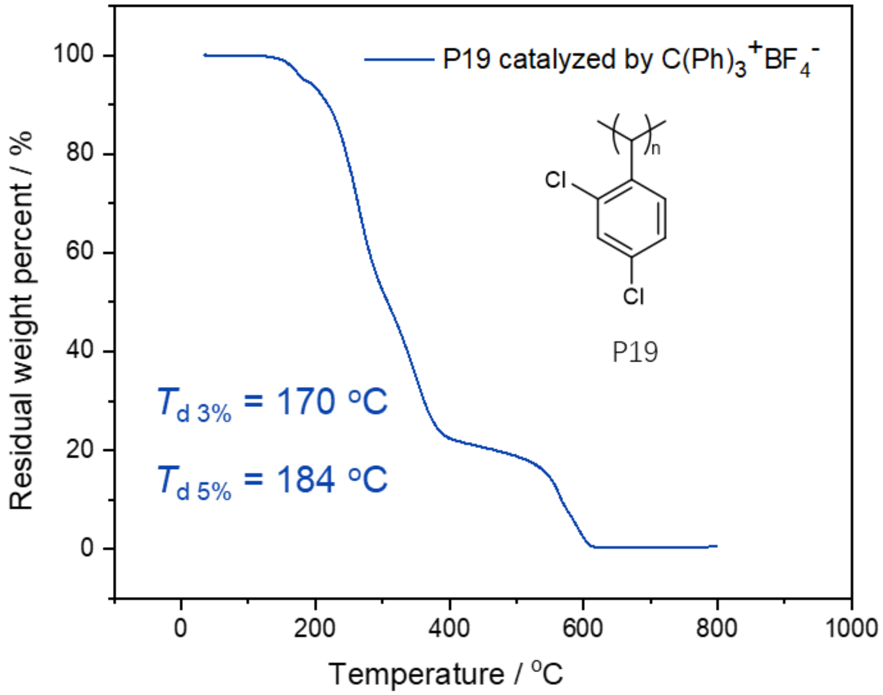

Fig. S224. TGA curve of polymer **P19** synthesized using $C(Ph)_3^+BF_4^-$ as a catalyst, recorded under a nitrogen atmosphere with a heating rate of 10 °C/min.

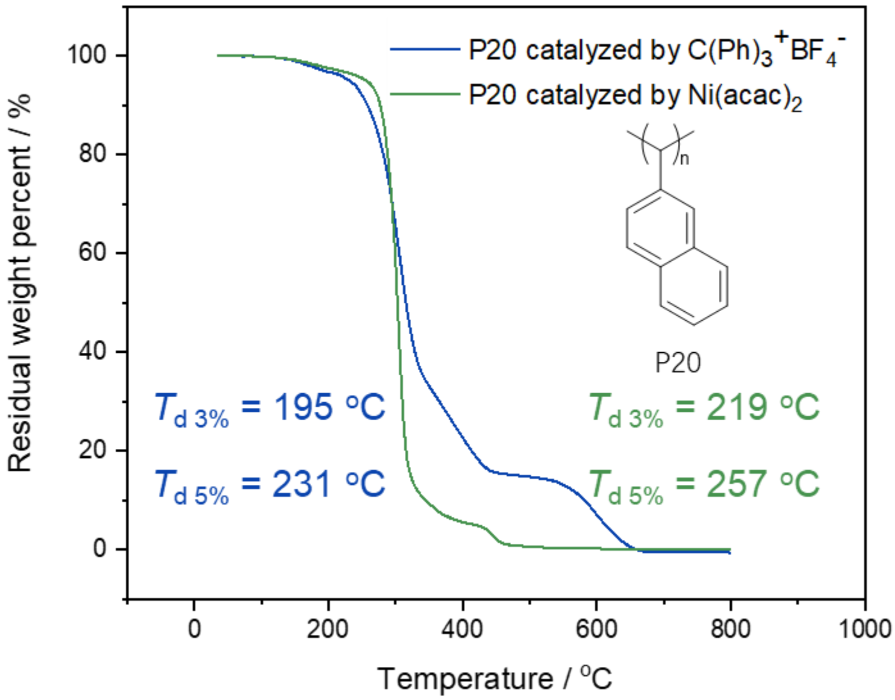

Fig. S225. TGA curves of polymer **P20** synthesized using $C(Ph)_3^+BF_4^-$ (blue) and $Ni(acac)_2$ (green) as catalysts, recorded under a nitrogen atmosphere with a heating rate of 10 °C/min.

DSC curves

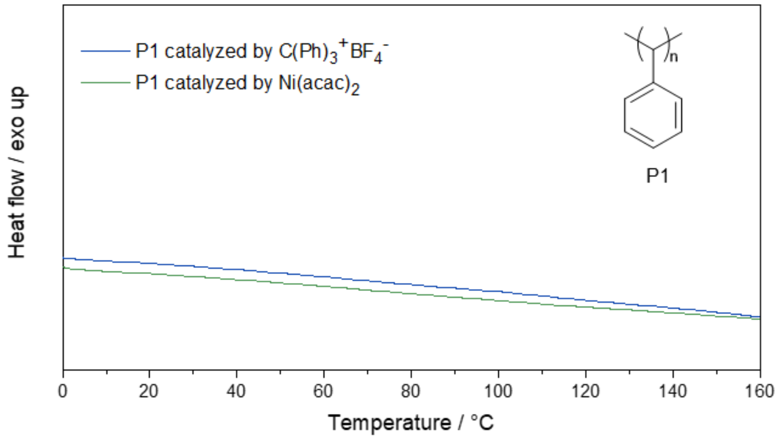

Fig. S226. Second-heating DSC scan curves (10 °C min^{-1}) of polymer **P1** synthesized using $C(Ph)_3^+BF_4^-$ (blue) and $Ni(acac)_2$ (green) as catalysts.

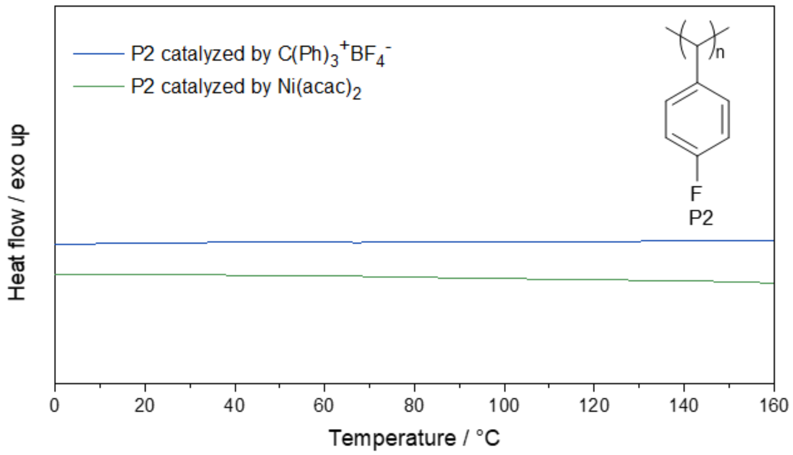

Fig. S227. Second-heating DSC scan curves (10 °C min^{-1}) of polymer **P2** synthesized using $C(Ph)_3^+BF_4^-$ (blue) and $Ni(acac)_2$ (green) as catalysts.

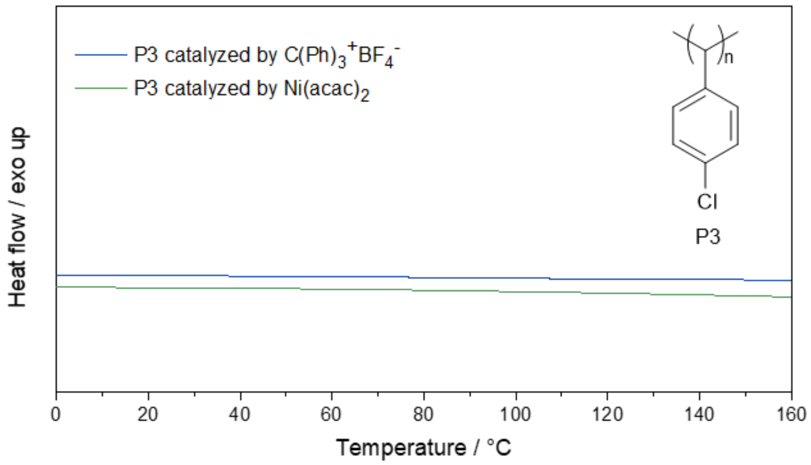

Fig. S228. Second-heating DSC scan curves (10 °C min^{-1}) of polymer **P3** synthesized using $C(Ph)_3^+BF_4^-$ (blue) and $Ni(acac)_2$ (green) as catalysts.

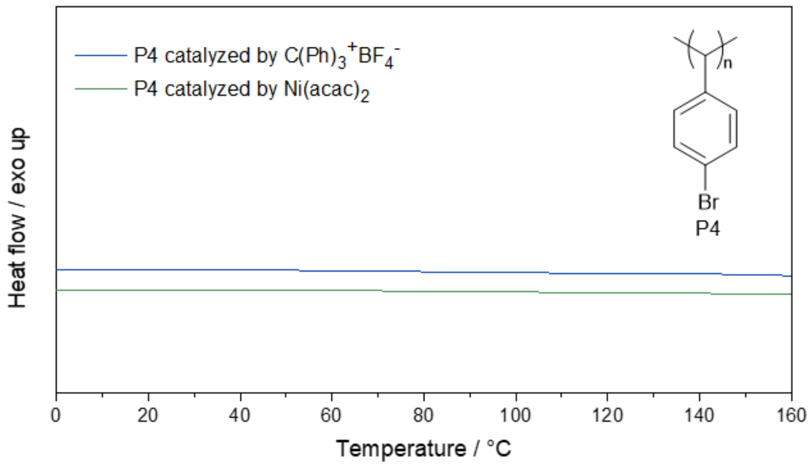

Fig. S229. Second-heating DSC scan curves (10 °C min^{-1}) of polymer **P4** synthesized using $C(Ph)_3^+BF_4^-$ (blue) and $Ni(acac)_2$ (green) as catalysts.

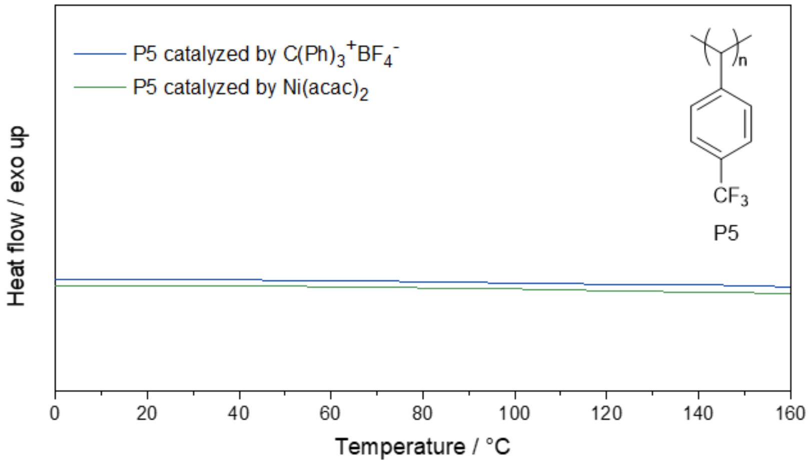

Fig. S230. Second-heating DSC scan curves (10 °C min^{-1}) of polymer **P5** synthesized using $C(Ph)_3^+BF_4^-$ (blue) and $Ni(acac)_2$ (green) as catalysts.

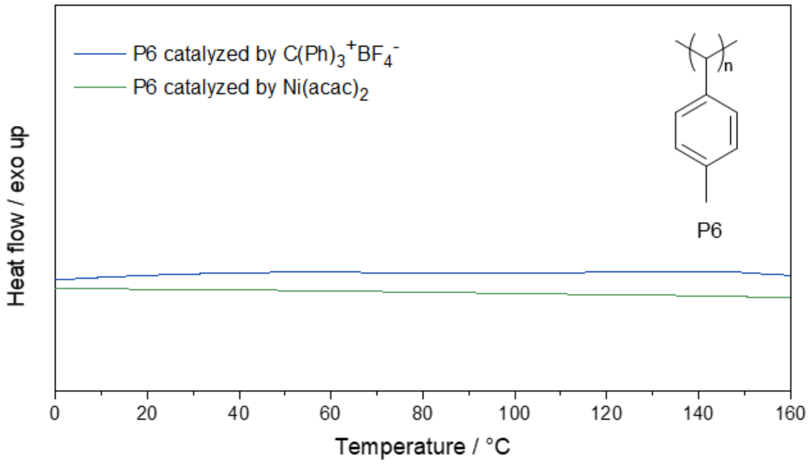

Fig. S231. Second-heating DSC scan curves (10 °C min^{-1}) of polymer **P6** synthesized using $C(Ph)_3^+BF_4^-$ (blue) and $Ni(acac)_2$ (green) as catalysts.

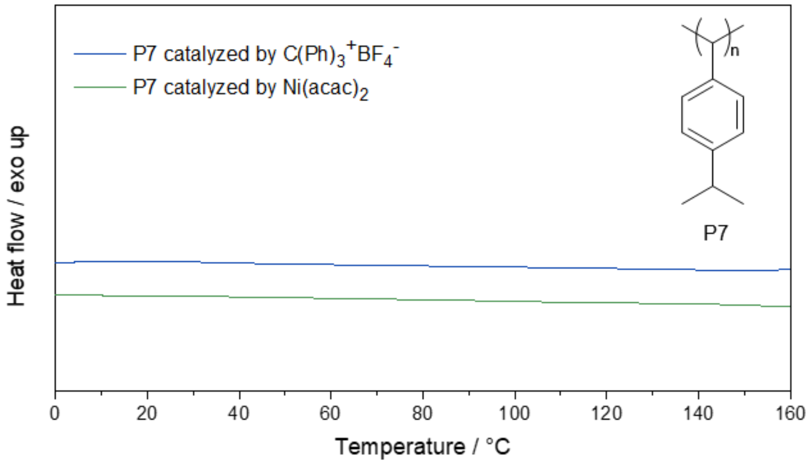

Fig. S232. Second-heating DSC scan curves (10 °C min^{-1}) of polymer **P7** synthesized using $C(Ph)_3^+BF_4^-$ (blue) and $Ni(acac)_2$ (green) as catalysts.

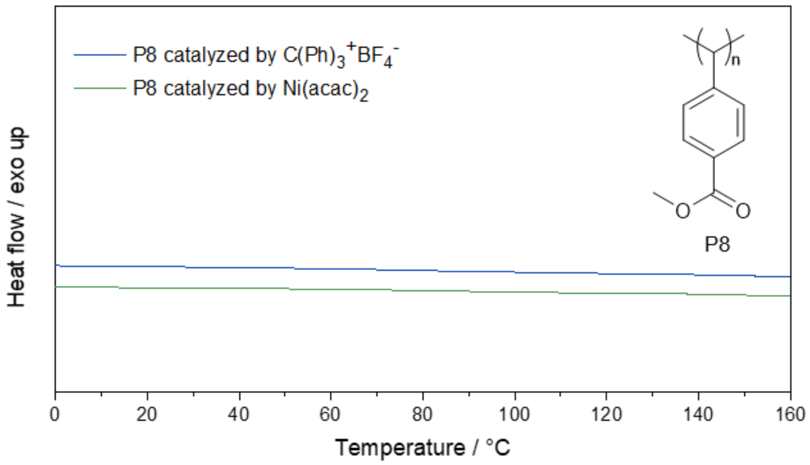

Fig. S233. Second-heating DSC scan curves (10 °C min^{-1}) of polymer **P8** synthesized using $C(Ph)_3^+BF_4^-$ (blue) and $Ni(acac)_2$ (green) as catalysts.

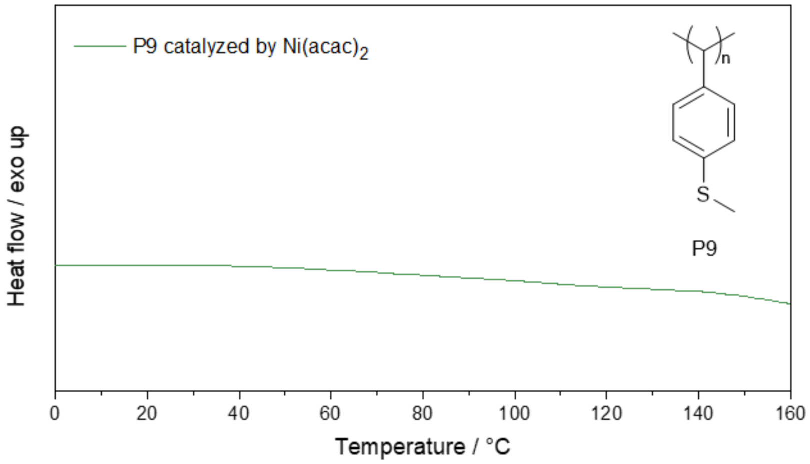

Fig. S234. Second-heating DSC scan curve (10 °C min^{-1}) of polymer **P9** synthesized using $Ni(acac)_2$ as a catalyst.

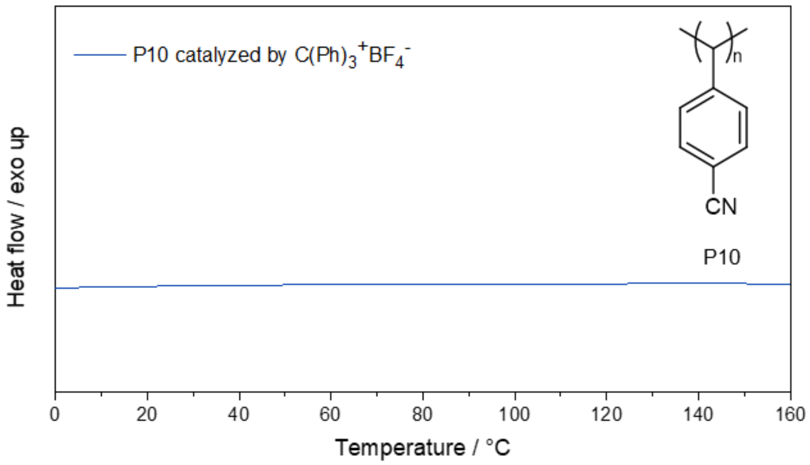

Fig. S235. Second-heating DSC scan curve (10 °C min^{-1}) of polymer **P10** synthesized using $C(Ph)_3^+BF_4^-$ as a catalyst.

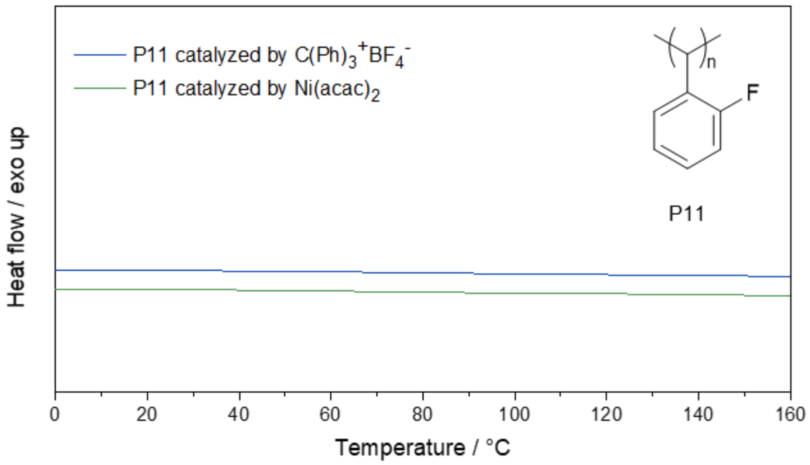

Fig. S236. Second-heating DSC scan curves (10 °C min^{-1}) of polymer **P11** synthesized using $C(Ph)_3^+BF_4^-$ (blue) and $Ni(acac)_2$ (green) as catalysts.

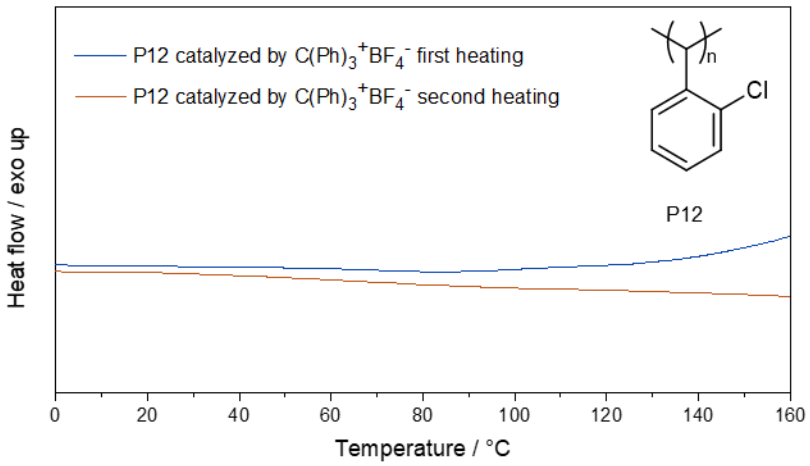

Fig. S237. First and second-heating DSC scan curves (10 °C min^{-1}) of polymer **P12** synthesized using $C(Ph)_3^+BF_4^-$ as a catalyst (Thermal degradation occurs during the first heating).

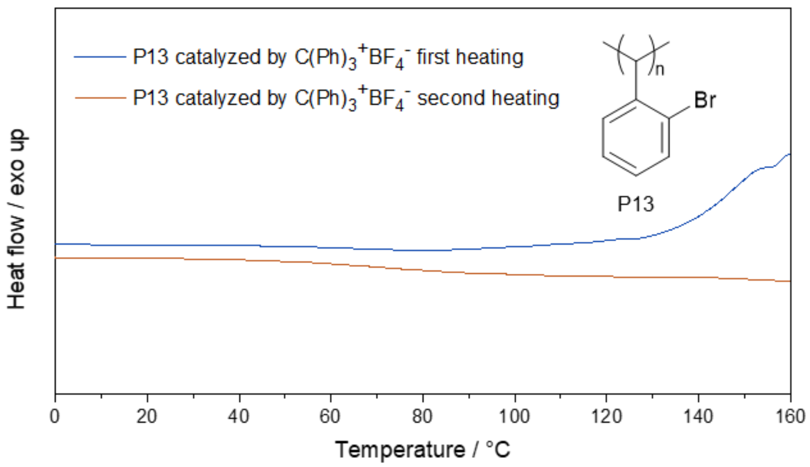

Fig. S238. First and second-heating DSC scan curves (10 °C min^{-1}) of polymer **P13** synthesized using $C(Ph)_3^+BF_4^-$ as a catalyst (Thermal degradation occurs during the first heating).

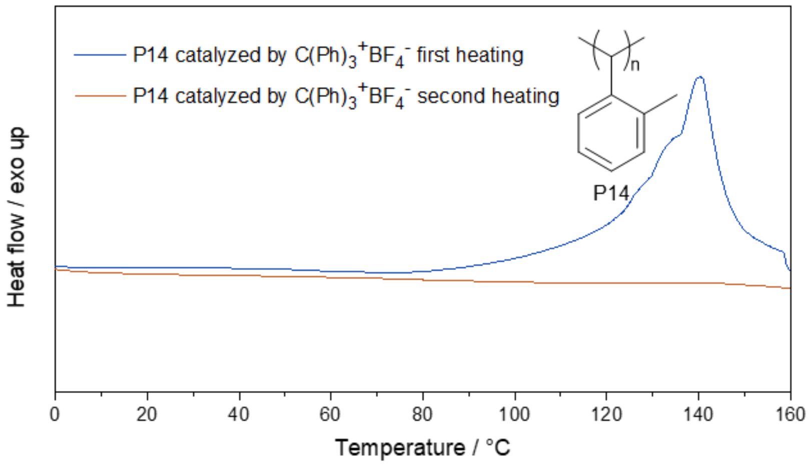

Fig. S239. First and second-heating DSC scan curves (10 °C min^{-1}) of polymer **P14** synthesized using $C(Ph)_3^+BF_4^-$ as a catalyst (Thermal degradation occurs during the first heating).

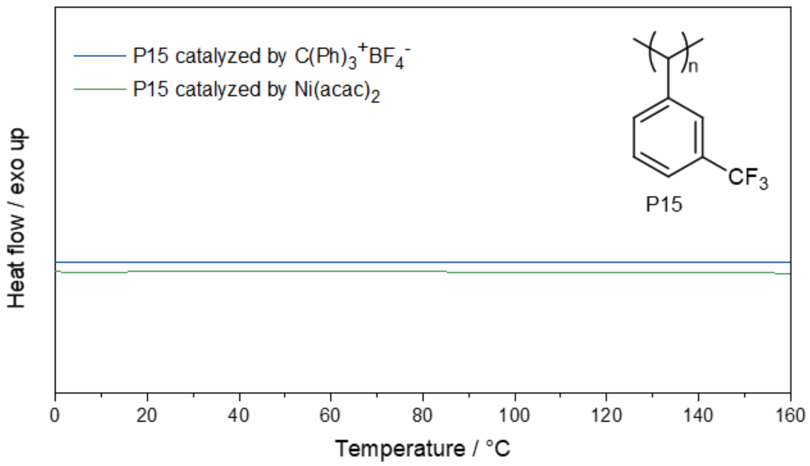

Fig. S240. Second-heating DSC scan curves (10 °C min^{-1}) of polymer **P15** synthesized using $C(Ph)_3^+BF_4^-$ (blue) and $Ni(acac)_2$ (green) as catalysts.

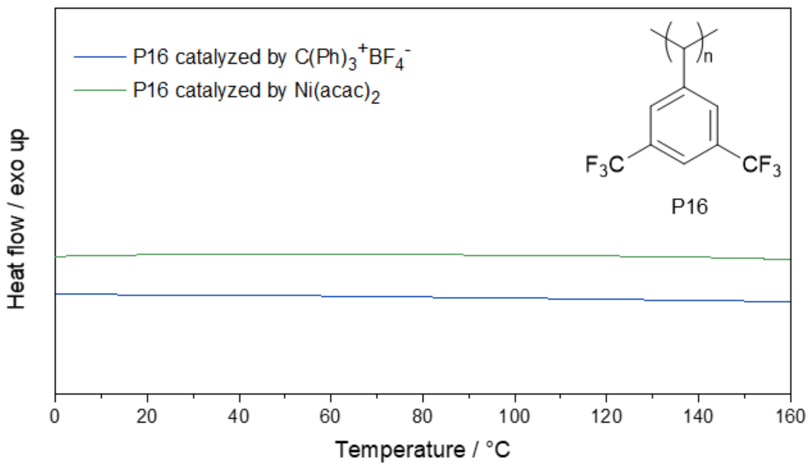

Fig. S241. Second-heating DSC scan curves (10 °C min^{-1}) of polymer **P16** synthesized using $C(Ph)_3^+BF_4^-$ (blue) and $Ni(acac)_2$ (green) as catalysts.

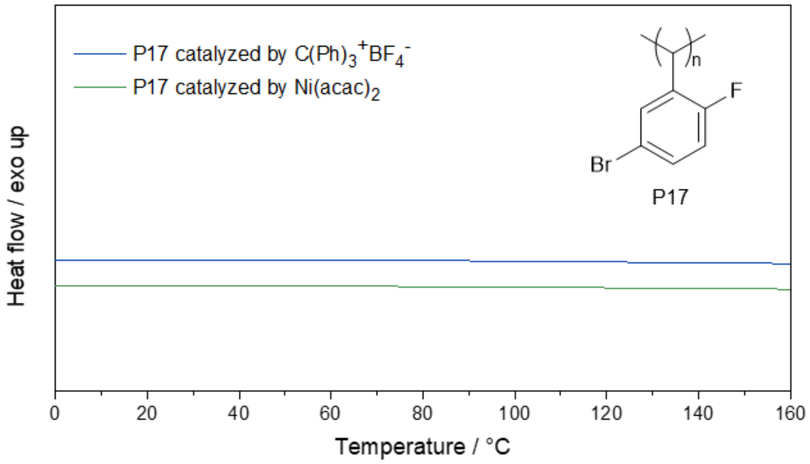

Fig. S242. Second-heating DSC scan curves (10 °C min^{-1}) of polymer **P17** synthesized using $C(Ph)_3^+BF_4^-$ (blue) and $Ni(acac)_2$ (green) as catalysts.

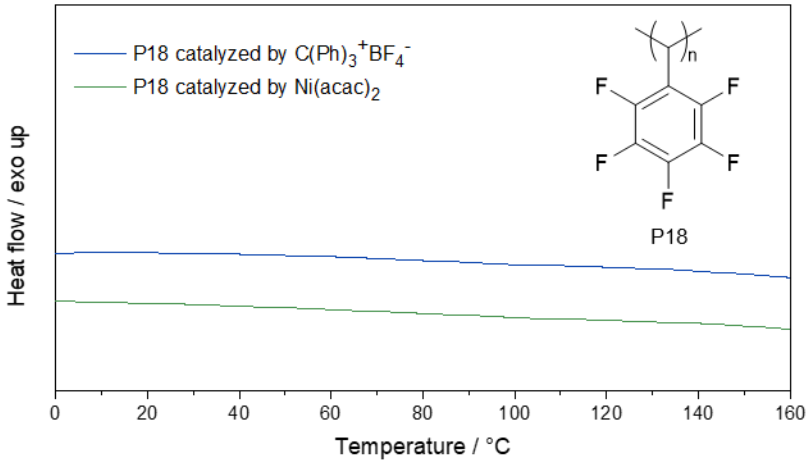

Fig. S243. Second-heating DSC scan curves (10 °C min^{-1}) of polymer **P18** synthesized using $C(Ph)_3^+BF_4^-$ (blue) and $Ni(acac)_2$ (green) as catalysts.

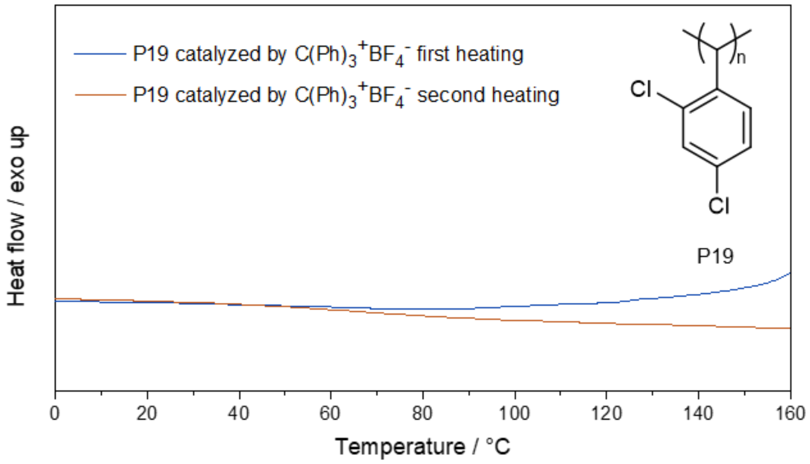

Fig. S244. First and second-heating DSC scan curves (10 °C min^{-1}) of polymer **P19** synthesized using $C(Ph)_3^+BF_4^-$ as a catalyst (Thermal degradation occurs during the first heating).

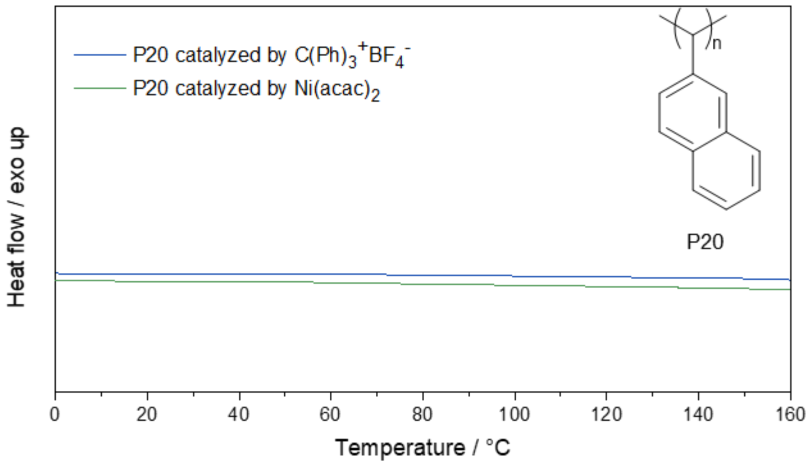

Fig. S245. Second-heating DSC scan curves (10 °C min^{-1}) of polymer **P20** synthesized using $C(Ph)_3^+BF_4^-$ (blue) and $Ni(acac)_2$ (green) as catalysts.

SEC traces

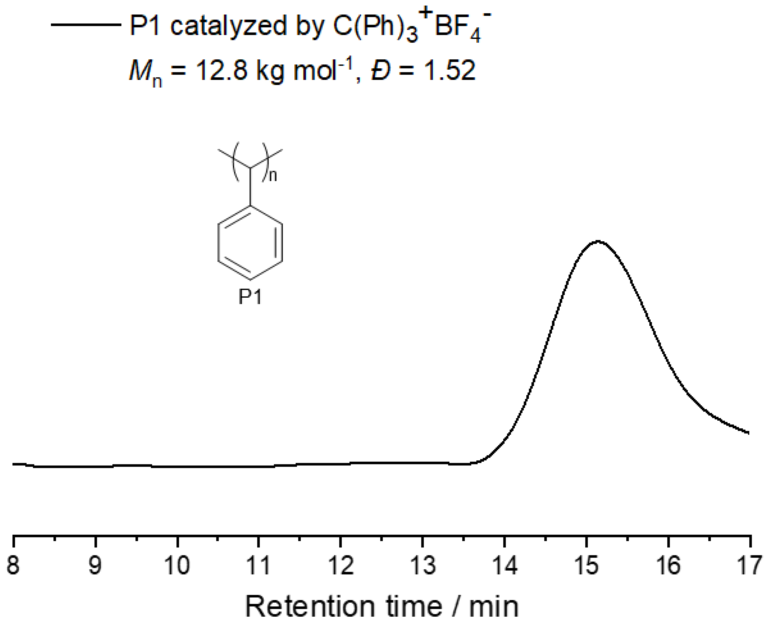

Fig. S246. SEC trace of polymer **P1** synthesized using $C(Ph)_3^+BF_4^-$ as a catalyst (Entry 1, Table S1).

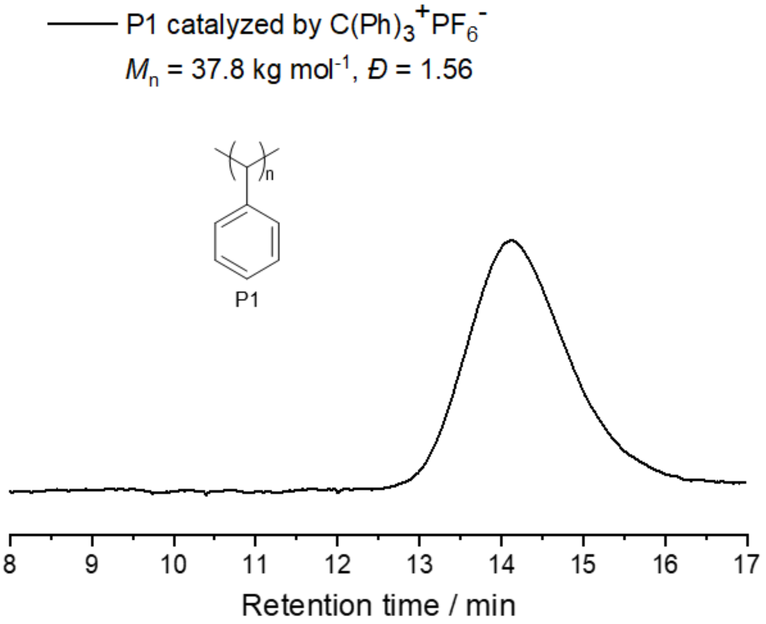

Fig. S247. SEC trace of polymer **P1** synthesized using $C(Ph)_3^+PF_6^-$ as a catalyst (Entry 2, Table S1).

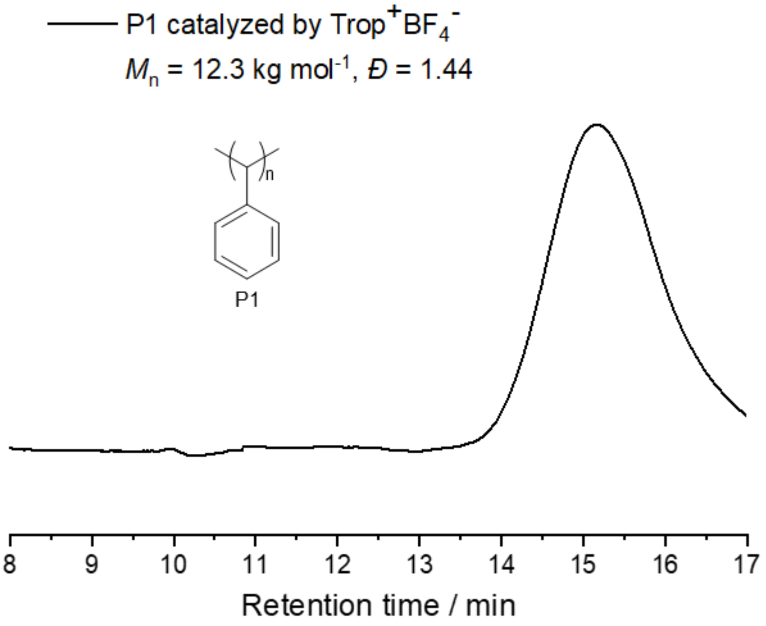

Fig. S248. SEC trace of polymer **P1** synthesized using $Trop^+BF_4^-$ as a catalyst (Entry 3, Table S1).

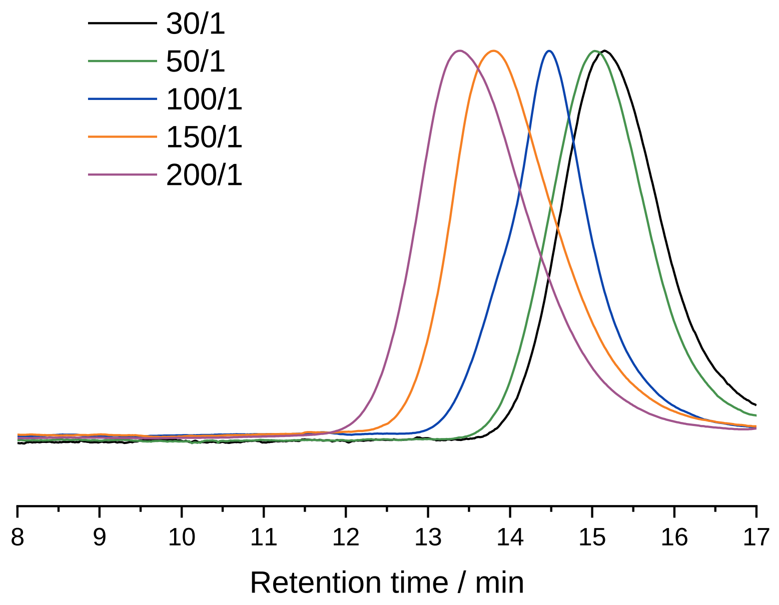

Fig. S249. SEC traces of polymer **P1** synthesized using $C(Ph)_3^+BF_4^-$ as a catalyst at the different [1]/[cat.] ratios (Entries 1, 5-8, Table S1).

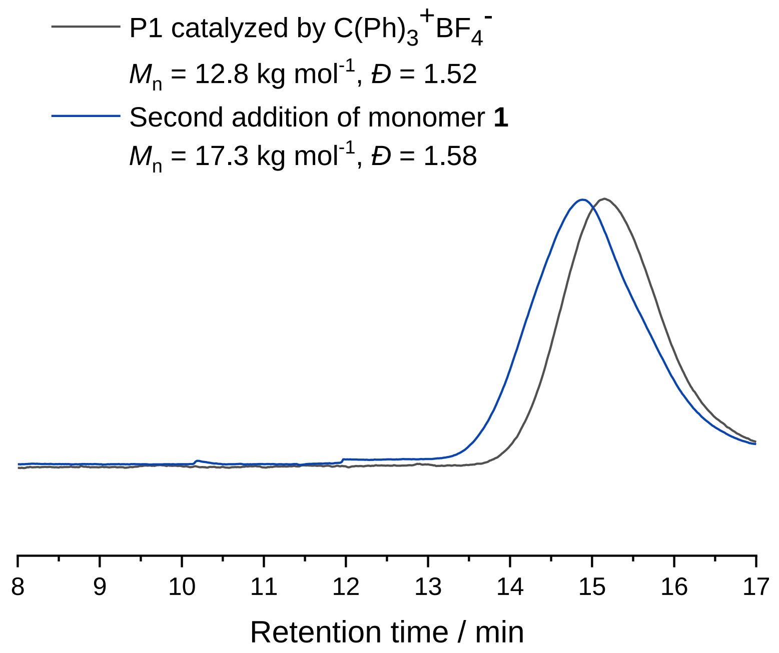

Fig. S250. SEC traces of polymer **P1** synthesized using $C(Ph)_3^+BF_4^-$ as a catalyst, before and after chain extension via a second monomer feed.

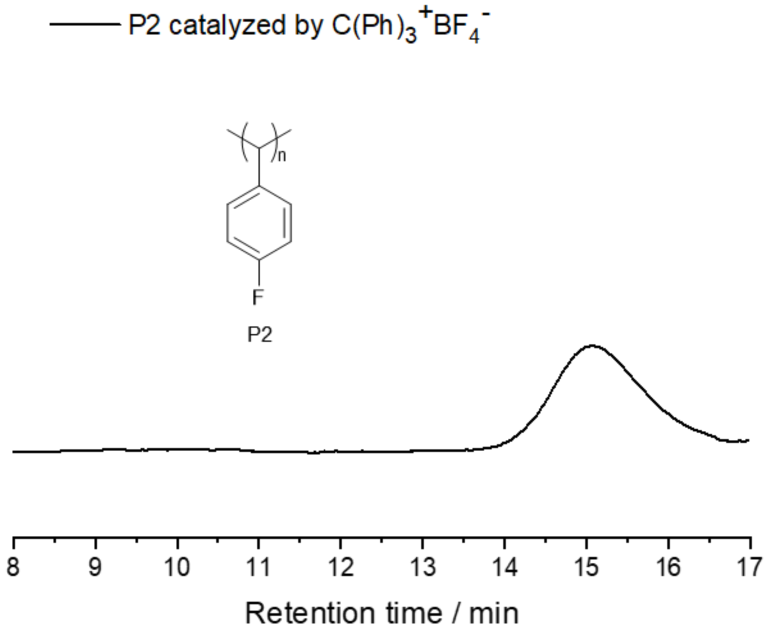

Fig. S251. SEC trace of polymer **P2** synthesized using $C(Ph)_3^+BF_4^-$ as a catalyst (Entry 2, Table S4).

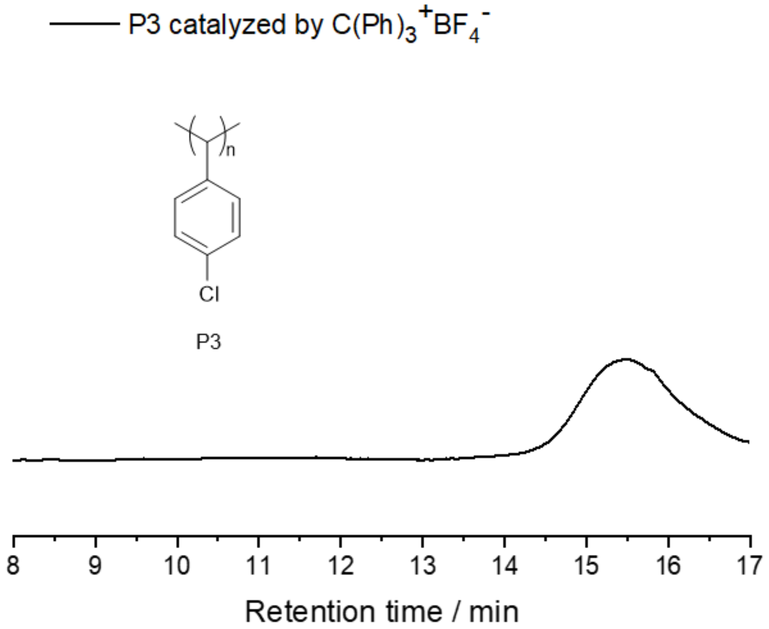

Fig. S252. SEC trace of polymer **P3** synthesized using $C(Ph)_3^+BF_4^-$ as a catalyst (Entry 3, Table S4).

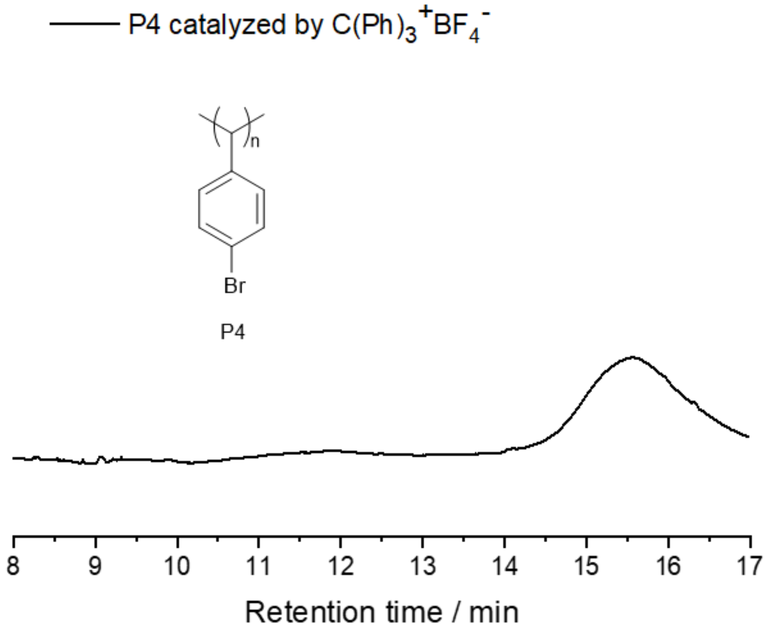

Fig. S253. SEC trace of polymer **P4** synthesized using $C(Ph)_3^+BF_4^-$ as a catalyst (Entry 4, Table S4).

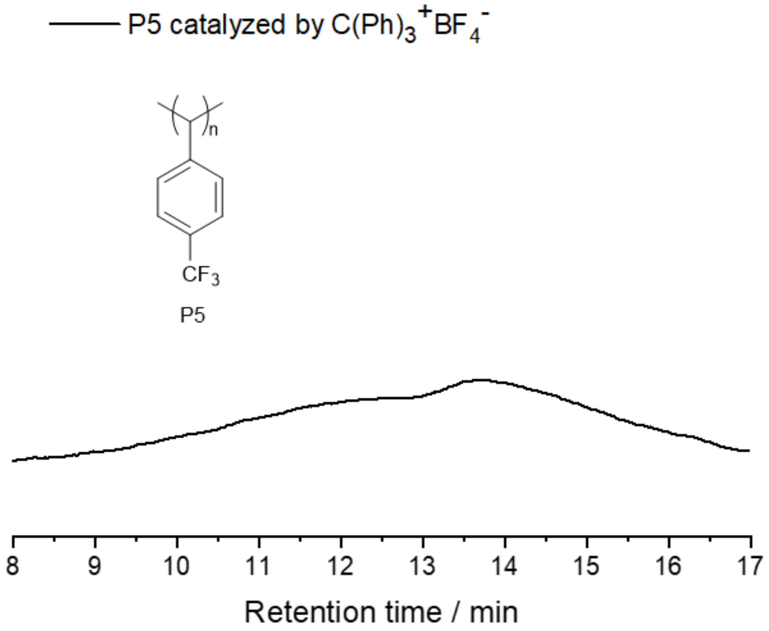

Fig. S254. SEC trace of polymer **P5** synthesized using $C(Ph)_3^+BF_4^-$ as a catalyst (The polymer exhibits poor solubility in THF) (Entry 5, Table S4).

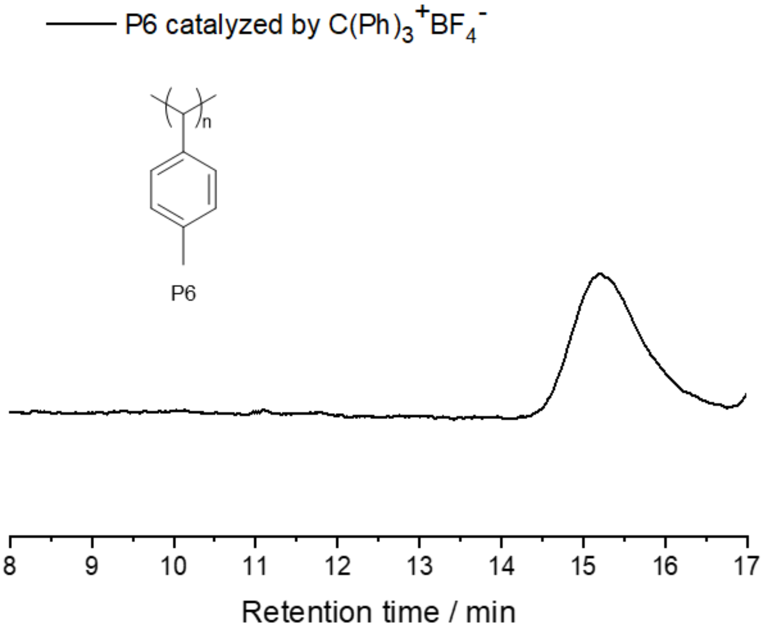

Fig. S255. SEC trace of polymer **P6** synthesized using $C(Ph)_3^+BF_4^-$ as a catalyst (Entry 6, Table S4).

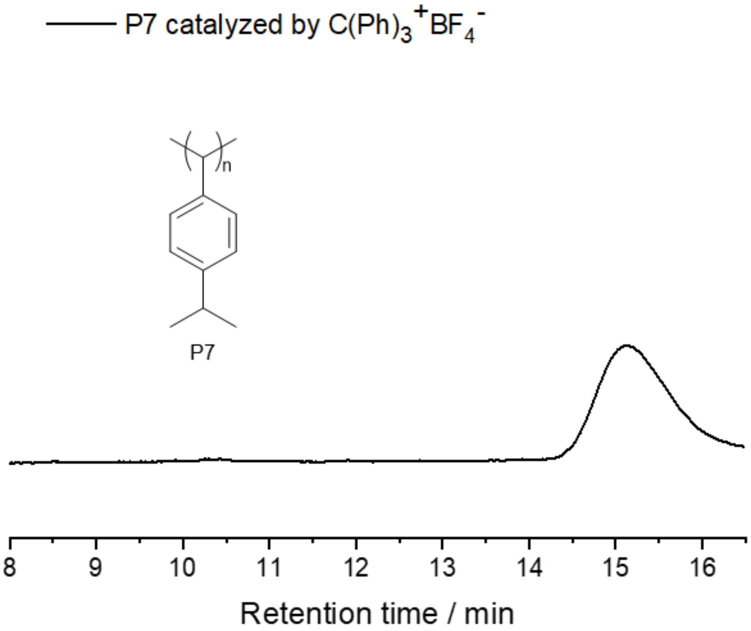

Fig. S256. SEC trace of polymer **P7** synthesized using $C(Ph)_3^+BF_4^-$ as a catalyst (Entry 7, Table S4).

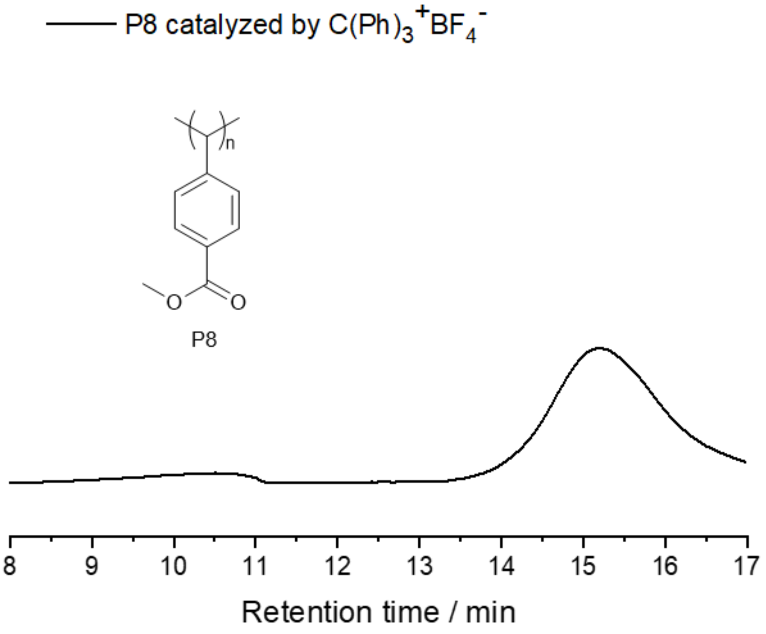

Fig. S257. SEC trace of polymer **P8** synthesized using $C(Ph)_3^+BF_4^-$ as a catalyst (Entry 8, Table S4).

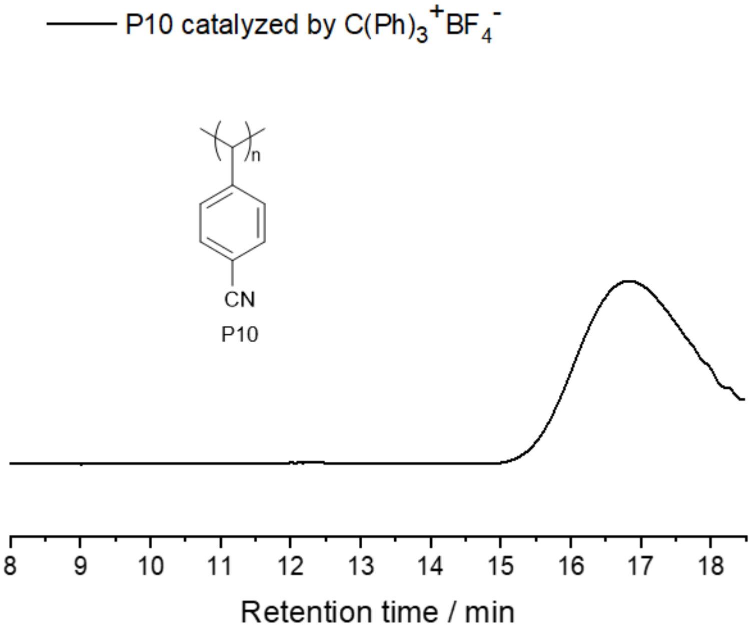

Fig. S258. SEC trace of polymer **P10** synthesized using $C(Ph)_3^+BF_4^-$ as a catalyst (Entry 10, Table S4).

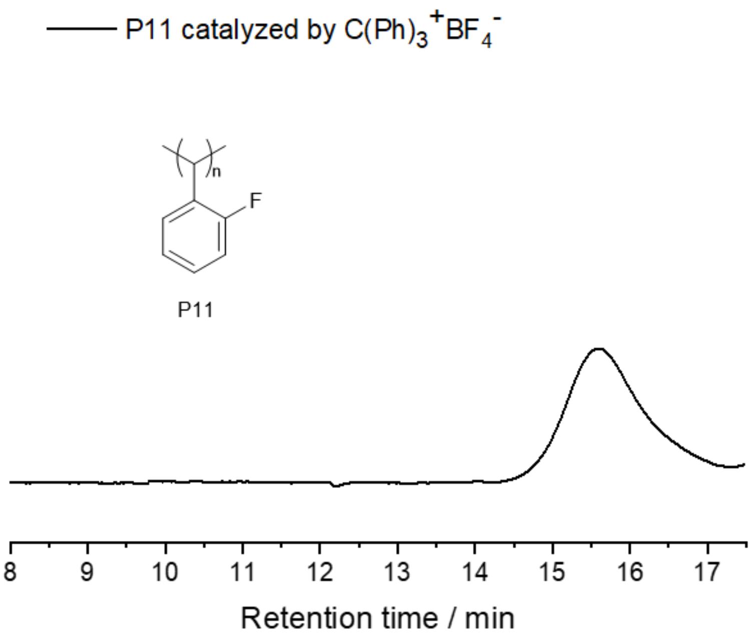

Fig. S259. SEC trace of polymer **P11** synthesized using $C(Ph)_3^+BF_4^-$ as a catalyst (Entry 11, Table S4).

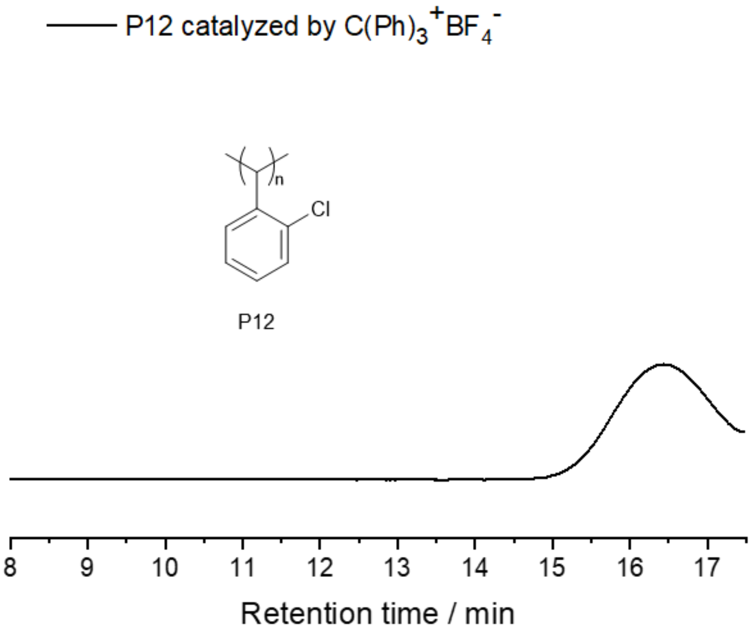

Fig. S260. SEC trace of polymer **P12** synthesized using $C(Ph)_3^+BF_4^-$ as a catalyst (Entry 12, Table S4).

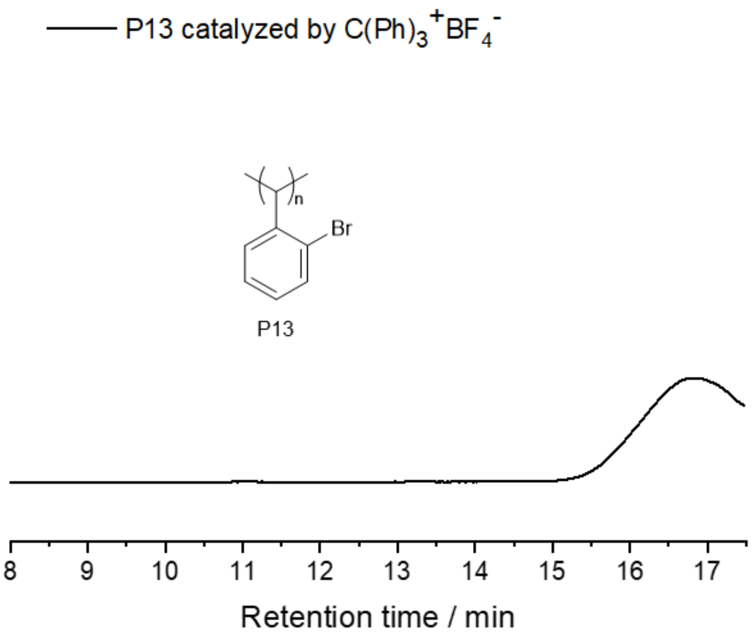

Fig. S261. SEC trace of polymer **P13** synthesized using $C(Ph)_3^+BF_4^-$ as a catalyst (Entry 13, Table S4).

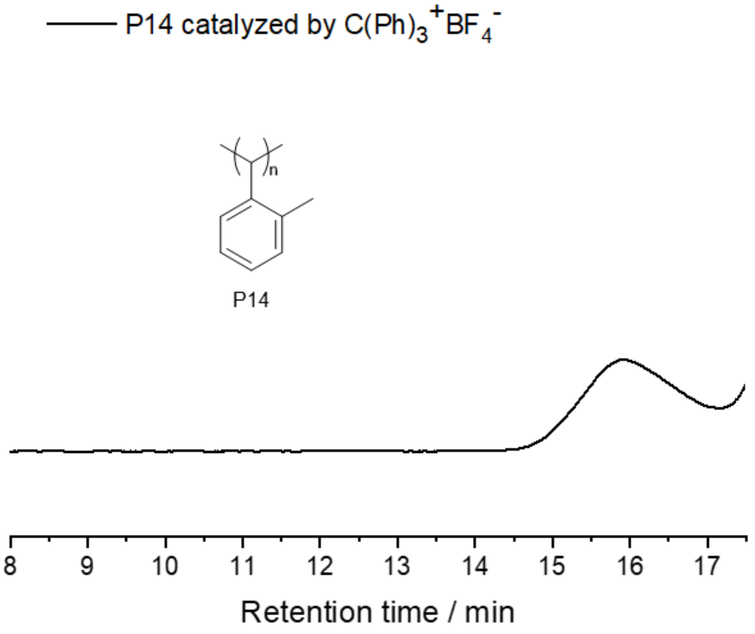

Fig. S262. SEC trace of polymer **P14** synthesized using $C(Ph)_3^+BF_4^-$ as a catalyst (Entry 14, Table S4).

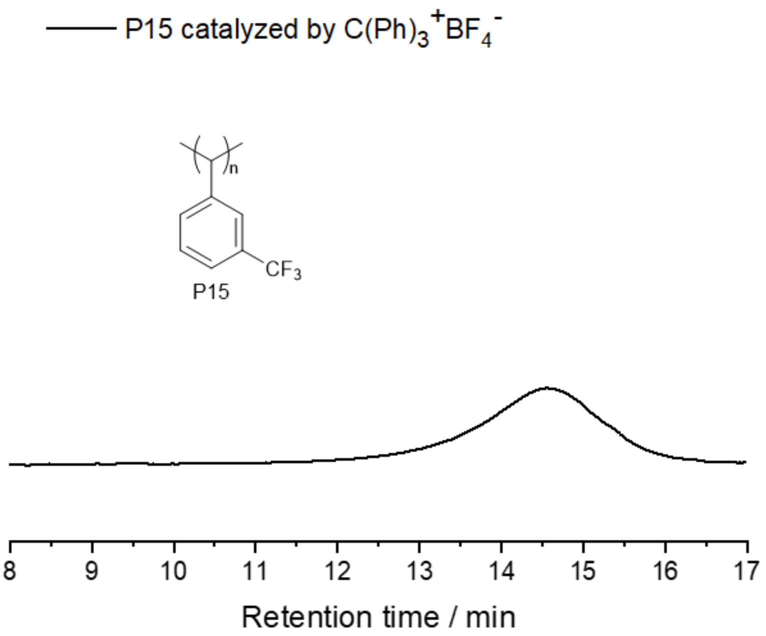

Fig. S263. SEC trace of polymer **P15** synthesized using $C(Ph)_3{}^+BF_4{}^-$ as a catalyst (The polymer exhibits poor solubility in THF) (Entry 15, Table S4).

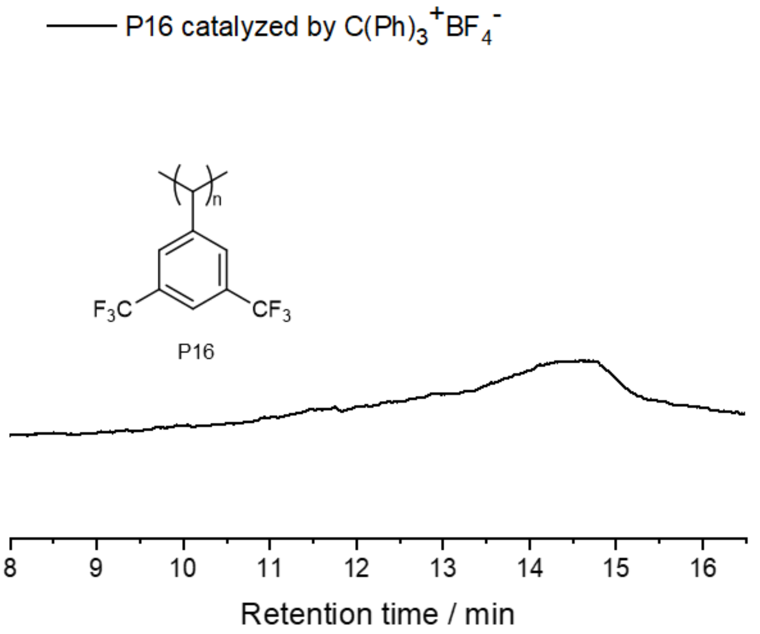

Fig. S264. SEC trace of polymer **P16** synthesized using $C(Ph)_3{}^+BF_4{}^-$ as a catalyst (The polymer exhibits poor solubility in THF) (Entry 16, Table S4).

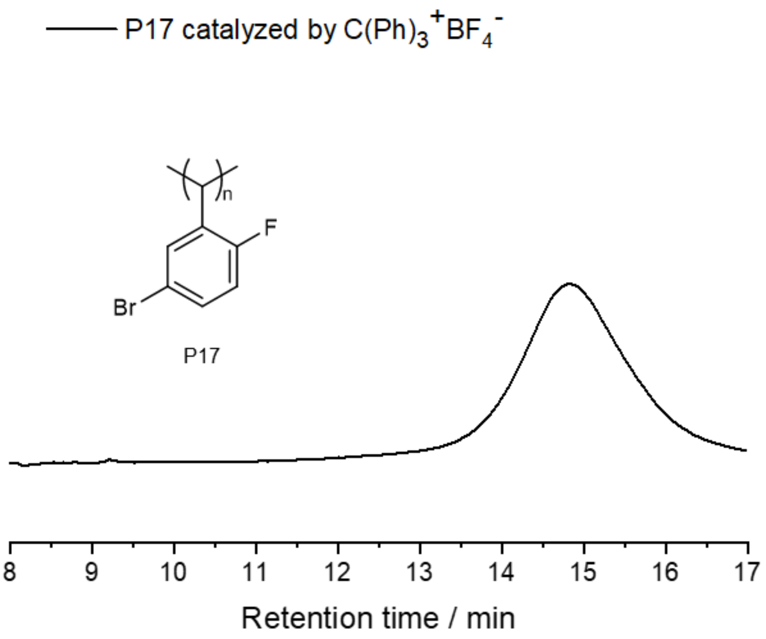

Fig. S265. SEC trace of polymer **P17** synthesized using $C(Ph)_3^+BF_4^-$ as a catalyst (Entry 17, Table S4).

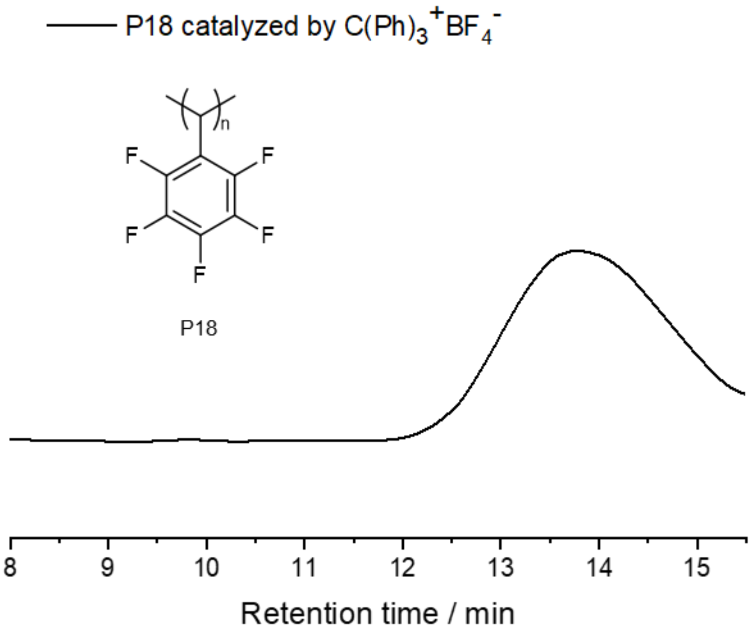

Fig. S266. SEC trace of polymer **P18** synthesized using $C(Ph)_3^+BF_4^-$ as a catalyst (The polymer exhibits poor solubility in THF) (Entry 18, Table S4).

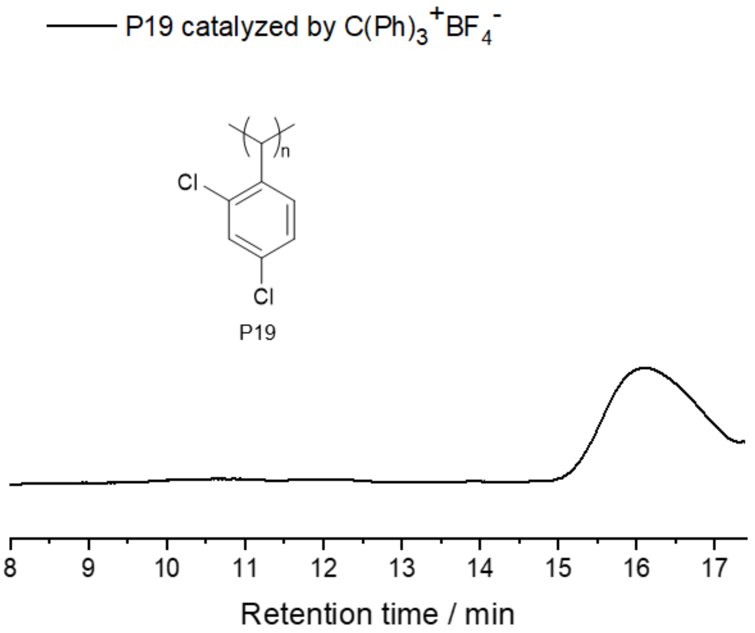

Fig. S267. SEC trace of polymer **P19** synthesized using $C(Ph)_3{}^+BF_4{}^-$ as a catalyst (Entry 19, Table S4).

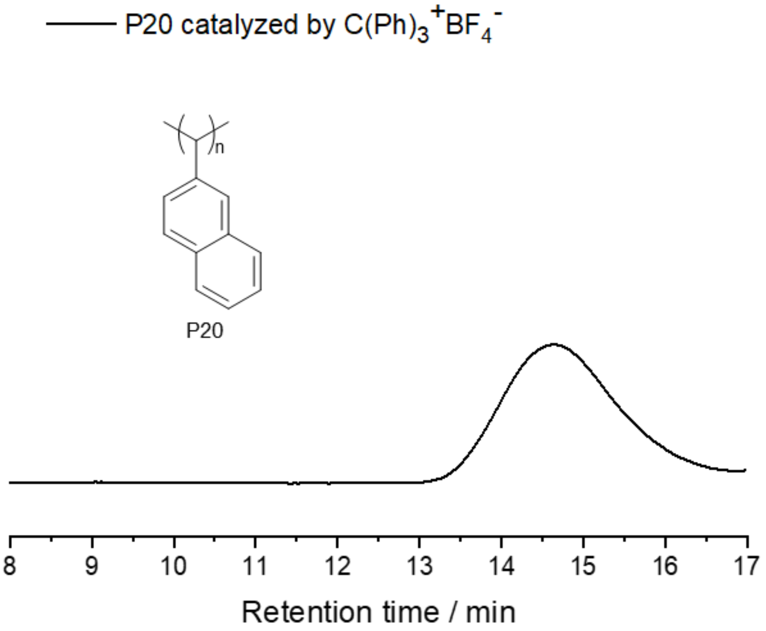

Fig. S268. SEC trace of polymer **P20** synthesized using $C(Ph)_3{}^+BF_4{}^-$ as a catalyst (Entry 20, Table S4).

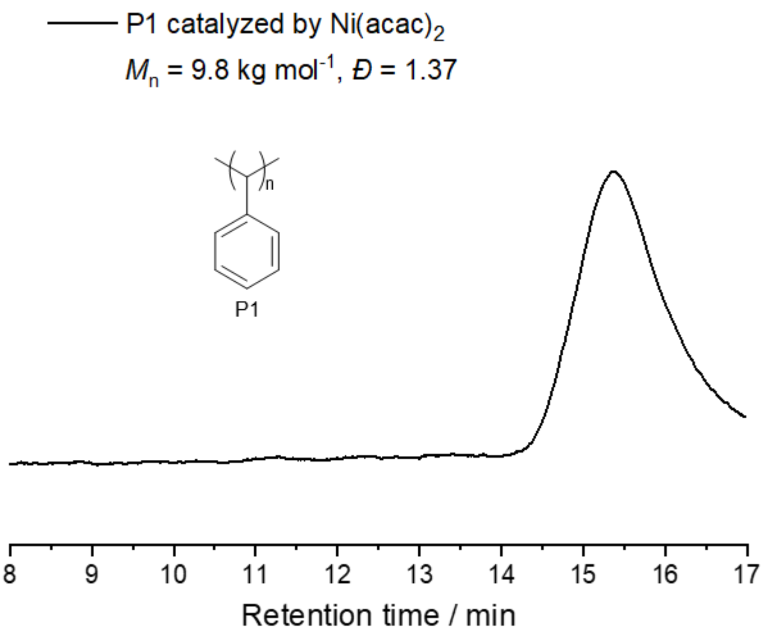

Fig. S269. SEC trace of polymer **P1** synthesized using Ni(acac)$_2$ as a catalyst (The polymer exhibits poor solubility in THF) (Entry 2, Table S2).

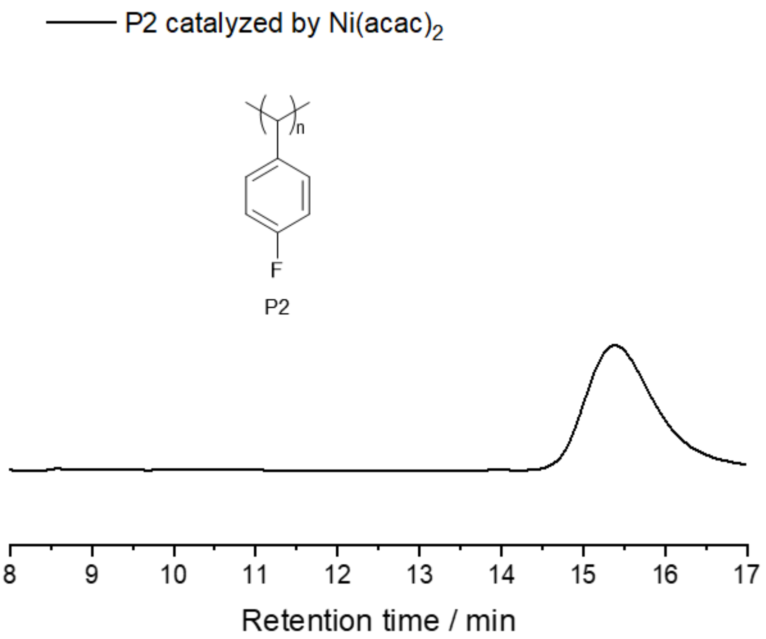

Fig. S270. SEC trace of polymer **P2** synthesized using Ni(acac)$_2$ as a catalyst (The polymer exhibits poor solubility in THF) (Entry 2, Table S5).

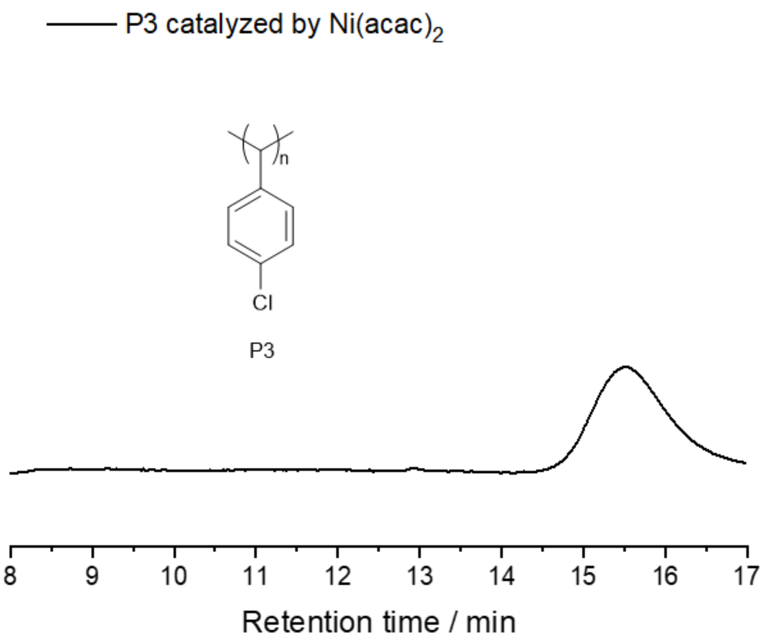

Fig. S271. SEC trace of polymer **P3** synthesized using $Ni(acac)_2$ as a catalyst (Entry 3, Table S5).

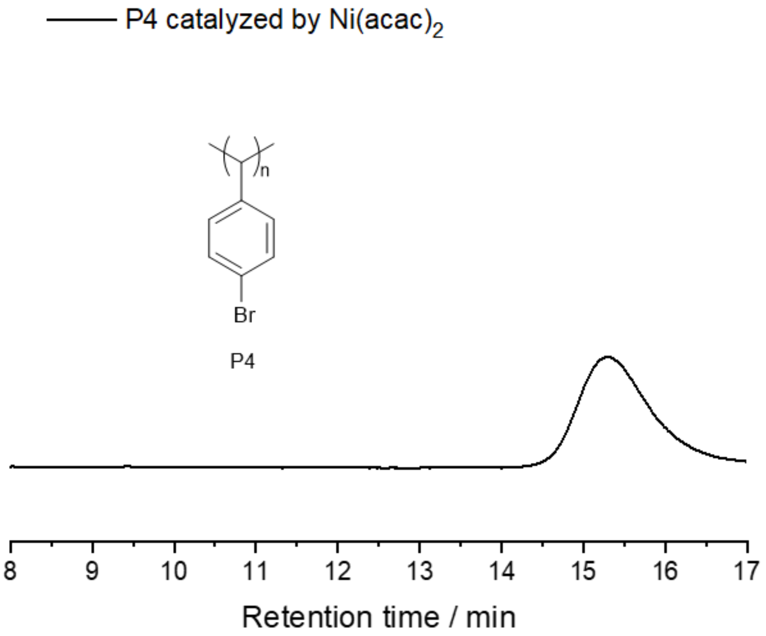

Fig. S272. SEC trace of polymer **P4** synthesized using $Ni(acac)_2$ as a catalyst (Entry 4, Table S5).

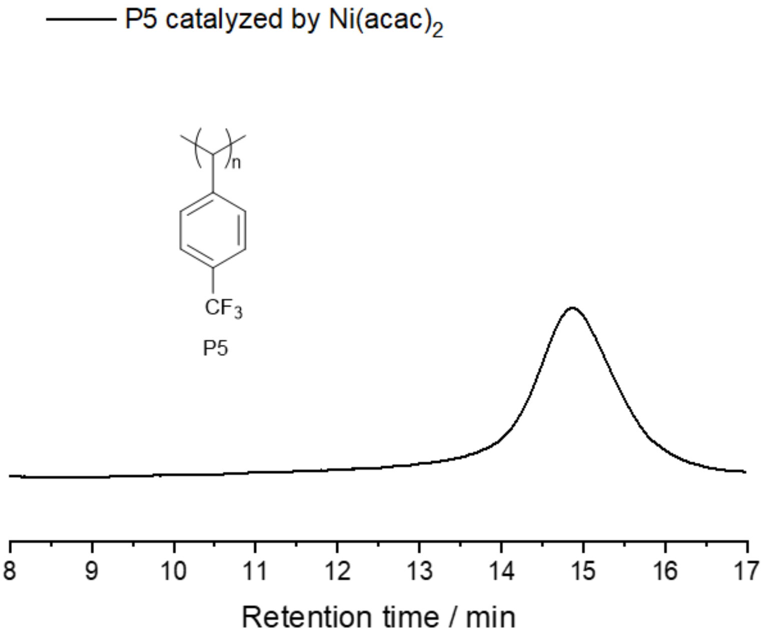

Fig. S273. SEC trace of polymer **P5** synthesized using Ni(acac)$_2$ as a catalyst (The polymer exhibits poor solubility in THF) (Entry 5, Table S5).

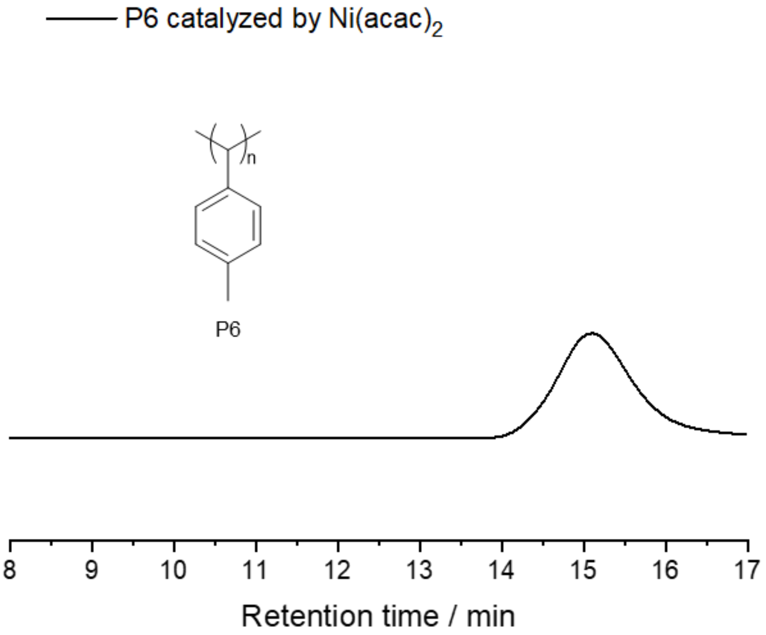

Fig. S274. SEC trace of polymer **P6** synthesized using Ni(acac)$_2$ as a catalyst (Entry 6, Table S5).

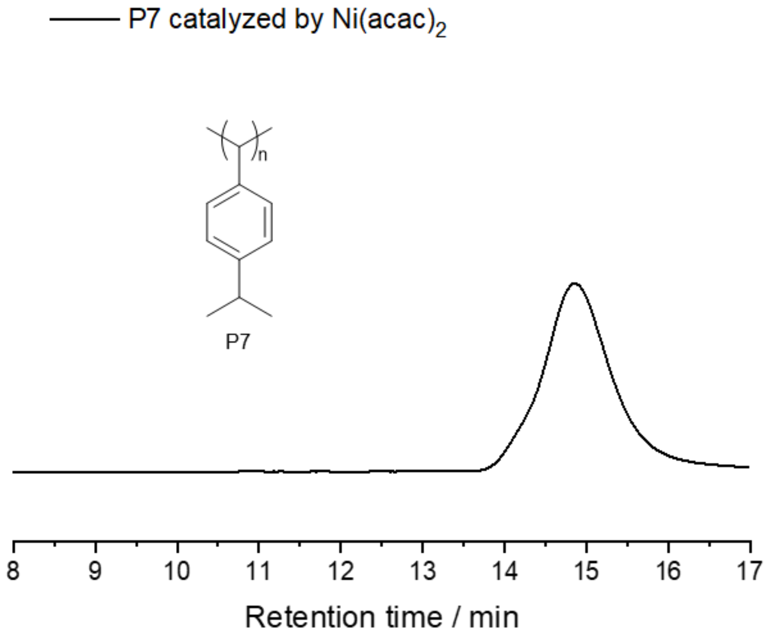

Fig. S275. SEC trace of polymer **P7** synthesized using Ni(acac)$_2$ as a catalyst (Entry 7, Table S5).

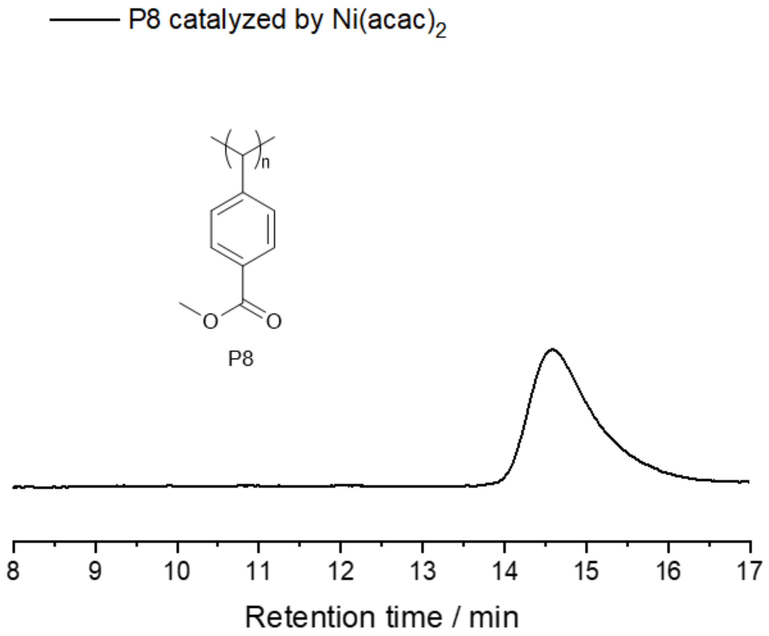

Fig. S276. SEC trace of polymer **P8** synthesized using Ni(acac)$_2$ as a catalyst (The polymer exhibits poor solubility in THF) (Entry 8, Table S5).

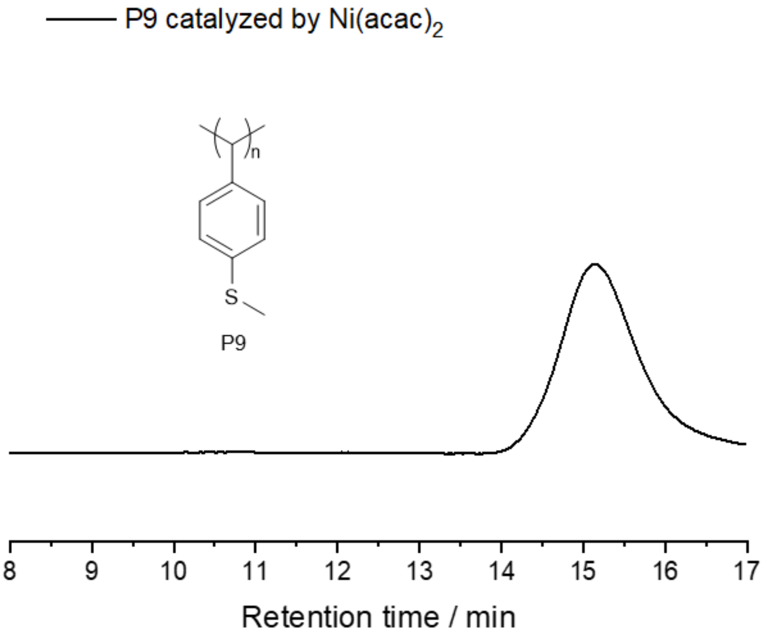

Fig. S277. SEC trace of polymer **P9** synthesized using Ni(acac)$_2$ as a catalyst (Entry 9, Table S5).

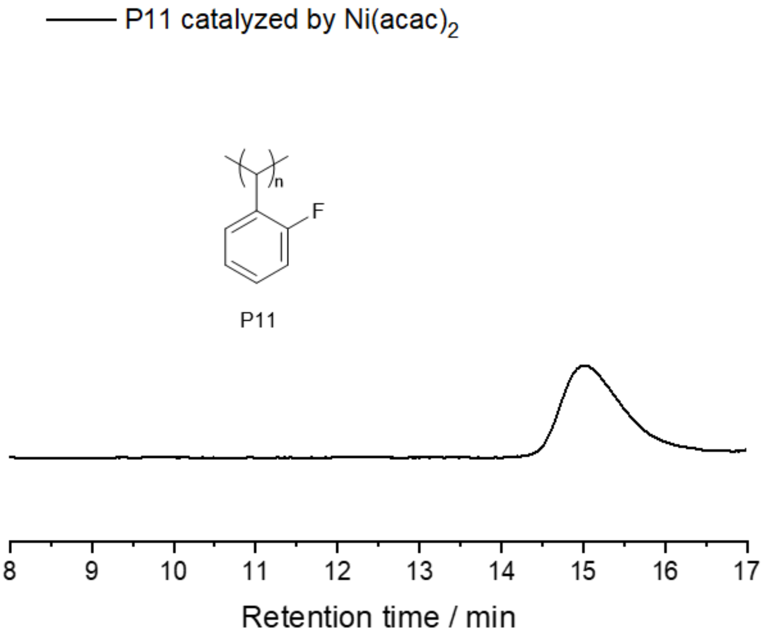

Fig. S278. SEC trace of polymer **P11** synthesized using Ni(acac)$_2$ as a catalyst (The polymer exhibits poor solubility in THF) (Entry 11, Table S5).

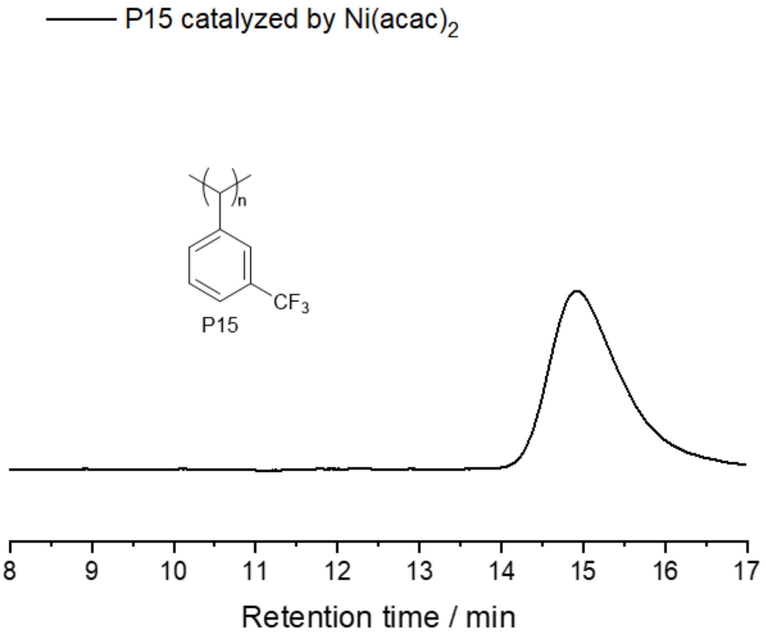

Fig. S279. SEC trace of polymer **P15** synthesized using Ni(acac)$_2$ as a catalyst (The polymer exhibits poor solubility in THF) (Entry 15, Table S5).

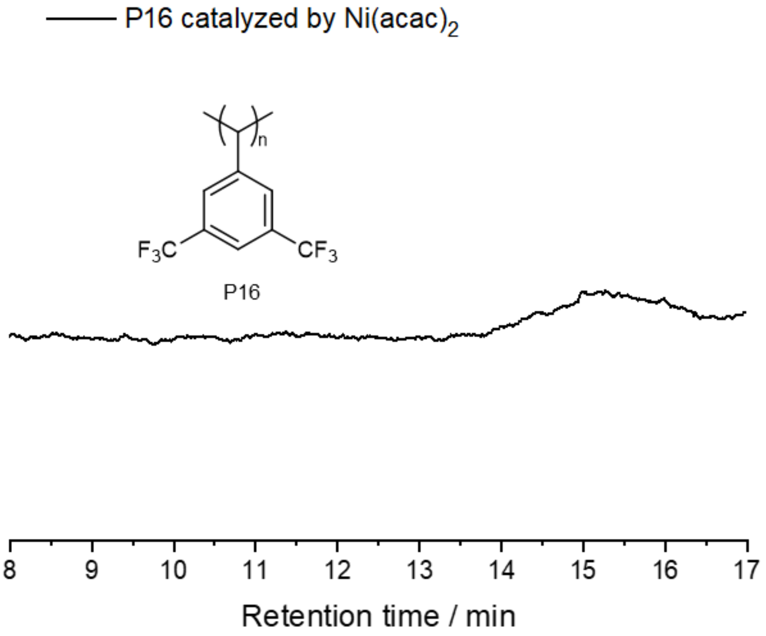

Fig. S280. SEC trace of polymer **P16** synthesized using Ni(acac)$_2$ as a catalyst (The polymer exhibits poor solubility in THF) (Entry 16, Table S5).

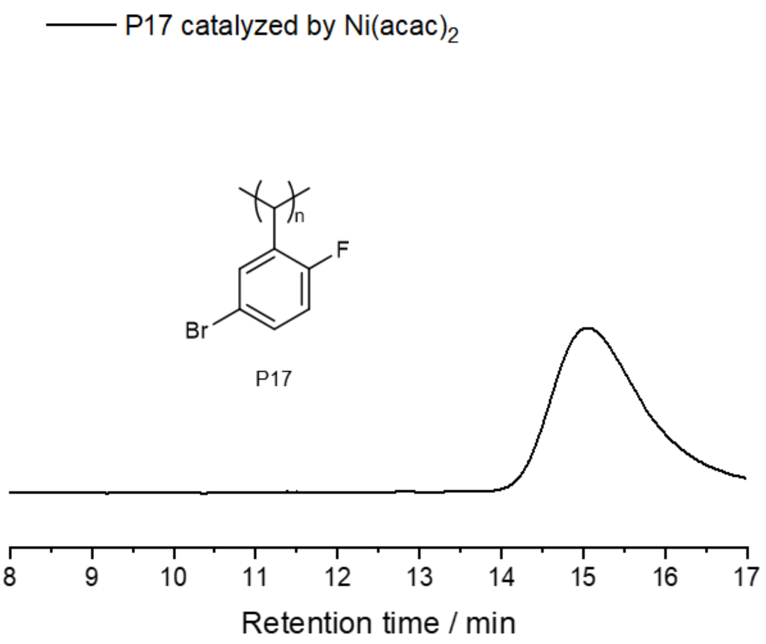

Fig. S281. SEC trace of polymer **P17** synthesized using Ni(acac)$_2$ as a catalyst (Entry 17, Table S5).

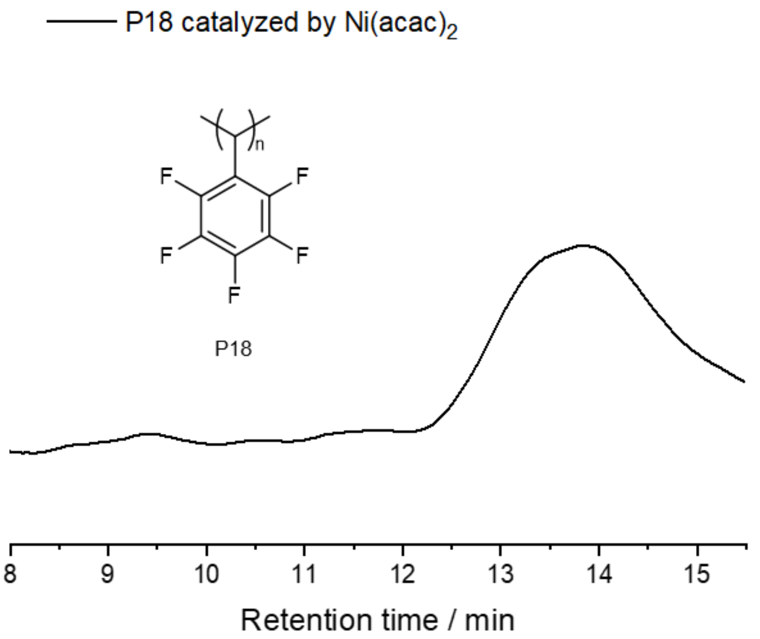

Fig. S282. SEC trace of polymer **P18** synthesized using Ni(acac)$_2$ as a catalyst (The polymer exhibits poor solubility in THF) (Entry 18, Table S5).

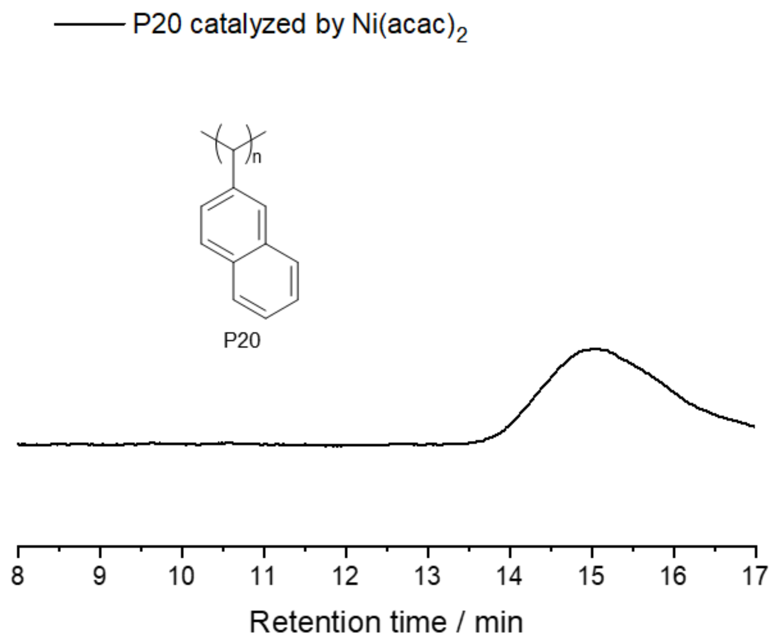

Fig. S283. SEC trace of polymer **P20** synthesized using $Ni(acac)_2$ as a catalyst (The polymer exhibits poor solubility in THF) (Entry 20, Table S5).

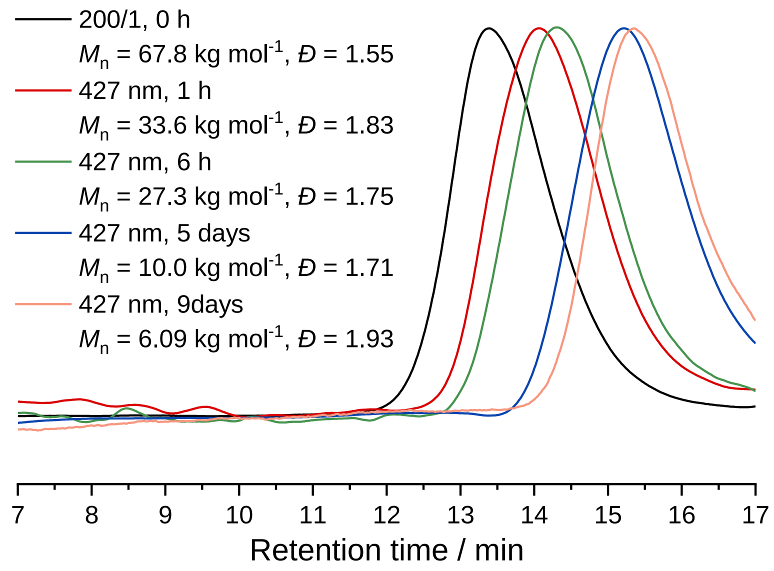

Fig. S284. SEC traces of polymer **P1** synthesized using $C(Ph)_3^+BF_4^-$ as a catalyst ($[1]_0/[Cat.]_0$ = 200/1, before and after UV light (427 nm) irradiation for different durations.

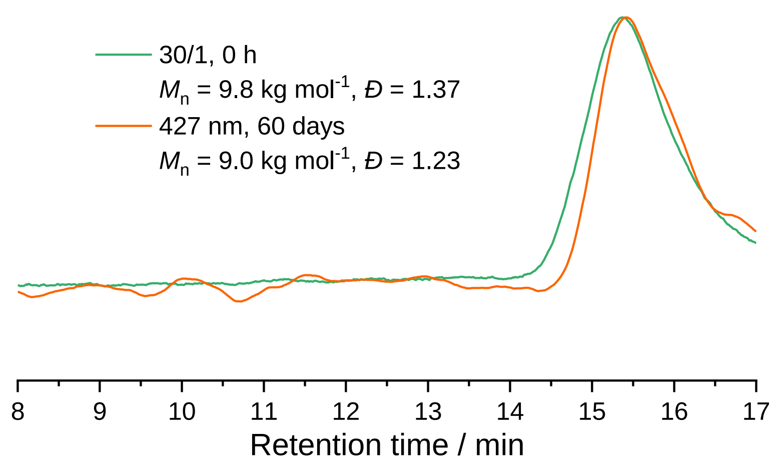

Fig. S285. SEC traces of polymer **P1** synthesized using $Ni(acac)_2$ as a catalyst ($[1]_0/[Cat.]_0$ = 30/1, before and after UV light (427 nm) irradiation for different durations (The polymer exhibits poor solubility in THF).

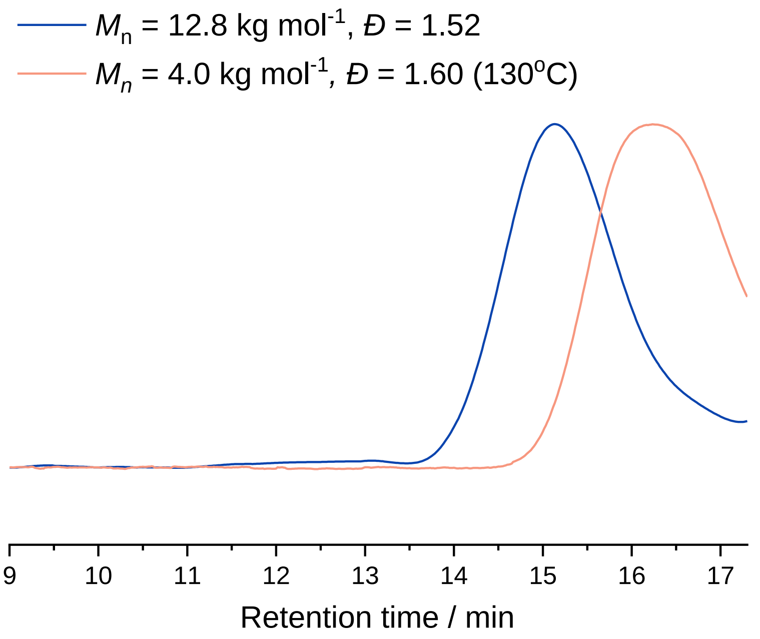

Fig. S286. SEC traces of polymer **P1** synthesized using $C(Ph)_3^+BF_4^-$ as a catalyst ($[1]_0/[Cat.]_0$ = 30/1 (blue) and after heating at 130 ^{o}C (orange).

Photoluminescent (PL) spectra

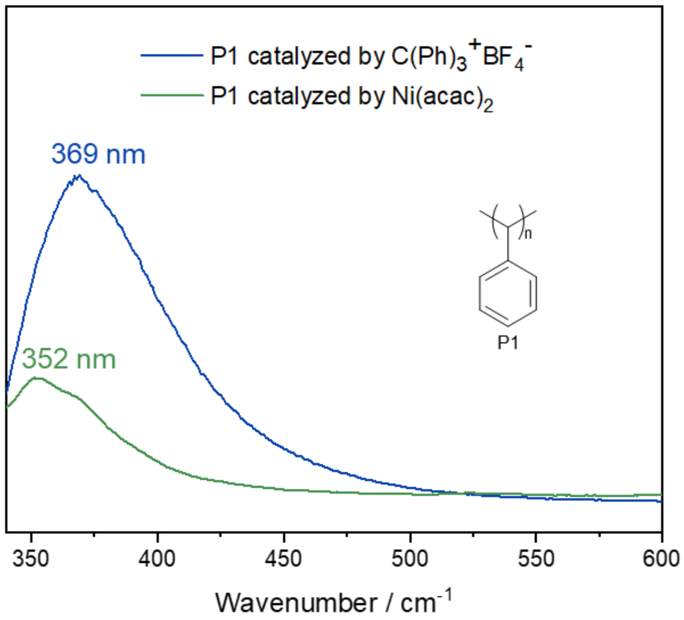

Fig. S287. Photoluminescent (PL) spectra of polymer **P1** synthesized using $C(Ph)_3^+BF_4^-$ (blue) and $Ni(acac)_2$ (green) as catalysts ([**P1**] = 0.5 mg ml^{-1}) (λ_{ex} = 320 nm).

Circular dichroism (CD) spectrum

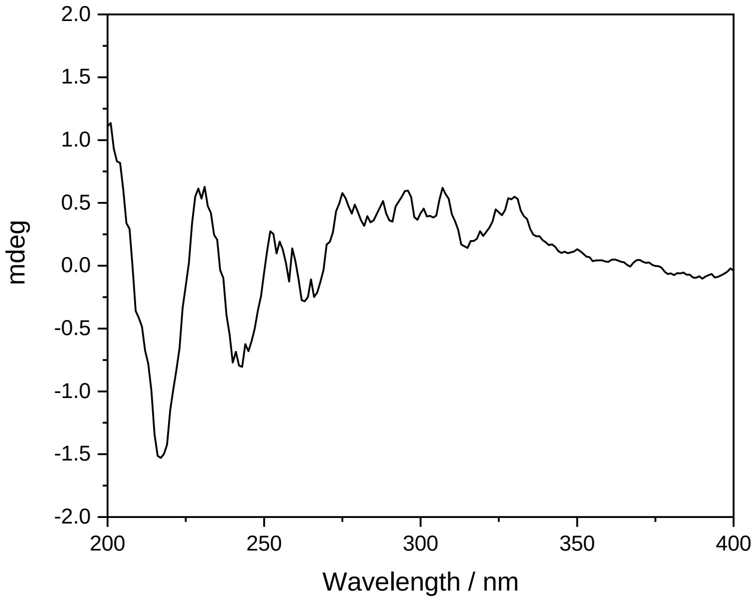

Fig. S288. CD spectrum of polymer **P1** synthesized using $C(Ph)_3^+BF_4^-$ ([**P1**] = 0.5 mg mL^{-1} in THF at room temperature).

Proposed Mechanism

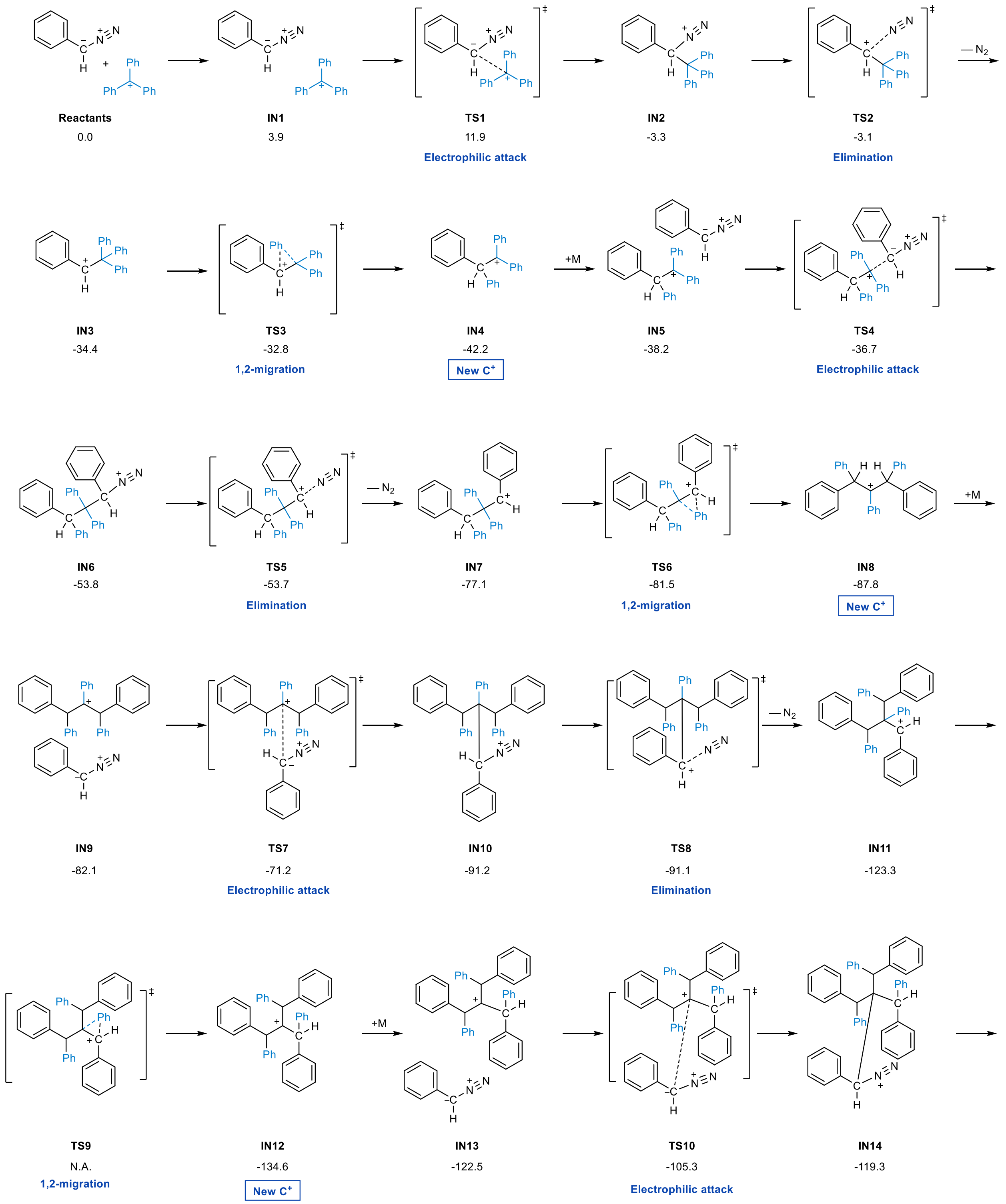

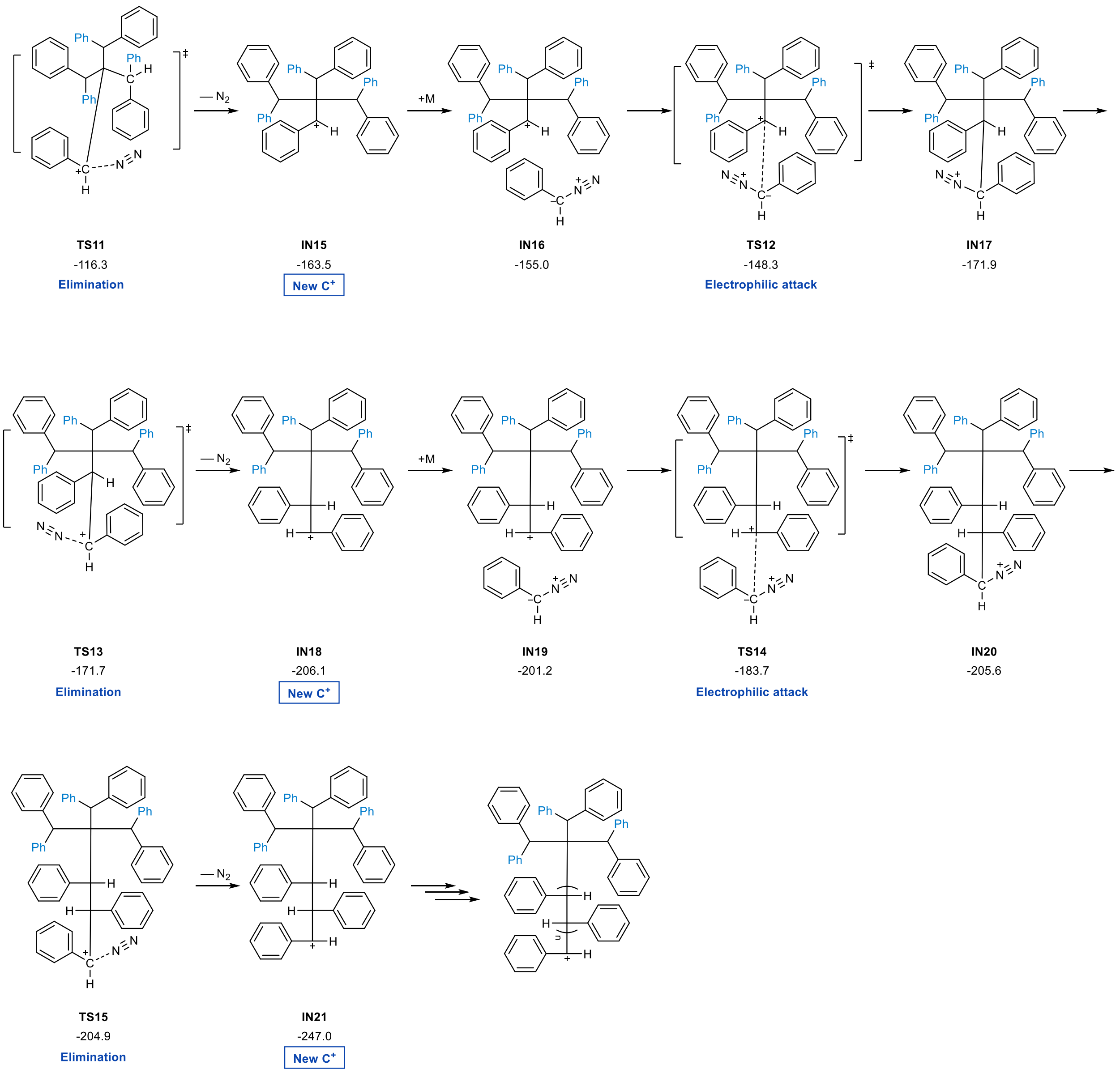

Fig. S289. Proposed mechanism and DFT-calculated results for the C1 polymerization of monomer **1** catalyzed by $C(Ph)_3{}^+BF_4{}^-$. Relative Gibbs free energies (in kcal mol^{-1}) were computed at 298 K using the SMD(toluene)/M06-2X/6-311++G(d,p)//M06-2X/6-31++G(d,p) level of theory. The $BF_4{}^-$ counterion was omitted in the calculations for computational efficiency.

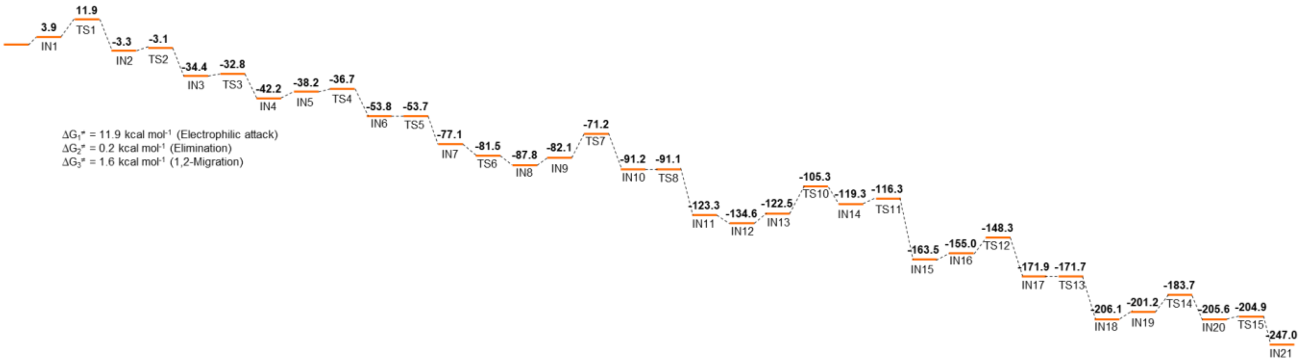

Fig. S290. DFT-calculated free-energy profiles for the isotactic sequence propagation in C1 polymerization catalyzed by $C(Ph)_3{}^+BF_4{}^-$.

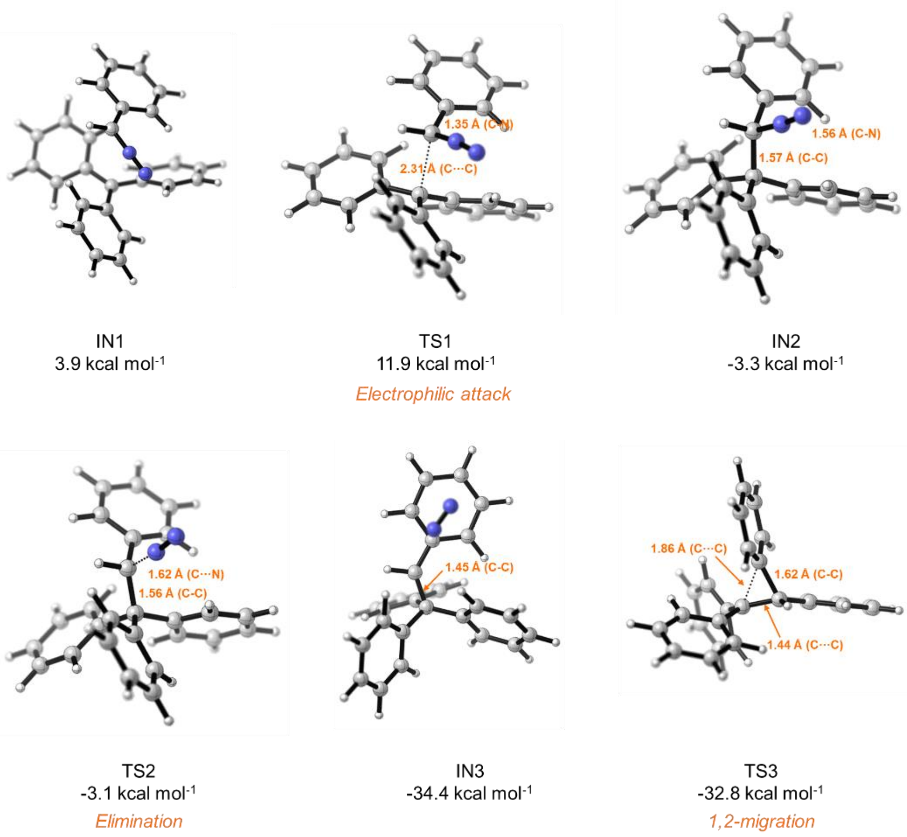

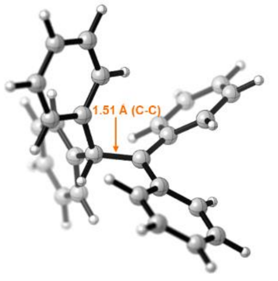

IN4
-42.2 kcal mol^{-1}

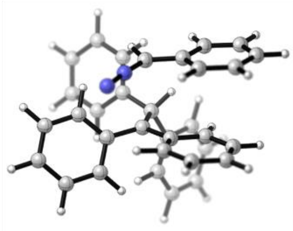

IN5
-38.2 kcal mol^{-1}

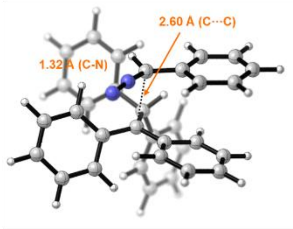

TS4
-36.7 kcal mol^{-1}
Electrophilic attack

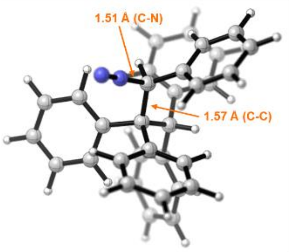

IN6
-53.8 kcal mol^{-1}

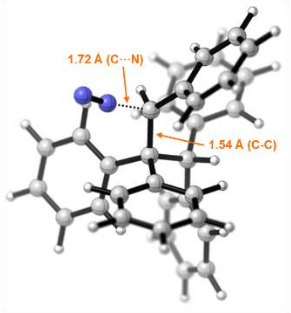

TS5
-53.7 kcal mol^{-1}
Elimination

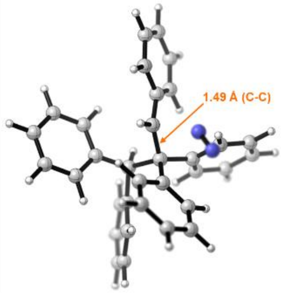

IN7
-77.1 kcal mol^{-1}

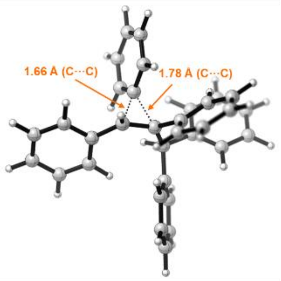

TS6
-81.5 kcal mol^{-1}
1,2-migration

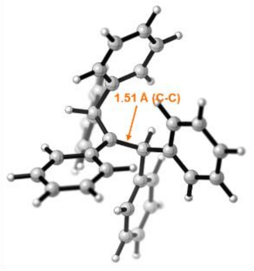

IN8
-87.8 kcal mol^{-1}

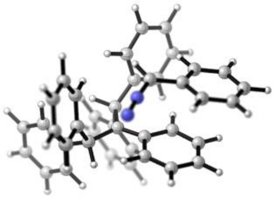

IN9
-82.1 kcal mol^{-1}

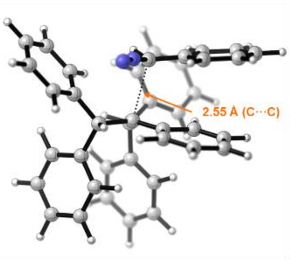

TS7
-71.2 kcal mol^{-1}
Electrophilic attack

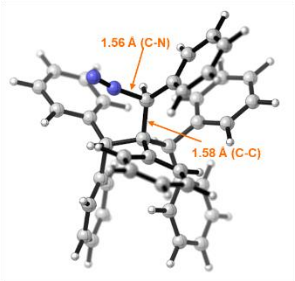

IN10
-91.2 kcal mol^{-1}

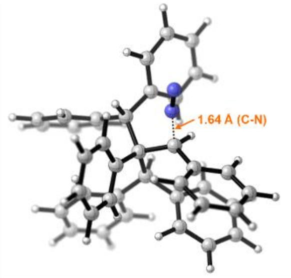

TS8
-91.1 kcal mol^{-1}
Elimination

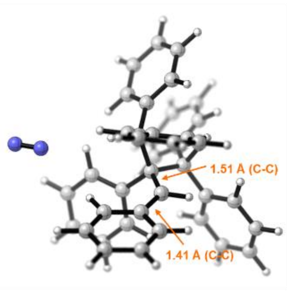

IN11
-123.3 kcal mol^{-1}

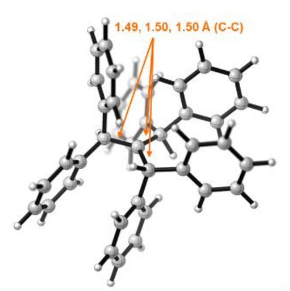

IN12
-134.6 kcal mol^{-1}

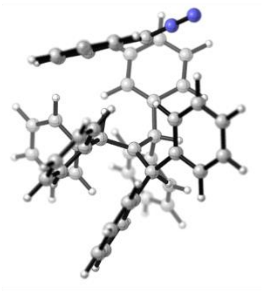

IN13
-122.5 kcal mol^{-1}

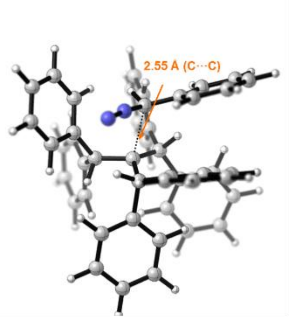

TS10
-105.3 kcal mol^{-1}
Electrophilic attack

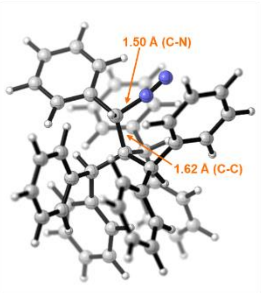

IN14
-119.3 kcal mol^{-1}

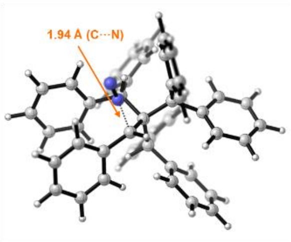

TS11
-116.3 kcal mol^{-1}
Elimination

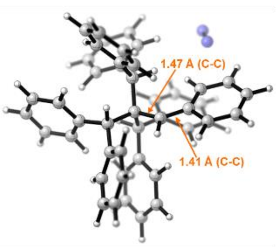

IN15
-163.5 kcal mol^{-1}

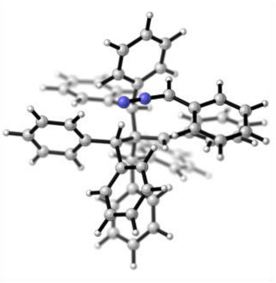

IN16
-155.0 kcal mol^{-1}

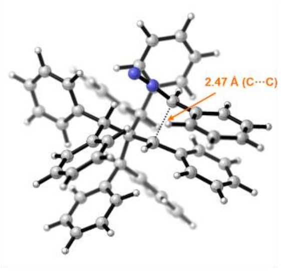

TS2
-148.3 kcal mol^{-1}
Electrophilic attack

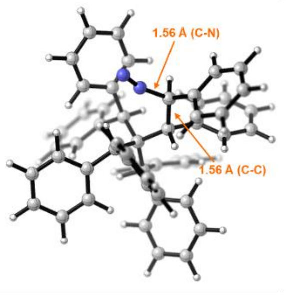

IN17
-171.9 kcal mol^{-1}

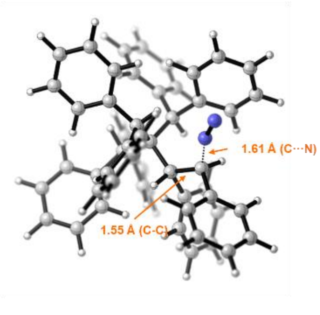

TS13
-171.7 kcal mol^{-1}
Elimination

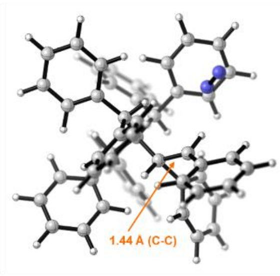

IN18
-206.1 kcal mol^{-1}

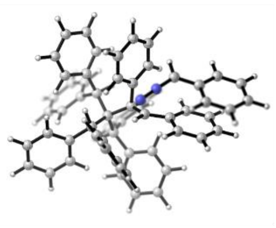

IN19
-201.2 kcal mol^{-1}

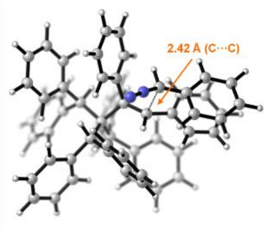

TS14
-183.7 kcal mol^{-1}
Electrophilic attack

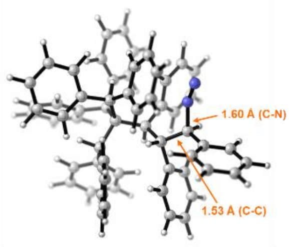

IN20
-205.6 kcal mol^{-1}

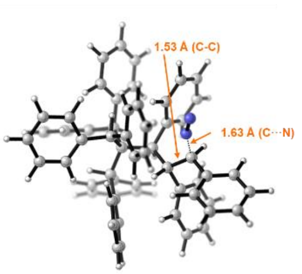

TS15
-204.9 kcal mol^{-1}
Elimination

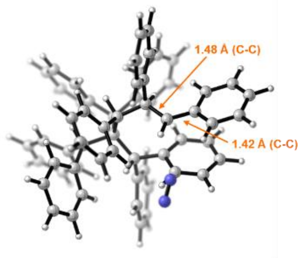

IN21
-247.0 kcal mol^{-1}

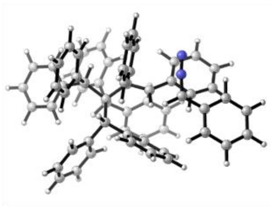

IN19-2
-200.9 kcal mol^{-1}

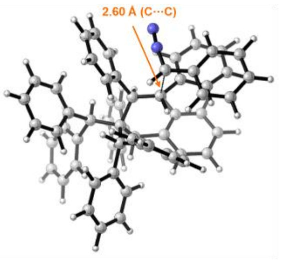

TS14-2
-186.7 kcal mol^{-1}
Electrophilic attack

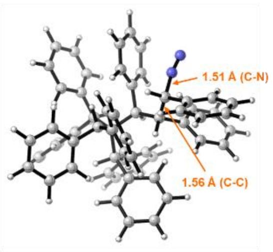

IN20-2
-212.1 kcal mol^{-1}

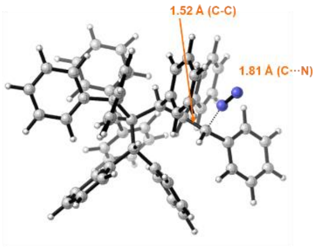

TS15-2
-211.0 kcal mol^{-1}
Elimination

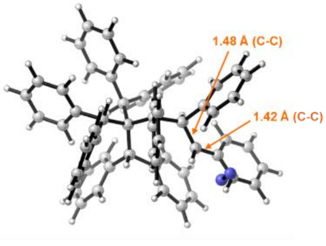

IN21-2
-245.9 kcal mol^{-1}

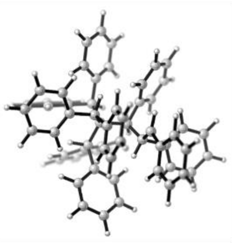

IN21$_{anti}$

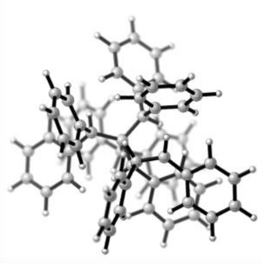

IN21$_{syn}$
6.6 kcal mol^{-1}
higher than IN21$_{anti}$

Fig. S291. Optimized intermediates and transition states in the C1 polymerization mediated by $C(Ph)_3^+BF_4^-$.

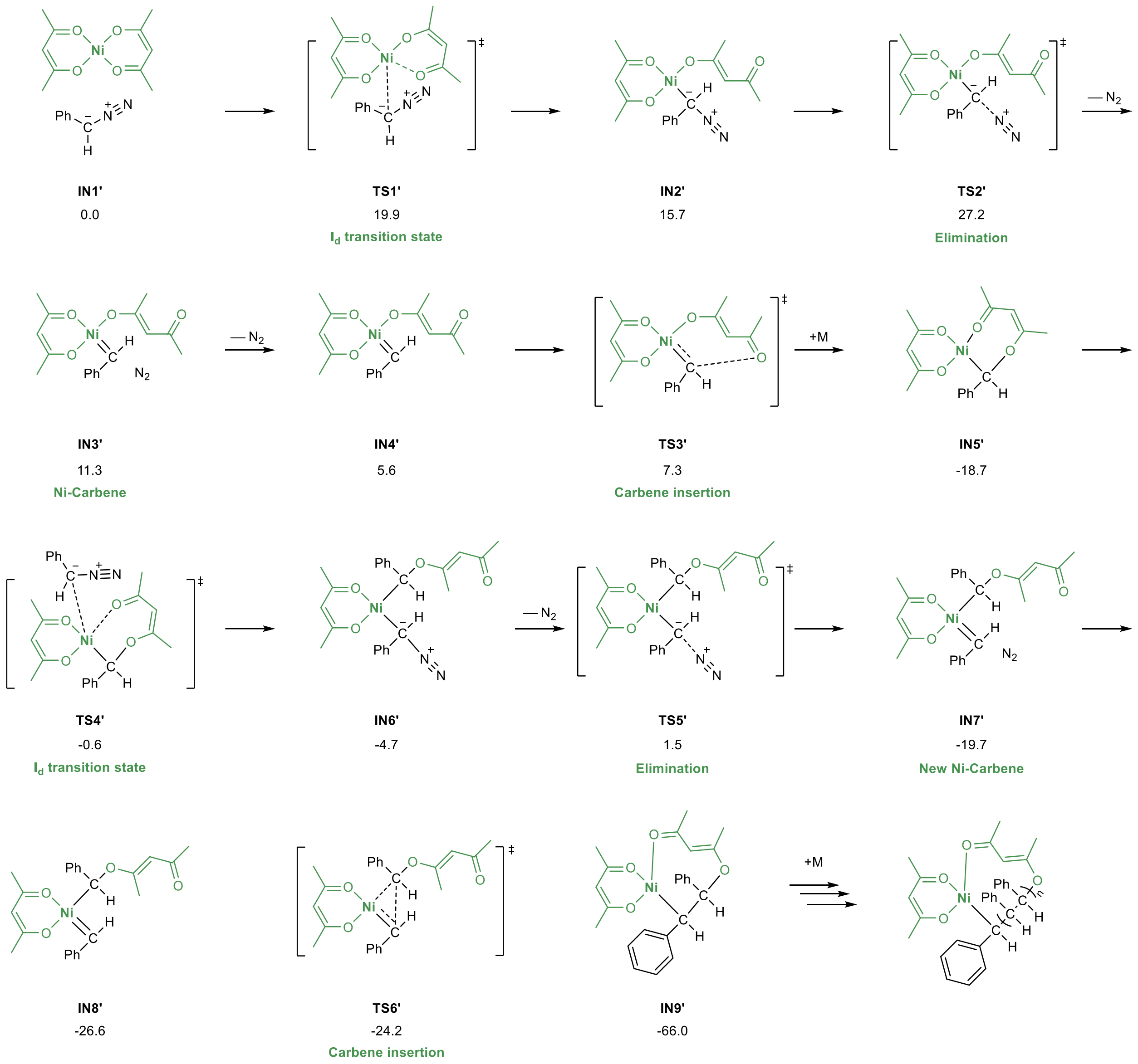

Fig. S292. Proposed mechanism and DFT-calculated results for the C1 polymerization of monomer **1** catalyzed by $Ni(acac)_2$. Relative Gibbs free energies (in kcal mol^{-1}) were computed at 298 K using the SMD(toluene)/M06/def2TZVP//M06/6-311++G(d,p) level of theory.

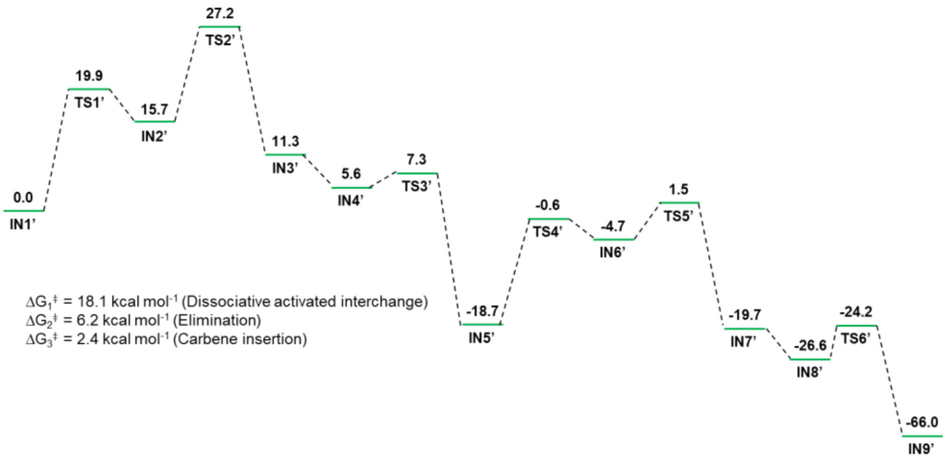

Fig. S293. DFT-calculated free-energy profiles for the syndiotactic sequence propagation in C1 polymerization catalyzed by Ni(acac)$_2$.

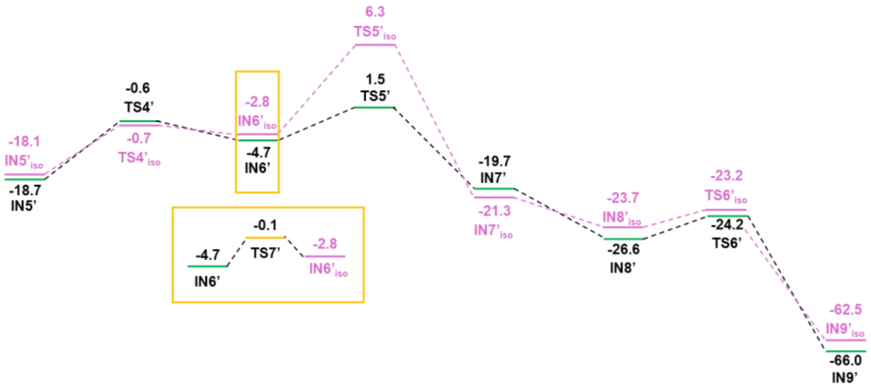

Fig. S294. DFT-calculated free-energy profiles for the syndiotactic (green) and isotactic (pink) sequence propagation in C1 polymerization catalyzed by Ni(acac)$_2$.

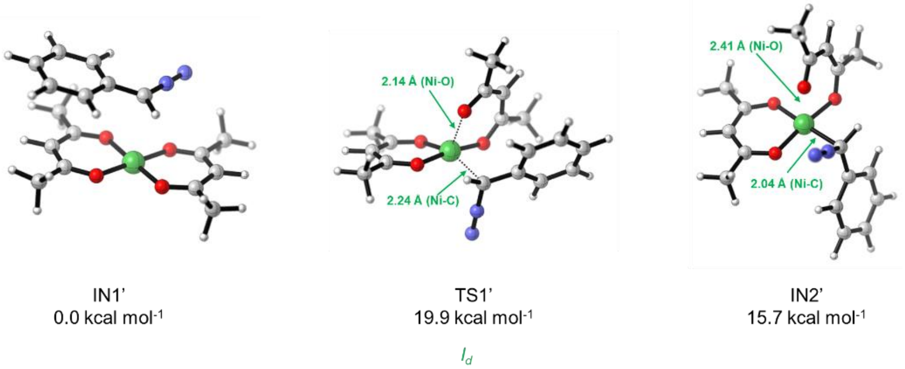

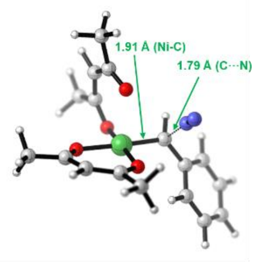

TS2'
27.2 kcal mol^{-1}
Elimination

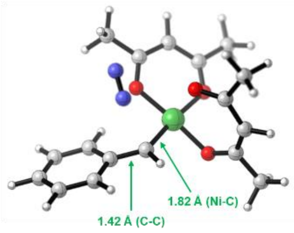

IN3'
11.3 kcal mol^{-1}

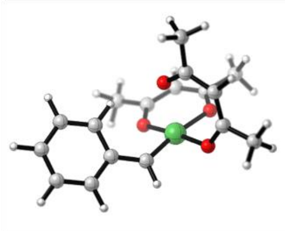

IN4'
5.6 kcal mol^{-1}

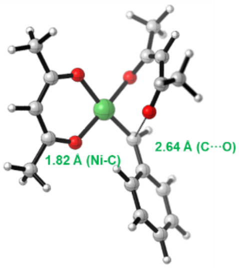

TS3'
7.3 kcal mol^{-1}
Carbene insertion

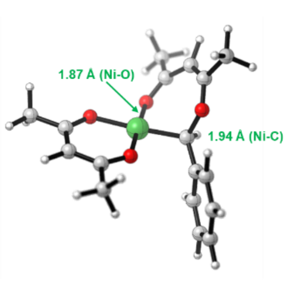

IN5'
-18.7 kcal mol^{-1}

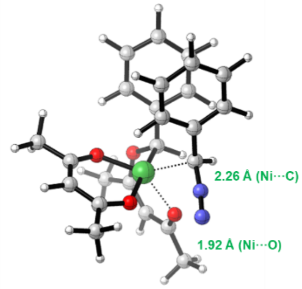

TS4'
-0.6 kcal mol^{-1}
I_d

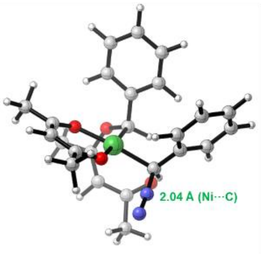

IN6'
-4.7 kcal mol^{-1}

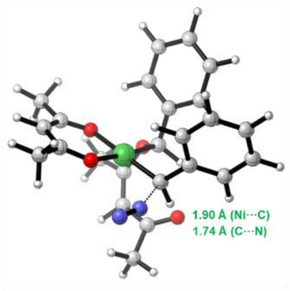

TS5'
1.5 kcal mol^{-1}
Elimination

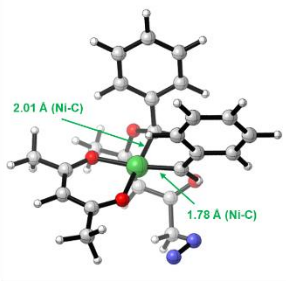

IN7'
-19.7 kcal mol^{-1}

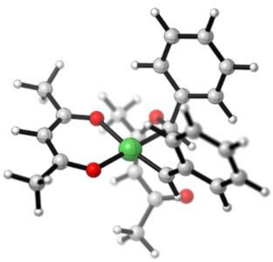

IN8'
-26.6 kcal mol^{-1}

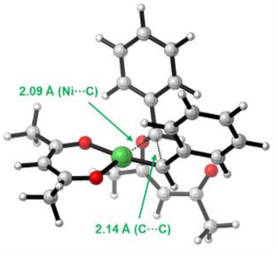

TS6'
-24.2 kcal mol^{-1}
Carbene insertion

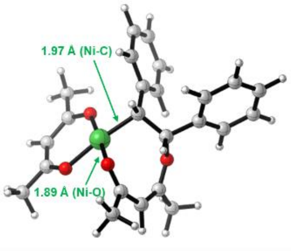

IN9'
-66.0 kcal mol^{-1}

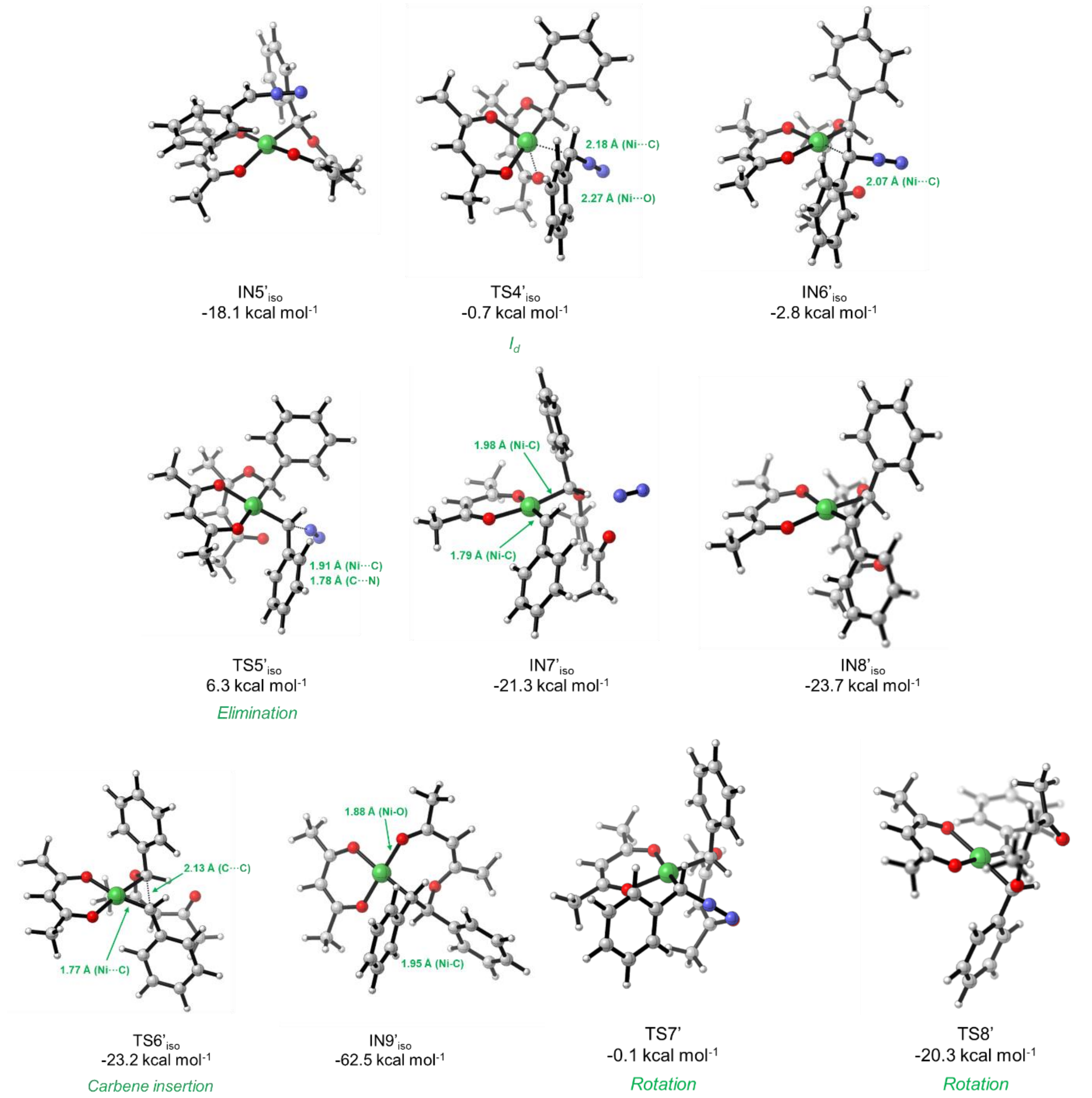

Fig. S295. Optimized intermediates and transition states in the C1 polymerization catalyzed by Ni(acac)$_2$.

Computational methods and results

All geometry optimizations were performed using M06-2X/6-31++G(d,p) (for $C(Ph)_3^+BF_4^-$ catalysts) or M06/6-311++G(d,p) (for Ni(acac)$_2$ catalysts) level of theory by the Gaussian 16 C.02 program[3]. The harmonic vibrational frequency analyses were performed on all structures, including intermediates and transition states at the same level. Single-point energies were further refined using M06-2X/6-31++G(d,p) (for $C(Ph)_3^+BF_4^-$ catalysts) or M06/def2TZVP (for Ni(acac)$_2$ catalysts) level of theory. The reported Gibbs energies were gained by adding the single-point energies and the Gibbs free energy correction at 298.15 K. The three-dimensional structures of the molecules were drawn with CYL view[4].

Cartesian coordinates for the optimized structures

M06-2X/6-31++G(d,p)

$PhCHN_2$

C	-1.47990000	1.42534200	-0.00000300
C	-0.16047900	0.98467800	0.00000000
C	0.12873500	-0.38866900	0.00000000
C	-0.93659200	-1.30225400	-0.00000400
C	-2.25247500	-0.85367300	-0.00000800
C	-2.53385400	0.51241100	-0.00000800
H	-1.68449000	2.49148600	-0.00000300
H	0.64726100	1.71172500	0.00000300
H	-0.72574800	-2.36807100	-0.00000500
H	-3.06281400	-1.57612400	-0.00001100
H	-3.56112600	0.86093400	-0.00001000
C	1.49334800	-0.90199100	0.00000400
H	1.73511200	-1.95515800	0.00000300
N	2.50566900	-0.08752800	0.00000800
N	3.36563200	0.65612000	0.00001200

$C(Ph)_3^+$

C	1.08878600	-2.12730300	-0.67269800
C	0.04751600	-1.44427900	-0.00031700
C	-0.94631300	-2.19466000	0.67205600
C	-0.88609900	-3.57917200	0.68338000
C	0.13983400	-4.23758000	-0.00053700
C	1.12010300	-3.51272300	-0.68433600
H	1.83119000	-1.56030000	-1.22505400
H	-1.72435000	-1.67797100	1.22470600
H	-1.63195800	-4.14916500	1.22619900
H	0.17570700	-5.32239500	-0.00061500

H	1.90199000	-4.03218700	-1.22713400
C	-0.00013800	0.00051200	-0.00045700
C	-1.27515600	0.68147700	-0.00047400
C	-2.38809100	0.11980300	-0.67048500
C	-1.42756700	1.91860300	0.66982600
C	-3.60377700	0.78493800	-0.68150300
H	-2.26878900	-0.80742900	-1.22160100
C	-2.65678200	2.55840700	0.68159000
H	-0.59054400	2.33531600	1.22068900
C	-3.74077800	1.99748300	0.00028300
H	-4.44522400	0.36629100	-1.22235200
H	-2.77702200	3.49030600	1.22281600
H	-4.69837500	2.50847500	0.00066000
C	1.22740400	0.76379100	-0.00027300
C	1.29905900	2.00773500	-0.67136100
C	2.37389000	0.27684700	0.67155600
C	2.48363100	2.72692800	-0.68193300
H	0.43721900	2.36818900	-1.22366100
C	3.54323400	1.02050900	0.68387600
H	2.31497800	-0.65594600	1.22304400
C	3.60116000	2.23902300	0.00142700
H	2.54310400	3.66436200	-1.22370100
H	4.40957200	0.65838800	1.22622800
H	4.52300500	2.81201300	0.00205900

IN1

C	3.10030900	-1.42855900	0.89382900
C	1.87788200	-1.18873200	1.51454700
C	1.60110900	0.06646800	2.07953400
C	2.59299800	1.05856400	2.04293000
C	3.81814200	0.80742900	1.43328800
C	4.07503100	-0.43017700	0.84114100

H	3.29736900	-2.40616400	0.46423000
H	1.14288500	-1.98675800	1.57174900
H	2.39774100	2.02897200	2.49079800
H	4.57838400	1.58251800	1.42364700
H	5.03177500	-0.62325700	0.36797800
C	0.31116100	0.38246400	2.68415100
H	0.11083100	1.29979700	3.22105600
N	-0.65431900	-0.48815600	2.66889000
N	-1.48525700	-1.26270800	2.61274000
C	-0.73946700	2.53054500	-1.49614000
C	-0.06890000	1.33779900	-1.12728100
C	1.34070500	1.35171300	-1.00316400
C	2.04653500	2.52442600	-1.22897200
C	1.37176100	3.68544100	-1.61019200
C	-0.02042000	3.68601300	-1.75062700
H	-1.81576300	2.51819400	-1.63403000
H	1.86530300	0.45915300	-0.67397100
H	3.12356300	2.53259100	-1.09980000
H	1.93101200	4.59616900	-1.80034100
H	-0.53593900	4.58664200	-2.06540400
C	-0.82156300	0.12514600	-0.89465300
C	-0.25129500	-1.15915500	-1.23492800
C	0.69097200	-1.27365500	-2.28526700
C	-0.61716400	-2.32377500	-0.51711100
C	1.25129500	-2.50260300	-2.59400300
H	0.93594100	-0.40167900	-2.88213200
C	-0.03627800	-3.54539100	-0.81988600
H	-1.31556800	-2.24652300	0.31003800
C	0.89687100	-3.63582200	-1.85621500
H	1.95500300	-2.58537500	-3.41492600
H	-0.30349400	-4.42746200	-0.24802600
H	1.34334500	-4.59579000	-2.09637300

C	-2.15230800	0.20381200	-0.32908400
C	-3.15393700	-0.72251900	-0.70028900
C	-2.46715500	1.21163200	0.61271300
C	-4.42342700	-0.63963100	-0.14907800
H	-2.93956800	-1.46676800	-1.46038900
C	-3.72915000	1.26733100	1.18186900
H	-1.69209300	1.90339100	0.92807100
C	-4.70800700	0.34534000	0.79926100
H	-5.19396800	-1.33724600	-0.45793100
H	-3.95562700	2.02342400	1.92573700
H	-5.69830700	0.39760200	1.24038100

TS1

C	-3.84108700	-1.36711100	-0.98677300
C	-2.45757100	-1.32525200	-1.13020200
C	-1.82785900	-0.12694200	-1.48979100
C	-2.60083700	1.01924000	-1.72578300
C	-3.98213600	0.97106100	-1.57498300
C	-4.60472500	-0.22028700	-1.20079000
H	-4.32294000	-2.29918500	-0.71153700
H	-1.87570200	-2.22723000	-0.96688000
H	-2.11652500	1.94501800	-2.02483400
H	-4.57437800	1.86045000	-1.76246900
H	-5.68313700	-0.25775900	-1.09003900
C	-0.36273500	-0.01335700	-1.60789100
H	0.05271600	0.90711000	-2.00782900
N	0.28034800	-1.08278200	-2.12299300
N	0.88100900	-1.97974000	-2.41437400
C	1.59913900	2.51519100	0.69925100
C	0.52572500	1.61163200	0.58379400
C	-0.78615400	2.11306300	0.70342300
C	-1.01482800	3.46963400	0.88855100

C	0.06091800	4.35435400	0.98848600
C	1.36513400	3.87093300	0.90701700
H	2.62042700	2.15356000	0.66771000
H	-1.62893700	1.43363300	0.64490000
H	-2.03320200	3.83647900	0.96492800
H	-0.11830400	5.41325600	1.14354700
H	2.20565900	4.54829200	1.01287200
C	0.76984500	0.16116700	0.40093300
C	-0.00164000	-0.79363500	1.22564200
C	-0.68850300	-0.37432100	2.38036000
C	-0.01392300	-2.16956000	0.91396100
C	-1.37593500	-1.28912300	3.17248400
H	-0.65007500	0.66338700	2.68876200
C	-0.70893800	-3.07810700	1.69804900
H	0.53448600	-2.53525600	0.05190400
C	-1.39872400	-2.63865200	2.83032100
H	-1.88432300	-0.94452800	4.06647400
H	-0.70698300	-4.13041600	1.43353500
H	-1.93533600	-3.34937200	3.45027800
C	2.11140500	-0.25158200	-0.07657500
C	2.83238900	-1.27027600	0.57024500
C	2.72353400	0.40721900	-1.16430900
C	4.10969100	-1.62345500	0.13785800
H	2.41500400	-1.75813200	1.44363100
C	3.98560600	0.04125000	-1.60482600
H	2.20071000	1.21519200	-1.66984500
C	4.68418200	-0.98055600	-0.95345600
H	4.65452900	-2.40074800	0.66264900
H	4.43054300	0.55261400	-2.45175300
H	5.67444000	-1.26387400	-1.29439600

IN2

C	-3.97840700	-1.00821300	-0.50561000
C	-2.58755700	-1.04189700	-0.53865200
C	-1.89156200	-0.00801000	-1.17810600
C	-2.59096300	1.03392700	-1.79787900
C	-3.98075500	1.06626900	-1.74809200
C	-4.67401400	0.04525600	-1.09989100
H	-4.51936800	-1.80784300	-0.01144300
H	-2.06064200	-1.86557800	-0.06829500
H	-2.04922400	1.83642400	-2.29190000
H	-4.51838300	1.88363500	-2.21592700
H	-5.75820900	0.06545200	-1.06393100
C	-0.38835100	0.06257100	-1.23406200
H	-0.08583100	0.87383700	-1.90068300
N	0.01423600	-1.15754400	-2.12522500
N	0.17165500	-2.02017700	-2.78597200
C	1.40869600	2.38755600	0.72768600
C	0.33102000	1.50311800	0.59872600
C	-0.94368000	1.95467100	0.98152600
C	-1.13035400	3.24562400	1.46732300
C	-0.04849400	4.11563000	1.58743500
C	1.21982100	3.67835700	1.22153300
H	2.41075700	2.07962100	0.45420200
H	-1.80329100	1.29594800	0.92820000
H	-2.12514600	3.56535500	1.75992600
H	-0.19369300	5.12069500	1.96919700
H	2.07482700	4.33913400	1.31810000
C	0.54195800	0.07433300	0.03172400
C	0.13741200	-1.05630700	0.99662700
C	-0.48686800	-0.82539700	2.22265600
C	0.39460000	-2.38741000	0.62765600
C	-0.90003800	-1.89213200	3.02572600
H	-0.64916200	0.18452500	2.57658500

C	-0.02395000	-3.45079800	1.41986400
H	0.97677000	-2.60868700	-0.26236200
C	-0.69161900	-3.20545600	2.62092200
H	-1.38256800	-1.68451600	3.97511000
H	0.18935700	-4.46864600	1.11004800
H	-1.01776000	-4.03115700	3.24433900
C	2.00632100	-0.10411400	-0.41945600
C	2.93476400	-0.64174500	0.48024700
C	2.47767800	0.35347000	-1.65695600
C	4.28218800	-0.74158100	0.14459800
H	2.60531600	-0.97763400	1.45794500
C	3.82530300	0.24234000	-2.00045300
H	1.82520600	0.84753000	-2.37230300
C	4.73274200	-0.30946800	-1.10165400
H	4.98064100	-1.15883800	0.86234400
H	4.16211400	0.60378700	-2.96639700
H	5.78169900	-0.39257900	-1.36500500

TS2

C	-3.98130000	-0.99906600	-0.47435600
C	-2.59068600	-1.02683800	-0.49737100
C	-1.89446000	-0.00895600	-1.16448200
C	-2.59716300	1.01002000	-1.82013200
C	-3.98707100	1.03801900	-1.77854200
C	-4.67862500	0.03315900	-1.10385300
H	-4.52178500	-1.78644100	0.03961200
H	-2.06331900	-1.83438500	-0.00114100
H	-2.05655200	1.79943900	-2.33601700
H	-4.52609600	1.83842500	-2.27325200
H	-5.76314000	0.04885700	-1.07444000
C	-0.39901600	0.07587400	-1.21399800
H	-0.09345700	0.87202100	-1.89600200

N	0.01797500	-1.18214900	-2.14134600
N	0.14193300	-2.03502000	-2.82145900
C	1.40639400	2.39431300	0.71355800
C	0.32947700	1.50699700	0.59863900
C	-0.94273300	1.95620600	0.99214700
C	-1.12770900	3.24773700	1.47700300
C	-0.04673500	4.12051300	1.58413100
C	1.21903200	3.68570100	1.20622200
H	2.40633200	2.08761500	0.43110300
H	-1.80125300	1.29519800	0.94764500
H	-2.12031100	3.56596500	1.77846200
H	-0.19052300	5.12612500	1.96498600
H	2.07320300	4.34895900	1.29264700
C	0.53823800	0.07490000	0.03290900
C	0.14433000	-1.05231000	1.00605100
C	-0.45650900	-0.81702800	2.24276400
C	0.39065500	-2.38419700	0.63392700
C	-0.85846500	-1.88134000	3.05479400
H	-0.60964100	0.19419000	2.59736600
C	-0.01679600	-3.44511000	1.43514500
H	0.95386200	-2.60784200	-0.26757900
C	-0.66146900	-3.19585500	2.64798300
H	-1.32316300	-1.67104200	4.01245400
H	0.18676000	-4.46413100	1.12267500
H	-0.97896600	-4.01966900	3.27831900
C	2.00013300	-0.10255200	-0.42684400
C	2.93407300	-0.63387000	0.47097300
C	2.46391900	0.34757800	-1.66982500
C	4.27957700	-0.73489300	0.12838800
H	2.61032600	-0.96454000	1.45234300
C	3.80979000	0.23540300	-2.02003600
H	1.80713000	0.83539700	-2.38531600

C	4.72276200	-0.30996300	-1.12300700
H	4.98230900	-1.14753200	0.84462200
H	4.14077200	0.59081500	-2.99022100
H	5.77028500	-0.39387800	-1.39177200

IN3

C	-3.89527700	0.07734900	0.59467900
C	-2.50815700	0.08030800	0.55464200
C	-1.84153700	0.49997800	-0.61317700
C	-2.60179600	0.92964500	-1.71647000
C	-3.99013900	0.92192300	-1.67137300
C	-4.63830200	0.49180900	-0.51488900
H	-4.40347500	-0.25792300	1.49233900
H	-1.95351700	-0.26164300	1.42018900
H	-2.09471400	1.25799600	-2.61996200
H	-4.56288700	1.24599100	-2.53326500
H	-5.72260100	0.47738700	-0.47453900
C	-0.39543100	0.46686800	-0.82239700
H	-0.10987500	0.68876300	-1.84884400
N	-2.29315800	-2.39107400	-1.90393400
N	-3.35569200	-2.58002500	-1.69658400
C	1.50301200	2.37293600	-0.70274000
C	0.67004300	1.58966500	0.13306200
C	-0.02187600	2.21600800	1.19691400
C	0.15353400	3.56527300	1.44183100
C	0.99211200	4.32099800	0.61375800
C	1.65906100	3.72732400	-0.46141400
H	2.03514500	1.89800200	-1.52179800
H	-0.70073100	1.63811900	1.81461300
H	-0.37023100	4.03800100	2.26498200
H	1.11790000	5.38238100	0.80382700
H	2.30077700	4.32117700	-1.10241300

C	0.70404400	-0.00447000	-0.00401400
C	0.47416700	-0.80798000	1.25805000
C	0.94281100	-0.40967600	2.50931200
C	-0.11754000	-2.06727500	1.11078100
C	0.78505600	-1.24728000	3.61403100
H	1.43452500	0.55176500	2.63163400
C	-0.27550900	-2.90042100	2.21315400
H	-0.45521000	-2.39121400	0.12908900
C	0.17076900	-2.48868200	3.46979300
H	1.14904500	-0.92847500	4.58507500
H	-0.74204400	-3.87228800	2.09105500
H	0.04810800	-3.13800000	4.33009000
C	1.91215800	-0.51746000	-0.76303900
C	3.19221700	-0.35077200	-0.22589800
C	1.74774200	-1.22573500	-1.95338000
C	4.29880200	-0.86619200	-0.88971800
H	3.32028300	0.18671200	0.71074000
C	2.85997300	-1.74906100	-2.61566600
H	0.75268000	-1.39586800	-2.35872100
C	4.13413400	-1.56526900	-2.08858400
H	5.29062900	-0.72845200	-0.47226600
H	2.72484200	-2.30316400	-3.53851000
H	4.99888600	-1.97044900	-2.60317700

TS3

C	4.08579500	0.58739000	-0.57359300
C	2.72141100	0.47260100	-0.31879100
C	1.97443400	-0.53943700	-0.93767500
C	2.62594000	-1.42562700	-1.80539800
C	3.98879600	-1.30676100	-2.05931800
C	4.72312100	-0.29740600	-1.44184100
H	4.65130400	1.37748300	-0.09090500

H	2.25755200	1.18338700	0.35494500
H	2.06075300	-2.21793300	-2.28964900
H	4.47290800	-2.00136400	-2.73734000
H	5.78596200	-0.19914600	-1.63569000
C	0.50543500	-0.78049600	-0.75805600
H	0.14243700	-1.48939500	-1.49825100
C	-0.99894200	-2.35178300	0.68708900
C	0.00490600	-1.35905000	0.66520200
C	0.81344300	-1.15993300	1.80298900
C	0.62325400	-1.94456100	2.92995100
C	-0.36936700	-2.92795300	2.93643300
C	-1.17692400	-3.13585600	1.81300900
H	-1.64094300	-2.48711700	-0.17897100
H	1.60658100	-0.42108400	1.78513800
H	1.25455900	-1.80007600	3.79959800
H	-0.51422900	-3.53875300	3.82201700
H	-1.94302400	-3.90282800	1.82399900
C	-0.51343100	0.13610300	-0.30602600
C	-0.21008600	1.43259100	0.37978400
C	-0.35258300	1.66826600	1.74728800
C	0.13833500	2.48551600	-0.47677200
C	-0.10693700	2.94105100	2.26242700
H	-0.65512200	0.86392100	2.41127700
C	0.37951600	3.75304100	0.04309000
H	0.23434300	2.30662100	-1.54459500
C	0.26388800	3.98161400	1.41443500
H	-0.21196300	3.11614300	3.32791500
H	0.66076100	4.56053500	-0.62442900
H	0.45514200	4.96996700	1.81850500
C	-1.89279500	0.02268900	-0.84402000
C	-2.97854800	0.47697100	-0.07882900
C	-2.12236400	-0.47825200	-2.13422500

C	-4.27046100	0.39132800	-0.57890600
H	-2.80619000	0.88953400	0.91031000
C	-3.41763900	-0.53833700	-2.64158000
H	-1.29435600	-0.78421500	-2.76589000
C	-4.49231900	-0.11686300	-1.86138600
H	-5.10574800	0.73070800	0.02398200
H	-3.58430900	-0.90725300	-3.64784800
H	-5.50168600	-0.17019900	-2.25580000

IN4

C	1.06839600	2.65493000	2.40427300
C	0.83739400	1.56422200	1.56729700
C	0.58420100	1.76336300	0.20639200
C	0.56121200	3.06773800	-0.29953000
C	0.79345500	4.15572200	0.53783500
C	1.04287600	3.95116300	1.89399300
H	1.26987800	2.48823700	3.45727500
H	0.87683200	0.56167900	1.98307500
H	0.38475600	3.23205300	-1.36010700
H	0.78356200	5.16075500	0.12916000
H	1.22048800	4.79765300	2.54877700
C	0.37852300	0.61055500	-0.78348800
H	-0.04236800	1.05302800	-1.69004200
C	1.55696600	-0.99181300	-2.30182800
C	1.65421600	-0.09753200	-1.22833100
C	2.89202000	0.11843200	-0.62400800
C	4.01693000	-0.57582900	-1.07278900
C	3.91115500	-1.48579800	-2.12194000
C	2.67699100	-1.68906700	-2.74275600
H	0.59770300	-1.14380700	-2.79365000
H	2.98467300	0.82337700	0.19574800
H	4.97765500	-0.40081000	-0.59984800

H	4.78753000	-2.02513100	-2.46556000
H	2.59105000	-2.38191000	-3.57332200
C	-0.68531200	-0.30604900	-0.22101000
C	-0.41815300	-1.29573200	0.79534800
C	0.76538700	-2.07538400	0.86873300
C	-1.37344600	-1.41921600	1.84224000
C	0.96506500	-2.93835200	1.93428500
H	1.48940900	-2.05191500	0.06652300
C	-1.14092500	-2.25665200	2.92105700
H	-2.25588200	-0.78884200	1.83365200
C	0.02471200	-3.02383500	2.96505700
H	1.85804100	-3.55300400	1.96151600
H	-1.86236600	-2.30877200	3.72892500
H	0.20157700	-3.69069600	3.80308400
C	-2.04618800	-0.11526600	-0.65598700
C	-2.93376200	-1.22026000	-0.68445900
C	-2.51149000	1.14633100	-1.10538300
C	-4.23189600	-1.06755800	-1.14884100
H	-2.57293400	-2.20243300	-0.39821400
C	-3.81982600	1.29643000	-1.52607700
H	-1.86118100	2.01358100	-1.05661100
C	-4.67795800	0.18937200	-1.55821800
H	-4.89363600	-1.92508100	-1.19776800
H	-4.18083200	2.27186800	-1.83261000
H	-5.69722700	0.31068300	-1.91119200

IN5

C	-1.89201900	3.19334000	-2.42309300
C	-1.56751900	2.01564200	-1.74894300
C	-1.07321900	2.06598300	-0.44520600
C	-0.91206900	3.30692000	0.17805900
C	-1.23703000	4.48128400	-0.49427300

C	-1.72614400	4.42697600	-1.79966400
H	-2.27652700	3.14262000	-3.43655900
H	-1.71179100	1.06224900	-2.25020700
H	-0.53961900	3.35081200	1.19949600
H	-1.11006500	5.43815900	0.00145600
H	-1.97870100	5.34165500	-2.32527500
C	-0.74787500	0.80711500	0.37751300
H	0.07310600	1.08410400	1.05149300
C	-1.78048500	0.28484000	2.62200200
C	-1.94607000	0.44725500	1.24511900
C	-3.22892900	0.31810400	0.70187700
C	-4.30883500	-0.01693400	1.51231700
C	-4.12677600	-0.21209200	2.88244600
C	-2.86135600	-0.04881800	3.43953500
H	-0.80280600	0.44206900	3.07187500
H	-3.38508800	0.47698000	-0.36178400
H	-5.29692700	-0.11872000	1.07559200
H	-4.97099000	-0.46899800	3.51355000
H	-2.71455100	-0.16633600	4.50826700
C	-0.19129200	-0.27925300	-0.53494100
C	-1.02756400	-1.39090200	-0.98145200
C	-1.76661500	-2.19732900	-0.08880500
C	-1.14339000	-1.64680000	-2.36736000
C	-2.56239400	-3.22948800	-0.56362300
H	-1.68884100	-2.03540500	0.98108000
C	-1.97926200	-2.65103100	-2.83741900
H	-0.61423000	-1.01361400	-3.07307700
C	-2.68136900	-3.45156900	-1.93676900
H	-3.09934800	-3.85702100	0.13936300
H	-2.07953400	-2.81018700	-3.90556600
H	-3.32175200	-4.24714100	-2.30350100
C	1.10139400	-0.08285700	-1.13375600

C	1.73340700	-1.13478000	-1.85413500
C	1.82348100	1.12999300	-0.96622100
C	2.98076300	-0.95416000	-2.43123000
H	1.24785500	-2.10002100	-1.93900600
C	3.06724000	1.30018700	-1.54601400
H	1.38927500	1.95669200	-0.41802900
C	3.64246500	0.26710900	-2.29274100
H	3.44212400	-1.76696100	-2.98164700
H	3.59342600	2.24018900	-1.42111200
H	4.61518600	0.41069200	-2.75325700
C	4.63604100	-0.26571300	1.04155000
C	3.51569200	-1.08780000	1.03452700
C	2.34915200	-0.69826000	1.71118500
C	2.33901600	0.52627700	2.40235200
C	3.46441400	1.34486700	2.40093500
C	4.61552100	0.95549900	1.71698100
H	5.53533300	-0.58529200	0.52425400
H	3.55606300	-2.04217200	0.51613100
H	1.45829400	0.82206600	2.96642500
H	3.44559100	2.28151300	2.94857100
H	5.49616900	1.58882200	1.72678500
C	1.13905400	-1.50745200	1.69575600
H	0.30451200	-1.37237400	2.37220600
N	1.11872000	-2.62382300	1.00725700
N	1.10260500	-3.54065000	0.34608900

TS4

C	-1.83103900	3.15553100	-2.43966600
C	-1.51136600	1.98597000	-1.74907900
C	-1.03525400	2.05032800	-0.43945600
C	-0.88909000	3.29913600	0.17225600
C	-1.20879300	4.46566100	-0.51587800

C	-1.67911000	4.39639900	-1.82732500
H	-2.20077500	3.09249000	-3.45795600
H	-1.64499000	1.02793000	-2.24301300
H	-0.53126500	3.35575000	1.19838500
H	-1.09215200	5.42783400	-0.02790400
H	-1.92760600	5.30455900	-2.36602900
C	-0.71366500	0.80594500	0.40498300
H	0.09297500	1.10476100	1.08583300
C	-1.77677100	0.29742100	2.64315500
C	-1.92256600	0.45479500	1.26301300
C	-3.19994500	0.32897600	0.70560500
C	-4.29139000	0.00195600	1.50360000
C	-4.12786500	-0.18976000	2.87657600
C	-2.86891800	-0.02990300	3.44853200
H	-0.80621900	0.45550300	3.10830200
H	-3.34241600	0.48470100	-0.36039400
H	-5.27431000	-0.09643600	1.05453300
H	-4.98111400	-0.44051100	3.49792800
H	-2.73554600	-0.14351900	4.51944500
C	-0.13461400	-0.30594600	-0.47258800
C	-1.00047600	-1.39023100	-0.95885900
C	-1.77846400	-2.19105300	-0.09666200
C	-1.12082500	-1.60404200	-2.34972600
C	-2.61678000	-3.17455800	-0.60168500
H	-1.71135100	-2.05811700	0.97849900
C	-1.99155300	-2.56370000	-2.85217400
H	-0.56382500	-0.97628500	-3.03822800
C	-2.73384900	-3.35828100	-1.98032500
H	-3.18799400	-3.79270000	0.08249200
H	-2.08722000	-2.69048400	-3.92515000
H	-3.40292700	-4.11724300	-2.37230100
C	1.14479700	-0.07917100	-1.11302300

C	1.77041500	-1.10685200	-1.86726200
C	1.86055100	1.13131100	-0.93226500
C	3.01048400	-0.90789200	-2.45690500
H	1.28405300	-2.06879100	-1.98192500
C	3.09872800	1.32146700	-1.52309100
H	1.43265700	1.94345800	-0.35818000
C	3.67191700	0.31058100	-2.29822000
H	3.46357500	-1.70478700	-3.03694400
H	3.61932300	2.26259900	-1.38300500
H	4.63767500	0.46903300	-2.76799300
C	4.59927200	-0.34845900	1.10518600
C	3.44801400	-1.12157600	1.01964800
C	2.27568600	-0.71547200	1.67519400
C	2.28944200	0.46810600	2.43229300
C	3.44584900	1.23800600	2.50973300
C	4.60212400	0.83673200	1.84174000
H	5.50152300	-0.67658300	0.59908500
H	3.46834200	-2.04575600	0.44851500
H	1.40269300	0.77216000	2.98143500
H	3.44629700	2.14503100	3.10516700
H	5.50541100	1.43340700	1.91058400
C	1.02428500	-1.45422900	1.54954100
H	0.18500100	-1.33209500	2.22485600
N	1.04226200	-2.61934700	0.92120700
N	1.04270900	-3.54909600	0.28776000

IN6

C	3.91250600	-1.47212000	-1.57021100
C	2.66906100	-1.48692300	-0.94512700
C	1.60372200	-0.73469600	-1.45172900
C	1.80833000	0.00864500	-2.61832500
C	3.05336100	0.02733400	-3.24607800

C	4.11200300	-0.70818400	-2.71940800
H	4.72561300	-2.06228100	-1.16005800
H	2.53599500	-2.08885700	-0.05185000
H	0.99408000	0.58143800	-3.04957400
H	3.18960200	0.61030300	-4.15111600
H	5.08068600	-0.69863000	-3.20812400
C	0.24154200	-0.71781900	-0.76137800
H	-0.49190300	-0.46060600	-1.52663700
C	-0.97678400	-2.85117200	-1.19555800
C	-0.17277700	-2.11698300	-0.31471700
C	0.21607000	-2.72635200	0.88432100
C	-0.22711100	-4.00984200	1.21106000
C	-1.04359900	-4.71787700	0.33258200
C	-1.40580400	-4.13747000	-0.88294000
H	-1.27934700	-2.40174900	-2.13856200
H	0.88474500	-2.21592900	1.56923300
H	0.08369200	-4.46040400	2.14836900
H	-1.38176500	-5.71727000	0.58533200
H	-2.02638000	-4.68405700	-1.58574100
C	0.12222200	0.44915800	0.32390200
C	1.31903900	0.45866400	1.29562400
C	1.27537700	-0.02027900	2.61058900
C	2.53845700	0.98293100	0.83748300
C	2.40183100	0.01387600	3.43626100
H	0.37847400	-0.46361700	3.03606100
C	3.66269600	1.01391900	1.65333200
H	2.60786700	1.37468000	-0.17284300
C	3.60101300	0.52922300	2.95988800
H	2.33187700	-0.36844500	4.44922000
H	4.59052000	1.41940800	1.26363700
H	4.47774400	0.55599700	3.59814000
C	-0.04312800	1.83981800	-0.33716300

C	0.31815300	2.99281100	0.37926300
C	-0.65527700	2.02464200	-1.58353600
C	0.08174900	4.26964700	-0.12594500
H	0.82026000	2.90401600	1.33825100
C	-0.87763600	3.30000000	-2.10054000
H	-1.00151000	1.18408100	-2.17202600
C	-0.51352900	4.43015900	-1.37470900
H	0.37995500	5.13661900	0.45440600
H	-1.34729800	3.40313900	-3.07337800
H	-0.68737400	5.42231700	-1.77756300
C	-4.44289500	1.16751200	-0.63352700
C	-3.18788000	1.26420700	-0.04108300
C	-2.56108400	0.10976000	0.44308100
C	-3.21295300	-1.12620700	0.37621500
C	-4.46089600	-1.21633900	-0.23402700
C	-5.07311400	-0.07243500	-0.74242300
H	-4.92985300	2.06260800	-1.00505100
H	-2.71093900	2.23675500	0.04546300
H	-2.74295700	-2.01737500	0.78464200
H	-4.95724600	-2.17867000	-0.29765000
H	-6.05061000	-0.14259800	-1.20798700
C	-1.20419300	0.13911200	1.11329800
H	-1.07850800	-0.77101300	1.70739400
N	-1.32751600	1.19428000	2.19003200
N	-1.48194900	2.00569200	2.91384700

TS5

C	-3.95102000	1.56800200	-1.43219600
C	-2.68365400	1.55022900	-0.85731900
C	-1.65468000	0.78427900	-1.41565400
C	-1.92138300	0.05913400	-2.58139000
C	-3.19078600	0.07236200	-3.15826100

C	-4.21210800	0.82221400	-2.58090600
H	-4.73494300	2.16846000	-0.98220800
H	-2.50490900	2.13713900	0.03763100
H	-1.13725200	-0.52378400	-3.05270600
H	-3.37506300	-0.49709700	-4.06336800
H	-5.19985000	0.83736700	-3.02969700
C	-0.26541800	0.73158800	-0.78773100
H	0.42730200	0.45302500	-1.58187500
C	1.06033200	2.80135500	-1.22892100
C	0.21571100	2.11305300	-0.35000100
C	-0.14397000	2.74346800	0.84800500
C	0.36140100	4.00367000	1.17208200
C	1.21507900	4.66757800	0.29347100
C	1.55344500	4.06550000	-0.91779700
H	1.34419700	2.33457000	-2.16924300
H	-0.83094700	2.26307400	1.53704600
H	0.07238200	4.47140900	2.10793400
H	1.60149800	5.64984000	0.54428300
H	2.20464100	4.57716800	-1.61918300
C	-0.12482300	-0.45229100	0.29944200
C	-1.30009200	-0.44439900	1.29605200
C	-1.22533200	0.04957700	2.60375600
C	-2.53174700	-0.96474900	0.86779900
C	-2.33589500	0.03302000	3.45155300
H	-0.31504200	0.48533300	3.00679400
C	-3.63917200	-0.97983900	1.70637100
H	-2.62469500	-1.36414200	-0.13773600
C	-3.54766900	-0.47994500	3.00563500
H	-2.24298000	0.42564500	4.45872100
H	-4.57741500	-1.38343200	1.34038700
H	-4.41142700	-0.49351300	3.66178700
C	-0.00007800	-1.84096100	-0.37434300

C	-0.32260200	-2.98823800	0.36767400
C	0.53011200	-2.03203300	-1.65580900
C	-0.12240400	-4.26798700	-0.14489800
H	-0.76539800	-2.89132700	1.35456700
C	0.71612400	-3.31089900	-2.17979700
H	0.84289500	-1.19601100	-2.26867200
C	0.39497700	-4.43633000	-1.42726400
H	-0.38744300	-5.13139800	0.45647000
H	1.12327200	-3.42006800	-3.17976300
H	0.54101400	-5.43090400	-1.83510600
C	4.39309700	-1.21735400	-0.73540400
C	3.12552200	-1.29649700	-0.17208100
C	2.54374700	-0.14847400	0.38824900
C	3.25990600	1.05805200	0.41690700
C	4.51585400	1.13432200	-0.17574000
C	5.08046900	-0.00166100	-0.75276900
H	4.84853100	-2.10690000	-1.15677000
H	2.60883300	-2.25056900	-0.14495200
H	2.82061600	1.94211600	0.87421700
H	5.05592800	2.07478000	-0.17148600
H	6.06726300	0.05306200	-1.20083600
C	1.19560700	-0.12061800	1.01190500
H	1.09650200	0.74271400	1.67007400
N	1.32894700	-1.30159800	2.25518500
N	1.60265300	-2.06659000	2.99338500

IN7

C	-4.34097600	0.00518600	-1.18122900
C	-3.08431500	0.12222500	-0.58611500
C	-1.98428400	0.55907500	-1.33287400
C	-2.18902300	0.89792900	-2.67870700
C	-3.44333100	0.78985100	-3.26962700

C	-4.52671000	0.33306500	-2.52087000
H	-5.17911900	-0.34016600	-0.58461800
H	-2.98687200	-0.12600600	0.46345400
H	-1.35135700	1.25984300	-3.27188900
H	-3.57269200	1.06189700	-4.31208900
H	-5.50690100	0.24053000	-2.97647000
C	-0.58612800	0.80843600	-0.78147500
H	0.07922300	0.88470400	-1.65029300
C	0.58370700	3.01107000	-0.36543000
C	-0.49518000	2.16383900	-0.07675200
C	-1.47524500	2.61919600	0.81192800
C	-1.35175200	3.86059800	1.43227200
C	-0.25532400	4.67875300	1.16293800
C	0.70901900	4.25470600	0.25173700
H	1.32559600	2.70595600	-1.10068200
H	-2.34979000	2.01024500	1.01450200
H	-2.12475600	4.19548600	2.11632700
H	-0.16732800	5.64901100	1.64037300
H	1.54681900	4.89830100	0.00294300
C	0.07744700	-0.41976400	0.05718100
C	-0.79128100	-0.88193900	1.24943600
C	-0.97153600	-0.07689400	2.38146500
C	-1.44308400	-2.11965300	1.20698500
C	-1.78458500	-0.49088400	3.43461200
H	-0.50121200	0.89826100	2.45978500
C	-2.24732100	-2.53911000	2.26451700
H	-1.34023700	-2.76141600	0.33950500
C	-2.42256400	-1.72789400	3.38342300
H	-1.90965000	0.15461700	4.29791700
H	-2.73976100	-3.50427000	2.20766200
H	-3.04675300	-2.05699800	4.20747500
C	0.44373800	-1.61032100	-0.84274500

C	1.29503200	-2.58323600	-0.29659800
C	-0.00391500	-1.77729100	-2.15303800
C	1.71419000	-3.67480100	-1.04637900
H	1.62787600	-2.48762000	0.73470700
C	0.41282700	-2.87841600	-2.90746400
H	-0.68612200	-1.06948700	-2.60532300
C	1.27669100	-3.82322500	-2.36438500
H	2.37469200	-4.41246400	-0.60245000
H	0.05059600	-2.99164100	-3.92407400
H	1.59808000	-4.67493900	-2.95454100
C	4.15178500	0.48176300	-1.84016000
C	2.92629000	0.09494600	-1.34130600
C	2.57522300	0.42422500	0.00364900
C	3.51897300	1.12583800	0.81172900
C	4.75067800	1.49167300	0.30545100
C	5.06110000	1.17147700	-1.02055700
H	4.42207000	0.24201800	-2.86231300
H	2.23628700	-0.46016400	-1.96598800
H	3.24787700	1.38137300	1.83238700
H	5.46557400	2.02343200	0.92263200
H	6.02736700	1.45801600	-1.42548700
C	1.32526700	0.19461300	0.59580900
H	1.25187500	0.56948000	1.61551000
N	1.53636700	-1.88308100	3.27567500
N	0.98594000	-2.63952100	3.85239900

TS6

C	-3.97791900	1.02910400	-1.44084700
C	-2.71420900	1.24988200	-0.89951700
C	-1.61940600	0.49124100	-1.32411500
C	-1.80646300	-0.46710200	-2.32230800
C	-3.07139800	-0.69165600	-2.86556100

C	-4.16202400	0.05197000	-2.41926900
H	-4.81958700	1.62280500	-1.09929600
H	-2.57890000	2.01042600	-0.13547100
H	-0.96145900	-1.04539000	-2.68619600
H	-3.20071300	-1.43704400	-3.64358600
H	-5.14662000	-0.11737300	-2.84259000
C	-0.24170800	0.67863800	-0.69470400
H	0.49620400	0.44000900	-1.46606300
C	-0.06811500	3.07076200	-1.37960800
C	0.00817600	2.14673600	-0.32805400
C	0.34777900	2.60627800	0.94231800
C	0.60028000	3.96258300	1.16472400
C	0.52061900	4.87194800	0.11676700
C	0.18687900	4.41944800	-1.16149000
H	-0.33863600	2.72698300	-2.37521500
H	0.43077900	1.92209400	1.77732400
H	0.86190600	4.30035600	2.16214100
H	0.71740800	5.92464000	0.28969600
H	0.12343500	5.11875100	-1.98878200
C	0.02009800	-0.27159800	0.49108400
C	-1.05037900	-0.41140300	1.53902800
C	-0.75581200	-0.06295400	2.86402500
C	-2.32866300	-0.90813200	1.24055600
C	-1.72446500	-0.16195200	3.86093600
H	0.23179200	0.30475500	3.12894900
C	-3.28815500	-1.02167400	2.23958700
H	-2.56432400	-1.24302000	0.23581700
C	-2.99444600	-0.63796000	3.54937400
H	-1.48035800	0.12826200	4.87715600
H	-4.26843600	-1.41621900	1.99392300
H	-3.74959900	-0.72241100	4.32385200
C	0.57867900	-1.87757600	-0.03366300

C	-0.01659900	-2.96711100	0.63950000
C	1.05220900	-2.05752200	-1.35219000
C	-0.15694800	-4.18911800	0.00376400
H	-0.37949500	-2.83890600	1.65471400
C	0.90369200	-3.27946400	-1.98380300
H	1.58409000	-1.25652000	-1.85624000
C	0.29160400	-4.34200800	-1.31089200
H	-0.61524400	-5.02094700	0.52658900
H	1.27663200	-3.41411100	-2.99306200
H	0.17574000	-5.29864300	-1.81077300
C	4.33174000	1.03347300	-0.76071500
C	3.00976500	0.78976900	-0.38733900
C	2.70143100	-0.32884300	0.39391900
C	3.73679000	-1.18616300	0.79688500
C	5.05036500	-0.94346200	0.41814600
C	5.35050700	0.17061400	-0.36756400
H	4.56031700	1.91095300	-1.35631400
H	2.24878800	1.50026000	-0.68255500
H	3.50459400	-2.05580400	1.40774200
H	5.83850000	-1.61660500	0.73802100
H	6.37562500	0.36817300	-0.66272800
C	1.33704900	-0.71059300	0.87230300
H	1.37185400	-1.13239300	1.87527900

IN8

C	2.96217500	-2.21812900	-2.62898800
C	1.71976000	-1.95045600	-2.05129700
C	1.50887600	-0.76125800	-1.35087700
C	2.55853800	0.16203500	-1.24656800
C	3.79739600	-0.11049200	-1.81877900
C	4.00470800	-1.30371300	-2.51173900
H	3.10893400	-3.14605900	-3.17188900

H	0.91888400	-2.67510400	-2.15199500
H	2.42285700	1.09310200	-0.70387500
H	4.60105200	0.61264800	-1.72373300
H	4.97057000	-1.51533500	-2.95826600
C	0.13424600	-0.41905000	-0.76473900
H	-0.28318600	0.37768200	-1.38612500
C	-1.93127100	-1.43136900	-1.77644800
C	-0.89423400	-1.56200800	-0.84833100
C	-0.81478400	-2.72185600	-0.07230500
C	-1.75920500	-3.73563100	-0.22655700
C	-2.79032200	-3.59906700	-1.15443500
C	-2.86978000	-2.44670500	-1.93532600
H	-2.01066300	-0.52631400	-2.37314500
H	-0.01379600	-2.85497000	0.64822100
H	-1.68585000	-4.63291300	0.37900100
H	-3.52596300	-4.38811900	-1.27031100
H	-3.66503600	-2.33364500	-2.66475000
C	0.08960400	0.09154500	0.64967700
C	0.70932800	-0.57766400	1.76086400
C	0.08745000	-0.48992200	3.03695500
C	1.85603300	-1.40735900	1.63104100
C	0.57296700	-1.20932200	4.11541600
H	-0.82209800	0.08589700	3.16609300
C	2.35717600	-2.08859500	2.72721200
H	2.36976000	-1.48186600	0.68202000
C	1.71753500	-1.99658700	3.96678900
H	0.06422400	-1.15858500	5.07157600
H	3.24909000	-2.69565300	2.61871400
H	2.11110500	-2.54201600	4.81895100
C	0.38016200	2.36457800	0.34672600
C	1.39927400	2.78418800	1.21078900
C	0.38971300	2.80449800	-0.98421100

C	2.39834300	3.64537700	0.76510100
H	1.41045000	2.43155200	2.23974600
C	1.38414300	3.67159400	-1.42633800
H	-0.38591700	2.49098200	-1.67581700
C	2.39224000	4.08962600	-0.55627800
H	3.17662600	3.96875700	1.44801700
H	1.37395800	4.01733700	-2.45453200
H	3.16872600	4.76081800	-0.90804100
C	-4.29656700	0.44350400	0.29977400
C	-2.94940500	0.37374500	0.64116700
C	-2.09050600	1.44983100	0.38760100
C	-2.61620800	2.61594200	-0.17422100
C	-3.96622200	2.68553500	-0.51657100
C	-4.80722400	1.59846200	-0.28999700
H	-4.94395800	-0.40512900	0.49530700
H	-2.56769400	-0.53625500	1.10020400
H	-1.98546900	3.48478300	-0.32888800
H	-4.35990700	3.59750100	-0.95301200
H	-5.85665500	1.65572100	-0.55926000
C	-0.65947000	1.37282500	0.90188200
H	-0.68684100	1.57057700	1.97385700

IN9

C	-0.96156400	-3.74608200	2.08834200
C	-0.17355700	-3.01547600	1.20141700
C	0.10810400	-1.66534100	1.43680500
C	-0.37082200	-1.08793500	2.61776000
C	-1.14604200	-1.81863600	3.51709400
C	-1.45515800	-3.14981500	3.24828000
H	-1.16777000	-4.79166200	1.88349400
H	0.24592600	-3.51150700	0.32950700
H	-0.11240700	-0.06354900	2.86698700

H	-1.49701500	-1.34924200	4.43049300
H	-2.05522700	-3.72385200	3.94640700
C	1.05423000	-0.85397900	0.54494700
H	1.66709100	-0.26405200	1.23862800
C	3.24058600	-2.08308800	0.52963200
C	2.09084500	-1.72973700	-0.18596100
C	1.92933600	-2.19188400	-1.49325400
C	2.90731900	-3.00049600	-2.07716400
C	4.04852100	-3.34734100	-1.36190800
C	4.20984900	-2.89026900	-0.05186300
H	3.37563300	-1.71691800	1.54411300
H	1.05722500	-1.92371300	-2.08092200
H	2.77320800	-3.35129900	-3.09525200
H	4.81016400	-3.97021700	-1.81912700
H	5.09665100	-3.15680300	0.51393800
C	-0.61339300	0.03382200	-1.24765200
C	-1.05396500	1.10013600	-2.09725100
C	-1.29642600	-1.21359500	-1.35038700
C	-2.06785400	0.90384700	-3.00659800
H	-0.61901600	2.08876100	-2.00817900
C	-2.27371200	-1.41639400	-2.31054000
H	-1.02179000	-2.03362300	-0.70411000
C	-2.66171000	-0.36406000	-3.13598100
H	-2.40271600	1.72710600	-3.62811900
H	-2.75827800	-2.38274100	-2.39037900
H	-3.44341000	-0.51852900	-3.87380400
C	0.82978400	2.59780000	0.13638800
C	0.75088400	3.83632500	-0.50753000
C	0.41292600	2.50212300	1.46644100
C	0.26398900	4.95442300	0.16456800
H	1.08631600	3.93053800	-1.53792800
C	-0.07847900	3.61902200	2.14113700

H	0.48917100	1.55707900	1.99217500
C	-0.15794400	4.84766400	1.48920500
H	0.21089500	5.90866400	-0.34881600
H	-0.39327200	3.52871600	3.17620000
H	-0.54245000	5.71768800	2.01053500
C	5.05949100	0.46909500	-1.21451400
C	3.70457600	0.71770100	-1.40469100
C	2.91071500	1.19598400	-0.35643000
C	3.50517700	1.45464800	0.87994400
C	4.86389200	1.20231500	1.07401900
C	5.64109300	0.70332800	0.03172700
H	5.65974500	0.09389500	-2.03695600
H	3.25360100	0.52297200	-2.37538600
H	2.92042500	1.87292500	1.69417500
H	5.31501300	1.41004500	2.03892600
H	6.69835400	0.51247000	0.18410500
C	1.42317700	1.41002800	-0.62655800
H	1.35885400	1.66507300	-1.68706800
C	0.54055600	0.17385700	-0.43507000
C	-5.42580200	-0.44518500	-1.14031000
C	-4.61021500	0.53380600	-0.58399300
C	-3.67295500	0.19860200	0.40567200
C	-3.59159100	-1.13733300	0.83572000
C	-4.42194600	-2.10972400	0.28314800
C	-5.33613600	-1.77341800	-0.71343300
H	-6.15302500	-0.16731500	-1.89735000
H	-4.70804500	1.56441500	-0.91600700
H	-2.87835700	-1.41014600	1.60797600
H	-4.35021200	-3.13366600	0.63792100
H	-5.98754300	-2.52931600	-1.13952600
C	-2.76471900	1.18613300	0.97905900
H	-2.17036800	1.02645700	1.86724300

N	-2.61517400	2.32774900	0.37297100
N	-2.51678700	3.28169600	-0.23657300

TS7

C	-2.16121500	-2.78456100	2.72962700
C	-1.23722600	-2.39440300	1.76278700
C	-0.59210200	-1.15361900	1.83432300
C	-0.83964100	-0.36346800	2.96378200
C	-1.74966600	-0.75445800	3.94557300
C	-2.43217800	-1.96165200	3.82173000
H	-2.64923100	-3.74985100	2.64085100
H	-0.99568700	-3.08641200	0.96151100
H	-0.30217000	0.56672700	3.11296100
H	-1.91188600	-0.11899000	4.81021000
H	-3.14054400	-2.27274100	4.58228300
C	0.49348400	-0.71826500	0.83055400
H	1.19748900	-0.13185100	1.42580500
C	2.43381700	-2.24180900	1.22563900
C	1.33730600	-1.94720000	0.40760100
C	1.05621000	-2.80460800	-0.65883700
C	1.84813700	-3.92912400	-0.89334500
C	2.93575500	-4.21231900	-0.07321500
C	3.22585900	-3.36114500	0.99132600
H	2.67502200	-1.58298000	2.05623100
H	0.23612100	-2.61163000	-1.33873500
H	1.61128100	-4.57956200	-1.72935300
H	3.55317700	-5.08416600	-0.26241200
H	4.07286600	-3.56266500	1.63924000
C	-0.66288600	-0.19748200	-1.47827800
C	-0.61185900	0.41267500	-2.76170000
C	-1.63941900	-1.21096700	-1.29670400
C	-1.43267000	-0.01705900	-3.79566600

H	0.09556500	1.19900100	-2.99077000
C	-2.45394900	-1.63923300	-2.33261300
H	-1.75616000	-1.66658200	-0.32615800
C	-2.34682300	-1.05232500	-3.59282100
H	-1.34767100	0.45159900	-4.77026600
H	-3.18022400	-2.42447200	-2.15139300
H	-2.97602900	-1.39083100	-4.40991000
C	1.42889300	2.47652300	0.25232800
C	1.90250500	3.63516000	-0.37791700
C	1.17093500	2.53626600	1.62056400
C	2.08775700	4.81808800	0.32922100
H	2.12756600	3.60793400	-1.44171000
C	1.34755300	3.72356400	2.33676500
H	0.84622000	1.65716900	2.15787400
C	1.80127700	4.86993100	1.69395600
H	2.45178600	5.70054500	-0.18659000
H	1.13836500	3.74110500	3.40172900
H	1.93856400	5.79178400	2.24880000
C	4.05736300	-1.02909600	-1.99696200
C	2.85853800	-0.33625900	-1.86499300
C	2.64840400	0.52058900	-0.78042400
C	3.66827900	0.69518500	0.15648500
C	4.87464500	0.00949600	0.01760100
C	5.06923800	-0.85978600	-1.05183200
H	4.20073800	-1.70221700	-2.83599700
H	2.07020400	-0.48821000	-2.59925000
H	3.53582600	1.37498800	0.99220700
H	5.66215800	0.15894500	0.74929000
H	6.00504300	-1.39949600	-1.15389600
C	1.29227900	1.24401600	-0.64680700
H	1.09647500	1.67009700	-1.62780300
C	0.19019700	0.20264700	-0.37613400

C	-4.80511300	0.66920900	-1.55661200
C	-3.62944100	1.31526200	-1.19301900
C	-3.01712000	1.03267200	0.03857900
C	-3.61429100	0.09774700	0.90119600
C	-4.78619400	-0.54874100	0.52520000
C	-5.38481900	-0.27003700	-0.70413600
H	-5.27098900	0.90362400	-2.50822000
H	-3.19950900	2.04195200	-1.87668700
H	-3.16343500	-0.11927500	1.86343800
H	-5.23635200	-1.26668800	1.20328600
H	-6.30449500	-0.77027700	-0.98916200
C	-1.74808400	1.65491400	0.42899300
H	-1.41318300	1.73247200	1.45449000
N	-1.37322900	2.67289500	-0.35103400
N	-1.07953100	3.42039000	-1.13364800

IN10

C	-3.20157700	-2.15409900	2.00584300
C	-2.04741100	-1.92691200	1.26432500
C	-1.14012000	-0.92182000	1.62747200
C	-1.41637600	-0.19060400	2.78827000
C	-2.56992900	-0.41671100	3.54212300
C	-3.47436000	-1.39519400	3.14538800
H	-3.88796300	-2.93595300	1.69665300
H	-1.86517000	-2.55378600	0.40079200
H	-0.70619700	0.55316700	3.14005300
H	-2.74585200	0.16121900	4.44395700
H	-4.37122400	-1.58289500	3.72671700
C	0.17657500	-0.64422600	0.88437900
H	0.70604100	0.03954500	1.55183300
C	1.12364800	-2.58481600	2.14142100
C	1.08071000	-1.89968800	0.91717700

C	1.93375000	-2.33932500	-0.09852100
C	2.79098200	-3.42128300	0.09809600
C	2.82136000	-4.09049900	1.31703700
C	1.98053400	-3.66310800	2.34187100
H	0.48048000	-2.27336800	2.95915300
H	1.96277100	-1.84137200	-1.05680000
H	3.44359300	-3.72889900	-0.71308500
H	3.49084300	-4.93084700	1.46806200
H	1.98745900	-4.16705400	3.30296100
C	-0.06651400	-0.52954700	-1.78265700
C	0.07609000	0.12231800	-3.01900600
C	-0.47397100	-1.86737300	-1.81883900
C	-0.20016300	-0.52321000	-4.22397600
H	0.40653400	1.15323000	-3.08903800
C	-0.74337400	-2.52238100	-3.01770800
H	-0.55392500	-2.42960900	-0.90155100
C	-0.62004700	-1.85041900	-4.23067300
H	-0.07942700	0.01767900	-5.15695400
H	-1.04854200	-3.56344400	-2.99664400
H	-0.83311200	-2.35547100	-5.16676800
C	1.17558700	2.58256400	0.28715800
C	1.52477400	3.75948400	-0.39239300
C	0.83706400	2.69891100	1.64103100
C	1.52154300	4.99935300	0.23943000
H	1.82177700	3.69914900	-1.43825700
C	0.82378900	3.93982800	2.27977800
H	0.59170900	1.82486600	2.23163200
C	1.16256100	5.09576800	1.58289700
H	1.80308400	5.88751900	-0.31681600
H	0.55781700	3.99525000	3.33057900
H	1.15587900	6.05875700	2.08205100
C	4.61796000	-0.32749800	-1.38909100

C	3.36450900	0.27177600	-1.49338700
C	2.65527700	0.65358700	-0.35031100
C	3.25101500	0.47195700	0.90088600
C	4.49860400	-0.13524200	1.00981200
C	5.18215200	-0.54480400	-0.13411300
H	5.15236800	-0.62011800	-2.28720500
H	2.92619700	0.42835800	-2.47653900
H	2.74928200	0.80616800	1.80465200
H	4.93907300	-0.28419400	1.99016200
H	6.15570000	-1.01592100	-0.04731400
C	1.27552700	1.27998500	-0.51903500
H	1.27632300	1.62929000	-1.55155900
C	0.06363300	0.23552000	-0.44930300
C	-4.44883200	-0.42854700	-1.77313000
C	-3.11444700	-0.04892600	-1.65859700
C	-2.68082400	0.60164300	-0.49555200
C	-3.60571100	0.92253300	0.50595200
C	-4.93520400	0.53670500	0.38492800
C	-5.35662500	-0.15012100	-0.75203600
H	-4.77927900	-0.93858000	-2.67168000
H	-2.43881000	-0.25571600	-2.47956300
H	-3.27728300	1.44069200	1.40256200
H	-5.63638200	0.76954300	1.17916300
H	-6.39354800	-0.45432300	-0.85031800
C	-1.26122400	1.07755000	-0.26472400
H	-1.20782000	1.62205700	0.68350400
N	-1.17673200	2.28820300	-1.24510400
N	-1.29012700	3.11269400	-1.96171600

TS8

C	3.19364800	-2.18226400	-1.97678900
C	2.03264800	-1.94845700	-1.24822000

C	1.13266300	-0.94193200	-1.62532100
C	1.42211200	-0.21683800	-2.78663100
C	2.58243500	-0.45010400	-3.52769100
C	3.47999700	-1.42919600	-3.11701400
H	3.87483600	-2.96445700	-1.65709500
H	1.83990600	-2.56877700	-0.38208400
H	0.71828100	0.52873500	-3.14736100
H	2.76955800	0.12329100	-4.43015700
H	4.38249900	-1.62165700	-3.68797600
C	-0.18479200	-0.65066200	-0.88864500
H	-0.70435700	0.04043200	-1.55608800
C	-1.14358500	-2.58433500	-2.14644500
C	-1.10251700	-1.89585900	-0.92410800
C	-1.96830700	-2.32223100	0.08641600
C	-2.83633300	-3.39484100	-0.11335900
C	-2.86517700	-4.06744300	-1.33055200
C	-2.01156300	-3.65322800	-2.35011900
H	-0.49036700	-2.28337800	-2.96008800
H	-1.99769000	-1.82139500	1.04315600
H	-3.49851900	-3.69233600	0.69382100
H	-3.54318900	-4.90045200	-1.48415800
H	-2.01688400	-4.16017600	-3.30963300
C	0.05669700	-0.53860500	1.78135500
C	-0.06585200	0.12085800	3.01551700
C	0.43267400	-1.88553400	1.82042800
C	0.19858200	-0.52669300	4.22191800
H	-0.36894400	1.16032700	3.08172000
C	0.69053500	-2.54238700	3.02104300
H	0.49774800	-2.45307800	0.90505800
C	0.58668400	-1.86359400	4.23200500
H	0.09445500	0.02033900	5.15331500
H	0.97129300	-3.59037100	3.00275800

H	0.79080100	-2.37033200	5.16918200
C	-1.14586800	2.58809600	-0.29389500
C	-1.48366100	3.76985000	0.38297700
C	-0.80804900	2.69803300	-1.64857400
C	-1.46970900	5.00805300	-0.25178700
H	-1.77966800	3.71470600	1.42935000
C	-0.78374300	3.93725000	-2.29019500
H	-0.57141200	1.82057300	-2.23774200
C	-1.11108100	5.09797500	-1.59577400
H	-1.74253900	5.90008400	0.30261500
H	-0.51837000	3.98770900	-3.34140200
H	-1.09599200	6.05965100	-2.09727900
C	-4.61854700	-0.27604800	1.39701400
C	-3.35740400	0.30761400	1.49736100
C	-2.64673400	0.67955100	0.35188700
C	-3.24815800	0.50426300	-0.89752100
C	-4.50363600	-0.08716700	-1.00244800
C	-5.18900600	-0.48717000	0.14377600
H	-5.15412700	-0.56120100	2.29683700
H	-2.91425000	0.45966800	2.47904700
H	-2.74461500	0.83141800	-1.80287900
H	-4.94877300	-0.23137300	-1.98140100
H	-6.16873500	-0.94589400	0.06016700
C	-1.25919500	1.28913200	0.51585900
H	-1.25128500	1.64032700	1.54777300
C	-0.06029900	0.22903400	0.44787200
C	4.44202600	-0.47671100	1.75726400
C	3.10711900	-0.10169000	1.64391600
C	2.67583400	0.57512000	0.49313300
C	3.60705600	0.92025700	-0.49674800
C	4.93688900	0.53600400	-0.37829100
C	5.35422500	-0.17275200	0.74663000

H	4.77074000	-1.00419500	2.64629000
H	2.42879100	-0.32913000	2.45654200
H	3.28111400	1.45715700	-1.38336800
H	5.64159200	0.78754700	-1.16357600
H	6.39181300	-0.47510500	0.84425100
C	1.26675300	1.03877200	0.25023200
H	1.21432700	1.61020400	-0.68071300
N	1.18514900	2.31467600	1.27159600
N	1.36196500	3.14338300	1.97015600

IN11

C	1.41601700	4.67241800	-0.25504700
C	1.52199000	3.31144100	0.02122100
C	1.08636000	2.35461900	-0.90371000
C	0.59332000	2.81033400	-2.13473100
C	0.48775600	4.17127500	-2.41815500
C	0.89100900	5.10880400	-1.47096000
H	1.76231800	5.39482400	0.47710200
H	1.96458000	2.99431900	0.95958900
H	0.31539000	2.08901100	-2.90281500
H	0.11161800	4.49488600	-3.38339300
H	0.82012000	6.16966900	-1.68680100
C	1.20021800	0.84813900	-0.65647500
H	1.16839600	0.39466200	-1.65355900
C	3.53913300	0.08622000	-1.06106600
C	2.58577300	0.49623700	-0.12111400
C	2.98943500	0.62722500	1.21316300
C	4.29406100	0.31174700	1.59410100
C	5.22331300	-0.11999900	0.65156600
C	4.84199500	-0.22094200	-0.68522700
H	3.24943700	-0.01498000	-2.10438700
H	2.30182500	0.97185200	1.97637000

H	4.58239400	0.41353700	2.63571700
H	6.23654500	-0.36477600	0.95322300
H	5.55492000	-0.54814500	-1.43547700
C	-0.06690300	0.45049500	1.60345900
C	0.14135900	-0.53368300	2.57320000
C	-0.34394000	1.75324400	2.04489900
C	0.11475300	-0.22324200	3.93276900
H	0.32199700	-1.56267100	2.29408100
C	-0.36846300	2.06903400	3.40037700
H	-0.54574800	2.54009500	1.32438800
C	-0.13092100	1.08078100	4.35421600
H	0.28837200	-1.00921400	4.66119200
H	-0.58019300	3.08780000	3.70863300
H	-0.14771600	1.32215800	5.41173400
C	-1.25268000	-1.52595000	-1.38831600
C	-2.39508900	-2.28138800	-1.10230100
C	-1.20289400	-0.83891300	-2.61307000
C	-3.48225100	-2.28951500	-1.96886400
H	-2.44750000	-2.83101600	-0.16608400
C	-2.28747100	-0.85535100	-3.49339300
H	-0.31005000	-0.28910400	-2.89695000
C	-3.43784900	-1.56287100	-3.16430400
H	-4.36957400	-2.86064700	-1.71471800
H	-2.22488100	-0.31751800	-4.43409600
H	-4.28745200	-1.57149600	-3.83910800
C	3.18463200	-3.18200900	0.07750000
C	2.01766400	-2.48515300	0.36328700
C	1.11676100	-2.12147000	-0.65011300
C	1.42634200	-2.50154500	-1.96062700
C	2.59911100	-3.20011100	-2.25224200
C	3.48500400	-3.54004200	-1.23670500
H	3.86532100	-3.43591900	0.88359700

H	1.83833600	-2.19801100	1.39144500
H	0.74760300	-2.28241200	-2.77607400
H	2.80879600	-3.48366600	-3.27860600
H	4.39776200	-4.08170100	-1.46243100
C	-0.17551700	-1.38175800	-0.31276100
H	-0.60432000	-1.85429100	0.57527900
C	-0.04656600	0.18687600	0.08785900
C	-4.40981100	0.03683600	1.47938100
C	-3.07168500	0.05396000	1.14732300
C	-2.63975200	0.74750300	-0.01745000
C	-3.60629300	1.40394200	-0.82536500
C	-4.94749300	1.38794300	-0.47875300
C	-5.34456700	0.70772600	0.67361300
H	-4.74127900	-0.48779900	2.36874400
H	-2.35036900	-0.45412500	1.77648700
H	-3.27809000	1.91929300	-1.72435000
H	-5.67998200	1.89649600	-1.09508800
H	-6.39388700	0.69594500	0.95284600
C	-1.30867600	0.78205900	-0.48969100
H	-1.18190900	1.38569600	-1.38469800
N	-2.09706000	-2.99948700	2.68156600
N	-1.99092500	-3.52797800	3.63889900

IN12

C	1.78041800	-3.58479300	2.67924600
C	1.87697300	-2.42577200	1.90691600
C	0.85734200	-1.47381700	1.93562800
C	-0.24822400	-1.69212300	2.76994300
C	-0.34623200	-2.84737600	3.53853300
C	0.67051800	-3.80206000	3.49051500
H	2.58310300	-4.31438000	2.64931800
H	2.75505000	-2.26707100	1.29034200

H	-1.04620500	-0.95262100	2.81655900
H	-1.20842400	-2.99876200	4.17973800
H	0.60095100	-4.70239800	4.09176800
C	0.90409600	-0.19248500	1.10939700
H	0.62877900	0.64143300	1.77829800
C	2.98193000	1.20634400	1.36750300
C	2.31477500	0.21474900	0.64268600
C	2.96831400	-0.40582000	-0.42381900
C	4.26122300	-0.01833500	-0.77396600
C	4.91342400	0.98507500	-0.06225600
C	4.27188100	1.59113400	1.01715900
H	2.48618600	1.69380900	2.20275300
H	2.49096200	-1.19997600	-0.98395800
H	4.75602300	-0.50943000	-1.60597300
H	5.91758600	1.28797300	-0.34022100
H	4.77181400	2.36881800	1.58531100
C	0.11029300	-1.78052900	-1.48500900
C	0.46431800	-1.05372200	-2.62969700
C	0.70135400	-3.03201300	-1.25868000
C	1.38012600	-1.57719300	-3.54015100
H	0.03490500	-0.07795000	-2.82840200
C	1.61272600	-3.55283500	-2.17089300
H	0.43714600	-3.59909500	-0.37074900
C	1.95957400	-2.82321200	-3.30983300
H	1.64284400	-1.00712800	-4.42504300
H	2.05145900	-4.52915500	-1.99372600
H	2.67531800	-3.22851200	-4.01743300
C	-1.86731600	1.62270300	0.33230500
C	-2.97130000	2.03752000	-0.42141300
C	-1.97973800	1.55605900	1.72404100
C	-4.16442400	2.37442100	0.20442400
H	-2.89609300	2.08985700	-1.50368200

C	-3.18361500	1.88336000	2.35049100
H	-1.13761600	1.27681600	2.34823400
C	-4.27889600	2.28529500	1.59313200
H	-5.00916500	2.69977600	-0.39389900
H	-3.25598100	1.82900500	3.43180700
H	-5.21480000	2.53802400	2.08023500
C	2.44502500	3.38762900	-1.42565800
C	1.45947300	2.40887100	-1.40909600
C	0.47985000	2.39785400	-0.40863800
C	0.48584000	3.40377200	0.55718200
C	1.47533600	4.38913800	0.54025700
C	2.45745500	4.38079900	-0.44439600
H	3.20343600	3.37576800	-2.20168400
H	1.46874400	1.63038200	-2.16829700
H	-0.28646200	3.44103400	1.31827600
H	1.46663300	5.16857000	1.29521900
H	3.22447200	5.14828500	-0.45661100
C	-0.56576300	1.29145500	-0.43393600
H	-0.90892600	1.15121100	-1.46651100
C	-5.05845400	-0.82230600	-1.51218200
C	-4.66867100	-1.07716100	-0.19624300
C	-3.32179800	-1.22504100	0.11093100
C	-2.34380200	-1.11167000	-0.88524800
C	-2.74101300	-0.88723600	-2.20249600
C	-4.09462500	-0.73721700	-2.51215500
H	-6.10943600	-0.70938200	-1.75732300
H	-5.41350200	-1.16355700	0.58821100
H	-3.02168300	-1.42203700	1.13807800
H	-2.01274500	-0.85163900	-3.00508300
H	-4.39148700	-0.56711500	-3.54197600
C	-0.89876500	-1.26714900	-0.43958200
H	-0.87948900	-2.01259300	0.35738500

C	-0.16686400	-0.06430300	0.05989600

IN13

C	2.80648300	-0.66504100	-2.76770000
C	1.65233700	-0.49784600	-2.00048300
C	0.43556900	-1.01931000	-2.44224900
C	0.38019500	-1.69487600	-3.66659200
C	1.53371800	-1.86937700	-4.42321900
C	2.75110100	-1.35423500	-3.97499000
H	3.74831900	-0.25441400	-2.41969800
H	1.73448100	0.05154400	-1.06386700
H	-0.56647100	-2.09871100	-4.01669400
H	1.48030800	-2.40268700	-5.36657700
H	3.65024000	-1.48665600	-4.56729400
C	-0.89254700	-0.85841800	-1.67649000
H	-1.53316900	-0.34325200	-2.40956500
C	-3.01337900	-2.03038000	-1.10834600
C	-1.63769400	-2.13571800	-1.33997800
C	-1.03137600	-3.39341300	-1.31895600
C	-1.78702800	-4.52248600	-1.02181700
C	-3.15165300	-4.40859800	-0.74914000
C	-3.76800300	-3.16063400	-0.79975400
H	-3.50279000	-1.06170900	-1.20118700
H	0.03204000	-3.48661500	-1.51577000
H	-1.30715300	-5.49561600	-0.99595000
H	-3.73482000	-5.29372000	-0.51678900
H	-4.83431000	-3.06860500	-0.62073200
C	0.31433500	2.58468900	-0.64306700
C	0.43497400	3.02883600	0.67749700
C	1.12077700	3.18008400	-1.62229300
C	1.31660400	4.06098600	1.00180200
H	-0.17187700	2.60112400	1.46672500

C	1.99773200	4.21379000	-1.30149500
H	1.05371400	2.84057000	-2.65295400
C	2.09144700	4.66564400	0.01416800
H	1.38569600	4.39856300	2.03129700
H	2.60086600	4.66748900	-2.08115600
H	2.76606000	5.47635500	0.26863000
C	-1.54935700	0.46632500	1.81791400
C	-1.06780400	0.95454400	3.03664300
C	-2.92532900	0.42030100	1.59603100
C	-1.94895300	1.42774400	4.00391200
H	0.00288200	0.94980400	3.22887200
C	-3.80948700	0.88747000	2.56739900
H	-3.31713900	0.02267700	0.66496600
C	-3.32443300	1.39933600	3.76803000
H	-1.56393300	1.80811500	4.94435800
H	-4.87775700	0.85143500	2.37990300
H	-4.01308200	1.76445000	4.52270700
C	1.31889800	-3.45379300	1.25503500
C	1.01681300	-2.13036400	0.93584100
C	-0.25525800	-1.61302900	1.18991600
C	-1.20950800	-2.42583600	1.81395900
C	-0.90605200	-3.74373400	2.13403400
C	0.35729200	-4.26568100	1.84762100
H	2.31561400	-3.83503000	1.05620800
H	1.79600300	-1.49959900	0.51744000
H	-2.19598300	-2.03595000	2.04248100
H	-1.65874800	-4.36622800	2.60707100
H	0.59194700	-5.29441700	2.10212200
C	-0.55021600	-0.15793300	0.83909700
H	0.40700400	0.39557500	1.00123800
C	-4.79344800	3.06909100	-1.32871200
C	-4.27869600	2.28511600	-2.35869100

C	-2.97268700	1.80611300	-2.27991200
C	-2.17146800	2.09535100	-1.17024500
C	-2.69060800	2.89157500	-0.14744500
C	-3.99167500	3.37906000	-0.23137900
H	-5.80810200	3.44867200	-1.38783600
H	-4.88180400	2.05924800	-3.23168500
H	-2.57498100	1.23504000	-3.11547000
H	-2.09243800	3.14153700	0.72087300
H	-4.37743400	4.00261300	0.56847500
C	-0.70362000	1.57123300	-1.15301800
H	-0.46052300	1.43466700	-2.21111800
C	-0.74681200	0.16713000	-0.60027800
C	4.29957800	-1.72931100	2.15513200
C	4.41978000	-0.77784400	1.14639600
C	3.68834400	0.41952700	1.21060800
C	2.82374300	0.62456700	2.30015500
C	2.70474300	-0.33710900	3.29993400
C	3.44265000	-1.51792400	3.23540300
H	4.88417800	-2.64244100	2.09707000
H	5.09697700	-0.95972300	0.31551000
H	2.28146700	1.56542700	2.37152600
H	2.04412900	-0.16047300	4.14397300
H	3.35067900	-2.26439500	4.01681000
C	3.79749800	1.46411300	0.20086500
H	3.20043200	2.36529400	0.20992700
N	4.72734400	1.41085700	-0.70838400
N	5.54184800	1.32794600	-1.49600200

TS10

C	-1.16475500	-4.53960500	-1.15240400
C	-1.32396000	-3.23909500	-0.67863100
C	-1.23277500	-2.14269100	-1.54519400

C	-1.02280700	-2.39327800	-2.90498200
C	-0.86494500	-3.69284100	-3.38528200
C	-0.92323500	-4.77112000	-2.50686600
H	-1.23795500	-5.37382900	-0.46190700
H	-1.52279100	-3.08347900	0.37765300
H	-0.98471600	-1.56410100	-3.60628100
H	-0.69740000	-3.85784500	-4.44448400
H	-0.79712300	-5.78417400	-2.87367300
C	-1.39110200	-0.68895200	-1.09275100
H	-1.29936400	-0.14046600	-2.03285500
C	-3.29555900	0.91732500	-1.07147500
C	-2.81054600	-0.32812100	-0.65306700
C	-3.68622500	-1.19142100	0.01194700
C	-4.98679000	-0.79330600	0.30235700
C	-5.44111200	0.47206100	-0.06849400
C	-4.59275100	1.32616600	-0.76742500
H	-2.65984500	1.56498300	-1.67319700
H	-3.36702100	-2.17757000	0.32474300
H	-5.64684500	-1.47645800	0.82741600
H	-6.45579900	0.77838500	0.16395400
H	-4.94384000	2.29792600	-1.09960100
C	2.32148000	0.63103400	-0.94253000
C	2.80376500	1.25222800	0.21214300
C	3.24644000	0.25680100	-1.92637600
C	4.16823500	1.47748300	0.38333800
H	2.12742300	1.59194000	0.98324800
C	4.61100300	0.48136500	-1.75964500
H	2.89165000	-0.19341700	-2.85158700
C	5.07792500	1.09468300	-0.59935700
H	4.51620700	1.95865700	1.29211000
H	5.30360100	0.18822700	-2.54222400
H	6.13894600	1.27929700	-0.46585500

C	-0.28619500	1.89529400	1.39825500
C	0.46281000	2.34228600	2.49475700
C	-1.08570100	2.81154000	0.71950900
C	0.43919300	3.67801200	2.88096500
H	1.06627400	1.63119900	3.05730400
C	-1.11512000	4.15214100	1.10439500
H	-1.69710300	2.48973900	-0.11308900
C	-0.35000400	4.59147800	2.18033100
H	1.02933500	4.00375000	3.73125300
H	-1.74028300	4.84981200	0.55605000
H	-0.37308100	5.63435400	2.47877000
C	-2.30512300	-2.15466300	3.25896600
C	-1.31776100	-1.58290100	2.45570600
C	-1.41023400	-0.26120100	2.00449900
C	-2.51464100	0.48748700	2.43344300
C	-3.49902400	-0.07434200	3.23960400
C	-3.40753000	-1.40247900	3.65043100
H	-2.19538800	-3.18219600	3.59185200
H	-0.45934400	-2.19650300	2.21586600
H	-2.62564000	1.52126900	2.12871700
H	-4.34592500	0.53327000	3.54198600
H	-4.17479700	-1.83903900	4.28126600
C	-0.30011800	0.37784400	1.14355100
H	0.66111100	0.05626000	1.56929600
C	-0.36765600	3.96795900	-3.57732900
C	-0.64053100	2.69069600	-4.06022200
C	-0.25942100	1.57290700	-3.31970500
C	0.37838700	1.70873500	-2.08215500
C	0.68780100	2.99464500	-1.63073000
C	0.30931400	4.11364000	-2.36745100
H	-0.65782000	4.84212700	-4.15075000
H	-1.13247400	2.55852200	-5.01820100

H	-0.44026500	0.58553600	-3.73756800
H	1.23242800	3.13276000	-0.70279000
H	0.55485600	5.10293800	-1.99463900
C	0.85433300	0.44603200	-1.31057900
H	0.84721900	-0.33467900	-2.08709100
C	-0.19363400	-0.08118800	-0.30962700
C	4.87690400	-1.63235600	1.57107300
C	3.80804200	-1.86748300	0.71769300
C	2.48953300	-1.77840800	1.18803200
C	2.27241700	-1.46738400	2.53987800
C	3.35141000	-1.21670600	3.38592700
C	4.65564400	-1.29467800	2.90688300
H	5.88920700	-1.70662400	1.18728100
H	4.01021600	-2.10963600	-0.32162000
H	1.26964500	-1.43646100	2.95260700
H	3.16461700	-0.97922900	4.42822500
H	5.49394500	-1.10893200	3.56977800
C	1.32714500	-2.02696700	0.30608700
H	0.46691900	-2.55645200	0.68149800
N	1.62867000	-2.46147900	-0.93101900
N	1.89647800	-2.71163400	-1.98732800

IN14

C	-0.65976500	4.25899600	2.45356200
C	-1.04784300	3.18797500	1.65253500
C	-0.83787900	1.85735900	2.05510100
C	-0.30327700	1.64972700	3.33438600
C	0.09369100	2.71510100	4.13835100
C	-0.06195200	4.02791100	3.69127100
H	-0.82826000	5.27444200	2.10973000
H	-1.52331400	3.40535400	0.70369400
H	-0.17308300	0.64219700	3.71277500

H	0.51855600	2.51972700	5.11773500
H	0.25096100	4.85944300	4.31393000
C	-1.16113300	0.66540300	1.14735500
H	-1.16439500	-0.19965000	1.80671100
C	-3.51726700	-0.11370700	1.37974200
C	-2.63655400	0.75990100	0.72897300
C	-3.20151800	1.73292000	-0.10389400
C	-4.57330800	1.79053300	-0.32333000
C	-5.42839700	0.88441800	0.30209800
C	-4.89355700	-0.06599100	1.16498800
H	-3.12322900	-0.84699600	2.08147400
H	-2.58053400	2.44077300	-0.63579600
H	-4.97101600	2.54458400	-0.99528800
H	-6.49813300	0.92818500	0.12651600
H	-5.54210700	-0.76609700	1.68151500
C	2.52880700	-0.63378900	0.58055100
C	2.92576100	-1.15717000	-0.65664200
C	3.52637000	-0.20793900	1.46331400
C	4.26995600	-1.21967700	-1.00907000
H	2.18366800	-1.52909200	-1.35503100
C	4.87651900	-0.26757300	1.11755000
H	3.24504800	0.15809400	2.45022200
C	5.25151900	-0.76850600	-0.12598700
H	4.55151300	-1.62305000	-1.97673000
H	5.63023800	0.06463800	1.82442800
H	6.29981700	-0.82022400	-0.40172100
C	-0.69529300	-1.96266600	-1.07948700
C	-0.08105300	-2.80898300	-2.00528700
C	-1.61975600	-2.51743300	-0.19028200
C	-0.35192100	-4.17752000	-2.02540400
H	0.60737700	-2.39412900	-2.73988000
C	-1.89807200	-3.87960600	-0.20488600

H	-2.13948200	-1.88317200	0.51452100
C	-1.25967900	-4.71794800	-1.11972500
H	0.13963700	-4.81398100	-2.75422700
H	-2.61271700	-4.28730700	0.50316400
H	-1.47550800	-5.78144000	-1.12975300
C	-2.37091800	2.06010600	-3.41487900
C	-1.39490300	1.50343000	-2.58933200
C	-1.51494000	0.20905900	-2.05455300
C	-2.66893000	-0.50383900	-2.41365400
C	-3.65303600	0.04989200	-3.22856100
C	-3.51351300	1.33690200	-3.73836900
H	-2.22743800	3.06387900	-3.80301900
H	-0.54297000	2.13831200	-2.38081800
H	-2.82171200	-1.51158400	-2.05057900
H	-4.53336200	-0.53897300	-3.46536200
H	-4.27557300	1.76528300	-4.38101400
C	-0.41089700	-0.46861900	-1.19397400
H	0.48802300	-0.44393600	-1.81067900
C	0.07652200	-4.50362500	2.72992200
C	-0.40205800	-3.33934700	3.31954000
C	-0.08118200	-2.09585500	2.77500900
C	0.69284200	-1.97562200	1.61360700
C	1.20135700	-3.15999200	1.06336700
C	0.89316600	-4.40252000	1.60523600
H	-0.16341200	-5.47391500	3.15211000
H	-1.00860400	-3.38782800	4.21838900
H	-0.44318500	-1.21705000	3.29572100
H	1.84121900	-3.13027800	0.19122600
H	1.29541700	-5.29679500	1.13986600
C	1.08159800	-0.58434300	1.06231800
H	1.11975700	0.03665500	1.96638800
C	0.04857000	0.25436800	0.14392600

C	4.32380600	2.11458700	-1.99264900
C	3.30931700	2.04154900	-1.04364300
C	2.01916600	1.60916200	-1.38005300
C	1.78741300	1.26966900	-2.72309700
C	2.80897200	1.33761600	-3.66795100
C	4.08478700	1.75964500	-3.31447300
H	5.30980200	2.43959200	-1.67779500
H	3.59486400	2.28680300	-0.02955100
H	0.82315800	0.94310300	-3.07899300
H	2.58519400	1.06077000	-4.69290200
H	4.87603100	1.81288600	-4.05432100
C	0.85577400	1.56929900	-0.33568100
H	0.10613700	2.32463100	-0.59714200
N	1.45855500	2.21974300	0.87015300
N	2.06683800	2.71691400	1.64034100

TS11

C	-0.76241300	-4.41179100	-2.16689400
C	-1.05202800	-3.28832600	-1.39831100
C	-0.87322700	-1.98987500	-1.90444200
C	-0.46114900	-1.86948200	-3.23742300
C	-0.16929300	-2.98925700	-4.01361400
C	-0.30109000	-4.26770700	-3.47502800
H	-0.90564300	-5.40147200	-1.74524800
H	-1.43610900	-3.44126400	-0.39570200
H	-0.35565900	-0.88797600	-3.68631900
H	0.15526400	-2.86067300	-5.04117600
H	-0.07272900	-5.14201600	-4.07533900
C	-1.15518900	-0.73343400	-1.07224000
H	-1.15149100	0.08383400	-1.78906000
C	-3.50448400	0.05521300	-1.35794000
C	-2.63224000	-0.77777300	-0.64512400

C	-3.20796300	-1.68876500	0.24785100
C	-4.58009600	-1.71458100	0.47223200
C	-5.42562500	-0.84353500	-0.21283100
C	-4.88115600	0.03677700	-1.14177700
H	-3.10305800	0.73165000	-2.11099300
H	-2.59569100	-2.37613000	0.81446800
H	-4.98613400	-2.41973400	1.19074300
H	-6.49568300	-0.86336000	-0.03493800
H	-5.52255300	0.70381700	-1.70856500
C	2.54832900	0.61058500	-0.62110800
C	2.95207400	1.20474000	0.58126000
C	3.54071700	0.14212100	-1.48736600
C	4.29796300	1.28702300	0.92286000
H	2.21404300	1.61246600	1.26397200
C	4.89254700	0.22384700	-1.15369000
H	3.25407300	-0.27865800	-2.44967000
C	5.27462400	0.78903000	0.05976900
H	4.58447100	1.74305800	1.86534200
H	5.64241200	-0.14424000	-1.84668800
H	6.32443100	0.85557200	0.32633300
C	-0.68991400	2.04012000	0.97432900
C	-0.06537400	2.95219300	1.82900800
C	-1.63973400	2.52222300	0.07065600
C	-0.35671600	4.31488800	1.76781500
H	0.64514800	2.59567500	2.57293000
C	-1.93439100	3.87948700	0.00237400
H	-2.16558700	1.83681700	-0.57826800
C	-1.29015900	4.78328100	0.84809900
H	0.13998300	5.00330400	2.44397200
H	-2.66849100	4.23121600	-0.71567000
H	-1.52081200	5.84230800	0.79377900
C	-2.27564400	-1.89094900	3.53656600

C	-1.33740800	-1.35984800	2.65424300
C	-1.46995700	-0.07545100	2.10542200
C	-2.59711500	0.66000000	2.49737600
C	-3.54845400	0.13015600	3.36652400
C	-3.39532400	-1.14723000	3.89596800
H	-2.12713200	-2.88974700	3.93510500
H	-0.50427700	-2.00788100	2.40315600
H	-2.75264100	1.66232200	2.11948700
H	-4.41360900	0.72984200	3.63011200
H	-4.13227600	-1.55588100	4.57940400
C	-0.39005800	0.56386700	1.19876600
H	0.51568700	0.59565900	1.80433300
C	0.03986200	4.32819300	-2.96099700
C	-0.42309900	3.12622100	-3.48419000
C	-0.08525800	1.92038200	-2.87045500
C	0.69083500	1.87700100	-1.70493900
C	1.18774300	3.09692000	-1.22511500
C	0.86134800	4.30233800	-1.83530100
H	-0.21383400	5.26981500	-3.43673300
H	-1.02920000	3.11589900	-4.38453000
H	-0.42754300	1.00628400	-3.34082700
H	1.83501200	3.12394000	-0.35847500
H	1.25436500	5.22688400	-1.42442500
C	1.09891800	0.52511900	-1.08502200
H	1.11775300	-0.15918200	-1.94321500
C	0.06350900	-0.27385000	-0.10817700
C	4.30655700	-2.03355200	2.04372300
C	3.27569400	-1.97911500	1.11306300
C	2.00483800	-1.48589300	1.44927200
C	1.81742900	-1.07126100	2.78333100
C	2.85540300	-1.13154900	3.70978200
C	4.10945400	-1.60885400	3.35202200

H	5.27585500	-2.39997700	1.72241600
H	3.52546400	-2.27037800	0.10527800
H	0.86773700	-0.70790100	3.14468100
H	2.65994700	-0.80056800	4.72441300
H	4.91563000	-1.64915600	4.07629600
C	0.82522200	-1.46911400	0.48237600
H	0.20247500	-2.36146700	0.56958600
N	1.65212100	-2.31948100	-1.04754100
N	2.15064100	-2.96279100	-1.78619900

IN15

C	-2.75968000	2.21117700	-2.96369300
C	-2.37601700	1.22403000	-2.05785600
C	-2.26651600	1.50137400	-0.68918200
C	-2.61854600	2.78558900	-0.25481800
C	-3.00125600	3.77739200	-1.15539000
C	-3.05845800	3.49789200	-2.51902000
H	-2.82829800	1.96967500	-4.01982300
H	-2.16224100	0.23369200	-2.43668200
H	-2.58292000	3.01749700	0.80597200
H	-3.25762300	4.76616400	-0.78855600
H	-3.35168100	4.26753500	-3.22545800
C	-1.84578300	0.49375300	0.37203400
H	-1.84299600	1.08122300	1.29067700
C	-3.28313000	-0.78130300	1.99198400
C	-2.94813000	-0.53658600	0.65078600
C	-3.71248700	-1.16924400	-0.33473400
C	-4.73905900	-2.04548600	0.00840500
C	-5.03142800	-2.30874300	1.34529300
C	-4.30144700	-1.66632400	2.34260400
H	-2.75400400	-0.24360600	2.77948100
H	-3.51287500	-0.99290600	-1.38572700

H	-5.31262400	-2.52458600	-0.77877500
H	-5.83417800	-2.99035700	1.60678900
H	-4.53942100	-1.82996700	3.38892300
C	-0.81868000	-1.83239500	-1.56984600
C	-1.13523500	-1.86994700	-2.93432700
C	-1.24302500	-2.89839400	-0.77561200
C	-1.89376500	-2.90517600	-3.47295900
H	-0.77160600	-1.07892000	-3.58719100
C	-1.99424000	-3.94462100	-1.30689600
H	-1.01331600	-2.93215100	0.28601300
C	-2.33353400	-3.94652200	-2.65652700
H	-2.13279600	-2.90179600	-4.53146200
H	-2.32584500	-4.74883100	-0.65811100
H	-2.92618000	-4.75531500	-3.07082700
C	1.36824400	2.00393100	-0.43534500
C	2.75361600	2.17035200	-0.31184500
C	0.72911100	2.61069200	-1.52179700
C	3.49190700	2.87006000	-1.26102100
H	3.26518300	1.73975400	0.54663700
C	1.46559500	3.30402300	-2.48304200
H	-0.34545000	2.55574200	-1.63342500
C	2.84876800	3.42766500	-2.36517000
H	4.56588100	2.97595600	-1.14182800
H	0.94648700	3.75495300	-3.32309000
H	3.41681200	3.96696800	-3.11610200
C	-0.67067200	2.47330500	4.04836800
C	-0.18841600	1.65559100	3.03206700
C	0.12155900	2.16869700	1.76428200
C	-0.02915600	3.54094100	1.55929300
C	-0.50715600	4.36839500	2.57824000
C	-0.83776400	3.84043400	3.82121700
H	-0.90587400	2.04776100	5.01872200

H	-0.05040300	0.59632700	3.24345700
H	0.22156200	3.97719100	0.59862600
H	-0.61808400	5.43176200	2.39138100
H	-1.21156000	4.48406900	4.61049800
C	0.68695500	1.23271500	0.68352800
H	1.51573900	0.75036200	1.19420600
C	4.10912000	-2.10297000	-2.00298400
C	3.67996000	-0.84892100	-2.43237000
C	2.40604000	-0.39063100	-2.10453200
C	1.53324900	-1.16844800	-1.33034300
C	1.96645100	-2.44965000	-0.94597700
C	3.24232700	-2.90636200	-1.26426700
H	5.09947800	-2.46232900	-2.26462500
H	4.33061300	-0.22361900	-3.03616200
H	2.08310000	0.57507500	-2.47982400
H	1.29226900	-3.11718100	-0.41799600
H	3.54803600	-3.90088300	-0.95492200
C	0.09689100	-0.70288700	-1.10592100
H	-0.05533500	0.14802900	-1.77717800
C	-0.31218300	0.00016600	0.32675200
C	3.33342800	-2.07198000	2.42405400
C	2.36218700	-1.38613500	1.71826700
C	0.97997500	-1.63637600	1.97847700
C	0.64712900	-2.61556800	2.96594100
C	1.62446800	-3.29187100	3.66517300
C	2.97048300	-3.01332300	3.39352600
H	4.37931300	-1.87819700	2.21621000
H	2.67352300	-0.68232200	0.95639900
H	-0.40258500	-2.81998000	3.16364300
H	1.35674800	-4.02827900	4.41417800
H	3.74605300	-3.53859000	3.94316500
C	-0.13722000	-1.03002200	1.36337600

H	-1.07526000	-1.43111900	1.74579500
N	5.99750000	0.46604000	-0.21447900
N	5.30614200	-0.05011600	0.46559600

IN16

C	3.91162300	-2.88129300	-1.35176100
C	2.87393300	-2.25305400	-0.66560200
C	1.63532500	-2.03133800	-1.27626600
C	1.44749900	-2.53664900	-2.56992600
C	2.48047400	-3.16269300	-3.26269900
C	3.72785200	-3.32059100	-2.66044900
H	4.86740900	-3.02504500	-0.85792900
H	3.04860200	-1.95013800	0.35765300
H	0.47022600	-2.44435500	-3.03906100
H	2.30782100	-3.53169300	-4.26857600
H	4.53915500	-3.80149700	-3.19683800
C	0.42903100	-1.38732500	-0.60490700
H	-0.24818900	-1.12917300	-1.42856600
C	-1.72681900	-2.53535800	0.15032400
C	-0.32639600	-2.49755900	0.16178400
C	0.33533800	-3.56052500	0.79048900
C	-0.37035300	-4.56892400	1.43763700
C	-1.76430300	-4.55503800	1.46739700
C	-2.44276000	-3.53431100	0.80978000
H	-2.28391400	-1.77791300	-0.39272300
H	1.41761100	-3.60817200	0.78870000
H	0.17717700	-5.36959900	1.92471600
H	-2.31192700	-5.34180500	1.97583100
H	-3.52875200	-3.51886200	0.78768300
C	1.34733300	-1.09459700	2.40335000
C	2.44525300	-1.82059900	2.87317700
C	0.09836500	-1.34640200	2.98048900

C	2.29625700	-2.79789500	3.85638000
H	3.43704800	-1.61114100	2.47813500
C	-0.05828000	-2.31354600	3.96721300
H	-0.78547400	-0.80476300	2.65228300
C	1.04154100	-3.05120600	4.40366000
H	3.16392300	-3.35310400	4.19785400
H	-1.04302400	-2.50150600	4.38289700
H	0.92044600	-3.81258000	5.16714000
C	2.58594100	1.37635800	-1.33936700
C	3.16705800	2.62614600	-1.06846600
C	3.31316000	0.49681900	-2.14932800
C	4.43858500	2.96311600	-1.52052000
H	2.61192700	3.35661400	-0.48385900
C	4.59426400	0.82300200	-2.59449000
H	2.89877700	-0.44827700	-2.46604200
C	5.16971100	2.04930300	-2.27638600
H	4.85173000	3.93911700	-1.28626100
H	5.13309400	0.10862300	-3.20900400
H	6.16476200	2.29874400	-2.62987000
C	-1.23830900	3.02762100	-3.29368600
C	-0.50495000	2.67871100	-2.15909800
C	0.22819200	1.48593600	-2.10152100
C	0.22079400	0.66074200	-3.23252800
C	-0.51847000	0.99801100	-4.36501500
C	-1.25345600	2.18293500	-4.40187000
H	-1.78453600	3.96545700	-3.31421100
H	-0.48270900	3.36686300	-1.31867600
H	0.82730700	-0.23762300	-3.25224900
H	-0.50224500	0.34040600	-5.22895300
H	-1.81762800	2.45090400	-5.28897400
C	1.12326200	1.22167800	-0.88883700
H	0.99330500	2.09542300	-0.25817000

C	2.39752500	3.61638400	3.70265200
C	3.27347800	3.19709700	2.70344500
C	2.99295500	2.04931300	1.96387400
C	1.82175700	1.31316800	2.17886900
C	0.97697200	1.72082700	3.22416800
C	1.25630700	2.86204100	3.97089700
H	2.61933900	4.50187400	4.28948900
H	4.18947000	3.74712000	2.51142500
H	3.70454000	1.71251800	1.21755500
H	0.10136000	1.13371900	3.48166300
H	0.58664300	3.15220300	4.77454600
C	1.60636200	0.01851600	1.39719900
H	2.56345200	-0.17713300	0.90510700
C	0.61762300	0.05090200	0.10157800
C	-1.53963300	3.91766300	1.81795400
C	-0.78952000	2.90841200	1.25311600
C	-1.35730000	1.60807100	1.05949000
C	-2.71189100	1.40553500	1.47911000
C	-3.44650000	2.42077900	2.06368300
C	-2.86027600	3.67669000	2.22763000
H	-1.09646100	4.89609100	1.96655700
H	0.23798600	3.11934300	0.99072100
H	-3.16410700	0.42519100	1.34115800
H	-4.46791900	2.23967400	2.37852500
H	-3.43167600	4.48081500	2.68216400
C	-0.74076500	0.44149700	0.56489000
H	-1.44067200	-0.38654600	0.57323400
C	-5.94708800	-1.13728900	-0.11624200
C	-4.81306200	-1.17773900	-0.92030200
C	-4.19238900	0.01457100	-1.33196200
C	-4.74884300	1.24055700	-0.93134900
C	-5.89106400	1.27129200	-0.13641000

C	-6.49110400	0.08531300	0.28454700
H	-6.41790900	-2.06639800	0.18935600
H	-4.41708100	-2.13892700	-1.24113800
H	-4.28488900	2.16721700	-1.26122700
H	-6.31950600	2.22832500	0.14674900
H	-7.38377300	0.11003300	0.90018500
C	-2.98588900	0.04375400	-2.14575600
H	-2.50009900	0.96158700	-2.45038600
N	-2.54854300	-1.03405500	-2.74012900
N	-2.15527400	-1.97945900	-3.22684900

TS12

C	-4.78519600	0.52852800	-1.63407800
C	-3.50415300	0.70776800	-1.11596000
C	-2.37185900	0.32383900	-1.84047200
C	-2.56956200	-0.17800800	-3.13397700
C	-3.84583800	-0.36609300	-3.65752300
C	-4.96457100	-0.02651800	-2.89831900
H	-5.64553600	0.81986800	-1.04005000
H	-3.40893600	1.15664900	-0.13657000
H	-1.70431800	-0.41920100	-3.74947800
H	-3.96474700	-0.76949000	-4.65794800
H	-5.96303300	-0.17444500	-3.29626800
C	-0.92788900	0.51856000	-1.39141700
H	-0.35085800	-0.12651200	-2.05807400
C	0.77434600	2.21403400	-2.30864600
C	-0.50688400	1.95277100	-1.80516000
C	-1.41285600	3.02237600	-1.80622200
C	-1.03359100	4.29626400	-2.21417100
C	0.26359900	4.54408600	-2.66044400
C	1.16662100	3.48909000	-2.71691000
H	1.49927000	1.41345100	-2.39455700

H	-2.43301900	2.87241800	-1.47644400
H	-1.76193600	5.10029400	-2.18153300
H	0.55690900	5.53883100	-2.97920500
H	2.17193900	3.64620300	-3.09653100
C	-1.08320600	2.36079900	1.16843100
C	-2.27842100	3.05636400	1.37597500
C	0.09022000	3.10905100	1.02293900
C	-2.31535900	4.44965800	1.38443600
H	-3.19827600	2.50243700	1.55066600
C	0.06171600	4.49967100	1.02711200
H	1.04864000	2.61812900	0.89500800
C	-1.14418700	5.17826200	1.19878300
H	-3.25926900	4.96143000	1.54275900
H	0.98488900	5.05439300	0.89154300
H	-1.16644300	6.26309700	1.19928800
C	-2.28078500	-2.06505400	0.65538400
C	-2.49816500	-2.62889900	1.92132200
C	-3.29220000	-2.23122100	-0.29923300
C	-3.69971200	-3.24765400	2.25693200
H	-1.70914500	-2.58396400	2.66731000
C	-4.50012100	-2.84179300	0.03130300
H	-3.15514400	-1.90721000	-1.32188500
C	-4.71868000	-3.34065100	1.31315600
H	-3.82993900	-3.66446800	3.25073000
H	-5.26643100	-2.93857500	-0.73150000
H	-5.65960200	-3.81936700	1.56402400
C	1.20966300	-4.60105100	-0.71557500
C	0.60438300	-3.60704200	0.05302000
C	-0.21404900	-2.62810200	-0.52775300
C	-0.48198300	-2.73798400	-1.89760200
C	0.12223800	-3.72733300	-2.67265000
C	0.99066900	-4.65053700	-2.09111200

H	1.84296100	-5.34093500	-0.23632400
H	0.76273200	-3.60996900	1.12728700
H	-1.18261400	-2.06031700	-2.37059700
H	-0.09834100	-3.78334700	-3.73468800
H	1.46202200	-5.41806000	-2.69568300
C	-0.84848700	-1.56854100	0.36821900
H	-0.37384300	-1.68852500	1.34154100
C	-0.51098200	0.03383500	5.49918900
C	-1.68875900	-0.33612500	4.85576900
C	-1.84179400	-0.09806600	3.49117100
C	-0.82328100	0.48694800	2.72863500
C	0.32609600	0.91594200	3.40916800
C	0.48853400	0.67701300	4.77063700
H	-0.38690400	-0.14317100	6.56261900
H	-2.49959600	-0.79303500	5.41453500
H	-2.77997800	-0.36056600	3.01273700
H	1.09685600	1.47544700	2.89125000
H	1.39206500	1.01798800	5.26674200
C	-1.12999600	0.83923600	1.26765500
H	-2.17522000	0.54570600	1.14294500
C	-0.47572100	-0.03719400	0.05304600
C	3.09691000	-1.24407500	2.95419900
C	2.06212700	-1.14440100	2.03684400
C	2.03490500	-0.09446000	1.09089000
C	3.10996700	0.83312900	1.10782200
C	4.12488600	0.75166400	2.04776800
C	4.12085100	-0.29345900	2.97279200
H	3.09205100	-2.05091200	3.67917000
H	1.26769500	-1.87296000	2.08094300
H	3.11953800	1.64146300	0.38067000
H	4.92190100	1.48733100	2.05046800
H	4.91579700	-0.36997600	3.70821200

C	1.02595700	0.17750700	0.10156500
H	1.30773200	1.04416000	-0.48233400
C	5.62182400	0.69402700	-1.67500900
C	4.29729500	0.38306900	-1.96511100
C	3.70041100	-0.74653600	-1.38711200
C	4.44676900	-1.55756900	-0.52237800
C	5.76848900	-1.23624700	-0.23031700
C	6.35829900	-0.10996500	-0.80268900
H	6.08126900	1.56459700	-2.13142600
H	3.73267500	1.02131800	-2.64184900
H	3.98563900	-2.44020200	-0.08609400
H	6.34012500	-1.87062500	0.43946700
H	7.39153800	0.13587300	-0.58121000
C	2.28887300	-1.10143100	-1.59667100
H	1.92853400	-2.04302200	-1.19834100
N	1.82139800	-0.91170800	-2.84642700
N	1.41091500	-0.69073700	-3.86317900

IN17

C	3.01944900	-2.59705000	3.00284800
C	1.97631800	-1.88164300	2.41932700
C	1.39121800	-2.30600800	1.22442200
C	1.80211300	-3.53837900	0.70012600
C	2.83626100	-4.26650200	1.28420800
C	3.47030000	-3.78330400	2.42662400
H	3.46698300	-2.23209800	3.92226000
H	1.60558100	-0.99700100	2.91855600
H	1.30672200	-3.94558600	-0.17711400
H	3.14194900	-5.21177500	0.84734300
H	4.28345200	-4.34024100	2.88006800
C	0.21132700	-1.57895500	0.56669900
H	0.04100300	-2.16699300	-0.33738700

C	-1.65966700	-3.14286200	1.18707000
C	-0.98029100	-1.94915900	1.47088600
C	-1.33073000	-1.27653900	2.64680000
C	-2.37075300	-1.73198900	3.45652100
C	-3.07089800	-2.89203500	3.12776600
C	-2.69547800	-3.60981700	1.99395300
H	-1.34590500	-3.74891000	0.33952300
H	-0.78562200	-0.39341400	2.95131100
H	-2.61937900	-1.18020700	4.35796800
H	-3.87645900	-3.24768300	3.76172200
H	-3.19748900	-4.53860000	1.74216800
C	0.88221400	1.30390200	2.32033700
C	1.92032300	1.28978500	3.26568700
C	-0.33461900	1.85681500	2.72337400
C	1.73519200	1.74925800	4.56512500
H	2.89930900	0.91812000	2.96900800
C	-0.53304600	2.31809600	4.02534600
H	-1.15143900	1.94098000	2.02364100
C	0.49766900	2.25876700	4.95747200
H	2.56108100	1.71751500	5.26853200
H	-1.49685000	2.73434500	4.30257800
H	0.34673800	2.61898400	5.96960900
C	2.64243700	-0.15638900	-1.56564500
C	3.19173700	0.61593400	-2.59909900
C	3.51173500	-0.94572000	-0.81208100
C	4.55702700	0.62842700	-2.85396200
H	2.53709200	1.24482800	-3.19978100
C	4.88678600	-0.93434700	-1.06014300
H	3.14790200	-1.56244800	-0.00349500
C	5.41785300	-0.14637700	-2.07574300
H	4.95026400	1.25157800	-3.65080300
H	5.53808500	-1.54799000	-0.44532300

H	6.48666100	-0.13610700	-2.26233100
C	-0.61674300	-1.50754100	-4.59245300
C	-0.16988000	-0.62528200	-3.60592000
C	0.60051100	-1.06615400	-2.52163000
C	1.01022800	-2.40721600	-2.52917600
C	0.56196400	-3.29793800	-3.49871600
C	-0.27657600	-2.85631300	-4.52593100
H	-1.21190600	-1.13417900	-5.42004300
H	-0.41657300	0.42958800	-3.70207400
H	1.72186300	-2.74230800	-1.78265200
H	0.88835000	-4.33299700	-3.47113900
H	-0.62208900	-3.54739200	-5.28762900
C	1.10921900	-0.09238800	-1.45231800
H	0.89137700	0.90832800	-1.82367800
C	2.97846600	4.66390600	-0.54088500
C	3.75591900	3.51741100	-0.41167900
C	3.17701700	2.32022400	0.00493100
C	1.80704500	2.22344700	0.28528600
C	1.05954000	3.40740300	0.22436300
C	1.62759000	4.60416000	-0.20431000
H	3.42406400	5.59879000	-0.86525700
H	4.81959200	3.54634000	-0.62649800
H	3.81535500	1.44917500	0.11662600
H	0.02691600	3.42402000	0.55095800
H	1.01223600	5.49786000	-0.24654800
C	1.25825900	0.90321400	0.88660800
H	2.15227500	0.28550400	1.00829200
C	0.36128700	-0.07719400	-0.02945500
C	-1.51192200	3.80727500	-2.08692800
C	-1.07456800	2.52363600	-1.76693600
C	-1.50776800	1.87916900	-0.60153200
C	-2.46597000	2.53917900	0.18149300

C	-2.90300200	3.82313600	-0.12760400
C	-2.41515300	4.47037200	-1.26127600
H	-1.12917600	4.29165300	-2.97923700
H	-0.35712700	2.06407200	-2.43345300
H	-2.89617500	2.03370500	1.04280800
H	-3.63278900	4.30984700	0.51093500
H	-2.74779800	5.47359500	-1.50633700
C	-1.14821400	0.42859500	-0.21264300
H	-1.59944000	0.28032700	0.76833700
C	-5.46227200	-0.43497100	0.63819700
C	-4.11200200	-0.60479500	0.34770100
C	-3.60823200	-0.13303200	-0.86726700
C	-4.44734600	0.49589400	-1.78920500
C	-5.79411200	0.67524900	-1.48508700
C	-6.30104300	0.20808000	-0.27312900
H	-5.85618200	-0.80433600	1.57932800
H	-3.46314500	-1.09984300	1.06467200
H	-4.04378800	0.87362300	-2.72553000
H	-6.44429700	1.17923800	-2.19192000
H	-7.35170700	0.34396600	-0.03875300
C	-2.14074500	-0.25675200	-1.20934200
H	-1.95181400	0.08578500	-2.22669600
N	-1.99385800	-1.77895100	-1.50698700
N	-2.12379200	-2.80387000	-1.88315300

TS13

C	3.00711300	-2.58359400	3.02856800
C	1.97100400	-1.86013000	2.44248200
C	1.36179800	-2.29891700	1.26484500
C	1.74107100	-3.55060600	0.76306600
C	2.76822100	-4.28638500	1.34988700
C	3.42682600	-3.79098100	2.47298400

H	3.47402100	-2.20776100	3.93383900
H	1.62497300	-0.95707700	2.92615300
H	1.22551400	-3.96676800	-0.09826600
H	3.04927000	-5.24705900	0.93042600
H	4.23483200	-4.35394000	2.92818100
C	0.18964100	-1.56344800	0.60361200
H	0.00036100	-2.16543200	-0.28749900
C	-1.72279500	-3.06368700	1.26001300
C	-1.00175700	-1.89028400	1.52411700
C	-1.31639600	-1.19549400	2.69750700
C	-2.36310100	-1.60714300	3.52166900
C	-3.10571000	-2.74589100	3.21145300
C	-2.76541000	-3.48749400	2.08226000
H	-1.43692000	-3.68977800	0.41772800
H	-0.73851300	-0.32904700	2.98863000
H	-2.58321200	-1.03865000	4.42016200
H	-3.91631100	-3.06778000	3.85692300
H	-3.30014400	-4.40185400	1.84547200
C	0.89093900	1.34729600	2.29634700
C	1.92082500	1.33249800	3.25027900
C	-0.31914800	1.92946700	2.67896000
C	1.73276400	1.81746900	4.54030700
H	2.89623700	0.94058900	2.96834400
C	-0.52024200	2.41680200	3.97068000
H	-1.12861800	2.01547600	1.97111100
C	0.50123500	2.35486800	4.91308700
H	2.55213000	1.78375000	5.25119600
H	-1.47870100	2.85500000	4.23218000
H	0.34796200	2.73470900	5.91766900
C	2.64257500	-0.21149400	-1.55425500
C	3.20190100	0.53999100	-2.59760400
C	3.50204700	-0.99982400	-0.78831200

C	4.56762900	0.53393000	-2.85043800
H	2.55509500	1.16762300	-3.20804900
C	4.87753000	-1.00656500	-1.03394600
H	3.13013200	-1.60216700	0.02744200
C	5.41875700	-0.23882700	-2.05967600
H	4.96880800	1.14133000	-3.65544500
H	5.52114200	-1.61869300	-0.40958900
H	6.48791000	-0.24284800	-2.24454700
C	-0.61958000	-1.58653300	-4.56351700
C	-0.16914100	-0.69082000	-3.59110300
C	0.59220900	-1.11916700	-2.49531300
C	0.98822600	-2.46419000	-2.47581200
C	0.53579400	-3.36803900	-3.43106900
C	-0.29293300	-2.93709500	-4.47062000
H	-1.20689200	-1.22265000	-5.40088200
H	-0.40477400	0.36431600	-3.70848100
H	1.69267700	-2.79279100	-1.71969200
H	0.85152800	-4.40564900	-3.38273100
H	-0.64124400	-3.63861500	-5.22142900
C	1.10981200	-0.12994600	-1.44463900
H	0.90342700	0.86547700	-1.83610800
C	3.04251900	4.61610700	-0.63008200
C	3.80573200	3.46459600	-0.46505200
C	3.20938800	2.28475200	-0.02456700
C	1.83600400	2.21048500	0.24577000
C	1.10245900	3.40062700	0.14822400
C	1.68801200	4.57983800	-0.30510500
H	3.50161500	5.53754600	-0.97351700
H	4.87147600	3.47619700	-0.67101200
H	3.83613000	1.40883700	0.11291700
H	0.06725800	3.43730700	0.46489300
H	1.08334000	5.47904400	-0.37560200

C	1.26817700	0.91109100	0.87350200
H	2.15389300	0.28483200	1.01136100
C	0.36141700	-0.07694000	-0.02362100
C	-1.46393000	3.77967200	-2.17448700
C	-1.04471500	2.49800600	-1.82397100
C	-1.48887000	1.88680800	-0.64468100
C	-2.44105700	2.57703700	0.12006900
C	-2.85980300	3.85902100	-0.21979900
C	-2.36013100	4.47339700	-1.36683500
H	-1.07276900	4.23805700	-3.07678500
H	-0.33230200	2.01361300	-2.47789200
H	-2.87892500	2.09770800	0.99228900
H	-3.58444900	4.37026800	0.40523500
H	-2.67896300	5.47494800	-1.63590500
C	-1.14256800	0.44158700	-0.21684900
H	-1.59148300	0.32596600	0.76950000
C	-5.45707400	-0.35281300	0.66134700
C	-4.10232000	-0.51059700	0.38714600
C	-3.60515300	-0.11979700	-0.85978500
C	-4.45788500	0.41645300	-1.82779700
C	-5.80935900	0.58589500	-1.54048500
C	-6.30835100	0.19889700	-0.29733600
H	-5.84544800	-0.66024700	1.62671400
H	-3.44544000	-0.93557800	1.14069600
H	-4.06119200	0.72939500	-2.79059400
H	-6.46939900	1.01920800	-2.28403600
H	-7.36281000	0.32603400	-0.07528800
C	-2.14176300	-0.23918900	-1.19514200
H	-1.94588600	0.05974400	-2.22416300
N	-2.00169000	-1.81290700	-1.47815900
N	-2.17258600	-2.82806300	-1.86459000

IN18

C	-2.31281800	2.24144900	3.79195400
C	-1.54416500	1.42015400	2.97052100
C	-0.71885300	1.95717700	1.97909600
C	-0.59980200	3.35126600	1.91711100
C	-1.35889100	4.18026800	2.74028300
C	-2.24132500	3.62752300	3.66581000
H	-2.95723800	1.79487500	4.54289900
H	-1.57520800	0.35115700	3.12393300
H	0.10559800	3.79847400	1.22158400
H	-1.25370300	5.25764600	2.66198100
H	-2.84171400	4.26877700	4.30249700
C	0.18581300	1.10763300	1.08632500
H	0.62593100	1.81217500	0.37252300
C	2.52737000	1.52246100	1.88216700
C	1.37490200	0.73548600	1.99030500
C	1.33656300	-0.22874700	3.00626100
C	2.43685000	-0.43383900	3.83780900
C	3.58441200	0.34888100	3.70470500
C	3.61871700	1.34211800	2.73021900
H	2.56914600	2.29927600	1.12390600
H	0.44654300	-0.82258200	3.17263700
H	2.38102300	-1.19454200	4.61054800
H	4.43049900	0.19848000	4.36762500
H	4.49482400	1.97379000	2.62047100
C	-1.20213000	-1.93693300	1.81793200
C	-2.03413900	-1.93536000	2.94599300
C	-0.21692700	-2.93057700	1.75948800
C	-1.82857300	-2.80827200	4.01198600
H	-2.87204500	-1.24352400	2.98675600
C	0.00036900	-3.80562400	2.82144600
H	0.38519000	-3.05863500	0.87063300

C	-0.78972800	-3.73485600	3.96620800
H	-2.48734500	-2.76748500	4.87341900
H	0.78326800	-4.55420600	2.74613800
H	-0.61877800	-4.41224800	4.79609800
C	-2.58348900	1.10510000	-1.16548900
C	-3.32933100	0.81155000	-2.31430100
C	-3.21777300	1.80283300	-0.13573000
C	-4.66814400	1.16727400	-2.42386300
H	-2.85781900	0.26780000	-3.13126000
C	-4.56535600	2.15493500	-0.23571300
H	-2.68427900	2.07399700	0.76553800
C	-5.29870900	1.83581000	-1.37468900
H	-5.22152500	0.91188100	-3.32195900
H	-5.03481600	2.68576200	0.58683500
H	-6.34582400	2.11053100	-1.44882000
C	0.72612700	2.85553600	-3.84498700
C	0.21343700	1.75676200	-3.15350600
C	-0.39397500	1.89077500	-1.89845400
C	-0.52202400	3.19074300	-1.39348000
C	-0.00578900	4.29270400	-2.06851800
C	0.63001300	4.13114300	-3.29876500
H	1.17866300	2.71055500	-4.82109200
H	0.26656400	0.78939700	-3.64394600
H	-1.08764300	3.35050800	-0.48372100
H	-0.12908600	5.28470300	-1.64464900
H	1.02040500	4.99009900	-3.83455700
C	-1.09883900	0.70530900	-1.21018200
H	-1.10735300	-0.10853200	-1.93491600
C	-4.10023600	-3.37397000	-1.91003300
C	-4.61758200	-2.32886400	-1.14892200
C	-3.77404200	-1.55339600	-0.35801400
C	-2.39572100	-1.79452200	-0.30256900

C	-1.90370300	-2.88799300	-1.02439100
C	-2.73797200	-3.65515100	-1.83603800
H	-4.75266600	-3.97709100	-2.53297100
H	-5.67956600	-2.10595300	-1.17292100
H	-4.19711900	-0.72951300	0.21167200
H	-0.86854700	-3.19190200	-0.92739900
H	-2.32236000	-4.49360400	-2.38767700
C	-1.54389100	-0.96733300	0.68001600
H	-2.25208800	-0.25750300	1.11508500
C	-0.43285400	0.00577400	0.07015100
C	0.57424900	-2.96902300	-3.54921000
C	0.51055400	-1.84666100	-2.73217100
C	1.11229000	-1.84764400	-1.45617100
C	1.83755600	-3.00352200	-1.06433700
C	1.87655300	-4.12592600	-1.86437600
C	1.23955500	-4.11106100	-3.11426900
H	0.09314600	-2.95005500	-4.52067200
H	-0.02239000	-0.98181500	-3.09343600
H	2.37100700	-3.00264500	-0.11785400
H	2.41322600	-5.00857200	-1.53474800
H	1.28147000	-4.98923300	-3.75099000
C	0.88071100	-0.76506800	-0.29830300
H	1.24683400	-1.24758200	0.60314000
C	5.31299200	-0.94988700	0.48745600
C	3.93612200	-0.86291700	0.33580200
C	3.39541500	-0.38254300	-0.86844500
C	4.25599000	0.01577700	-1.90443600
C	5.63342700	-0.07161700	-1.74849000
C	6.16131000	-0.55951700	-0.55224700
H	5.72678700	-1.30339500	1.42605700
H	3.29099900	-1.12824100	1.17081100
H	3.83309900	0.39713600	-2.83111600

H	6.29313900	0.23980000	-2.55093200
H	7.23696500	-0.62639700	-0.42440300
C	1.95907600	-0.25793200	-1.10766300
H	1.69038500	0.38503100	-1.94148700
N	2.85810900	2.86258400	-1.33809400
N	3.61372800	3.65702900	-1.41461500

IN19

C	-4.19827400	-1.78850900	3.32715900
C	-3.08514700	-1.51808300	2.53587600
C	-2.55396600	-0.22812300	2.44828800
C	-3.09649300	0.75326800	3.28640300
C	-4.20529300	0.48993700	4.08869800
C	-4.78146400	-0.77772000	4.08964300
H	-4.59891000	-2.79692500	3.36114300
H	-2.60885000	-2.33110800	2.00630700
H	-2.64190400	1.73874200	3.33037200
H	-4.61234100	1.27596500	4.71655300
H	-5.65138400	-0.98517000	4.70376300
C	-1.30478400	0.10219100	1.62211600
H	-1.18447000	1.18255300	1.74011400
C	0.48364700	0.35344900	3.36615300
C	-0.15790400	-0.49686500	2.45397100
C	0.23606100	-1.84027700	2.43197800
C	1.27930400	-2.29338200	3.23867100
C	1.93969200	-1.42304900	4.10441200
C	1.52428000	-0.09506200	4.17672400
H	0.15822000	1.38871100	3.45560500
H	-0.25790000	-2.55164100	1.78616700
H	1.56778400	-3.33901400	3.18746300
H	2.75113500	-1.77918200	4.73083300
H	2.00975800	0.59576800	4.85829800

C	-1.18916500	-2.75466700	-0.31101300
C	-1.85933300	-3.82805300	0.28969500
C	0.09324800	-2.99927600	-0.80807100
C	-1.22733600	-5.05261700	0.49781400
H	-2.89709700	-3.71327300	0.59107700
C	0.74099900	-4.21243200	-0.59374900
H	0.60228600	-2.24904800	-1.39296300
C	0.09100300	-5.24066200	0.08638400
H	-1.76934800	-5.86094600	0.97800700
H	1.74917200	-4.35302700	-0.97401900
H	0.59005400	-6.18824400	0.25982400
C	-3.67121200	0.90162900	-0.95394100
C	-4.14817400	1.21245600	-2.23514300
C	-4.60139400	0.53993300	0.02139500
C	-5.49932500	1.13074000	-2.54583100
H	-3.44132200	1.49416800	-3.01333600
C	-5.96005700	0.44494900	-0.28912300
H	-4.29045500	0.31740100	1.03276400
C	-6.41565200	0.73327600	-1.57151700
H	-5.83513900	1.36224000	-3.55173500
H	-6.65964200	0.14810200	0.48642000
H	-7.47182600	0.65806800	-1.80913600
C	-1.40957900	4.81092200	-1.11146400
C	-1.49793900	3.44161400	-1.34988400
C	-1.98002400	2.56141600	-0.37459700
C	-2.44985500	3.11299900	0.82168400
C	-2.34268800	4.47798700	1.07906000
C	-1.80747800	5.33399400	0.11708400
H	-1.03506100	5.46946700	-1.88994600
H	-1.19645900	3.05041900	-2.31874300
H	-2.95924600	2.47623900	1.53383500
H	-2.71259100	4.87799300	2.01826000

H	-1.73905200	6.39993700	0.30867600
C	-2.16221400	1.08555100	-0.73537500
H	-1.75470300	1.00766800	-1.74989600
C	-3.48026400	-1.82739900	-4.58146300
C	-4.25850800	-2.10689200	-3.46003100
C	-3.72959600	-1.93989100	-2.18554200
C	-2.41596500	-1.49636900	-1.98438400
C	-1.63433400	-1.26590700	-3.12214000
C	-2.16392500	-1.41134700	-4.40497500
H	-3.88919500	-1.94373800	-5.57960200
H	-5.28579600	-2.43790100	-3.57397800
H	-4.36459400	-2.12281900	-1.32154300
H	-0.58724400	-0.98787600	-3.04945000
H	-1.53530600	-1.21385200	-5.26784800
C	-1.92852000	-1.43386600	-0.52379900
H	-2.85188700	-1.50717600	0.05543000
C	-1.31341000	-0.05504900	0.02758100
C	1.47368300	3.67526200	0.33552600
C	0.96562300	2.39124100	0.49518800
C	0.87763600	1.51535100	-0.60613700
C	1.36293600	1.95020400	-1.86105000
C	1.85375600	3.23311500	-2.01917500
C	1.90667800	4.09987100	-0.91861800
H	1.52023200	4.34340500	1.18873900
H	0.64679200	2.06432700	1.47714000
H	1.31198300	1.28546700	-2.71849900
H	2.19106700	3.56843000	-2.99430300
H	2.29234300	5.10705600	-1.04405900
C	0.14765600	0.12296200	-0.55287200
H	0.12264500	-0.20897100	-1.58549200
C	3.81175600	-1.89252600	-2.33345500
C	2.72348400	-1.15697300	-1.89987500

C	2.57943300	-0.84712800	-0.53239100
C	3.55054400	-1.29434400	0.37865300
C	4.62645500	-2.06293300	-0.05711000
C	4.76269000	-2.35276200	-1.41031000
H	3.92473300	-2.12203000	-3.38764100
H	1.98165200	-0.83268100	-2.62468300
H	3.43832200	-1.05346200	1.43485200
H	5.36675200	-2.41145000	0.65416700
H	5.61259700	-2.93213800	-1.75643100
C	1.42913600	-0.18837700	0.05599500
H	1.54773400	0.02257500	1.11260000
C	7.64557100	-0.30450200	0.36178600
C	6.56372700	0.48279200	0.74367700
C	5.75776100	1.09585600	-0.22720500
C	6.07121700	0.91911300	-1.58198100
C	7.15482000	0.13168300	-1.95687000
C	7.94349400	-0.49061900	-0.98839900
H	8.26552000	-0.76679900	1.12369000
H	6.35032500	0.62827300	1.80002000
H	5.45339900	1.39386800	-2.34009500
H	7.38646900	0.00497000	-3.01000100
H	8.79307100	-1.09799900	-1.28213900
C	4.58858800	1.89811700	0.12132400
H	4.06211400	2.52607600	-0.58384500
N	4.08812000	1.85464100	1.31685400
N	3.65500900	1.77982300	2.36808900

TS14

C	-3.40789600	-0.74608900	4.17890600
C	-2.48684100	-0.70389300	3.13391200
C	-2.05744300	0.50791400	2.58432700
C	-2.52729200	1.68103800	3.19363000

C	-3.44471200	1.65096600	4.24003000
C	-3.90551000	0.43128800	4.72977700
H	-3.73286300	-1.70823800	4.56215600
H	-2.12382000	-1.64200600	2.74648400
H	-2.16238100	2.64527100	2.84766000
H	-3.79398900	2.58253400	4.67361600
H	-4.62682500	0.39920900	5.53936200
C	-0.96349900	0.67479500	1.50945400
H	-1.06558600	1.71867000	1.20443000
C	0.93234900	1.87753000	2.66718800
C	0.31556200	0.66153600	2.35683500
C	0.84799100	-0.49913300	2.92943300
C	2.00433000	-0.45908400	3.70369900
C	2.64149200	0.75642100	3.95925700
C	2.08410000	1.92981600	3.45580400
H	0.50312500	2.80846300	2.30300400
H	0.37032300	-1.45196700	2.75776400
H	2.39809700	-1.38194300	4.11965800
H	3.53538700	0.79162600	4.57403500
H	2.53308000	2.89131300	3.68817700
C	-0.24660400	-2.68288700	0.53642600
C	-0.32670900	-3.35832400	1.76187900
C	0.68145600	-3.17846400	-0.38714600
C	0.54465900	-4.39530800	2.09233500
H	-1.09435300	-3.10023500	2.48251800
C	1.55529400	-4.21397700	-0.07419100
H	0.69455000	-2.78436000	-1.39571900
C	1.50714400	-4.81823800	1.18012500
H	0.44711400	-4.88824700	3.05450100
H	2.25668500	-4.56465800	-0.82535900
H	2.17799000	-5.63503100	1.42593600
C	-3.60966400	-0.07021600	-0.78480800

C	-4.22222900	-0.37389200	-2.00913800
C	-4.36569700	-0.22934000	0.37653200
C	-5.51420300	-0.87940400	-2.06950400
H	-3.66429000	-0.23806200	-2.93263800
C	-5.66058800	-0.74961000	0.32394800
H	-3.96563200	0.05063600	1.34003000
C	-6.23810800	-1.08852400	-0.89513200
H	-5.95099200	-1.12216800	-3.03324200
H	-6.21767300	-0.87575600	1.24756000
H	-7.24384900	-1.49421800	-0.93393300
C	-2.72779000	3.97216200	-2.44214000
C	-2.40185200	2.63459700	-2.24433100
C	-2.46476100	2.04587400	-0.97517400
C	-2.96353900	2.82439000	0.07212200
C	-3.26964700	4.17240400	-0.11066100
C	-3.13678300	4.75974100	-1.36693600
H	-2.65950200	4.39932700	-3.43817000
H	-2.07977400	2.03774600	-3.09361200
H	-3.16924900	2.36625900	1.03039800
H	-3.64711000	4.75334000	0.72572500
H	-3.38498200	5.80587300	-1.51394400
C	-2.19707500	0.53909700	-0.85544500
H	-1.85046500	0.25631000	-1.85345800
C	-3.23541400	-3.72222000	-3.20182300
C	-3.65009200	-3.87882300	-1.88001600
C	-3.01463000	-3.17728500	-0.86307900
C	-1.95863900	-2.29418000	-1.12351800
C	-1.53854600	-2.16960500	-2.45209100
C	-2.17317700	-2.86816000	-3.47997500
H	-3.73183500	-4.26010400	-4.00267800
H	-4.47905400	-4.53714000	-1.64059200
H	-3.36532500	-3.29293700	0.16027300

H	-0.72097300	-1.51137400	-2.73113800
H	-1.83212800	-2.73830100	-4.50272400
C	-1.30757600	-1.64531500	0.11819500
H	-2.10076200	-1.70701900	0.86306700
C	-1.00676000	-0.06574600	0.08312600
C	0.58143100	3.98646500	-1.57874500
C	0.40684500	2.80442200	-0.86673000
C	0.43373700	1.56411800	-1.51193300
C	0.63019000	1.54392400	-2.89483300
C	0.80998800	2.72626700	-3.61658000
C	0.79244000	3.95254700	-2.95845300
H	0.53682300	4.93801300	-1.05718600
H	0.24056600	2.85473200	0.20292100
H	0.63024700	0.59511500	-3.42706300
H	0.95581600	2.68412300	-4.69133200
H	0.92632200	4.87485200	-3.51393900
C	0.36405400	0.22706800	-0.76686100
H	0.30540800	-0.51930900	-1.55811400
C	3.70914400	-2.31959800	-2.34917200
C	2.75388900	-1.39930400	-1.95274500
C	2.68360500	-0.97607000	-0.60623200
C	3.62362800	-1.48678400	0.31470600
C	4.57229500	-2.41956400	-0.08389400
C	4.61595300	-2.83542800	-1.41322000
H	3.75611700	-2.64136900	-3.38428400
H	2.05811200	-1.00125000	-2.68578700
H	3.56672100	-1.16911000	1.35268700
H	5.27778700	-2.81788300	0.63754200
H	5.35820500	-3.56248700	-1.72849900
C	1.73584900	-0.01372000	-0.12349900
H	1.88256200	0.26914400	0.90712900
C	6.55145800	0.54321700	0.91475000

C	5.29356400	1.12579800	0.80842400
C	4.66190000	1.20941400	-0.44255600
C	5.30131900	0.69491100	-1.57699200
C	6.55155700	0.09736200	-1.45964000
C	7.18036600	0.02157000	-0.21680500
H	7.03952800	0.49079300	1.88237900
H	4.79660400	1.50488400	1.69996700
H	4.81865300	0.76972100	-2.54731400
H	7.04161200	-0.29964800	-2.34285900
H	8.16160200	-0.43338600	-0.13113100
C	3.31212900	1.76773200	-0.59168200
H	2.89475600	1.94799500	-1.57796400
N	3.06241700	2.80268500	0.23895800
N	2.87486600	3.61803900	0.97802300

IN20

C	2.90165800	-0.01176300	4.34385200
C	2.02233300	0.05683300	3.26128600
C	1.81654700	-1.03828900	2.40931000
C	2.52337800	-2.21093900	2.73322200
C	3.40067400	-2.28787400	3.80902600
C	3.60200000	-1.17787700	4.62570000
H	3.03631500	0.86980500	4.96258800
H	1.53552900	1.00668800	3.10847500
H	2.39954100	-3.09858500	2.12183400
H	3.92851700	-3.21622200	4.00233900
H	4.28924300	-1.22314700	5.46388100
C	0.89638700	-1.13497600	1.15257400
H	1.42256300	-1.87109300	0.55096400
C	-0.09810900	-3.41113200	1.43552700
C	-0.31091500	-2.02531400	1.60056300
C	-1.47995000	-1.68350500	2.30264300

C	-2.35297200	-2.64777600	2.81748700
C	-2.10002200	-4.00219200	2.65043900
C	-0.95878800	-4.37902300	1.94046800
H	0.78258100	-3.75007100	0.89450300
H	-1.73898600	-0.65542100	2.49915500
H	-3.23350000	-2.32051400	3.36306900
H	-2.77140300	-4.74920600	3.06050300
H	-0.73175200	-5.42901600	1.78390700
C	-0.16004800	2.16044800	1.55455600
C	-0.70098200	1.42671400	2.61556900
C	-0.54488300	3.50999500	1.47643400
C	-1.62156100	1.96409300	3.51363200
H	-0.35710800	0.41968300	2.78194200
C	-1.44403400	4.06775900	2.38560300
H	-0.14928400	4.15203900	0.70004900
C	-2.00549500	3.29893900	3.40083200
H	-2.00400100	1.34660500	4.32235300
H	-1.70576600	5.11705500	2.28913000
H	-2.70575700	3.73562000	4.10549500
C	3.49534300	0.63753600	-0.50921900
C	4.11462800	1.39449700	-1.51334300
C	4.18589900	0.46415700	0.69195400
C	5.35219900	1.99384000	-1.31530900
H	3.59968000	1.54279400	-2.46076800
C	5.42053000	1.08075000	0.90545600
H	3.77933500	-0.15133300	1.48235200
C	6.00818500	1.85249700	-0.09239200
H	5.79648400	2.58453700	-2.11037300
H	5.92388100	0.94068500	1.85734100
H	6.96994700	2.32680700	0.07401700
C	3.13459400	-2.75347100	-3.42575300
C	2.64786200	-1.58549300	-2.84683100

C	2.63626600	-1.40308800	-1.45898700
C	3.23495900	-2.39940400	-0.67689200
C	3.70503700	-3.58231600	-1.24563400
C	3.63991500	-3.77502200	-2.62403000
H	3.11760300	-2.86314200	-4.50593200
H	2.26136700	-0.79995200	-3.48962200
H	3.39350800	-2.23654300	0.38386600
H	4.15768400	-4.33772900	-0.61006900
H	4.01479500	-4.69076200	-3.06946200
C	2.17444800	-0.04915300	-0.89383800
H	1.84573700	0.51431700	-1.77321000
C	2.14790700	4.91212400	-1.88746000
C	2.87117700	4.53757200	-0.75747300
C	2.47507100	3.43276100	-0.01056400
C	1.35095800	2.67003100	-0.35336600
C	0.60492400	3.09537400	-1.46094700
C	1.00885400	4.18823200	-2.22998200
H	2.45525000	5.76700100	-2.48101900
H	3.75429600	5.09436700	-0.46047200
H	3.07011300	3.14249300	0.85294500
H	-0.34668300	2.64150200	-1.71088700
H	0.40625400	4.48811900	-3.08213100
C	0.93839600	1.56871200	0.63946500
H	1.81637100	1.49604000	1.28650200
C	0.84910500	0.06084800	0.06903700
C	-0.23229100	-3.42452900	-2.86693500
C	-0.20338300	-2.50365700	-1.82181300
C	-0.31486500	-1.12873600	-2.05170100
C	-0.41275500	-0.71119200	-3.38725500
C	-0.44770700	-1.62319300	-4.44126400
C	-0.36956400	-2.98972400	-4.18347700
H	-0.11992200	-4.48262000	-2.64993300

H	-0.05378800	-2.87025800	-0.81647800
H	-0.41791400	0.35285600	-3.61281100
H	-0.51615400	-1.26218600	-5.46277200
H	-0.38645300	-3.70489000	-4.99934400
C	-0.36641600	-0.05286500	-0.96357700
H	-0.21841400	0.85446800	-1.54070200
C	-3.31509200	2.81052100	-2.72091800
C	-2.68366900	1.65411300	-2.26959300
C	-2.50851300	1.42213200	-0.89948400
C	-3.02419900	2.34619900	0.00815400
C	-3.65073500	3.50704600	-0.43996300
C	-3.79290400	3.74667200	-1.80466700
H	-3.43534400	2.97737100	-3.78649200
H	-2.32497400	0.93461900	-2.99910300
H	-2.92490300	2.16891000	1.07539500
H	-4.02423700	4.22443800	0.28402500
H	-4.27989500	4.65150000	-2.15308000
C	-1.83701200	0.13479200	-0.39088100
H	-1.81279300	0.19577400	0.69423400
C	-5.88141600	-0.21775700	1.46176200
C	-4.56790800	-0.36435900	1.03151000
C	-4.31000700	-0.70264700	-0.30084800
C	-5.36640100	-0.89497700	-1.19368600
C	-6.68059500	-0.73853400	-0.76060000
C	-6.93717000	-0.40464100	0.56746600
H	-6.08332500	0.05083300	2.49320400
H	-3.75119700	-0.19562900	1.72650900
H	-5.16244900	-1.14849700	-2.23112300
H	-7.49926800	-0.87481300	-1.45866900
H	-7.96052200	-0.28405900	0.90712300
C	-2.90590500	-0.87904500	-0.81360700
H	-2.88054200	-1.04167000	-1.89677000

N	-2.61364300	-2.39791000	-0.39253800
N	-2.75751000	-3.47979300	-0.25230500

TS15

C	2.88805500	-0.02373200	4.34998400
C	2.01197200	0.04845500	3.26502100
C	1.80561800	-1.04514700	2.41127000
C	2.50828200	-2.22011000	2.73585000
C	3.38233900	-2.30061600	3.81401800
C	3.58442200	-1.19206600	4.63248300
H	3.02340200	0.85677900	4.97007700
H	1.52825500	0.99980800	3.11179100
H	2.38364600	-3.10674100	2.12316800
H	3.90707600	-3.23062600	4.00780000
H	4.26919700	-1.24013400	5.47252700
C	0.88899600	-1.13771100	1.15169200
H	1.41491100	-1.87448400	0.55072100
C	-0.11229800	-3.41158900	1.42897500
C	-0.32207200	-2.02544600	1.59483000
C	-1.49229800	-1.68140400	2.29388600
C	-2.36937500	-2.64384600	2.80502500
C	-2.11921900	-3.99873000	2.63744600
C	-0.97688200	-4.37774600	1.93049100
H	0.76894600	-3.75220900	0.88989700
H	-1.74927200	-0.65299100	2.49095000
H	-3.25087500	-2.31469900	3.34789500
H	-2.79371400	-4.74447400	3.04470000
H	-0.75202400	-5.42815900	1.77362000
C	-0.15549500	2.16177300	1.55547600
C	-0.70430900	1.42722500	2.61182700
C	-0.53241100	3.51378300	1.48097400
C	-1.62560700	1.96620500	3.50818900

H	-0.36609700	0.41791200	2.77582800
C	-1.43212000	4.07307300	2.38870600
H	-0.13013400	4.15653900	0.70860900
C	-2.00199000	3.30350400	3.39868700
H	-2.01442100	1.34786600	4.31322700
H	-1.68759100	5.12418800	2.29529000
H	-2.70277100	3.74137500	4.10209600
C	3.49877100	0.62645100	-0.50077800
C	4.12462000	1.38159700	-1.50220400
C	4.18485500	0.44879000	0.70231200
C	5.36427700	1.97517000	-1.29972100
H	3.61336500	1.53302800	-2.45113000
C	5.42158900	1.05959300	0.92029200
H	3.77317500	-0.16566300	1.49082800
C	6.01582500	1.82968200	-0.07492600
H	5.81373100	2.56454900	-2.09285600
H	5.92131400	0.91628200	1.87359900
H	6.97919500	2.29948000	0.09493600
C	3.13148000	-2.75758100	-3.42447000
C	2.64778100	-1.58879400	-2.84465400
C	2.63377600	-1.40871700	-1.45653300
C	3.22688100	-2.40862800	-0.67475300
C	3.69377300	-3.59232800	-1.24447000
C	3.63114000	-3.78238500	-2.62335100
H	3.11646000	-2.86538100	-4.50486600
H	2.26553800	-0.80078700	-3.48697900
H	3.38360800	-2.24821900	0.38667200
H	4.14202500	-4.35058600	-0.60918200
H	4.00357800	-4.69877200	-3.06949900
C	2.17596600	-0.05391400	-0.89017600
H	1.85190200	0.51243700	-1.76943500
C	2.16409100	4.90869700	-1.88174200

C	2.88718200	4.52929000	-0.75330000
C	2.48733100	3.42501300	-0.00747800
C	1.35955700	2.66764200	-0.35003400
C	0.61395500	3.09795400	-1.45604700
C	1.02148700	4.19021000	-2.22394300
H	2.47432900	5.76320100	-2.47433100
H	3.77299000	5.08191700	-0.45651100
H	3.08205100	3.13116800	0.85504000
H	-0.33985800	2.64844400	-1.70541400
H	0.41905300	4.49394400	-3.07484800
C	0.94232400	1.56701100	0.64163300
H	1.81908900	1.49093600	1.28998400
C	0.84865900	0.05977000	0.06954600
C	-0.23797100	-3.41720100	-2.87410600
C	-0.20852400	-2.49809500	-1.82748700
C	-0.31552700	-1.12253100	-2.05552900
C	-0.40911800	-0.70258000	-3.39065300
C	-0.44457000	-1.61281000	-4.44616600
C	-0.37111600	-2.97995800	-4.19025200
H	-0.12938900	-4.47596600	-2.65849100
H	-0.06218000	-2.86666100	-0.82241300
H	-0.41028900	0.36184600	-3.61453700
H	-0.50961300	-1.25003300	-5.46726900
H	-0.38838000	-3.69383100	-5.00725100
C	-0.36479200	-0.04791000	-0.96614900
H	-0.21166500	0.85954100	-1.54180200
C	-3.29696200	2.82579800	-2.73258900
C	-2.67465400	1.66540600	-2.27867300
C	-2.50193100	1.43517800	-0.90790000
C	-3.01174000	2.36460500	-0.00216400
C	-3.62856700	3.52938000	-0.45308900
C	-3.76784800	3.76743400	-1.81850400

H	-3.41534300	2.99138700	-3.79855500
H	-2.32041900	0.94207300	-3.00646400
H	-2.91448000	2.18895100	1.06546200
H	-3.99669700	4.25125200	0.26918700
H	-4.24740700	4.67542400	-2.16898500
C	-1.83533800	0.14544100	-0.39569100
H	-1.81253800	0.20914000	0.68921500
C	-5.87498300	-0.20127800	1.46715200
C	-4.56269100	-0.33724600	1.03018300
C	-4.30888900	-0.68826000	-0.30001300
C	-5.36933300	-0.90383500	-1.18343600
C	-6.68236500	-0.75829800	-0.74382600
C	-6.93415100	-0.41166200	0.58202100
H	-6.07361500	0.07708500	2.49662600
H	-3.74392500	-0.15094000	1.71821200
H	-5.16891400	-1.16790000	-2.21897300
H	-7.50394900	-0.91290300	-1.43460700
H	-7.95667300	-0.29949800	0.92707600
C	-2.90965100	-0.85632200	-0.81994700
H	-2.88493600	-1.02612100	-1.90167000
N	-2.61438100	-2.39918000	-0.39962900
N	-2.77574900	-3.47977500	-0.26869100

IN21

C	-2.32883100	-1.01626200	4.45943000
C	-1.63456500	-0.92724300	3.25573000
C	-1.27384600	0.30696800	2.70755800
C	-1.53381900	1.44740700	3.47956800
C	-2.22269700	1.37077500	4.68783600
C	-2.64666700	0.13650300	5.17391500
H	-2.61258300	-1.99158300	4.84223100
H	-1.36242600	-1.83935800	2.74890600

H	-1.19111100	2.41925700	3.13379700
H	-2.42268900	2.27771900	5.24929700
H	-3.19314300	0.07154100	6.10880500
C	-0.43292500	0.46751300	1.42409800
H	-0.45366000	1.54311600	1.22928900
C	1.69440100	1.32376600	2.45890900
C	0.97296300	0.20697600	1.98879500
C	1.43182100	-1.06580800	2.37205700
C	2.55064500	-1.20945900	3.18003500
C	3.24789500	-0.08578900	3.64161000
C	2.81189500	1.18431000	3.27907600
H	1.32750000	2.32202400	2.23009800
H	0.89628300	-1.95264200	2.05473800
H	2.87621400	-2.20459400	3.46826000
H	4.11296800	-0.20644000	4.28610900
H	3.33070900	2.06735600	3.64032900
C	-0.42694400	-2.83838100	0.15909100
C	-0.55306400	-3.71002700	1.24864200
C	0.42909100	-3.24237200	-0.87404200
C	0.22365300	-4.86310000	1.36382200
H	-1.29566500	-3.51731800	2.01568600
C	1.20985000	-4.39102100	-0.77123000
H	0.45318400	-2.67297400	-1.79921900
C	1.13007400	-5.19890800	0.36311700
H	0.09787600	-5.50869000	2.22718700
H	1.85271800	-4.68277800	-1.59758000
H	1.72776300	-6.10120700	0.43952400
C	-3.60076300	0.21529900	-0.24957100
C	-4.49067100	0.10790800	-1.32745800
C	-4.10050100	-0.01528700	1.03297200
C	-5.81459900	-0.26862500	-1.14131400
H	-4.12946100	0.29516700	-2.33631800

C	-5.42706000	-0.40649400	1.22544400
H	-3.46942600	0.10351700	1.90316300
C	-6.28832400	-0.54476800	0.14155400
H	-6.47267400	-0.35996900	-1.99983100
H	-5.78349900	-0.59218300	2.23434200
H	-7.31862300	-0.84991300	0.29328100
C	-2.56796700	4.34240500	-1.67152000
C	-2.38281800	2.96358000	-1.68532700
C	-2.28213700	2.22718300	-0.49905400
C	-2.48787400	2.91258600	0.70002100
C	-2.65344800	4.29649600	0.72747000
C	-2.67407800	5.02401600	-0.46023800
H	-2.62948400	4.88361600	-2.61093300
H	-2.30733700	2.45090200	-2.63937200
H	-2.58069200	2.35486700	1.62117100
H	-2.80355700	4.79940600	1.67846500
H	-2.81116400	6.10039800	-0.44427400
C	-2.17824700	0.69746400	-0.58019100
H	-2.08323100	0.49476000	-1.65145000
C	-4.09349300	-3.20434300	-3.04112100
C	-4.29953400	-3.41802900	-1.67873400
C	-3.41718300	-2.88206600	-0.74967400
C	-2.31117800	-2.11471600	-1.13980200
C	-2.09968000	-1.93839000	-2.51073300
C	-2.98692800	-2.46744400	-3.44997000
H	-4.78358300	-3.61060800	-3.77316800
H	-5.15785200	-3.98759500	-1.33688000
H	-3.61055700	-3.02992500	0.31073700
H	-1.25344900	-1.37615700	-2.89045400
H	-2.80403500	-2.30003400	-4.50704400
C	-1.38356400	-1.64450400	0.00360300
H	-2.03762300	-1.67602100	0.87642300

C	-0.88910300	-0.11439000	0.01008200
C	0.90596200	3.91825800	-1.56765100
C	0.73046500	2.69008600	-0.93479600
C	0.37219800	1.54644400	-1.66062500
C	0.19875600	1.68106600	-3.04225900
C	0.37858800	2.90395100	-3.68495600
C	0.73686900	4.02920800	-2.94766800
H	1.15586400	4.79619600	-0.98023100
H	0.84844000	2.64286500	0.14318700
H	-0.06399500	0.80529100	-3.63223500
H	0.24019000	2.97426500	-4.75927400
H	0.87260200	4.98737200	-3.43861300
C	0.27901900	0.14962700	-1.06198900
H	-0.02293100	-0.47810100	-1.90035100
C	3.82290200	0.58833000	-3.92938900
C	3.04788600	0.69736700	-2.77553700
C	2.56922200	-0.45289600	-2.12862200
C	2.94060200	-1.70585900	-2.63230500
C	3.70313900	-1.81161700	-3.79123800
C	4.14366100	-0.66233400	-4.44807200
H	4.17527700	1.49030200	-4.41903400
H	2.81923900	1.68828700	-2.39705600
H	2.63480600	-2.60851500	-2.11564500
H	3.95784200	-2.79313600	-4.17790100
H	4.74283500	-0.74421200	-5.34886700
C	1.74051900	-0.38887700	-0.81292600
H	1.67924200	-1.40695300	-0.43440600
C	5.38123400	-2.07295600	1.01731800
C	4.15728200	-1.59825900	0.58511800
C	3.91762600	-0.20237500	0.52764000
C	4.93091400	0.68596800	0.95330600
C	6.15845600	0.20409600	1.38412500

C	6.38185900	-1.17301100	1.41022400
H	5.56772700	-3.14059600	1.05381700
H	3.38104400	-2.30064700	0.29268200
H	4.73893300	1.75444200	0.91977900
H	6.93789600	0.89098900	1.69428600
H	7.34243100	-1.55445600	1.74299400
C	2.72224700	0.35821300	0.00214600
H	2.68266200	1.44642500	-0.02426300
N	3.28967500	3.91432000	0.98025500
N	2.93419500	4.87676200	1.37374600

IN19-2

C	3.04173100	1.52989200	4.19701000
C	2.20906800	1.31095200	3.10291700
C	1.80090800	0.02197700	2.74922000
C	2.14729200	-1.02639400	3.60979000
C	2.97241500	-0.81567000	4.71317100
C	3.44935800	0.46184300	4.99487400
H	3.35658800	2.54075100	4.43699700
H	1.84753900	2.15956700	2.53900900
H	1.76059800	-2.02512900	3.42995100
H	3.23623400	-1.65115500	5.35354900
H	4.10111200	0.62939500	5.84581100
C	0.85065500	-0.25207800	1.57724200
H	0.74906700	-1.34050300	1.56842300
C	-1.32373600	-0.68649700	2.74739000
C	-0.50860600	0.24893900	2.09325100
C	-0.96139600	1.57186600	2.03320500
C	-2.22150200	1.91978300	2.51993800
C	-3.04804800	0.96388800	3.10800700
C	-2.57820900	-0.34135300	3.24482100
H	-0.96324300	-1.70502900	2.88621800

H	-0.35216400	2.34492200	1.58581200
H	-2.55279600	2.95026200	2.43398200
H	-4.03332300	1.23505100	3.47357600
H	-3.17908300	-1.09123800	3.75050000
C	1.20896400	2.76338700	-0.07043700
C	1.62729200	3.78115900	0.79622000
C	0.13324900	3.04545600	-0.91596900
C	0.92168900	4.97853800	0.90235600
H	2.52722200	3.64569100	1.38999500
C	-0.58916400	4.23040100	-0.80943600
H	-0.14622100	2.34937900	-1.69154000
C	-0.21285800	5.19599300	0.12261400
H	1.26406300	5.74328100	1.59206800
H	-1.43648500	4.39893800	-1.46854900
H	-0.77205300	6.12124500	0.21367900
C	3.85924900	-0.84162500	-0.27213200
C	4.68684800	-1.06548100	-1.38179400
C	4.46664200	-0.54768300	0.94908100
C	6.06884700	-0.96685000	-1.28776700
H	4.23590300	-1.29219100	-2.34604500
C	5.85593200	-0.43773700	1.04663000
H	3.87708400	-0.38681500	1.84084600
C	6.66286500	-0.64045500	-0.06804700
H	6.68117900	-1.12994400	-2.16902600
H	6.30038800	-0.19507500	2.00714900
H	7.74155300	-0.55268800	0.01135800
C	1.70849400	-4.68253400	-1.41620800
C	1.85785400	-3.29981500	-1.49622200
C	2.08402200	-2.52404200	-0.35408000
C	2.23499900	-3.19256200	0.86578500
C	2.06275600	-4.57153300	0.95977800
C	1.78459500	-5.32347600	-0.18167100

H	1.53985200	-5.25896100	-2.32134500
H	1.81198500	-2.81553600	-2.46897400
H	2.55171700	-2.63395400	1.73802900
H	2.18446900	-5.06430200	1.91961700
H	1.66896300	-6.40037700	-0.11379800
C	2.35439600	-1.02739000	-0.51388400
H	2.25747300	-0.85417500	-1.59129800
C	4.70043900	2.18243600	-3.52343000
C	5.10495600	2.36652300	-2.20288400
C	4.22334900	2.10304000	-1.16092500
C	2.91762500	1.65378200	-1.39651300
C	2.51296400	1.52217100	-2.72967400
C	3.39779400	1.76531100	-3.78083600
H	5.38627300	2.37389100	-4.34199900
H	6.11382200	2.69837500	-1.97948300
H	4.56795700	2.21432200	-0.13539300
H	1.49544200	1.24945100	-2.99284200
H	3.05768000	1.64295400	-4.80454800
C	2.01903400	1.47020600	-0.15915200
H	2.72403700	1.49556700	0.67517700
C	1.30296900	0.04722200	0.06917200
C	-1.45159600	-3.64419900	-0.61690400
C	-1.01165300	-2.37973900	-0.25596900
C	-0.66840000	-1.42702200	-1.24406600
C	-0.81105500	-1.78739200	-2.60649400
C	-1.23886900	-3.05202900	-2.96186500
C	-1.56247800	-3.98114700	-1.96645400
H	-1.69801400	-4.36911700	0.15141400
H	-0.94774100	-2.11550700	0.79105100
H	-0.56541200	-1.06978100	-3.38266400
H	-1.33564800	-3.31712600	-4.00876200
H	-1.90823000	-4.97114600	-2.24767700

C	0.08217700	-0.06606400	-0.92104500
H	0.41328200	0.29334300	-1.89001500
C	-2.90404600	2.04175900	-3.63381800
C	-1.98342300	1.29329500	-2.91853200
C	-2.25664700	0.91123800	-1.59337000
C	-3.46195400	1.31434500	-1.00020000
C	-4.37920800	2.08111600	-1.71575900
C	-4.10643300	2.43555400	-3.03336600
H	-2.68780400	2.32963600	-4.65699100
H	-1.04536600	1.02281700	-3.39573300
H	-3.67311400	1.03708600	0.03037000
H	-5.30449100	2.38943800	-1.24025000
H	-4.82176100	3.02528300	-3.59750400
C	-1.32667700	0.19268900	-0.72030800
H	-1.74678100	-0.02644000	0.25292500
C	-7.06119200	-0.21883900	2.33739700
C	-5.93474000	-0.90183600	1.89137300
C	-5.62855100	-0.95463400	0.52353100
C	-6.48475600	-0.31671100	-0.38706700
C	-7.61138300	0.36338800	0.06648100
C	-7.90385700	0.42123800	1.42867100
H	-7.28671100	-0.19367400	3.39892600
H	-5.28939800	-1.40925400	2.60230000
H	-6.27592200	-0.35653800	-1.45337200
H	-8.26910600	0.84420100	-0.65102300
H	-8.78476200	0.94921000	1.77732100
C	-4.42279200	-1.66132700	0.09608400
H	-3.74154100	-2.15131700	0.77868700
N	-4.13838700	-1.74926400	-1.16937000
N	-3.94496900	-1.77055200	-2.29123900

TS14-2

C	2.82289600	-0.94890400	4.36992100
C	2.00686000	-0.59577400	3.29584500
C	1.75676400	-1.48183300	2.24182300
C	2.28708000	-2.77477600	2.36935600
C	3.10119700	-3.13857400	3.43745500
C	3.39147100	-2.21671700	4.44042300
H	3.00974800	-0.22211100	5.15416200
H	1.59132100	0.39939600	3.30154600
H	2.05127400	-3.52385900	1.61838100
H	3.50329500	-4.14547500	3.48543900
H	4.03244300	-2.48866800	5.27223500
C	0.80837600	-1.23228600	1.04359200
H	1.07520600	-2.02162200	0.33873300
C	-0.83941700	-3.10990300	1.37139100
C	-0.51947800	-1.76985700	1.63866300
C	-1.23750000	-1.14091400	2.66624800
C	-2.19279500	-1.82358500	3.41402300
C	-2.46262200	-3.16870600	3.15980100
C	-1.79423200	-3.80398700	2.11779300
H	-0.27210500	-3.65889000	0.62215000
H	-1.02279500	-0.11106200	2.91777900
H	-2.70969800	-1.30561300	4.21636500
H	-3.18420500	-3.71061200	3.76290200
H	-1.98719600	-4.84987000	1.89986800
C	0.01425300	2.25336700	1.36255300
C	-0.39402900	2.13330000	2.69738300
C	-0.61223900	3.26027900	0.60768600
C	-1.41703500	2.91671000	3.23445900
H	0.11266200	1.44674000	3.36369500
C	-1.61963100	4.05762000	1.13464200
H	-0.29793200	3.44990900	-0.41174800
C	-2.04158500	3.88222400	2.45224900

H	-1.69459200	2.79132000	4.27653300
H	-2.07210400	4.82402600	0.51225800
H	-2.82291500	4.50924000	2.87003400
C	3.67650000	0.29856300	-0.40449300
C	4.44347400	1.00766000	-1.34052300
C	4.27408000	-0.01650700	0.81604500
C	5.73028800	1.44050400	-1.04913500
H	4.01255600	1.24590900	-2.31022500
C	5.56521500	0.42190800	1.11820600
H	3.75094000	-0.60376700	1.55536400
C	6.29624100	1.16174200	0.19554800
H	6.28775700	2.00432500	-1.79071500
H	5.99554400	0.17100000	2.08318800
H	7.29776900	1.50504800	0.43360900
C	2.85724300	-2.73506200	-3.65117600
C	2.53076700	-1.56722800	-2.96764200
C	2.55110400	-1.50641100	-1.56984500
C	3.01970700	-2.63293800	-0.88607800
C	3.32858100	-3.81386100	-1.55829900
C	3.22996800	-3.87793000	-2.94695100
H	2.82189900	-2.74809200	-4.73645600
H	2.25203700	-0.68510800	-3.53599000
H	3.20407700	-2.57233100	0.17891000
H	3.68316700	-4.67276100	-0.99620100
H	3.47998800	-4.79272200	-3.47440200
C	2.28450600	-0.16162300	-0.87569800
H	2.05968800	0.51724900	-1.70325700
C	3.71075900	4.57877700	-0.98922600
C	3.88912500	4.15068500	0.32464300
C	3.08261300	3.14491600	0.84573700
C	2.08708300	2.52599400	0.08014400
C	1.91118600	2.98213500	-1.23190800

C	2.71399600	3.99129100	-1.76233400
H	4.34001600	5.35871500	-1.40500100
H	4.66484400	4.59020300	0.94344000
H	3.24981500	2.80760500	1.86618900
H	1.15582800	2.55422700	-1.88539800
H	2.55792900	4.31398300	-2.78730600
C	1.22865300	1.47411000	0.81330300
H	1.84464000	1.22011000	1.67331000
C	0.98820000	0.05906100	0.09175000
C	-0.62256900	-2.84868800	-3.15812100
C	-0.49000400	-2.05496900	-2.02390400
C	-0.34284400	-0.66765700	-2.12566800
C	-0.30964300	-0.10157500	-3.40220600
C	-0.44968200	-0.88950800	-4.54600500
C	-0.61616500	-2.26564600	-4.42682000
H	-0.71603900	-3.92494800	-3.05245300
H	-0.49062100	-2.52335800	-1.04774800
H	-0.15092200	0.96844900	-3.51727600
H	-0.42469800	-0.42374300	-5.52599400
H	-0.72414400	-2.88287700	-5.31253600
C	-0.25518300	0.25637900	-0.90478100
H	-0.06880300	1.24102900	-1.33914700
C	-3.48043200	3.12677300	-2.07275700
C	-2.54179200	2.13914300	-1.82895700
C	-2.59232900	1.38738400	-0.62721100
C	-3.63032200	1.65543300	0.30120300
C	-4.54840700	2.66774300	0.05899600
C	-4.47673100	3.39944200	-1.12515500
H	-3.43676100	3.69947000	-2.99296100
H	-1.75975000	1.95208500	-2.55546800
H	-3.67025800	1.08739900	1.22793600
H	-5.32040700	2.88147900	0.79061400

H	-5.19561400	4.19027100	-1.31598300
C	-1.63302400	0.39753500	-0.27556400
H	-1.82809500	-0.11641000	0.65853500
C	-5.82485200	-1.45222000	1.71982500
C	-4.69340000	-1.71044600	0.95900600
C	-4.56736400	-1.16600600	-0.32789800
C	-5.59775600	-0.36530600	-0.84169600
C	-6.73922800	-0.12757000	-0.07950900
C	-6.85517000	-0.66157200	1.20182800
H	-5.90984100	-1.87759300	2.71498900
H	-3.89614800	-2.33287300	1.34973800
H	-5.51980100	0.07211400	-1.83421300
H	-7.53736000	0.48250200	-0.48985100
H	-7.74538200	-0.47168600	1.79208700
C	-3.32447000	-1.42042900	-1.04425900
H	-2.64538800	-2.21782700	-0.77846900
N	-3.27866100	-1.00897400	-2.30777400
N	-3.31111200	-0.49534000	-3.30758700

IN20-2

C	-3.05608700	2.54038600	-3.39428900
C	-2.12416500	1.98237900	-2.52277600
C	-1.87022400	0.61085100	-2.50423600
C	-2.48610400	-0.16630900	-3.49453500
C	-3.41578200	0.38024200	-4.37619300
C	-3.72917000	1.73617100	-4.31099500
H	-3.24276300	3.60959900	-3.36373600
H	-1.57051400	2.63343400	-1.86691400
H	-2.23354800	-1.21909000	-3.59029700
H	-3.88858400	-0.25515900	-5.11824100
H	-4.46067500	2.16452200	-4.98806100
C	-0.80303900	-0.03881000	-1.59944100

H	-0.95915100	-1.10882800	-1.75148100
C	0.83303500	-0.63380700	-3.41060800
C	0.47034600	0.28412300	-2.41027300
C	1.07810400	1.54839700	-2.44995000
C	1.96649100	1.89072500	-3.46817400
C	2.26930000	0.98386500	-4.48412600
C	1.71163000	-0.29187300	-4.44003000
H	0.34800000	-1.60796800	-3.44536300
H	0.81472500	2.30412500	-1.71906500
H	2.39624600	2.88774100	-3.48021100
H	2.93014500	1.26744900	-5.29702800
H	1.93492700	-1.01560100	-5.21780300
C	-0.29293300	2.78672100	0.33313200
C	-0.91039700	3.93642000	-0.17992500
C	1.02458400	2.92220900	0.76926400
C	-0.20275900	5.12184300	-0.37110500
H	-1.97147300	3.91575800	-0.41655600
C	1.74639500	4.09769100	0.58163400
H	1.48109900	2.11496200	1.31729300
C	1.14262000	5.20088200	-0.01860000
H	-0.71105400	5.98838500	-0.78181600
H	2.77244400	4.16079600	0.93405200
H	1.69757500	6.12105300	-0.16804000
C	-3.53501700	-0.11060000	0.75752800
C	-4.16057700	-0.27622700	2.00031200
C	-4.26353300	0.51579900	-0.25698800
C	-5.44601800	0.19599100	2.23940700
H	-3.61688500	-0.76079500	2.80896600
C	-5.54622300	1.01186600	-0.01741300
H	-3.84767200	0.63074900	-1.24884600
C	-6.14380300	0.85867200	1.23059900
H	-5.89528900	0.05908000	3.21806100

H	-6.07953700	1.50836000	-0.82235900
H	-7.14339000	1.24008700	1.41202500
C	-2.95845900	-4.51775900	0.63395700
C	-2.55426200	-3.24092900	1.01155100
C	-2.51604000	-2.18260300	0.09589400
C	-3.01531100	-2.43083500	-1.18407700
C	-3.40741800	-3.70845800	-1.57989100
C	-3.35979600	-4.76793000	-0.67735800
H	-2.96571500	-5.31701000	1.36928400
H	-2.26474700	-3.06369400	2.04329800
H	-3.15805000	-1.60584500	-1.86527700
H	-3.78377800	-3.86469000	-2.58668200
H	-3.67125800	-5.76310800	-0.97758400
C	-2.15470200	-0.77322900	0.59607000
H	-1.83086400	-0.92374000	1.63253400
C	-2.03050600	2.11798900	4.84050800
C	-2.93674500	2.47626200	3.84555200
C	-2.63835400	2.23737100	2.50743000
C	-1.43412100	1.63755900	2.11678400
C	-0.51129400	1.33536700	3.12833400
C	-0.81205600	1.55346300	4.47280800
H	-2.26107400	2.29535600	5.88587100
H	-3.88518300	2.93515000	4.10665700
H	-3.36990900	2.50690900	1.74830000
H	0.48445700	0.97303800	2.89764100
H	-0.07429000	1.30327800	5.22939700
C	-1.14814600	1.54085800	0.60299700
H	-2.10654900	1.77790900	0.13547100
C	-0.87628500	0.06928300	-0.00616000
C	0.44804500	-4.38264400	-0.47384000
C	0.37407600	-2.99286000	-0.52353500
C	0.34121000	-2.21651500	0.64796000

C	0.34671300	-2.90344300	1.86894800
C	0.46555100	-4.28899100	1.93051600
C	0.52805300	-5.03656300	0.75450600
H	0.44856100	-4.95253900	-1.39746600
H	0.33585300	-2.51746900	-1.49669800
H	0.23221000	-2.34285800	2.79378500
H	0.48238300	-4.78691000	2.89507800
H	0.60663000	-6.11800400	0.79551800
C	0.38599700	-0.68667300	0.67276700
H	0.23731300	-0.45113500	1.72463200
C	3.52086800	-0.31569000	3.89551200
C	2.69002600	-0.62558200	2.81677700
C	2.70744100	0.14483900	1.65051700
C	3.60265700	1.22214900	1.58661300
C	4.42625100	1.54082400	2.66076900
C	4.38729800	0.77129200	3.82351600
H	3.48271100	-0.92524300	4.79256900
H	2.02519000	-1.47602300	2.90333400
H	3.65615900	1.81493200	0.67549100
H	5.10301300	2.38603200	2.58675300
H	5.02638000	1.01780800	4.66486900
C	1.89086700	-0.21135300	0.40584400
H	1.87770000	0.66962000	-0.24404200
C	5.62701500	0.30288700	-2.42927400
C	4.37680300	-0.18851500	-2.05912700
C	4.18936100	-0.72070400	-0.78589300
C	5.25960700	-0.79748400	0.11105000
C	6.51104500	-0.32781100	-0.27247100
C	6.69472200	0.23052100	-1.53861700
H	5.76313900	0.72575000	-3.41927200
H	3.54826800	-0.15401600	-2.75632100
H	5.12693200	-1.20078700	1.11285200

H	7.34166400	-0.39091000	0.42226600
H	7.67245800	0.59952100	-1.83017100
C	2.77983200	-1.17149600	-0.44737900
H	2.25226100	-1.48818300	-1.35272600
N	2.97976900	-2.47751400	0.28527000
N	3.33077700	-3.37720500	0.81264600

TS15-2

C	-2.97941200	-1.59673100	3.97492700
C	-2.05648800	-1.26756600	2.98567100
C	-1.83751600	0.05472900	2.59981500
C	-2.47109800	1.05298200	3.35200500
C	-3.39092500	0.73790500	4.34979700
C	-3.67535600	-0.59344700	4.64566400
H	-3.13907900	-2.63959000	4.23206200
H	-1.47656400	-2.05340900	2.53030700
H	-2.24052500	2.09858800	3.16799400
H	-3.87906700	1.53614800	4.89975300
H	-4.39989200	-0.84325000	5.41356200
C	-0.78227100	0.45246300	1.54802100
H	-0.93009300	1.52803600	1.43008100
C	0.85342000	1.43802900	3.17489600
C	0.50222000	0.32538700	2.39148700
C	1.15176500	-0.88584000	2.66946000
C	2.08498000	-0.98501600	3.70126600
C	2.37957000	0.11810800	4.50152500
C	1.76702400	1.33869900	4.22429500
H	0.33754500	2.38426300	3.02052000
H	0.89222700	-1.78387300	2.11913200
H	2.55358300	-1.94415800	3.89964400
H	3.07497700	0.02672900	5.32989200
H	1.97934300	2.21075800	4.83539400

C	-0.36972600	-2.79398200	0.35851100
C	-0.99597700	-3.74941400	1.17171200
C	0.91477200	-3.09719500	-0.09138100
C	-0.31907600	-4.88153700	1.62175200
H	-2.04086600	-3.62097700	1.44306100
C	1.60650900	-4.22082700	0.35281300
H	1.35928700	-2.47894900	-0.85410100
C	1.00080300	-5.10931200	1.23907900
H	-0.83285500	-5.59415700	2.25907000
H	2.60667200	-4.42107900	-0.02230600
H	1.53133400	-5.98877000	1.58847900
C	-3.53680600	-0.00537000	-0.73027700
C	-4.18855600	-0.11442100	-1.96581800
C	-4.24917800	-0.37396900	0.41385900
C	-5.48530700	-0.60475400	-2.06610000
H	-3.65709700	0.16441800	-2.87370100
C	-5.54447800	-0.88573400	0.31854500
H	-3.80962400	-0.27539500	1.39747800
C	-6.16905200	-1.00742900	-0.91960600
H	-5.95506200	-0.68591200	-3.04132800
H	-6.06534900	-1.17767300	1.22542400
H	-7.17791200	-1.40083200	-0.99020500
C	-2.87090600	4.28530500	-1.70214500
C	-2.48592800	2.94899100	-1.75658800
C	-2.47219400	2.14285400	-0.61261500
C	-2.97694200	2.70000200	0.56383300
C	-3.34878300	4.04096700	0.63646000
C	-3.27639900	4.85058000	-0.49451200
H	-2.85894300	4.88353400	-2.60853400
H	-2.19405800	2.52368400	-2.71216200
H	-3.14357100	2.06328800	1.41927400
H	-3.73043500	4.44089900	1.57139300

H	-3.57259000	5.89340100	-0.44647100
C	-2.14190700	0.64689900	-0.75205000
H	-1.82869200	0.53025800	-1.79596300
C	-2.21264500	-3.19889300	-4.13190200
C	-3.08721100	-3.29448900	-3.05219000
C	-2.74483800	-2.74022900	-1.82288400
C	-1.52627700	-2.07717600	-1.62634500
C	-0.63715600	-2.03720100	-2.70982000
C	-0.98241400	-2.57312200	-3.95079500
H	-2.47744000	-3.62236800	-5.09519000
H	-4.04565900	-3.79147000	-3.16460200
H	-3.45357900	-2.80586900	-0.99970400
H	0.36540300	-1.63578600	-2.60883400
H	-0.27023600	-2.51968300	-4.76881200
C	-1.19562600	-1.62071900	-0.18853500
H	-2.14495700	-1.70712600	0.34435300
C	-0.87458100	-0.05121800	0.03234700
C	0.54675900	4.34014600	-0.57860400
C	0.46258600	3.00308400	-0.19999100
C	0.39994000	1.97372300	-1.15443800
C	0.39910700	2.35268600	-2.50311100
C	0.52468300	3.68366400	-2.89240200
C	0.60686500	4.68605800	-1.92785200
H	0.57009500	5.11181200	0.18421600
H	0.43963000	2.77330100	0.85906900
H	0.26892900	1.59034100	-3.26809000
H	0.53006600	3.93904900	-3.94758000
H	0.68829500	5.72696900	-2.22327100
C	0.39850200	0.47951800	-0.82173600
H	0.22147000	0.00957600	-1.78662200
C	3.48676100	-0.69657800	-3.90994100
C	2.69611300	-0.11849700	-2.91490400

C	2.67137800	-0.64721100	-1.62195000
C	3.48219400	-1.75637500	-1.34530900
C	4.26635900	-2.34071400	-2.33441200
C	4.26955800	-1.81238600	-3.62525000
H	3.48548800	-0.27141500	-4.90839200
H	2.10324900	0.75329000	-3.16240100
H	3.50867300	-2.15825000	-0.33493100
H	4.87954100	-3.20359700	-2.09491600
H	4.87777900	-2.26574200	-4.40085800
C	1.89558700	0.01352900	-0.47985800
H	1.84180000	-0.72381100	0.33573300
C	5.73931400	0.59411900	2.45842800
C	4.47174400	0.88962900	1.96314900
C	4.19978200	0.75287600	0.59719300
C	5.23384500	0.40402500	-0.28914800
C	6.50422200	0.15043500	0.20733400
C	6.75421900	0.22432500	1.58139900
H	5.93296300	0.67721500	3.52254800
H	3.68489100	1.20783900	2.63785300
H	5.05913900	0.35970900	-1.35965900
H	7.30525600	-0.10363200	-0.47840200
H	7.74927000	0.01449900	1.96018300
C	2.79836000	1.02500300	0.20096900
H	2.27813800	1.65312800	0.92592400
N	3.07412500	2.33808000	-1.00824400
N	3.51096700	3.11903700	-1.64716100

IN21-2

C	-2.72926700	-2.91468100	3.35622200
C	-1.93971000	-2.29900600	2.38880900
C	-1.50527200	-0.97776300	2.52996800
C	-1.79355400	-0.33811700	3.74298700

C	-2.57784400	-0.94572800	4.72086700
C	-3.07248700	-2.23213200	4.52112400
H	-3.06771100	-3.93433800	3.20104500
H	-1.65022000	-2.86878900	1.52009300
H	-1.39724400	0.65587100	3.93340900
H	-2.79684300	-0.41220200	5.64020200
H	-3.69323800	-2.70592600	5.27414800
C	-0.56642300	-0.27079400	1.53067500
H	-0.53671000	0.76356400	1.88326700
C	1.50214500	-0.17507800	2.96293500
C	0.78371700	-0.86479400	1.96367400
C	1.16796300	-2.18967600	1.68937600
C	2.21184600	-2.78954100	2.37812300
C	2.90419400	-2.09013400	3.37503500
C	2.54132900	-0.77957700	3.66716700
H	1.19443200	0.83191600	3.23747300
H	0.63121900	-2.76557700	0.94500100
H	2.48036500	-3.81704200	2.15108600
H	3.70859300	-2.57194200	3.92200000
H	3.05725000	-0.22794400	4.44704400
C	-0.58718600	-2.55101100	-1.18123400
C	-0.83140200	-3.83093900	-0.66588700
C	0.33308900	-2.46114600	-2.23401700
C	-0.10926800	-4.94636600	-1.09031200
H	-1.62523900	-3.98235700	0.05802200
C	1.05959500	-3.56759100	-2.66725100
H	0.45317900	-1.51728800	-2.75901000
C	0.86040900	-4.81681800	-2.07969600
H	-0.32643400	-5.91935900	-0.66118300
H	1.75420300	-3.46612500	-3.49695800
H	1.41658300	-5.68227200	-2.42443900
C	-3.60979000	0.52534900	-0.20121200

C	-4.42295300	1.01027000	-1.23522000
C	-4.20887600	-0.26060400	0.78420900
C	-5.76978000	0.68046100	-1.31543000
H	-3.98318600	1.63542900	-2.00924900
C	-5.55933400	-0.60631400	0.70215100
H	-3.63855300	-0.61859200	1.63050100
C	-6.34464600	-0.14720100	-0.35060400
H	-6.36702700	1.05829700	-2.13946300
H	-5.99400100	-1.23111400	1.47680100
H	-7.39370500	-0.41790500	-0.41287300
C	-2.32884000	4.74933000	0.59722300
C	-2.19410500	3.53769300	-0.07320700
C	-2.20525300	2.31736300	0.61249500
C	-2.47216800	2.35470800	1.98263100
C	-2.58871300	3.56282900	2.66825700
C	-2.49623400	4.77085300	1.98086400
H	-2.30277400	5.67792500	0.03491400
H	-2.07042800	3.54235500	-1.15186900
H	-2.65177700	1.43059200	2.51351200
H	-2.78866600	3.55603800	3.73586400
H	-2.59422700	5.71379100	2.50897400
C	-2.15043300	1.01098900	-0.19223100
H	-1.98515000	1.34062300	-1.22268200
C	-4.02054800	-1.08509900	-4.32091500
C	-4.33362500	-1.91315800	-3.24366600
C	-3.50277200	-1.95085000	-2.13133200
C	-2.34392800	-1.16689100	-2.04907600
C	-2.02591900	-0.36798500	-3.15168100
C	-2.86074700	-0.31896200	-4.26979500
H	-4.66934100	-1.04169100	-5.18945700
H	-5.23485200	-2.51771700	-3.26073000
H	-3.77897400	-2.57687900	-1.28543500

H	-1.13366700	0.24841300	-3.17158800
H	-2.59479800	0.32321100	-5.10391900
C	-1.48545300	-1.36918700	-0.77977700
H	-2.20162500	-1.77462100	-0.06340800
C	-0.93793300	-0.06844000	-0.00831600
C	1.11010000	4.09298000	0.65791800
C	0.84374600	2.72724100	0.60506500
C	0.50047000	2.10177400	-0.60117600
C	0.43268900	2.89774000	-1.74972800
C	0.70698400	4.26313000	-1.70826200
C	1.05253200	4.86595700	-0.50140500
H	1.35308100	4.55521000	1.60986000
H	0.88075900	2.16059800	1.52962000
H	0.18105200	2.43579400	-2.70226600
H	0.65206400	4.85215200	-2.61839500
H	1.26354600	5.92968100	-0.45946200
C	0.31535900	0.59886900	-0.76092100
H	0.05646600	0.47396900	-1.81234000
C	4.07835000	2.10504900	-2.89072600
C	3.20933400	1.70767500	-1.87569400
C	2.65842800	0.41694100	-1.87185200
C	3.04684900	-0.47667500	-2.87779100
C	3.90335400	-0.07411400	-3.89774100
C	4.42068000	1.22173700	-3.90992100
H	4.48642700	3.11084800	-2.87714600
H	2.96202400	2.41886700	-1.09594500
H	2.68073600	-1.49703300	-2.86508500
H	4.17171000	-0.77601200	-4.68075900
H	5.09383400	1.53324000	-4.70171400
C	1.73483500	-0.09061000	-0.72796100
H	1.61022300	-1.16097700	-0.87917300
C	5.98023400	-0.86296100	1.75274800

C	4.80072800	-0.16792100	1.52953800
C	3.81377200	-0.68396100	0.65916100
C	4.02635400	-1.94582500	0.04975200
C	5.20308600	-2.63475600	0.27613800
C	6.18079200	-2.09084100	1.12058000
H	6.74016800	-0.45497900	2.40972300
H	4.62801600	0.79145600	2.00917300
H	3.26606100	-2.37674100	-0.59637000
H	5.36977000	-3.59589400	-0.19755400
H	7.10456100	-2.63612500	1.28856000
C	2.67371100	0.12212400	0.39615800
H	2.66320600	1.09550000	0.88503900
N	4.36621000	3.32068200	1.03526900
N	4.73716000	4.18006100	0.45991200

M06/6-311++G(d,p)

$PhCHN_2$'

C	1.48528500	1.41841900	0.00000300
C	0.16961700	0.98600700	0.00000000
C	-0.12697500	-0.38113800	0.00000000
C	0.93170500	-1.29557600	0.00000400
C	2.24436400	-0.85532600	0.00000800
C	2.53084100	0.50384300	0.00000700
H	1.69615200	2.48361500	0.00000300
H	-0.63452700	1.71754800	-0.00000300
H	0.71458000	-2.36078200	0.00000500
H	3.05191900	-1.58128100	0.00001100
H	3.56002000	0.84762300	0.00001000
C	-1.48600500	-0.88797200	-0.00000300
H	-1.72042200	-1.94529700	-0.00000300

N	-2.50459300	-0.08546800	-0.00000800
N	-3.37551000	0.64390100	-0.00001200

$Ni(acac)_2$

H	3.00853200	-3.09249600	-0.87875700
H	3.00842900	-3.09235100	0.87940800
H	4.36826400	-2.34301100	0.00035200
C	3.19365400	0.00000100	0.00028900
H	4.27697600	-0.00000100	0.00050400
C	3.28914400	-2.50485500	0.00029200
C	2.51783100	-1.22098200	0.00014300
O	1.25979200	-1.35914300	-0.00006300
O	1.25979000	1.35914100	-0.00006600
C	3.28913900	2.50485700	0.00046600
H	4.36825900	2.34302000	0.00099300
H	3.00804100	3.09252200	0.87934200
H	3.00890300	3.09232600	-0.87882300
C	2.51783400	1.22098100	0.00022600
H	-3.00735900	-3.09288000	0.87897500
H	-3.00958800	-3.09197200	-0.87918900
H	-4.36825700	-2.34301900	0.00197100
C	-3.19365300	-0.00000200	0.00096700
H	-4.27697500	-0.00000400	0.00188100
C	-3.28913800	-2.50485800	0.00055700
C	-2.51783000	-1.22098400	0.00023600
O	-1.25979000	-1.35914100	-0.00093600
O	-1.25979200	1.35914300	-0.00085000
C	-3.28914400	2.50485400	0.00029900
H	-4.36826400	2.34301300	0.00075500
H	-3.00882200	3.09233500	-0.87894800

H	-3.00813600	3.09250800	0.87921700
C	-2.51783400	1.22098000	0.00013200
Ni	-0.00000100	0.00000200	-0.00050000

IN1’

H	-1.83140900	-1.20270100	3.78988300
H	-2.12058500	-2.18364700	2.35689500
H	-3.34043200	-0.98234800	2.86577300
C	-2.23189700	0.95964500	1.50232600
H	-3.27966100	1.07635300	1.75215500
C	-2.27151000	-1.18941300	2.78754700
C	-1.55670400	-0.17566400	1.94861500
O	-0.34047500	-0.44377500	1.71423400
O	-0.41005900	1.90694200	0.32712600
C	-2.39231000	3.13489200	0.27478800
H	-3.45772000	3.04345400	0.49448300
H	-2.24841000	3.28673100	-0.79881100
H	-1.99697000	4.02209500	0.78034300
C	-1.61656900	1.93194300	0.71555100
H	3.39331600	-2.99106300	0.65688300
H	4.03549600	-2.12811600	2.05011800
H	4.96499400	-2.15298100	0.52582900
C	3.78767900	0.01855800	-0.36216900
H	4.78543500	-0.18030400	-0.73484000
C	3.96440800	-2.10709200	0.95879000
C	3.21183900	-0.88460900	0.53677000
O	2.06113100	-0.76676200	1.04755700
O	1.98465500	1.55028600	-0.38967600
C	3.85030500	2.16996400	-1.64458300
H	4.82268300	1.80346100	-1.97753800

H	3.98551200	3.11529900	-1.10993200
H	3.22170100	2.38464100	-2.51370600
C	3.14789900	1.19780100	-0.74688400
Ni	0.82159600	0.56681300	0.68070600
C	-3.56127900	-1.74201500	-0.62544900
C	-2.19673700	-1.86191300	-0.83139900
C	-1.49647100	-0.87615800	-1.53443700
C	-2.20214200	0.23362800	-2.01130800
C	-3.56616500	0.34581000	-1.80196000
C	-4.25644000	-0.64069500	-1.10897100
H	-4.08759400	-2.51806200	-0.07639800
H	-1.66530400	-2.72369800	-0.43364800
H	-1.66361300	1.01342000	-2.54498100
H	-4.09611300	1.21457800	-2.18291600
H	-5.32598800	-0.55243300	-0.94685100
C	-0.06789200	-0.94788000	-1.77233500
H	0.48995000	-0.15317400	-2.25462500
N	0.61981600	-1.99756800	-1.44105300
N	1.20474200	-2.92194900	-1.13911300

TS1’

H	1.13264100	-2.84597600	2.74035800
H	2.46110200	-1.70487000	2.54570300
H	2.51852000	-3.26898500	1.69548700
C	1.41813800	-2.21494200	-0.41640400
H	2.13095500	-3.02377700	-0.53463400
C	1.86351800	-2.46021100	2.02344800
C	1.14441100	-1.80474600	0.88176100
O	0.33776200	-0.88886100	1.25265300
O	0.08756600	-0.67834900	-1.63524500

C	1.42439300	-2.12635900	-2.91426700
H	2.17309400	-1.41674200	-3.28693000
H	0.61075200	-2.15418900	-3.64302100
H	1.89357700	-3.10980000	-2.83854800
C	0.90408900	-1.62106000	-1.59438800
H	-4.28851900	-1.71944800	2.41358100
H	-4.05484800	-2.87514400	1.10926100
H	-5.41881900	-1.72757700	1.03396400
C	-3.99192100	0.04816500	-0.25793500
H	-5.05983400	0.04027900	-0.44082600
C	-4.37633500	-1.85329700	1.33124600
C	-3.45820400	-0.88898700	0.64125300
O	-2.24076800	-0.99690300	0.94036100
O	-1.96159200	1.15322400	-0.77907400
C	-3.87297900	2.00943300	-1.80395300
H	-4.95253400	1.86702200	-1.87159500
H	-3.43302000	1.92660600	-2.80248700
H	-3.66410100	3.02161600	-1.44402900
C	-3.22078300	1.01303600	-0.89610900
Ni	-0.82665600	0.03561400	0.16838800
C	4.09451900	0.72269500	1.12686700
C	2.77120100	1.12794500	1.20155600
C	2.03572200	1.34381900	0.03650300
C	2.64853200	1.14324100	-1.20082600
C	3.97019400	0.73388300	-1.26884800
C	4.69976500	0.52066700	-0.10661000
H	4.65589700	0.55700600	2.04178000
H	2.30148700	1.25528500	2.17407200
H	2.07065800	1.29255900	-2.10909300
H	4.43404200	0.57873900	-2.23869600

H	5.73506100	0.19946800	-0.16121900
C	0.62025600	1.73903800	0.05354200
H	0.14177500	2.09085600	-0.85807900
N	0.17759600	2.37825100	1.13530500
N	-0.18793100	2.86647200	2.08033700

IN2’

H	1.93939700	-3.05067100	-2.60449800
H	1.07684300	-4.13685900	-1.52329300
H	2.84983400	-4.00335600	-1.40157900
C	2.71383100	-2.05728500	0.31889000
H	3.60509900	-2.67286700	0.25284400
C	1.92714500	-3.45016500	-1.58523300
C	1.72594300	-2.31846100	-0.61772900
O	0.60972500	-1.71309300	-0.78297200
O	1.72121200	-0.35673600	1.64821600
C	3.92078500	-0.97231900	2.22359500
H	4.61784800	-1.80408900	2.09840300
H	3.63432600	-0.88191000	3.27471900
H	4.43596400	-0.04458500	1.95026600
C	2.67827800	-1.09610300	1.37309100
H	2.52364300	1.74749000	-3.59893200
H	3.74503200	1.54137400	-2.35067200
H	3.11500600	3.17313400	-2.70622200
C	1.20785800	2.94574700	-0.92569900
H	1.57239800	3.94391400	-1.13711700
C	2.85532800	2.11646700	-2.62365800
C	1.77899300	1.86462500	-1.61169600
O	1.45942400	0.65406100	-1.46130800
O	-0.35240600	1.69532200	0.33918400

C	-0.41208300	4.00121700	0.66609100
H	0.06501600	4.93112600	0.35294400
H	-0.31169300	3.88933600	1.75065000
H	-1.48302700	4.05182100	0.44463300
C	0.17524300	2.79859200	-0.00513300
Ni	0.21853400	0.01630800	-0.19991400
C	-4.04501900	-1.70622300	-1.22191900
C	-2.83798100	-1.74577700	-0.54422600
C	-2.51290000	-0.74146600	0.36841500
C	-3.40880400	0.30415400	0.58335900
C	-4.61715700	0.33419400	-0.09616300
C	-4.94120400	-0.66867000	-0.99946300
H	-4.28689900	-2.49164300	-1.93133100
H	-2.12367900	-2.54290200	-0.73078400
H	-3.15969700	1.10581100	1.27382200
H	-5.31028100	1.15090000	0.08115200
H	-5.88763700	-0.64054400	-1.52990200
C	-1.18764700	-0.82075500	1.02460300
N	-1.05080000	-0.10931500	2.17333000
N	-0.89515800	0.55523400	3.05031500
H	-0.74203200	-1.80658100	1.15925700

TS2’

H	1.81085500	-3.25742600	-2.68625200
H	0.56452400	-4.13286000	-1.80807900
H	2.27865900	-4.38126700	-1.38327600
C	2.26773000	-2.45111600	0.35296400
H	2.99890500	-3.25324600	0.40399400
C	1.53798000	-3.64443800	-1.69923300
C	1.41416200	-2.49348000	-0.73863100

O	0.49382800	-1.67173300	-1.07713400
O	1.56868000	-0.53094900	1.57664300
C	3.47708800	-1.67708000	2.38217800
H	3.53230600	-2.71286700	2.73204900
H	3.35378200	-1.00459600	3.23264800
H	4.42973000	-1.45371100	1.88838100
C	2.33799400	-1.48521800	1.40458400
H	3.41120500	1.67228200	-3.12609500
H	4.31432500	1.04514400	-1.75397400
H	4.09074600	2.80743100	-1.93085900
C	1.87169700	2.78234400	-0.53757000
H	2.45858300	3.69308200	-0.51994200
C	3.61999300	1.83180800	-2.06380600
C	2.34508700	1.69331900	-1.28741400
O	1.77275600	0.57745300	-1.38261900
O	-0.14205000	1.77783000	0.20868300
C	0.19053700	3.99090400	0.86334600
H	0.88616900	4.82611000	0.76755700
H	0.05925100	3.75173000	1.92399200
H	-0.78919000	4.29102000	0.47805300
C	0.65891300	2.76508800	0.13963000
Ni	0.24855800	0.04478900	-0.38739200
C	-4.87520200	-1.37505200	-0.29588200
C	-3.57431500	-1.54857100	0.14944200
C	-2.66718400	-0.48597200	0.11874600
C	-3.08999500	0.76162300	-0.35321300
C	-4.39642500	0.93888600	-0.77277800
C	-5.28716600	-0.12934200	-0.75016900
H	-5.57036300	-2.20847700	-0.28357100
H	-3.24474900	-2.51765400	0.51517300

H	-2.37134800	1.57589100	-0.38039000
H	-4.72321900	1.90894600	-1.13399200
H	-6.30814500	0.01027600	-1.09265800
C	-1.26685300	-0.70470200	0.49827400
N	-1.11460300	0.05726200	2.11689300
N	-0.88723900	0.79571400	2.89524200
H	-1.05870700	-1.71954500	0.85187700

IN3'

H	3.07090100	-2.86457600	-2.56641300
H	1.78891600	-3.96129900	-2.07033100
H	3.34642200	-3.97697900	-1.20111100
C	2.55929700	-2.18325800	0.50022100
H	3.33818000	-2.89728000	0.75290500
C	2.60969500	-3.34160400	-1.69582000
C	2.05884300	-2.27605500	-0.78788600
O	1.15905300	-1.56956200	-1.36120400
O	1.35438800	-0.36817300	1.46110600
C	3.04119800	-1.35454000	2.79371400
H	3.04435200	-2.38239500	3.17096400
H	2.63928100	-0.68306500	3.55393800
H	4.08343300	-1.08343600	2.59134100
C	2.22461800	-1.24436700	1.52487700
H	3.69524600	2.50918200	-2.33617800
H	4.35359100	1.90581700	-0.82157100
H	3.79673100	3.59852300	-0.92818400
C	1.36792600	2.96695300	-0.17387500
H	1.71841600	3.96426400	0.06451900
C	3.59933200	2.57379600	-1.24795500
C	2.23719100	2.09284700	-0.84598100

O	1.96385800	0.90773800	-1.16447200
O	-0.51034700	1.51738400	-0.07400700
C	-0.82753400	3.64940200	0.80642600
H	-0.35393800	4.62923300	0.88749400
H	-1.09130700	3.29350000	1.80756000
H	-1.76109400	3.73981200	0.24185600
C	0.05774600	2.63465800	0.14875300
Ni	0.37318300	-0.03016400	-0.65619100
C	-4.75523200	-1.53341000	0.07876000
C	-3.39463400	-1.74741300	0.15108000
C	-2.48637100	-0.81289600	-0.39147600
C	-2.99634600	0.33810300	-1.02772100
C	-4.35937600	0.53903500	-1.11342900
C	-5.23392100	-0.39030800	-0.55679500
H	-5.44888400	-2.25045800	0.50503700
H	-2.99850900	-2.63349100	0.63999100
H	-2.29169200	1.04962200	-1.44205200
H	-4.75162800	1.41936700	-1.61187800
H	-6.30544500	-0.22441400	-0.62319700
C	-1.09010500	-1.05037200	-0.29835800
N	-1.40709600	-0.33211600	2.67715800
N	-1.29132300	0.71412600	2.97678300
H	-0.84271900	-2.04393500	0.10246300

IN4’

H	3.29424000	-2.96244700	-1.90461500
H	1.89540500	-4.02348400	-1.79695500
H	3.14378700	-4.08236400	-0.52514300
C	1.94604700	-2.27641200	0.90232500
H	2.59094600	-3.01899200	1.36135700

C	2.59416900	-3.42648700	-1.20254900
C	1.83794900	-2.34679900	-0.47948500
O	1.15393900	-1.61304900	-1.27250700
O	0.49840200	-0.47823600	1.47086500
C	1.49621700	-1.62469700	3.27046600
H	2.42467600	-2.16059400	3.48264200
H	0.66387100	-2.21987600	3.66341500
H	1.48972200	-0.66606200	3.79459200
C	1.27288700	-1.39217500	1.79362900
H	4.39778600	2.08078600	-1.04758500
H	4.35773300	1.38722700	0.56780800
H	4.11446900	3.14349400	0.35567500
C	1.51646400	2.87309800	0.11774100
H	1.88141900	3.81942700	0.49939800
C	3.91385700	2.15824700	-0.06910200
C	2.44869100	1.87975200	-0.22230900
O	2.15425100	0.74512900	-0.67571400
O	-0.44108100	1.68393900	-0.51364600
C	-0.77675900	3.86532100	0.21730000
H	-0.25848100	4.72519200	0.64504900
H	-1.55509000	3.52577600	0.90888900
H	-1.28085600	4.17050700	-0.70510500
C	0.14866100	2.72360900	-0.07878900
Ni	0.38064100	0.03239300	-0.81930800
C	-3.79684900	0.28026600	1.31233000
C	-2.56581800	0.11860800	0.70735400
C	-2.47020100	-0.56915900	-0.52311900
C	-3.64490600	-1.09755300	-1.10347200
C	-4.87331200	-0.90409700	-0.50829800
C	-4.94469400	-0.21535800	0.70103200

H	-3.87098600	0.79229200	2.26623200
H	-1.65215300	0.47043200	1.17337000
H	-3.56395600	-1.64664200	-2.03795300
H	-5.77553100	-1.29568600	-0.96636300
H	-5.91011200	-0.07486500	1.17907700
C	-1.22453900	-0.73174600	-1.17616200
H	-1.23612100	-1.45990500	-1.99849100

TS3’

H	3.72143400	-2.36614100	-1.82496500
H	2.46801300	-3.50842400	-2.29191600
H	3.44861200	-3.82686300	-0.83680300
C	1.81306800	-2.52779500	0.71323000
H	2.49322500	-3.26337800	1.12974500
C	2.96299400	-3.04997300	-1.43017400
C	1.95559200	-2.26117400	-0.64225800
O	1.32397500	-1.40809100	-1.35068600
O	-0.06216300	-1.18950600	1.28651500
C	0.92233200	-2.39099700	3.04940700
H	1.73196600	-3.09351000	3.25936000
H	-0.03240900	-2.83973600	3.34054500
H	1.05451700	-1.49587600	3.66533400
C	0.85046100	-1.97455900	1.59858800
H	4.54436900	2.04193100	-0.42506700
H	4.26523100	1.44095200	1.20326800
H	4.12257200	3.18939400	0.87297200
C	1.56728500	2.98846300	0.31917400
H	1.91592600	3.94255600	0.69669800
C	3.93969700	2.18969000	0.47498700
C	2.49785400	1.95384200	0.14094400

O	2.22189600	0.80640200	-0.29793400
O	-0.34485800	1.79782400	-0.42802100
C	-0.71471100	4.00862500	0.19570200
H	-0.22588600	4.87377400	0.64679600
H	-1.55897400	3.69771800	0.82012100
H	-1.12793200	4.29486200	-0.77663400
C	0.21765800	2.84945100	0.01309800
Ni	0.48703000	0.16042100	-0.74957400
C	-3.91944400	0.49691500	1.07135800
C	-2.63467000	0.33060900	0.59549100
C	-2.39557700	-0.49448000	-0.52295800
C	-3.48815700	-1.15635000	-1.12190800
C	-4.77494600	-0.95796900	-0.66353400
C	-4.98689400	-0.13213000	0.43623100
H	-4.10014100	1.11703700	1.94350900
H	-1.78784400	0.79416800	1.08339100
H	-3.30020400	-1.81652600	-1.96458400
H	-5.61195900	-1.45533000	-1.14238000
H	-5.99604800	0.01232400	0.81159500
C	-1.10105200	-0.66867200	-1.07956700
H	-1.04106200	-1.50473600	-1.78876500

IN5’

H	4.86680400	-1.48233900	-0.65695500
H	4.15031000	-1.12231600	-2.24107500
H	4.15037000	-2.79382700	-1.59415400
C	2.38254800	-2.32745600	0.39878200
H	3.20128200	-2.96258300	0.72639200
C	4.05099000	-1.73362600	-1.34415400
C	2.73602100	-1.45345000	-0.67723800

O	2.11352300	-0.45337900	-1.08098700
O	0.04818300	-1.98990600	0.91554000
C	1.24521500	-3.29561500	2.38898000
H	2.23611100	-3.69120200	2.61217300
H	0.53553300	-4.12324300	2.29424800
H	0.90968200	-2.68102100	3.23035000
C	1.23993400	-2.46914800	1.14717200
H	2.59772300	4.33498800	-1.04313600
H	3.13677400	4.01029400	0.59867500
H	1.84992500	5.21189600	0.31851800
C	-0.02097300	3.39939100	0.59335100
H	-0.27060400	4.41557200	0.87605800
C	2.25765800	4.25285700	-0.00635700
C	1.26341400	3.13444800	0.09890500
O	1.67514000	2.00355200	-0.27787700
O	-0.84534700	1.18101400	0.46637400
C	-2.34658900	2.75576500	1.28072100
H	-2.44054200	3.81052200	1.54556000
H	-2.55842900	2.13523500	2.15716500
H	-3.09783800	2.49894800	0.52598900
C	-0.99029900	2.40957700	0.74718200
Ni	0.64328600	0.39079100	-0.29405000
C	-0.27583000	-1.31639600	-0.31883700
C	-1.74457300	-1.22023000	-0.44786100
C	-2.61312400	-1.27494400	0.64077500
C	-2.27879800	-0.99124900	-1.71665800
C	-3.97867900	-1.11010500	0.46114900
H	-2.20960100	-1.43504300	1.63513200
C	-3.64150500	-0.81712100	-1.89447400
H	-1.60714600	-0.93671000	-2.57161000

C	-4.50105600	-0.87702300	-0.80395600
H	-4.64215300	-1.15846900	1.32034400
H	-4.03701300	-0.63887900	-2.89033200
H	-5.57019500	-0.74596100	-0.94095100
H	0.14887900	-1.91231200	-1.13828000

TS4'

H	-3.24808000	4.18541500	-0.03941300
H	-3.44308300	3.11992400	1.34690500
H	-4.67710700	3.12344400	0.06372800
C	-3.46975800	1.22869700	-1.22765700
H	-4.52258000	1.46617100	-1.35960900
C	-3.60898700	3.19894200	0.26753600
C	-2.82386100	2.12839500	-0.42010300
O	-1.55503100	2.27564600	-0.13678500
O	-2.04077000	-0.62555100	-1.74713300
C	-4.10345400	-0.51082100	-2.88006600
H	-4.39815800	0.25982900	-3.59951800
H	-5.00812500	-0.80547700	-2.33648800
H	-3.70567400	-1.37986400	-3.40465900
C	-3.07766400	0.01960200	-1.91043600
H	-0.59014800	-4.56543800	1.36227300
H	-2.32277100	-4.28332400	1.44697900
H	-1.37212800	-4.33003500	2.95227800
C	-1.09461000	-1.71936100	2.87336400
H	-1.20081500	-2.17632800	3.85067100
C	-1.36539100	-4.01692100	1.90650400
C	-1.13926600	-2.54288200	1.74219100
O	-1.00852200	-2.14613600	0.55209200
O	-0.86256700	0.35098900	1.73888500

C	-0.97494300	0.48316400	4.06278800
H	-1.09739700	-0.12845200	4.95863500
H	-1.77874000	1.22475300	4.01726300
H	-0.03222900	1.03700400	4.12969200
C	-0.97396200	-0.32699800	2.79847600
Ni	-0.69248900	-0.34235300	-0.03201700
C	3.09687800	2.58803000	-0.85475400
C	1.84530700	2.11114400	-1.21419900
C	0.79412700	2.07378200	-0.29704300
C	1.02410400	2.56892700	0.99094200
C	2.27059200	3.05873500	1.34529900
C	3.31729300	3.06140900	0.43024800
H	3.90154100	2.59387900	-1.58485100
H	1.67821100	1.76023000	-2.23145400
H	0.21427700	2.54899400	1.71182100
H	2.43074500	3.44038100	2.35010600
H	4.29445400	3.43969800	0.71508100
C	-0.49834300	1.46514800	-0.68654000
H	-0.62424800	1.45480700	-1.77833000
C	3.74657000	-1.23936000	0.86336200
C	2.44909700	-1.28693700	0.37390900
C	2.20485600	-1.10603000	-0.98496600
C	3.27947700	-0.87914100	-1.84312300
C	4.57512800	-0.82515100	-1.35294500
C	4.81093600	-1.00574700	0.00330900
H	3.92503200	-1.37198500	1.92602400
H	1.61026500	-1.43997700	1.04865100
H	3.09184500	-0.73446100	-2.90488300
H	5.40300600	-0.63999000	-2.03097500
H	5.82410000	-0.95867400	0.39079100

C	0.80957300	-1.11985700	-1.52738900
N	0.33762100	-2.35081500	-1.78264400
N	-0.10655500	-3.37243400	-1.89379200
H	0.59295700	-0.47207700	-2.37790100

IN6’

H	-3.99787800	0.96858700	-2.92830900
H	-4.02849200	0.98581100	-1.16609500
H	-4.74517100	-0.39151700	-2.06021900
C	-2.47135200	-1.68128500	-1.96512500
H	-3.40229500	-2.21911200	-1.79952300
C	-3.92899300	0.33191400	-2.03991500
C	-2.58998500	-0.32571800	-2.01001100
O	-1.64331400	0.58748300	-2.01224300
O	-0.28233500	-2.14752500	-2.75529700
C	-1.44612900	-3.94105700	-1.72272500
H	-2.21402000	-4.45860500	-2.30889100
H	-1.77016200	-3.98000700	-0.67616600
H	-0.49807000	-4.46867900	-1.84832100
C	-1.31082600	-2.51035700	-2.19389100
H	0.52969400	-0.60202600	4.78054700
H	-0.52896800	-1.98780300	4.54466500
H	-1.15284800	-0.52722000	5.35864300
C	-1.93641700	0.54334800	3.09463100
H	-2.45107100	0.85741000	3.99528100
C	-0.50311900	-0.89333500	4.56175300
C	-0.88795900	-0.36915800	3.21045300
O	-0.20270300	-0.81477500	2.24303900
O	-1.87512600	0.79454000	0.73603400
C	-3.53276300	2.00623900	1.83695800

H	-3.86218800	2.30312000	2.83426000
H	-4.37122000	1.53292200	1.31441800
H	-3.25282700	2.89514400	1.26241200
C	-2.37015300	1.05853200	1.86770600
Ni	-0.36719900	-0.28615800	0.39007900
C	2.06118900	3.34458300	-0.94950100
C	1.55084600	2.08311600	-1.21493000
C	0.17348700	1.84420100	-1.21715600
C	-0.67883800	2.92795700	-0.97477200
C	-0.16803000	4.19039000	-0.72052900
C	1.20457600	4.40703700	-0.69646200
H	3.13697000	3.49808800	-0.95480500
H	2.23396100	1.26817000	-1.43508000
H	-1.75105700	2.76520600	-0.99274400
H	-0.84921400	5.01817300	-0.54161900
H	1.60110400	5.39766600	-0.49503200
C	-0.34124500	0.46579800	-1.42456200
H	0.29407000	-0.12504900	-2.09259700
C	4.22424500	0.22067600	1.11945800
C	2.97120200	-0.37157000	1.11954500
C	2.49959900	-0.99665400	-0.03234600
C	3.29108600	-1.02872100	-1.17756600
C	4.54032700	-0.42358400	-1.17559300
C	5.00844200	0.20133600	-0.02796300
H	4.58615900	0.71465200	2.01586600
H	2.33495600	-0.33164200	2.00058900
H	2.91217300	-1.51190800	-2.07563600
H	5.14871200	-0.44096700	-2.07455700
H	5.98505200	0.67539400	-0.02675700
C	1.12734800	-1.59223500	-0.09026700

N	0.98568400	-2.62317500	0.80790500
N	0.82115600	-3.39614500	1.58724500
H	0.85493400	-1.99464600	-1.07399500

TS5'

H	-3.25219400	2.36787000	-2.98518400
H	-3.58874000	1.94204400	-1.30849000
H	-4.30177900	0.97599700	-2.63946700
C	-2.25269300	-0.64851100	-2.62818200
H	-3.25618100	-1.04797600	-2.75856400
C	-3.41908500	1.52419300	-2.30701900
C	-2.19904000	0.66567800	-2.28205600
O	-1.16676200	1.37078900	-1.86722200
O	-0.04560400	-1.24801500	-3.26429500
C	-1.54919900	-3.03384300	-2.83209900
H	-2.37876500	-3.26789000	-3.50820000
H	-1.89469500	-3.26798700	-1.81762100
H	-0.69053300	-3.65732900	-3.08952800
C	-1.17746700	-1.57147000	-2.92666700
H	-0.74948400	-2.16877000	4.38882500
H	-1.86967200	-3.18681800	3.49226500
H	-2.50334300	-1.96583900	4.62804200
C	-2.61642000	-0.13382000	2.75413700
H	-3.31327200	-0.03890500	3.57882200
C	-1.72536300	-2.17784700	3.89244700
C	-1.71362900	-1.19446700	2.75967800
O	-0.84985100	-1.42100500	1.86046200
O	-1.93393600	0.81807600	0.69552900
C	-3.72899900	1.87255700	1.75550100
H	-4.20725700	1.95302300	2.73351200

H	-4.50034000	1.65126800	1.00909800
H	-3.28003700	2.83272000	1.48393300
C	-2.68047800	0.79921200	1.71156300
Ni	-0.49247700	-0.36005000	0.28802800
C	2.74814400	3.13552500	0.03849400
C	2.08343100	2.08611900	-0.57883000
C	0.68859800	2.01760500	-0.58766900
C	-0.02216000	3.06462300	0.00984700
C	0.64157900	4.11943500	0.61478000
C	2.03077900	4.15746500	0.64355400
H	3.83469100	3.15958800	0.02996500
H	2.65849900	1.31083400	-1.07759300
H	-1.10652100	3.03721000	-0.01185500
H	0.06932200	4.92418500	1.06841500
H	2.54771600	4.98490600	1.12046500
C	-0.00135900	0.85300500	-1.20907400
H	0.62748600	0.36716500	-1.96305800
C	4.02579900	-0.35946900	1.48284900
C	2.71057200	-0.70558300	1.22608100
C	2.34032600	-1.16895700	-0.03786600
C	3.30436100	-1.27358700	-1.04175800
C	4.61688500	-0.90167400	-0.79090600
C	4.97729000	-0.45046400	0.47235700
H	4.30986600	0.00394700	2.46531300
H	1.94575200	-0.60464600	1.99285300
H	3.00485300	-1.62083300	-2.02841100
H	5.36003100	-0.96596600	-1.57948500
H	6.00560600	-0.16350400	0.67094700
C	0.92507200	-1.46123100	-0.34818700
N	0.68572200	-3.00369400	0.41999500

N	0.37776600	-3.72450400	1.19051300
H	0.80151400	-1.82123000	-1.37497900

IN7’

H	-3.43554100	3.36628400	-1.87000800
H	-3.52657700	2.66065800	-0.25682200
H	-4.53969800	2.00538300	-1.58118000
C	-2.68462400	0.27871500	-2.22358500
H	-3.73051100	-0.02119400	-2.23863200
C	-3.56537200	2.42462600	-1.32573300
C	-2.45163000	1.49536000	-1.67142000
O	-1.29651300	2.03516900	-1.31109400
O	-0.72609500	-0.37456700	-3.39680700
C	-2.20096600	-2.11879100	-2.74751400
H	-3.20685400	-2.24660000	-3.16209800
H	-2.24753900	-2.42934900	-1.69605900
H	-1.49859900	-2.75461600	-3.29036300
C	-1.76392700	-0.67568000	-2.82619800
H	-0.44079700	-3.38325900	3.28532200
H	-1.88141200	-3.89179300	2.41654700
H	-2.05548600	-3.06677900	3.98364400
C	-2.34777700	-0.78374900	2.73446400
H	-2.96564500	-0.95464600	3.60862700
C	-1.47442700	-3.10473600	3.06026800
C	-1.49194000	-1.80325600	2.31420100
O	-0.70574600	-1.74106800	1.32319300
O	-1.80988800	0.77681900	1.03935600
C	-3.43489200	1.46453200	2.57200400
H	-3.81990500	1.22746400	3.56566800
H	-4.27952700	1.52562400	1.87627600

H	-2.95580600	2.44861800	2.58572400
C	-2.45880900	0.44142800	2.06804400
Ni	-0.46279700	-0.24923700	0.12853400
C	3.15085700	2.94130100	-0.09273800
C	2.20596900	2.20589500	-0.79424200
C	0.87157900	2.17224100	-0.38613000
C	0.49887500	2.92690600	0.72954100
C	1.44138100	3.66636100	1.42629100
C	2.77364700	3.66991100	1.02641000
H	4.18410400	2.95156900	-0.42899200
H	2.50494100	1.64850900	-1.67997600
H	-0.54042500	2.91272500	1.04551900
H	1.13591500	4.24734200	2.29216400
H	3.50845000	4.25032500	1.57639000
C	-0.09436500	1.29678500	-1.10111800
H	0.28981800	0.98905100	-2.07881300
C	3.91376800	-0.62398200	1.31500200
C	2.59031500	-0.62231500	0.92399700
C	2.22695900	-1.05025200	-0.36834300
C	3.23570200	-1.48858500	-1.24828300
C	4.56017200	-1.48116100	-0.85768300
C	4.89497000	-1.04626400	0.42230300
H	4.19153200	-0.28328200	2.30685400
H	1.80776800	-0.27516600	1.59395900
H	2.95039600	-1.81938600	-2.24366600
H	5.33675800	-1.80954600	-1.54068000
H	5.93734600	-1.03676500	0.72803200
C	0.87161100	-0.97596000	-0.80257300
N	0.43559800	-3.95030800	-0.73604500
N	-0.11697500	-4.44793400	0.06725300

H	0.70871800	-1.32164500	-1.83381500

IN8’

H	3.28920500	-3.64428000	-0.87060700
H	3.42254700	-2.49853500	0.46240900
H	4.44742600	-2.30377400	-0.99430000
C	2.65539800	-0.78663800	-2.13942600
H	3.71405000	-0.56212800	-2.24953600
C	3.46026300	-2.58979000	-0.62826300
C	2.37887800	-1.76146000	-1.23589800
O	1.21133000	-2.11572900	-0.72339800
O	0.69997800	-0.42702800	-3.44093500
C	2.29436700	1.32901400	-3.42504900
H	3.34852400	1.28943000	-3.71682800
H	2.22664400	2.00575100	-2.56334200
H	1.69656800	1.72959500	-4.24588200
C	1.77526200	-0.02850800	-3.01666000
H	0.57253000	4.38086800	2.25307400
H	1.83882300	4.65235800	1.06413500
H	2.28235300	4.31127900	2.75645700
C	2.42874200	1.74222400	2.28366400
H	3.07875100	2.14686500	3.05100200
C	1.57966300	4.07299900	1.95581900
C	1.57028100	2.61307600	1.60940900
O	0.75238700	2.28325600	0.70185300
O	1.80741500	-0.23424900	1.14047600
C	3.46844800	-0.48241300	2.76585400
H	3.89946600	0.03080200	3.62759900
H	4.28142600	-0.78043900	2.09401600
H	2.96822500	-1.39923600	3.09371800

C	2.49579900	0.37267700	2.00671500
Ni	0.46768900	0.51938200	-0.01411800
C	-3.24994800	-2.50929200	0.66807800
C	-2.28442500	-2.03587800	-0.20944600
C	-0.95375300	-1.89994700	0.18915700
C	-0.60663100	-2.28408300	1.48734000
C	-1.56963500	-2.76207900	2.36167400
C	-2.89775100	-2.86799100	1.96119700
H	-4.27974800	-2.60637500	0.33507500
H	-2.56401200	-1.77078800	-1.22728400
H	0.42966500	-2.18985000	1.79966300
H	-1.28398600	-3.05563800	3.36805400
H	-3.64875800	-3.24252600	2.65041500
C	0.03837600	-1.30275200	-0.74427200
H	-0.33985800	-1.28173400	-1.77087200
C	-3.92461100	1.29846500	0.93277500
C	-2.59449200	1.17399900	0.58854700
C	-2.20154800	1.19777400	-0.76501800
C	-3.18976500	1.35334300	-1.75700500
C	-4.52208100	1.46268400	-1.41034700
C	-4.88481500	1.43443900	-0.06631800
H	-4.22470000	1.27245400	1.97500100
H	-1.82760200	1.04156100	1.34740800
H	-2.88408200	1.36565700	-2.79994200
H	-5.28275400	1.56939900	-2.17674700
H	-5.93300800	1.51896200	0.20619100
C	-0.83990400	0.99061600	-1.12727300
H	-0.64858400	1.04475200	-2.20907900

TS6’

H	-2.25156800	2.91572400	2.65404100
H	-2.84737300	2.28103900	1.12431100
H	-2.44602700	4.02332000	1.27328500
C	-0.01899200	3.54144100	0.42350900
H	-0.56623400	4.41249500	0.07101600
C	-2.16156200	3.01233200	1.56690100
C	-0.76587200	2.67811600	1.16225200
O	-0.44925800	1.47562800	1.62496100
O	2.24439700	2.85117900	0.67114700
C	1.82223500	4.55212000	-0.92542500
H	1.44347300	5.55366900	-0.69729700
H	1.39338000	4.26460400	-1.89368700
H	2.91005600	4.57940000	-1.00301400
C	1.40489800	3.55511300	0.12851300
H	-2.41636100	-2.28045900	-4.06352900
H	-2.91387100	-0.64215300	-4.46244900
H	-4.12133600	-1.80287700	-3.85056100
C	-3.70883200	-1.06240400	-1.37559600
H	-4.69798900	-1.42503300	-1.63041500
C	-3.08601300	-1.46802800	-3.76538200
C	-2.73617900	-1.02235400	-2.37673000
O	-1.53966400	-0.63245400	-2.23064900
O	-2.36572100	-0.20056000	0.36636700
C	-4.56828800	-0.71358800	0.95384400
H	-5.50041000	-1.10807900	0.54524600
H	-4.74783000	0.28722100	1.36076300
H	-4.24385300	-1.34250000	1.78976200
C	-3.46630800	-0.64661500	-0.06205800
Ni	-0.73594400	-0.02476300	-0.63974900
C	1.91117000	-2.50702500	2.62409700

C	1.77439800	-1.33818200	1.88897700
C	0.54579400	-0.67774800	1.81376800
C	-0.53301500	-1.19339600	2.53650300
C	-0.38740400	-2.34914600	3.28636600
C	0.82951100	-3.01956200	3.32486800
H	2.87550500	-3.00548800	2.66440400
H	2.63971300	-0.92310900	1.37786800
H	-1.48553400	-0.67758500	2.49779200
H	-1.23407700	-2.73268400	3.84889400
H	0.93643700	-3.92767800	3.91059400
C	0.43761200	0.55699000	0.98488600
H	1.42257100	1.03141500	0.91331700
C	3.04462800	-2.62186700	-1.43324200
C	1.93290900	-1.81173200	-1.29044800
C	2.07072300	-0.41636200	-1.25354200
C	3.35228900	0.14499000	-1.36208800
C	4.46542300	-0.66761300	-1.48262400
C	4.30948500	-2.04973300	-1.51972800
H	2.93241700	-3.70081100	-1.46665400
H	0.93583200	-2.23627600	-1.20506600
H	3.45734000	1.22657000	-1.31461200
H	5.45641100	-0.23008500	-1.54933600
H	5.18326400	-2.68717300	-1.61956200
C	0.90767300	0.42611900	-1.09639200
H	1.11675800	1.47836200	-1.32412200

IN9’

H	-0.95223300	-2.76258400	3.21797400
H	0.39115900	-1.63759300	3.39542800
H	0.72848400	-3.36615300	3.15503600

C	0.82584700	-3.06251900	0.57084500
H	1.23621600	-3.95711100	1.03212800
C	0.06593400	-2.54613200	2.87801700
C	0.07351600	-2.28877300	1.40969500
O	-0.73612800	-1.29327600	1.12027900
O	1.22719100	-1.80229600	-1.41273400
C	1.66023900	-4.10317000	-1.54009000
H	0.98285500	-4.94712500	-1.38263600
H	2.64275100	-4.39450000	-1.15062400
H	1.76019800	-3.88402800	-2.60314800
C	1.19833100	-2.88361600	-0.80452900
H	1.51172200	3.68886300	1.51706200
H	2.14530300	4.22431300	-0.02901300
H	3.26084000	4.04475900	1.35061800
C	3.82897400	1.59409700	0.55899600
H	4.64582300	2.17470000	0.97219100
C	2.38238200	3.62511000	0.85660300
C	2.57619500	2.19973200	0.43897500
O	1.52892500	1.65137100	-0.01549900
O	3.21600100	-0.49947900	-0.34581100
C	5.45488800	-0.29473900	0.30964900
H	6.15823300	0.41359700	0.75148800
H	5.82613300	-0.60772000	-0.67116700
H	5.40631800	-1.19227600	0.93418600
C	4.07602400	0.27727400	0.15559300
Ni	1.35676700	-0.07874500	-0.63448200
C	-4.89210600	-0.61745400	-1.19607900
C	-3.51090100	-0.70624700	-1.24155400
C	-2.77173900	-0.93802700	-0.08224700
C	-3.44777900	-1.07857300	1.12355500

C	-4.83367100	-0.98360700	1.17074000
C	-5.56105000	-0.75442800	0.01393100
H	-5.45003400	-0.43403800	-2.10940800
H	-2.99329100	-0.58264500	-2.18994100
H	-2.88779000	-1.25005300	2.03692200
H	-5.34565700	-1.08781000	2.12297300
H	-6.64335200	-0.67890100	0.05256800
C	-1.26784700	-1.04599400	-0.21584000
H	-1.06478200	-1.94515600	-0.80752800
C	-1.62467600	3.75869600	-1.14198900
C	-1.22404900	2.47157300	-1.46866500
C	-1.08651000	1.48205700	-0.49426200
C	-1.37515500	1.83013600	0.82858300
C	-1.78392900	3.11224400	1.15892300
C	-1.90820300	4.08772500	0.17657300
H	-1.72097100	4.50844200	-1.92268600
H	-0.99628200	2.22331300	-2.50393100
H	-1.25162000	1.08816400	1.61294300
H	-2.00346200	3.35631200	2.19507100
H	-2.22706800	5.09272900	0.43663300
C	-0.58248500	0.14117900	-0.87461200
H	-0.67027500	0.00752700	-1.96239300

IN5’$_{iso}$

H	-4.99452300	-2.36175300	1.24657400
H	-4.04334000	-2.04968000	2.69376000
H	-4.07738800	-3.68932700	2.00757300
C	-1.99420400	-3.21451200	0.51647800
H	-2.30912100	-4.23347900	0.72497500
C	-4.06739500	-2.62686800	1.76388900

C	-2.88787300	-2.25721100	0.92933300
O	-2.89373100	-0.98012300	0.65743300
O	0.03270400	-2.10747300	-0.12436900
C	-0.07471900	-4.40178600	-0.53783700
H	-0.80550400	-5.02215400	-1.06302400
H	0.29346900	-4.97496100	0.32091800
H	0.76966900	-4.17887600	-1.19154100
C	-0.68626000	-3.12141000	-0.05211900
H	3.98413200	-0.43263900	1.93727800
H	3.16418300	-0.89101800	3.43084300
H	3.81184400	0.76173300	3.25241800
C	1.52658700	1.63329100	2.30949300
H	2.11831200	2.33005500	2.89241400
C	3.32491100	-0.06491100	2.73084600
C	2.01367700	0.32956200	2.12158300
O	1.42661700	-0.56953200	1.46366600
O	-0.47837300	1.37880000	1.06834200
C	-0.14411800	3.47753300	2.01112700
H	0.56057500	4.05980500	2.60770200
H	-1.11680400	3.45631900	2.51369800
H	-0.30290300	3.96864100	1.04503500
C	0.31887400	2.07157500	1.77594300
Ni	-0.23693900	-0.38036100	0.53984300
C	-2.66345500	2.63656400	-2.42422800
C	-2.26268400	1.37190500	-2.01829300
C	-2.51973300	0.91495500	-0.72490300
C	-3.21510500	1.75378000	0.14710300
C	-3.63066300	3.01006900	-0.26248300
C	-3.34953700	3.46399900	-1.54571500
H	-2.45454700	2.96965200	-3.43681700

H	-1.75557100	0.71651300	-2.72476600
H	-3.41398100	1.41621200	1.15920700
H	-4.17317700	3.64800200	0.42994300
H	-3.67258700	4.45120300	-1.86187000
C	-1.98797200	-0.39961000	-0.31079000
H	-1.94470600	-1.08837500	-1.16646800
C	4.65536300	-0.78091700	-1.00285500
C	3.36466600	-0.81339000	-1.50278400
C	2.56387500	0.33261100	-1.47033800
C	3.08254000	1.49776100	-0.89747600
C	4.37236500	1.52177700	-0.39344900
C	5.17070100	0.38624400	-0.45022300
H	5.26535600	-1.67880400	-1.03965700
H	2.96747700	-1.73901300	-1.91249500
H	2.46060200	2.38851500	-0.84897400
H	4.75676700	2.43663900	0.04873100
H	6.18388200	0.40758300	-0.06086900
C	1.21424600	0.35752800	-2.00112900
N	0.73129400	-0.65373900	-2.65146100
N	0.32116300	-1.55752200	-3.20602300
H	0.55270100	1.21353200	-1.90542900

TS4’$_{iso}$

H	5.02915500	-1.65516000	0.49863100
H	4.12465900	-2.13316100	-0.93425700
H	4.32440100	-3.29144100	0.40624400
C	2.08435100	-2.51768400	1.45593600
H	2.51602800	-3.50484500	1.60220700
C	4.17170700	-2.24179000	0.15537400
C	2.90573500	-1.69493700	0.73284700

O	2.82569000	-0.42176700	0.43014000
O	-0.04335100	-1.48404900	1.86362000
C	0.35396200	-3.56996100	2.88864400
H	1.09880000	-3.76400200	3.66716800
H	0.28926300	-4.47447000	2.27339600
H	-0.61800400	-3.37663000	3.34314300
C	0.76278100	-2.39673500	2.03226300
H	-3.77305900	-1.12748600	-2.02242900
H	-3.43037300	-2.70141800	-1.32009800
H	-3.22711300	-2.46281900	-3.07906800
C	-0.71449400	-1.72949300	-2.79438000
H	-0.98844400	-2.18720700	-3.73810500
C	-3.11253200	-1.99932500	-2.09725100
C	-1.70650900	-1.55850500	-1.81867800
O	-1.52189000	-1.04642300	-0.68346500
O	1.09373300	-0.83222500	-1.54731300
C	1.63535000	-1.66464600	-3.65566600
H	1.19128900	-2.08650700	-4.55927800
H	2.38197800	-2.36374900	-3.26407200
H	2.16399100	-0.73940400	-3.90747200
C	0.62035300	-1.37901500	-2.58632200
Ni	0.08566800	-0.24264200	-0.02985400
C	2.06354600	4.19924100	0.51158900
C	1.84467500	2.92428500	1.01708000
C	2.05323400	1.79000300	0.22889100
C	2.51690200	1.96837900	-1.07893500
C	2.74904800	3.23969300	-1.57674400
C	2.51657000	4.36250300	-0.78918800
H	1.89796500	5.06643000	1.14443600
H	1.52977400	2.80580000	2.05272400

H	2.67766400	1.09048300	-1.69760800
H	3.11119300	3.36012400	-2.59397000
H	2.69729600	5.35699500	-1.18547900
C	1.72456900	0.44236700	0.74971700
H	1.61397300	0.44335500	1.84323400
C	-4.76923600	0.19734500	0.94666900
C	-3.44254500	0.33686700	1.31980300
C	-2.58585500	1.14076200	0.57185000
C	-3.07168900	1.78551600	-0.56302100
C	-4.39831900	1.63654900	-0.93782300
C	-5.25451500	0.84608800	-0.18220200
H	-5.42853400	-0.43225500	1.53664300
H	-3.05952100	-0.21139100	2.17721400
H	-2.39835300	2.39619400	-1.15986800
H	-4.76609300	2.14548600	-1.82391200
H	-6.29432900	0.73348200	-0.47296900
C	-1.15364700	1.29040600	0.90650400
N	-0.85945200	1.19477500	2.21137200
N	-0.57521800	1.03440800	3.28297600
H	-0.61373800	2.13150900	0.46344600

IN6’$_{iso}$

H	-3.92292300	2.46941500	2.29949700
H	-3.46623400	2.88719600	0.65077900
H	-2.76903600	3.79447700	2.02756000
C	-0.74169600	2.16449700	2.14474800
H	-0.61759200	3.23463000	2.29342000
C	-3.08949600	2.81192200	1.67747700
C	-1.98480100	1.80742200	1.72678500
O	-2.43979300	0.65393500	1.27500900

O	0.46151600	0.21108000	2.77754500
C	1.75646700	2.11037200	2.19480600
H	1.67148400	3.18361600	2.38359600
H	2.04686100	1.98424200	1.14202000
H	2.54306700	1.68034300	2.81949800
C	0.45158700	1.37709600	2.40250100
H	2.98694600	0.90621900	-3.46130300
H	3.65039100	1.42002900	-1.91692800
H	3.06357500	2.64776000	-3.07975600
C	0.63975700	2.59170000	-2.03969100
H	0.93337100	3.51513400	-2.52541900
C	2.88518100	1.65301200	-2.66699100
C	1.53542600	1.51787900	-2.02924400
O	1.29817300	0.39379800	-1.50831500
O	-1.13110200	1.48835700	-0.90961000
C	-1.59537200	3.64742900	-1.65781100
H	-1.22763200	4.41149400	-2.34505900
H	-1.73969000	4.10063400	-0.66962800
H	-2.57247400	3.28730700	-1.99390400
C	-0.65269400	2.48876100	-1.52070500
Ni	-0.16418100	-0.02918300	-0.32995500
C	-3.31690800	-3.54924100	-0.58493300
C	-2.55384700	-2.72680400	0.23313100
C	-2.53298100	-1.34186500	0.04816200
C	-3.33239800	-0.80245100	-0.96699800
C	-4.10244600	-1.62210600	-1.77418700
C	-4.09278100	-3.00121600	-1.59560800
H	-3.32210500	-4.62220600	-0.41512800
H	-1.99670300	-3.16922000	1.05532400
H	-3.32850300	0.27349000	-1.11447200

H	-4.71633200	-1.18284100	-2.55552300
H	-4.69608800	-3.64130700	-2.23201000
C	-1.65081000	-0.46634100	0.86167800
H	-1.27258600	-0.94556500	1.76957800
C	4.49021900	-0.70785200	1.06550300
C	3.12255900	-0.93383400	1.12971700
C	2.46045500	-1.50049700	0.04526100
C	3.17793700	-1.84646500	-1.09577500
C	4.54325400	-1.61750900	-1.15615100
C	5.20456400	-1.04598400	-0.07607800
H	4.99937400	-0.26024300	1.91424700
H	2.55398100	-0.64594700	2.01237100
H	2.65598300	-2.27275600	-1.94843800
H	5.09434000	-1.88593300	-2.05255600
H	6.27439000	-0.86763200	-0.12401300
C	0.97782200	-1.70990700	0.07223100
N	0.60135700	-2.30411300	1.24477400
N	0.26907800	-2.68641100	2.23361200
H	0.57384500	-2.31699700	-0.74772500

TS5’$_{iso}$

H	-4.40489500	2.07310400	1.83775700
H	-3.79593100	2.55785400	0.25751800
H	-3.40643900	3.53857500	1.70267600
C	-1.21389600	2.19097000	2.08291400
H	-1.25719100	3.26847700	2.22612800
C	-3.54960600	2.52496100	1.32495100
C	-2.33944100	1.67127500	1.52426700
O	-2.58629500	0.46890300	1.04264400
O	0.20452400	0.43923300	2.86361900

C	1.23449100	2.52496400	2.40508200
H	0.98844600	3.52819100	2.76529600
H	1.53264200	2.62634800	1.35255000
H	2.07283800	2.12150800	2.97735500
C	0.04912400	1.58963600	2.47645400
H	3.03187100	1.21063900	-3.40824500
H	3.65174500	1.77994800	-1.86457900
H	2.98350100	2.95314400	-3.04072500
C	0.52916800	2.69141200	-2.10910900
H	0.74582200	3.60689300	-2.64762500
C	2.87542600	1.95232900	-2.61784400
C	1.53204000	1.72441900	-1.99137900
O	1.40654500	0.62581300	-1.38398800
O	-1.16320800	1.48023300	-0.97333100
C	-1.80339500	3.55174200	-1.83584900
H	-1.46447400	4.34657400	-2.50279800
H	-2.06795500	3.98958000	-0.86600800
H	-2.71131100	3.09431000	-2.24098700
C	-0.76315500	2.49315700	-1.61669700
Ni	-0.10322100	0.04547600	-0.31886300
C	-2.74869400	-3.92908300	-0.51395800
C	-2.17545600	-2.94935700	0.28665200
C	-2.29803900	-1.59489300	-0.03027500
C	-3.04437800	-1.24853400	-1.16228000
C	-3.62743000	-2.22443500	-1.95240400
C	-3.47601300	-3.57128100	-1.63918300
H	-2.64411500	-4.97596000	-0.24304100
H	-1.65052100	-3.24283800	1.19289100
H	-3.15071900	-0.19694800	-1.41355500
H	-4.20375800	-1.93468100	-2.82662100

H	-3.93234800	-4.33409300	-2.26260500
C	-1.61427600	-0.55018200	0.77495800
H	-1.25783400	-0.91250400	1.74097300
C	4.45838900	-0.31157300	0.88011200
C	3.09028200	-0.49406300	0.95844700
C	2.44984200	-1.38477000	0.09386100
C	3.20072000	-2.08678700	-0.84570900
C	4.56908800	-1.87594200	-0.94715400
C	5.19884600	-0.99295300	-0.08244700
H	4.95361100	0.37376800	1.56135900
H	2.49258400	0.04618200	1.69023800
H	2.70258700	-2.78053300	-1.51831500
H	5.14572100	-2.41160300	-1.69485800
H	6.27122700	-0.83723800	-0.15238100
C	0.97710900	-1.48563300	0.06904600
N	0.70125800	-2.18806000	1.68606500
N	0.68218300	-2.35430400	2.77074300
H	0.62400400	-2.37120600	-0.47817200

IN7’$_{iso}$

H	1.94937900	1.23847600	4.12786000
H	2.02061900	-0.37352400	3.41641300
H	0.78161500	0.00493000	4.65573700
C	-0.91164000	0.68845800	2.77917200
H	-1.24428100	0.13021100	3.65178800
C	1.33643200	0.39204900	3.80024000
C	0.43835000	0.83372200	2.69403500
O	1.16550600	1.32766200	1.70758900
O	-1.86769700	2.15600400	1.16103400
C	-3.35970500	0.72028400	2.31405400

H	-3.64604700	1.02756900	3.32677000
H	-3.36655000	-0.37517800	2.29479000
H	-4.09112400	1.11103300	1.60413100
C	-1.98742900	1.26175300	1.98677500
H	0.54807000	-4.13559500	-2.89614300
H	-0.51245700	-4.73691900	-1.62976200
H	1.18292000	-5.28993000	-1.69571000
C	2.00644600	-3.33518400	-0.16113000
H	2.59300700	-4.24667100	-0.16606900
C	0.51806700	-4.43705400	-1.84435600
C	0.88232400	-3.26124600	-0.98653300
O	0.10693200	-2.26543100	-1.08331200
O	1.82730000	-1.17248100	0.78345300
C	3.59629100	-2.47592500	1.57254300
H	4.12468800	-3.41253300	1.38516700
H	3.27596800	-2.45426000	2.61999700
H	4.27983200	-1.63312300	1.42703100
C	2.40075100	-2.29305000	0.68450300
Ni	0.34773500	-0.53102000	-0.25627000
C	2.87636200	2.33417300	-2.62404900
C	1.79537700	2.09564100	-1.79079000
C	1.97808400	1.59598600	-0.49740800
C	3.28342000	1.35936700	-0.05762400
C	4.36381500	1.60867800	-0.88936200
C	4.16869200	2.09050800	-2.17762500
H	2.70999100	2.72313000	-3.62445400
H	0.78852200	2.30625500	-2.14460900
H	3.43581400	0.97962700	0.94729600
H	5.37226800	1.42692600	-0.52767700
H	5.01794700	2.28311200	-2.82607200

C	0.78784300	1.30318300	0.33666600
H	-0.01830400	2.01886700	0.17889800
C	-4.01792300	-2.11663900	-0.23332400
C	-2.73334500	-1.61063300	-0.27226500
C	-2.47014300	-0.37829900	-0.90259100
C	-3.54566700	0.32982100	-1.47585700
C	-4.82931200	-0.17813700	-1.43342500
C	-5.06246100	-1.40243400	-0.81374500
H	-4.21581300	-3.06771400	0.25074800
H	-1.90396400	-2.14877800	0.17417700
H	-3.34547300	1.29090100	-1.94210400
H	-5.65244800	0.37339100	-1.87592100
H	-6.07163800	-1.80298700	-0.77927600
C	-1.15111700	0.17011800	-0.95135400
N	-1.84829100	3.72080600	-1.45923500
N	-1.67236900	4.61573500	-0.85456300
H	-1.14188100	1.13869700	-1.47029700

IN8’$_{iso}$

H	-3.50774100	0.84152900	3.27256800
H	-3.57896500	1.62367200	1.69484300
H	-2.84155500	2.47721100	3.08610200
C	-0.48444900	1.50120200	2.55578300
H	-0.60585900	2.51288900	2.93812100
C	-2.96616600	1.51051200	2.59517400
C	-1.65599200	0.89046200	2.23442200
O	-1.87944000	-0.24160500	1.59563500
O	1.25328900	-0.13047400	2.55041700
C	1.91847600	2.14908100	2.50721000
H	1.76453800	2.82959000	3.35157100

H	1.80625600	2.74915300	1.59458200
H	2.92582200	1.73132000	2.55886100
C	0.89215600	1.03571400	2.52134100
H	1.45371200	2.62269300	-4.09396500
H	2.44057700	3.14917100	-2.73768600
H	1.15436100	4.21842100	-3.36186000
C	-0.65940000	3.19569100	-1.77497900
H	-0.81371700	4.21064400	-2.12307800
C	1.42902900	3.18262900	-3.15366200
C	0.47779900	2.51000800	-2.20788200
O	0.79525100	1.33210400	-1.87391900
O	-1.60299500	1.44611400	-0.48759700
C	-2.83065000	3.43569100	-0.56158900
H	-2.89799300	4.37724100	-1.10971600
H	-2.76832800	3.65517200	0.51087800
H	-3.74248000	2.85194400	-0.71898600
C	-1.63461000	2.61911300	-0.95422200
Ni	-0.15936400	0.19515500	-0.63292500
C	-1.86109000	-4.39269300	-0.51282600
C	-1.18956000	-3.36462700	0.13621700
C	-1.70232200	-2.06704700	0.14620900
C	-2.91985500	-1.82760400	-0.49460900
C	-3.59556100	-2.85524700	-1.13221100
C	-3.06743000	-4.14169100	-1.15026000
H	-1.44878900	-5.39774400	-0.50401700
H	-0.25933000	-3.57134400	0.66414200
H	-3.32443500	-0.81916700	-0.48687100
H	-4.54206300	-2.65266300	-1.62549700
H	-3.59849100	-4.94453900	-1.65258000
C	-0.94179700	-0.96366400	0.79198200

H	-0.14425300	-1.33367500	1.44382600
C	1.02789300	-1.13233400	-0.78525400
C	2.40481300	-1.18480800	-0.44476200
C	3.08000000	-0.03647400	0.01167600
C	3.11303300	-2.40133300	-0.52897800
C	4.41214100	-0.10362800	0.36314700
H	2.52728700	0.89526500	0.06501700
C	4.44166100	-2.46936400	-0.16310700
H	2.58814800	-3.28680700	-0.87912700
C	5.08731800	-1.31810600	0.28058700
H	4.93166500	0.78293100	0.71252500
H	4.98148400	-3.40875800	-0.21944400
H	6.13361900	-1.36964900	0.56809000
H	0.64727300	-2.08685900	-1.17913200

TS6’$_{iso}$

H	0.48169400	0.90845500	3.84775100
H	0.33955400	-0.64047100	3.03244900
H	-0.97964300	-0.08031500	4.11168000
C	-2.19083000	0.97507200	2.03532100
H	-2.76053700	0.44148900	2.79304000
C	-0.23580000	0.22836900	3.37598300
C	-0.84709800	0.90218900	2.19506700
O	0.11518300	1.38871800	1.41208700
O	-2.55800100	2.51736300	0.25722700
C	-4.48300100	1.52528400	1.22539800
H	-4.81781600	1.83618600	2.22147500
H	-4.72767800	0.46191000	1.11868500
H	-5.01168500	2.10395600	0.46674500
C	-2.99545200	1.73537100	1.08600000

H	2.20439400	-4.30467200	-2.30150200
H	1.10144400	-4.97235400	-1.10642900
H	2.86139300	-5.07010800	-0.83167000
C	2.90112700	-2.78124200	0.43547900
H	3.66638600	-3.50572100	0.68937500
C	2.05515400	-4.44962000	-1.22703100
C	1.97501000	-3.11050900	-0.55621900
O	1.03327800	-2.36767600	-0.96550200
O	2.04293000	-0.63675200	0.93027100
C	3.90160800	-1.28511200	2.18427600
H	4.58460200	-2.12092700	2.34717300
H	3.39280700	-1.04147100	3.12292800
H	4.47674400	-0.39868800	1.89495200
C	2.87768600	-1.56238500	1.12369500
Ni	0.66453200	-0.60379400	-0.40549100
C	2.42766900	3.11628800	-2.39198100
C	1.31313600	2.49166500	-1.85491900
C	1.31863400	2.01709400	-0.54152800
C	2.46396000	2.21422000	0.23257900
C	3.57236100	2.85046700	-0.30392100
C	3.56620500	3.29703900	-1.61871800
H	2.40008700	3.47788800	-3.41561800
H	0.41678400	2.38855900	-2.46204900
H	2.47605900	1.85677800	1.25524000
H	4.45274900	3.00143100	0.31472700
H	4.43792300	3.79292600	-2.03448700
C	0.10645000	1.33117400	-0.00597900
H	-0.79375600	1.83855900	-0.37407400
C	-0.78438600	-0.11657200	-1.29417600
C	-2.13632300	-0.59287200	-1.08398500

C	-2.39547900	-1.62248300	-0.16558500
C	-3.19476800	-0.10581600	-1.86419000
C	-3.66682000	-2.14906300	-0.03932300
H	-1.57031700	-2.00298100	0.43153200
C	-4.46968900	-0.63146200	-1.73711400
H	-3.00094300	0.70019300	-2.56726500
C	-4.70375000	-1.65487800	-0.82709300
H	-3.85659100	-2.95236800	0.66566000
H	-5.28183700	-0.24627000	-2.34543100
H	-5.70155100	-2.07305600	-0.73010400
H	-0.71788700	0.56076700	-2.15591000

IN9’$_{iso}$

H	-0.24302500	-3.88614100	3.21584800
H	1.50984300	-3.55663200	3.06757800
H	0.72873700	-4.83342100	2.09512500
C	-0.87211700	-3.10015800	0.67289600
H	-1.41268000	-3.97754600	1.01977400
C	0.60606700	-3.83379300	2.52586500
C	0.33006200	-2.83625800	1.43763400
O	1.15334000	-1.92953100	1.29325800
O	-0.86824100	-1.49930600	-1.11689300
C	-2.55274400	-3.15701100	-1.14106100
H	-3.39282300	-2.45123100	-1.13493400
H	-2.86546600	-4.09633800	-0.68224200
H	-2.29873400	-3.32882700	-2.19241400
C	-1.37915800	-2.54284200	-0.45724400
H	2.28436600	3.48964100	-1.52466600
H	1.87054700	2.61262500	-2.99285400
H	3.57668300	3.00956600	-2.66186000

C	3.94089300	0.72444500	-1.42086300
H	4.79100700	1.14683600	-1.94415900
C	2.62994500	2.70842300	-2.20981700
C	2.73229000	1.41558400	-1.45815700
O	1.64966800	1.05357200	-0.90192700
O	3.20190100	-1.09495700	-0.10723500
C	5.42824600	-1.20139400	-0.82064600
H	6.17582300	-0.63992000	-1.38422100
H	5.29561700	-2.18642500	-1.27882400
H	5.79178800	-1.36982900	0.19757200
C	4.10045700	-0.50416300	-0.76122800
Ni	1.41922000	-0.42494000	0.19083500
C	-4.78540600	0.21703300	0.38218800
C	-3.40359000	0.09228600	0.45133400
C	-2.63651600	0.10701500	-0.70903100
C	-3.27491400	0.27373300	-1.93649600
C	-4.65305200	0.40232500	-2.00978400
C	-5.41284400	0.36786900	-0.84648600
H	-5.37433300	0.20559800	1.29442500
H	-2.91687100	-0.00920300	1.41751800
H	-2.67447700	0.28658600	-2.84416200
H	-5.13717100	0.53241300	-2.97307400
H	-6.49263300	0.47015800	-0.89778100
C	-1.14226000	-0.11024400	-0.69418500
H	-0.70058500	0.44918500	-1.52622500
C	-0.39380500	0.19746600	0.56829200
C	-0.39618100	1.60945700	1.01309900
C	-0.61881100	2.69774400	0.16252000
C	-0.13075500	1.88857100	2.35969400
C	-0.56755400	4.00053500	0.63714500

H	-0.83038800	2.53225500	-0.89022300
C	-0.07704200	3.18774100	2.83524500
H	0.05105000	1.05568100	3.03734500
C	-0.29499100	4.25635300	1.97381000
H	-0.74782800	4.82585600	-0.04657100
H	0.13551100	3.36928300	3.88514100
H	-0.25767500	5.27696400	2.34233400
H	-0.75511900	-0.43135200	1.39217700

TS7'

H	4.36286100	-2.39281100	1.49956100
H	3.67997500	-2.40969200	-0.12641800
H	3.19394000	-3.65550600	1.06839700
C	1.25819800	-2.16602300	2.02315600
H	1.12002800	-3.24468400	2.00295200
C	3.45283600	-2.60430900	0.92668800
C	2.36202100	-1.69888800	1.38599300
O	2.64651500	-0.44232900	1.07848300
O	0.53523200	-0.35080700	3.36093700
C	-1.04886700	-2.08378300	2.99617500
H	-0.93945700	-3.12072000	3.33089400
H	-1.56947800	-2.11467600	2.03001800
H	-1.64768900	-1.52865700	3.72221800
C	0.29636800	-1.42102400	2.81682400
H	-3.36244400	-1.16935800	-2.75825500
H	-3.45331100	-2.47406400	-1.58753900
H	-2.84857000	-2.81789700	-3.23063700
C	-0.36699900	-2.39214100	-2.43410300
H	-0.50176600	-3.14564100	-3.20162800
C	-2.86150600	-2.08389900	-2.42259000

C	-1.49089400	-1.73508000	-1.92582600
O	-1.45666300	-0.83394800	-1.04333100
O	1.24297100	-1.26810300	-1.10186800
C	2.07988000	-2.88443700	-2.56354700
H	1.81085000	-3.41990800	-3.47601800
H	2.42909100	-3.61350500	-1.82327000
H	2.91108100	-2.20198600	-2.76598800
C	0.92647100	-2.11591200	-1.98726000
Ni	0.08397800	-0.11802600	-0.14869000
C	2.43915800	3.96052500	-0.58993200
C	1.95920400	2.95806200	0.24117300
C	2.24495100	1.61233000	-0.00648000
C	3.05826800	1.30338200	-1.10215500
C	3.54843500	2.30522200	-1.92339600
C	3.23563100	3.63795000	-1.67959900
H	2.20929100	4.99956200	-0.37148100
H	1.38012000	3.22833800	1.12341000
H	3.29121300	0.26114800	-1.30057200
H	4.18126000	2.04571200	-2.76758400
H	3.62108000	4.41932600	-2.32732800
C	1.64457700	0.55473400	0.84169900
H	1.33366300	0.94114200	1.81754900
C	-4.39540500	1.59227600	-1.38542500
C	-3.07418700	1.78314700	-1.01105000
C	-2.59424200	1.22839700	0.17027000
C	-3.45272100	0.49040500	0.97828000
C	-4.77590300	0.30915000	0.60741700
C	-5.25134900	0.85552300	-0.57759600
H	-4.75959400	2.02622900	-2.31181700
H	-2.39990200	2.34750300	-1.65045500

H	-3.07913600	0.02872100	1.89045800
H	-5.43824400	-0.26950500	1.24404000
H	-6.28678200	0.70966700	-0.86882500
C	-1.14422300	1.38031400	0.49757400
N	-0.98820500	1.65413100	1.84131700
N	-0.86880400	1.79395500	2.93546300
H	-0.63306400	2.18535900	-0.04735300

References

1. Darbeau, R. W.; White, E. H.; Song, F.; Darbeau, N. R.; Chou, J., A Study of Essentially Free Carbocations Derived via Diazonium and Oxo Diazonium Ions in the Liquid Phase. *J. Org. Chem.* **1999,** *64*, 5966-5978.
2. Green, S. P.; Wheelhouse, K. M.; Payne, A. D.; Hallett, J. P.; Miller, P. W.; Bull, J. A., Thermal Stability and Explosive Hazard Assessment of Diazo Compounds and Diazo Transfer Reagents. *Org. Process Res. Dev.* **2020,** *24*, 67-84.
3. Gaussian 16, Revision C.02, M. J. Frisch, G. W. Trucks, H. B. Schlegel, G. E. Scuseria, M. A. Robb, J. R. Cheeseman, G. Scalmani, V. Barone, G. A. Petersson, H. Nakatsuji, X. Li, M. Caricato, A. V. Marenich, J. Bloino, B. G. Janesko, R. Gomperts, B. Mennucci, H. P. Hratchian, J. V. Ortiz, A. F. Izmaylov, J. L. Sonnenberg, D. Williams-Young, F. Ding, F. Lipparini, F. Egidi, J. Goings, B. Peng, A. Petrone, T. Henderson, D. Ranasinghe, V. G. Zakrzewski, J. Gao, N. Rega, G. Zheng, W. Liang, M. Hada, M. Ehara, K. Toyota, R. Fukuda, J. Hasegawa, M. Ishida, T. Nakajima, Y. Honda, O. Kitao, H. Nakai, T. Vreven, K. Throssell, J. A. Montgomery, Jr., J. E. Peralta, F. Ogliaro, M. J. Bearpark, J. J. Heyd, E. N. Brothers, K. N. Kudin, V. N. Staroverov, T. A. Keith, R. Kobayashi, J. Normand, K. Raghavachari, A. P. Rendell, J. C. Burant, S. S. Iyengar, J. Tomasi, M. Cossi, J. M. Millam, M. Klene, C. Adamo, R. Cammi, J. W. Ochterski, R. L. Martin, K. Morokuma, O. Farkas, J. B. Foresman, and D. J. Fox, Gaussian, Inc., Wallingford CT, 2019.
4. Legault, C. Y., CYLView. CYLView 2009, 1.0b, http://www.cylview.org
